# Pfh1 Is an Accessory Replicative Helicase that Interacts with the Replisome to Facilitate Fork Progression and Preserve Genome Integrity

**DOI:** 10.1371/journal.pgen.1006238

**Published:** 2016-09-09

**Authors:** Karin R. McDonald, Amanda J. Guise, Parham Pourbozorgi-Langroudi, Ileana M. Cristea, Virginia A. Zakian, John A. Capra, Nasim Sabouri

**Affiliations:** 1 Department of Molecular Biology, Princeton University, Princeton, New Jersey, United States of America; 2 Department of Medical Biochemistry and Biophysics, Umeå University, Umeå, Sweden; 3 Department of Biological Sciences, Vanderbilt Genetics Institute, and Center for Structural Biology, Vanderbilt University, Nashville, Tennessee, United States of America; CSIC/Universidad de Salamanca, SPAIN

## Abstract

Replicative DNA helicases expose the two strands of the double helix to the replication apparatus, but accessory helicases are often needed to help forks move past naturally occurring hard-to-replicate sites, such as tightly bound proteins, RNA/DNA hybrids, and DNA secondary structures. Although the *Schizosaccharomyces pombe* 5’-to-3’ DNA helicase Pfh1 is known to promote fork progression, its genomic targets, dynamics, and mechanisms of action are largely unknown. Here we address these questions by integrating genome-wide identification of Pfh1 binding sites, comprehensive analysis of the effects of Pfh1 depletion on replication and DNA damage, and proteomic analysis of Pfh1 interaction partners by immunoaffinity purification mass spectrometry. Of the 621 high confidence Pfh1-binding sites in wild type cells, about 40% were sites of fork slowing (as marked by high DNA polymerase occupancy) and/or DNA damage (as marked by high levels of phosphorylated H2A). The replication and integrity of tRNA and 5S rRNA genes, highly transcribed RNA polymerase II genes, and nucleosome depleted regions were particularly Pfh1-dependent. The association of Pfh1 with genomic integrity at highly transcribed genes was S phase dependent, and thus unlikely to be an artifact of high transcription rates. Although Pfh1 affected replication and suppressed DNA damage at discrete sites throughout the genome, Pfh1 and the replicative DNA polymerase bound to similar extents to both Pfh1-dependent and independent sites, suggesting that Pfh1 is proximal to the replication machinery during S phase. Consistent with this interpretation, Pfh1 co-purified with many key replisome components, including the hexameric MCM helicase, replicative DNA polymerases, RPA, and the processivity clamp PCNA in an S phase dependent manner. Thus, we conclude that Pfh1 is an accessory DNA helicase that interacts with the replisome and promotes replication and suppresses DNA damage at hard-to-replicate sites. These data provide insight into mechanisms by which this evolutionarily conserved helicase helps preserve genome integrity.

## Author Summary

Progression of the DNA replication machinery is challenged in every S phase by active transcription, tightly bound protein complexes, and formation of stable DNA secondary structures. Using genome-wide analyses, we show that the evolutionarily conserved fission yeast Pfh1 DNA helicase promotes fork progression and suppresses DNA damage at natural sites of fork pausing, which occur at “hard-to-replicate” sites. Our data suggest that Pfh1 interacts with the replication apparatus. First, mass spectrometry revealed that Pfh1 interacts with many components of the replication machinery. Second, Pfh1 and the leading strand DNA polymerase occupy many common regions genome-wide, not only hard-to-replicate sites, but also sites whose replication is not Pfh1-dependent. The human genome encodes a Pfh1 homolog, hPIF1, and contains all of the same hard-to-replicate features that make fission yeast DNA replication dependent upon Pfh1. Thus, human cells likely also require replicative accessory DNA helicases to facilitate replication at hard-to-replicate sites, and hPIF1 is a good candidate for this role.

## Introduction

Faithful and efficient replication of the genome is essential in every cell cycle, yet there are many naturally occurring obstacles that impede fork progression. These sites include stable protein complexes, DNA secondary structures, and ongoing transcription, each of which can challenge replication fork progression. Failure to circumvent these obstacles can cause DNA double strand breaks (DSBs) that impair genome integrity and increase the risk of cancer and other disorders that are associated with genome instability. As many of the proteins involved in DNA replication are highly conserved, model organisms such as *S*. *pombe* provide genetically tractable systems to identify and characterize genes with important roles in genome preservation whose human orthologs might have similar functions.

DNA replication is accomplished by the multi-subunit replisome, a complex that is assembled at and moves bi-directionally away from replication origins. Replicative helicases, such as the *Escherichia coli* DnaB and the eukaryotic hexameric MCM complex, are required to unwind the double helix to allow DNA polymerases access to the replication template. In addition, accessory helicases, such as the *E*. *coli* rep, dinG and UvrD proteins help the polymerase maneuver past protein complexes, RNA transcripts, and other naturally occurring impediments [1–5]. In *Bacillus subtilis* the essential accessory DNA helicase PcrA promotes fork movement through transcribed genes [6, 7]. *E*. *coli* rep physically interacts with the replicative DnaB helicase to bypass protein complexes on DNA [8].

The best-studied eukaryotic accessory DNA helicases are the two budding yeast enzymes, ScRrm3 and ScPif1, which are both members of the Pif1 family [9]. Although these two helicases have largely non-overlapping functions, they both promote progression past naturally occurring hard-to-replicate sites. ScRrm3 acts at over 1000 sites, including RNA polymerase III transcribed genes, the replication fork barrier (RFB) within ribosomal DNA (rDNA), inactive replication origins, silencers, telomeres, centromeres, and converged replication forks [10–14]. The diverse ScRrm3-sensitive sites have the common feature of being assembled into stable protein complexes. Removal of these proteins in *trans* or mutation of their binding sites in *cis* relieves the requirement for ScRrm3 at the affected site. In its absence, forks tend to stall and break at ScRrm3-sensitive sites. Rather than being recruited to its sites of action, ScRrm3 moves with the replisome through both ScRrm3-sensitive and insensitive sites [15] and interacts with leading strand DNA polymerase ε and PCNA [15, 16], suggesting that it is a replisome component.

ScPif1 also promotes fork progression, but so far this activity has been observed only at putative G-quadruplex (G4) structures [17–21]. G4 DNA is a stable, four-stranded secondary structure held together by non-canonical G-G base pairs [reviewed in 22]. In cells lacking ScPif1, DNA replication slows and DNA damage is detected at many G4 motifs. Current evidence suggests that ScPif1 is recruited to G4 motifs after their replication [18], and the abundance of ScPif1, unlike that of ScRrm3, is cell cycle regulated, peaking in late S phase [15, 23]. Thus, unlike ScRrm3, ScPif1 probably does not move with the leading strand DNA polymerase. ScRrm3 is a backup helicase for ScPif1 at G4 motifs [17]. As ScRrm3 and ScPif1 are both associated with stalled replication forks [24], ScPif1 might be a backup for ScRrm3 at some of its targets, such as RNA polymerase III transcribed genes. ScPif1 actions are not limited to its being an accessory DNA helicase, as it also inhibits telomerase by displacing it from DNA ends [25, 26], promotes formation of long flap Okazaki fragments [27, 28], and is needed for the stable maintenance of mitochondrial DNA [29] and break-induced replication [30–33].

Unlike budding yeast, most eukaryotes, including fission yeast and humans, encode a single Pif1 family helicase. While neither ScRrm3 nor ScPif1 is essential, the fission yeast Pfh1 DNA helicase is required for maintenance of both the mitochondrial and nuclear genomes [34]. Pfh1 also affects nuclear DNA repair: it localizes to DNA damage foci upon exogenous DNA damage, and its absence results in spontaneous DNA damage foci [34].

In earlier work, we and others used two-dimensional gels to show that Pfh1, like ScRrm3 promotes fork progression through specific stable protein complexes, including RNA polymerase II and III transcribed genes, silencers, converged replication forks [35, 36], and telomeres [37]. In addition, microscopic studies show that Pfh1 suppresses formation of ultrafine anaphase bridges that arise at incompletely replicated regions, such as Lac repressor bound *LacO* arrays [38], supporting the idea that it is needed to complete DNA replication. Like ScPif1, Pfh1 binds to and suppresses DNA damage at G4 motifs [39]. Also, ScPif1 and Pfh1 both unwind G4 structures *in vitro* [17, 40–43].

In this paper, we address two fundamental questions about Pfh1 function: where does Pfh1 act along the genome and how is it recruited to its sites of action? Earlier studies focused on Pfh1’s role in replication of a few examples of hard-to-replicate sites [35, 37, 39]. Here we used chromatin immunoprecipitation combined with genome-wide deep sequencing (ChIP-seq) to study the full range of Pfh1-sensitive sites. This analysis revealed hundreds of sites of Pfh1 binding where replication slows and DNA damage occurs, especially in the absence of Pfh1. These Pfh1-sensitive sites included all previously identified hard-to-replicate sites as well as novel sites, such as nucleosome depleted regions (NDR). Second, we assayed binding and fork progression to determine if Pfh1 is nearby the replisome during S phase or, if it is recruited to its sites of action. These analyses revealed that Pfh1 and the leading strand DNA polymerase bind both Pfh1-sensitive and Pfh1-insensitive sites to a similar extent. Likewise, mass spectrometry (MS) found that Pfh1 interacts with many key replisome components. Together these data argue that Pfh1 is not just recruited to its sites of action, but that it is in proximity to the replisome during DNA synthesis and functions as an accessory DNA helicase at all known classes of hard-to-replicate sites. These results inform our understanding of Pif1 helicases in higher eukaryotes, such as humans, which like *S*. *pombe*, encode only one Pif1 family helicase. Given that the helicase domains of Pfh1 and hPIF1 are related (36% sequence identity) [25], hPIF1 may have similar functions in preserving genome integrity.

## Results

### Many sites in the *S*. *pombe* genome display high Pfh1 occupancy

We analyzed 621 previously identified Pfh1 binding sites across the *S*. *pombe* genome from ChIP-seq on cycling WT cells (S1 Table) [39]. Given the low coverage of rDNA repeats and telomeres in the *S*. *pombe* annotated genome, the rest of this paper considers only non-telomeric, non-rDNA Pfh1 binding sites.

We determined if the peaks of Pfh1 binding correlated with any of sixteen annotated genomic features (Table 1; Methods). Because Pfh1 bound preferentially to GC-rich sites, we determined the significance of its association with features after accounting for GC content using random GC-matched controls (Methods). Pfh1 peaks were significantly associated with many known hard-to-replicate sites, such as tRNA genes, 5S ribosomal RNA (rRNA), and highly active RNA polymerase II transcribed genes (Table 1). For example, Pfh1 binding occurred ≤ 300 base pairs (bp), the shearing size of the ChIP DNA, from 80 out of 171 (47%) tRNA genes, 18 of 33 (55%) 5S rRNA genes, and 302 of the top 500 (60%) most highly transcribed RNA polymerase II genes (as defined in Rhind, Chen (44)). In addition, Pfh1 binding sites were significantly associated with meiotic double strand break (DSB) hotspots, nucleosome depleted regions (NDRs), 3’ untranslated regions (UTRs), and mating type loci (Table 1). In all following association analyses, associations with p-values less than the Bonferroni multiple testing adjusted threshold of 0.003 will be interpreted as significant.

**Table 1:** Genomic features associated with Pfh1 binding sites. Association p-values were computed with an empirical permutation-based procedure that accounts for length and GC content (Methods). Associations significant after Bonferroni multiple testing correction (p < 0.003) are in bold. All p-values less than 10^−50^ are reported as ≈0.

Genome Features	Number of Features	Fraction with Pfh1	p-value
500 Highest Expressed RNA polymerase II Genes	500	0.60 (302)	**≈ 0**
5S rRNA genes	33	0.55 (18)	**≈ 0**
tRNA genes	171	0.47 (80)	**≈ 0**
Meiotic Double Strand Break Hotspots	288	0.37 (106)	**≈ 0**
3’ UTRs	5144	0.18 (939)	**≈ 0**
Nucleosome Depleted Regions	2300	0.18 (406)	**≈ 0**
Mating Type Loci	5	0.40 (2)	**0.002**
G4 motifs*	446	0.20 (90)	**0.002**
RNA Pol II Transcribed Genes	5144	0.23 (1188)	0.005
Origins of Replication	741	0.23 (170)	0.014
Promoters	3237	0.16 (504)	0.042
Protein Coding Sequence	10244	0.14 (1459)	0.093
Dubious Genes	71	0.21 (15)	0.112
5’ UTRs	5144	0.13 (693)	0.139
Long Terminal Repeat	236	0.07 (16)	0.982
Centromeres	3	0.33 (1)	0.999

* from Sabouri, Capra [39].

### Pfh1 binding to highly transcribed genes is not an artifact of their high transcription

A recent study reported that highly transcribed *S*. *cerevisiae* genes are over-represented in ChIP experiments carried out with diverse nuclear proteins, suggesting that their presence might be a technical artifact caused by their high transcription rate [45]. The specific cause of the “hyper-ChIPability” of these regions has not been resolved. It has been proposed that, because highly transcribed genes are more accessible during the pull-down, they may be more likely to interact with beads or antibodies during the IP, and therefore be subject to nonspecific precipitation by the antibody.

To ensure that the association with highly transcribed RNA polymerase II genes was not due to this artifact, we used ChIP combined with quantitative PCR (qPCR) to examine Pfh1 association to four highly transcribed genes, *hsp90*^*+*^, *tdh1*^*+*^, *adh1*^*+*^, and *hta1*^*+*^, which are among the top 500 most highly transcribed genes and were all Pfh1-associated sites in the genome-wide analysis. Transcription of *hsp90*^*+*^, *tdh1*^*+*^, *adh1*^*+*^ occurs throughout the cell cycle, while *hta1*^*+*^ transcription peaks in S phase [46]. In fission yeast, the G2 phase comprises about 75% of the cell cycle, so the majority of cells in an asynchronous culture are in G2 phase, and most genes are transcribed in this interval [47]. Thus, if the association of Pfh1 with highly transcribed genes was non-specific, it should occur to a similar extent in asynchronous and G2-arrested cells for *hsp90*^*+*^, *tdh1*^*+*^, *adh1*^*+*^, but not *hta1*^*+*^.

We performed ChIP-qPCR experiments in both asynchronous and G2 arrested cells in a temperature sensitive *cdc25-22* strain that expressed Pfh1-13Myc. Pfh1 was significantly associated to all four genes in asynchronous cells grown at 25°C, compared to an untagged control strain (S1 Fig), which validated the peaks found in the ChIP-seq data. To arrest the cells in G2 phase, cells growing logarithmically at 25°C were shifted to 37°C for 4h.

**Fig 1:**
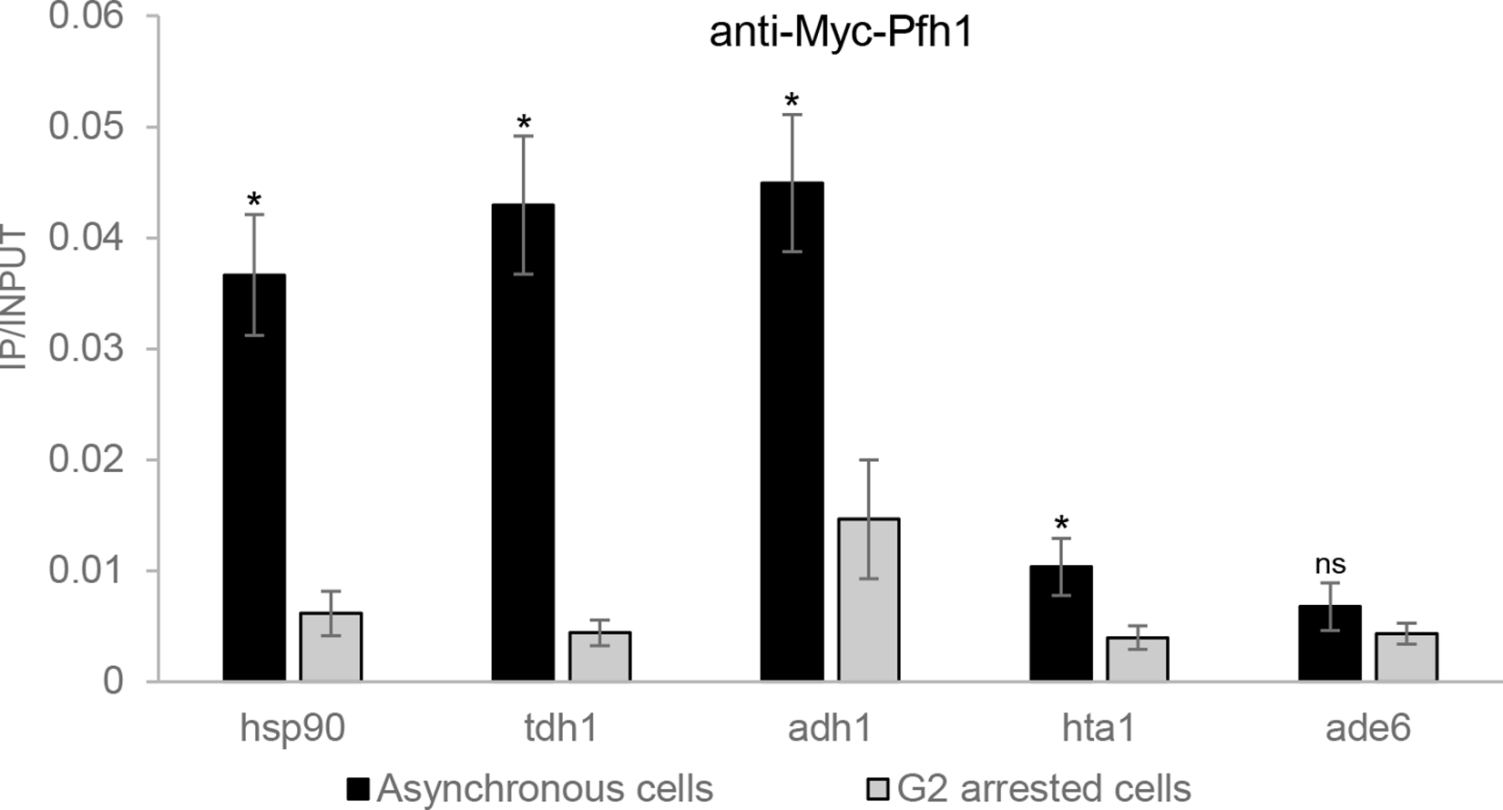
Pfh1 is enriched at highly transcribed genes in asynchronous cells compared to G2 arrested cells. Samples from asynchronous or G2 arrested cells expressing Pfh1-13Myc were chromatin immunoprecipitated using an anti-Myc antibody. The immunoprecipitated DNA was analyzed using quantitative PCR with primers specific for four highly transcribed genes, *hsp90*^*+*^, *tdh1*^*+*^, *adh1*^*+*^, *hta1*^*+*^, and a low/medium transcribed control gene, *ade6*^*+*^. Pfh1 association is shown as immunoprecipitated DNA divided by input DNA. Data are means of three independent replicates. Error bars are the standard deviation. The p-value was determined by two-tailed Student’s t-test. An “*” indicates significant (p < 0.05), and “ns” indicates a non-significant difference between asynchronous cells compared to G2 arrested cells.

Consistent with the expectation in the absence of bias, high Pfh1 binding to all four of the highly transcribed genes varied across the cell cycle; it was approximately four times higher in asynchronous compared to G2 arrested cells in the ChIP-qPCR assay (p ≤ 0.016; Fig 1). In contrast, Pfh1 binding to the much less frequently transcribed *ade6*^*+*^ gene was not significantly different in asynchronous versus G2 arrested cells (p = 0.14, Fig 1). Thus, the ChIP-qPCR experiment confirmed the high Pfh1 binding to these highly transcribed genes seen by ChIP-seq and established that this binding is not an artifact of the ChIP.

### Replisome pausing is increased genome-wide at hard-to-replicate sites in Pfh1-depleted cells

**Fig 2:**
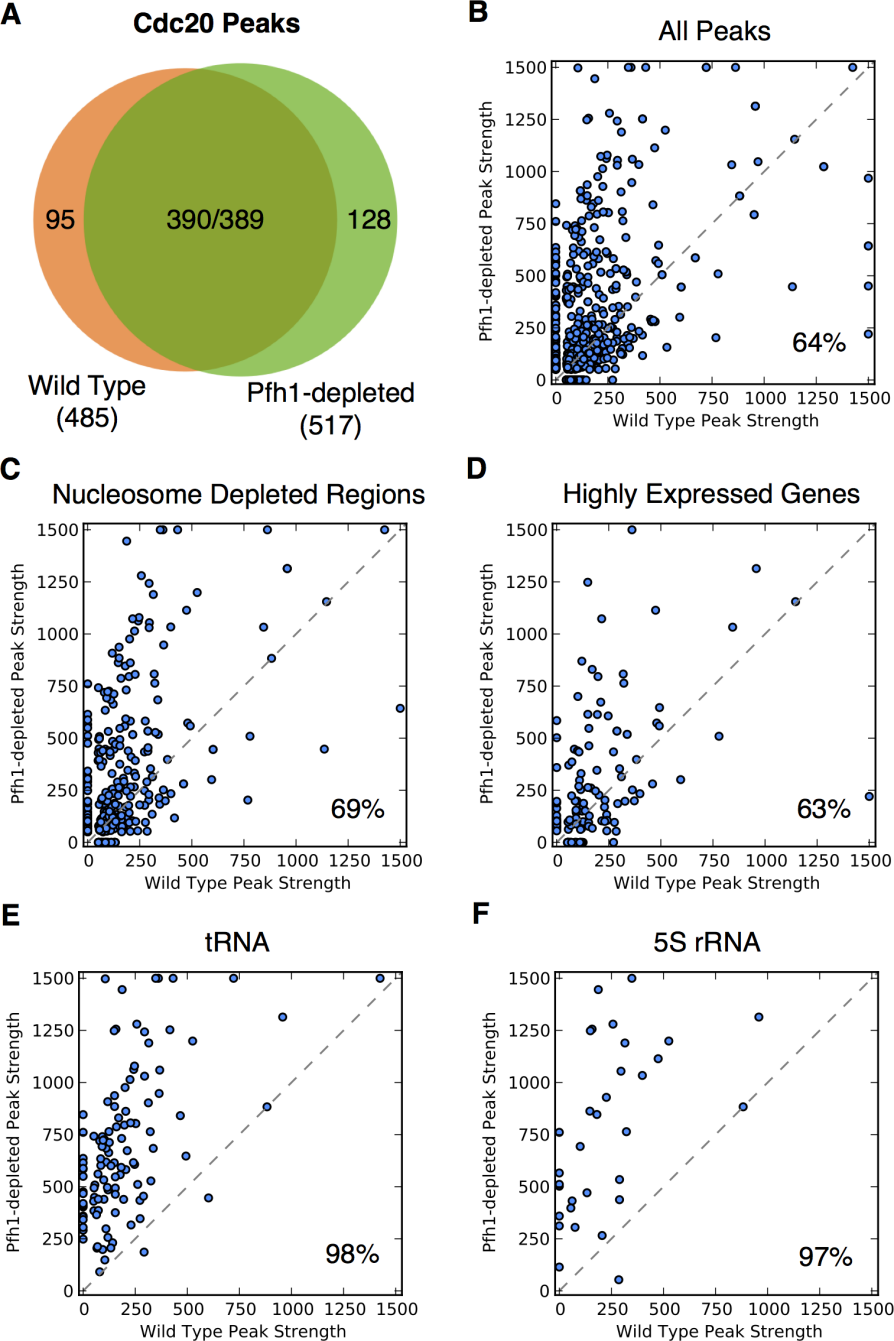
Fork pausing as marked by Cdc20 occupancy is increased in the absence of Pfh1. (A) Venn diagram showing the overlap of genome-wide Cdc20 peaks in the presence and absence of Pfh1. The two numbers in the intersection are the number of WT peaks that overlap a Pfh1-depleted peak and vice versa. (B) Scatter plot comparing Cdc20 peak strength (-10*log_10_(p-value)) in WT and Pfh1-depleted cells. Each point represents a genomic region with a Cdc20 occupancy peak in at least one context. If a peak was not present in a context, it is plotted at 0 on the corresponding axis. The number in the bottom right of each plot gives the percentage of peaks stronger in Pfh1-depleted cells. When all peaks were considered, 64% of peaks were significantly stronger in Pfh1-depleted cells (p ≈ 0, Wilcoxon signed-rank test). Likewise, peaks at most tested genomic features were significantly stronger in Pfh1-depleted cells (S3 Table), as shown for (C) NDRs (69%, p ≈ 0) and (D) highly expressed genes (63%, p = 2.4x10^-5^). The difference was particularly large for (E) tRNA (98%, p ≈ 0) and (F) 5S rRNA genes (97%, p = 1.4x10^-6^).

To identify genomic sites that require Pfh1 for their timely replication, we analyzed genome-wide occupancy of Cdc20, the catalytic subunit of the leading strand DNA polymerase ε [48] in WT and Pfh1-depleted cells. As previously reported [39], there are 485 peaks of high Cdc20 occupancy in WT cells and 517 in Pfh1-depleted cells (S1 Table). Although all genomic sites are Cdc20-associated when they are being replicated, high DNA polymerase occupancy correlates with replication fork slowing in both *S*. *pombe* and *S*. *cerevisiae* [39, 49]. Most of the high Cdc20 occupancy sites in WT cells were also found in Pfh1-depleted cells (390/485, 80%) and vice versa (389/517, 75%) (Fig 2A).

In an earlier study, we used these data to demonstrate that many G4 motifs bind Pfh1 and that fork slowing and DNA breakage is more frequent at G4 motifs than at other G-rich regions in Pfh1-depleted cells (S2 Fig) [39]. Here we extend this analysis from G4 motifs to the rest of the genome. This analysis showed that in addition to G4 motifs, tRNA genes, 5S rRNA genes, NDRs, replication origins, RNA polymerase II promoters, RNA polymerase II transcribed genes, and meiotic DSB hotspots were significantly enriched among high Cdc20 occupancy sites in both WT and Pfh1-depleted cells (Table 2 and S2 Table; p < 0.001). The sets of high Cdc20 occupancy were identical in the two conditions except that dubious genes were enriched in Pfh1-depleted but not in WT cells. Despite this similarity, the evidence for elevated Cdc20 occupancy was significantly greater in Pfh1-depleted cells (p ≈ 0, Wilcoxon signed-rank test, p-values < 10^−50^ are reported as ≈0; Fig 2B). This pattern held for all genomic features tested (Fig 2C–2F; S3 Table). For example, 69% of Cdc20 peaks near NDRs (p = 2.4x10^-5^; Fig 2C) and 62% of peaks near highly transcribed RNA polymerase II genes (p ≈ 0; Fig 2D) were significantly stronger in Pfh1-depleted cells. This effect was particularly striking at tRNA (p ≈ 0; Fig 2E) and 5S rRNA (p = 1.4x10^-6^; Fig 2F) genes, where over 97% of the peaks showed evidence of significantly elevated Cdc20 occupancy when cells were Pfh1-depleted compared to WT cells. These findings argue that these genomic features, all of which had significant Pfh1 occupancy in WT cells (Table 1), were particularly dependent on Pfh1 for timely replication.

**Table 2:** Association of genomic features with Cdc20 and γ-H2A in WT and Pfh1-depleted cells. Only features significantly associated with Pfh1 binding after correction for GC content and multiple testing (Bonferroni, p < 0.003) are shown. Each row gives the fraction of each feature associated with Cdc20 or γ-H2A; p-values for the association are in parentheses. Associations significant after Bonferroni multiple testing correction are bold. See S3 Table and S6 Table for full association results.

	WT		Pfh1-depleted	
Genome Features	Cdc20	γ-H2A	Cdc20	γ-H2A
5S rRNA genes	**0.73 (≈ 0)**	0.24 (0.08)	**0.97 (≈ 0)**	**0.85 (≈ 0)**
tRNA genes	**0.68 (≈ 0)**	0.28 (0.08)	**0.80 (≈ 0)**	**0.75 (≈ 0)**
Meiotic Double Strand Break Hotspots	**0.34 (≈ 0)**	0.22 (0.11)	**0.41 (≈ 0)**	**0.47 (≈ 0)**
500 Highest Expressed RNA Polymerase II Genes	**0.22 (≈ 0)**	0.14 (0.83)	**0.22 (≈ 0)**	0.36 (0.85)
Nucleosome Depleted Regions	**0.16 (≈ 0)**	0.15 (0.17)	**0.19 (≈ 0)**	**0.38 (≈ 0)**
G4 motifs*	0.11 (0.03)	0.17 (0.01)	**0.17 (≈ 0)**	**0.40 (≈ 0)**
3’ UTRs	0.08 (0.05)	0.13 (0.53)	0.09 (0.21)	0.33 (0.004)

*from Sabouri, Capra [39]

To compare the Pfh1-dependent effects in more detail, we analyzed the genomic features associated with the 95 peaks of high Cdc20 binding unique to WT cells versus those associated with the 128 peaks of high Cdc20 binding that were found only in Pfh1-depleted cells. No features were enriched among the unique WT peaks. In sharp contrast, 5S rRNA and tRNA genes, meiotic DSB hotspots, NDRs, and dubious genes were all significantly enriched amongst the Cdc20 peaks unique to Pfh1-depleted cells (S2 Table).

Taken together, our results show that several classes of genomic features, especially RNA polymerase III transcribed genes, depend on Pfh1 for normal fork progression. In cases where sites were hard to replicate even in WT cells, fork pausing at these sites was significantly more pronounced in Pfh1-depleted cells.

### Pfh1 protects the genome from DNA damage

In virtually all eukaryotes, including *S*. *pombe*, phosphorylation of H2A (γ-H2A) marks sites of DNA damage, typically DSBs [50]. To determine if the site-specific increases in replication pausing detected in Pfh1-depleted cells were associated with DNA damage, we analyzed peaks from previous ChIP-seq experiments using anti-γ-H2A antibodies in WT or Pfh1-depleted cells (S1 Table) [39]. We quantified the genomic distribution and Pfh1-dependence of the γ-H2A peaks with the same methods used for Cdc20.

**Fig 3:**
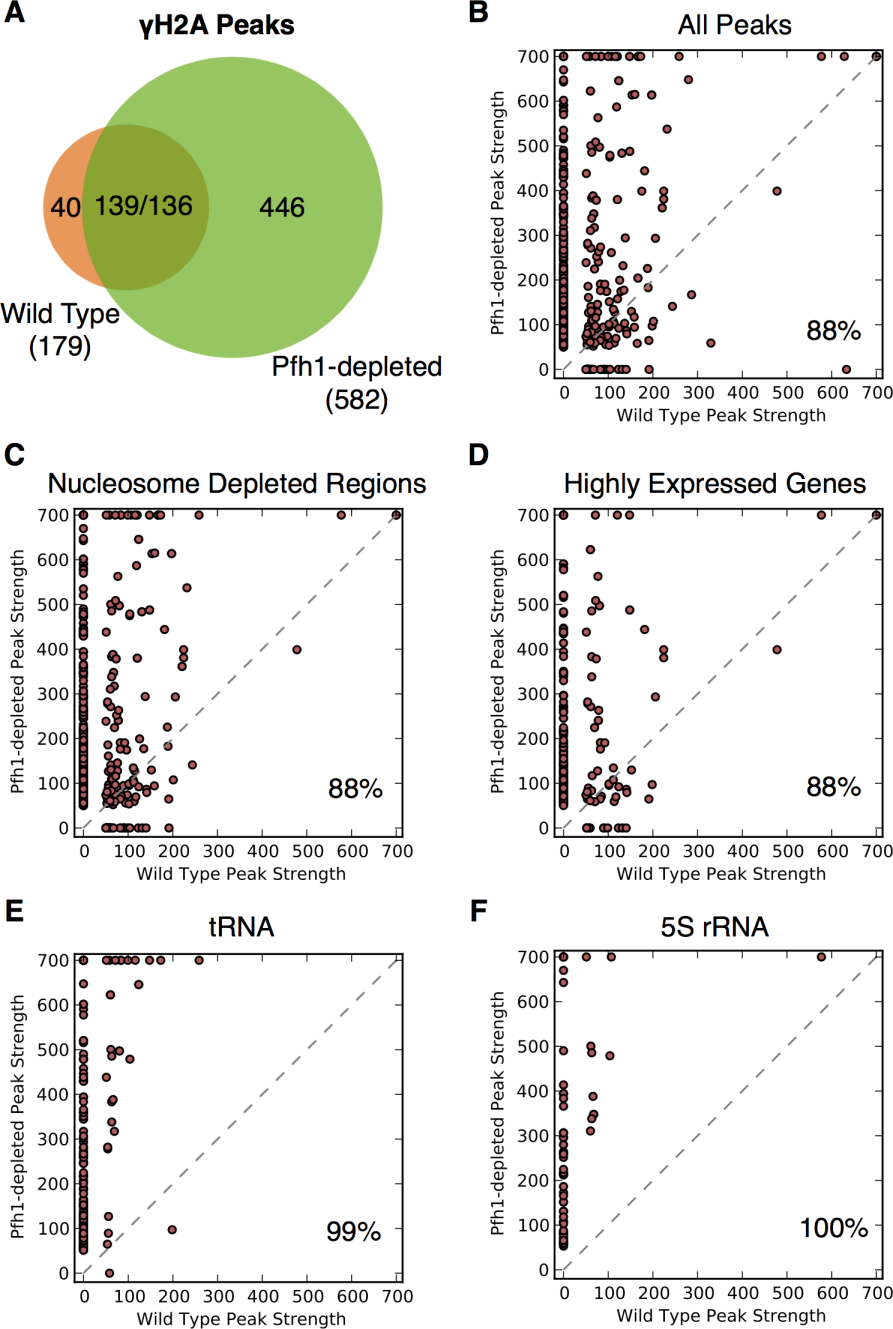
DNA damage as marked by phosphorylated histone H2A (γ-H2A) levels is increased in the absence of Pfh1. (A) Venn diagram showing the overlap of genome-wide γ-H2A occupancy peaks in the presence and absence of Pfh1. The two numbers in the intersection are the number of WT peaks that overlap a Pfh1-depleted peak and vice versa. (B) Scatter plot comparing γ-H2A peak strength in WT and Pfh1-depleted cells. The layout is the same as in Fig 2B. Overall, γ-H2A peaks were significantly stronger in Pfh1-depleted cells (88%, p ≈ 0, Wilcoxon signed-rank test). Peaks near most tested genomic features were significantly stronger in Pfh1-depleted cells (S3 Table), e.g., (C) NDRs (88%, p ≈ 0) and (D) highly expressed genes (88%, p ≈ 0). γ-H2A peaks associated with (E) tRNA (99%, p ≈ 0) and (F) 5S rRNA genes (100%, p ≈ 0) were almost universally stronger in Pfh1-depleted cells.

As demonstrated in previous work, WT cells had 179 γ-H2A peaks and Pfh1-depleted cells had 582 peaks (Fig 3A) [39]. Only two genomic features, the mating type loci and origins of replication, were significantly enriched near high occupancy γ-H2A sites in WT cells (p < 0.001 for both; S5 Table). However, in Pfh1-depleted cells, 5S rRNA and tRNA genes, meiotic DSB hotspots, NDRs, the mating type loci, and origins of replication all overlapped significantly with γ-H2A peaks (p < 0.001; Table 2 and S5 Table).

We also determined the degree of overlap between Cdc20 and γ-H2A peaks in Pfh1-depleted cells. Of the 582 γ-H2A peaks in Pfh1-depleted cells, 71% (411) also had high Cdc20 occupancy; this number is significantly more than expected by chance (p < 0.001). γ-H2A sites that were high occupancy for both γ-H2A and Cdc20 were enriched for multiple genomic features including 5S rRNA and tRNA genes, origins of replication, meiotic DSB hotspots, 3’ and 5’ UTRs, and promoters (p < 0.001 for all; S6 Table). These features include most of those with significant Pfh1 association in WT cells. In contrast, γ-H2A peaks without corresponding Cdc20 peaks were enriched only in 3’ and 5’ UTRs and promoters (p < 0.001 for both). The significant overlap between peaks of Cdc20 and γ-H2A binding supports the connection between Pfh1-dependent fork slowing (as marked by Cdc20 peaks) and DNA damage (as marked by nearby γ-H2A) at several classes of hard-to-replicate sites.

**Fig 4:**
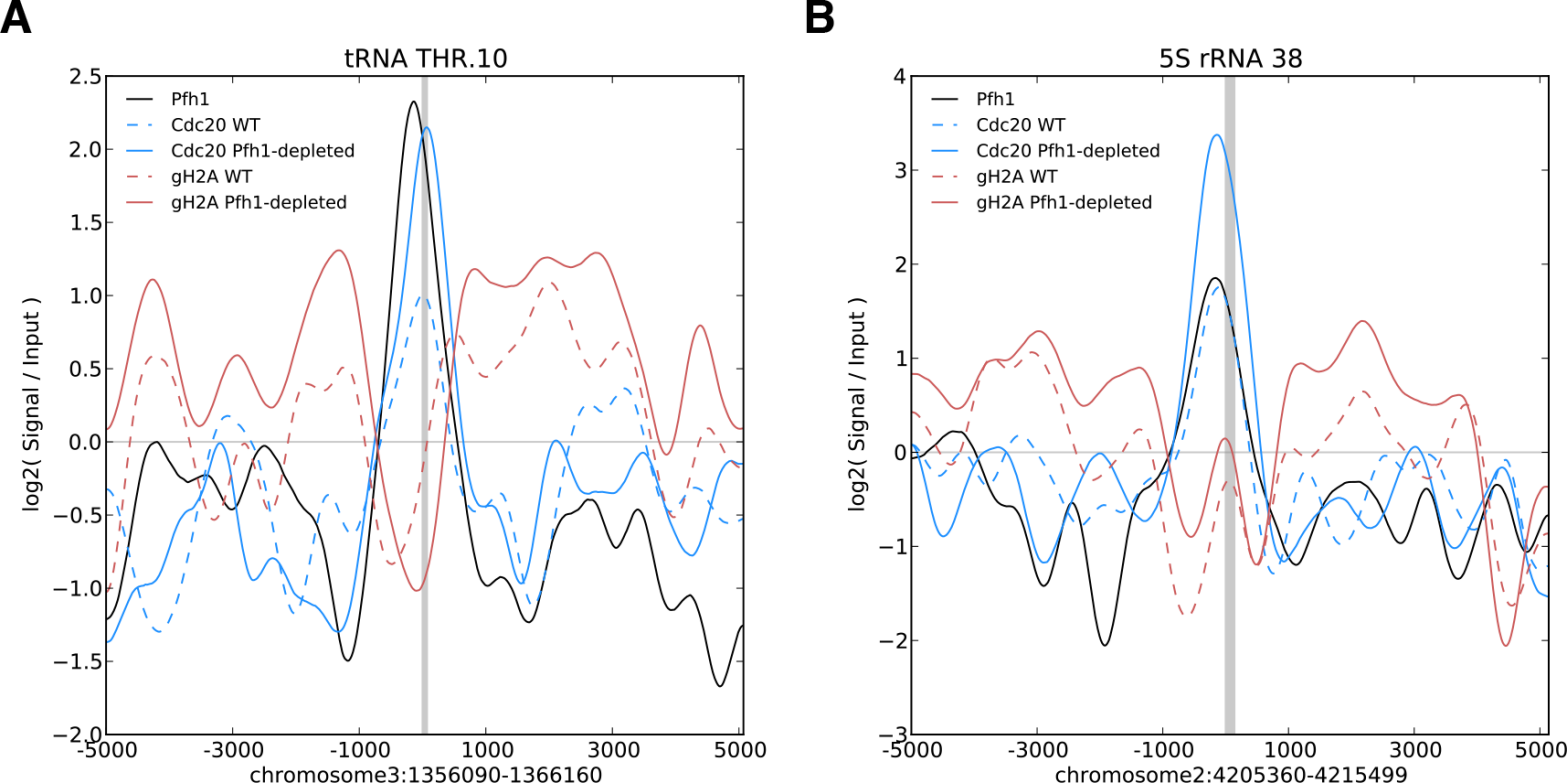
**ChIP-seq signal surrounding two representative RNA-PolIII transcribed genes: a threonine tRNA gene (A) and a 5S rRNA gene (B).** Each plot displays the smoothed base 2 logarithm of the ChIP-seq reads mapping to each position in the experimental context over the corresponding input only read count. The raw signal was smoothed by convolution with a 1 kb Hanning window. The coordinates on the x-axis give the genomic location of the gene (gray box). These examples highlight a common pattern for tRNA and 5S rRNA genes: a peak of Pfh1 occupancy centered on the gene (black), with overlapping Cdc20 binding that is increased in the absence of Pfh1 (blue), and elevated γ-H2A in the regions flanking the genes in Pfh1-depleted cells (red). Plots for all tRNA and 5S rRNA genes are given in S3 and S4 Figs.

These patterns are illustrated for two specific sites, a tRNA gene (Fig 4A) and a 5S rRNA gene (Fig 4B). The strengths of the Pfh1, Cdc20, and γ-H2A binding are shown relative to input for the different strains in a 10 kilobase (kb) window around each gene. At both sites, a Pfh1 peak overlapped the gene (grey vertical lines mark centers of genes). A Cdc20 peak showed a similar overlap with the gene in both WT (dashed blue line) and Pfh1-depleted cells (solid blue line), but the peak was stronger in Pfh1-depleted cells. Broad peaks of γ-H2A flanked both genes in Pfh1-depleted cells, consistent with the 40 kb domains of γ-H2A that flank DSB sites [50]. Plots for all tRNA and 5S rRNA genes are shown in S3 and S4 Figs.

When the strengths of all the γ-H2A peaks between WT and Pfh1-depleted cells were compared, 88% of all peaks were higher in Pfh1-depleted cells (p ≈ 0, Wilcoxon signed-rank test; Fig 3B). This pattern held for the subsets of γ-H2A peaks associated with nearly all classes of genomic features (Fig 3C–3F; S3 Table). However, as seen for Cdc20, the magnitude of the difference was strongest for the RNA polymerase III transcribed genes: 190 of 192 γ-H2A peaks near tRNA genes (p ≈ 0, Fig 3E) and all 67 γ-H2A 5S rRNA peaks (p ≈ 0, Fig 3F) were greater in Pfh1-depleted cells.

### During S phase, Pfh1 associates with Pfh1-sensitive and Pfh1-insensitive sites throughout the genome

Our data indicate that Pfh1 promotes replication and suppresses DNA damage at many discrete sites in the genome. We considered two models to explain this pattern of Pfh1 action. First, Pfh1 could act by binding nearby the replisome and mitigating replication obstacles as the replisome moves past Pfh1-sensitive sites. Alternatively, Pfh1 could be recruited only to sites that are hard to replicate or to stalled replication forks. In that case, Pfh1 would have low or no binding to sites that are Pfh1-insensitive. To distinguish between the two possibilities, we used ChIP-qPCR to monitor association of Pfh1 and Cdc20 in synchronized cells. For these experiments, we used a *cdc25-22* strain that expressed Pfh1-13Myc inserted at the *leu1*^*+*^ locus under the control of the *pfh1*^*+*^ promoter (the endogenous *pfh1*^*+*^ locus was unaltered). This strain also expressed Cdc20-3HA from its endogenous location.

**Fig 5:**
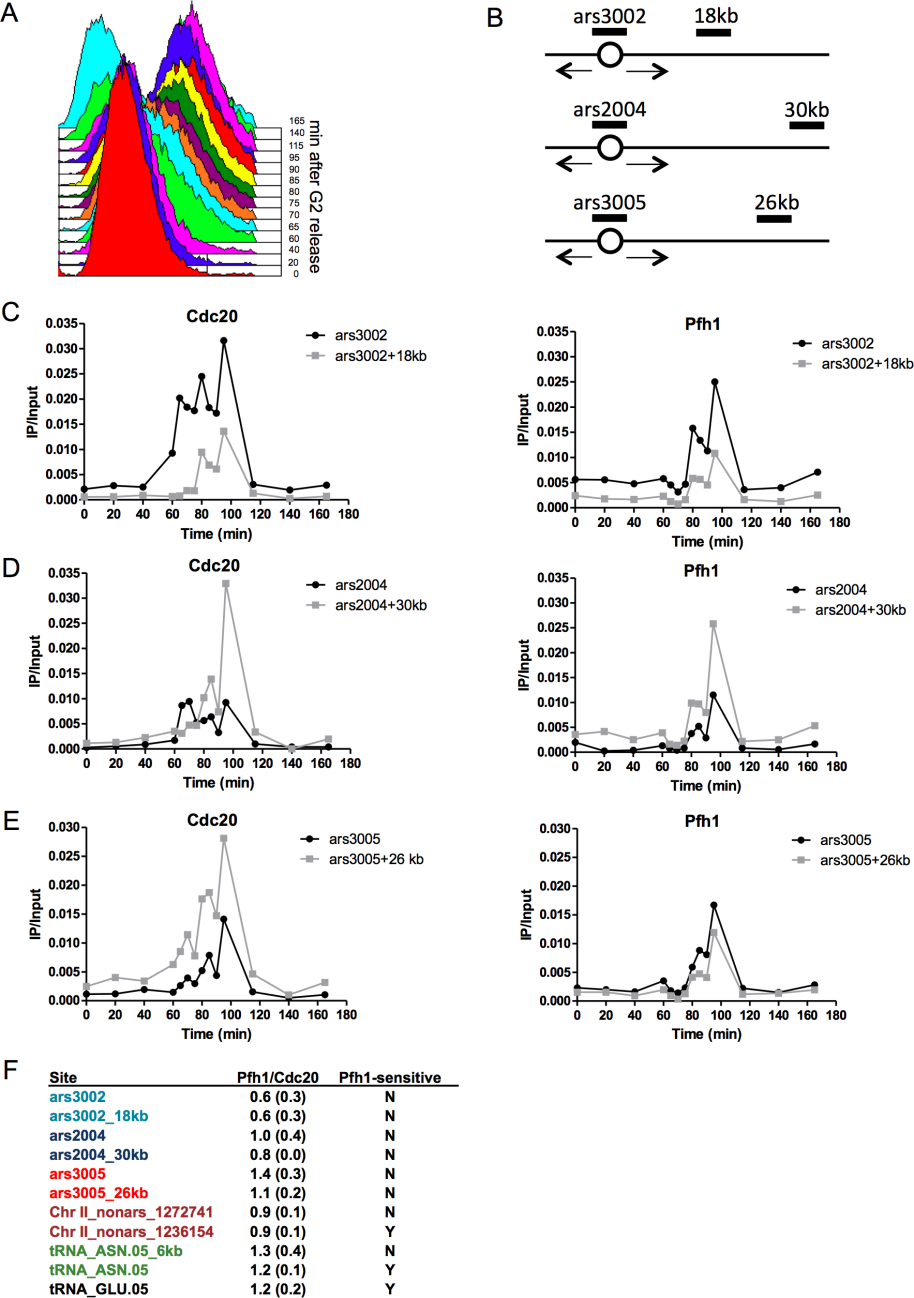
Pfh1 and DNA Pol ε both bind Pfh1-sensitive and insensitive sites. (A) FACS analysis of cell synchrony. (B) Schematic of the regions examined by qPCR. Open circles represent origins of replication. Boxes mark the position of primer-pairs for qPCR that detect ars3002, ars3002_18kb, ars2004, ars2004_30kb, ars3005, and ars3005_26kb for ChIP-qPCR experiments. (C-E) Samples from each time point of the synchronized Cdc20-3HA Pfh1-13Myc *cdc25-22* strain were immunoprecipitated with either anti-HA (left) or anti-Myc (right) antibody. The DNA was analyzed with qPCR using primer pairs for (C) ars3002 and ars3002_18kb, (D) ars2004 and ars2004_30kb, and (E) ars3005 and ars3005_26kb. Although cells appeared synchronous by FACS analysis, and Pfh1 and Cdc20 showed similar temporal patterns with peak binding at 95 min, we did not detect progression of the replisome from the ars2004 and ars3005 origins to their adjacent regions (ars2004_30kb and ars3005_26kb). This is likely due to the documented heterogeneity of origin usage in different cells in the same population [66] and to inefficient origins. The experiments were performed at least two times and the graphs show one representative biological replicate. (F) The ratio of Pfh1 IP/input to Cdc20 IP/input at peak binding after G2 release for eleven regions. Samples from Cdc20-3HA Pfh1-13Myc *cdc25-22* synchronized cells were immunoprecipitated with either anti-HA or anti-Myc antibody and analyzed by qPCR. The experiments were repeated two times and the reported ratio is the average of two biological replicates; the standard deviation is given in parentheses. The names of adjacent regions are in the same color. Y: yes, N: no.

Cells were arrested in G2 phase by incubation at the non-permissive temperature (37°C) and then released at permissive temperature (25°C). The position in the cell cycle and the quality of the synchrony were determined by FACS analysis (Fig 5A). To determine association and movement of the replication fork, samples were taken throughout one synchronous cell cycle. At each time point, we examined association of Pfh1-13Myc and Cdc20-3HA to three origins of replication and their adjacent regions. We examined binding to the efficient ars3002 and a region18 kb away from ars3002 (ars3002_18kb), ars2004 and a region 30 kb from ars2004 (ars2004_30kb), and ars3005 and a region 26 kb from ars3005 (ars3005_26kb) (Fig 5B).

If Pfh1 were nearby or associated with the replisome, Pfh1 and Cdc20 would have similar temporal patterns of binding to the three origins and their adjacent sites. If Pfh1 were recruited only to hard-to-replicate sites, then Cdc20 and Pfh1 would have different binding patterns. In fact, under the second model, Pfh1 should not bind to any of these sites, as none of them were Pfh1-dependent in the whole genome analysis. Consistent with the first model, Cdc20 and Pfh1 displayed similar association patterns at all three origins and their adjacent regions (Fig 5C–5E). We first examined the binding to ars3002 and its adjacent region ars3002_18kb (Fig 5C). Although Cdc20 bound in early S phase to ars3002, earlier than Pfh1, the peak binding for Cdc20 and Pfh1 was reached at 95 min (Fig 5C). Both proteins had their start of binding to ars3002_18kb at 80 min after release from G2 phase and their peak binding at 95 min (Fig 5C). Thus, Pfh1 and Cdc20 bound to the Pfh1-insensitive site located 18 kb downstream of ars3002 similarly. However, while clear movement of Cdc20 was detected in this region, movement from this origin to the downstream regions was not visible for Pfh1.

Next, we examined the binding of Pfh1 and Cdc20 to the four other Pfh1-insensitive sites ars2004, ars2004_30kb, ars3005, and ars3005_26kb. Pfh1 bound to all these four regions, similarly to Cdc20 (Fig 5D and 5E). However, movement of neither Cdc20 nor Pfh1 was detected at any of these origins to their adjacent sites.

Because the dynamics of Pfh1 and the replisome were not clear from the above experiments, we further investigated the binding pattern of Pfh1 and Cdc20 at five other regions, including both Pfh1-sensitive and Pfh1-insensitve sites (Fig 5F). We tested two regions that were not origins of replication on chromosome II: one Pfh1-sensitive (Chr II_nonars_1236154), and a Pfh1-insensitive site 36 kb away (Chr II_nonars_1272741). Finally, we examined two Pfh1-sensitive tRNAs (tRNA^GLU.05^ and tRNA^ASN.05^) and one Pfh1-insensitive site 6 kb away from tRNA^ASN.05^ (tRNA^ASN.05^_6kb).

At all five regions, both Pfh1 and Cdc20 had peak binding at 95 min after release from the G2 arrest, which by FACS analysis is mid-S phase (Fig 5). To determine if Pfh1 was recruited only to Pfh1-sensitive sites, we calculated the ratio of Pfh1 to Cdc20 binding at Pfh1-sensitive and -insensitive sites (IP/input of Pfh1 divided by IP/input for Cdc20) at the peak of replication for all sites (Fig 5F). If Pfh1 were recruited solely to Pfh1-sensitive sites, the ratio of Pfh1/Cdc20 would be higher at Pfh1-sensitive sites compared to Pfh1-insensitive sites. If Pfh1 were in proximity to the replisome, the ratios should be similar at the two classes of sites. Indeed, the Pfh1/Cdc20 ratio was on average 1.1 and 0.9 for Pfh1-sensitive and Pfh1-insensitive sites, respectively (Fig 5F). Thus, Pfh1 binds similarly to both Pfh1-sensitive and -insensitive sites. These data suggest that Pfh1 is near the replisome during S phase, rather than being recruited to its sites of action at Pfh1-sensitive sites. However, we cannot rule out the possibility that additional Pfh1 molecules are recruited to some or even all Pfh1-sensitive sites upon replisome pausing.

### Mass spectrometry identifies Pfh1 interacting proteins

**Fig 6:**
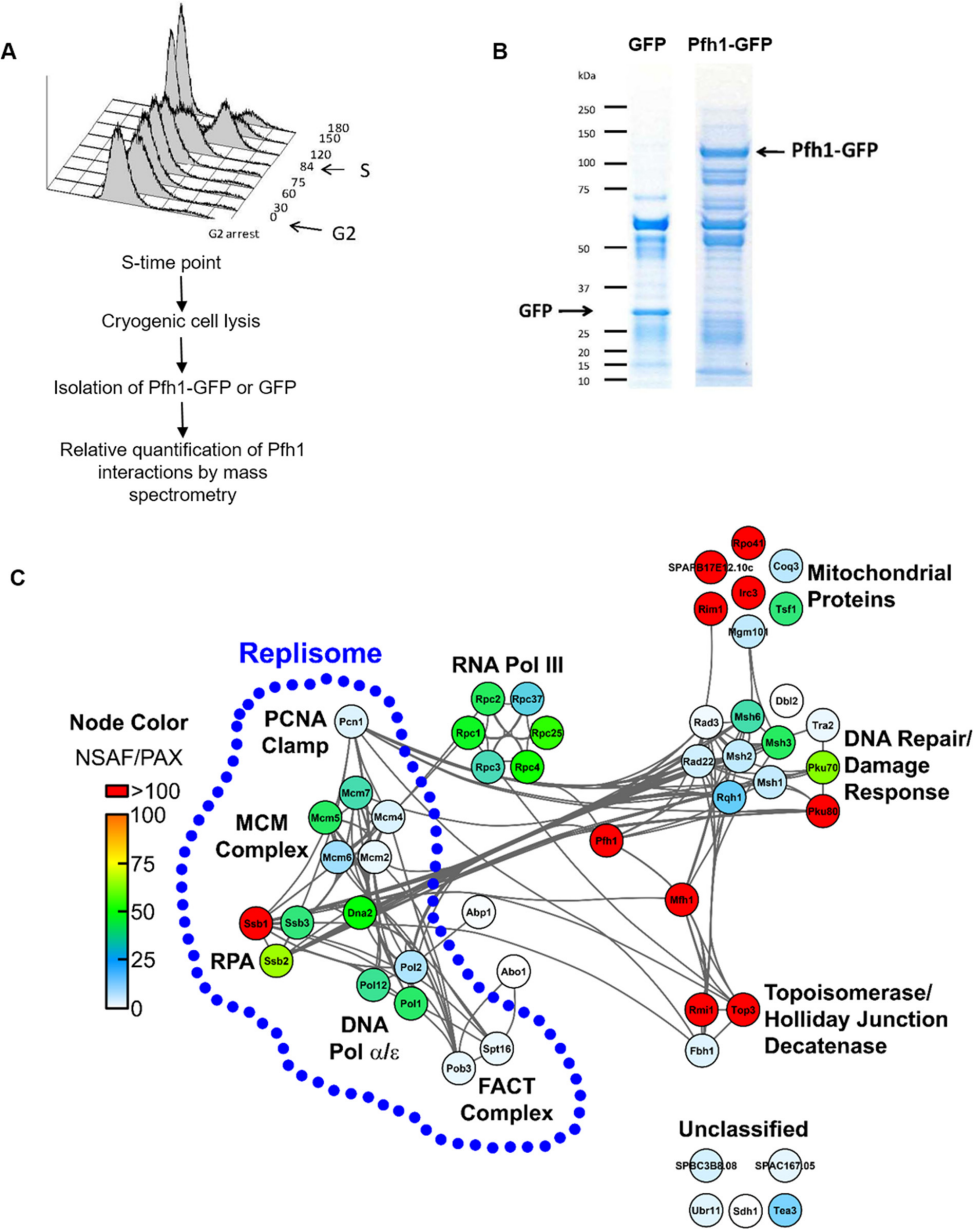
Identification of Pfh1 protein interactions during S phase. (A) Experimental design for parallel immunoaffinity purifications of Pfh1-GFP and NLS-GFP from cells harvested at S phase of the cell cycle and relative quantification of Pfh1 specific interactions by MS. As determined by FACS analysis, cdc25-22 cells were collected at the start of S phase, 84 minutes after G2 phase arrest. (B) Immunoaffinity purifications of GFP or Pfh1-GFP from S phase resolved by SDS-PAGE gel with the target and associated proteins visualized by Coomassie Blue staining. (C) Interaction network of high confidence Pfh1 protein associations during S phase, as identified by MS and assessed for specificity of binding by SAINT (p ≥ 0.80, n = 2). Nodes represent individual proteins interacting with Pfh1; node color represents relative enrichment compared to their abundance in the proteome (NSAF/PAX); and edges represent known interactions curated by the STRING database. Replisome components are highlighted by the blue dotted line.

If Pfh1 maintains proximity to the replisome, as suggested by its pattern of binding to chromosomal DNA (Fig 5), then Pfh1 should be associated *in vivo* with known replisome subunits. To address this possibility, we used immunoaffinity purification mass spectrometry (IP-MS) to identify the Pfh1 protein interactions in S phase cells (Fig 6A). In these experiments, Pfh1 was expressed under its endogenous promoter as a GFP fusion (Pfh1-GFP), allowing immunoaffinity isolation of Pfh1 and its associated proteins through the GFP tag [51]. Cells expressing a SV40 nuclear localization signal-GFP fusion (NLS-GFP) were used as a negative control for non-specific association of proteins to GFP. Two biological replicates of both Pfh1-GFP and NLS-GFP were isolated in parallel from S phase cells (Fig 6A) using anti-GFP antibodies (Fig 6B).

Following mass spectrometry analysis of Pfh1-GFP and NLS-GFP immunoisolates, the interaction specificity of individual co-isolating proteins was assessed using the SAINT (significance analysis of interactome) algorithm [52]. SAINT determines confidence scores (ranging from 0 to 1) for protein-protein interactions based on the spectrum count distributions obtained from bait (Pfh1-GFP) isolations relative to the negative control (NLS-GFP). High confidence Pfh1 interactions were defined as proteins having a SAINT score ≥ 0.80, a threshold used previously to identify functional protein interactions [53, 54]. By this metric, there were 50 high confidence Pfh1 protein associations that comprise the Pfh1 S phase interactome (Table 3 and S7 Table). Although five of the Pfh1 interacting proteins are uncharacterized, there is functional data for 45 of the 50 proteins. Table 3 lists these proteins.

**Table 3:** Pfh1-GFP interacting proteins. Pfh1-associated proteins during S phase (SAINT score ≥ 0.80) presented in alphabetical order. Names in bold are replisome components or replication related; italics indicate repair/recombination proteins; underlining indicates mitochondrial proteins. Functional annotations are from PomBase [68].

Protein	Accession number	Function	SAINT score
**Abp1**	P49777	ARS-binding protein, CENP-B homologue, less abundant in G2	0.83
Coq3	O74421	Mitochondrial hexaprenyldihydroxybenzoate methyltransferase	0.86
**Dna2**	Q9URU2	ATP-dependent helicase-nuclease, processes Okazaki fragments	0.95
*Fbh1*	Q9USU3	F-box DNA helicase, modulates homologous recombination	0.97
Irc3	Q1MTR1	Putative mitochondrial ATP-dependent helicase	1.00
**Mcm2**	P40377	Subunit of MCM replicative DNA helicase	0.89
**Mcm4**	P29458	Subunit of MCM replicative DNA helicase	0.92
**Mcm5**	P41389	Subunit of MCM replicative DNA helicase	0.90
**Mcm6**	P49731	Subunit of MCM replicative DNA helicase	0.83
**Mcm7**	O75001	Subunit of MCM replicative DNA helicase	0.94
*Mfh1*	Q9UT23	FANCM-like DNA helicase, repairs inter-strand crosslinks, promotes meiotic recombination	0.99
Mgm101	O14354	Mitochondrial genome maintenance protein	0.87
***Msh1***	O13921	DNA mismatch repair protein	0.98
***Msh2***	O74773	DNA mismatch repair protein	0.99
***Msh3***	P26359	DNA mismatch repair protein	0.99
***Msh6***	O74502	DNA mismatch repair protein	0.99
**Pcn1**	Q03392	Subunit of homotrimeric PCNA (Proliferating cell nuclear antigen)	0.90
*Pku70*	O94395	Subunit of Ku heterodimer	1.00
*Pku80*	Q9HGM8	Subunit of Ku heterodimer	1.00
**Pob3**	O94529	FACT complex subunit, remodels histones	0.86
**Pol1**	P28040	Catalytic subunit of DNA polymerase alpha	0.82
**Pol12**	O74946	Subunit B of DNA polymerase alpha	0.84
**Pol2**	P87154	Catalytic subunit of DNA polymerase epsilon; also called Cdc20	0.99
*Rad22*	P36592	DNA repair and recombination protein, homologue budding yeast Rad52	0.83
*Rad3*	Q02099	ATR-like checkpoint protein kinase	0.98
Rim1	O14087	Mitochondrial single-stranded DNA-binding protein	0.99
*Rmi1*	Q10160	Subunit of Rqh1/Top3 complex, suppresses DNA damage	0.89
Rpc1	O94666	Subunit of RNA polymerase III	0.98
Rpc2	Q10233	Subunit of RNA polymerase III	0.83
Rpc25	O94285	Subunit of RNA polymerase III	0.82
Rpc3	Q9C106	Subunit of RNA polymerase III	0.86
Rpc37	O74883	Subunit of RNA polymerase III	0.83
Rpc4	O74857	Subunit of RNA polymerase III	0.89
Rpo41	O13993	Mitochondrial RNA polymerase, priming mtDNA replication	0.89
*Rqh1*	Q09811	RecQ family DNA helicase subunit of Rqh1/Top3 complex, suppresses DNA damage	1.00
Sdh1	Q9UTJ7	Mitochondrial protein, probable succinate dehydrogenase flavoprotein subunit	0.83
SPAC167.05	P87132	Largely uncharacterized but implicated in meiotic chromosome segregation	0.84
SPAC1F5.11c	Q8TFH4	Tra2 subunit of NuA4 complex phosphatidylinositol pseudokinase	0.99
SPAC31G5.19	O14114	Uncharacterized AAA domain-containing protein	1.00
SPAPB17E12.10c	Q8TFH4	Mitochondrial protein, affects RNA processing	0.99
SPBC3B8.08	O59716	Uncharacterized protein	0.90
SPCC553.01c	O74939	Meiotic chromosome segregation protein Dbl2	0.99
**Spt16**	O94267	FACT complex subunit, remodels histones	0.86
**Ssb1**	Q92372	Replication factor A protein 1, RPA subunit	1.00
**Ssb2**	Q92373	Replication factor A protein 2, RPA subunit	0.99
**Ssb3**	Q92374	Replication factor A protein 3, RPA subunit	0.87
Tea3	O14248	Tip elongation aberrant protein 3, cell polarity	0.94
*Top3*	O60126	DNA topoisomerase 3, interacts with Rqh1 helicase, suppresses DNA damage	0.98
Tsf1	Q9HGL5	Mitochondrial translation elongation factor	0.85
Ubr11	O13731	E3 ubiquitin-protein ligase ubr11, affects chromosome stability, perhaps affects kinetochore	0.97

We also assessed the relative abundance of individual Pfh1 interacting proteins within the interaction network by calculating the normalized spectrum abundance factor (NSAF) for each protein relative to its proteome abundance value (PAX) [55]. NSAF values provide a measure of protein abundance by accounting for factors such as protein length and sample complexity that can influence the number of spectra acquired for a given protein within a sample. Normalizing NSAF values to PAX values, as described in [56], provides insight into proteins and functional protein classes that are enriched in the Pfh1 isolation relative to their abundances in the cellular proteome. These data are presented in Fig 6C, which also categorizes interacting proteins by function.

### Pfh1 interacts with multiple replisome components

The replisome is the multi-protein complex that is present at the replication fork as it moves through the chromosome. Multiple replisome components interacted with Pfh1 with high specificity (SAINT score ≥ 0.80; Fig 6C and Table 3). These proteins were: (1) five of the six subunits of the replicative helicase, the MCM complex (Mcm2 and Mcm4-7); (2) catalytic subunits of two of the three replicative polymerases (DNA Pol1 from DNA polymerase α; DNA Pol2/Cdc20, from DNA polymerase ε); (3) Pol12, the β subunit of DNA Pol1; (4) proliferating cell nuclear antigen (PCNA, Pcn1), a processivity factor that encircles and slides along the DNA; (5) the three subunits of the single-strand binding replication factor A (RPA, Ssb1, 2 and 3); (6) the Dna2 helicase-nuclease that is required for Okazaki fragment maturation; and (7) the two subunits of the FACT complex (facilitates chromatin transcription), Pob3 and Spt1, which facilitates nucleosome remodeling during both transcription and DNA replication. The association of FACT subunits with Pfh1 suggests that FACT and Pfh1 might act synergistically to promote replication through hard-to-replicate sites. Four mismatch repair (MMR) proteins, Msh1, 2, 3 and 6, were also Pfh1-associated. The Msh2/6 and Msh2/3 heterodimers interact directly with DNA for the recognition of base pair mismatches. Because MMR and DNA replication are strongly coupled in budding yeast, MMR proteins are proposed to track with the replisome and hence can also be considered replisome components [57, 58].

Additional replisome components were present in the Pfh1-GFP isolations but did not meet our SAINT score criterion. These proteins were: (1) the sixth Mcm subunit (Mcm3; SAINT score, 0.68); (2) the catalytic subunit of the lagging strand DNA polymerase; Pol3 (SAINT score, 0.15); (3) Dpb2, the second largest subunit of Pol ε (SAINT score 0.31); (4) Pri1 and Pri2, the primase subunits that function together with DNA polymerase α to synthesize the primers on the leading and lagging strand (SAINT scores of 0.65 and 0.33, respectively); and (5) Mcl1, the *S*. *cerevisiae* Ctf4 homologue that interacts with DNA polymerase α (SAINT score 0.70). While SAINT scores point to high confidence interactions, being based on detected protein spectral counts, they are influenced by sample complexity and the dynamic range of the co-isolated proteins, and thereby weighted towards large and abundant proteins, and stable interactions. Pfh1-associated replisome components with lower SAINT scores may be smaller proteins, have lower cellular abundances, and/or form transient interactions [59].

We performed two additional experiments to confirm the association of Pfh1 with the replisome. First, we isolated Pfh1 from asynchronous cells both in the presence and absence of DNase (S7 Table and S5 and S6 Figs). Because this experiment was performed with an asynchronous population, only a subset of the proteins that interacted with Pfh1 in S phase was detected (S6 Fig), even without DNase treatment. Of the 19 replisome/replication proteins that passed our stringent SAINT score threshold, ten were found in the DNase untreated sample, and nine of these retained their Pfh1 association after DNase treatment: Ssb1 and 2, Msh2, Mcm4, 5, and 6, Cdc20 (Pol2), Pol12, and Spt16. Because these interactions were not DNA-dependent, they are likely due to protein-protein interactions. Second, we isolated Pfh1 and its associated proteins from G2 arrested cells. We detected eight Pfh1-associated replication/replisome proteins in G2, and all eight were detected with fewer spectral counts in G2 extracts than in S phase extracts. The remaining eleven were not detected at all as Pfh1-interacting proteins in G2 phase (Table 3; S7 Table). Thus, as expected for a replisome component, Pfh1 association with known replisome subunits was either lost or diminished in G2 phase.

Together, these results show that Pfh1 associated *in vivo* with numerous replisome proteins, and that replisome and replication-related proteins represent a substantial subset of specific Pfh1 interactions (19 out of 50 proteins (38%) with SAINT score of ≥0.8) (Table 3; in bold; Fig 6C). Almost all of these associations were S phase-limited or S phase-enriched as well as DNA independent.

### Pfh1 interacts with multiple mitochondrial and repair proteins

Pfh1 is a multi-functional protein: in addition to its role in nuclear DNA replication, it promotes DNA repair and is essential for maintenance of mitochondrial (mt) DNA [34]. Consistent with Pfh1 having mt function, 8 of the 50 high confidence Pfh1 interaction proteins have mt annotations (Fig 6C; mt proteins are underlined in Table 3). This subset includes several proteins implicated in mtDNA replication, such as (1) Rim1, the mt single-strand DNA binding protein (MS analyses reveal that ScPif1 is also ScRim1-associated; [60]), (2) Rpo1, the mtRNA polymerase that is thought to prime mtDNA replication, and (3) Mgm101, which is required for maintenance of mtDNA by an unknown mechanism.

Consistent with the reported DNA repair functions of Pfh1 [34], we observed multiple repair proteins among the high confidence Pfh1 interactions (Table 3, *italics*), including Rad22, the *S*. *pombe* homolog of budding yeast Rad52, which is required for homologous recombination [61], Rad3, the ATR-like checkpoint kinase [62], and both subunits of the non-homologous end joining Ku complex, pKu70 and 80. In addition, Rqh1 (homolog of human BLM) DNA helicase and its two interacting partners, the topoisomerase Top3 and Rmi1, were Pfh1-associated. This highly conserved heterotrimeric complex has multiple functions, but is best known for suppressing DNA damage at hard-to-replicate sites, such as converged forks [63] and/or collapsed replication forks—functions relevant to those of Pfh1.

Finally, six subunits of the 26 subunit RNA polymerase III complex were Pfh1-associated with high confidence (Rpc1, 2, 3, 4, 25 and 37), while four others were Pfh1-associated but had SAINT scores <0.8 (Rpc6, 0.71; Rpc9 and 10, 0.30; Rpc19, 0.76) [64]. This finding is probably related to RNA polymerase III transcribed genes being among the most potent replication impediments in Pfh1-depleted cells (Figs 2–4; see Discussion).

## Discussion

### Pfh1 acts at diverse sites genome-wide

We used genome-wide assays to determine sites where replication and genome integrity are Pfh1-dependent. The most striking aspect of these data is the strong dependence of RNA polymerase III transcribed genes on Pfh1. 2D gel analyses showed previously that replication of five of five tested tRNA genes is Pfh1-dependent, and this dependence is seen regardless of whether replication is co-directional or opposite to the direction of transcription through the gene [35]. Here we show that close to 50% (80/171) of the tRNA genes bound Pfh1 (Table 1). Moreover, nearly all of the tRNA genes that bound Pfh1 were sites of fork pausing and DNA damage in both WT and Pfh1-depleted cells (Table 2; high Cdc20 binding: WT: 74/80, Pfh1-depleted: 80/80: high γ-H2A: WT: 25/80, Pfh1-depleted: 69/80). However, both Cdc20 (Fig 2E) and γ-H2A binding (Fig 3E) were significantly higher at virtually all (99%) tRNA genes in Pfh1-depleted compared to WT cells. Likewise, genome-wide analyses revealed that 55% (18/32) of 5S rRNA genes bound Pfh1 (Table 1), and again the majority of these genes were sites of fork pausing (Table 2; WT: 16/18, Pfh1-depleted: 18/18) and/or DNA damage (WT: 5/18, Pfh1-depleted: 14/18), and both features were significantly higher in Pfh1-depleted compared to WT cells (Figs 2F and 3F). The independent MS analysis also supports the importance of Pfh1 at RNA polymerase III transcribed genes, as Pfh1 interacted with multiple subunits of RNA polymerase III (Fig 6C; Table 3). The association of these subunits with Pfh1 is consistent with a model where Pfh1 promotes replication past genes by displacing these proteins from DNA.

In addition to RNA polymerase III genes, 60% of the 500 most highly expressed protein-coding genes over a range of growth conditions were bound by Pfh1 (Table 1), and 48% were sites of fork slowing and/or DNA damage in Pfh1-depeleted cells (Figs 2D and 3D; S2 and S3 Tables). The likelihood of Pfh1 association was significantly greater for highly expressed genes than expected from other RNA polymerase II expressed genes (p≈0; hypergeometric test) as only 23% of all genes were Pfh1 associated.

A recent paper suggested that association of proteins with highly expressed genes in *S*. *cerevisiae* might be an artifact of the ChIP assay [45]. This interpretation is unlikely for our *S*. *pombe* analyses as a non-ChIP method, 2D gel analysis of replication intermediates, also showed that replication of three of three tested highly expressed RNA polymerase II transcribed genes is Pfh1-dependent [35]. Also, the peak strength at the highly transcribed genes was elevated in Pfh1-depleted cells compared to WT cells, suggesting that the association is not a ChIP artifact. Moreover, the genome-wide approach in this paper showed that high association of Pfh1 to highly transcribed genes was S phase specific (Fig 1), even though transcription of the genes is not S phase-limited. In addition, Pfh1, Cdc20, and γ-H2A all associate with ribosomal DNA (rDNA), probably the most highly transcribed region in all organisms, but high binding of all three does not occur over the 18 or 28S coding regions [39]. Also, the patterns of γ-H2A occupancy—low at the gene site and highest in flanking regions—are inconsistent with transcription artifacts (Fig 4), because the artifactual enrichment was observed to be high across the gene body. Thus, high binding of anti-Myc (used for Pfh1-13Myc), anti-HA (used for Cdc20-3HA), and anti-γ-H2A to highly transcribed genes is unlikely an artifact of their high transcription.

Fork pausing at highly transcribed genes as marked by high Pol2 occupancy is also demonstrated in budding yeast [49]. In contrast to *S*. *pombe*, where Pfh1 depletion increased fork pausing, pausing at these sites is not affected in the absence of ScRrm3 [49] or ScPif1 [18]. However, cells with a double deletion have not been tested, so it is possible that ScRrm3 and ScPif1 have overlapping functions in promoting replication past these genes.

Replication of multiple classes of genomic features is Pfh1-dependent. In addition to highly transcribed RNA polymerase II and III genes, this study identified several novel sites of Pfh1 association, such as NDRs, 3’ UTRs, and preferred meiotic DSB sites. In previous work, we showed that Pfh1 promotes fork movement past G4 motifs [39]. We analyzed whether the novel Pfh1 associations could be explained by overlap with G4 motifs or other Pfh1-associated features (S7 Fig). For example, NDRs often overlapped other significantly Pfh1-associated features, such as highly transcribed genes (32%), but none were sufficient to explain the association completely. Given their open state, it is likely that nucleosome-free regions are enriched for tightly bound proteins, other interactions, or formation of stable secondary structures that pause replication. The association with meiotic DSB hotspots in mitotically growing cells is largely driven by overlap with other elements that challenge replication; 92% (265 of 289 DSB) overlap another hard-to-replicate site identified in this study, most notably highly expressed genes and 3’ UTRs.

As noted above, several lines of evidence point to RNA polymerase III genes as the most Pfh1-dependent set of sites. Significant, but relatively smaller, fractions of the other Pfh1-associated features ultimately produce fork stalling and DNA damage in the absence of Pfh1 (Table 2). However, given the greater length of RNA polymerase II transcribed genes, if some cause fork stalling throughout their entire length, they may have a greater impact on fork progression genome-wide than the shorter tRNA genes.

The comprehensive analysis of sites of Pfh1 activity provided here demonstrates that, in addition to G4 motifs [39], there are many classes of hard-to-replicate sites that depend on Pfh1 for their proper replication. Many of these other sites, in particular RNA polymerase III transcribed genes, exhibit even stronger Pfh1-dependent effects than G4 motifs (Tables 1 and 2 and S3 Table). Altogether, Pfh1 supports DNA replication at thousands of diverse sites across the genome.

### Pfh1 is an accessory helicase that maintains proximity to the replisome

Although accessory helicases are well studied in bacteria, it is not clear for any of the bacterial enzymes if they are recruited to their sites of action or if they move with the replisome through the genome. Here, we present several lines of evidence that support the association of Pfh1 with the replisome in *S*. *pombe*. First, using a strain in which Pfh1 and Cdc20 were both epitope tagged, Pfh1 had strong binding to three different origins during S phase (Fig 5), even though these origins were not a peak of Pfh1 binding in asynchronous cells. Pfh1 was also bound to adjacent regions of the origins, although neither of these sites were Pfh1-dependent sites. The temporal patterns of binding to the sites adjacent to the origins were similar to those of Cdc20 in the same cells; however, we could not detect a movement of Pfh1 from an origin to its adjacent region, as we detected for Cdc20 at ars3002. This may be due to the role of DNA polymerase ε in replication initiation [65], technical challenges, and/or stochastic origin usage in different cells in the synchronized population [66]. If Pfh1 were recruited only to Pfh1-dependent sites, we would have expected to see low binding to these Pfh1-independent sites compared to Cdc20. Second, the levels of Pfh1 and Cdc20 binding to five other sites were similar, regardless of whether the site was Pfh1-dependent or independent. This again argues against a model in which Pfh1 is only recruited and associated with sites that require it for their normal replication. Third, genome wide analyses show that many sites of high Cdc20 binding overlap with Pfh1 peaks (230 of the 485 high Cdc20 binding sites are also sites of high Pfh1 binding). These data are most consistent with Pfh1 being in close proximity to the replisome throughout the genome.

Our MS analyses also provide independent evidence in support of the hypothesis that Pfh1 maintains proximity to the replisome and is likely a replisome component. During S phase, Pfh1 was associated with many replisomal proteins, including the MCM replicative helicase, subunits of the replicative polymerases, the processivity clamp PCNA, RPA, and four MMR proteins (Fig 6C). The association of Pfh1 with replisome proteins was either S phase limited or S phase enhanced and almost all of these associations were DNase-resistant. While it is possible that Pfh1 would have interactions with replisome subunits if it were recruited after replisome pausing, in the context of the evidence presented above, we conclude that Pfh1’s strong associations with replication proteins are likely due to protein-protein interactions, as expected if it is a replisome subunit.

### Pfh1 interacts with the FACT complex

Pfh1 also strongly associated with Spt16 and Pob3 (homologs of human SPT16 and SSRP1), two subunits of the heterodimeric evolutionarily conserved chromatin remodeling FACT complex (Table 3). In budding yeast, FACT promotes replication at replication conflicts past transcribed regions, and when FACT is depleted, ScRrm3 occupancy increases at highly transcribed RNA polymerase II and III genes [67]. Also, R-loop formation is elevated in FACT depleted cells, suggesting that the FACT complex resolves R-loop-mediated transcription-replication conflicts. The S phase association of FACT with Pfh1 suggests that the two cooperate to promote replication through these genes in *S*. *pombe* as they do in budding yeast. Pfh1 may facilitate replication at these genes by removing R-loops, as does budding yeast ScPif1 [27, 41]. In combination with its biochemical activities, the enrichment of Pfh1 at 3’ UTRs may also reflect a role in resolving R-loop-mediated transcription-replication conflicts.

### Summary

The genomes of all organisms are littered with hard-to-replicate sites. Accessory helicases promote the movement of the replisome past these natural impediments. Although multiple bacterial accessory helicases have been characterized, much less is known about accessory helicases in eukaryotes. Our genomic and proteomic analyses, in combination with previous work, show that Pfh1 promotes replication and suppresses DNA damage at hundreds of diverse, naturally occurring hard-to-replicate sites. The pattern of binding of Pfh1 through the genome combined with its association with many replisome components argues that it is in close proximity with the replisome to help it maneuver past these obstacles. For many of the Pfh1-sensitive sites (e.g., tRNA and other highly transcribed genes), replication slowed at these sites even in WT cells, but usually did not result in significant DNA damage. However, when Pfh1 was depleted, fork slowing intensified and DNA damage increased dramatically at hard-to-replicate sites. In budding yeast, the two Pif1 family helicases, ScPif1 and ScRrm3, collaborate to promote fork progression past replication impediments. Our results establish the importance of this helicase family in *S*. *pombe*, a eukaryotic organism that is deeply diverged from *S*. *cerevisiae* and shares many genomic characteristics in common with higher eukaryotes. As the replication-impeding obstacles found in budding and fission yeasts are ubiquitous across genomes of other organisms, accessory helicases are likely to be required in all organisms, even though helicases that act at most of these sites have not been identified in higher eukaryotes. Thus, we propose that Pif1 family helicases present in multicellular eukaryotes also act as accessory helicases to promote fork progression and preserve genome stability.

## Materials and Methods

### ChIP analysis

ChIP-qPCR were performed in *cdc25-22 leu1-32*::*PJK148-Pfh1-13MYC-kanmx6 cdc20*::*cdc20-3HA-kanmx6* cells (S8 Table). Asynchronous samples were grown at 25°C. The G2 phase cells were arrested at 37°C and collected after 4h arrest. To perform ChIP-qPCR in synchronized cells, cells were arrested at 37°C for 4h and released at permissive temperature at 25°C. Samples from the cell synchrony were collected for FACS analysis and ChIP-qPCR at 0, 20, 40, 60, 65, 70, 75, 80, 85, 90, 95, 115, 140, and 165 min. The ChIP experiments were performed as described previously [35]. Briefly, cells were cross-linked in 1% formaldehyde at 25°C for 5 min. The chromatin was sheared to an average of ~300 bp with a Covaris E220 system. G2 phase and asynchronous cells were immunoprecipitated with anti-Myc antibody (Clontech Cat. nr 631206). The synchronous cells were divided (3/4 of sample for Pfh1-Myc and 1/4 of sample for Cdc20-HA) and immunoprecipitated with either anti-Myc antibody or anti-HA antibody (Santa Cruz Biotechnology Cat. nr Sc7392x). Both immunoprecipitated DNA and the corresponding input amount for each sample were purified and quantified by real-time PCR, using the primer pairs described in S9 Table. At least two biological replicates were performed for each synchronous ChIP analysis. To calculate the average ratio of the peak binding time in Fig 5F, the IP/Input of two biological replicates were used. The time for peak binding (highest binding) in replicate 1 was 95 min and 70 min for replicate 2. The difference between the highest peak binding for the two synchronies was due to different start times of S phase after G2 release, which was detected by FACS analysis.

### Flow cytometric analysis (FACS)

*S*. *pombe* cells were collected in 165 mM EDTA, 0.1% sodium azide 70% EtOH. Cells were pelleted, washed in 100% EtOH, and stored at 4°C. In preparation for FACS analysis, approximately 2 x 10^6^ cells were washed in 3 ml of 50 mM Na citrate, pH 7.2, and incubated overnight at 37°C in 0.5 ml 50 mM Na citrate plus 0.1 mg/ml RNaseA. Following sonication, cells were incubated in 1 μM Sytox Green (Molecular Probes) at room temperature for 30 minutes. Cells were analyzed using a FACScan single laser fixed-alignment benchtop analyzer.

### Genomic annotation enrichment analysis

We tested for enrichment between the genomic locations of the ChIP-seq peaks with sixteen sets of genomic annotations (Table 1). The ChIP-seq data for Pfh1, Cdc20, and γ-H2A are available in GEO data set GSE59178 [39]. All peaks analyzed are available in S1 Table. We took the location of genes, coding sequences, essential genes, dubious genes, 3’ and 5’ UTRs, promoters, tRNAs, centromeres, 5S rRNAs, long terminal repeats, and the mating type loci from PomBase [68]. We also considered the locations of meiotic DSB hotspots [69], NDRs [70] using podbat [71], origins of replication [72], the 500 most highly expressed genes from expression data collected across several growth conditions [44], and G4 motifs [39]. For Pfh1 and Cdc20 sites, regions within 300 bp were considered associated, and for γ-H2A peaks, we considered a window of 5 kb, since DNA damage results in elevated phosphorylation in a window of roughly this size around the break [50]. Overlaps between sets of genomic locations were calculated using BEDTools [73].

To determine if the observed association between two sets of genomic features, such as Pfh1 binding peaks and tRNA genes, was significantly greater than expected, we followed our previously described procedure for generating GC-content-aware empirical p-values [18, 39, 74]. In brief, we compared the observed number of overlaps to the number obtained when scrambling the peak locations 1,000 times in a manner that preserved the length, chromosome, and GC content of the regions. The number of randomized sets of peaks that obtain as many or more overlaps with the annotation (e.g., tRNA) as the actual peaks is the empirical p-value. If no random sets meet the level of overlap with the actual peaks, then the p-value is reported as < 0.001. We accounted for the testing of each set of ChIP-seq peaks with multiple genomic features using the Bonferroni correction.

### Immunoaffinity purification (IP)

Strains expressing Pfh1-GFP were previously described (S8 Table) [34]. Briefly, the pJK148-integrating vector was used to express Pfh1-GFP from the leu1 locus using the endogenous Pfh1 promoter. The control IP strain expressed GFP-NLS from the leu1 locus under the control of the P3nmt promoter. The GFP-NLS construct was generated in pJK148 using the plasmid pFA6a-kanMX6-P3nmt1-GFP tagging construct as a PCR template with the addition of two SV40 nuclear localization signals introduced by PCR primers.

For cell synchronization, *cdc25-22* strains were grown to early mid-log (0.5 x 10^7^ cells/ml) at the permissive temperature of 25°C. The cells were collected by filtration and shifted to 37°C for G2 arrest. After 4 hours of incubation at 37°C, the media was quickly cooled (2 minutes by swirling in an ice bath) to 25°C for synchronized growth. Cells harvested at the G2 time point were collected at the end of the 4 hour 37°C incubation. Cells harvested at the S phase time point were collected at 84 minutes, corresponding to the start of replication. Cell cycle progression and the timing of DNA replication were confirmed by FACS analysis. Strains expressing either Pfh1-GFP (yKM333) or GFP alone (yKM346) from the *S*. *pombe leu1-32* locus in a strain background containing the *cdc25-22* mutant were confirmed to progress normally through the cell cycle by FACS analysis.

Two liters of *S*. *pombe* cells were either synchronized or grown to mid-log and harvested by centrifugation at 4°C for 10 minutes at 4,000 rpm (2,704 x g). Cell pellets were resuspended in freezing buffer (20 mM Na-HEPES, 1.2% polyvinylpyrrolidone (W/V), pH 7.4 containing a protease inhibitor cocktail (v/v 1/100) (Sigma) and frozen as cell droplets in liquid nitrogen [75]. Frozen cell droplets were cryogenically ground using a Retsch MM301 Mixer Mill (20 cycles x 2.5 min at 30 Hz) (Retsch, Newtown, PA) to achieve greater than 85% cell lysis, as assessed using light microscopy. Approximately 12 grams of ground, frozen cells were resuspended in lysis buffer (100mM Hepes KOH, pH 7.9, 300mM potassium acetate, 10mM magnesium acetate, 10% glycerol, 0.1% NP-40, 2mM EDTA, 2mM B-glycerophosphate, 50mM NaF, 10mM NaVO_4_, 1mM DTT, protease inhibitor cocktail (Roche) in a ratio of 5ml of lysis buffer per 1 gram of cells. Cells were gradually added to the lysis buffer with continuous mixing to avoid cell clumps. Lysis buffer conditions of varying salt concentrations (50–900 mM potassium acetate) were optimized for efficiency of Pfh1-GFP purification. Cell lysate was homogenized using a PT 10–35 Poyltron (Kinematica) for 3 sets of 10 seconds (30 seconds total) with 1 minute on ice in between each set. Insoluble material from the cell lysate was removed by centrifugation at 8000 rpm (9265 x g) for 10 minutes at 4°C. For immunoaffinity purification of Pfh1-GFP, the cell lysate supernatant was incubated for 30 minutes at 4°C with approximately 20 mg of M-270 epoxy magnetic beads (Invitrogen Dynal) conjugated with 50 μg of in-house developed rabbit polyclonal anti-GFP [51]. Following incubation, the beads were collected and washed six times with 1ml of lysis buffer. Proteins were eluted from the beads by incubation with 40 μl of 1x LDS sample buffer (Life Technologies) by shaking for 10 minutes at room temperature, followed by 10 minutes at 70°C. Eluted proteins were alkylated with 50 mM chloroacetamide for 30 minutes at room temperature in the dark.

To determine if DNA mediates the interactions of Pfh1-GFP, chromosomal DNA of the cell lysate was incubated with an excess of DNaseI (640 U/g of cell or ~70 ug/ml, 30 min. at 4°C) during the IP experiment immediately before the addition of conjugated beads (S6 Fig). DNaseI digestion was assessed by precipitation of DNA from the cell lysate before and after DNaseI treatment and was visualized on an agarose gel by ethidium bromide staining (S6B Fig). Low molecular weight DNA was observed in samples of cell lysate taken before and after DNaseI treatment (S6B Fig, lanes 1 and 2), suggesting that chromosomal DNA was degraded during earlier steps of the IP experiment before the addition of DNaseI. Enzymatic activity of DNaseI was not affected in the cell lysate, as demonstrated by the digestion of plasmid DNA (pDNA) that was added to a sample of the cell lysate prior to DNaseI digestion (S6B Fig, lanes 3 and 4).

### Mass spectrometry analysis

Following immunoaffinity purifications (n = 2) of Pfh1-GFP or NLS-GFP from S phase cells, the isolated protein complexes were separated by gel electrophoresis. Samples were digested in-gel with trypsin and peptides were extracted from gel pieces using 0.5% formic acid. Peptides were concentrated by vacuum centrifugation to approximately 12 μl. 4uL of the sample was injected for nanoscale liquid chromatography tandem mass spectrometry (nLC-MS/MS) analysis on a Dionex Ultimate 3000 RSLC directly coupled to an LTQ-Orbitrap Velos with (ETD (ThermoFisher Scientific) instrument. Data were automatically acquired with MS^2^ fragmentation of the top 20 most intense precursor ions by collision-induced dissociation (CID). Parameters for data processing were also followed as described previously [56]. Briefly, raw files containing MS^2^ data were extracted by Proteome Discoverer (version 1.3; Thermo Scientific) and uploaded to SEQUEST (version 1.20) and searched against a compiled database of the yeast protein sequences from *S*. *cervisiae* and *S*. *pombe*. Post-search validation of the SEQUEST data was conducted by an X! Tandem algorithm in Scaffold (version Scaffold_3_00_04; Proteome Software) using the following filter selections to reduce peptide and protein global false discovery rate to < 1%: 99% protein confidence, 95% peptide confidence, and a minimum of two unique peptides per protein.

### Assessing specificity of interactions

Protein interactions were accessed for specificity and enrichment (Pfh1-GFP vs. NLS-GFP control) using the SAINT (significance analysis of interactome) algorithm [52]. A SAINT confidence score cutoff of 0.80 was selected to retain high confidence Pfh1 interactions.

### Building protein interaction networks

The protein interaction partners of Pfh1 were placed in the context of known protein interaction data from STRING (v9.1, medium confidence level, text mining = OFF) [76] and visualized using Cytoscape [77]. Within Cytoscape, nodes represent proteins that interact with Pfh1, and edges represent previously reported interactions among proteins in the network. To determine enrichment of protein interactions within the network relative to the background proteome, NSAF (normalized spectrum abundance factor) values were calculated to take into account protein length and the total number of spectra present in each individual IP experiment. NSAF values were normalized to proteome abundance (PAX) values for S phase *S*. *pombe* (pax-db.org), and the NSAF/PAX ratio was mapped onto each node (node color).

## Supporting Information

S1 Fig Pfh1 is bound to all DNA regions tested. Samples from asynchronous cells either expressing Pfh1-13Myc or an untagged control were chromatin immunoprecipitated using an anti-Myc antibody. Data for Pfh1-13Myc were also shown in Fig 1. The immunoprecipitated DNA was quantified using quantitative PCR with primers specific for *hsp90*^*+*^, *tdh1*^*+*^, *adh1*^*+*^, *hta1*^*+*^, and *ade6*^*+*^. Data are shown as immunoprecipitated DNA divided by input DNA. Data represent the mean of three independent replicates and error bars are standard deviation. The p-value was determined by two-tailed Student’s t-test and “*” indicates p < 0.05.
(PDF)

S2 Fig Fork pausing and DNA damage are increased at G4 motifs in the absence of Pfh1. (A) Scatter plot comparing Cdc20 peak strength (-10*log10(p-value)) at G4 motifs in WT and Pfh1-depleted cells. Each point represents a genomic region with a Cdc20 occupancy peak in at least one context. If a peak was not present in a context, it is plotted at 0 on the corresponding axis. The number in the bottom right of each plot gives the percentage of peaks stronger in Pfh1-depleted cells. (B) Scatter plot comparing γ-H2A peak strength at G4 motifs in WT and Pfh1-depleted cells. The layout is the same as in part (A).
(PDF)

S3 Fig Pfh1, Cdc20, and γ-H2A signals surrounding all tRNA genes. Details are as in Fig 3.
(PDF)

S4 Fig Pfh1, Cdc20, and γ-H2A signals surrounding all 5S rRNA genes. Details are as in Fig 3.
(PDF)

S5 Fig Interaction of Pfh1-GFP with DNA replication proteins Pol2 and the Mcm helicase complex observed in asynchronous cells. Immunoaffinity-purification of Pfh1-GFP from asynchronous cells. Proteins were resolved by SDS-PAGE and visualized by Coomassie stain. Peptides of identified proteins were confirmed by MS with nanoLC LTQ Orbitrap CID analyses. Pfh1 interaction was observed with proteins involved in DNA replication and repair such as the replicative DNA polymerase Pol2, members of the replicative Mcm helicase complex, and the Ku70/Ku80 heterodimer required for DNA repair and telomere maintenance in eukaryotic cells.
(PDF)

S6 Fig Immunoaffinity-purification of Pfh1-GFP is not affected by DNaseI treatment of the cell lysate. **(A) Proteins were resolved by SDS-PAGE and visualized by Coomassie stain.** (B) Ethidium bromide stained agarose gel of precipitated DNA from an aliquot of the cell lysate before and after DNAseI treatment (lanes 1 and 2) and with the addition of plasmid DNA (lanes 3 and 4) as a control for DNase I activity in the experimental lysis buffer.
(PDF)

S7 Fig **(A) Fraction of Pfh1-bound features of a given type that overlap Pfh1-bound instances of each other tested feature type.** (B) Fraction of Pfh1-sensitive (in terms of Cdc20) features of a given type that overlap Pfh1-bound instances of each other tested feature type. In each panel, the fraction is given in terms of the y-axis feature. For example, the 0.09 in the second entry in the first row of (A), indicates that 9% of the Pfh1-bound 3’ UTRs overlap a Pfh1-bound 5’ UTR.
(PDF)

S1 Table Genome-wide Peak Locations for Pfh1, Cdc20 in WT, Cdc20 in Pfh1-depleted cells, γ-H2A in WT, and γ-H2A in Pfh1-depleted Cells. (XLSX)

S2 Table Genomic Features Associated with Cdc20 Occupied Regions in WT and Pfh1-depleted Cells. (XLSX)

S3 Table Cdc20 and γ-H2A Peaks Are Stronger in Pfh1-depleted Cells than WT Cells. (XLSX)

S4 Table Analysis of Pfh1-binding at sites that are sensitive to the depletion of Pfh1. (XLSX)

S5 Table Genomic Features Associated with γ-H2A Occupied Regions in WT and Pfh1-depleted Cells. (XLSX)

S6 Table Genomic Features Associated with shared and unique Cdc20 and γ-H2A Occupied Regions. (XLSX)

S7 Table Proteins Interacting with Pfh1 in either S or G2 Phase Identified by Immunoaffinity Purification Mass Spectrometry. (XLSX)

S8 Table *S*. *pombe* strains used in this study. (DOCX)

S9 Table Oligonucleotides used for qPCR experiments. (DOCX)
