# Supplementary figures and images for: Pfh1 Is an Accessory Replicative Helicase that Interacts with the Replisome to Facilitate Fork Progression and Preserve Genome Integrity
Source: PLoS Genet. 2016 Sep 9;12(9):e1006238. doi: 10.1371/journal.pgen.1006238 (PMC5017727; doi:10.1371/journal.pgen.1006238)

Fig S1

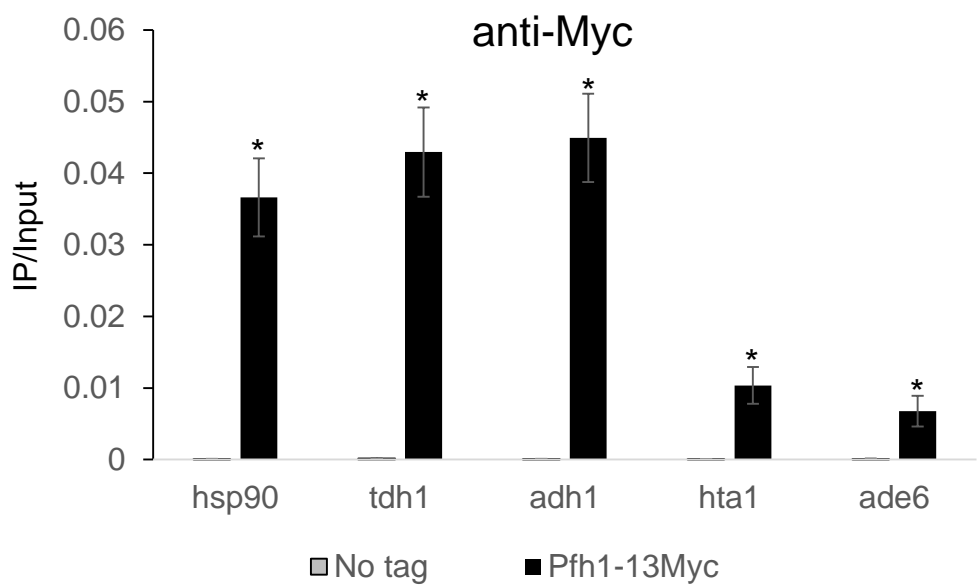

Supplement: S1 Fig — Samples from asynchronous cells either expressing Pfh1-13Myc or an untagged control were chromatin immunoprecipitated using an anti-Myc antibody. Data for Pfh1-13Myc were also shown in Fig 1. The immunoprecipitated DNA was quantified using quantitative PCR with primers specific for hsp90+, tdh1+, adh1+, hta1+, and ade6+. Data are shown as immunoprecipitated DNA divided by input DNA. Data represent the mean of three independent replicates and error bars are standard deviation. The p-value was determined by two-tailed Student’s t-test and “*” indicates p < 0.05. (PDF) [file pgen.1006238.s001.pdf]

Fig S2

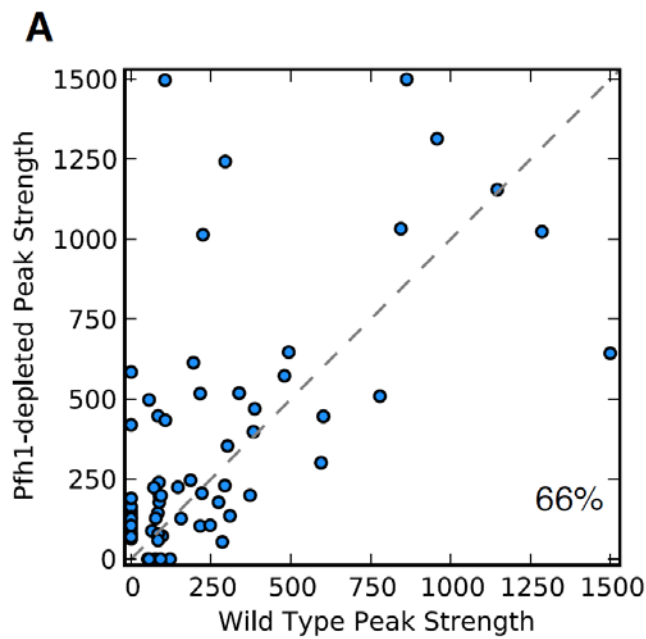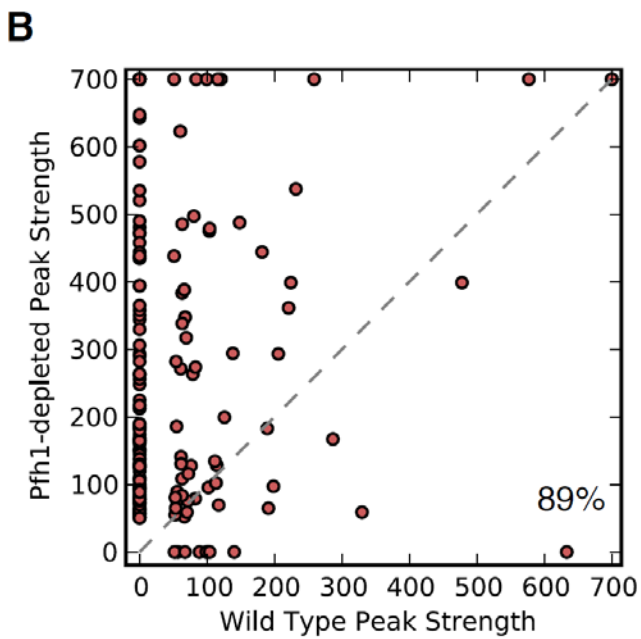

Supplement: S2 Fig — (A) Scatter plot comparing Cdc20 peak strength (-10*log10(p-value)) at G4 motifs in WT and Pfh1-depleted cells. Each point represents a genomic region with a Cdc20 occupancy peak in at least one context. If a peak was not present in a context, it is plotted at 0 on the corresponding axis. The number in the bottom right of each plot gives the percentage of peaks stronger in Pfh1-depleted cells. (B) Scatter plot comparing γ-H2A peak strength at G4 motifs in WT and Pfh1-depleted cells. The layout is the same as in part (A). (PDF) [file pgen.1006238.s002.pdf]

Fig S5

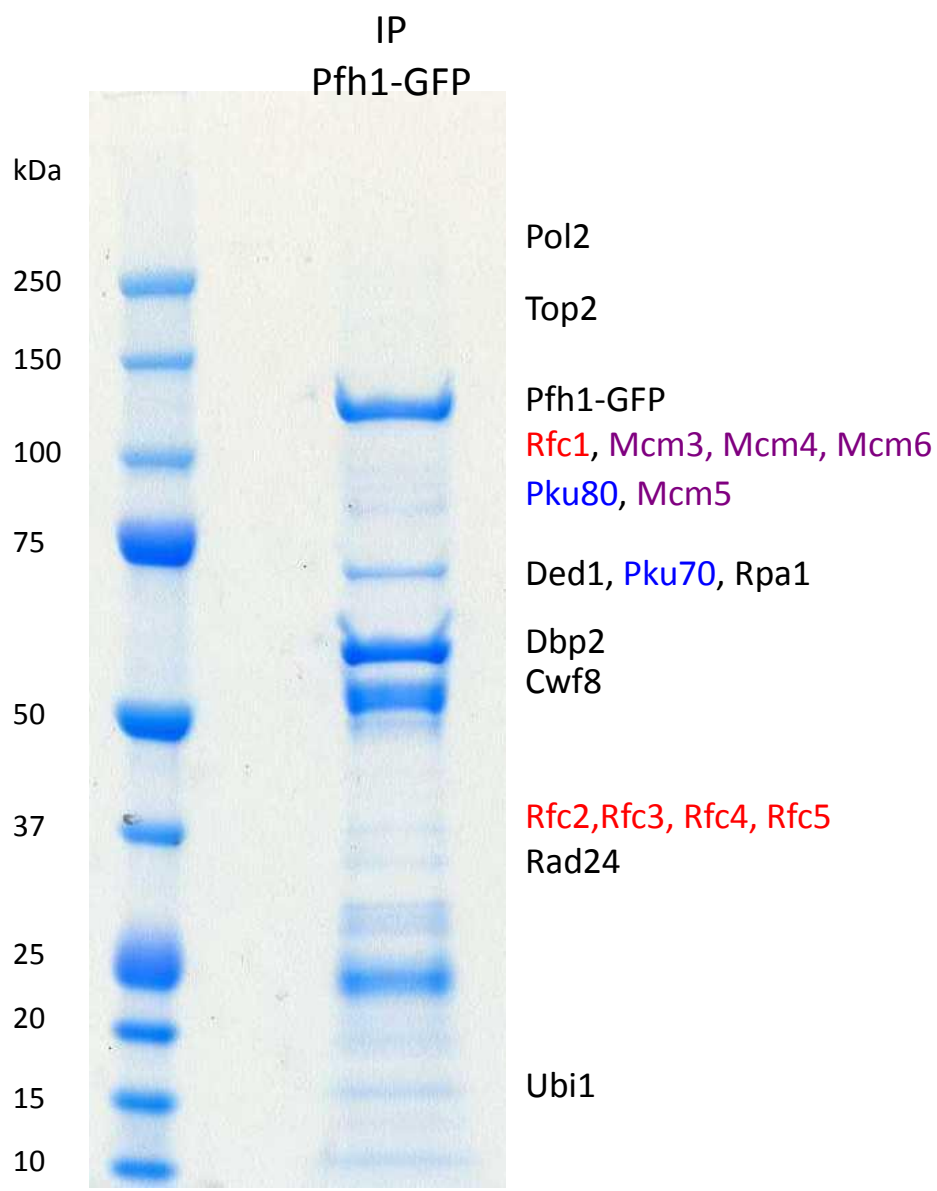

Supplement: S5 Fig — Immunoaffinity-purification of Pfh1-GFP from asynchronous cells. Proteins were resolved by SDS-PAGE and visualized by Coomassie stain. Peptides of identified proteins were confirmed by MS with nanoLC LTQ Orbitrap CID analyses. Pfh1 interaction was observed with proteins involved in DNA replication and repair such as the replicative DNA polymerase Pol2, members of the replicative Mcm helicase complex, and the Ku70/Ku80 heterodimer required for DNA repair and telomere maintenance in eukaryotic cells. (PDF) [file pgen.1006238.s005.pdf]

Fig S6

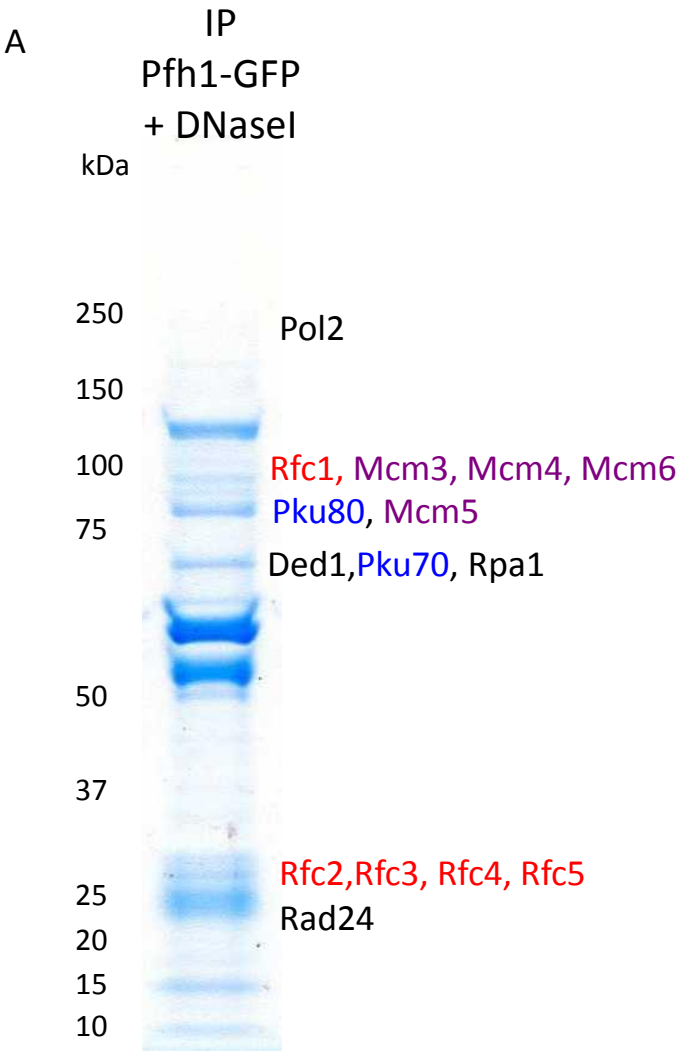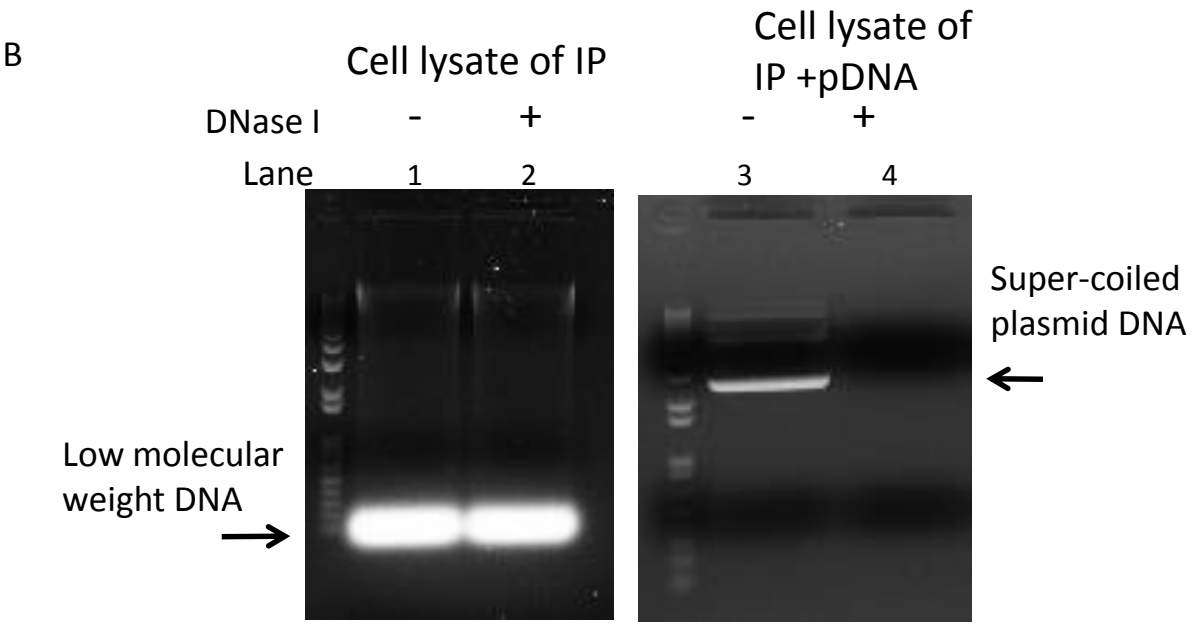

Supplement: S6 Fig — (A) Proteins were resolved by SDS-PAGE and visualized by Coomassie stain. (B) Ethidium bromide stained agarose gel of precipitated DNA from an aliquot of the cell lysate before and after DNAseI treatment (lanes 1 and 2) and with the addition of plasmid DNA (lanes 3 and 4) as a control for DNase I activity in the experimental lysis buffer. (PDF) [file pgen.1006238.s006.pdf]

Fig S7

A

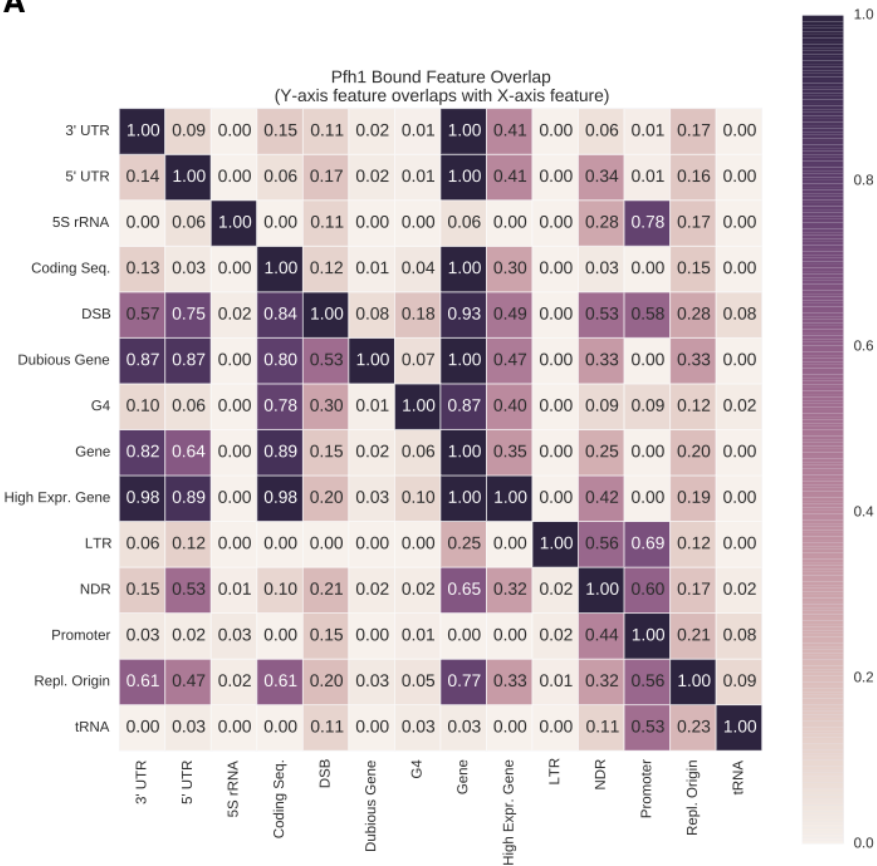

B

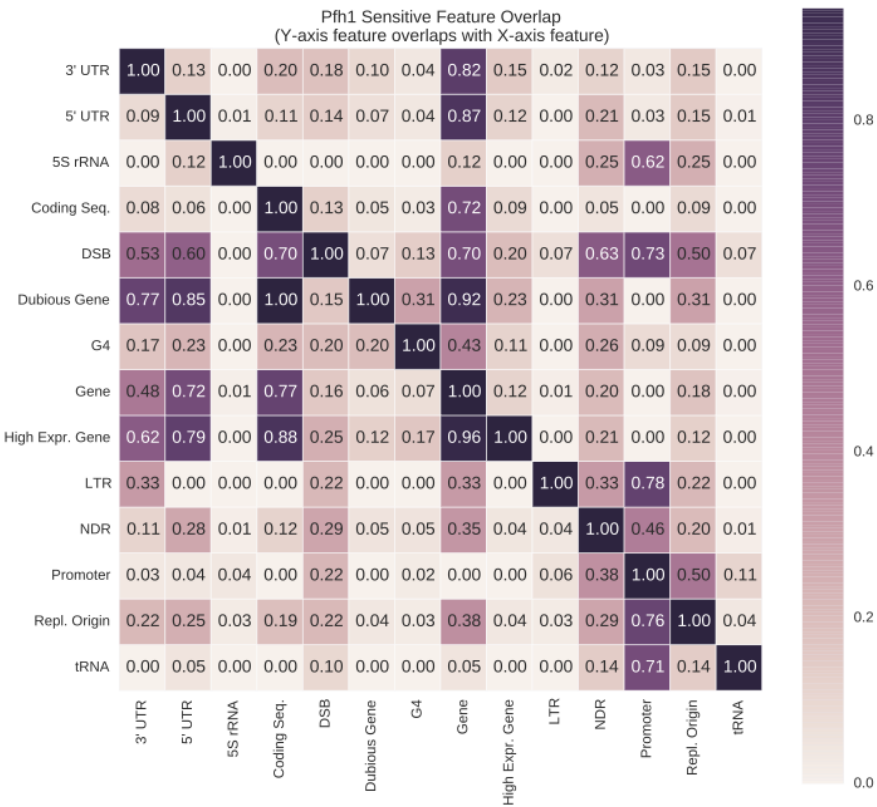

Supplement: S7 Fig — (A) Fraction of Pfh1-bound features of a given type that overlap Pfh1-bound instances of each other tested feature type. (B) Fraction of Pfh1-sensitive (in terms of Cdc20) features of a given type that overlap Pfh1-bound instances of each other tested feature type. In each panel, the fraction is given in terms of the y-axis feature. For example, the 0.09 in the second entry in the first row of (A), indicates that 9% of the Pfh1-bound 3’ UTRs overlap a Pfh1-bound 5’ UTR. (PDF) [file pgen.1006238.s007.pdf]
