# Supplementary material for: Pfh1 Is an Accessory Replicative Helicase that Interacts with the Replisome to Facilitate Fork Progression and Preserve Genome Integrity
Source: PLoS Genet. 2016 Sep 9;12(9):e1006238. doi: 10.1371/journal.pgen.1006238 (PMC5017727; doi:10.1371/journal.pgen.1006238)
Supplement: S3 Fig — Details are as in Fig 3. (PDF) [file pgen.1006238.s003.pdf]

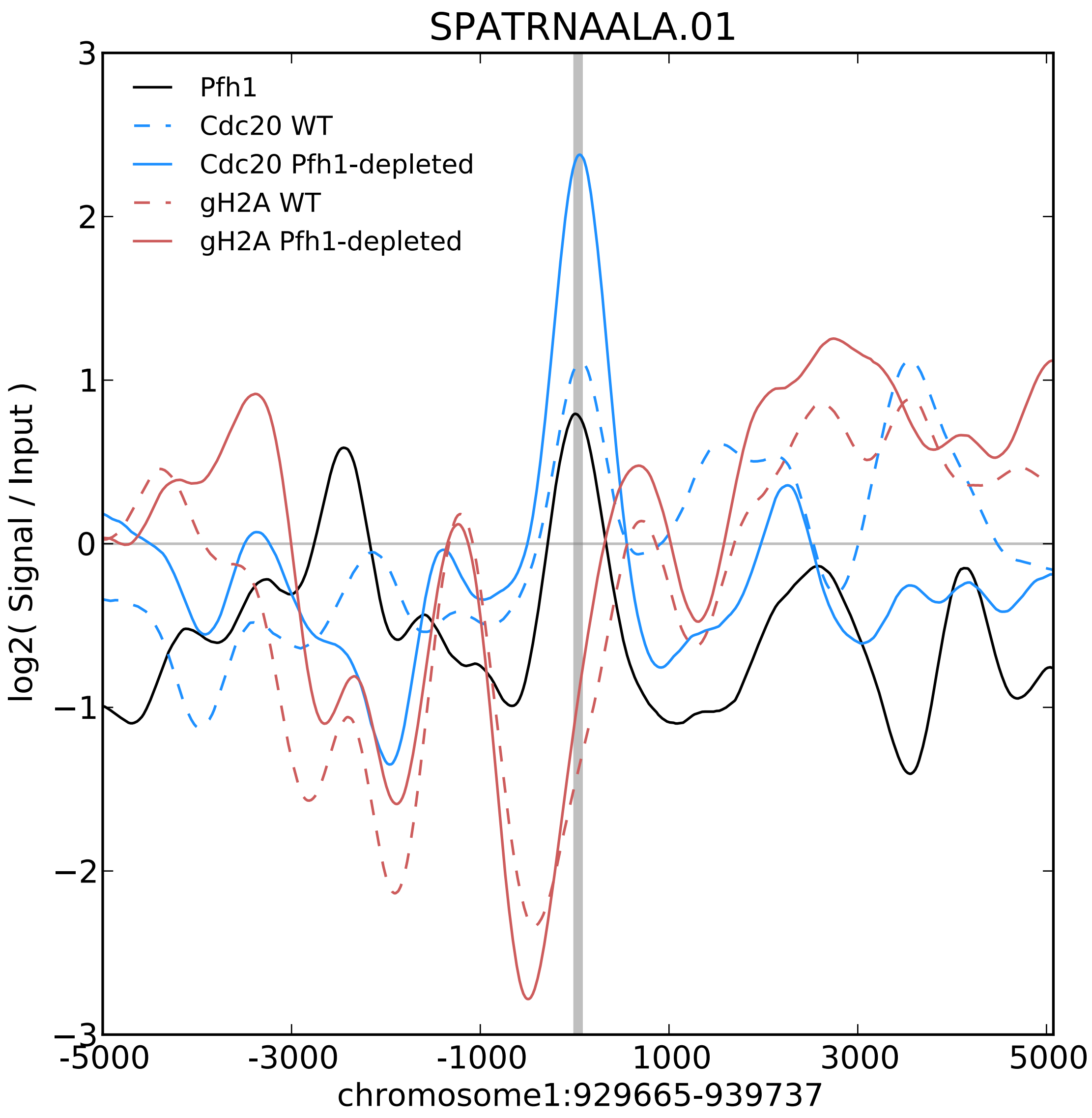
SPATRNAALA.01
Pfh1
Cdc20 WT
Cdc20 Pfh1-depleted
gH2A WT
gH2A Pfh1-depleted
3
2
1
0
−1
−2
−3
log2(Signal / Input)
-5000
-3000
-1000
1000
3000
5000
chromosome1:929665-939737

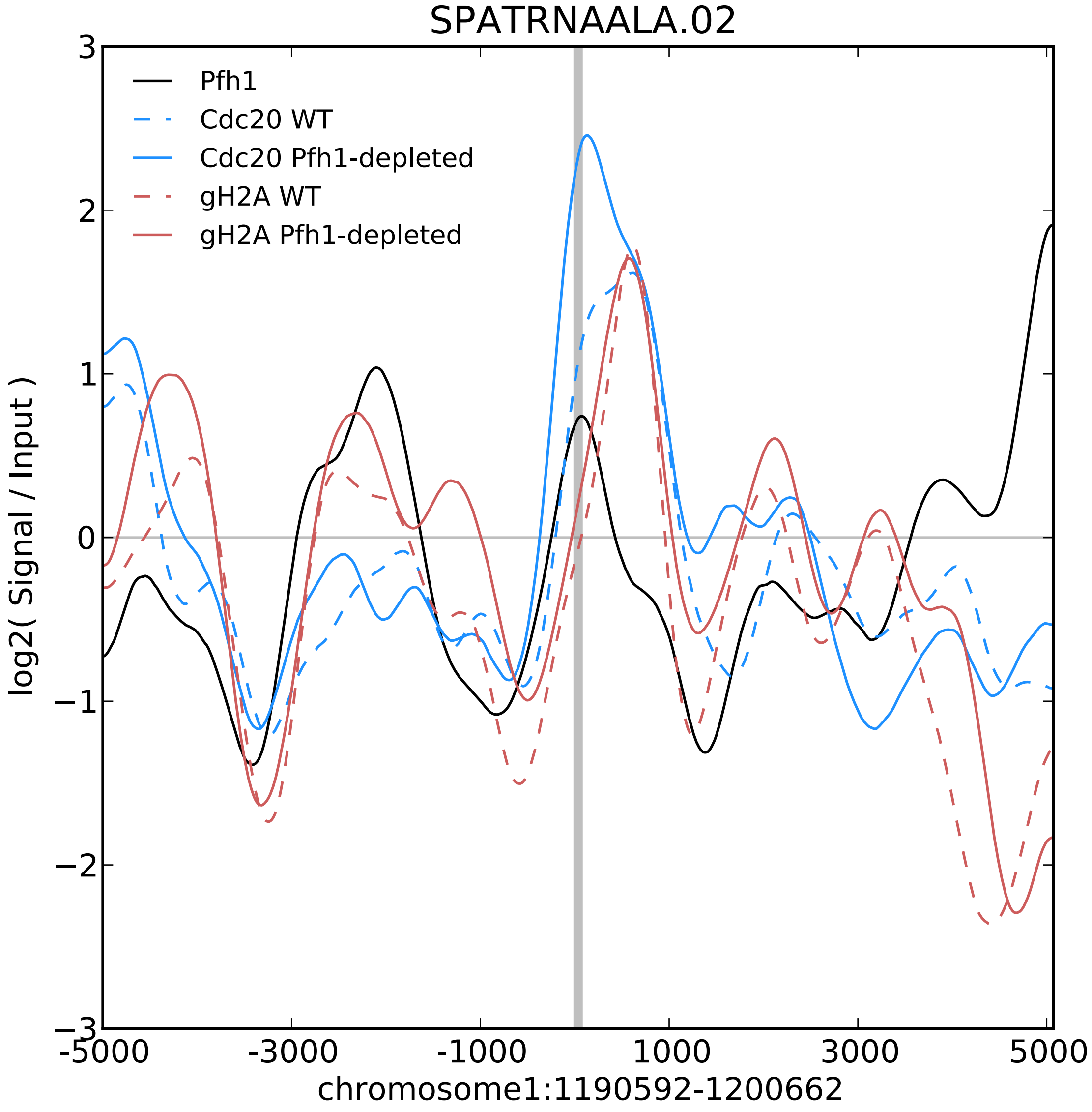

SPATRNAALA.02
Pfh1
Cdc20 WT
Cdc20 Pfh1-depleted
gH2A WT
gH2A Pfh1-depleted
log2(Signal / Input)
3
2
1
0
−1
−2
−3
-5000
-3000
-1000
1000
3000
5000
chromosome1:1190592-1200662

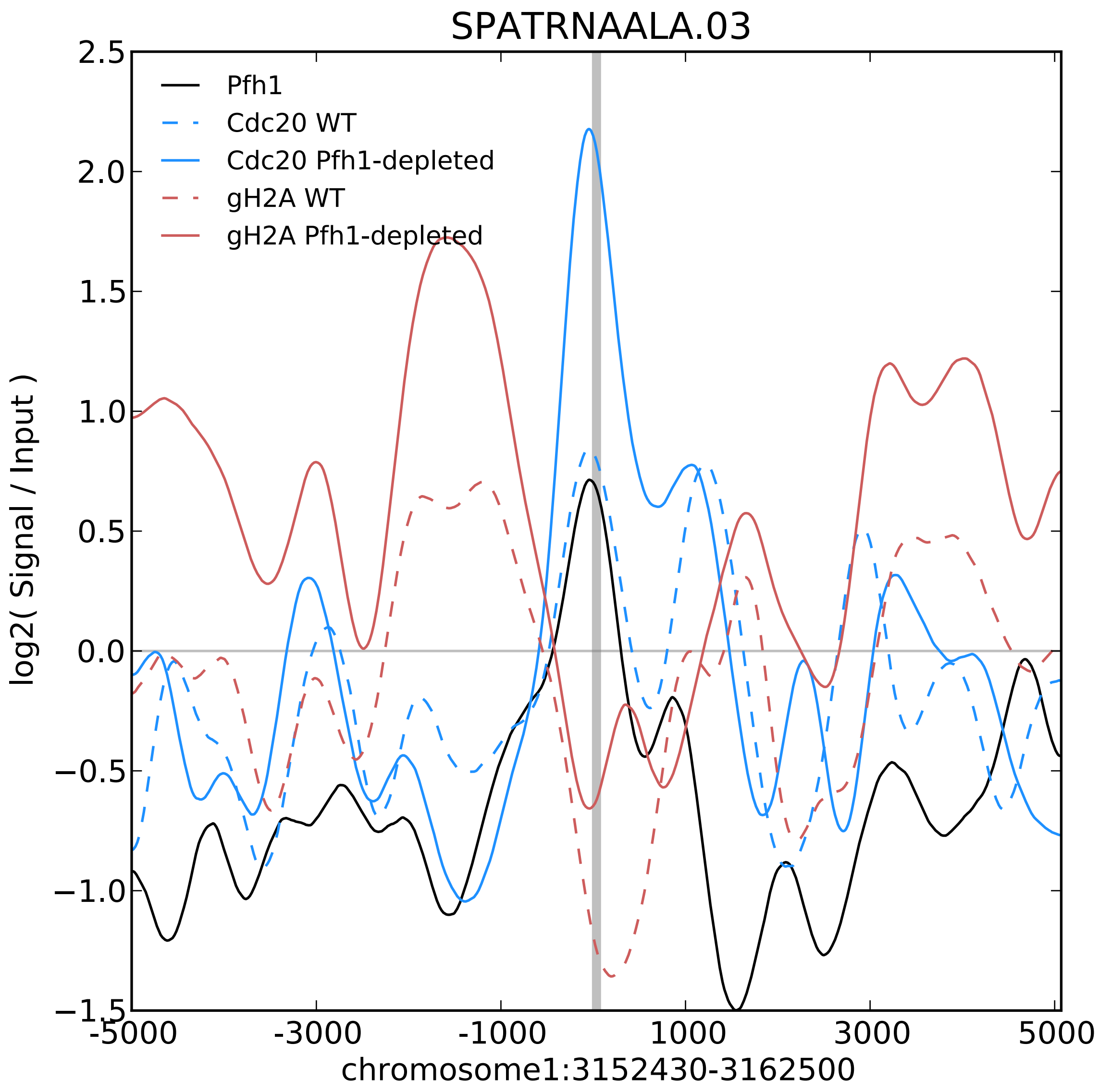

SPATRNAALA.03
Pfh1
Cdc20 WT
Cdc20 Pfh1-depleted
gH2A WT
gH2A Pfh1-depleted
2.5
2.0
1.5
1.0
0.5
0.0
−0.5
−1.0
−1.5
log2(Signal / Input)
-5000
-3000
-1000
1000
3000
5000
chromosome1:3152430-3162500

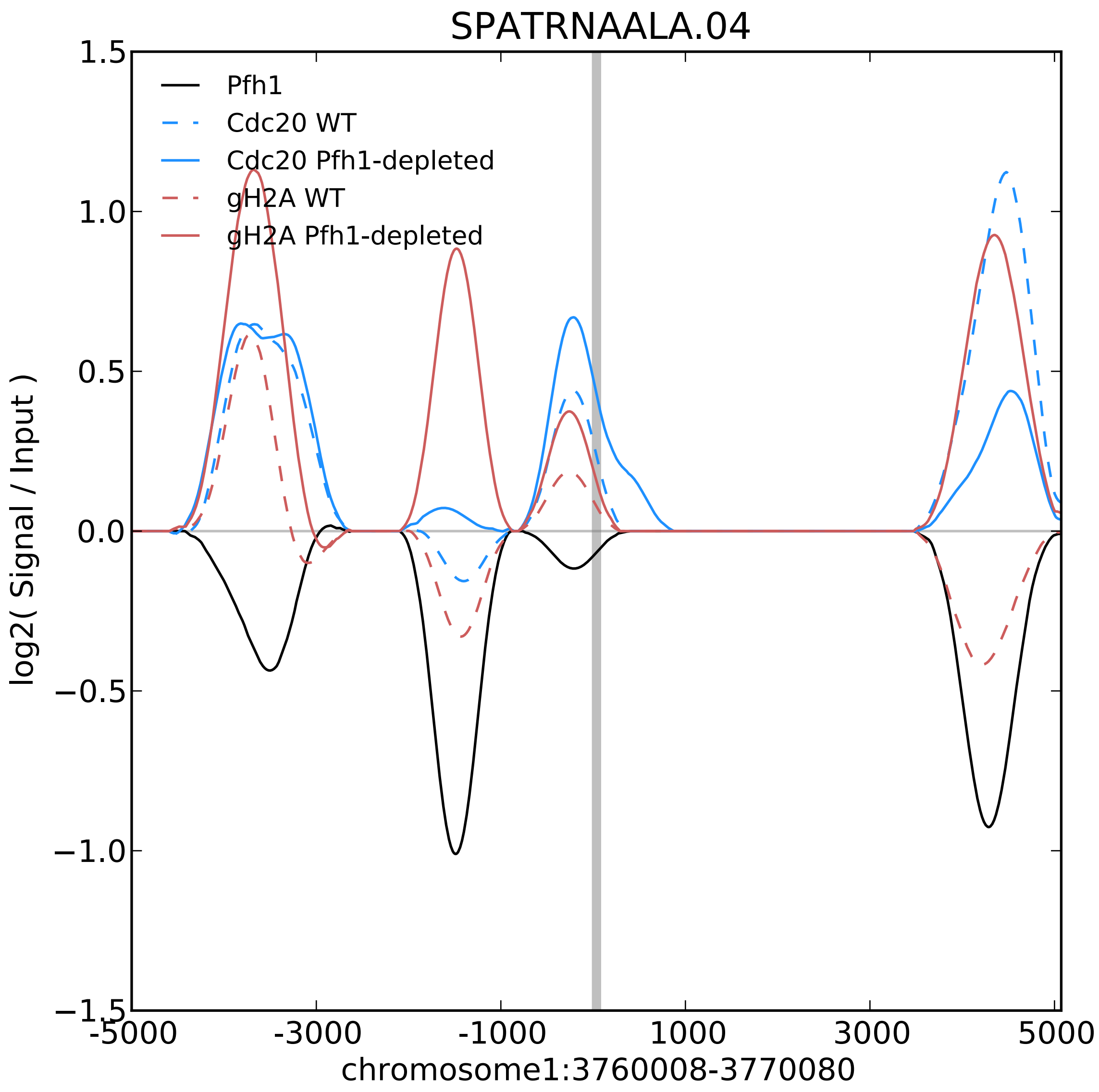
SPATRNAALA.04
Pfh1
Cdc20 WT
Cdc20 Pfh1-depleted
gH2A WT
gH2A Pfh1-depleted
log2(Signal / Input)
1.5
1.0
0.5
0.0
−0.5
−1.0
−1.5
-5000
-3000
-1000
1000
3000
5000
chromosome1:3760008-3770080

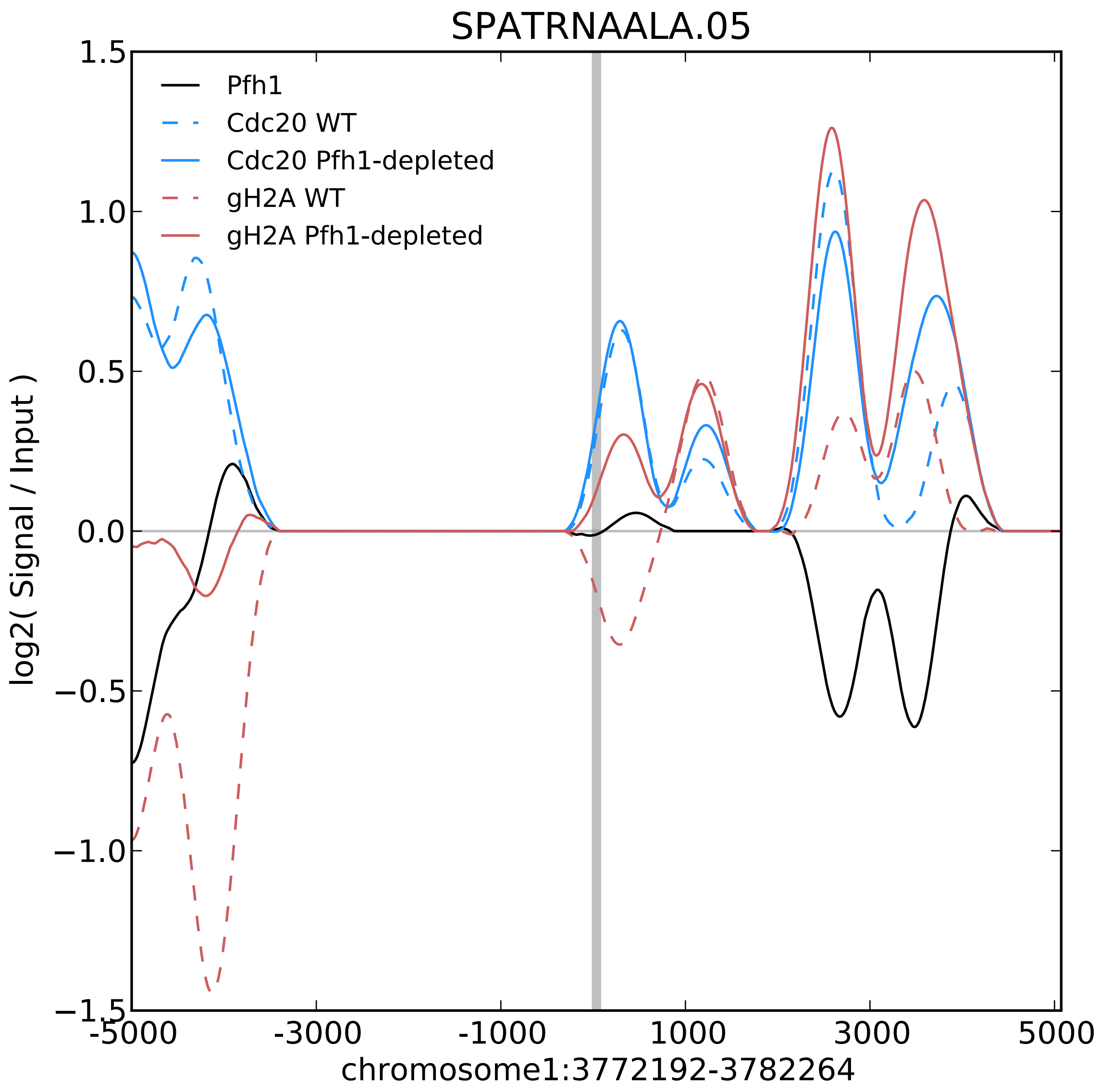
SPATRNAALA.05
Pfh1
Cdc20 WT
Cdc20 Pfh1-depleted
gH2A WT
gH2A Pfh1-depleted
1.5
1.0
0.5
0.0
−0.5
−1.0
−1.5
log2(Signal / Input)
-5000
-3000
-1000
1000
3000
5000
chromosome1:3772192-3782264

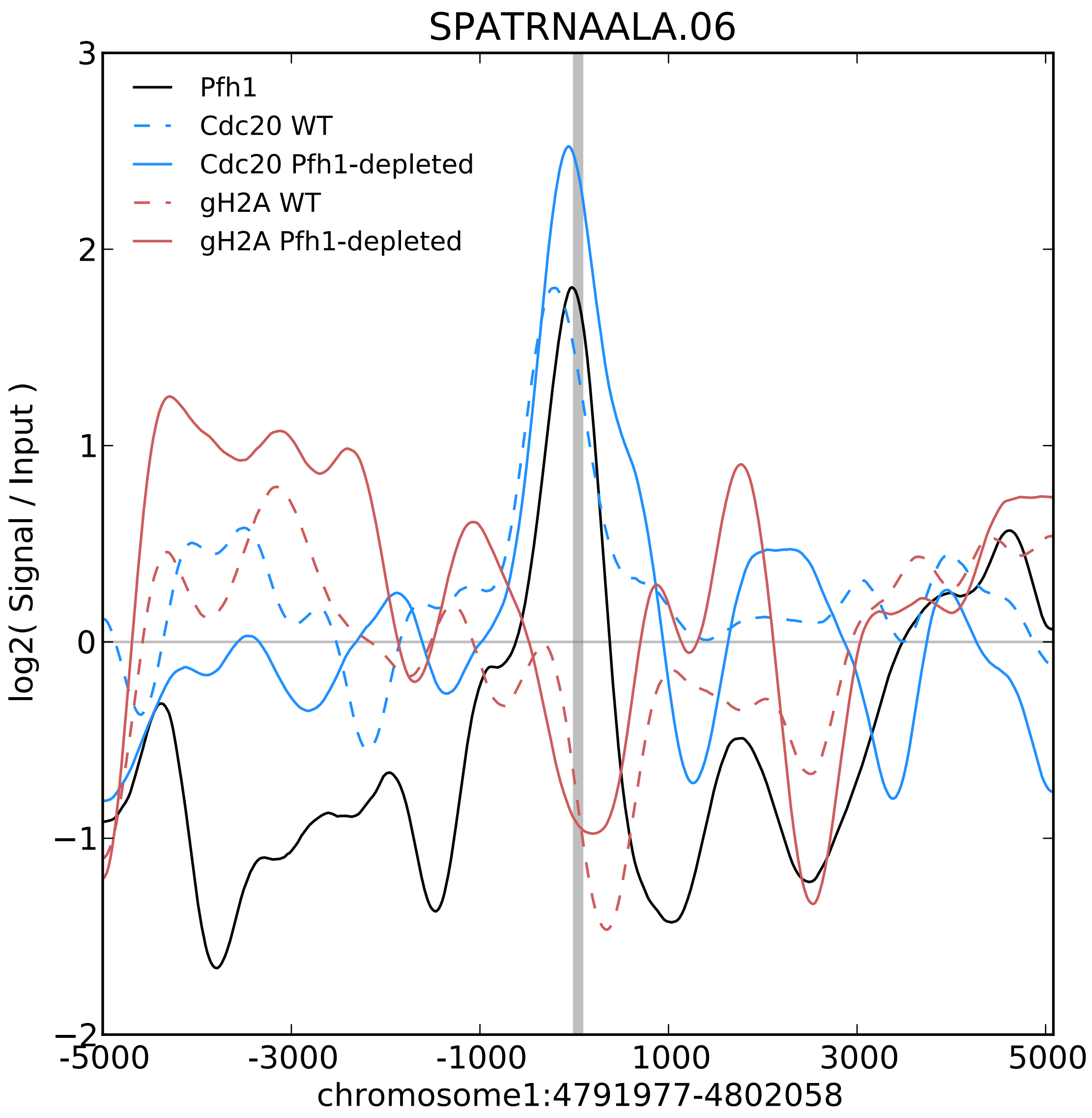

SPATRNAALA.06
Pfh1
Cdc20 WT
Cdc20 Pfh1-depleted
gH2A WT
gH2A Pfh1-depleted
log2(Signal / Input)
3
2
1
0
−1
−2
-5000
-3000
-1000
1000
3000
5000
chromosome1:4791977-4802058

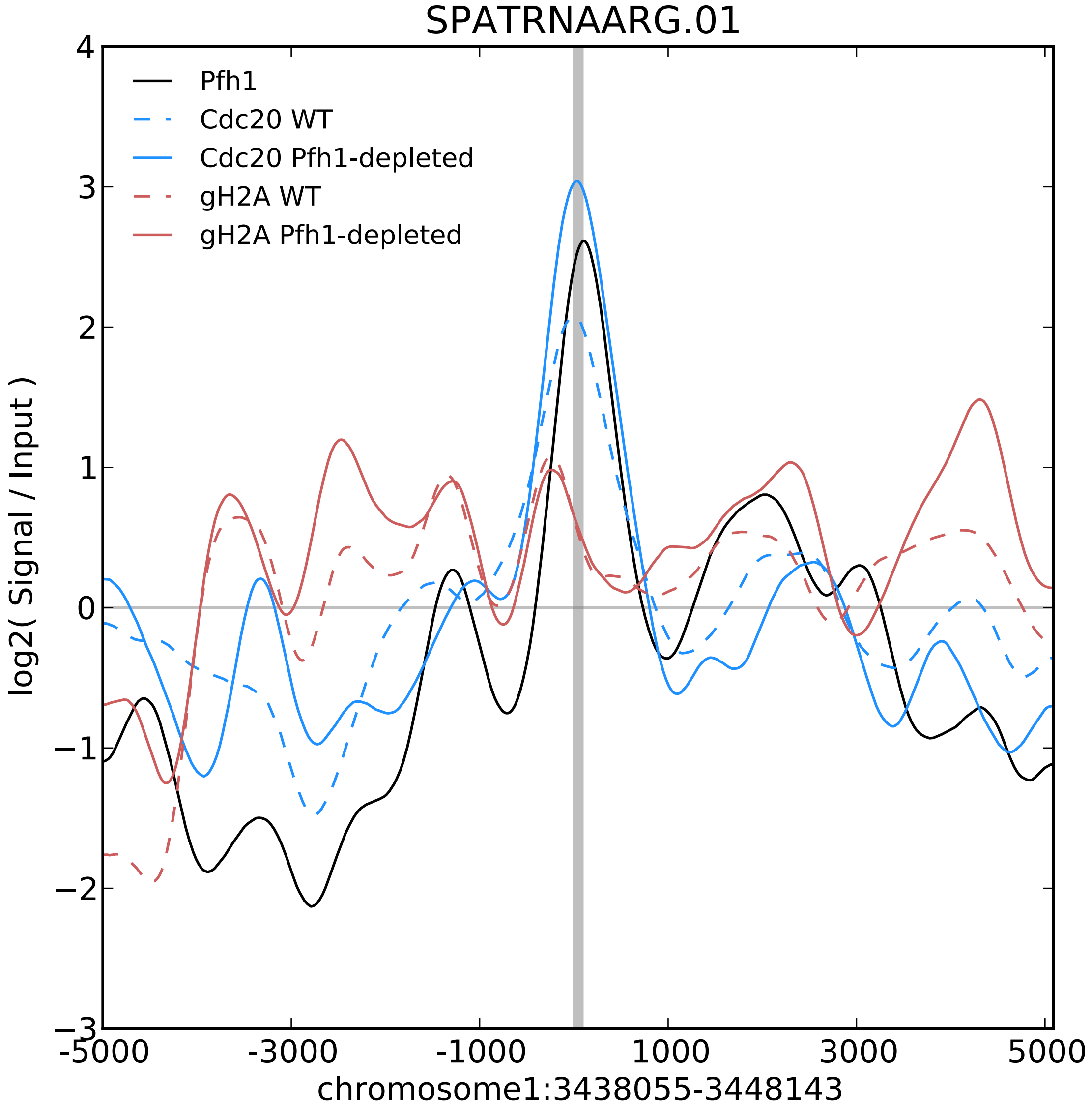

SPATRNAARG.01
Pfh1
Cdc20 WT
Cdc20 Pfh1-depleted
gH2A WT
gH2A Pfh1-depleted
log2(Signal / Input)
4
3
2
1
0
−1
−2
−3
-5000
-3000
-1000
1000
3000
5000
chromosome1:3438055-3448143

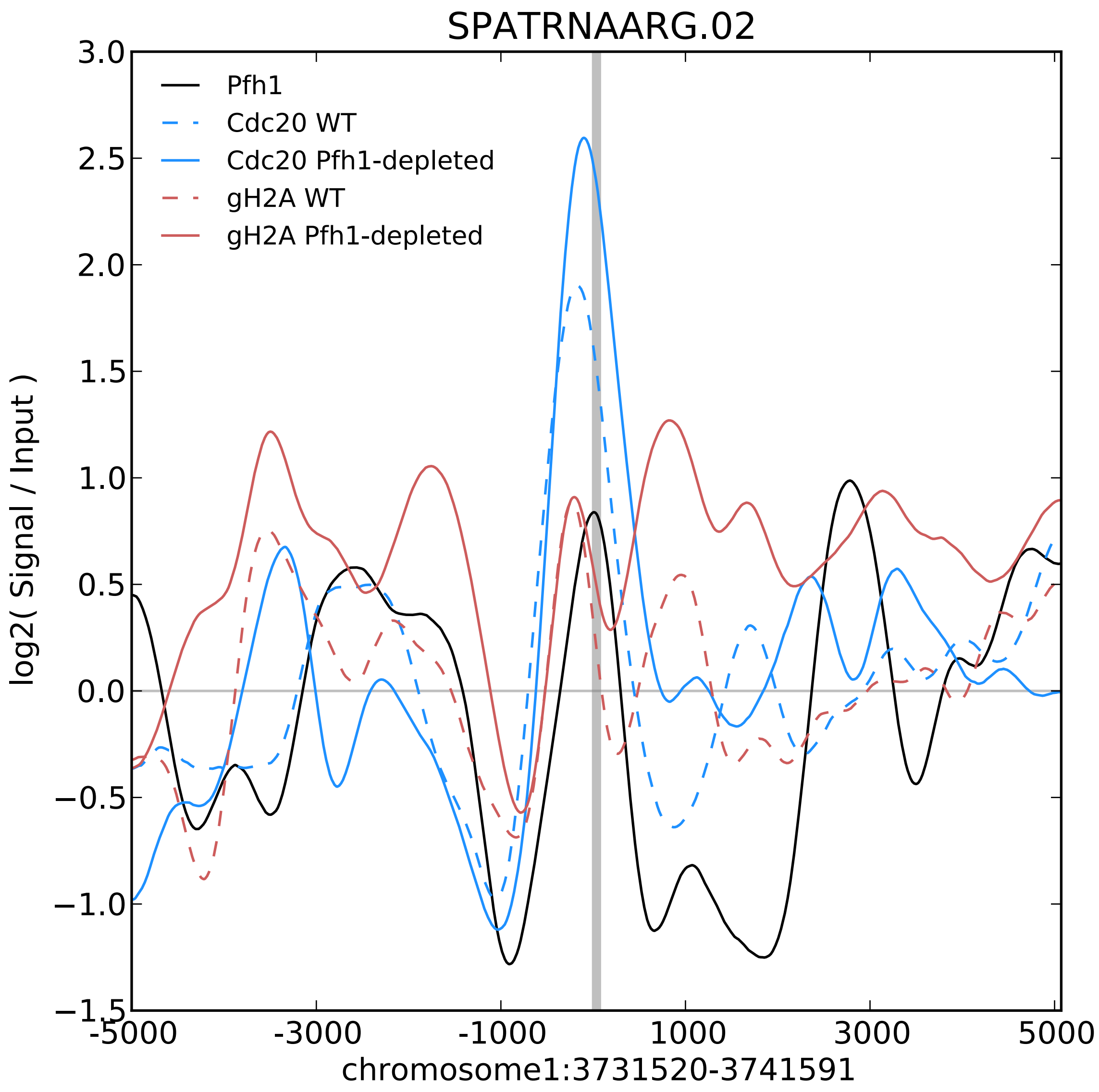

SPATRNAARG.02
Pfh1
Cdc20 WT
Cdc20 Pfh1-depleted
gH2A WT
gH2A Pfh1-depleted
log2(Signal / Input)
3.0
2.5
2.0
1.5
1.0
0.5
0.0
−0.5
−1.0
−1.5
-5000
-3000
-1000
1000
3000
5000
chromosome1:3731520-3741591

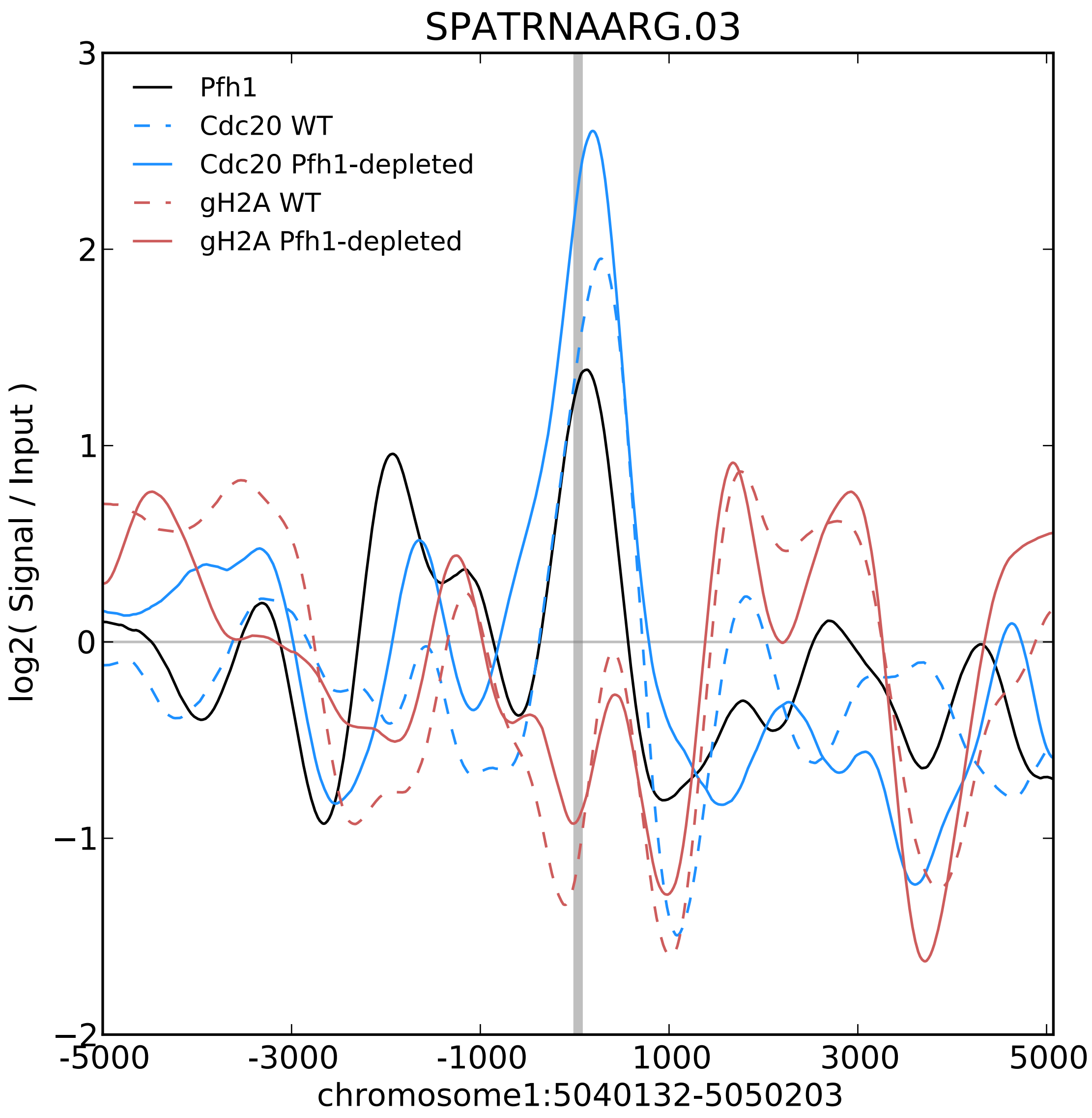

SPATRNAARG.03
Pfh1
Cdc20 WT
Cdc20 Pfh1-depleted
gH2A WT
gH2A Pfh1-depleted
log2(Signal / Input)
3
2
1
0
−1
−2
-5000
-3000
-1000
1000
3000
5000
chromosome1:5040132-5050203

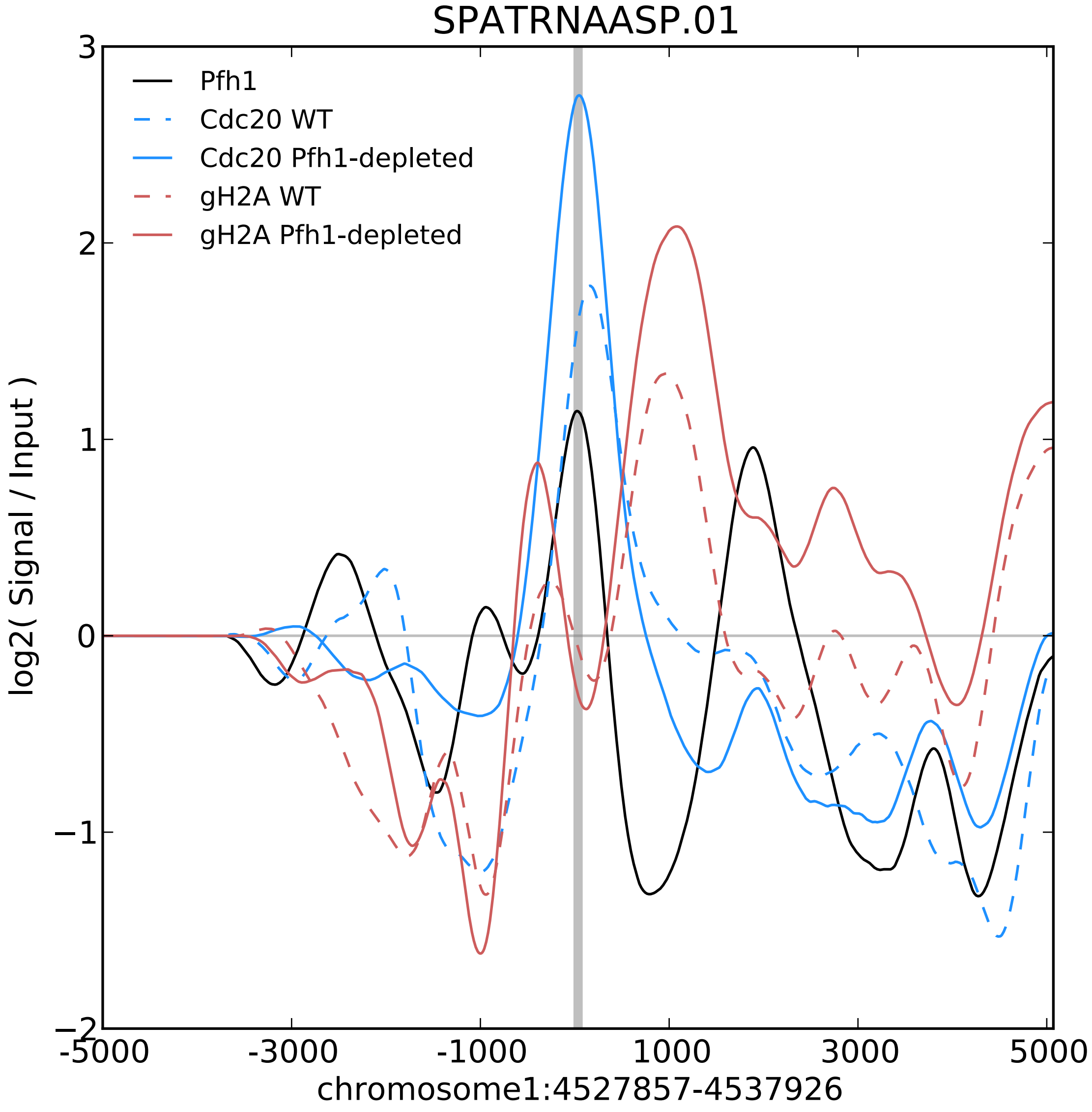
SPATRNAASP.01
Pfh1
Cdc20 WT
Cdc20 Pfh1-depleted
gH2A WT
gH2A Pfh1-depleted
log2(Signal / Input)
3
2
1
0
−1
−2
-5000
-3000
-1000
1000
3000
5000
chromosome1:4527857-4537926

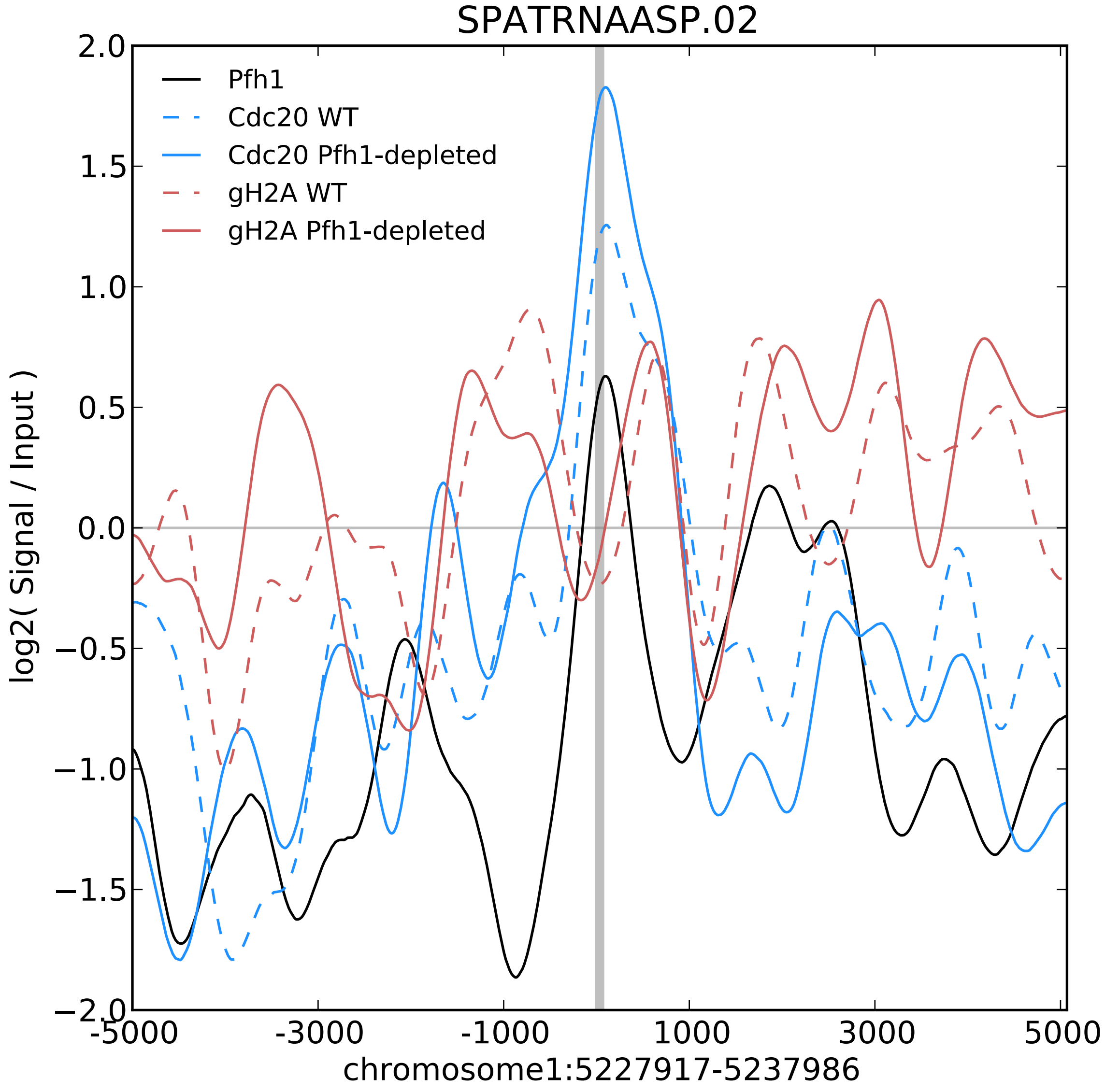
SPATRNAASP.02
Pfh1
Cdc20 WT
Cdc20 Pfh1-depleted
gH2A WT
gH2A Pfh1-depleted
log2(Signal / Input)
2.0
1.5
1.0
0.5
0.0
−0.5
−1.0
−1.5
−2.0
-5000
-3000
-1000
1000
3000
5000
chromosome1:5227917-5237986

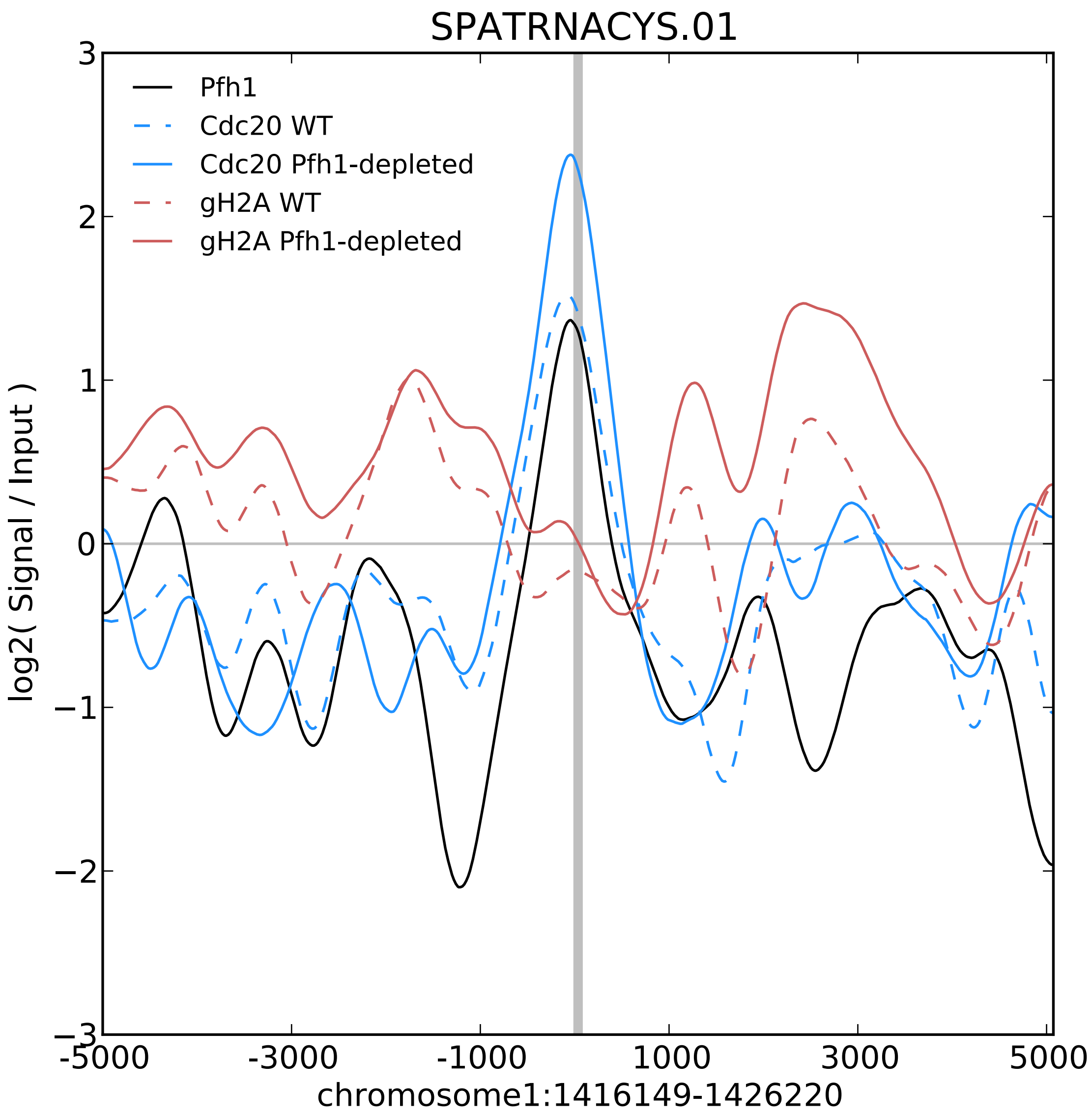
SPATRNACYS.01
Pfh1
Cdc20 WT
Cdc20 Pfh1-depleted
gH2A WT
gH2A Pfh1-depleted
log2(Signal / Input)
3
2
1
0
−1
−2
−3
-5000
-3000
-1000
1000
3000
5000
chromosome1:1416149-1426220

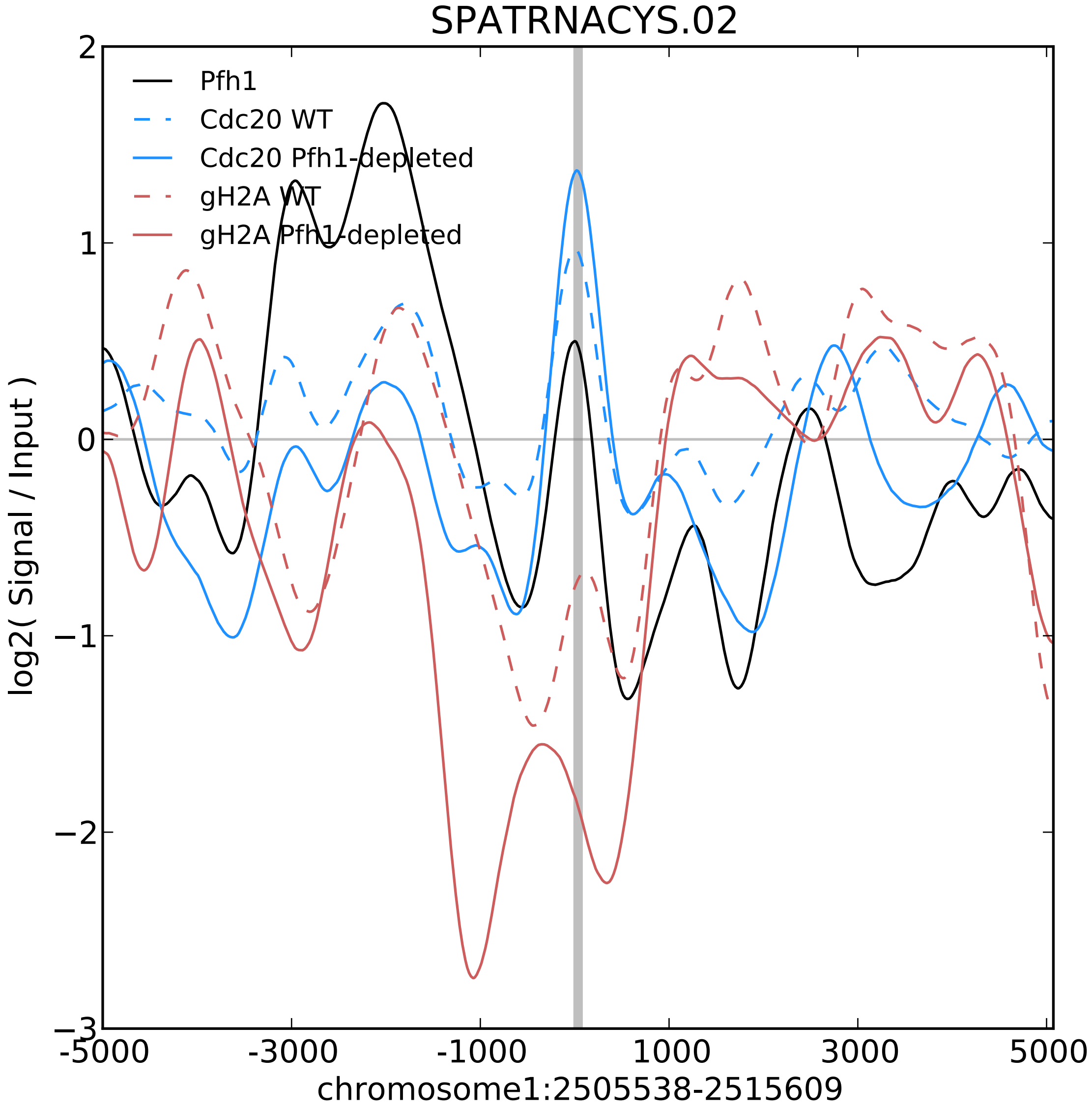
SPATRNACYS.02
Pfh1
Cdc20 WT
Cdc20 Pfh1-depleted
gH2A WT
gH2A Pfh1-depleted
2
1
0
−1
−2
−3
log2(Signal / Input)
-5000
-3000
-1000
1000
3000
5000
chromosome1:2505538-2515609

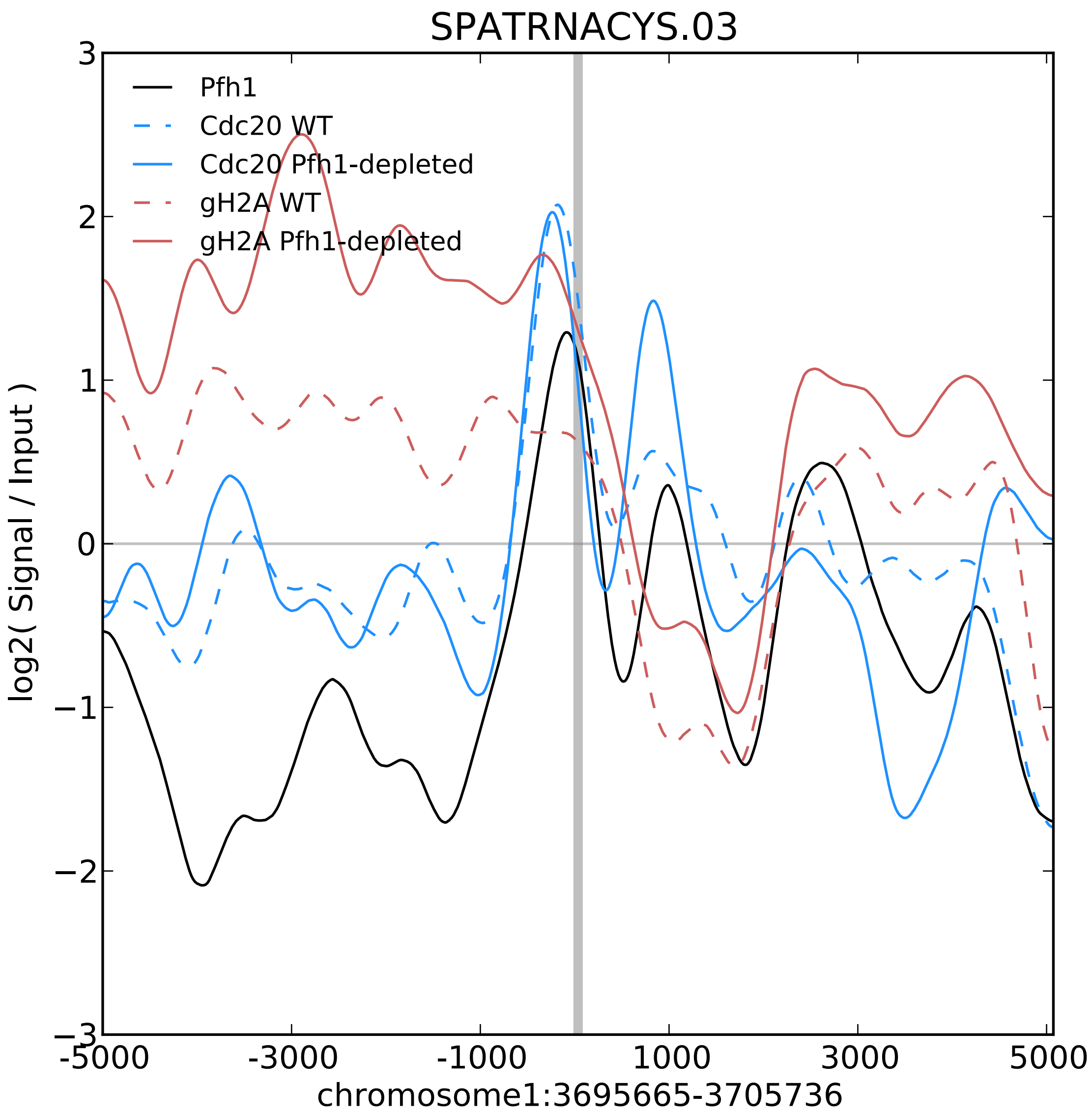
SPATRNACYS.03
Pfh1
Cdc20 WT
Cdc20 Pfh1-depleted
gH2A WT
gH2A Pfh1-depleted
log2(Signal / Input)
3
2
1
0
−1
−2
−3
-5000
-3000
-1000
1000
3000
5000
chromosome1:3695665-3705736

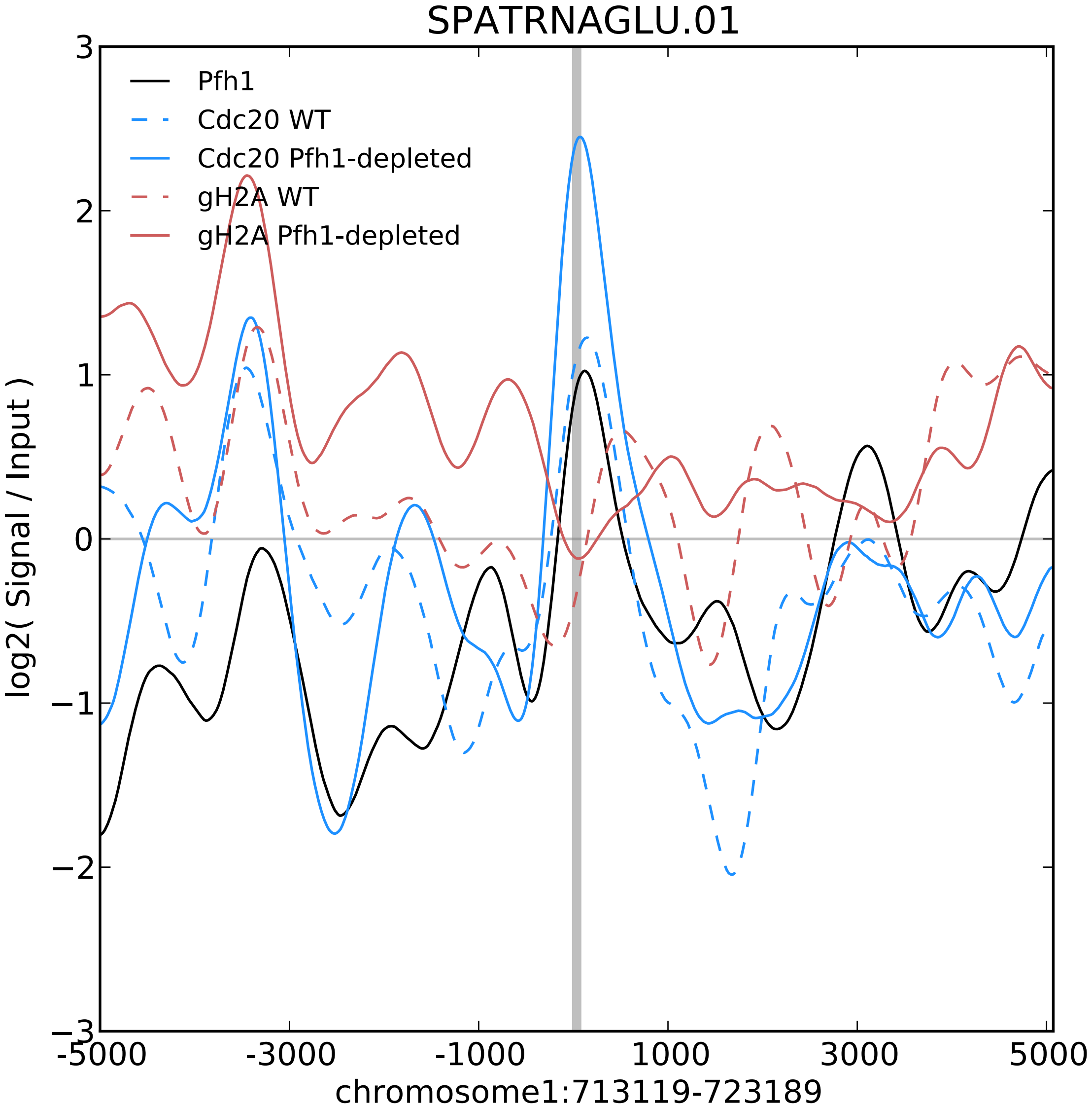
SPATRNAGLU.01
Pfh1
Cdc20 WT
Cdc20 Pfh1-depleted
gH2A WT
gH2A Pfh1-depleted
log2(Signal / Input)
3
2
1
0
−1
−2
−3
-5000
-3000
-1000
1000
3000
5000
chromosome1:713119-723189

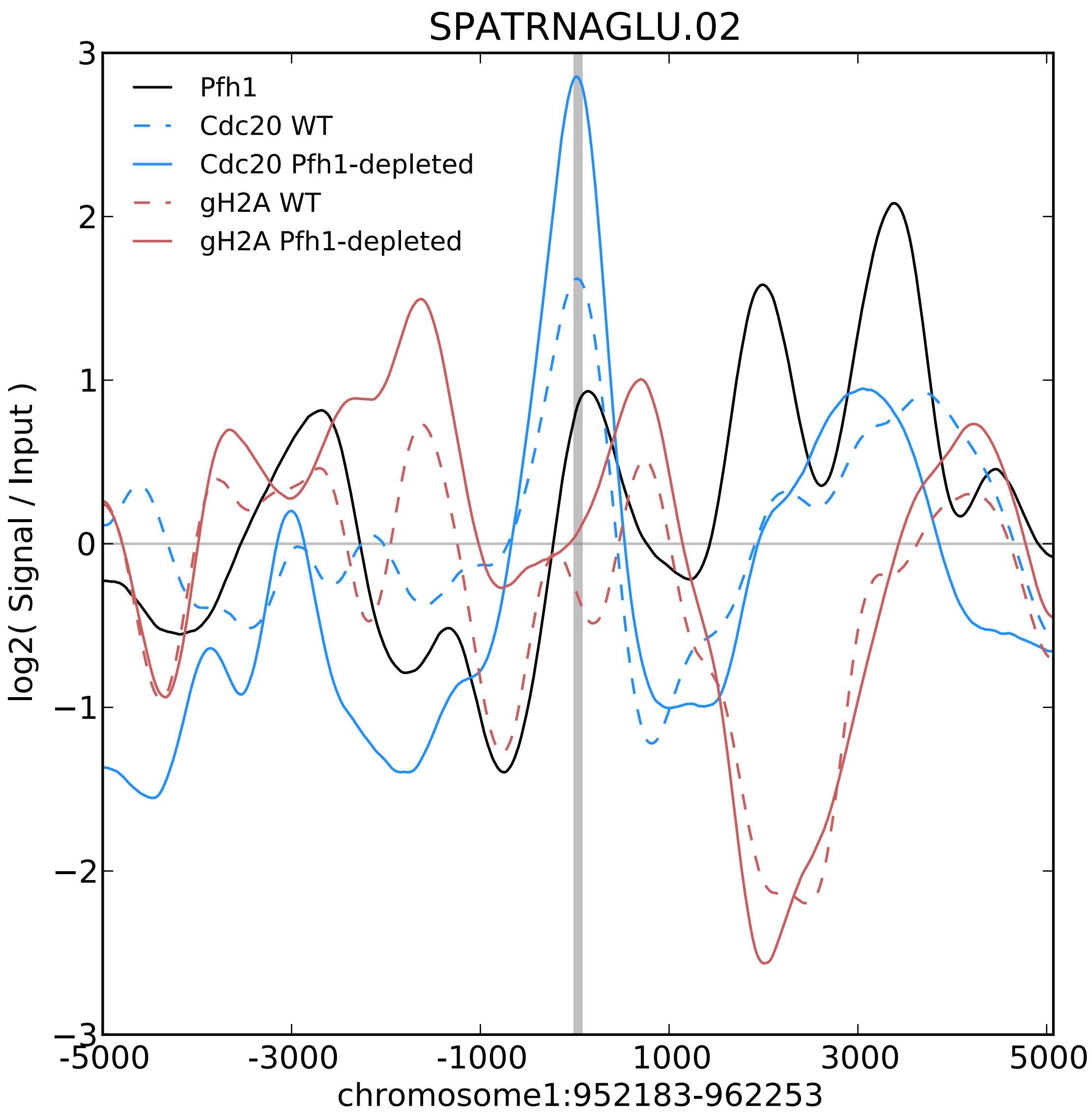
SPATRNAGLU.02
Pfh1
Cdc20 WT
Cdc20 Pfh1-depleted
gH2A WT
gH2A Pfh1-depleted
log2(Signal / Input)
3
2
1
0
−1
−2
−3
-5000
-3000
-1000
1000
3000
5000
chromosome1:952183-962253

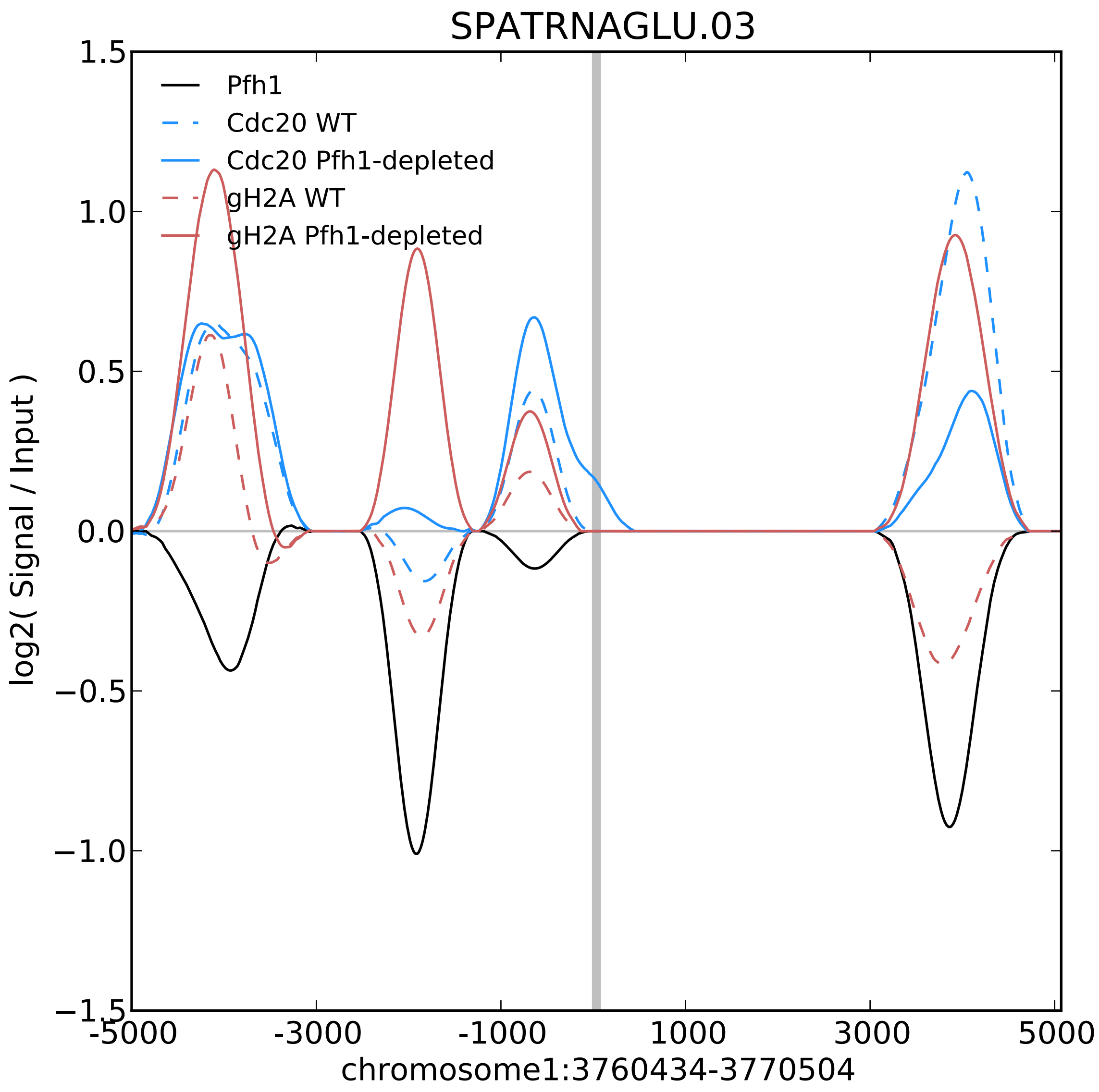

SPATRNAGLU.03
Pfh1
Cdc20 WT
Cdc20 Pfh1-depleted
gH2A WT
gH2A Pfh1-depleted
log2(Signal / Input)
1.5
1.0
0.5
0.0
−0.5
−1.0
−1.5
-5000
-3000
-1000
1000
3000
5000
chromosome1:3760434-3770504

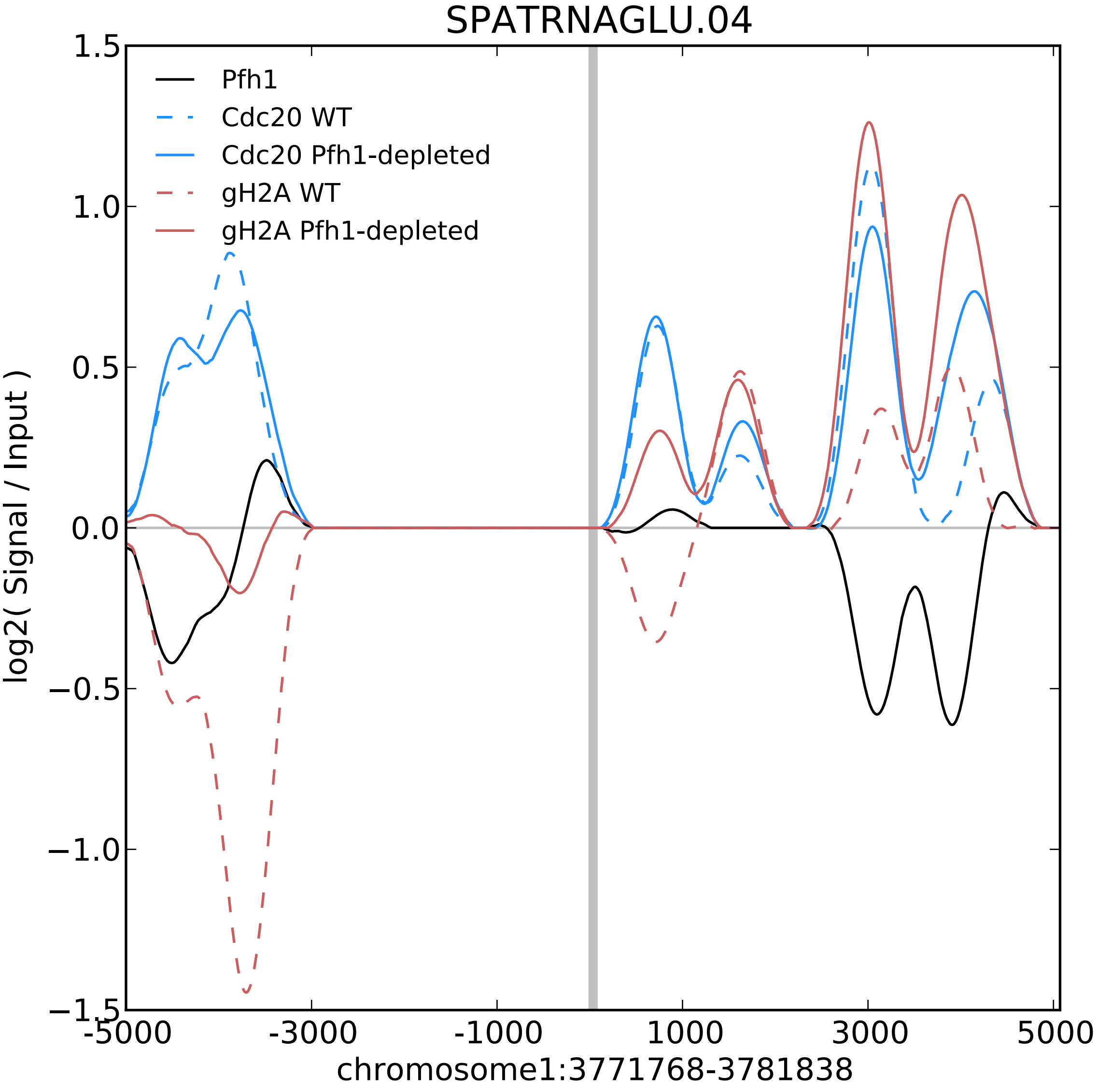

SPATRNAGLU.04
Pfh1
Cdc20 WT
Cdc20 Pfh1-depleted
gH2A WT
gH2A Pfh1-depleted
1.5
1.0
0.5
0.0
−0.5
−1.0
−1.5
log2(Signal / Input)
-5000
-3000
-1000
1000
3000
5000
chromosome1:3771768-3781838

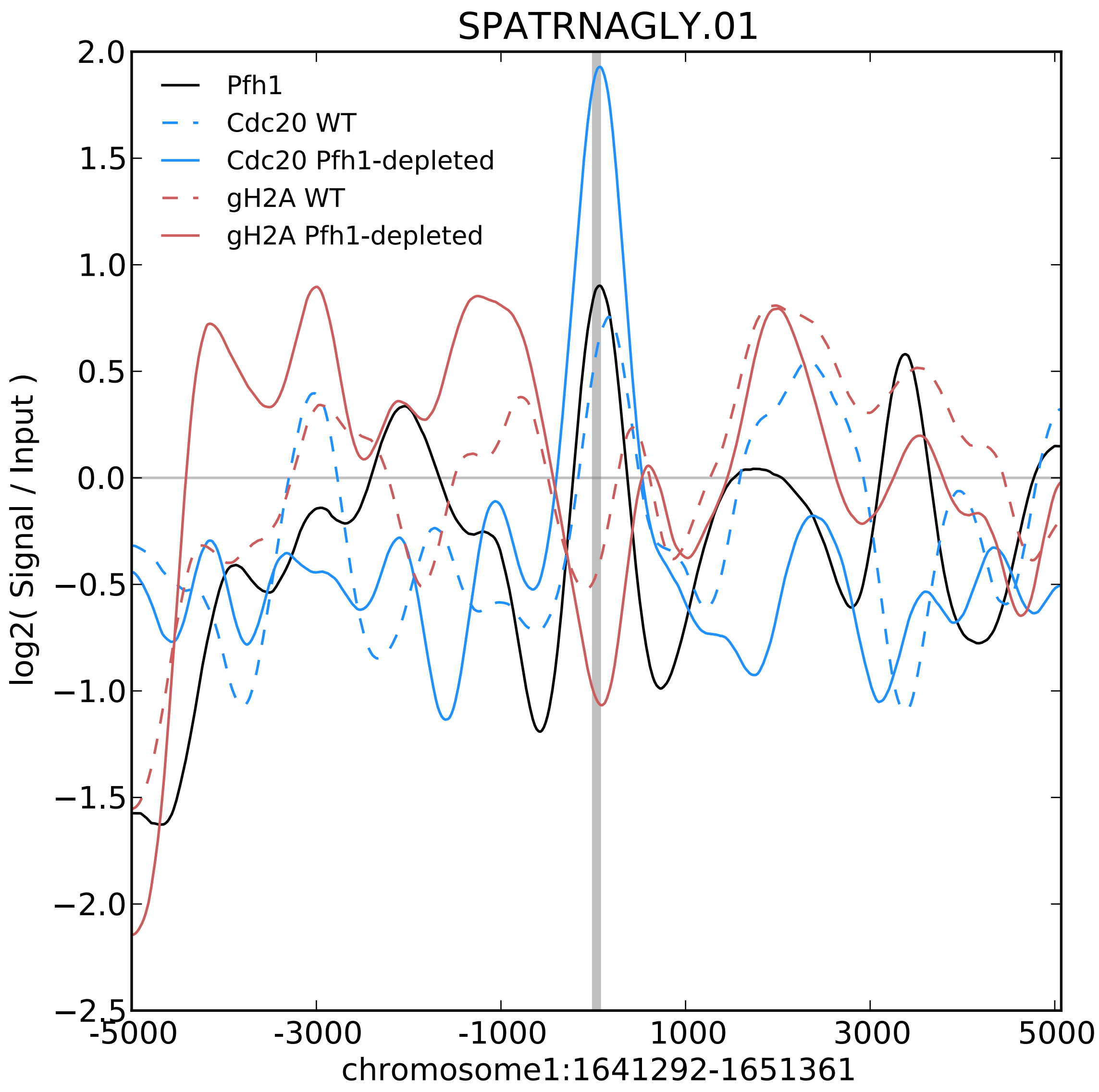

SPATRNAGLY.01
Pfh1
Cdc20 WT
Cdc20 Pfh1-depleted
gH2A WT
gH2A Pfh1-depleted
2.0
1.5
1.0
0.5
0.0
−0.5
−1.0
−1.5
−2.0
−2.5
log2(Signal / Input)
-5000
-3000
-1000
1000
3000
5000
chromosome1:1641292-1651361

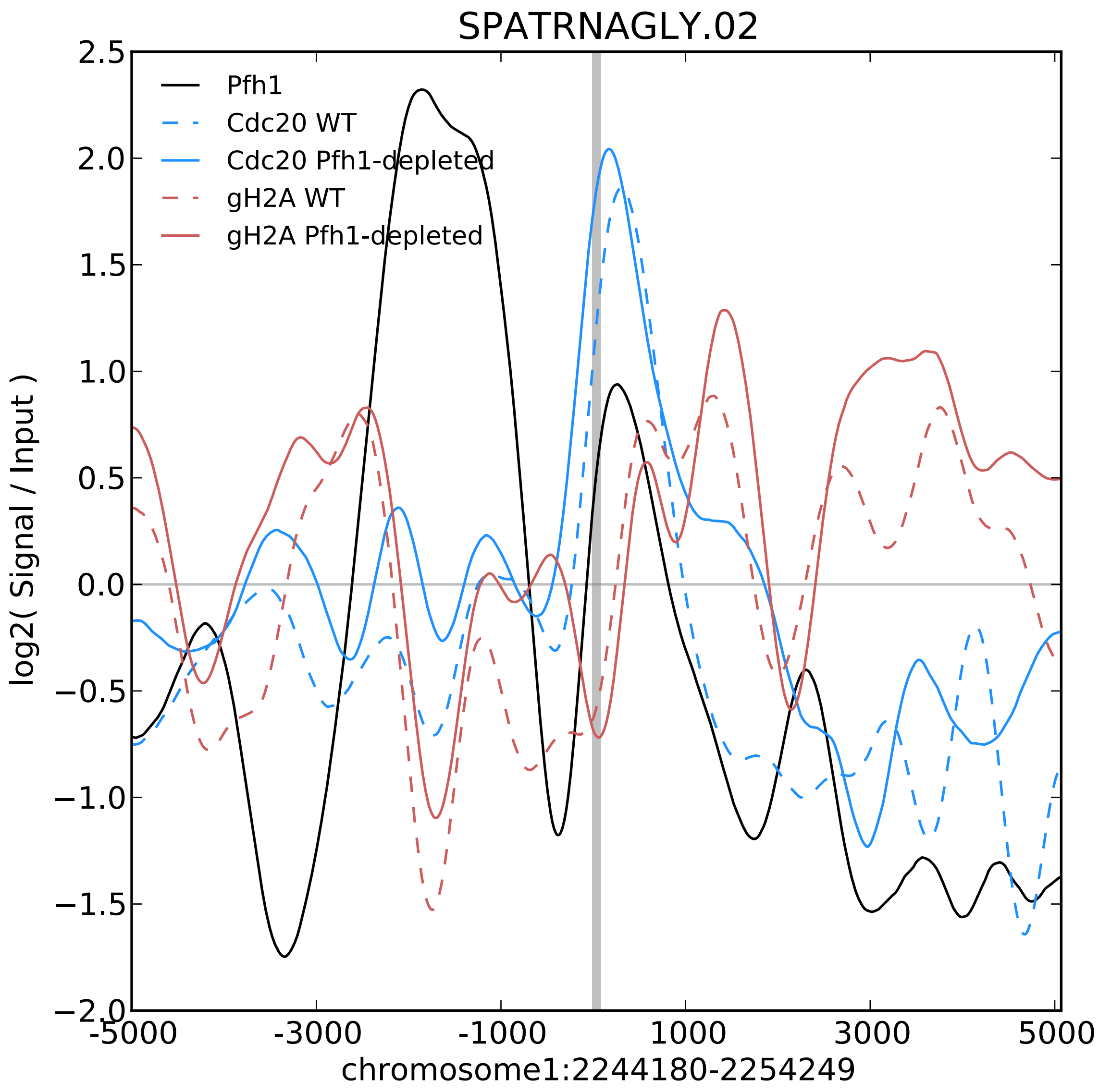

SPATRNAGLY.02
Pfh1
Cdc20 WT
Cdc20 Pfh1-depleted
gH2A WT
gH2A Pfh1-depleted
log2(Signal / Input)
2.5
2.0
1.5
1.0
0.5
0.0
−0.5
−1.0
−1.5
−2.0
-5000
-3000
-1000
1000
3000
5000
chromosome1:2244180-2254249

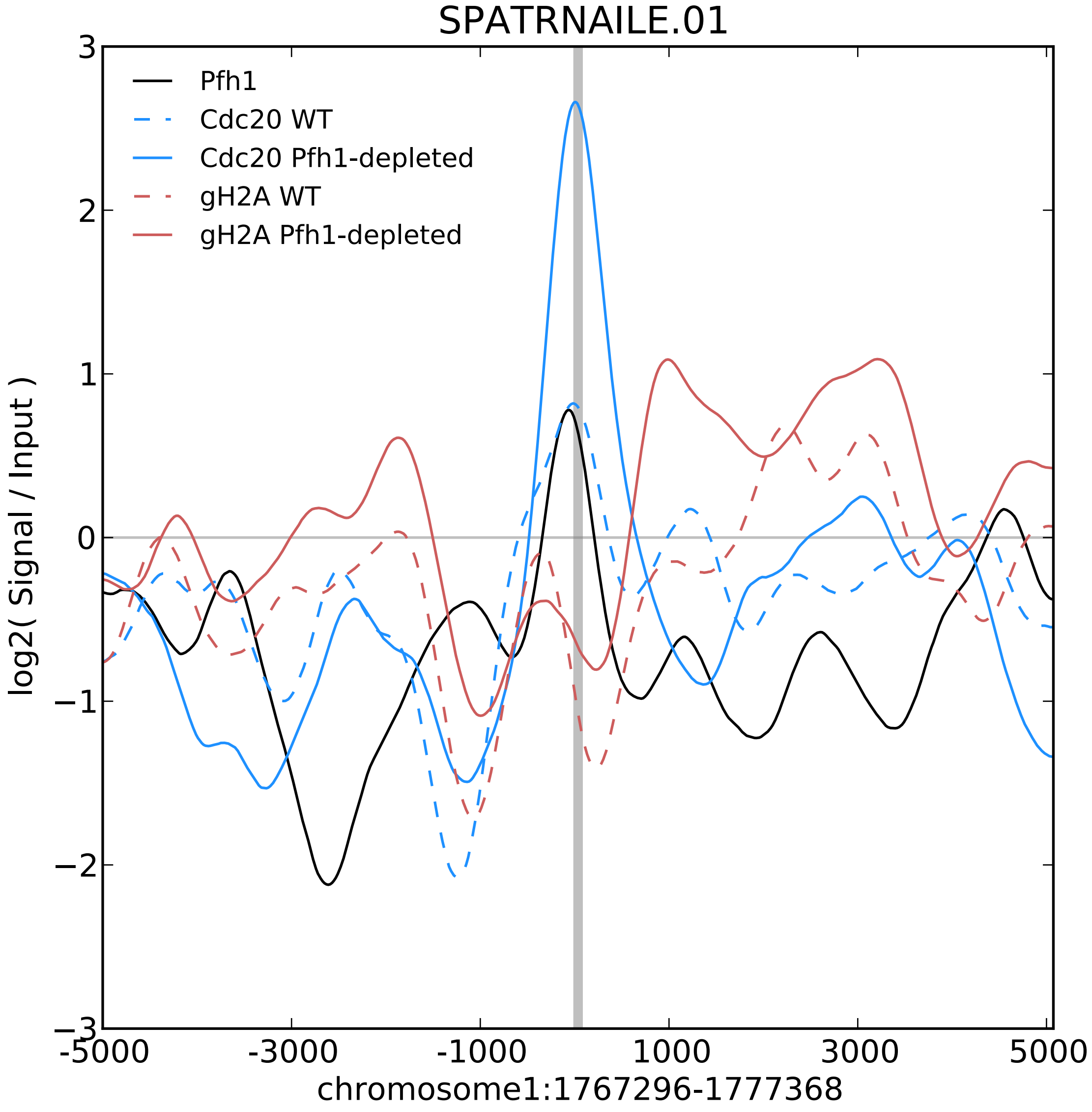

SPATRNAILE.01
Pfh1
Cdc20 WT
Cdc20 Pfh1-depleted
gH2A WT
gH2A Pfh1-depleted
log2(Signal / Input)
3
2
1
0
−1
−2
−3
-5000
-3000
-1000
1000
3000
5000
chromosome1:1767296-1777368

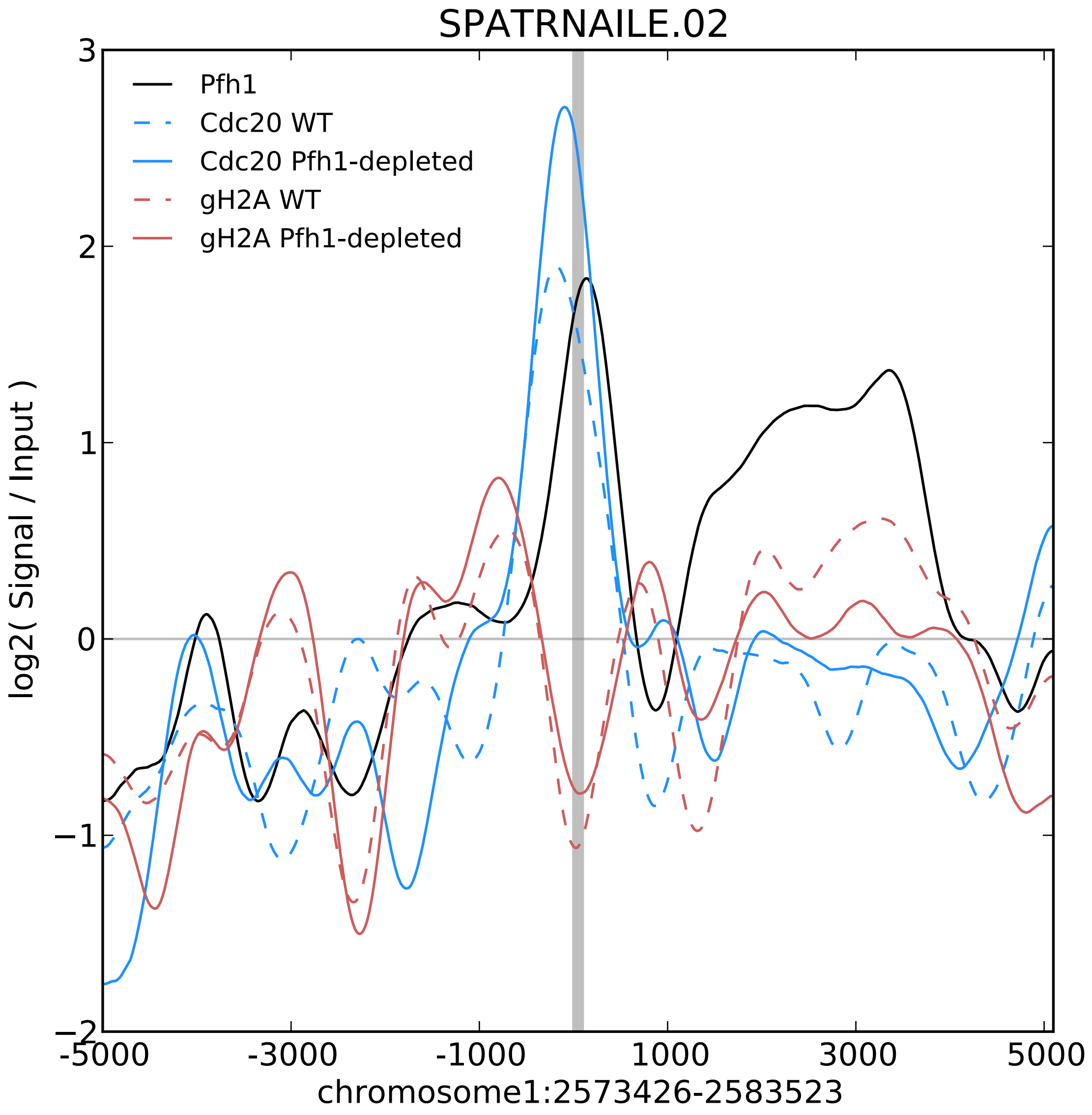
SPATRNAILE.02
Pfh1
Cdc20 WT
Cdc20 Pfh1-depleted
gH2A WT
gH2A Pfh1-depleted
log2(Signal / Input)
3
2
1
0
−1
−2
-5000
-3000
-1000
1000
3000
5000
chromosome1:2573426-2583523

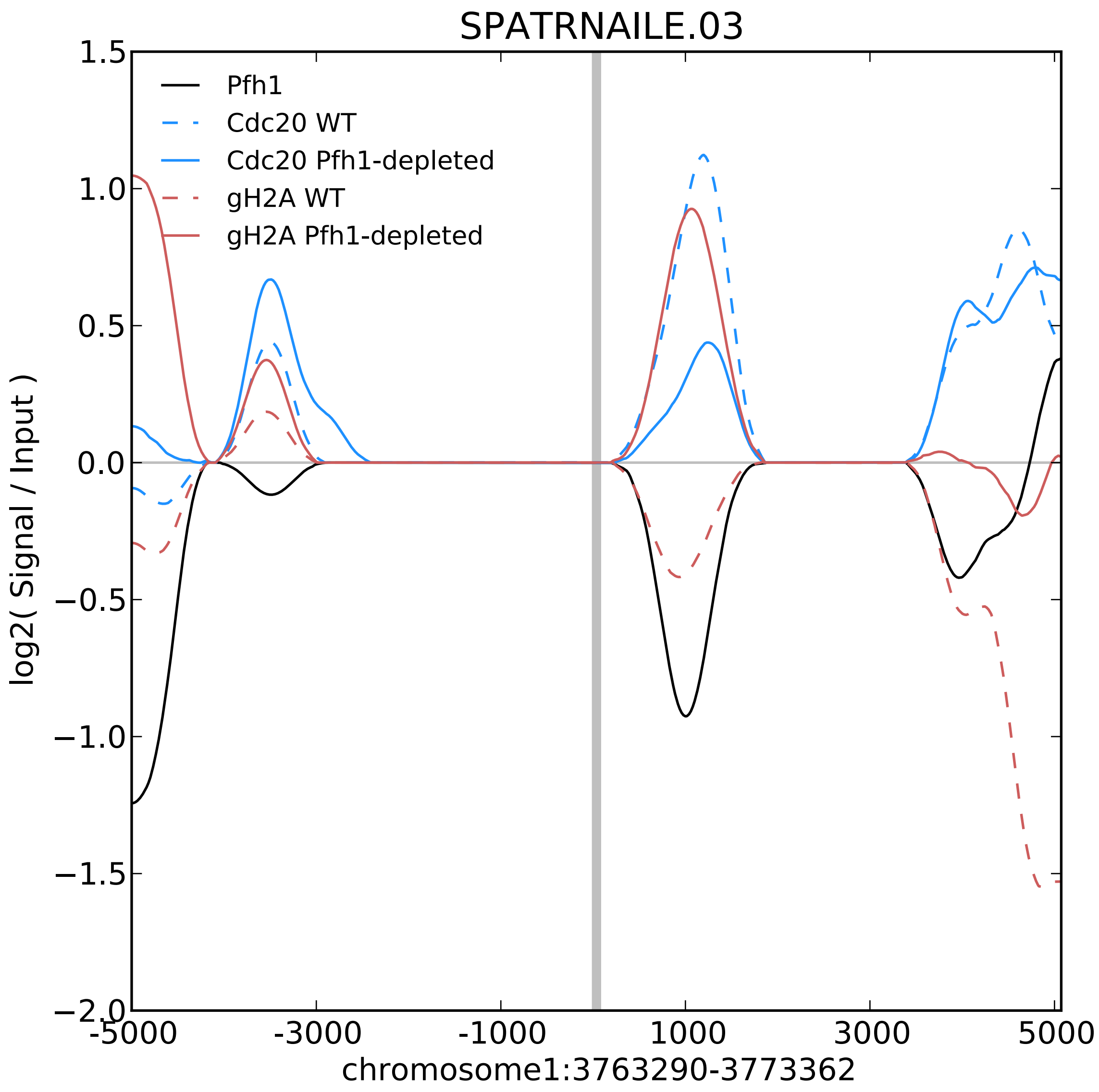

SPATRNAILE.03
Pfh1
Cdc20 WT
Cdc20 Pfh1-depleted
gH2A WT
gH2A Pfh1-depleted
1.5
1.0
0.5
0.0
−0.5
−1.0
−1.5
−2.0
log2(Signal / Input)
-5000
-3000
-1000
1000
3000
5000
chromosome1:3763290-3773362

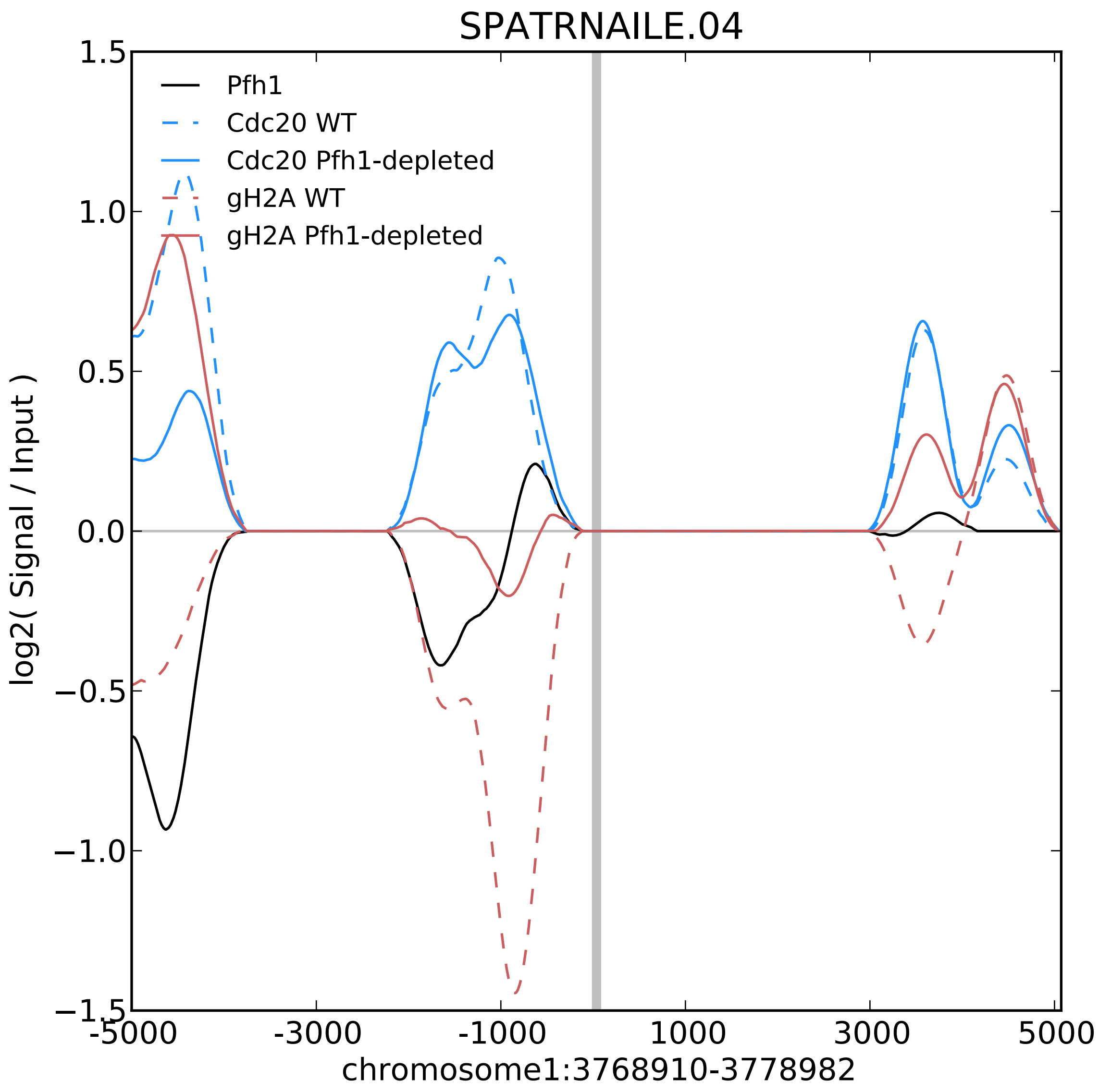
SPATRNAILE.04
Pfh1
Cdc20 WT
Cdc20 Pfh1-depleted
gH2A WT
gH2A Pfh1-depleted
log2(Signal / Input)
1.5
1.0
0.5
0.0
−0.5
−1.0
−1.5
-5000
-3000
-1000
1000
3000
5000
chromosome1:3768910-3778982

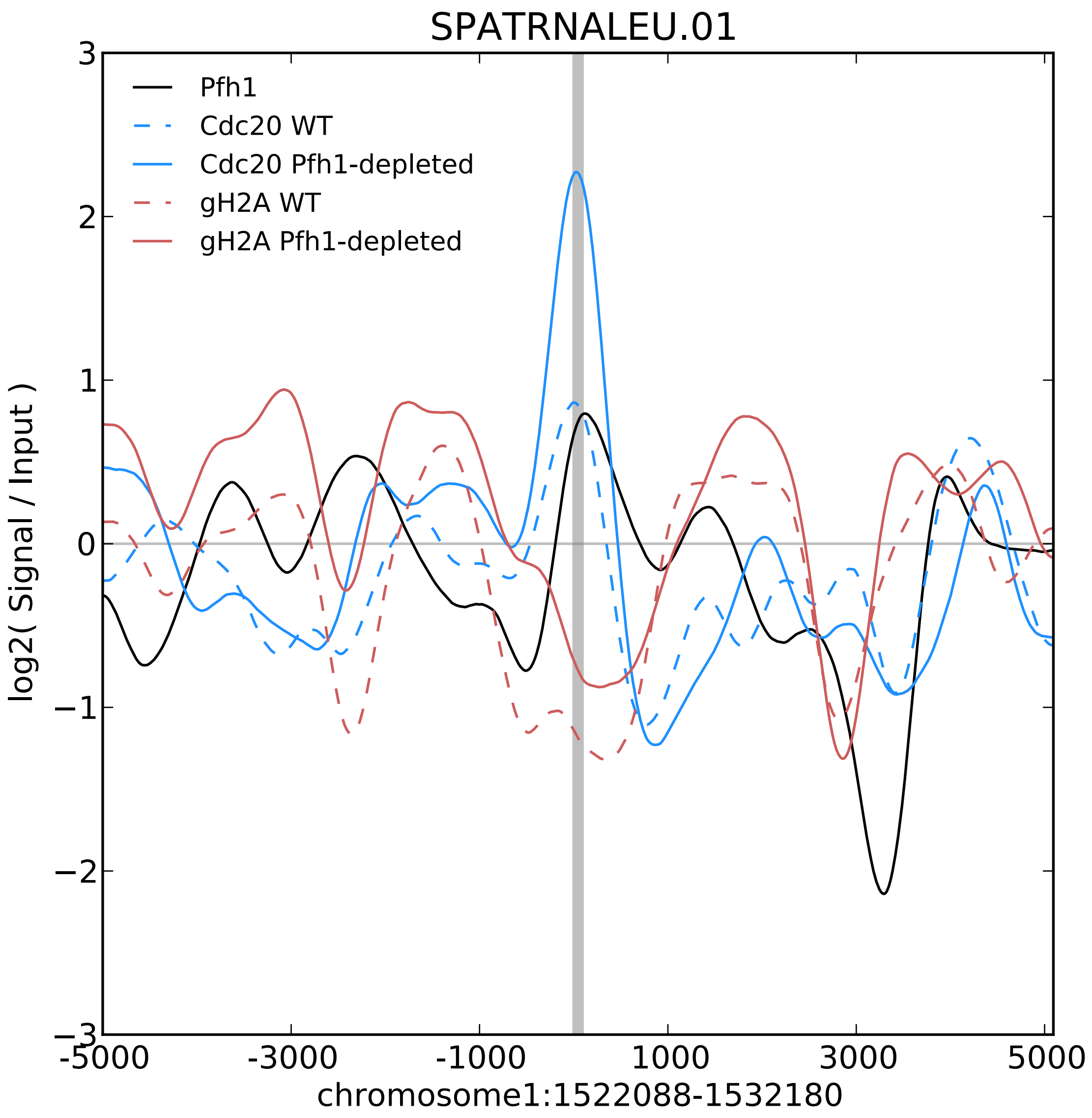

SPATRNALEU.01
Pfh1
Cdc20 WT
Cdc20 Pfh1-depleted
gH2A WT
gH2A Pfh1-depleted
log2(Signal / Input)
3
2
1
0
−1
−2
−3
-5000
-3000
-1000
1000
3000
5000
chromosome1:1522088-1532180

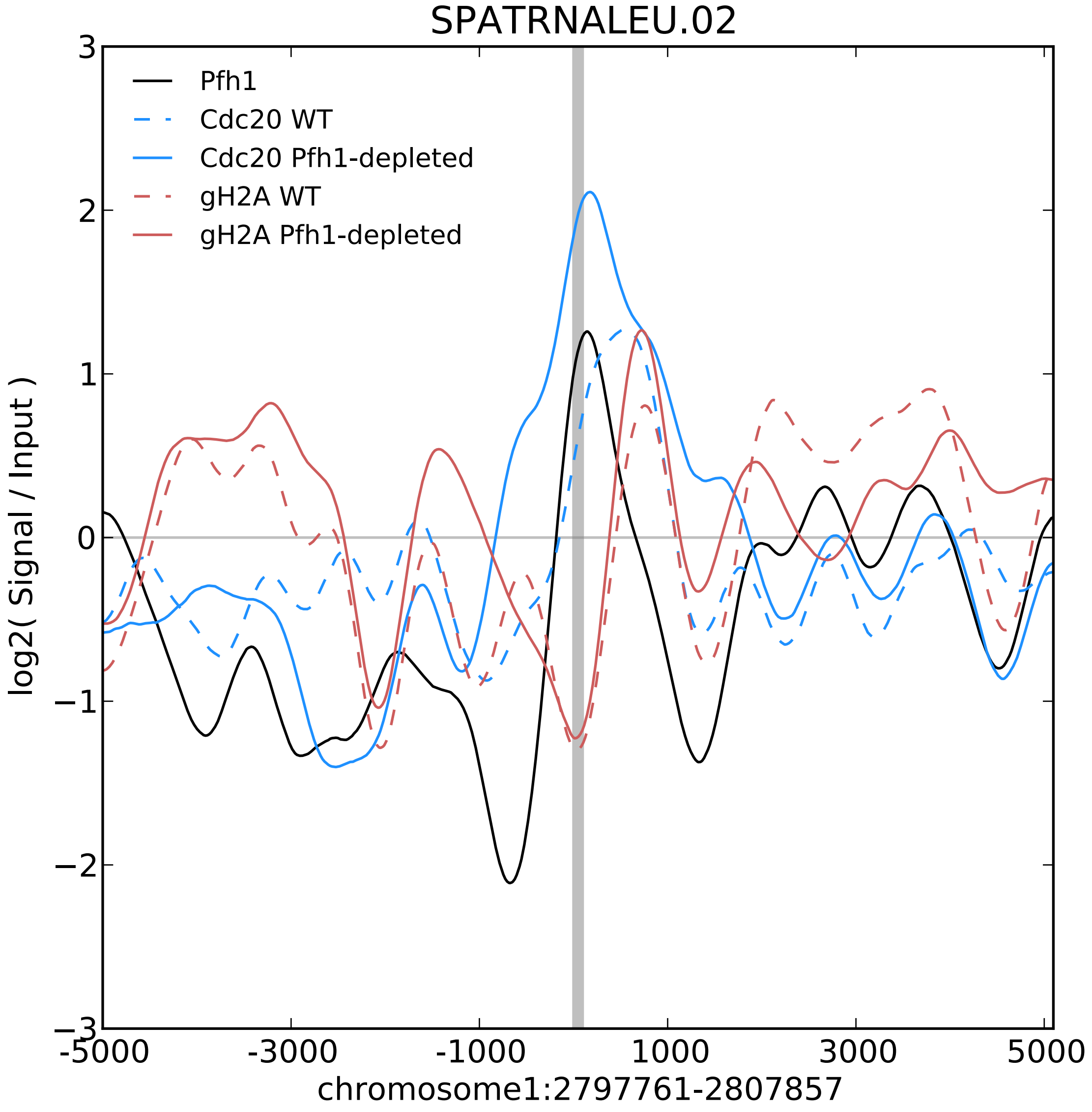
SPATRNALEU.02
Pfh1
Cdc20 WT
Cdc20 Pfh1-depleted
gH2A WT
gH2A Pfh1-depleted
log2(Signal / Input)
3
2
1
0
−1
−2
−3
-5000
-3000
-1000
1000
3000
5000
chromosome1:2797761-2807857

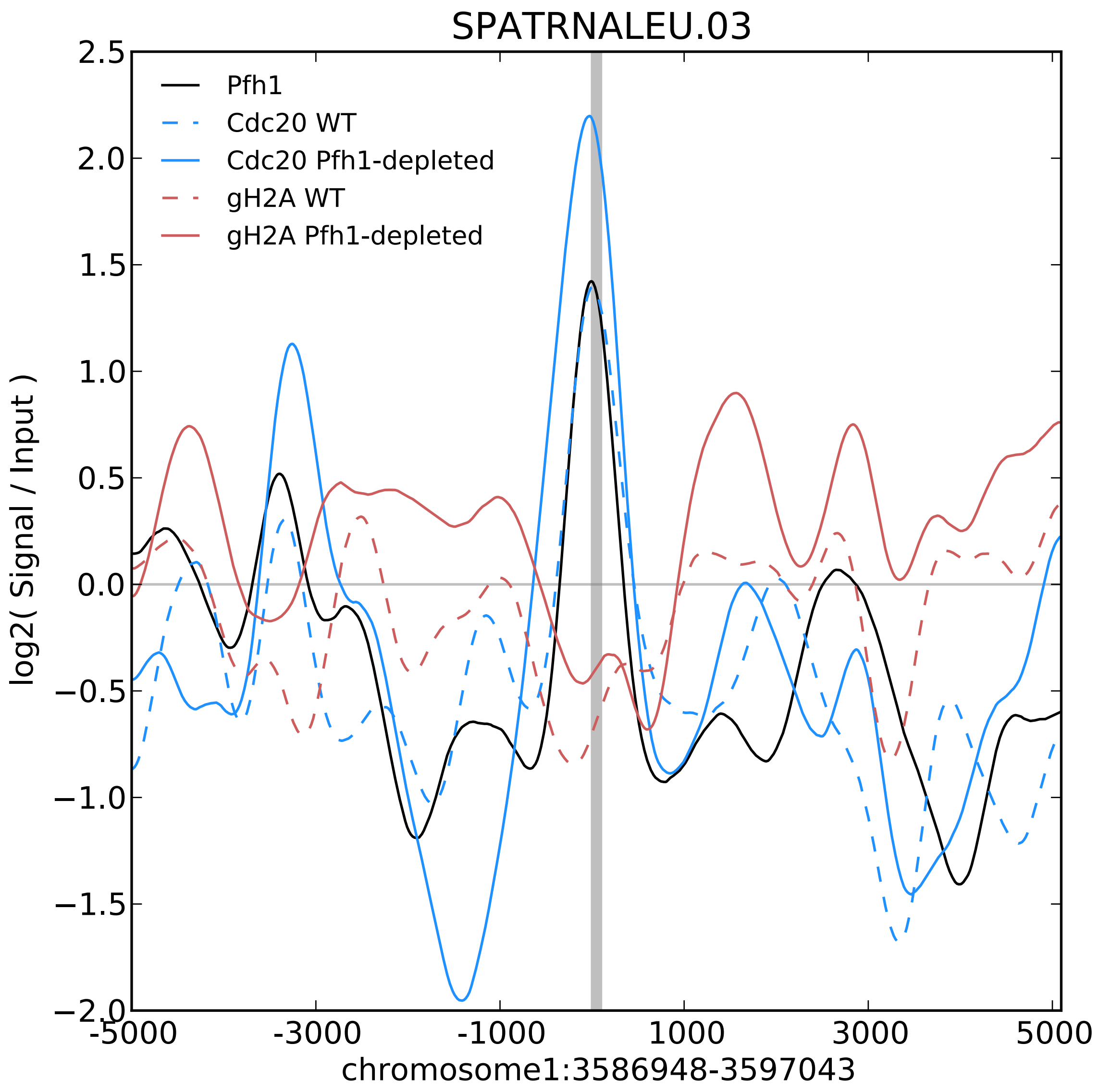
SPATRNALEU.03
Pfh1
Cdc20 WT
Cdc20 Pfh1-depleted
gH2A WT
gH2A Pfh1-depleted
log2(Signal / Input)
2.5
2.0
1.5
1.0
0.5
0.0
−0.5
−1.0
−1.5
−2.0
-5000
-3000
-1000
1000
3000
5000
chromosome1:3586948-3597043

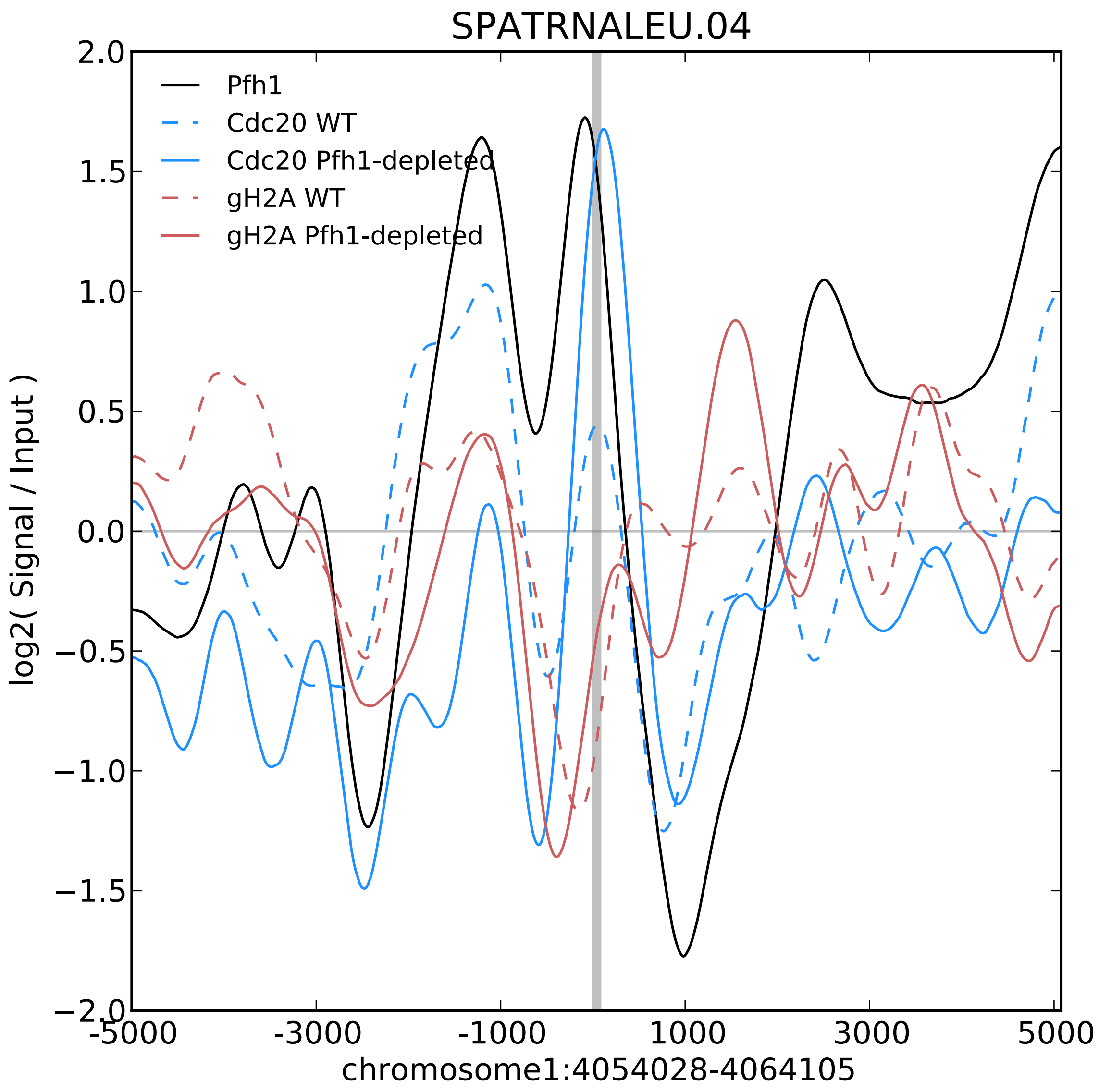

SPATRNALEU.04
Pfh1
Cdc20 WT
Cdc20 Pfh1-depleted
gH2A WT
gH2A Pfh1-depleted
log2(Signal / Input)
2.0
1.5
1.0
0.5
0.0
−0.5
−1.0
−1.5
−2.0
-5000
-3000
-1000
1000
3000
5000
chromosome1:4054028-4064105

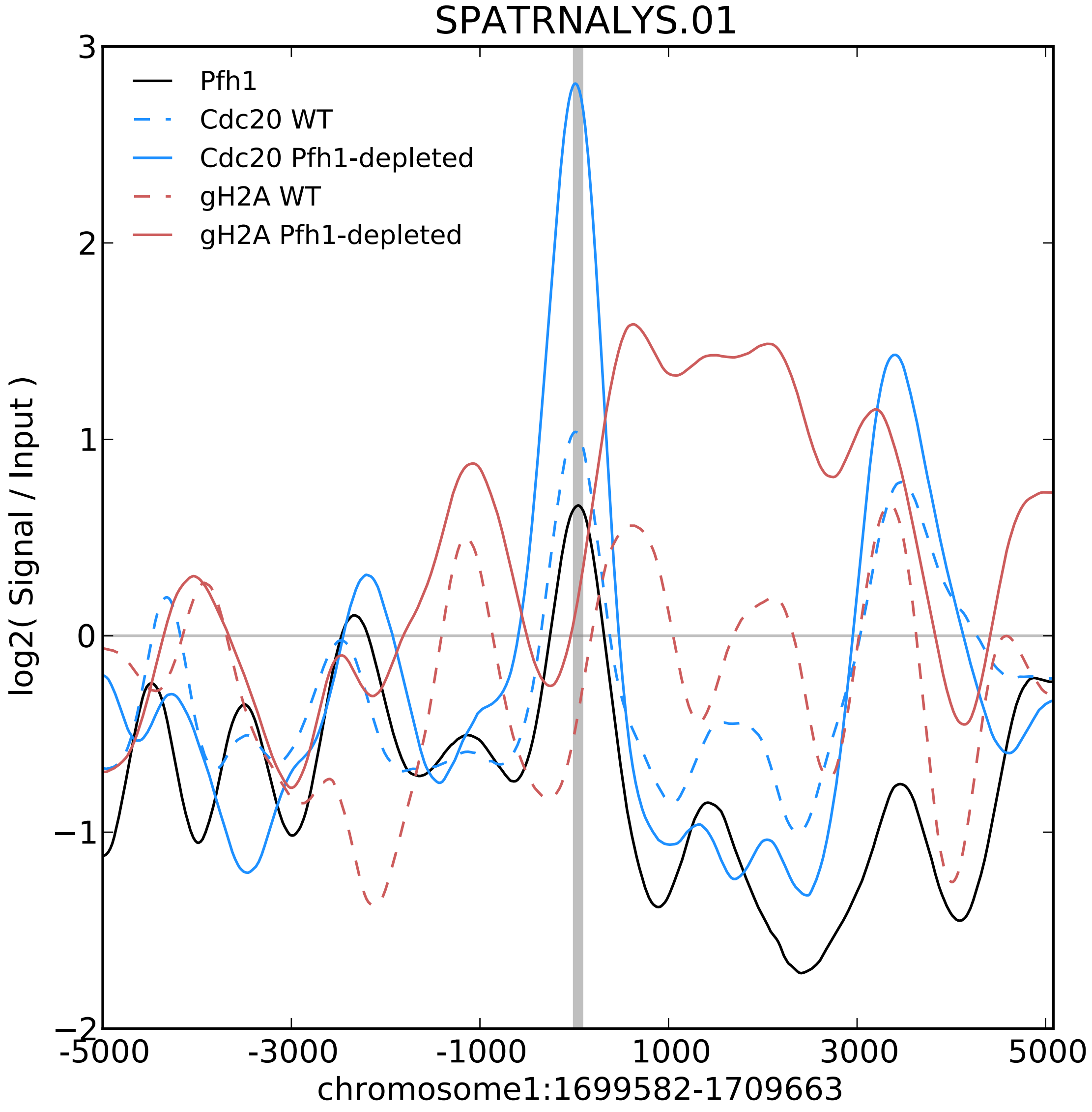
SPATRNALYS.01
Pfh1
Cdc20 WT
Cdc20 Pfh1-depleted
gH2A WT
gH2A Pfh1-depleted
log2(Signal / Input)
3
2
1
0
−1
−2
-5000
-3000
-1000
1000
3000
5000
chromosome1:1699582-1709663

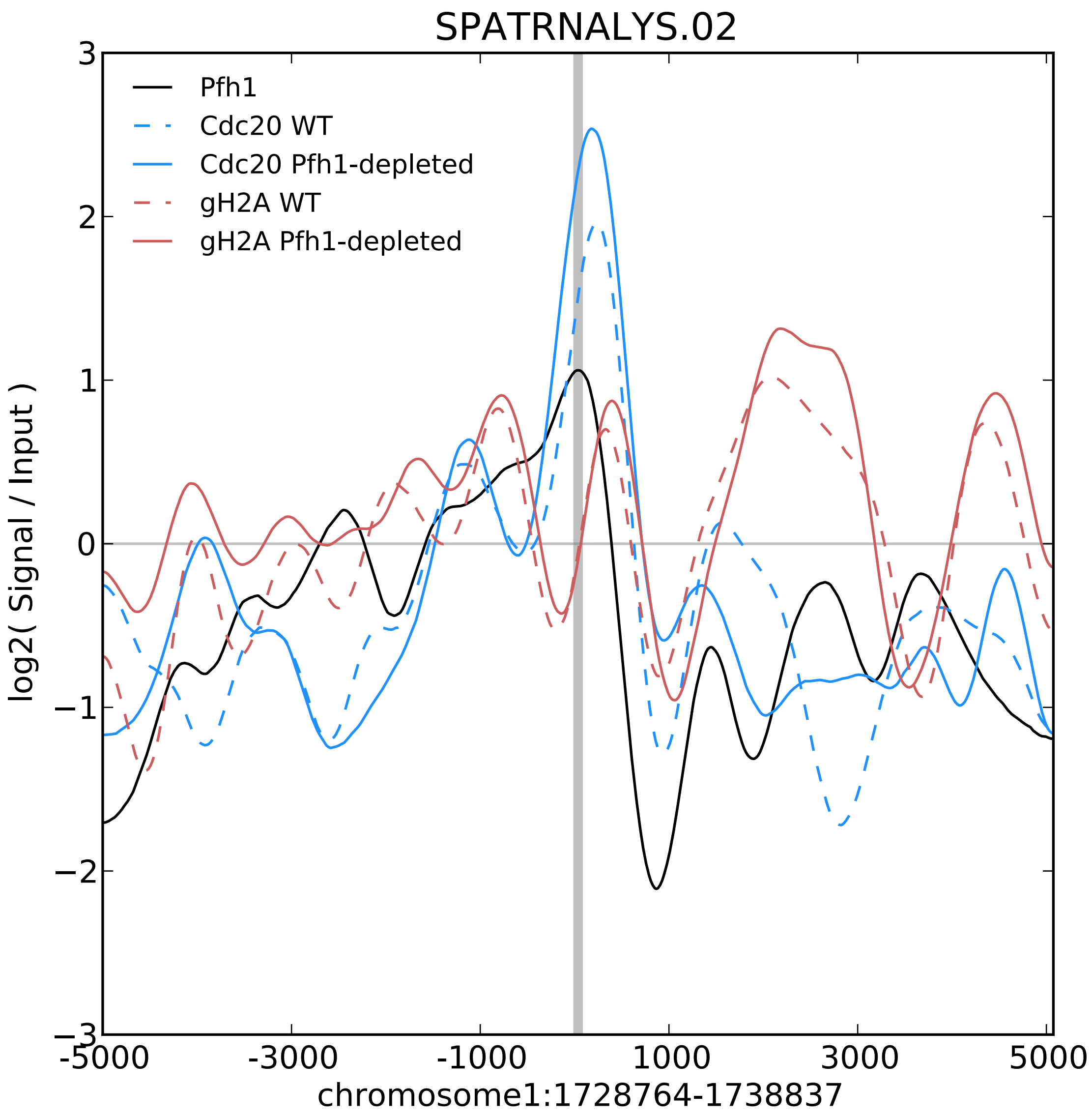
SPATRNALYS.02
Pfh1
Cdc20 WT
Cdc20 Pfh1-depleted
gH2A WT
gH2A Pfh1-depleted
log2(Signal / Input)
3
2
1
0
−1
−2
−3
-5000
-3000
-1000
1000
3000
5000
chromosome1:1728764-1738837

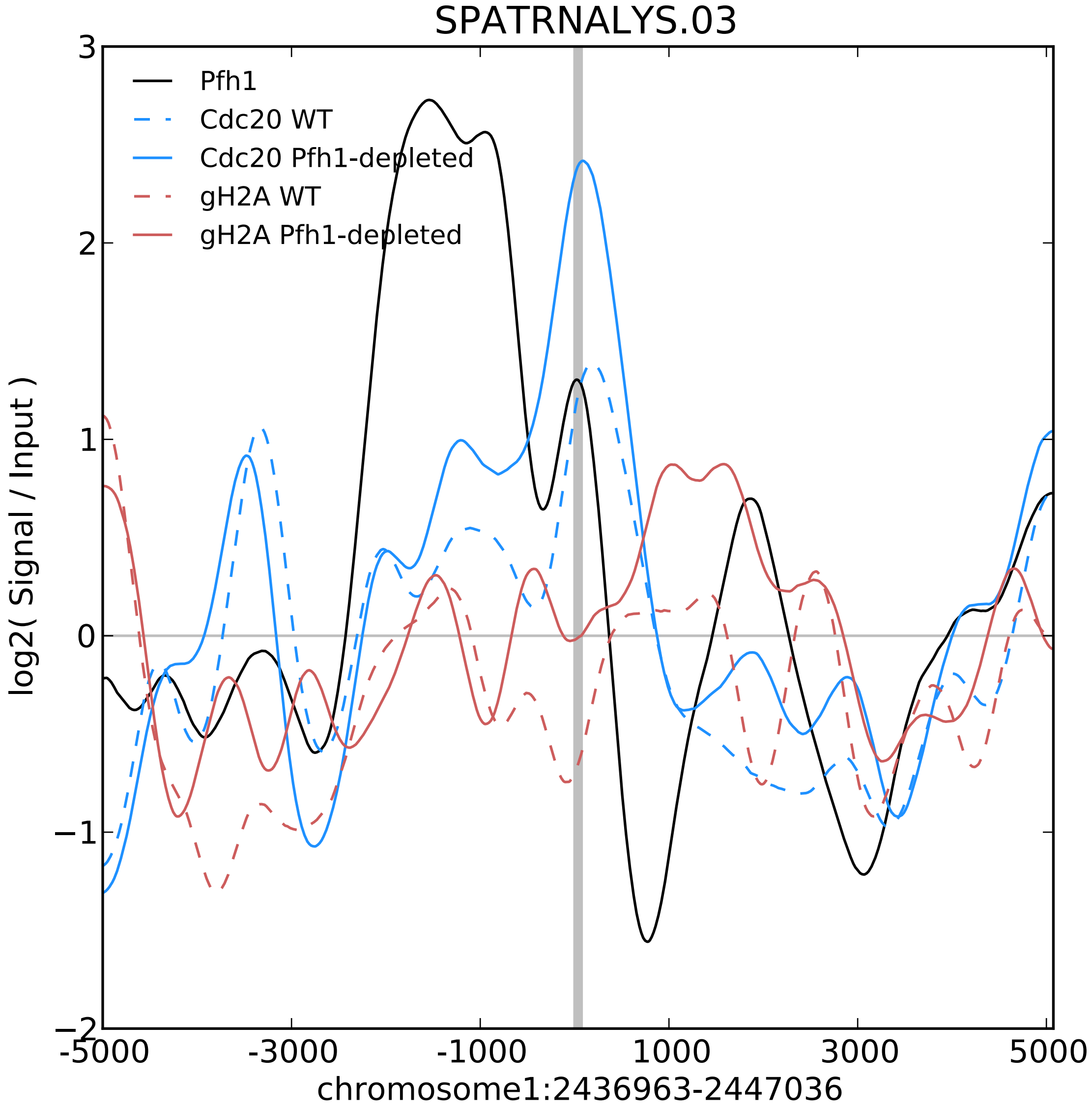
SPATRNALYS.03
Pfh1
Cdc20 WT
Cdc20 Pfh1-depleted
gH2A WT
gH2A Pfh1-depleted
log2(Signal / Input)
3
2
1
0
−1
−2
-5000
-3000
-1000
1000
3000
5000
chromosome1:2436963-2447036

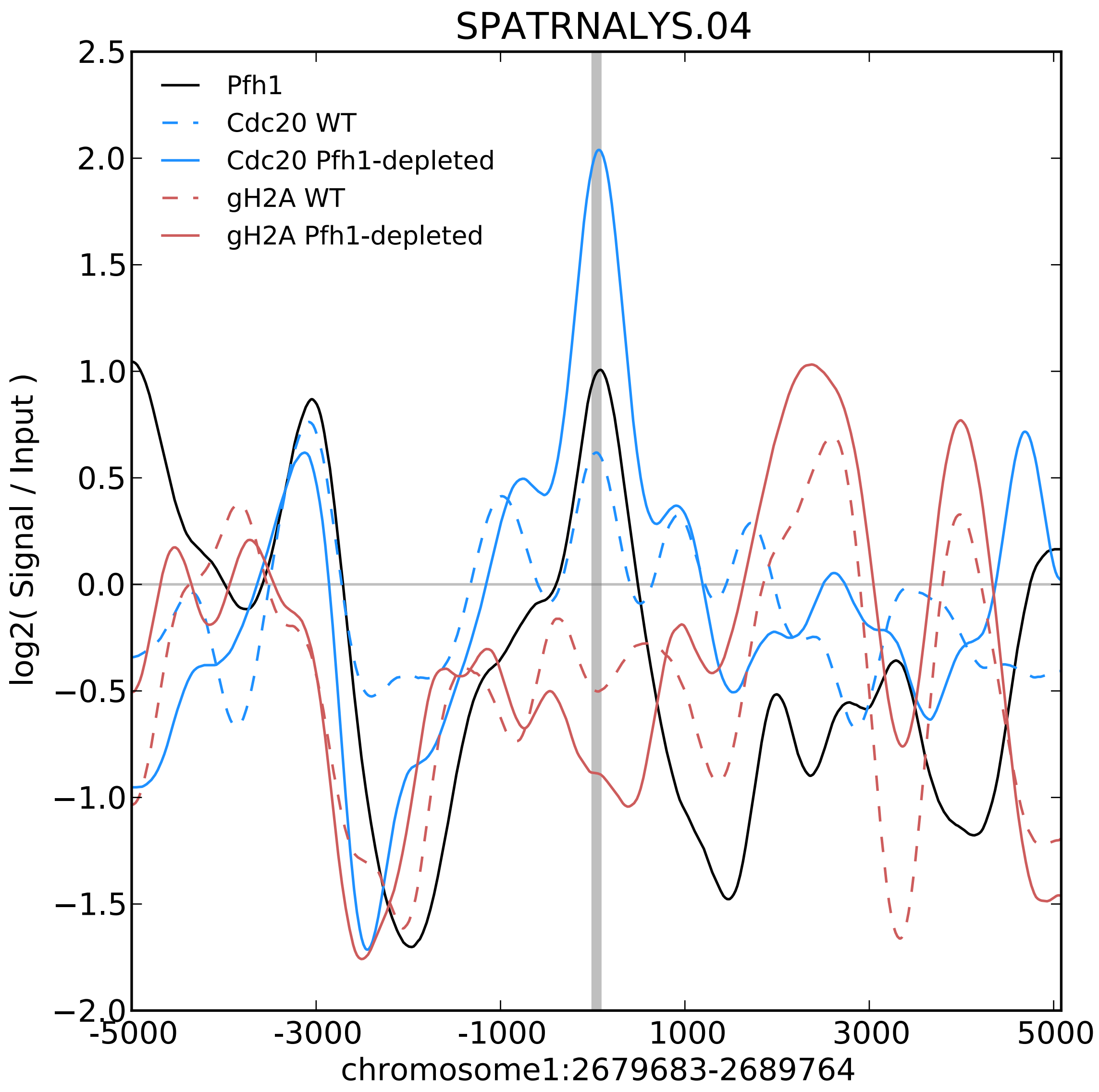

SPATRNALYS.04
Pfh1
Cdc20 WT
Cdc20 Pfh1-depleted
gH2A WT
gH2A Pfh1-depleted
2.5
2.0
1.5
1.0
0.5
0.0
−0.5
−1.0
−1.5
−2.0
log2(Signal / Input)
-5000
-3000
-1000
1000
3000
5000
chromosome1:2679683-2689764

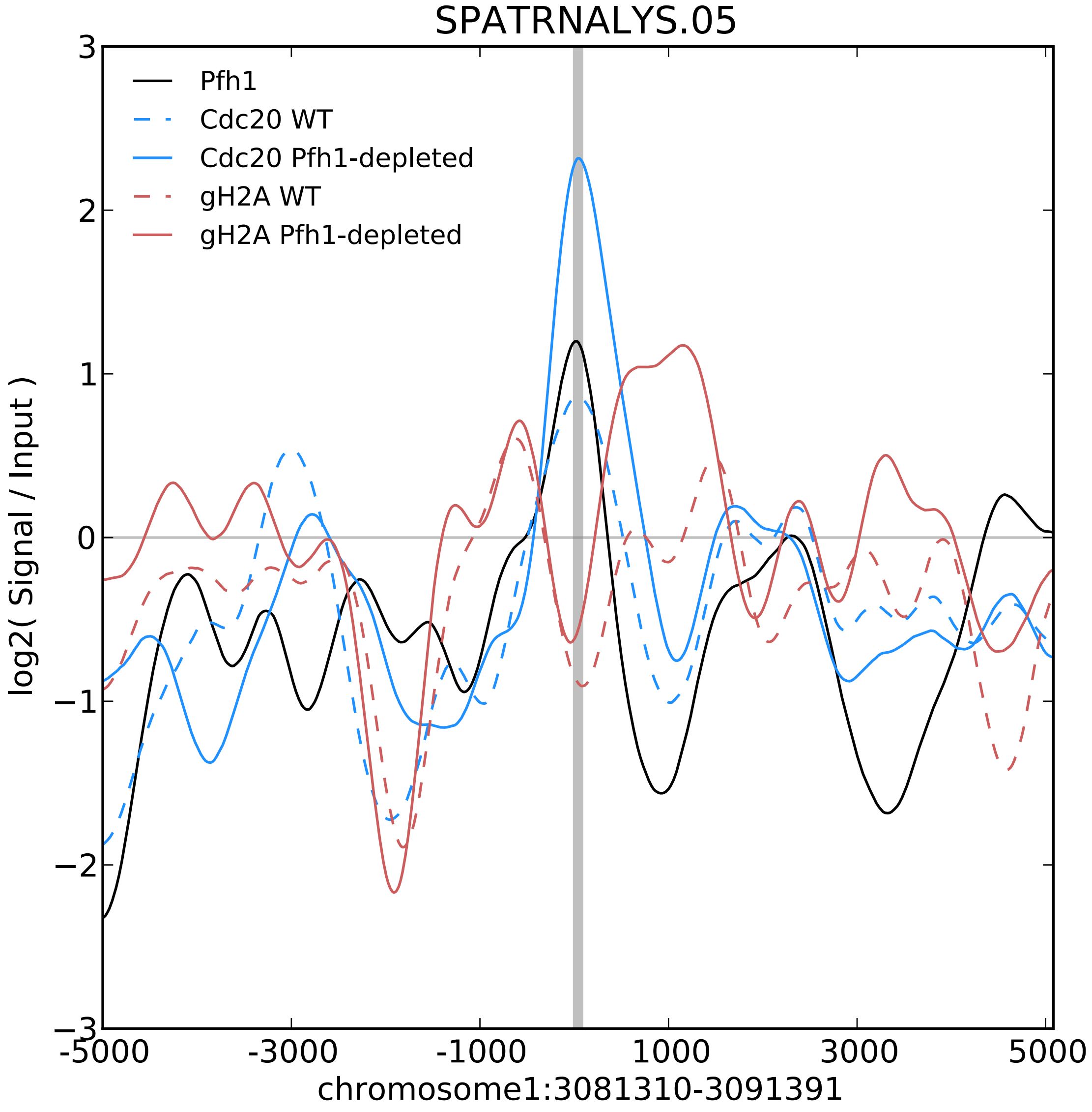

SPATRNALYS.05
Pfh1
Cdc20 WT
Cdc20 Pfh1-depleted
gH2A WT
gH2A Pfh1-depleted
log2(Signal / Input)
3
2
1
0
−1
−2
−3
-5000
-3000
-1000
1000
3000
5000
chromosome1:3081310-3091391

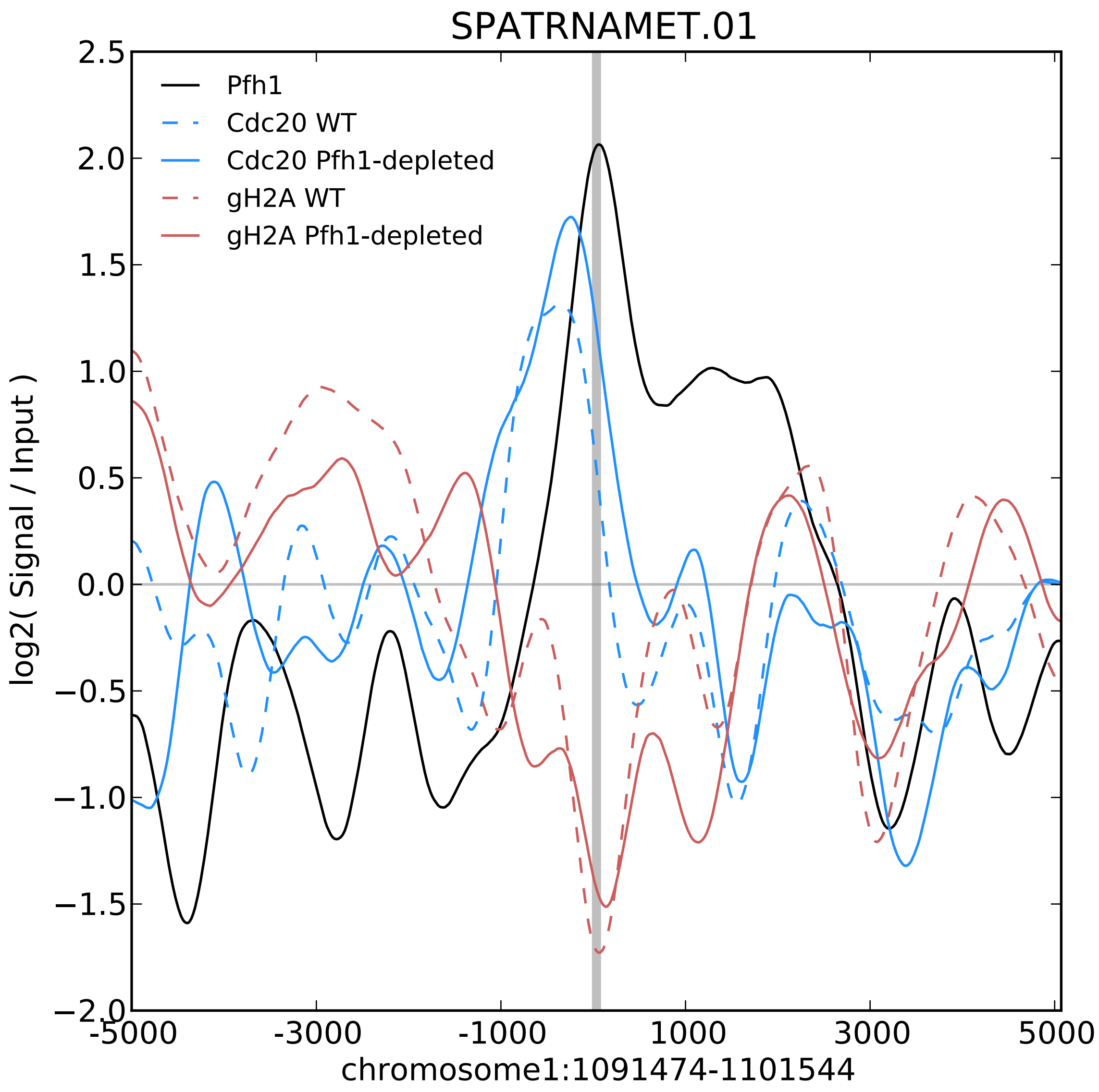

SPATRNAMET.01
Pfh1
Cdc20 WT
Cdc20 Pfh1-depleted
gH2A WT
gH2A Pfh1-depleted
log2(Signal / Input)
2.5
2.0
1.5
1.0
0.5
0.0
−0.5
−1.0
−1.5
−2.0
-5000
-3000
-1000
1000
3000
5000
chromosome1:1091474-1101544

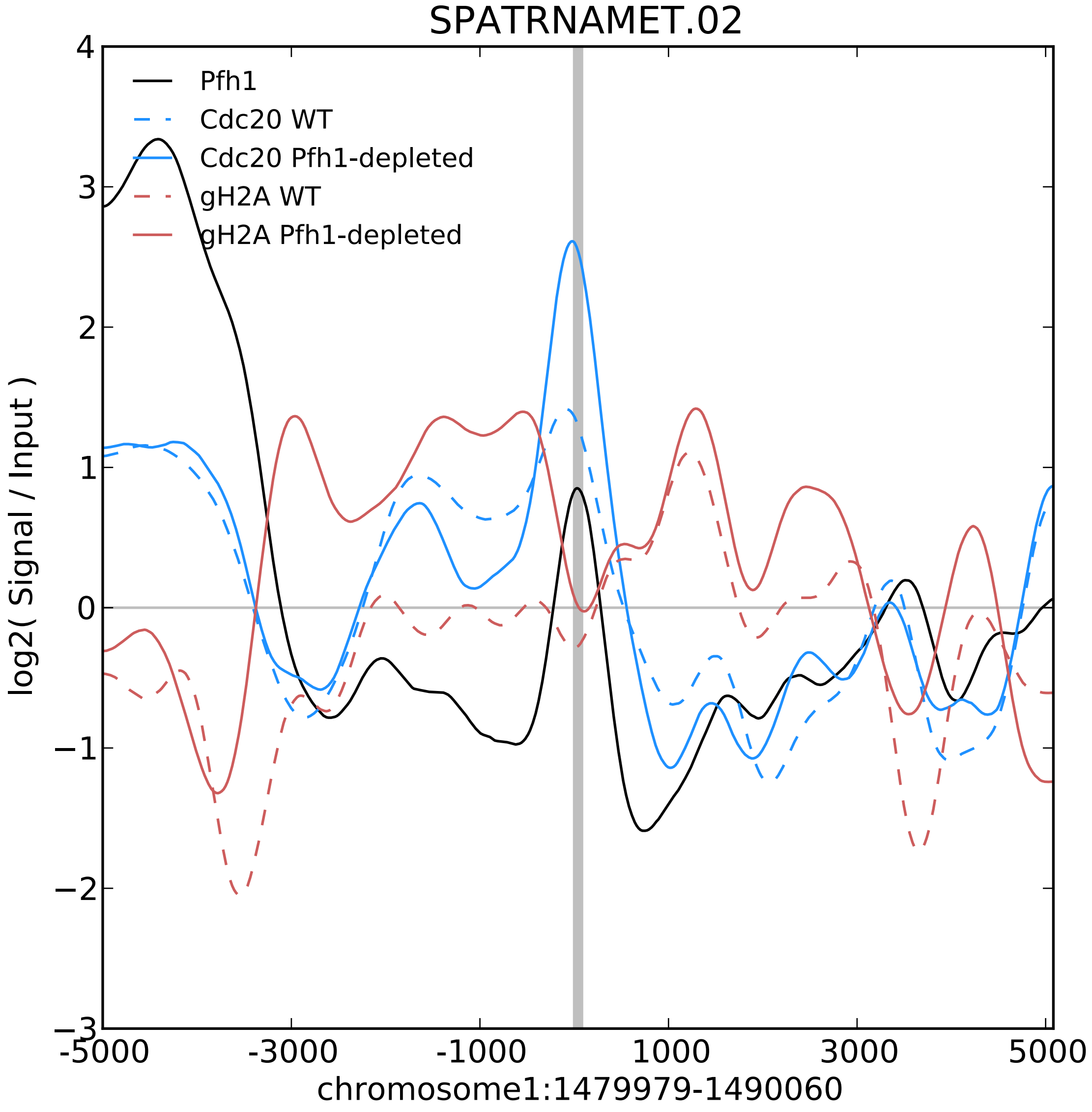
SPATRNAMET.02
Pfh1
Cdc20 WT
Cdc20 Pfh1-depleted
gH2A WT
gH2A Pfh1-depleted
log2(Signal / Input)
chromosome1:1479979-1490060
4
3
2
1
0
−1
−2
−3
-5000
-3000
-1000
1000
3000
5000

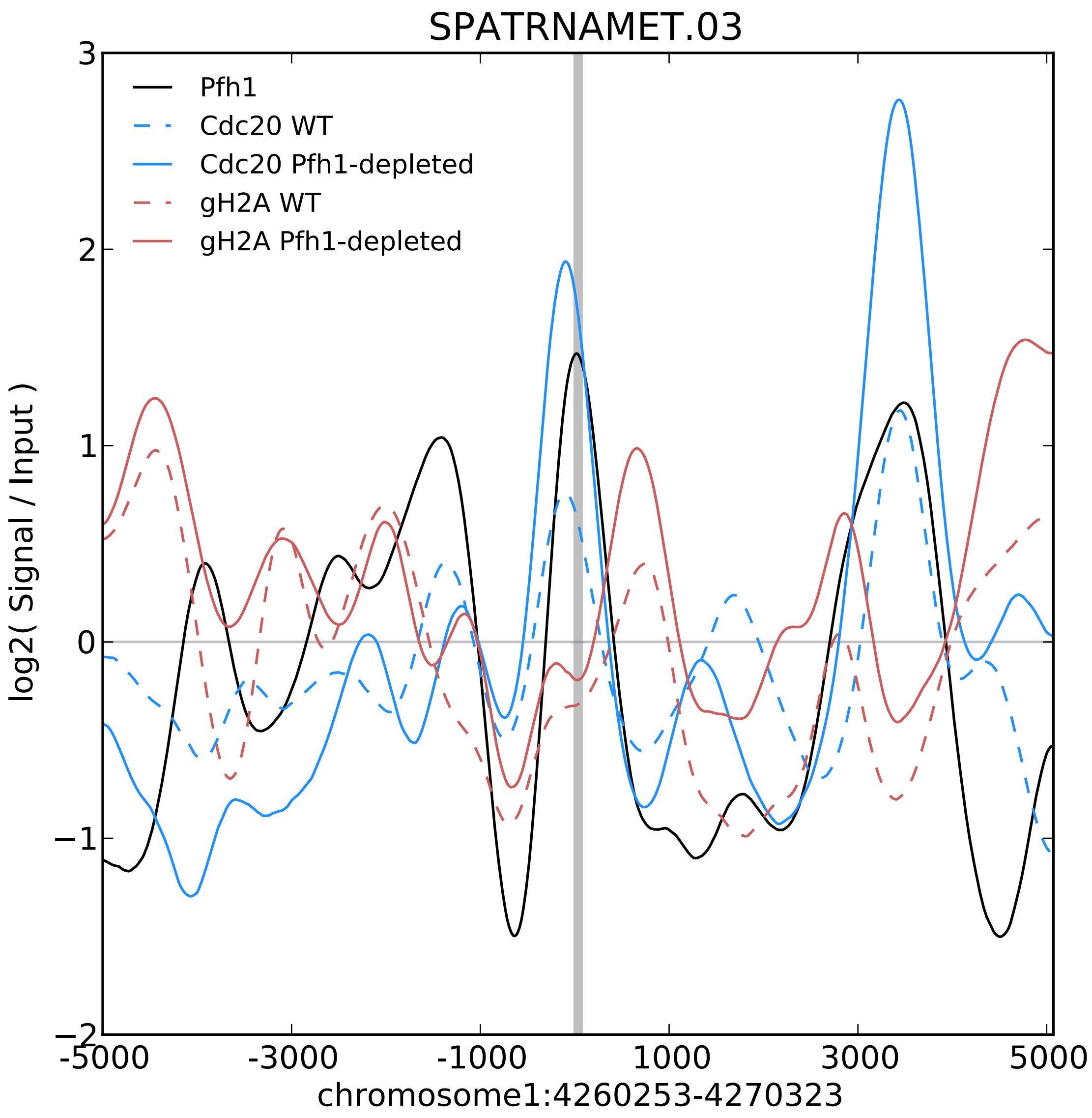
SPATRNAMET.03
Pfh1
Cdc20 WT
Cdc20 Pfh1-depleted
gH2A WT
gH2A Pfh1-depleted
log2(Signal / Input)
chromosome1:4260253-4270323
3
2
1
0
−1
−2
-5000
-3000
-1000
1000
3000
5000

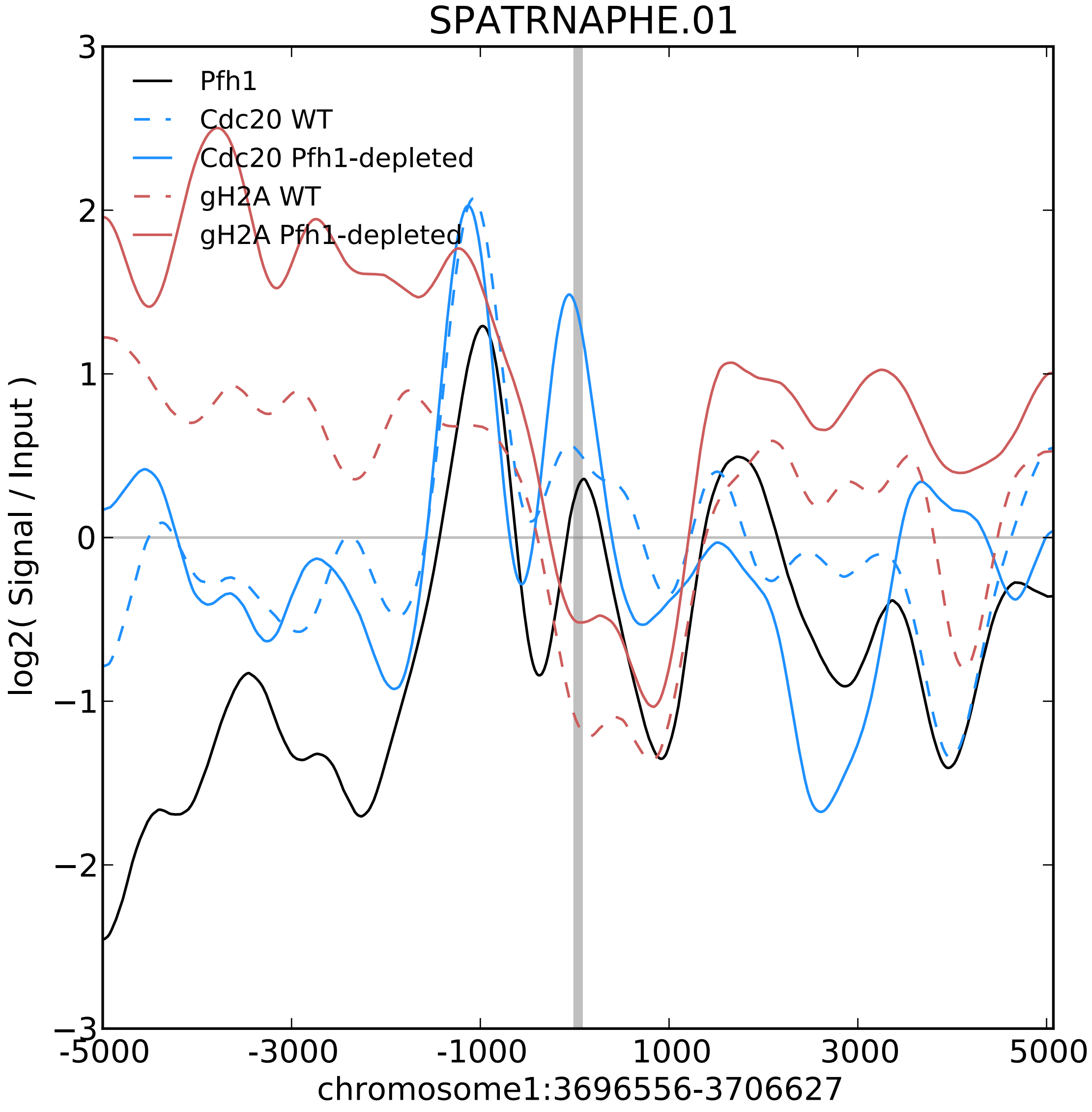

SPATRNAPHE.01
Pfh1
Cdc20 WT
Cdc20 Pfh1-depleted
gH2A WT
gH2A Pfh1-depleted
log2(Signal / Input)
3
2
1
0
−1
−2
−3
-5000
-3000
-1000
1000
3000
5000
chromosome1:3696556-3706627

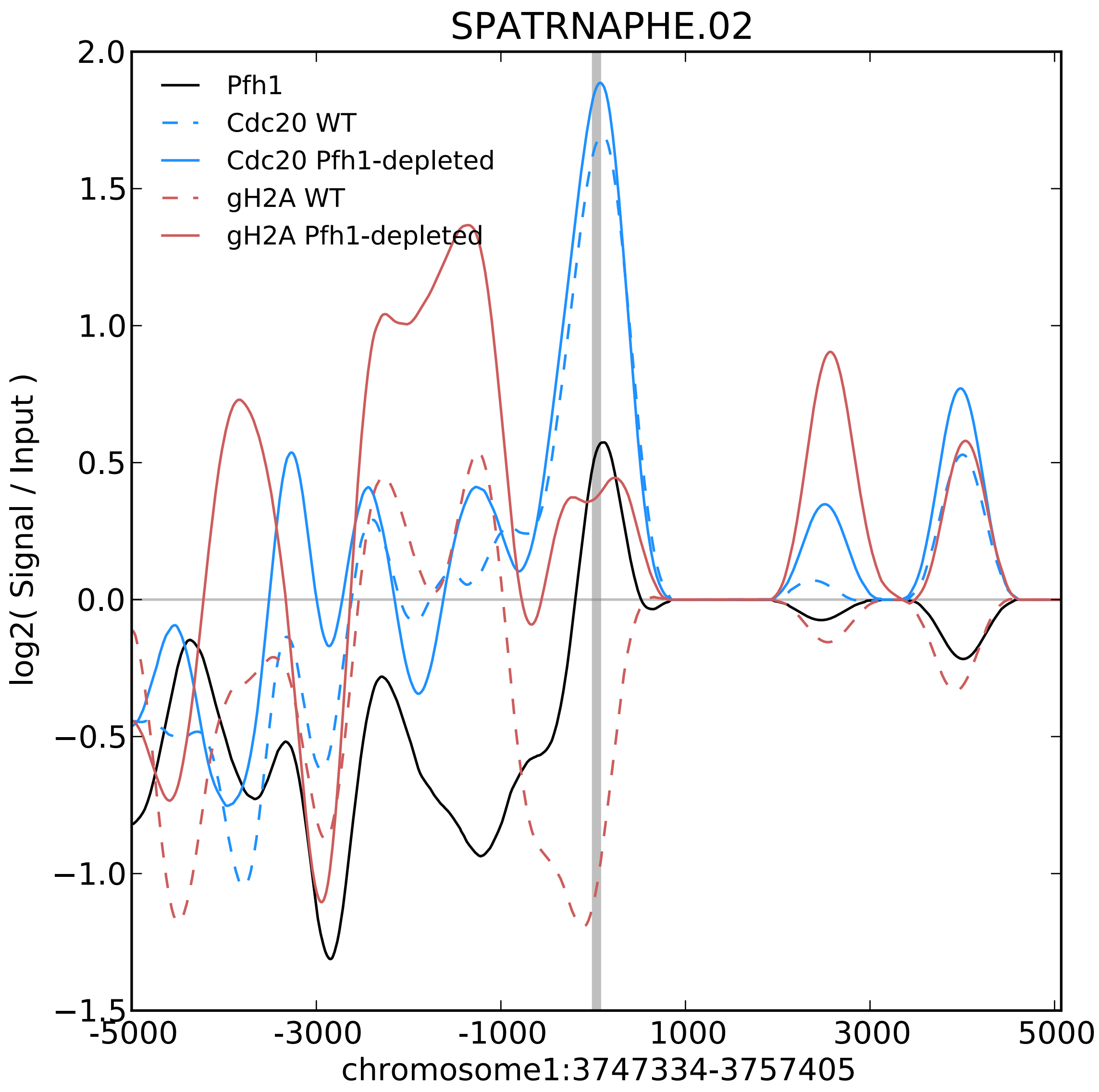
SPATRNAPHE.02
Pfh1
Cdc20 WT
Cdc20 Pfh1-depleted
gH2A WT
gH2A Pfh1-depleted
log2(Signal / Input)
2.0
1.5
1.0
0.5
0.0
−0.5
−1.0
−1.5
-5000
-3000
-1000
1000
3000
5000
chromosome1:3747334-3757405

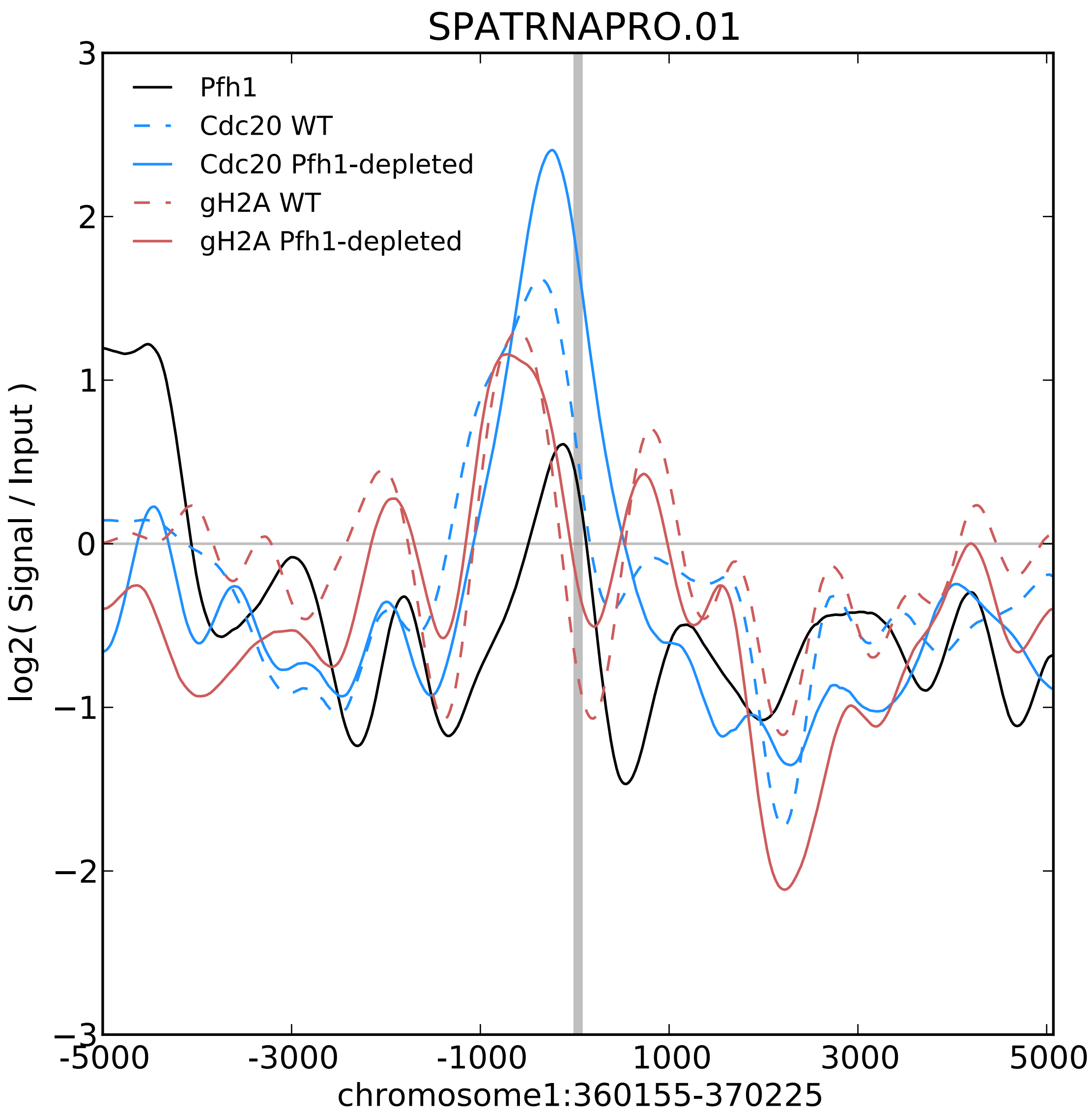
SPATRNAPRO.01
Pfh1
Cdc20 WT
Cdc20 Pfh1-depleted
gH2A WT
gH2A Pfh1-depleted
log2(Signal / Input)
3
2
1
0
−1
−2
−3
-5000
-3000
-1000
1000
3000
5000
chromosome1:360155-370225

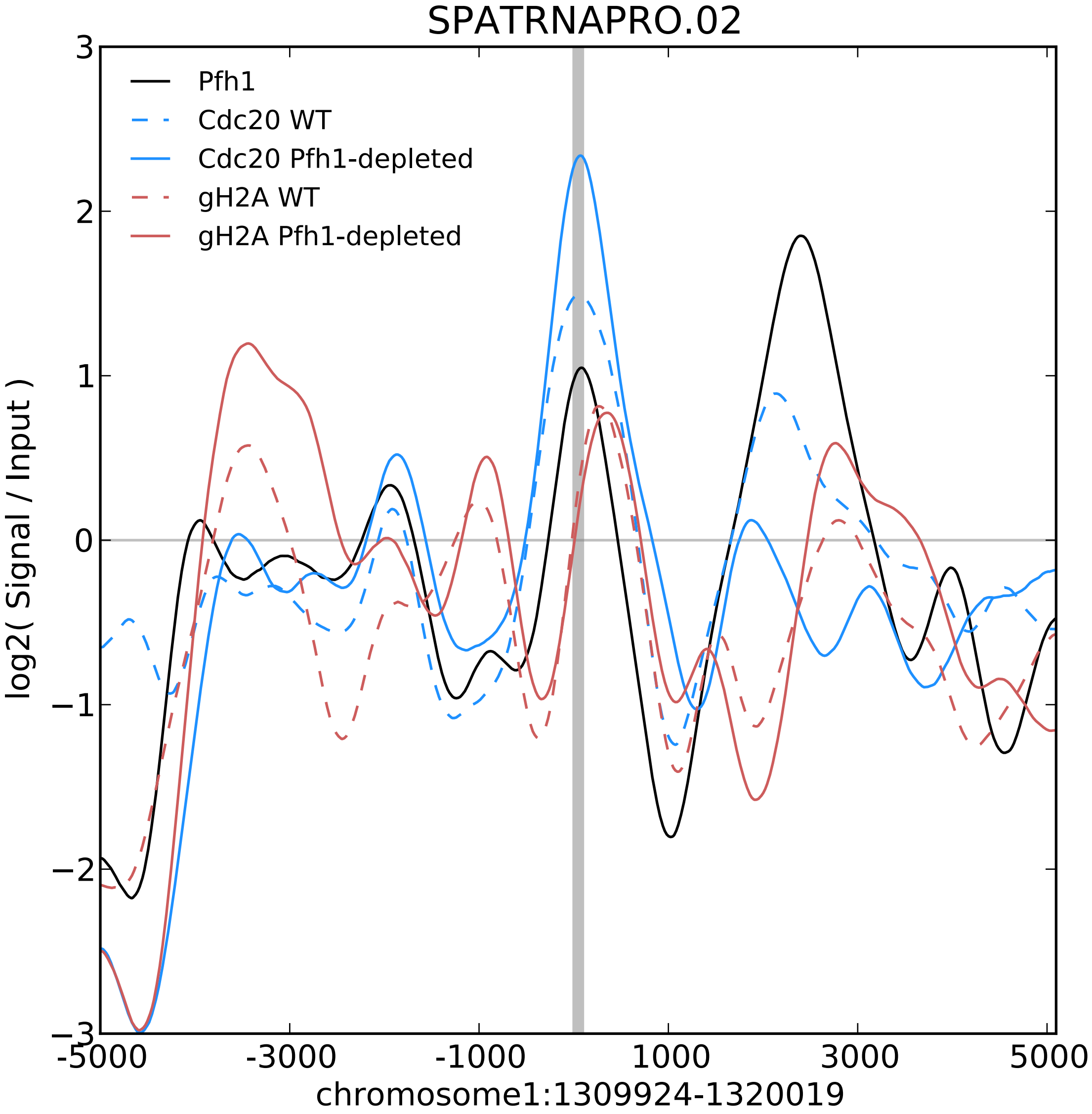
SPATRNAPRO.02
Pfh1
Cdc20 WT
Cdc20 Pfh1-depleted
gH2A WT
gH2A Pfh1-depleted
log2(Signal / Input)
chromosome1:1309924-1320019
3
2
1
0
−1
−2
−3
-5000
-3000
-1000
1000
3000
5000

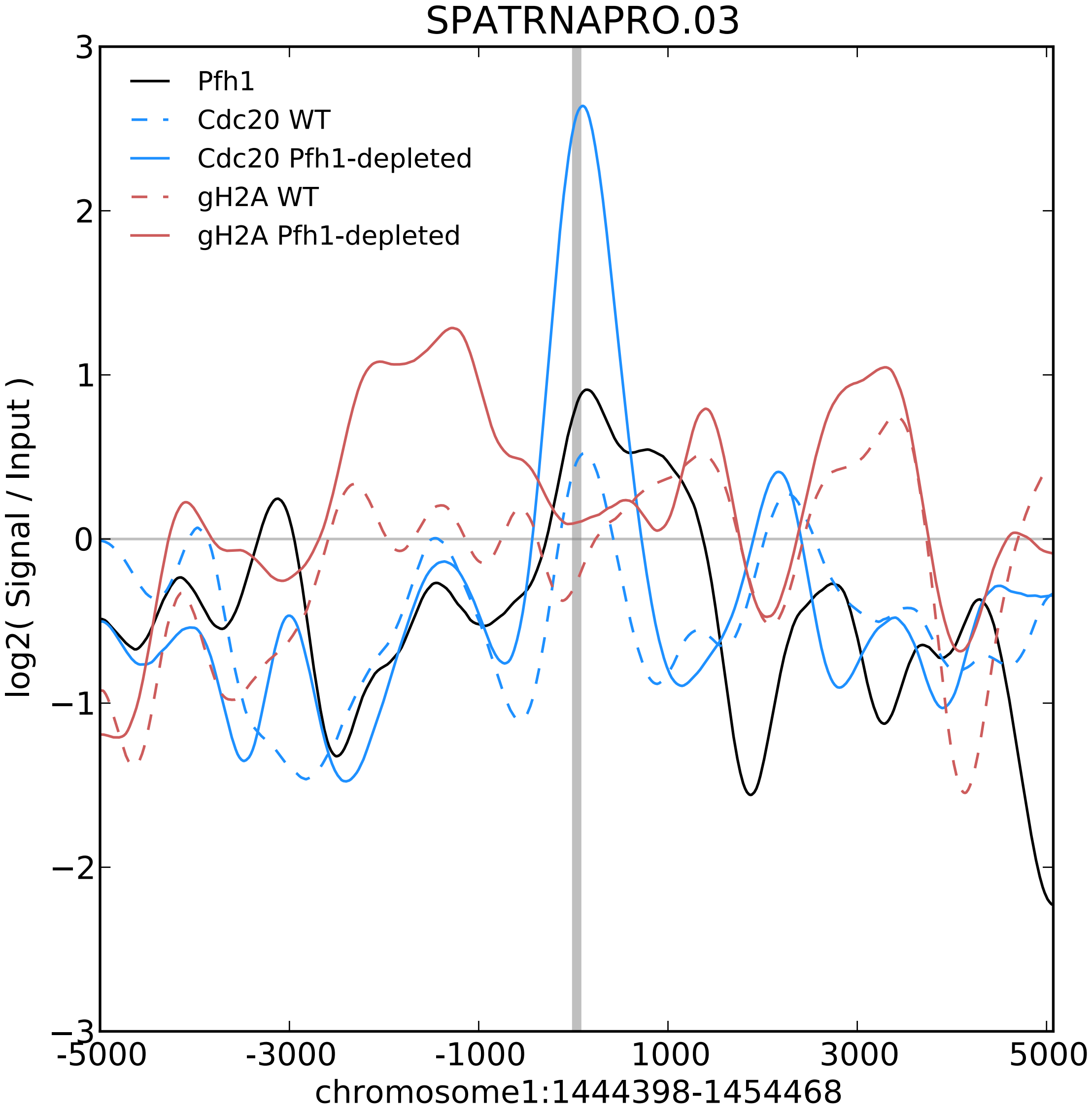

SPATRNAPRO.03
Pfh1
Cdc20 WT
Cdc20 Pfh1-depleted
gH2A WT
gH2A Pfh1-depleted
log2(Signal / Input)
3
2
1
0
−1
−2
−3
-5000
-3000
-1000
1000
3000
5000
chromosome1:1444398-1454468

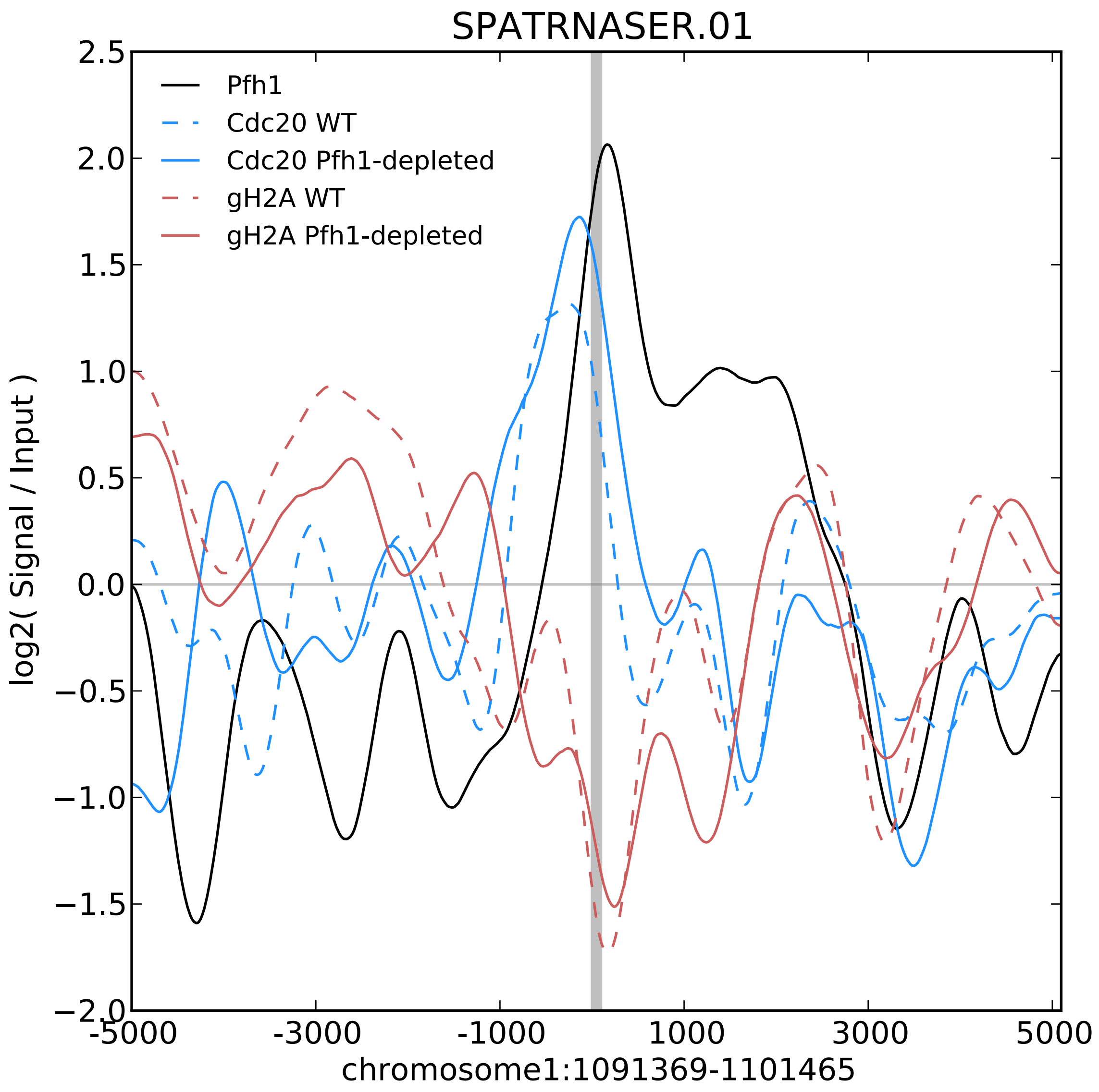
SPATRNASER.01
Pfh1
Cdc20 WT
Cdc20 Pfh1-depleted
gH2A WT
gH2A Pfh1-depleted
log2(Signal / Input)
chromosome1:1091369-1101465
2.5
2.0
1.5
1.0
0.5
0.0
−0.5
−1.0
−1.5
−2.0
-5000
-3000
-1000
1000
3000
5000

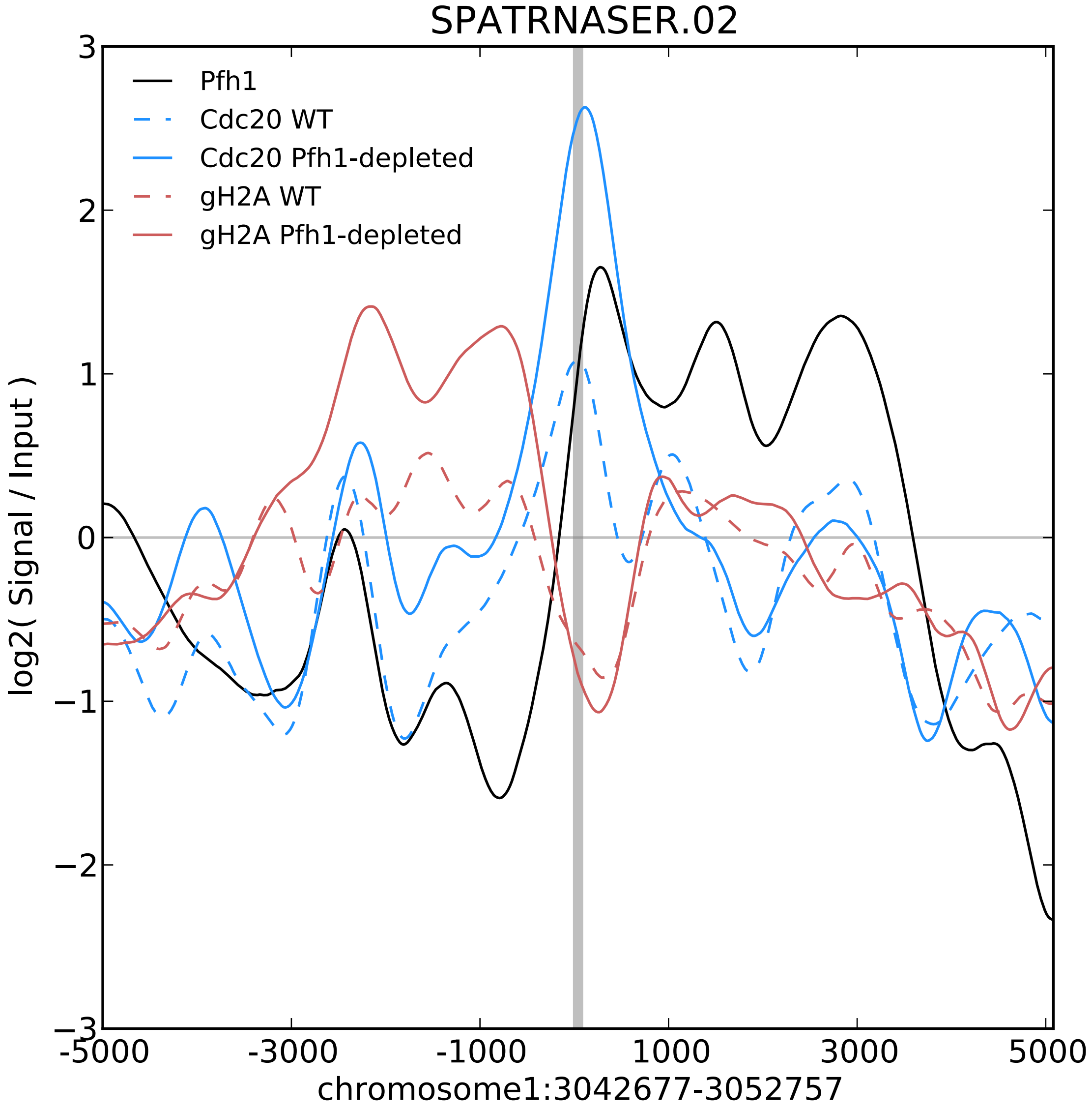
SPATRNASER.02
Pfh1
Cdc20 WT
Cdc20 Pfh1-depleted
gH2A WT
gH2A Pfh1-depleted
log2(Signal / Input)
3
2
1
0
−1
−2
−3
-5000
-3000
-1000
1000
3000
5000
chromosome1:3042677-3052757

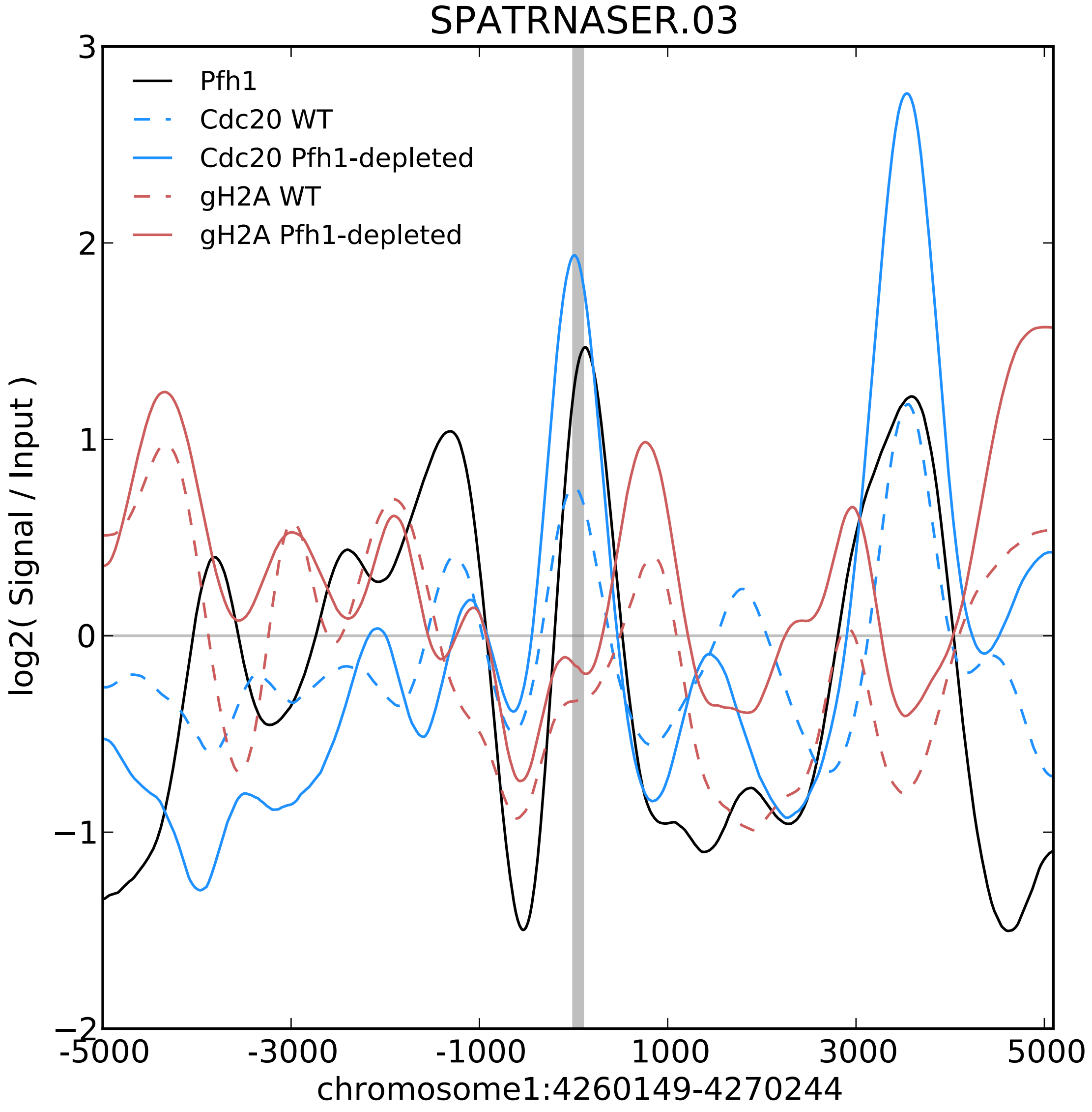

SPATRNASER.03
Pfh1
Cdc20 WT
Cdc20 Pfh1-depleted
gH2A WT
gH2A Pfh1-depleted
log2(Signal / Input)
3
2
1
0
−1
−2
-5000
-3000
-1000
1000
3000
5000
chromosome1:4260149-4270244

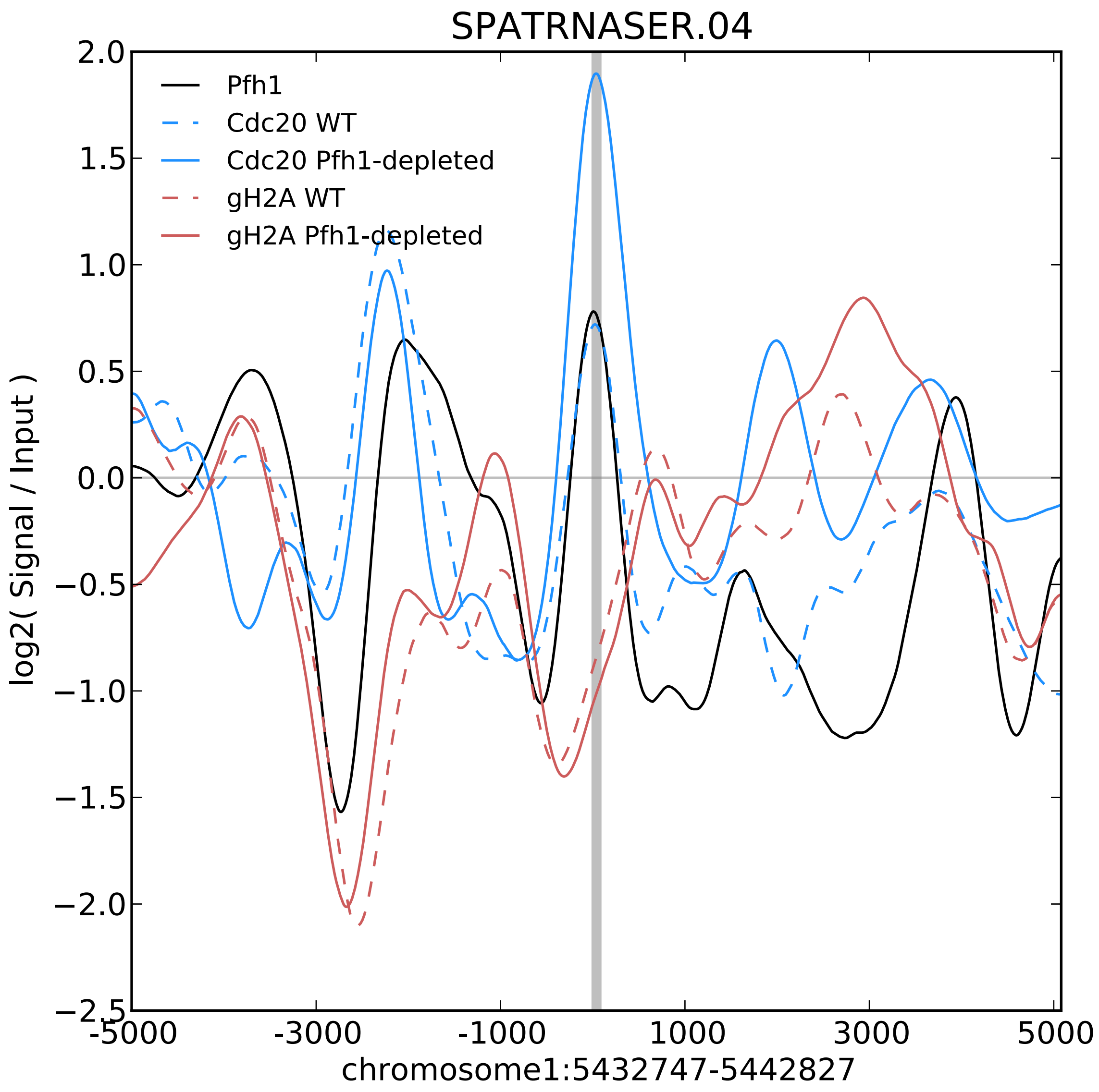
SPATRNASER.04
Pfh1
Cdc20 WT
Cdc20 Pfh1-depleted
gH2A WT
gH2A Pfh1-depleted
log2(Signal / Input)
2.0
1.5
1.0
0.5
0.0
−0.5
−1.0
−1.5
−2.0
−2.5
-5000
-3000
-1000
1000
3000
5000
chromosome1:5432747-5442827

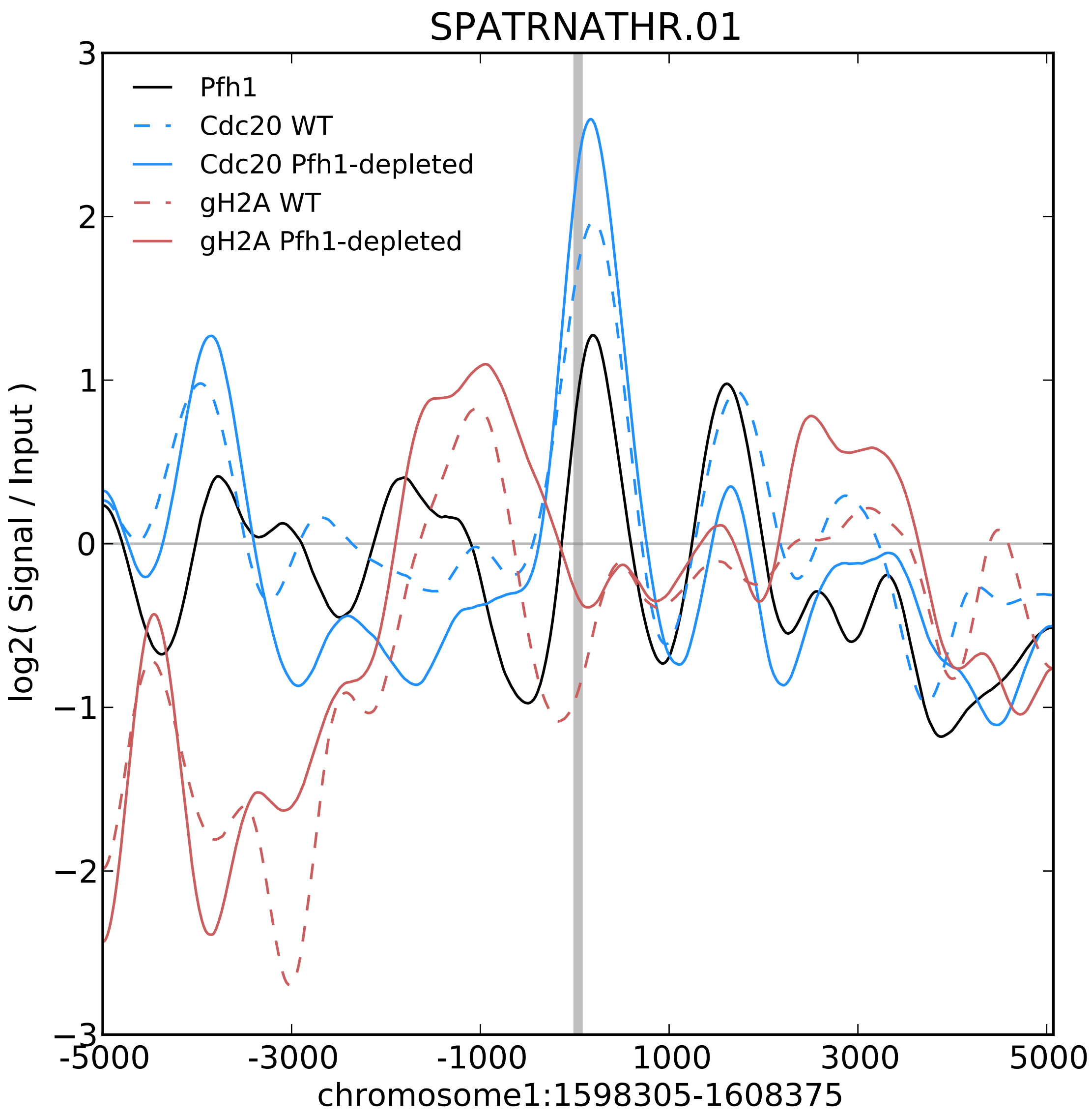
SPATRNATHR.01
Pfh1
Cdc20 WT
Cdc20 Pfh1-depleted
gH2A WT
gH2A Pfh1-depleted
log2(Signal / Input)
3
2
1
0
−1
−2
−3
-5000
-3000
-1000
1000
3000
5000
chromosome1:1598305-1608375

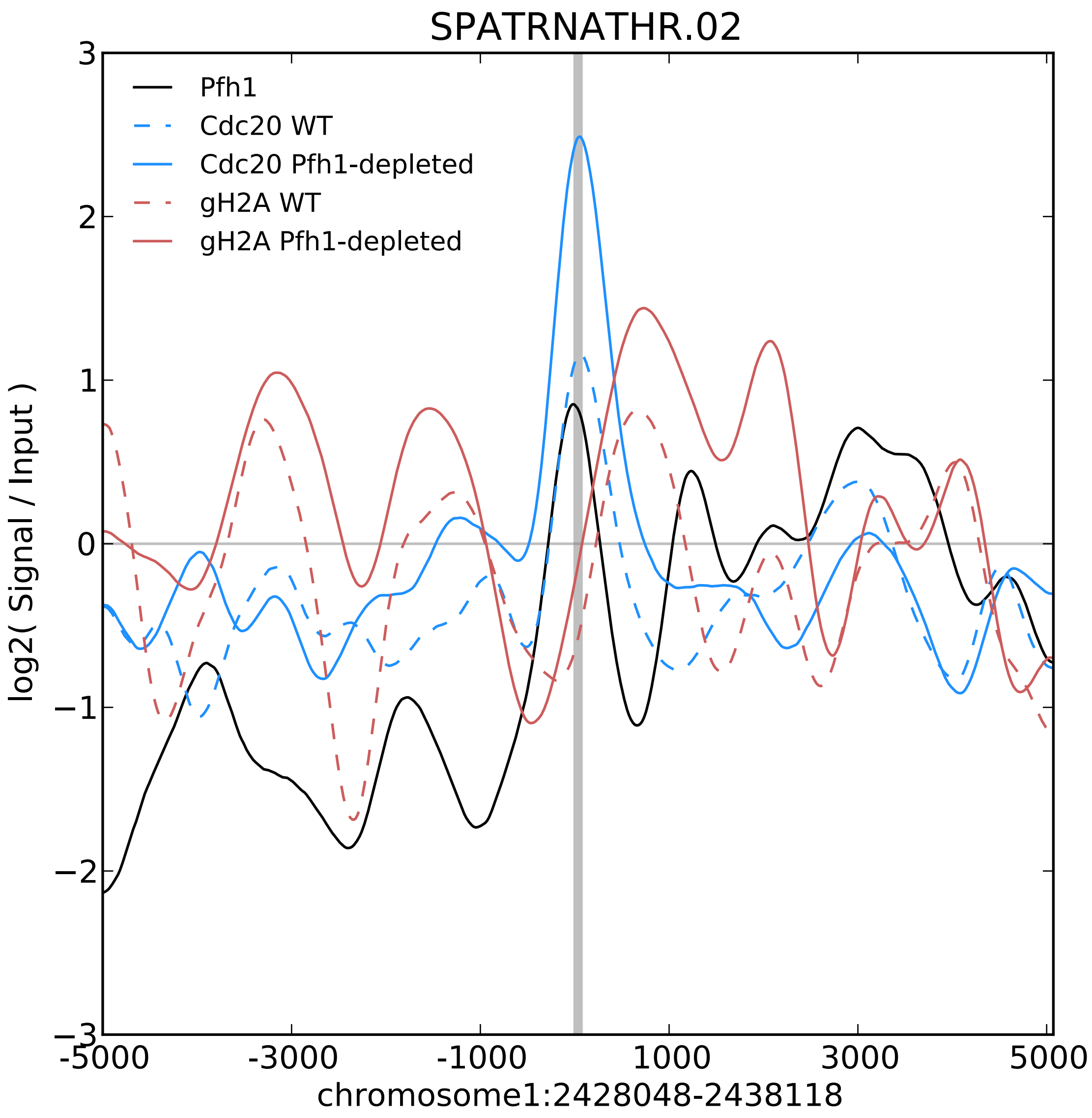
SPATRNATHR.02
Pfh1
Cdc20 WT
Cdc20 Pfh1-depleted
gH2A WT
gH2A Pfh1-depleted
log2(Signal / Input)
3
2
1
0
−1
−2
−3
-5000
-3000
-1000
1000
3000
5000
chromosome1:2428048-2438118

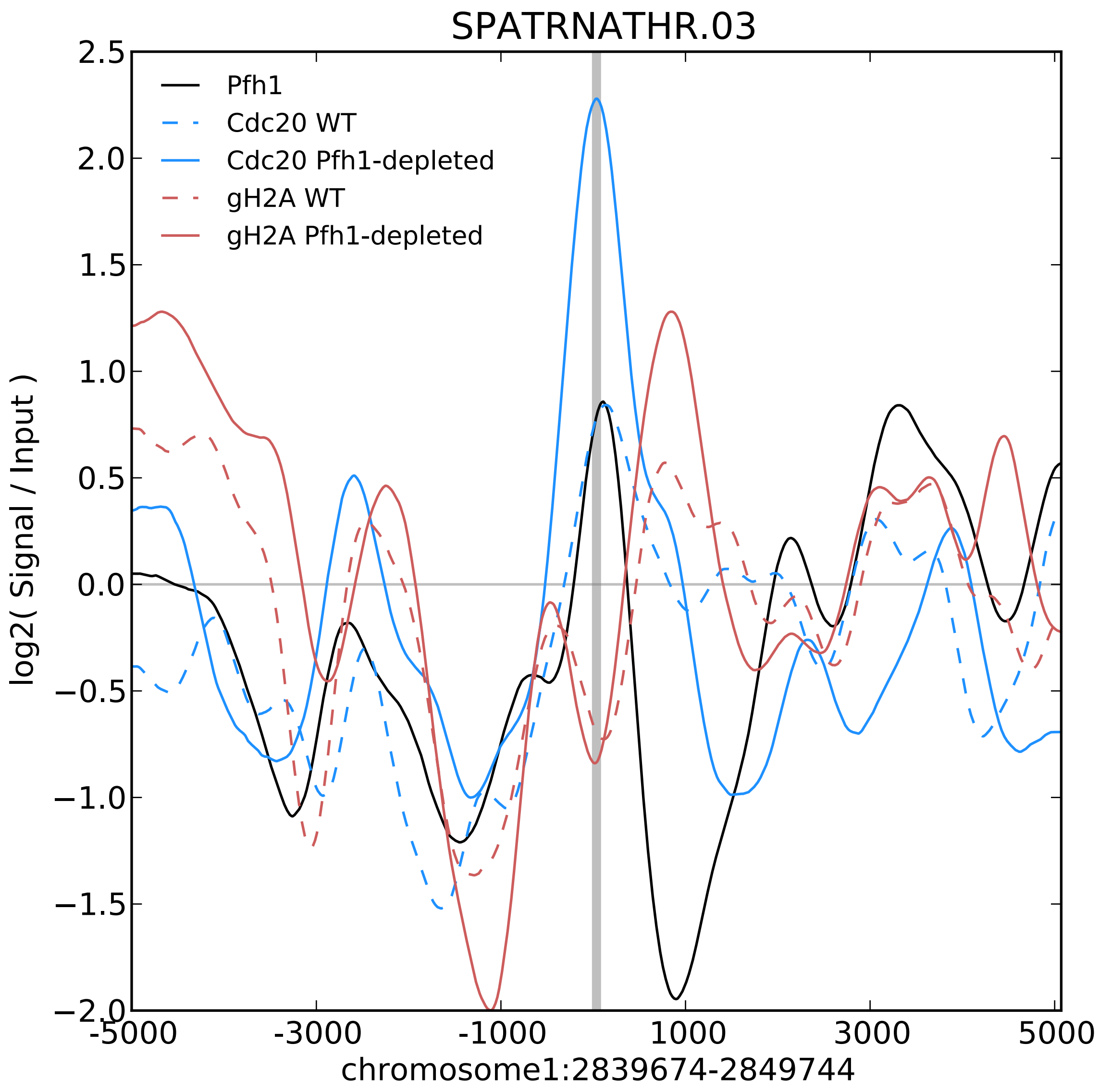
SPATRNATHR.03
Pfh1
Cdc20 WT
Cdc20 Pfh1-depleted
gH2A WT
gH2A Pfh1-depleted
log2(Signal / Input)
2.5
2.0
1.5
1.0
0.5
0.0
−0.5
−1.0
−1.5
−2.0
-5000
-3000
-1000
1000
3000
5000
chromosome1:2839674-2849744

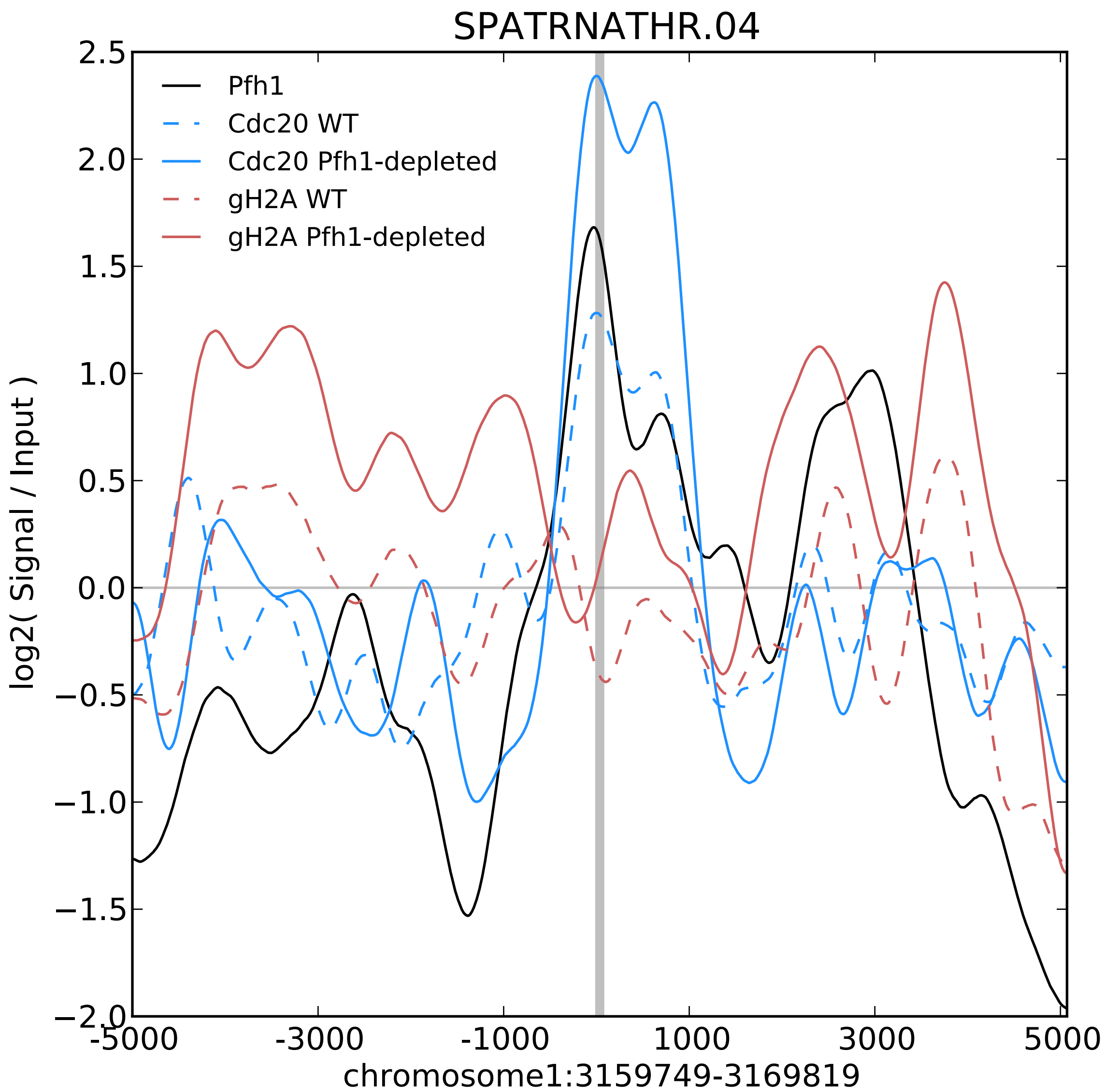

SPATRNATHR.04
Pfh1
Cdc20 WT
Cdc20 Pfh1-depleted
gH2A WT
gH2A Pfh1-depleted
log2(Signal / Input)
2.5
2.0
1.5
1.0
0.5
0.0
−0.5
−1.0
−1.5
−2.0
-5000
-3000
-1000
1000
3000
5000
chromosome1:3159749-3169819

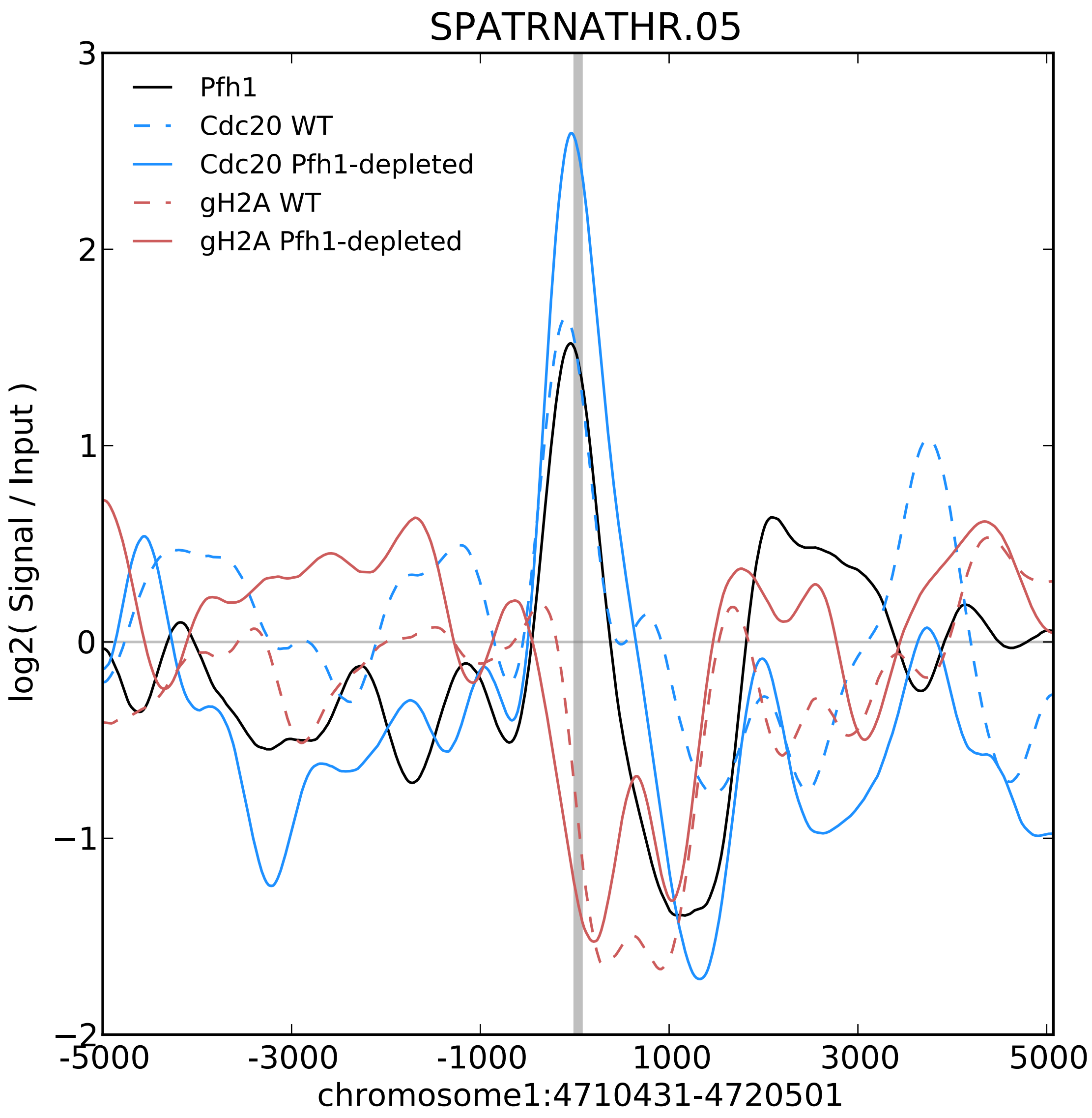

SPATRNATHR.05
Pfh1
Cdc20 WT
Cdc20 Pfh1-depleted
gH2A WT
gH2A Pfh1-depleted
log2(Signal / Input)
3
2
1
0
−1
−2
-5000
-3000
-1000
1000
3000
5000
chromosome1:4710431-4720501

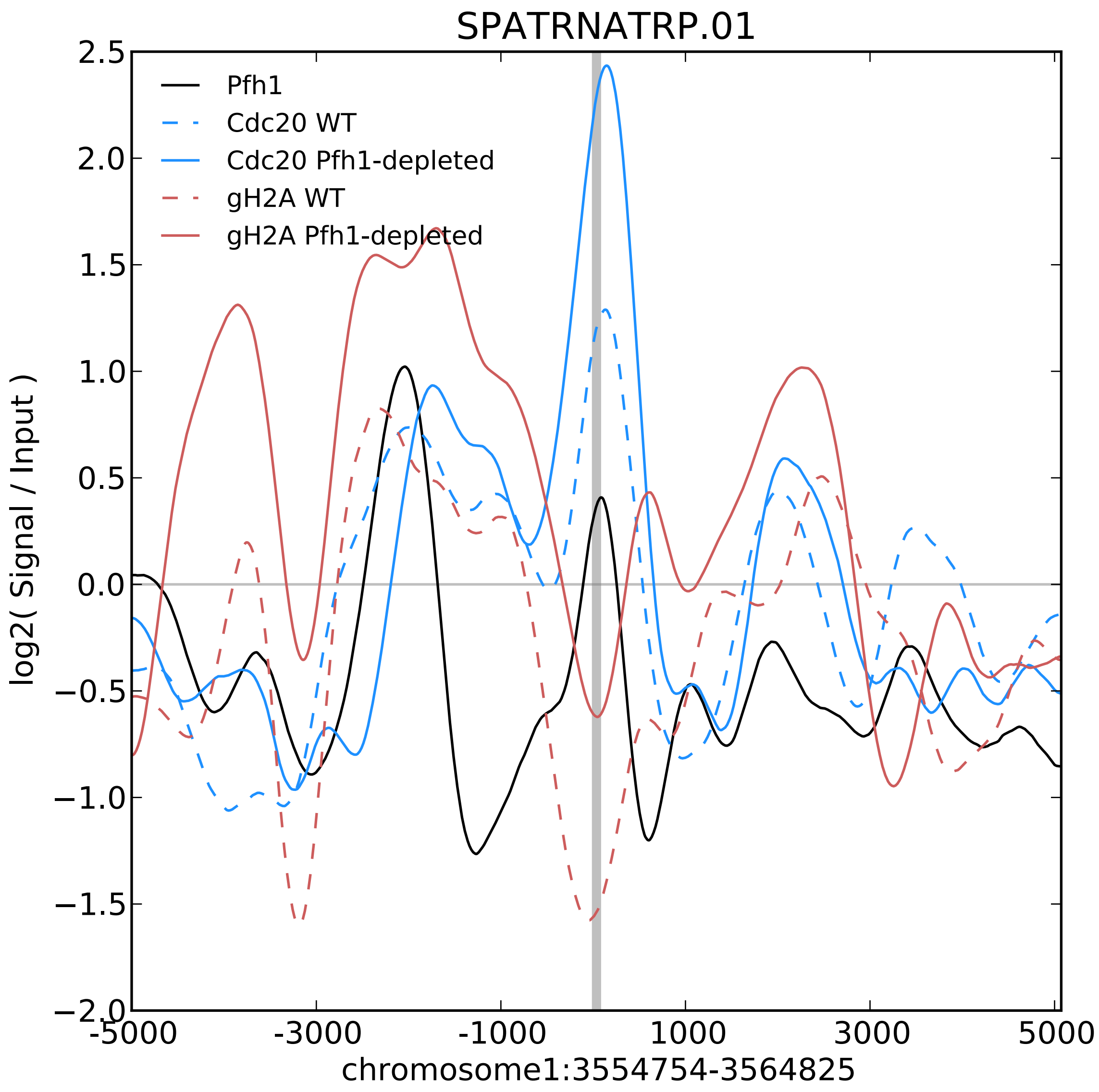
SPATRNATRP.01
Pfh1
Cdc20 WT
Cdc20 Pfh1-depleted
gH2A WT
gH2A Pfh1-depleted
log2(Signal / Input)
2.5
2.0
1.5
1.0
0.5
0.0
−0.5
−1.0
−1.5
−2.0
-5000
-3000
-1000
1000
3000
5000
chromosome1:3554754-3564825

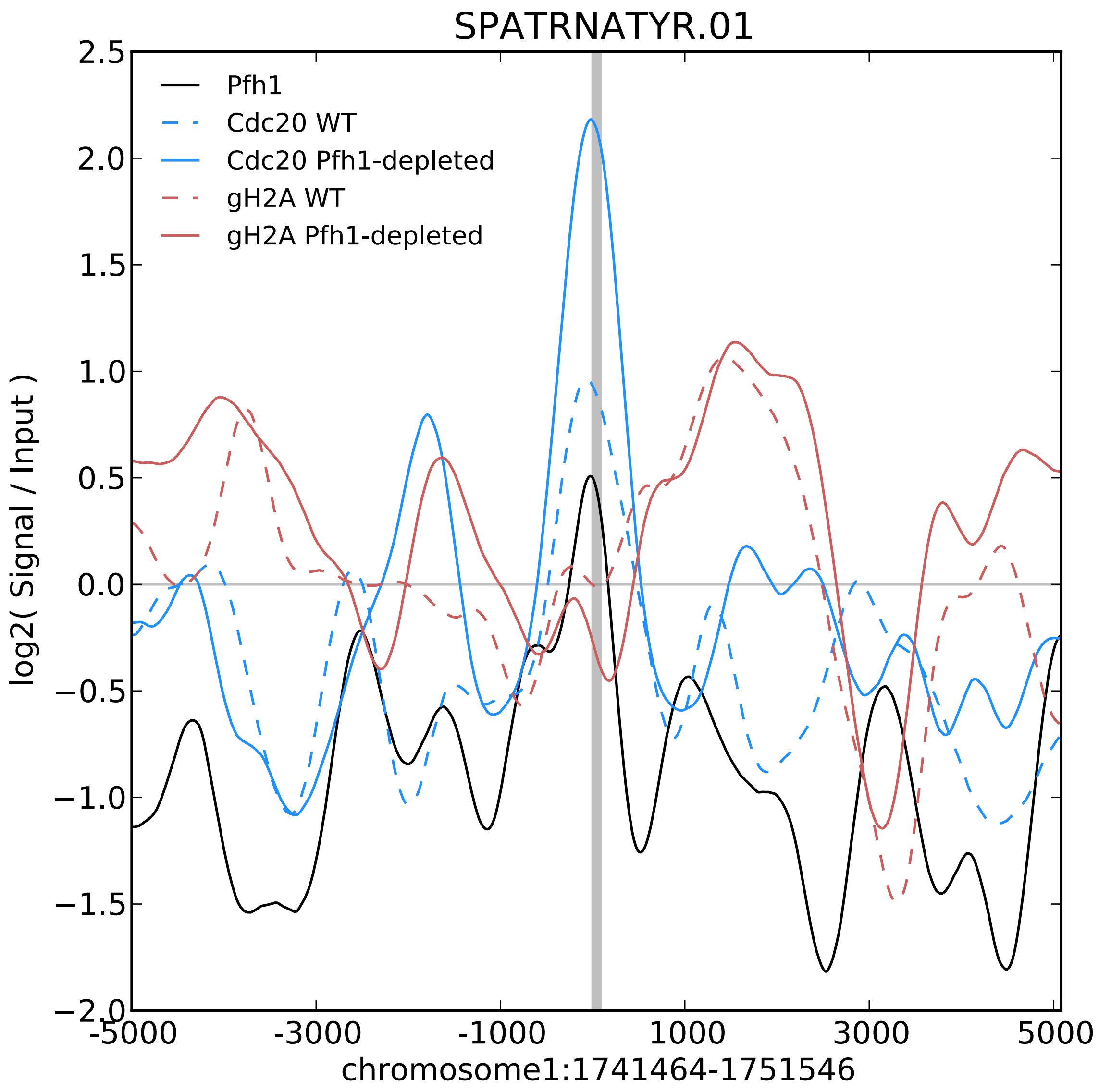
SPATRNATYR.01
Pfh1
Cdc20 WT
Cdc20 Pfh1-depleted
gH2A WT
gH2A Pfh1-depleted
log2(Signal / Input)
2.5
2.0
1.5
1.0
0.5
0.0
−0.5
−1.0
−1.5
−2.0
-5000
-3000
-1000
1000
3000
5000
chromosome1:1741464-1751546

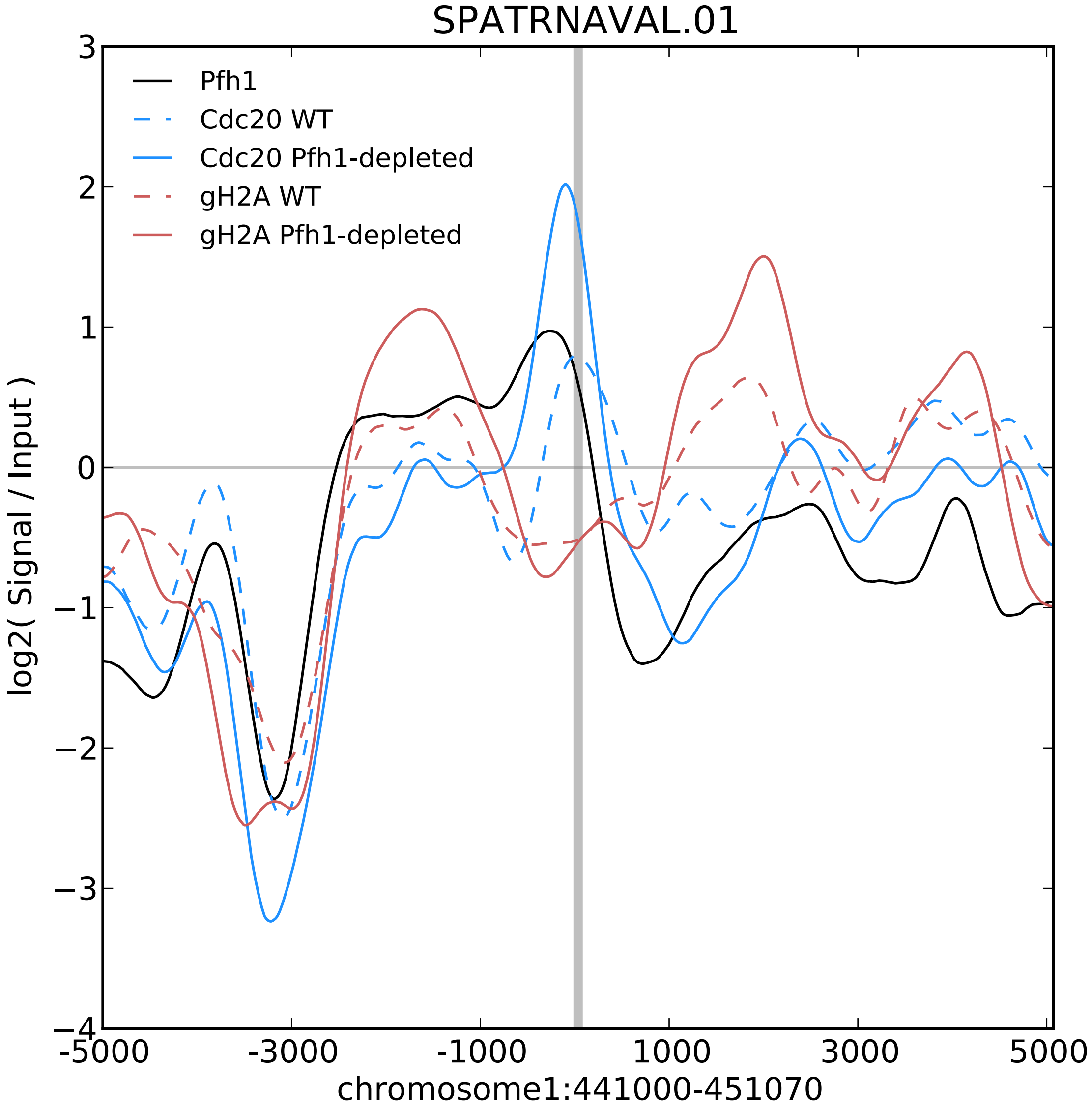
SPATRNAVAL.01
Pfh1
Cdc20 WT
Cdc20 Pfh1-depleted
gH2A WT
gH2A Pfh1-depleted
log2(Signal / Input)
chromosome1:441000-451070
3
2
1
0
−1
−2
−3
−4
-5000
-3000
-1000
1000
3000
5000

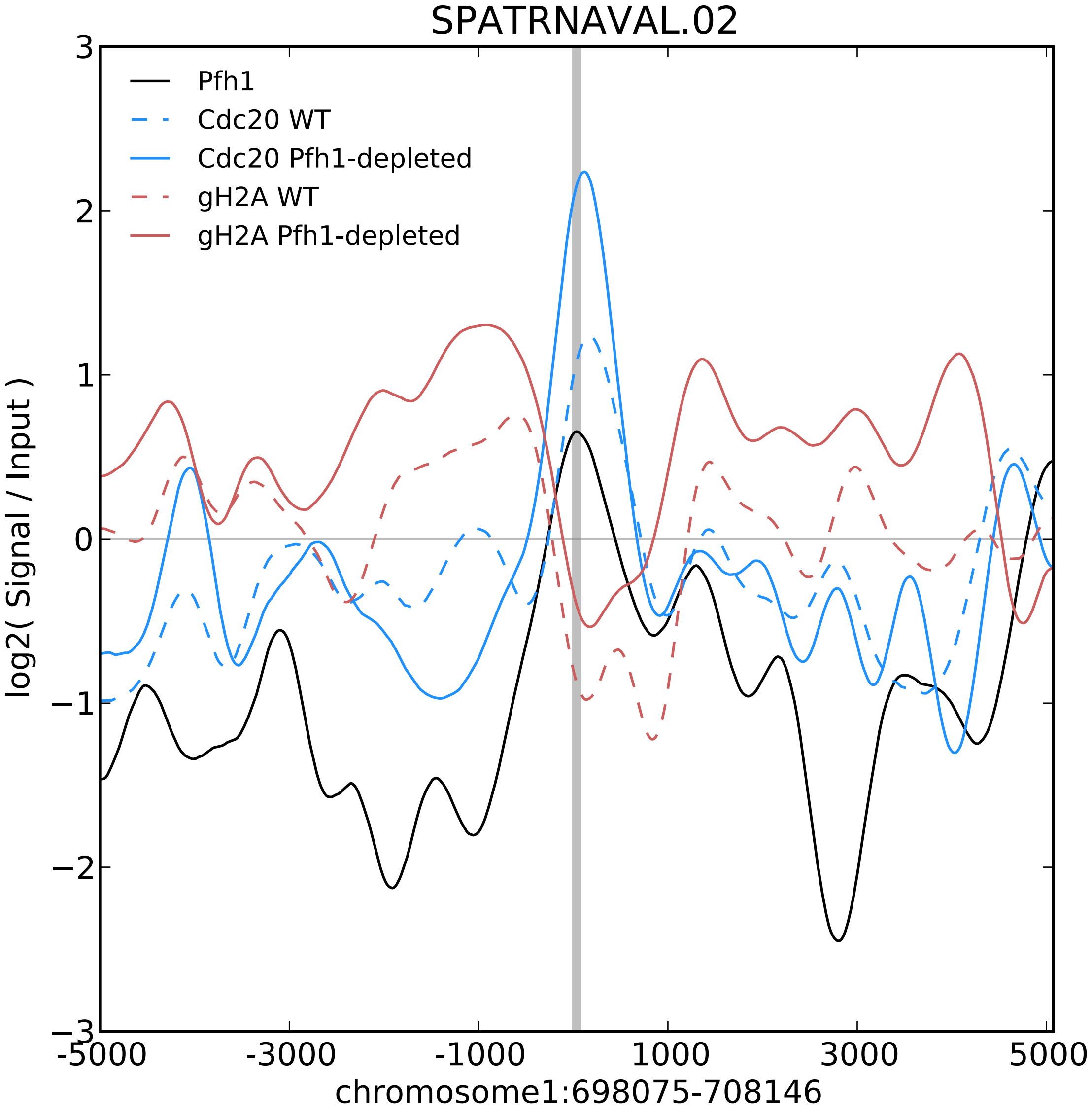
SPATRNAVAL.02
Pfh1
Cdc20 WT
Cdc20 Pfh1-depleted
gH2A WT
gH2A Pfh1-depleted
log2(Signal / Input)
3
2
1
0
−1
−2
−3
-5000
-3000
-1000
1000
3000
5000
chromosome1:698075-708146

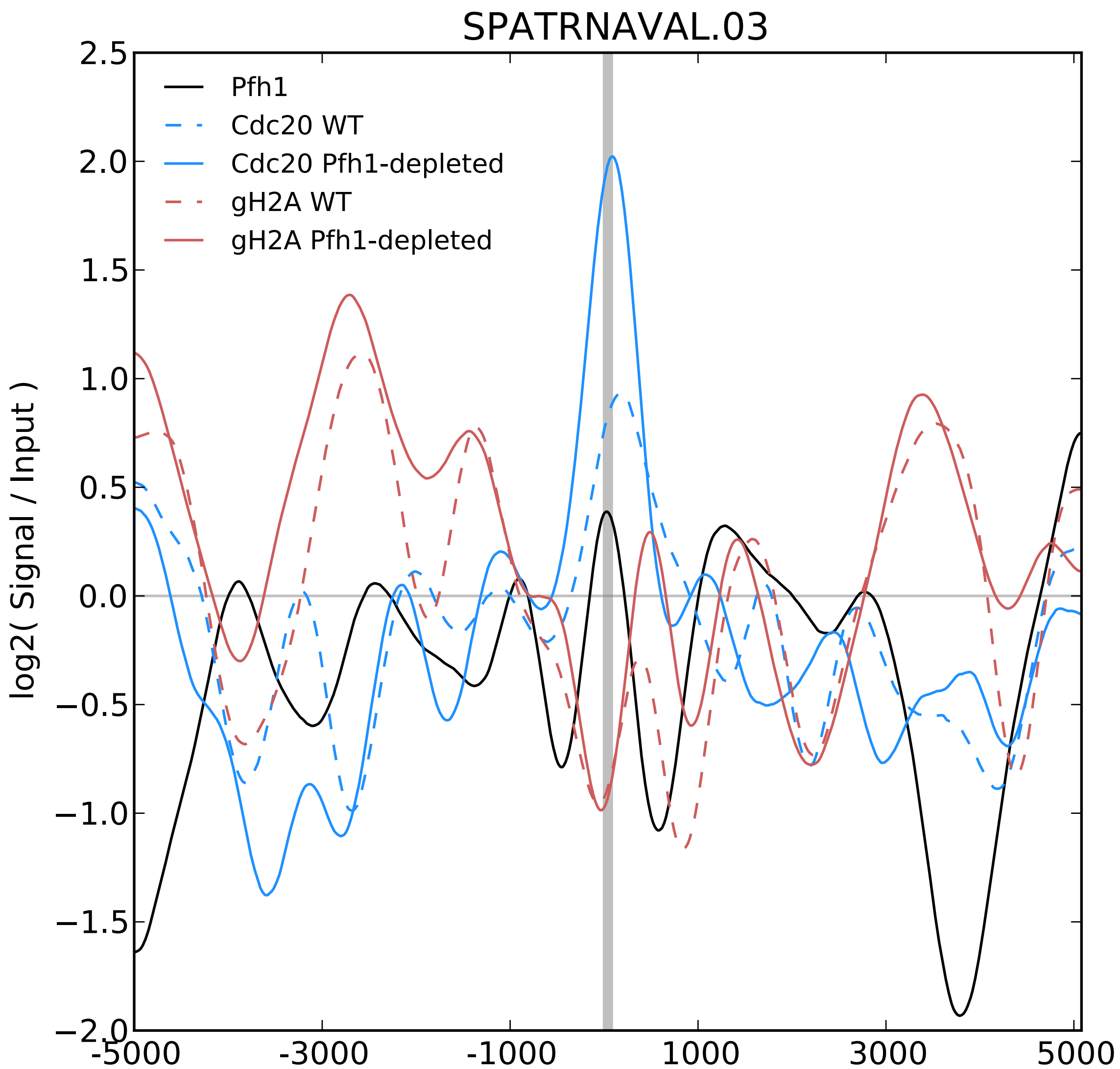

SPATRNAVAL.03
Pfh1
Cdc20 WT
Cdc20 Pfh1-depleted
gH2A WT
gH2A Pfh1-depleted
2.5
2.0
1.5
1.0
0.5
0.0
−0.5
−1.0
−1.5
−2.0
log2(Signal / Input)
-5000
-3000
-1000
1000
3000
5000
chromosome1:2209733-2219813

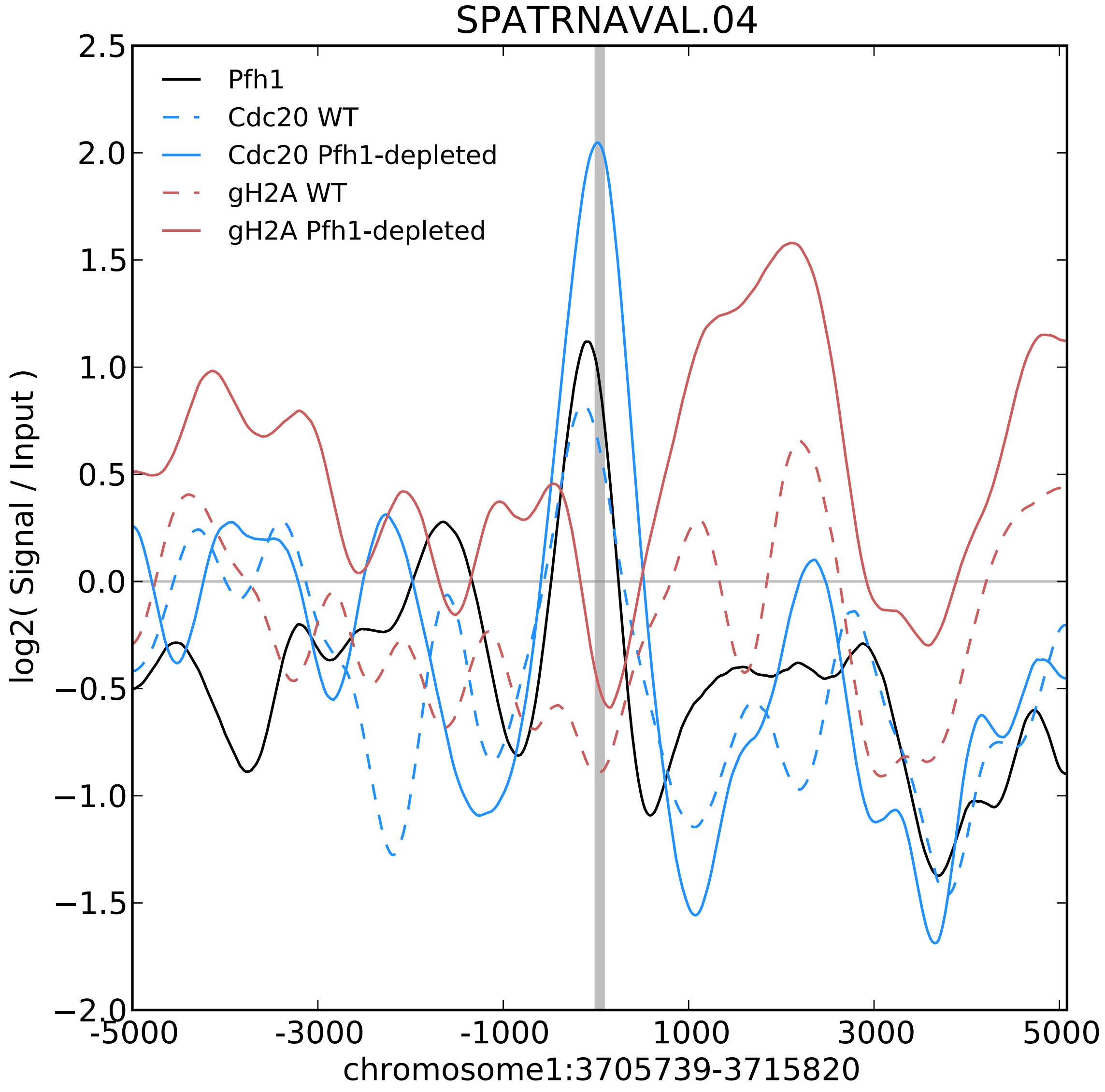

SPATRNAVAL.04
Pfh1
Cdc20 WT
Cdc20 Pfh1-depleted
gH2A WT
gH2A Pfh1-depleted
log2(Signal / Input)
2.5
2.0
1.5
1.0
0.5
0.0
−0.5
−1.0
−1.5
−2.0
-5000
-3000
-1000
1000
3000
5000
chromosome1:3705739-3715820

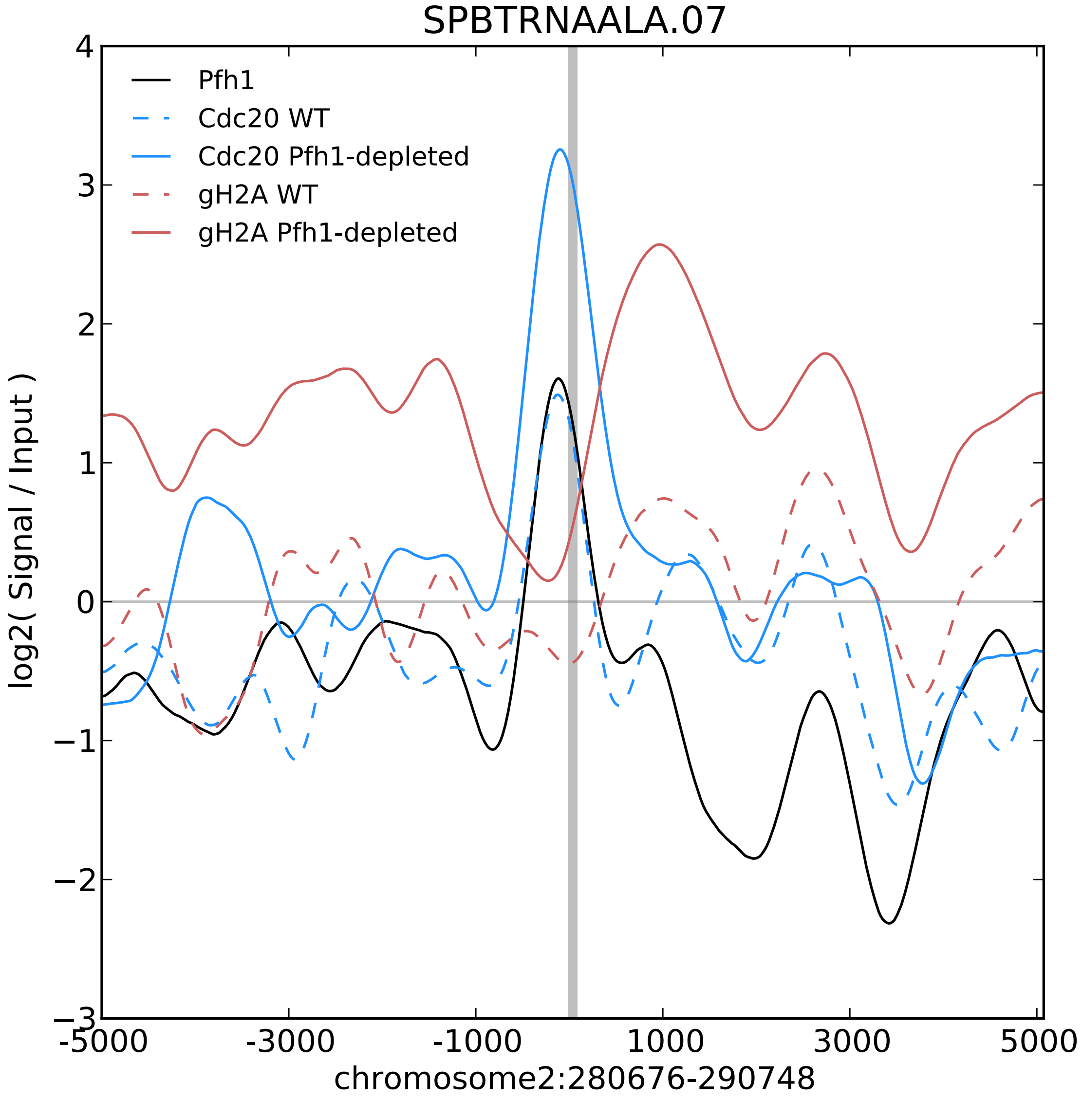
SPBTRNAALA.07
Pfh1
Cdc20 WT
Cdc20 Pfh1-depleted
gH2A WT
gH2A Pfh1-depleted
4
3
2
1
0
−1
−2
−3
log2(Signal / Input)
-5000
-3000
-1000
1000
3000
5000
chromosome2:280676-290748

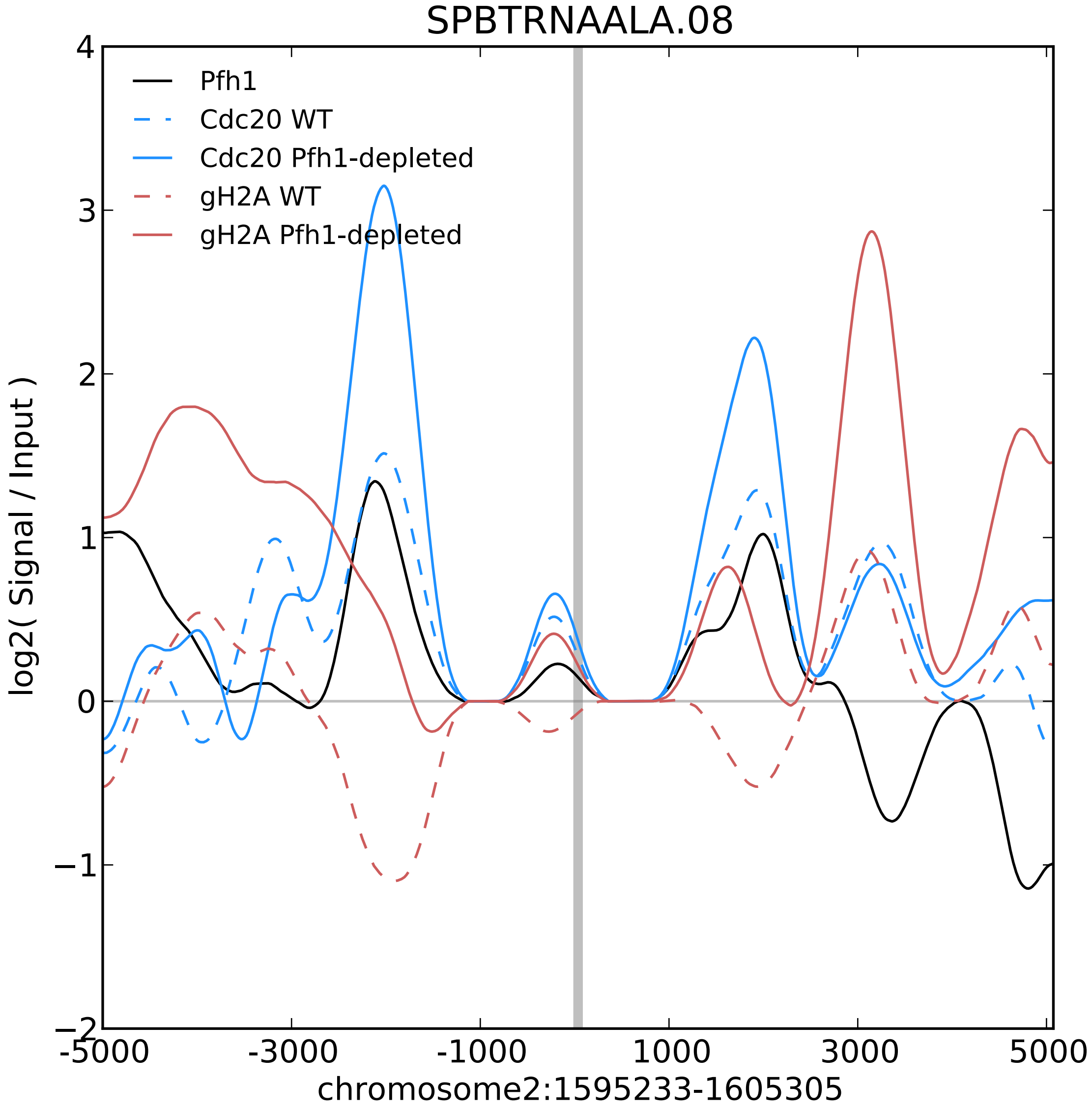
SPBTRNAALA.08
Pfh1
Cdc20 WT
Cdc20 Pfh1-depleted
gH2A WT
gH2A Pfh1-depleted
log2(Signal / Input)
4
3
2
1
0
−1
−2
-5000
-3000
-1000
1000
3000
5000
chromosome2:1595233-1605305

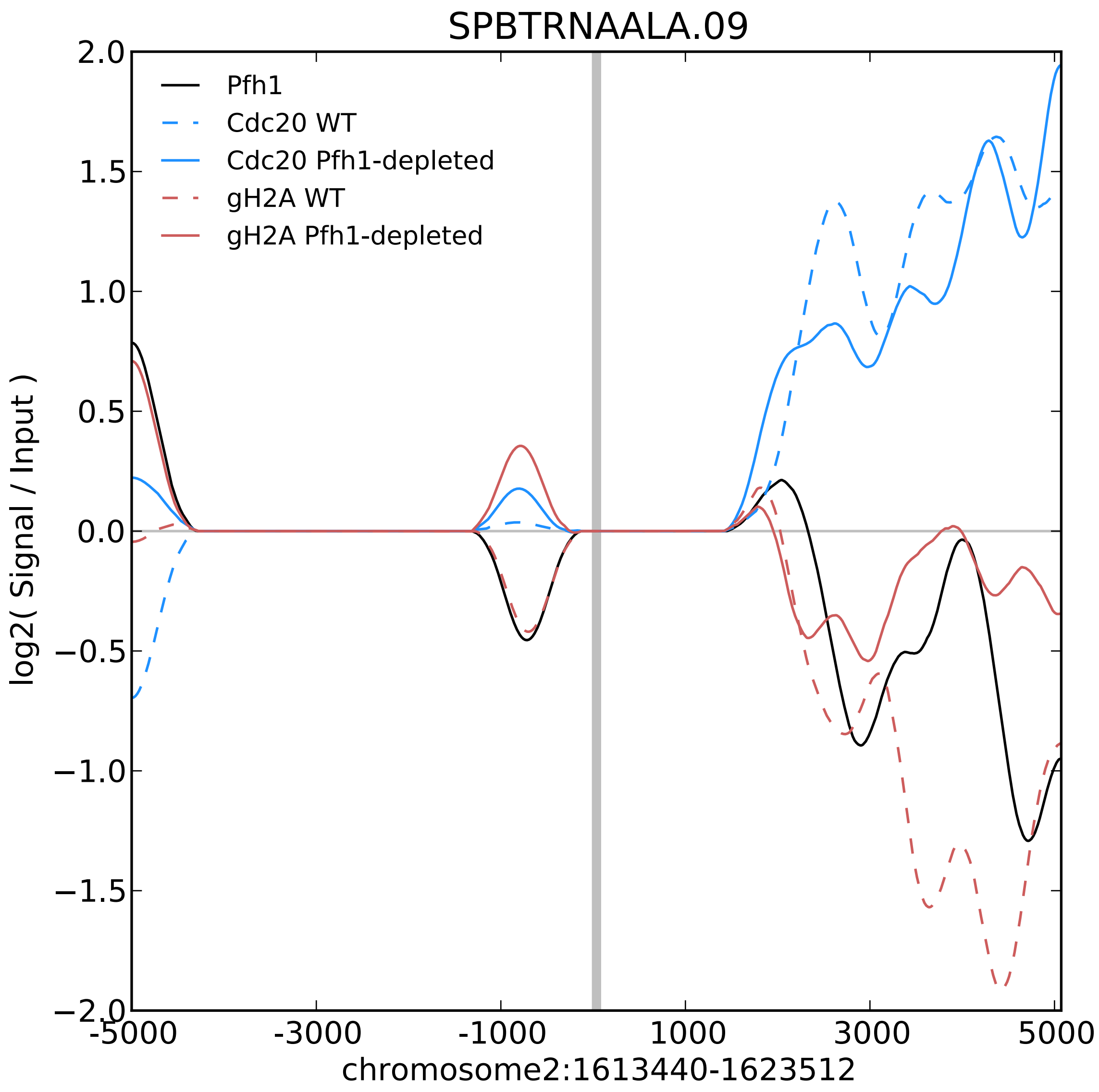

SPBTRNAALA.09
Pfh1
Cdc20 WT
Cdc20 Pfh1-depleted
gH2A WT
gH2A Pfh1-depleted
log2(Signal / Input)
2.0
1.5
1.0
0.5
0.0
−0.5
−1.0
−1.5
−2.0
-5000
-3000
-1000
1000
3000
5000
chromosome2:1613440-1623512

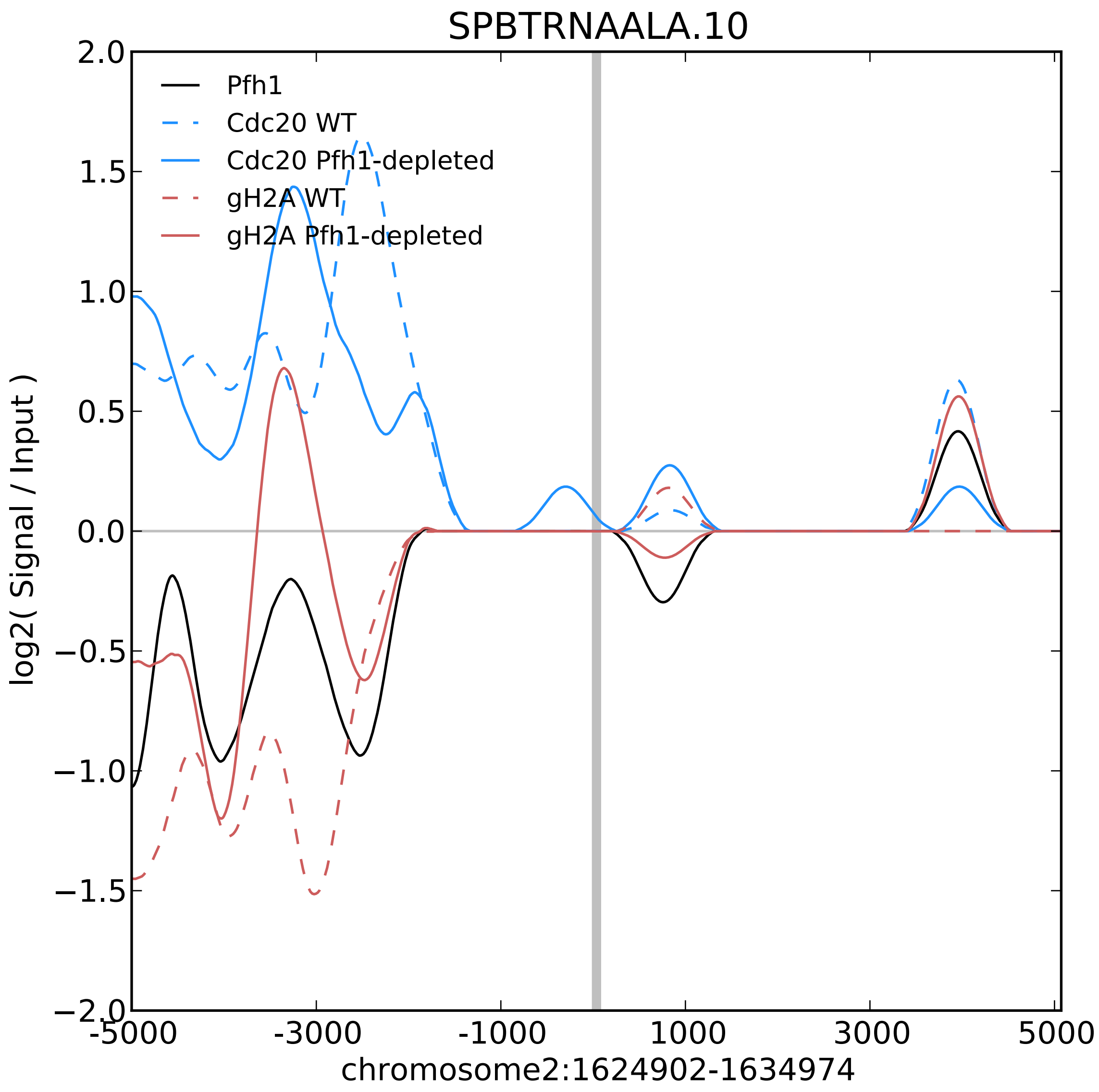

SPBTRNAALA.10
Pfh1
Cdc20 WT
Cdc20 Pfh1-depleted
gH2A WT
gH2A Pfh1-depleted
log2(Signal / Input)
2.0
1.5
1.0
0.5
0.0
−0.5
−1.0
−1.5
−2.0
-5000
-3000
-1000
1000
3000
5000
chromosome2:1624902-1634974

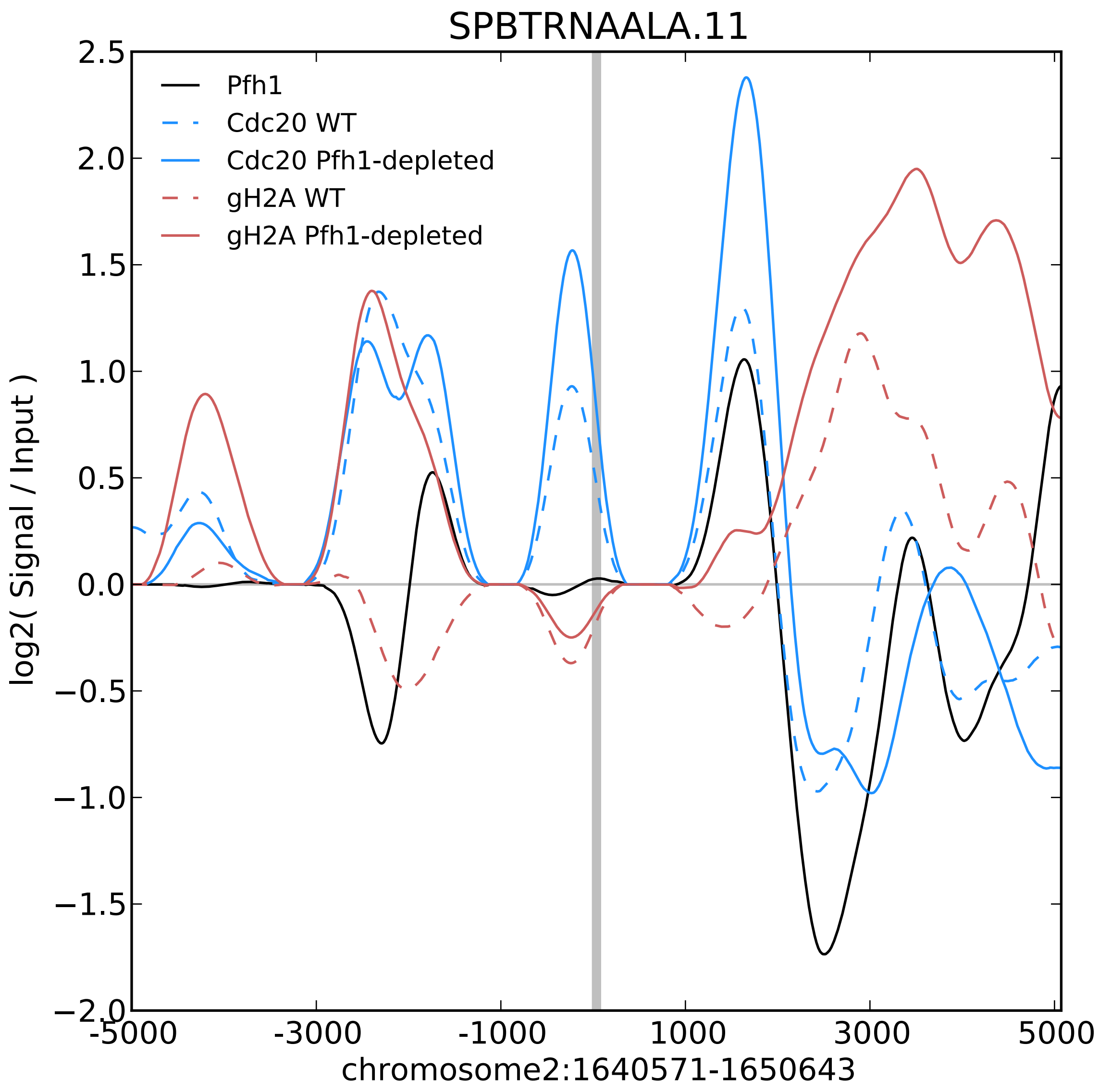

SPBTRNAALA.11
Pfh1
Cdc20 WT
Cdc20 Pfh1-depleted
gH2A WT
gH2A Pfh1-depleted
log2(Signal / Input)
2.5
2.0
1.5
1.0
0.5
0.0
−0.5
−1.0
−1.5
−2.0
-5000
-3000
-1000
1000
3000
5000
chromosome2:1640571-1650643

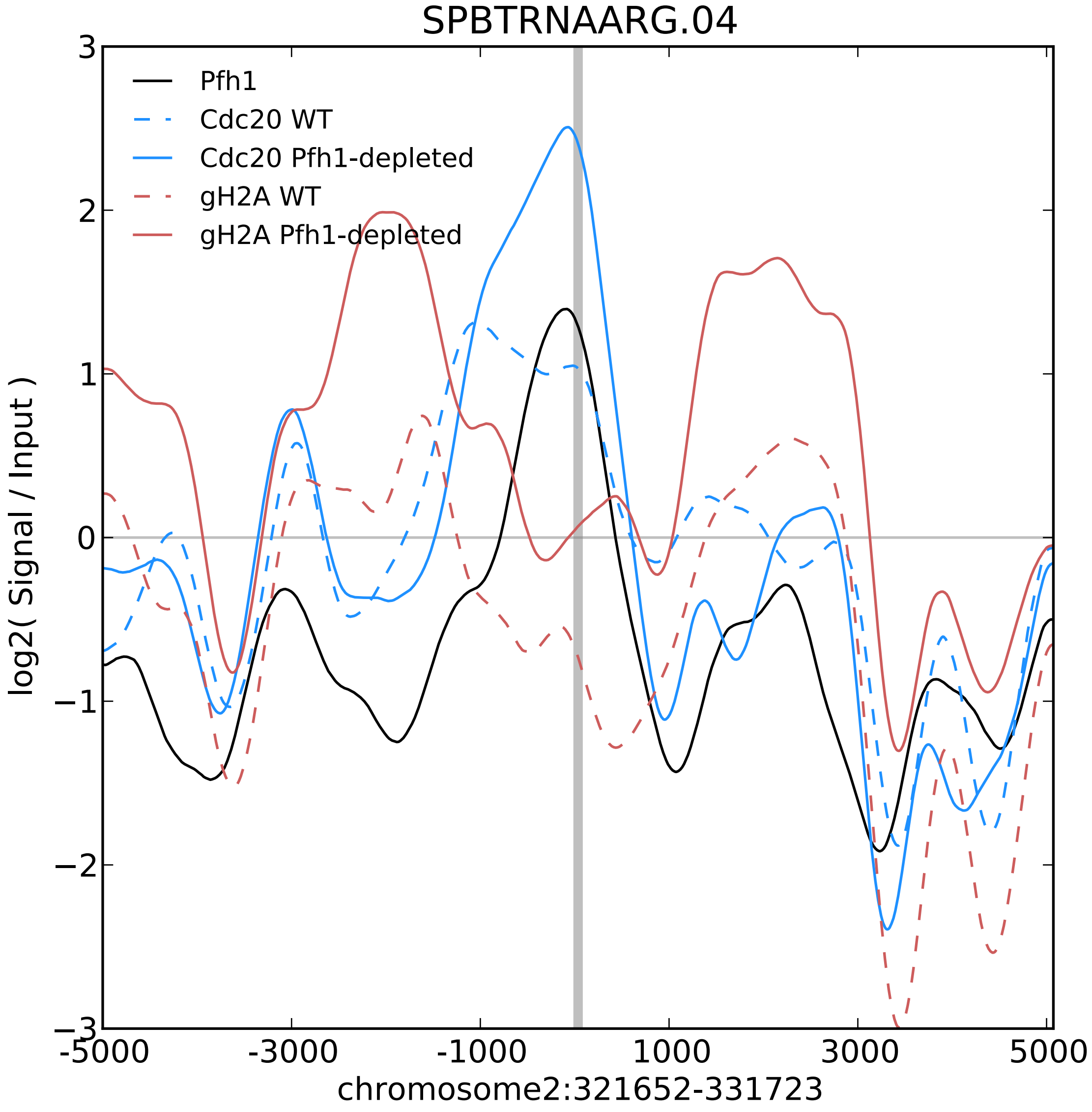

SPBTRNAARG.04
Pfh1
Cdc20 WT
Cdc20 Pfh1-depleted
gH2A WT
gH2A Pfh1-depleted
log2(Signal / Input)
3
2
1
0
−1
−2
−3
-5000
-3000
-1000
1000
3000
5000
chromosome2:321652-331723

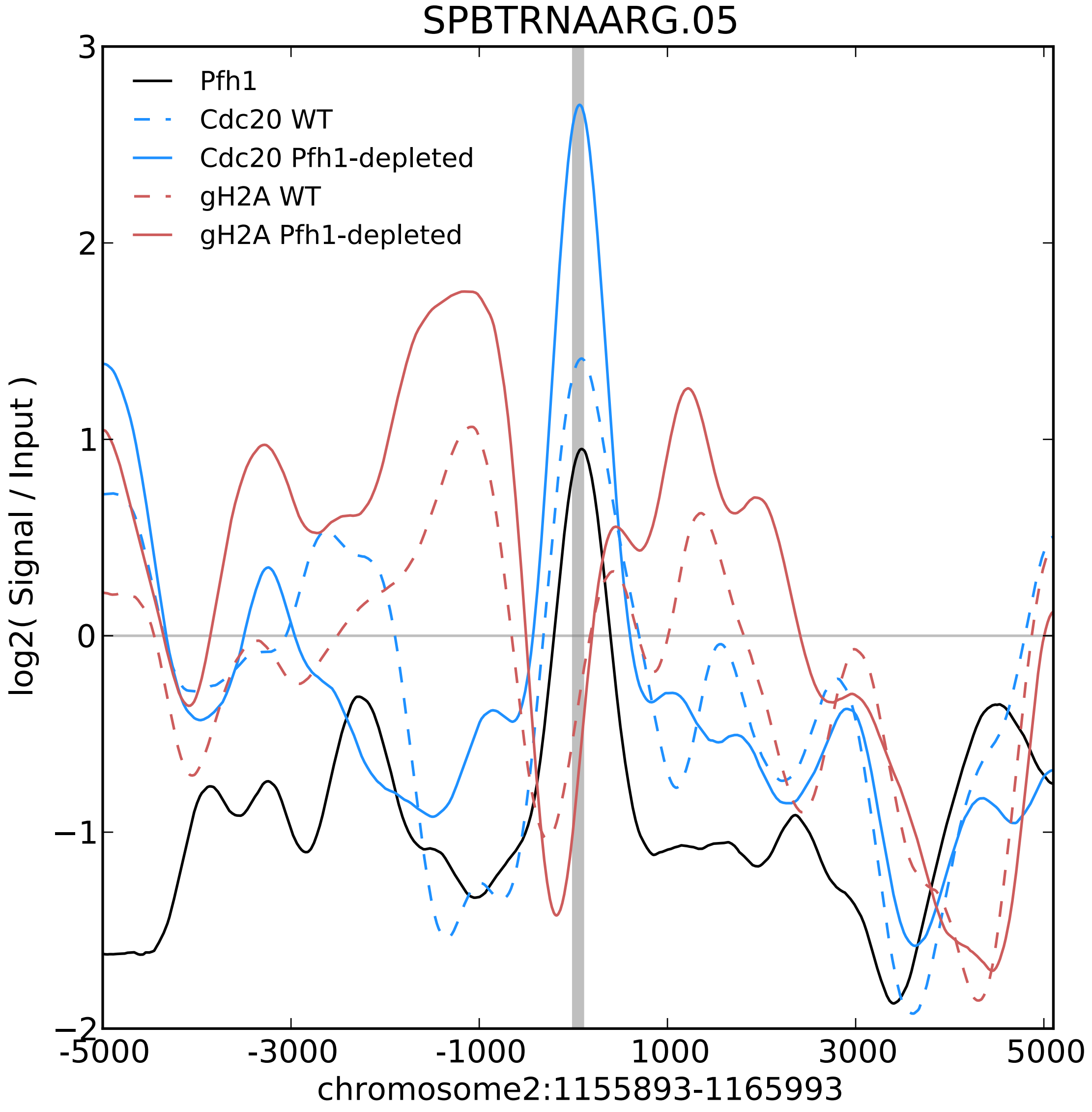
SPBTRNAARG.05
Pfh1
Cdc20 WT
Cdc20 Pfh1-depleted
gH2A WT
gH2A Pfh1-depleted
3
2
1
0
−1
−2
-5000
-3000
-1000
1000
3000
5000
log2(Signal / Input)
chromosome2:1155893-1165993

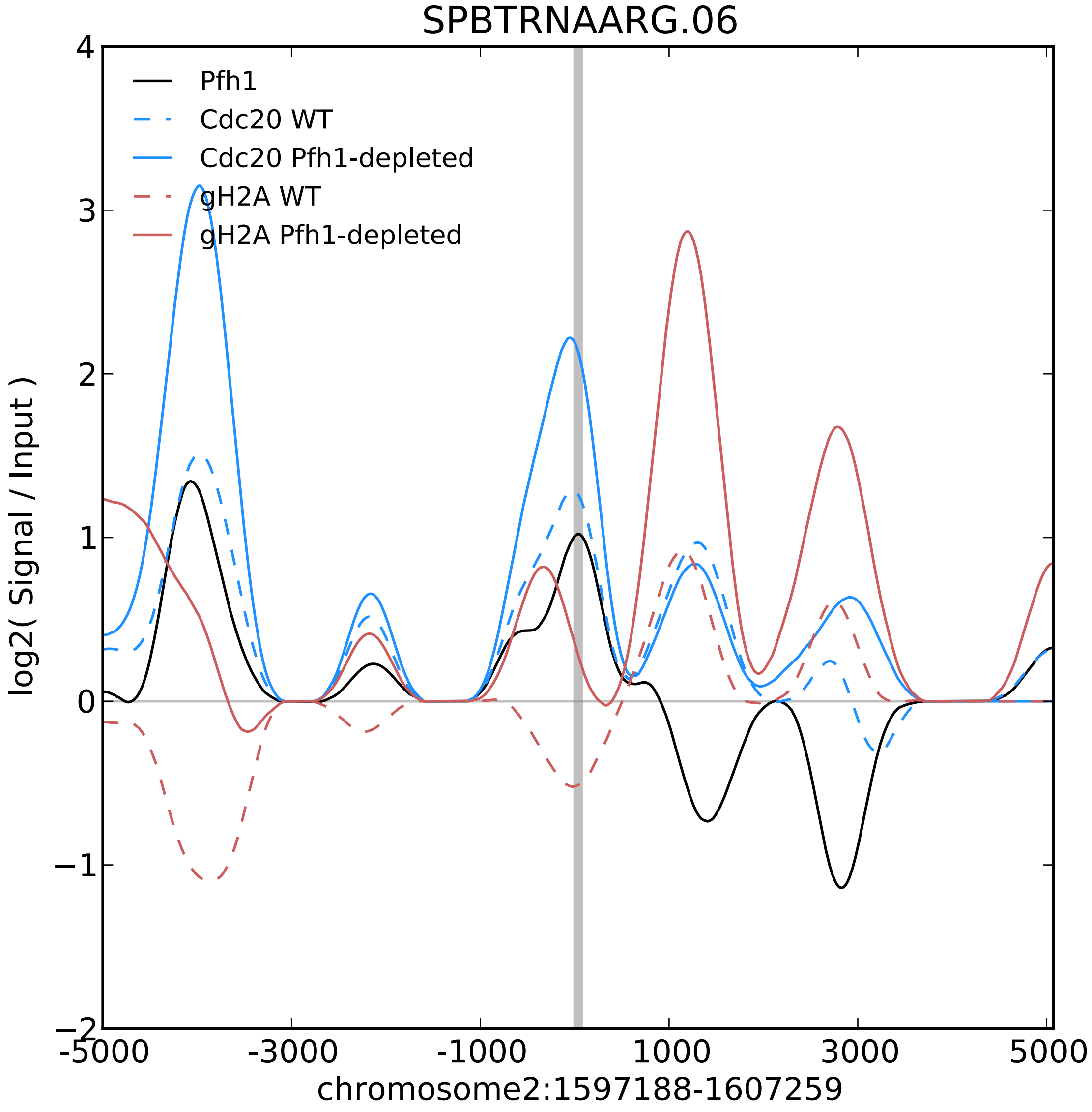

SPBTRNAARG.06
Pfh1
Cdc20 WT
Cdc20 Pfh1-depleted
gH2A WT
gH2A Pfh1-depleted
log2(Signal / Input)
4
3
2
1
0
−1
−2
-5000
-3000
-1000
1000
3000
5000
chromosome2:1597188-1607259

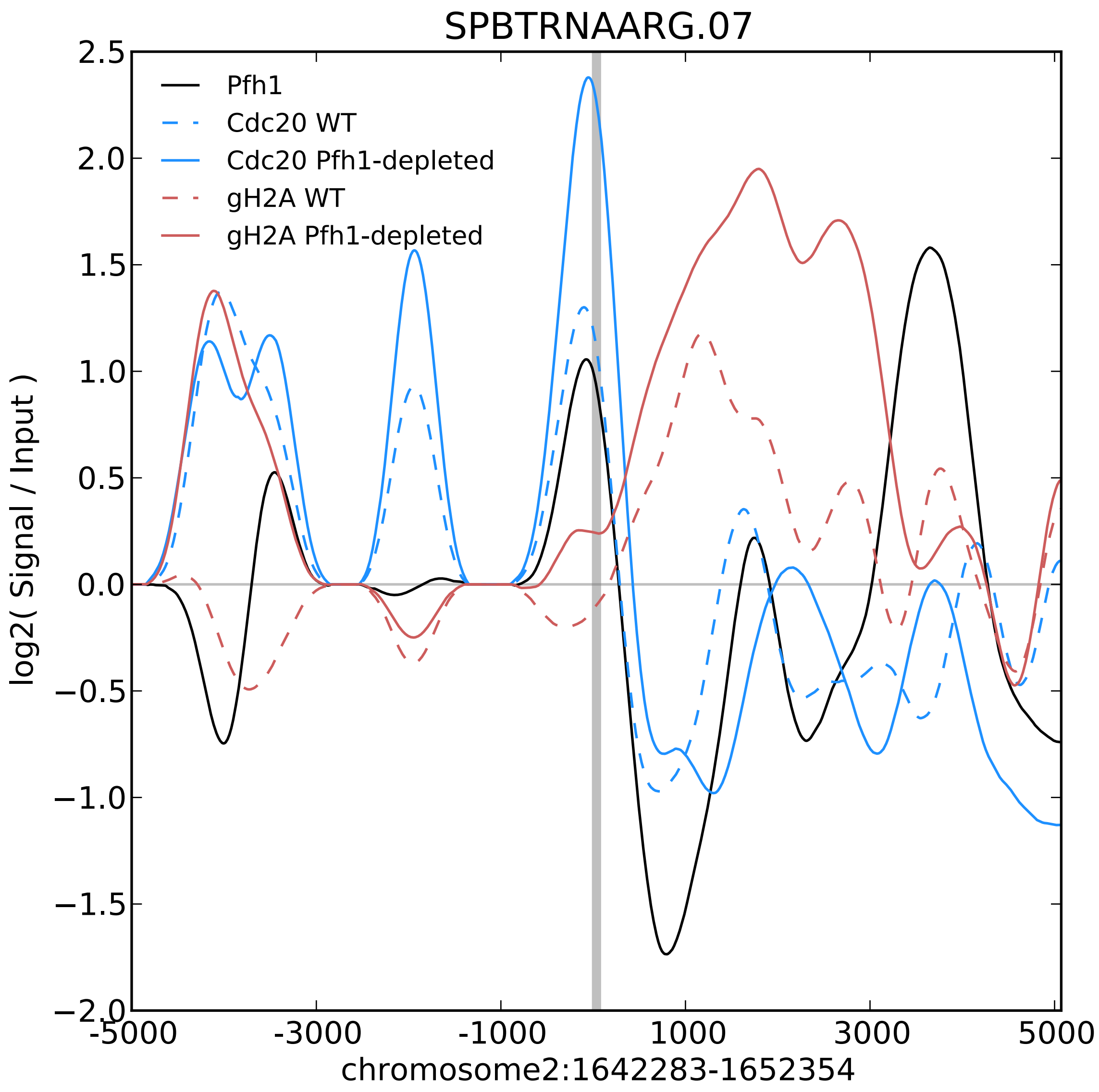
SPBTRNAARG.07
Pfh1
Cdc20 WT
Cdc20 Pfh1-depleted
gH2A WT
gH2A Pfh1-depleted
log2(Signal / Input)
2.5
2.0
1.5
1.0
0.5
0.0
−0.5
−1.0
−1.5
−2.0
-5000
-3000
-1000
1000
3000
5000
chromosome2:1642283-1652354

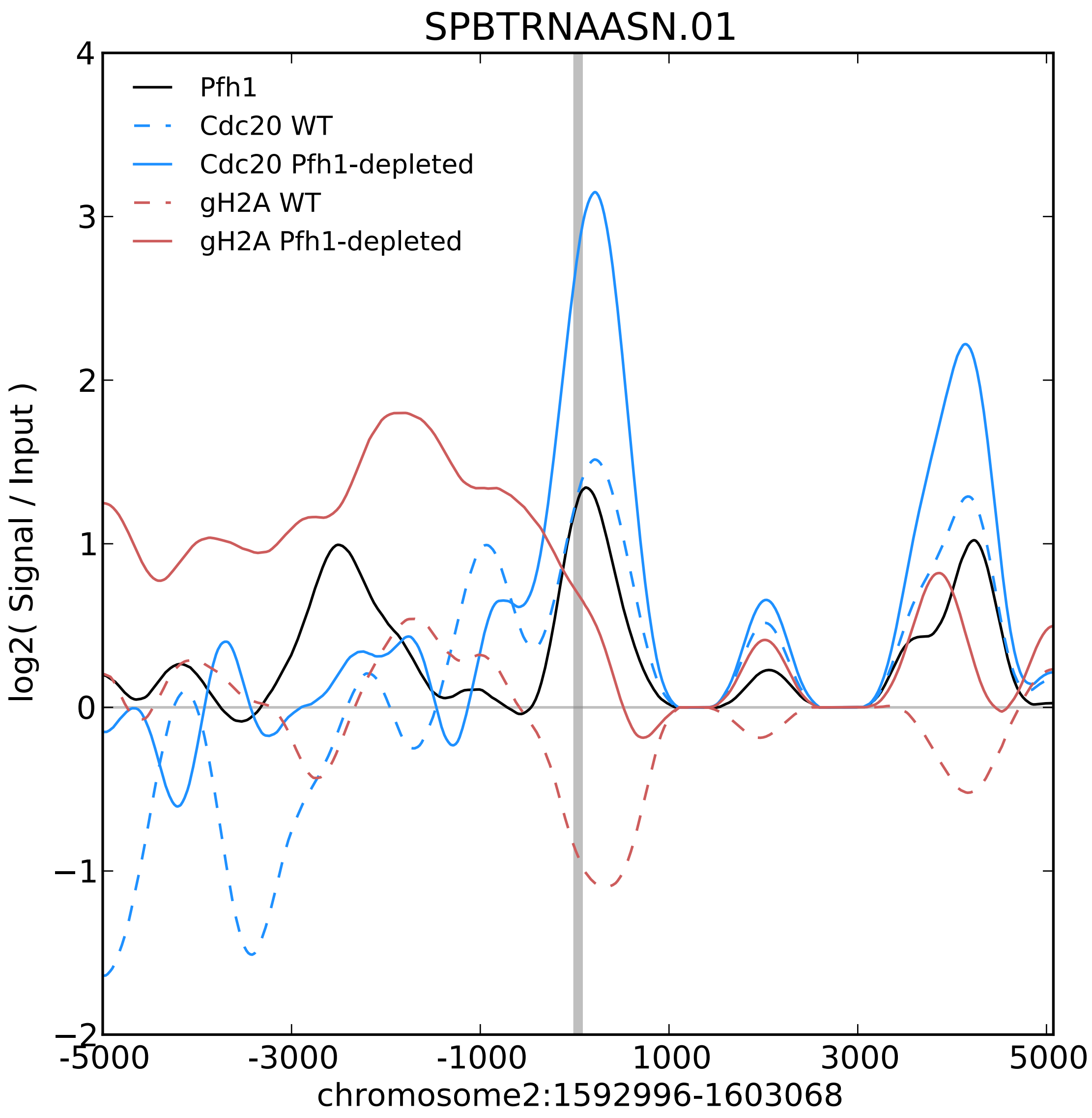

SPBTRNAASN.01
Pfh1
Cdc20 WT
Cdc20 Pfh1-depleted
gH2A WT
gH2A Pfh1-depleted
log2(Signal / Input)
4
3
2
1
0
−1
−2
-5000
-3000
-1000
1000
3000
5000
chromosome2:1592996-1603068

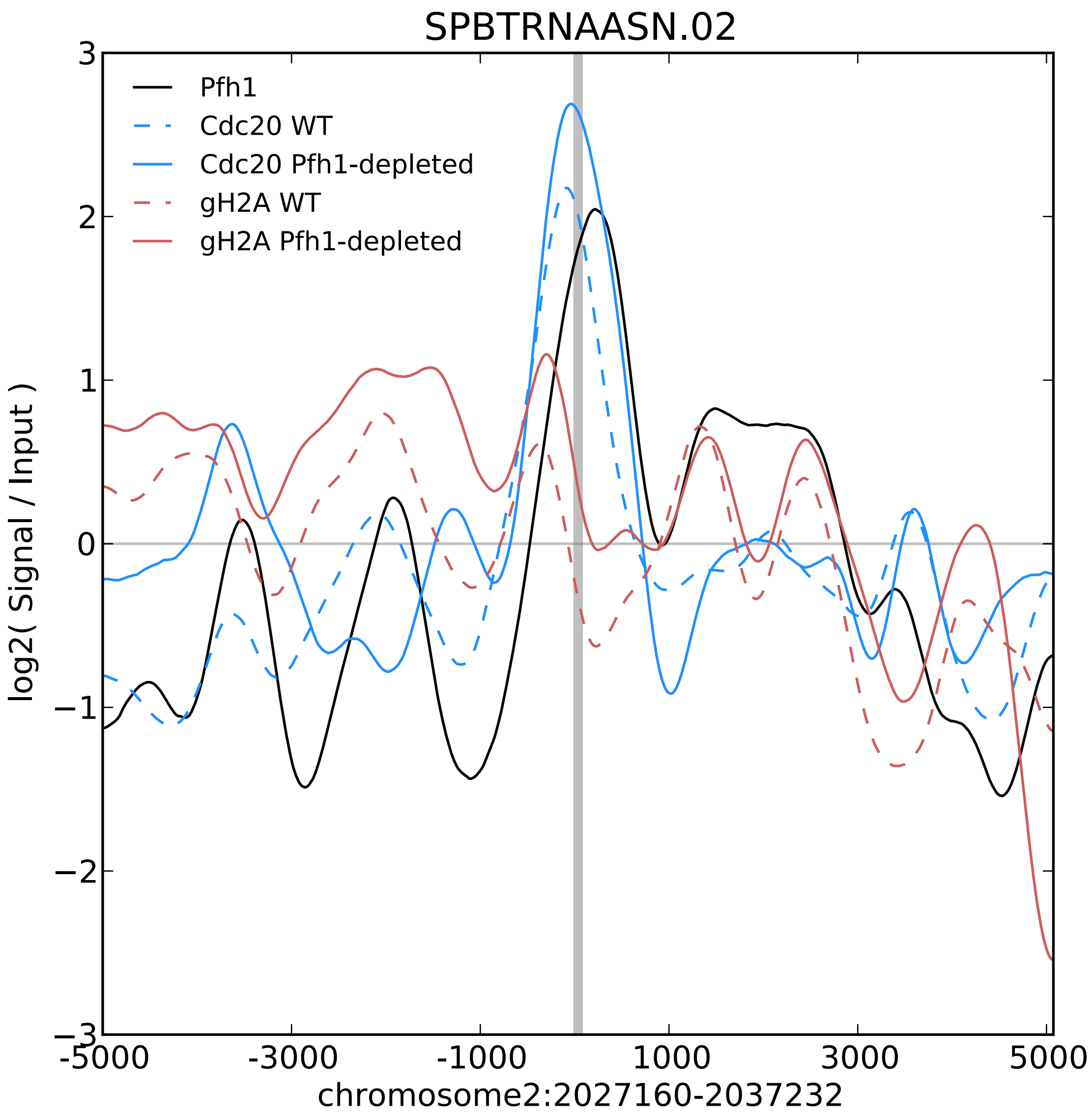
SPBTRNAASN.02
Pfh1
Cdc20 WT
Cdc20 Pfh1-depleted
gH2A WT
gH2A Pfh1-depleted
log2(Signal / Input)
3
2
1
0
−1
−2
−3
-5000
-3000
-1000
1000
3000
5000
chromosome2:2027160-2037232

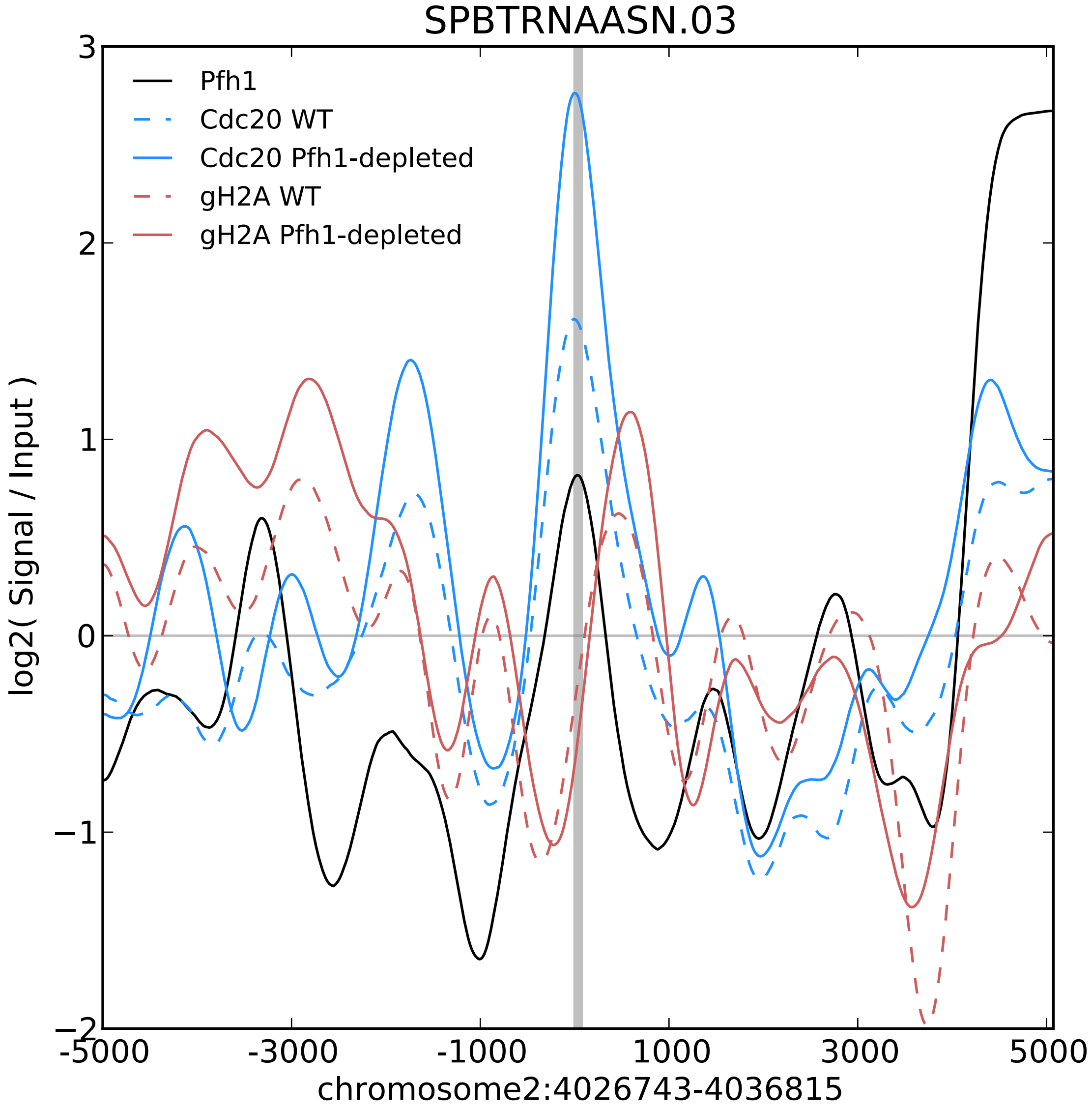
SPBTRNAASN.03
Pfh1
Cdc20 WT
Cdc20 Pfh1-depleted
gH2A WT
gH2A Pfh1-depleted
log2(Signal / Input)
3
2
1
0
−1
−2
-5000
-3000
-1000
1000
3000
5000
chromosome2:4026743-4036815

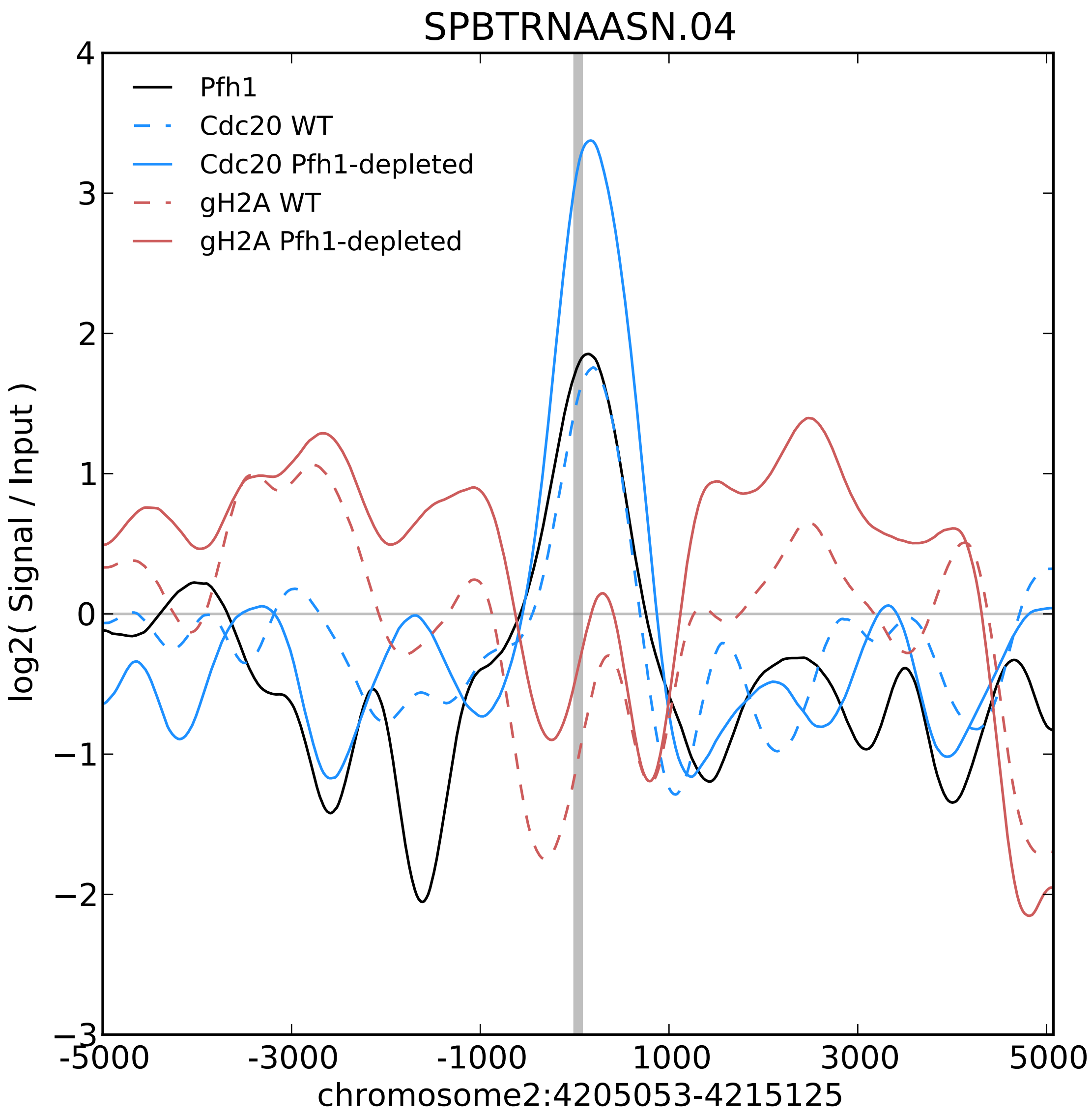

SPBTRNAASN.04
Pfh1
Cdc20 WT
Cdc20 Pfh1-depleted
gH2A WT
gH2A Pfh1-depleted
log2(Signal / Input)
4
3
2
1
0
−1
−2
−3
-5000
-3000
-1000
1000
3000
5000
chromosome2:4205053-4215125

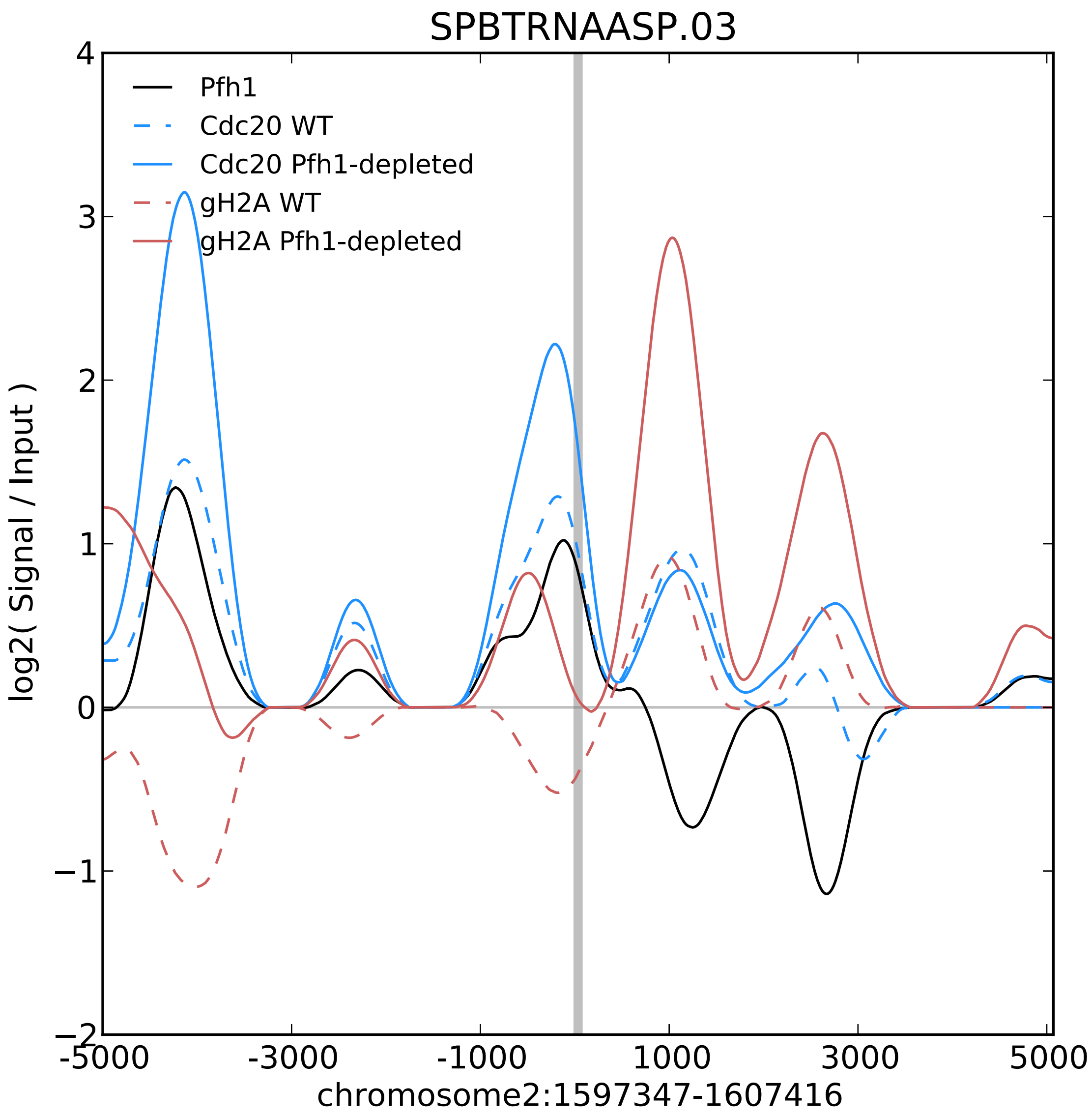

SPBTRNAASP.03
Pfh1
Cdc20 WT
Cdc20 Pfh1-depleted
gH2A WT
gH2A Pfh1-depleted
log2(Signal / Input)
4
3
2
1
0
−1
−2
-5000
-3000
-1000
1000
3000
5000
chromosome2:1597347-1607416

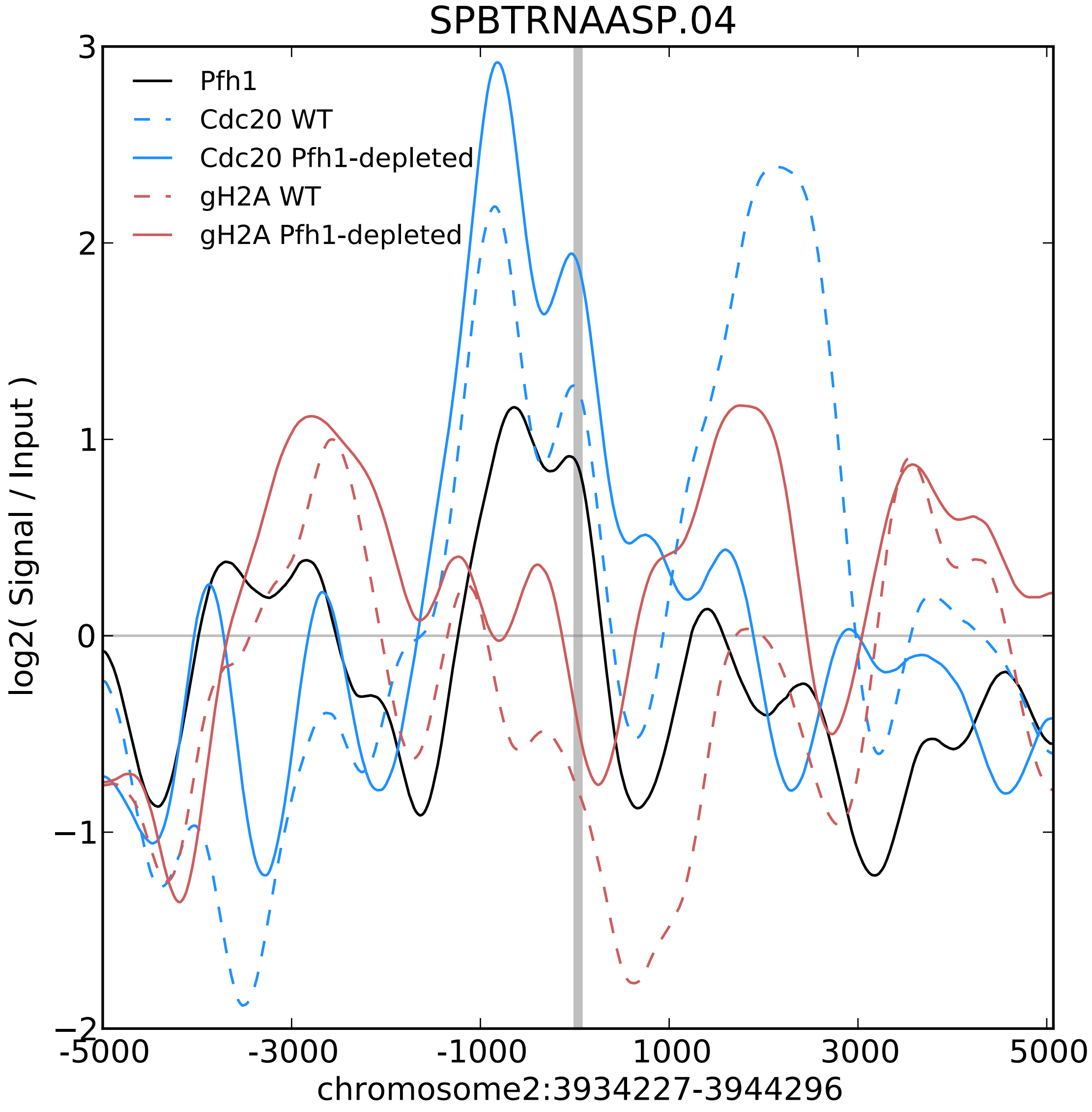

SPBTRNAASP.04
Pfh1
Cdc20 WT
Cdc20 Pfh1-depleted
gH2A WT
gH2A Pfh1-depleted
3
2
1
0
−1
−2
log2(Signal / Input)
-5000
-3000
-1000
1000
3000
5000
chromosome2:3934227-3944296

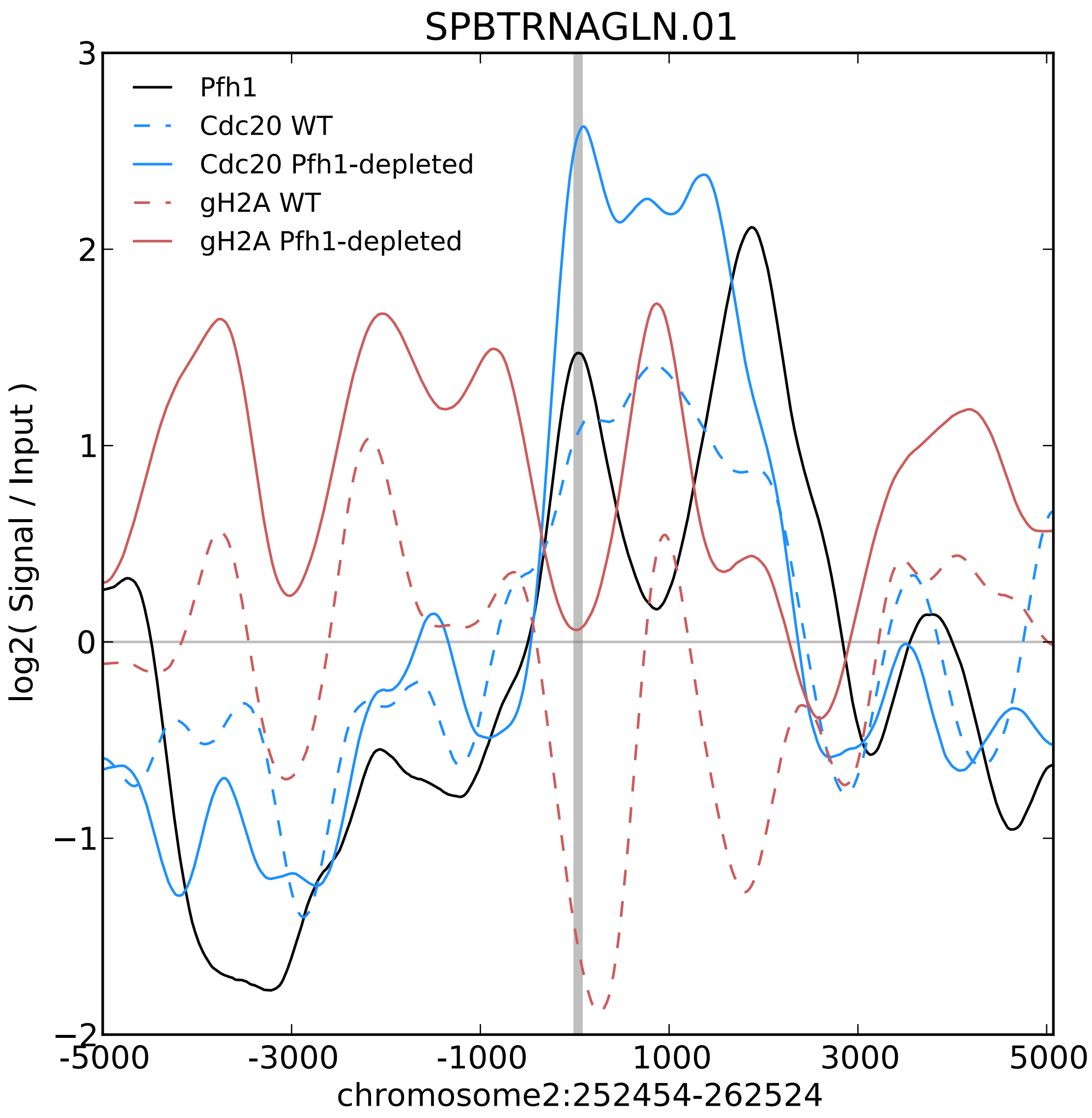
SPBTRNAGLN.01
Pfh1
Cdc20 WT
Cdc20 Pfh1-depleted
gH2A WT
gH2A Pfh1-depleted
3
2
1
0
−1
−2
log2(Signal / Input)
-5000
-3000
-1000
1000
3000
5000
chromosome2:252454-262524

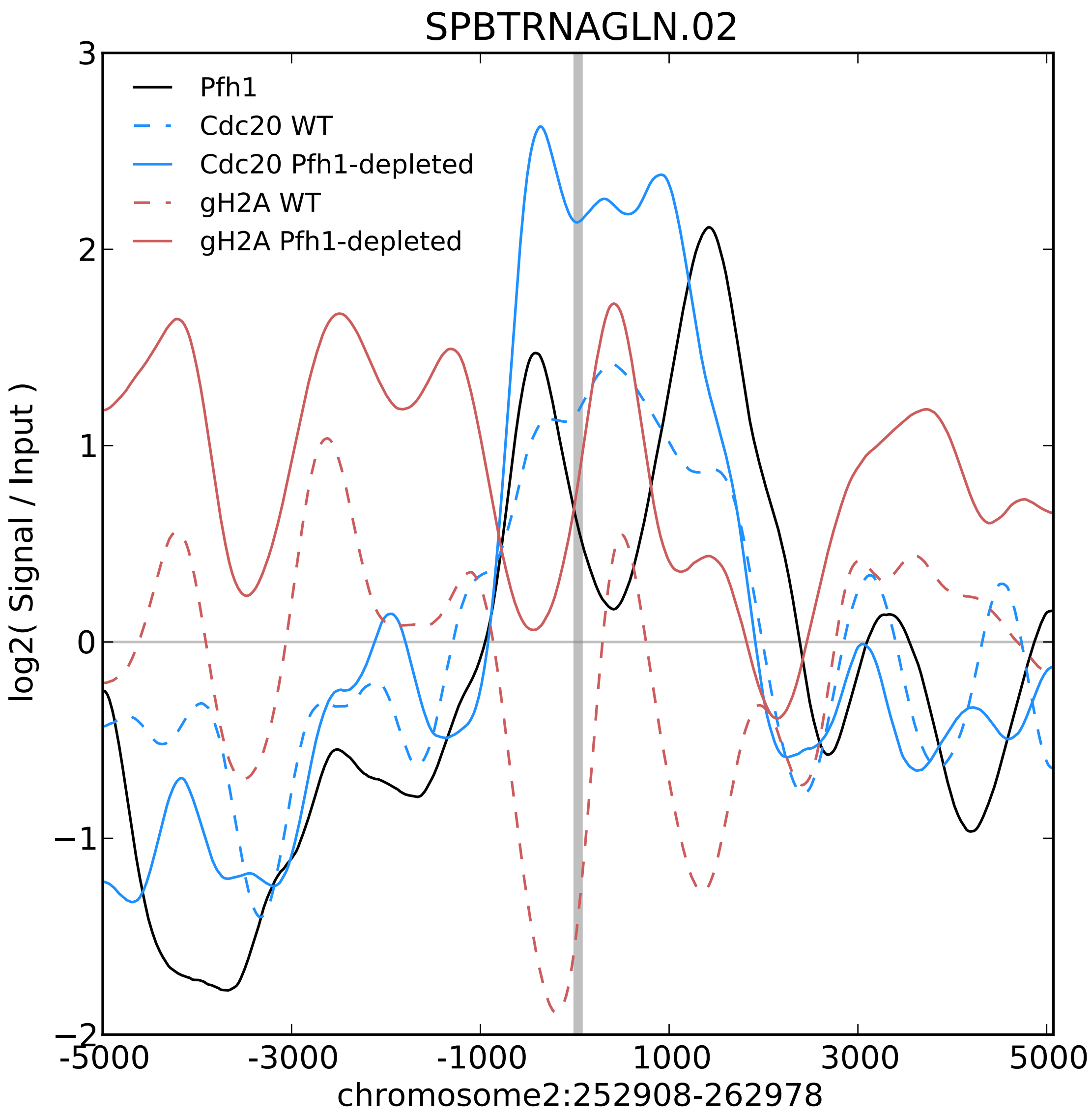

SPBTRNAGLN.02
Pfh1
Cdc20 WT
Cdc20 Pfh1-depleted
gH2A WT
gH2A Pfh1-depleted
log2(Signal / Input)
3
2
1
0
−1
−2
-5000
-3000
-1000
1000
3000
5000
chromosome2:252908-262978

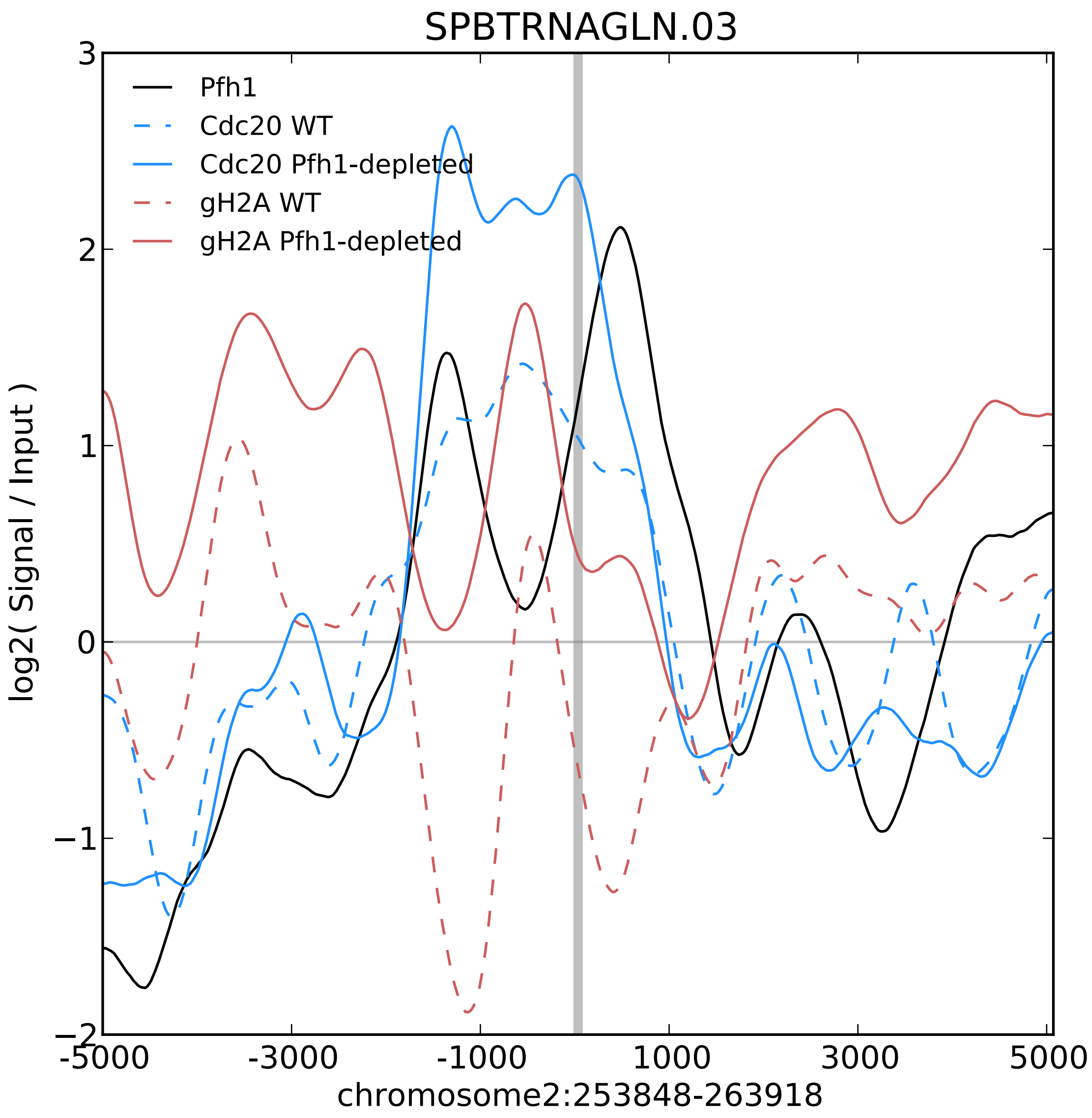
SPBTRNAGLN.03
Pfh1
Cdc20 WT
Cdc20 Pfh1-depleted
gH2A WT
gH2A Pfh1-depleted
log2(Signal / Input)
3
2
1
0
−1
−2
-5000
-3000
-1000
1000
3000
5000
chromosome2:253848-263918

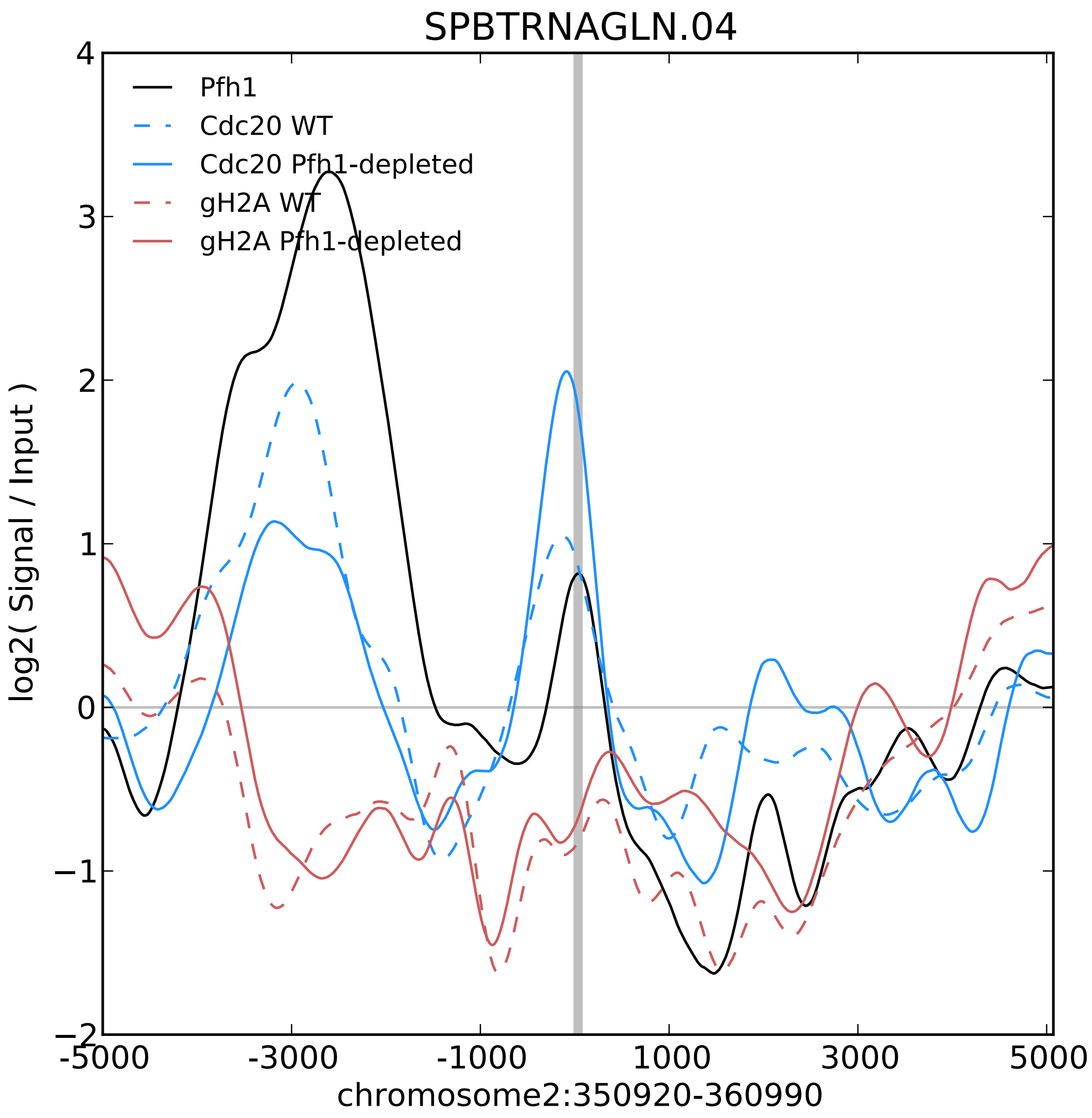
SPBTRNAGLN.04
Pfh1
Cdc20 WT
Cdc20 Pfh1-depleted
gH2A WT
gH2A Pfh1-depleted
log2(Signal / Input)
4
3
2
1
0
−1
−2
-5000
-3000
-1000
1000
3000
5000
chromosome2:350920-360990

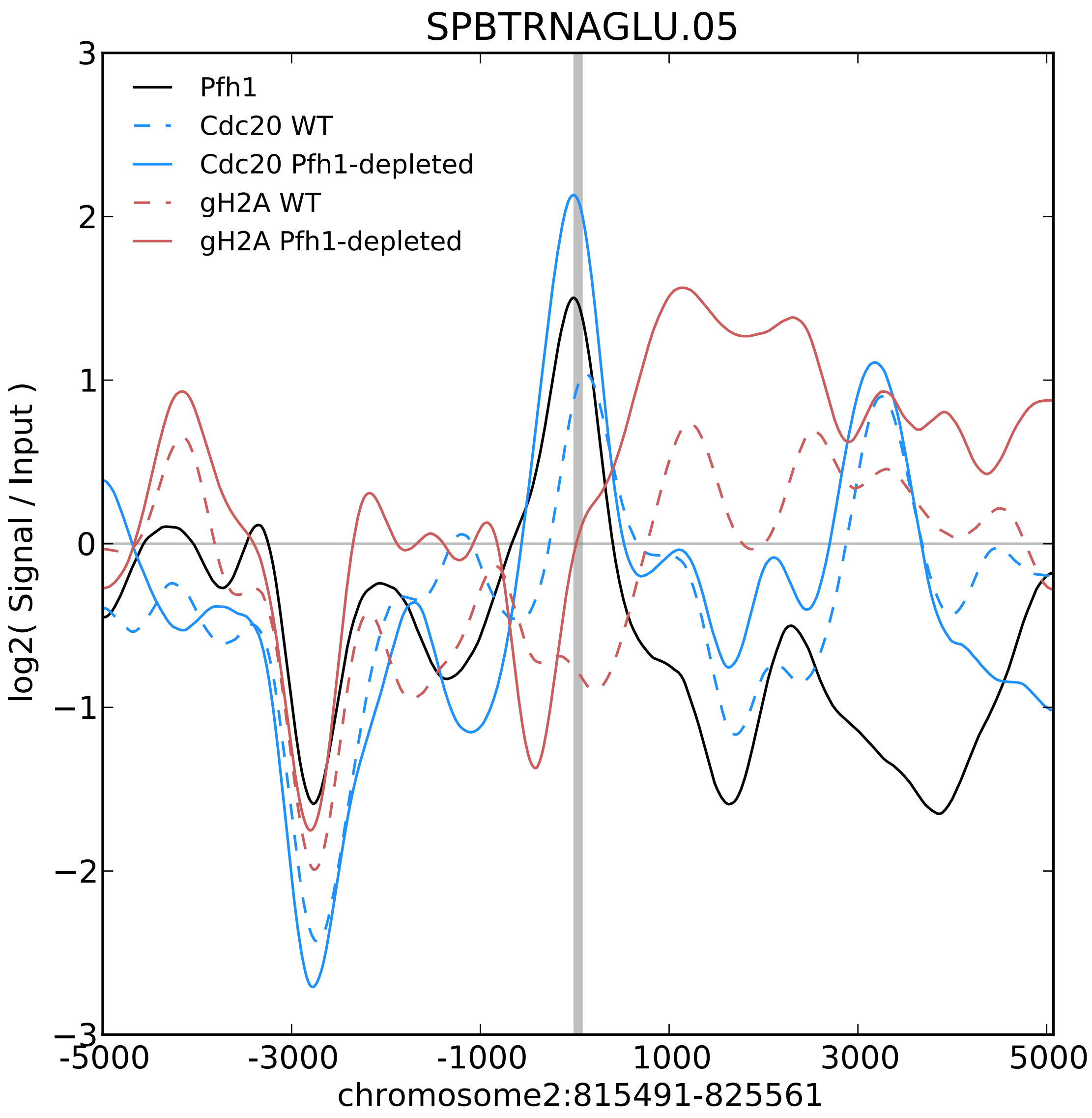
SPBTRNAGLU.05
Pfh1
Cdc20 WT
Cdc20 Pfh1-depleted
gH2A WT
gH2A Pfh1-depleted
log2(Signal / Input)
3
2
1
0
−1
−2
−3
-5000
-3000
-1000
1000
3000
5000
chromosome2:815491-825561

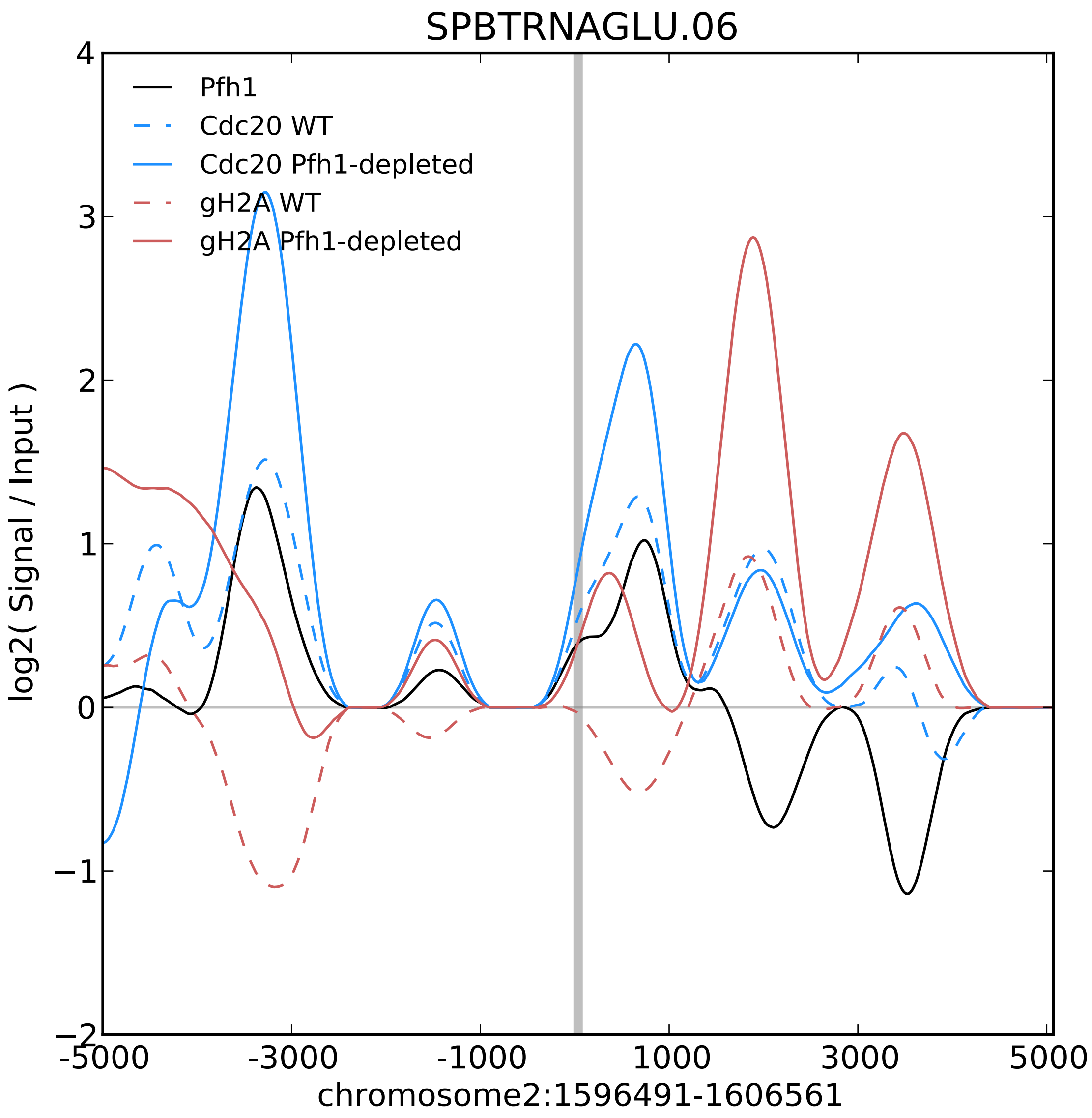
SPBTRNAGLU.06
Pfh1
Cdc20 WT
Cdc20 Pfh1-depleted
gH2A WT
gH2A Pfh1-depleted
log2(Signal / Input)
4
3
2
1
0
−1
−2
-5000
-3000
-1000
1000
3000
5000
chromosome2:1596491-1606561

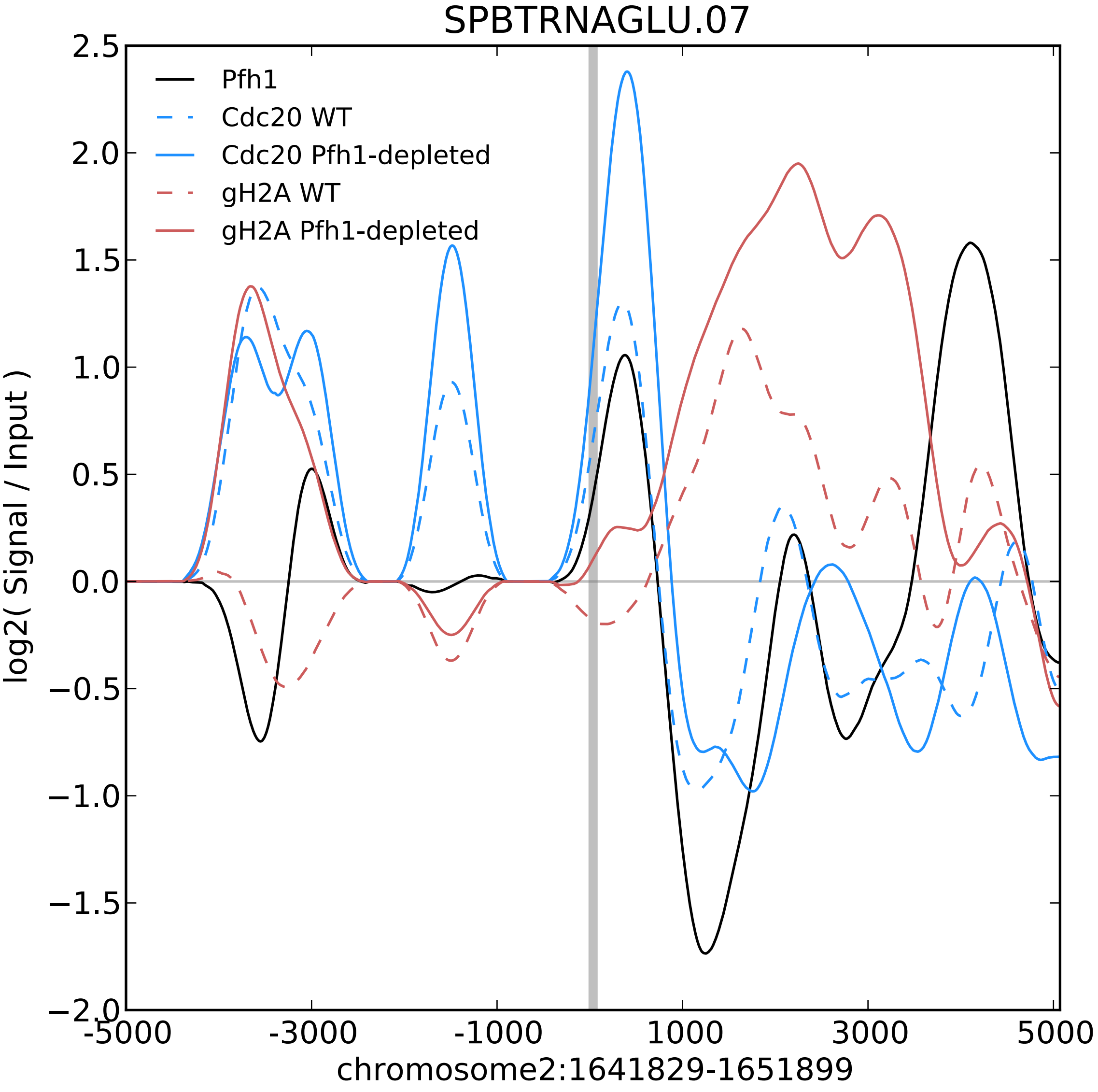
SPBTRNAGLU.07
Pfh1
Cdc20 WT
Cdc20 Pfh1-depleted
gH2A WT
gH2A Pfh1-depleted
log2(Signal / Input)
2.5
2.0
1.5
1.0
0.5
0.0
−0.5
−1.0
−1.5
−2.0
-5000
-3000
-1000
1000
3000
5000
chromosome2:1641829-1651899

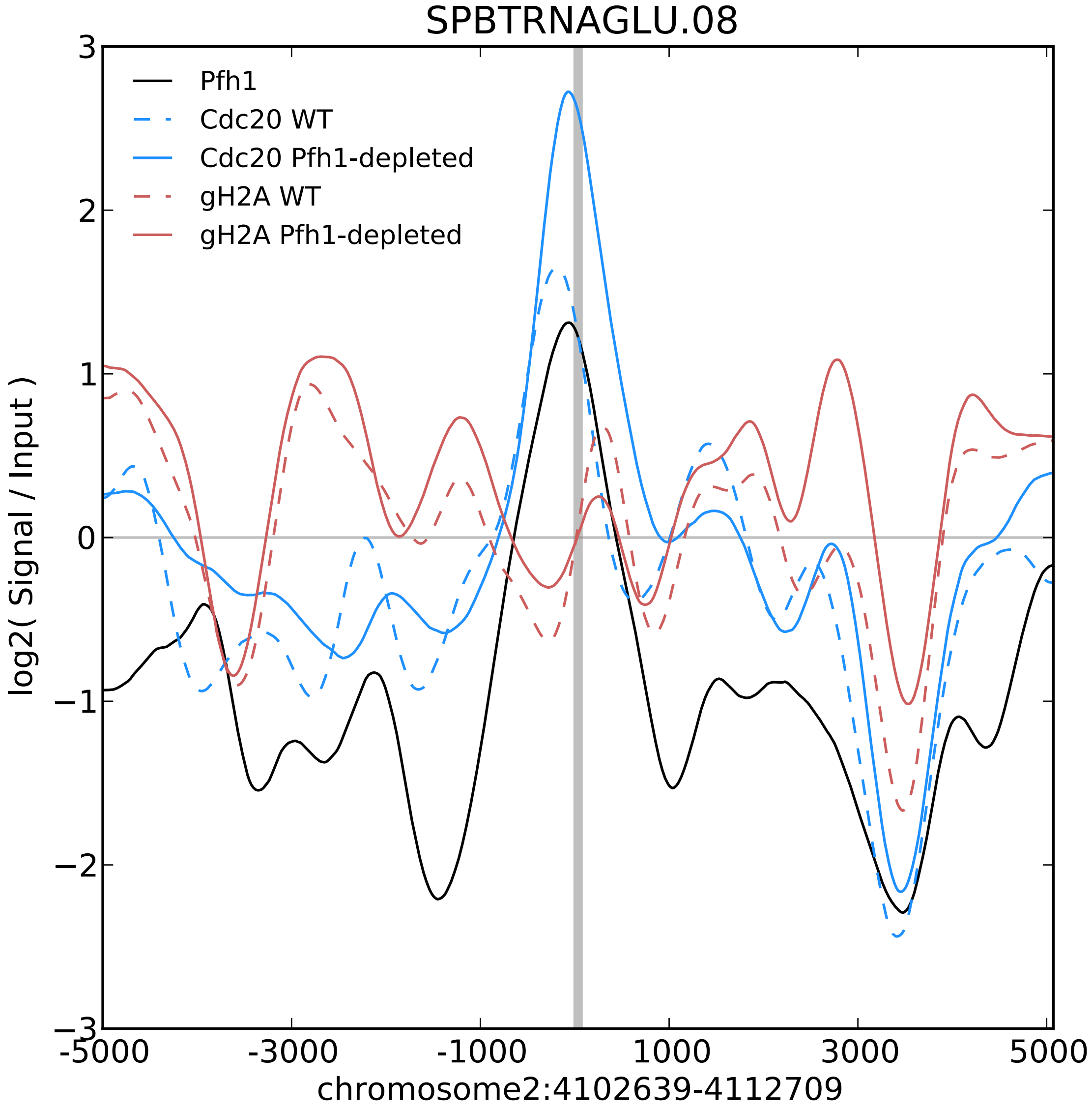
SPBTRNAGLU.08
Pfh1
Cdc20 WT
Cdc20 Pfh1-depleted
gH2A WT
gH2A Pfh1-depleted
log2(Signal / Input)
3
2
1
0
−1
−2
−3
-5000
-3000
-1000
1000
3000
5000
chromosome2:4102639-4112709

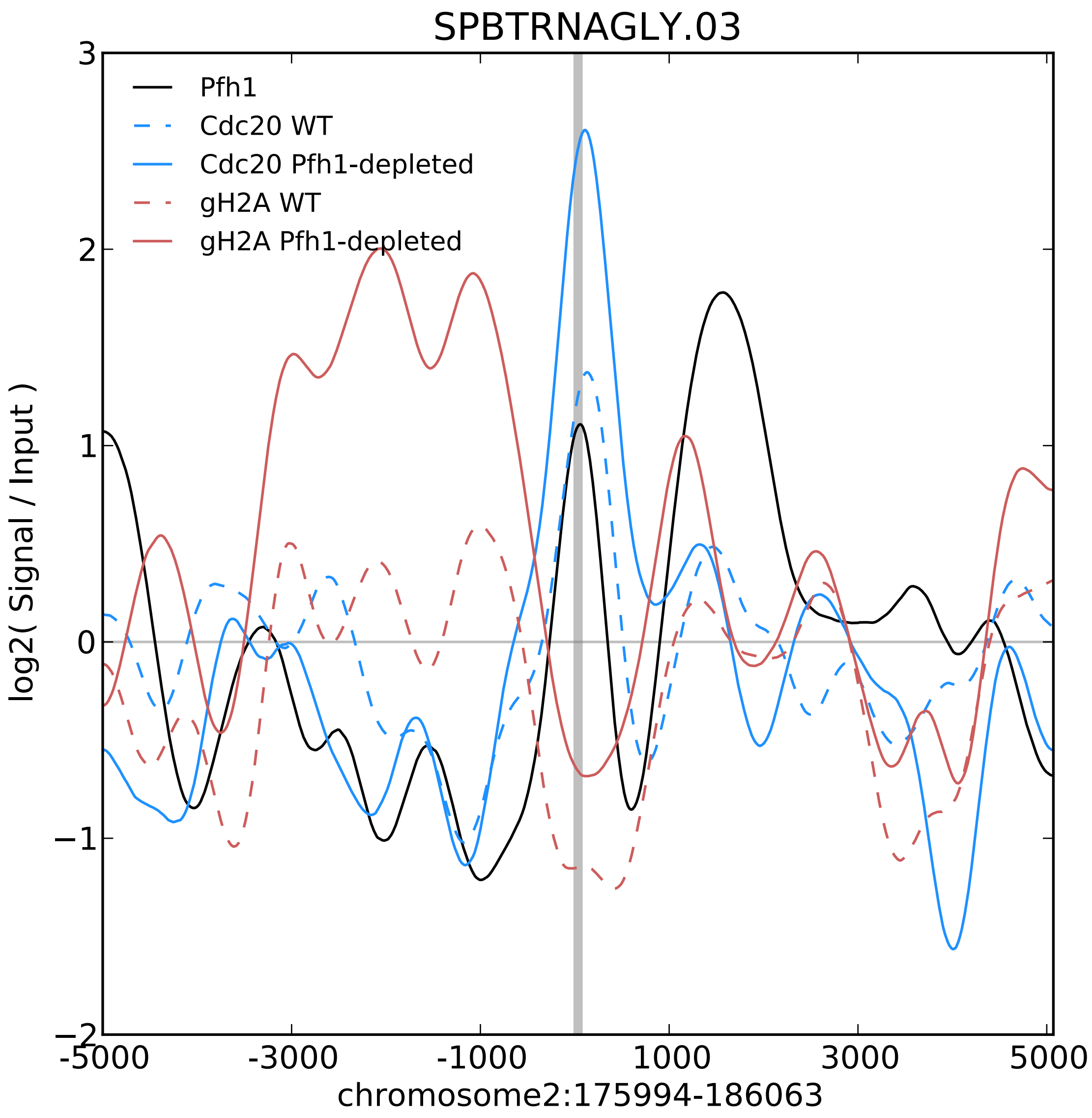

SPBTRNAGLY.03
Pfh1
Cdc20 WT
Cdc20 Pfh1-depleted
gH2A WT
gH2A Pfh1-depleted
log2(Signal / Input)
3
2
1
0
−1
−2
-5000
-3000
-1000
1000
3000
5000
chromosome2:175994-186063

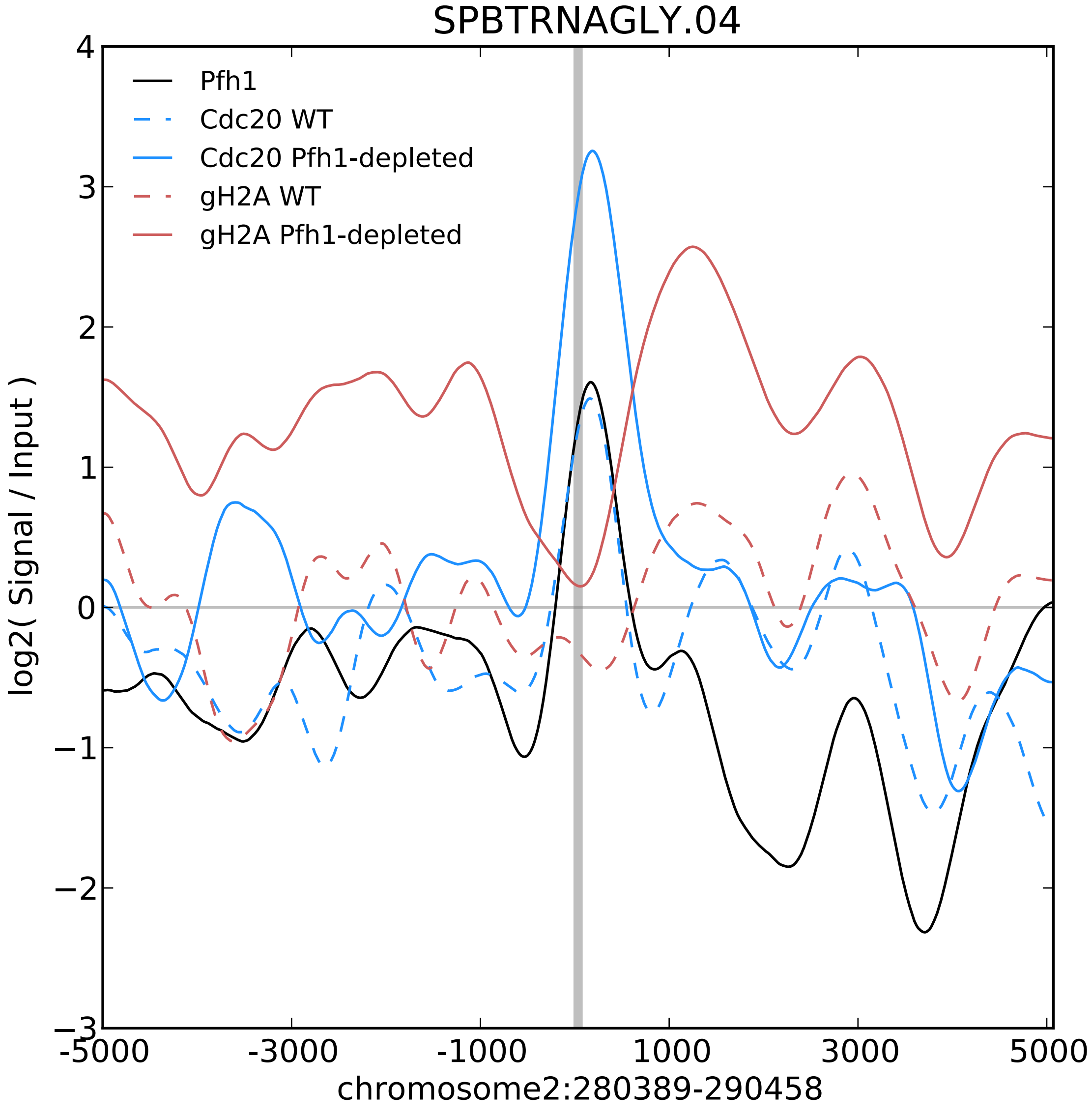

SPBTRNAGLY.04
Pfh1
Cdc20 WT
Cdc20 Pfh1-depleted
gH2A WT
gH2A Pfh1-depleted
log2(Signal / Input)
4
3
2
1
0
−1
−2
−3
-5000
-3000
-1000
1000
3000
5000
chromosome2:280389-290458

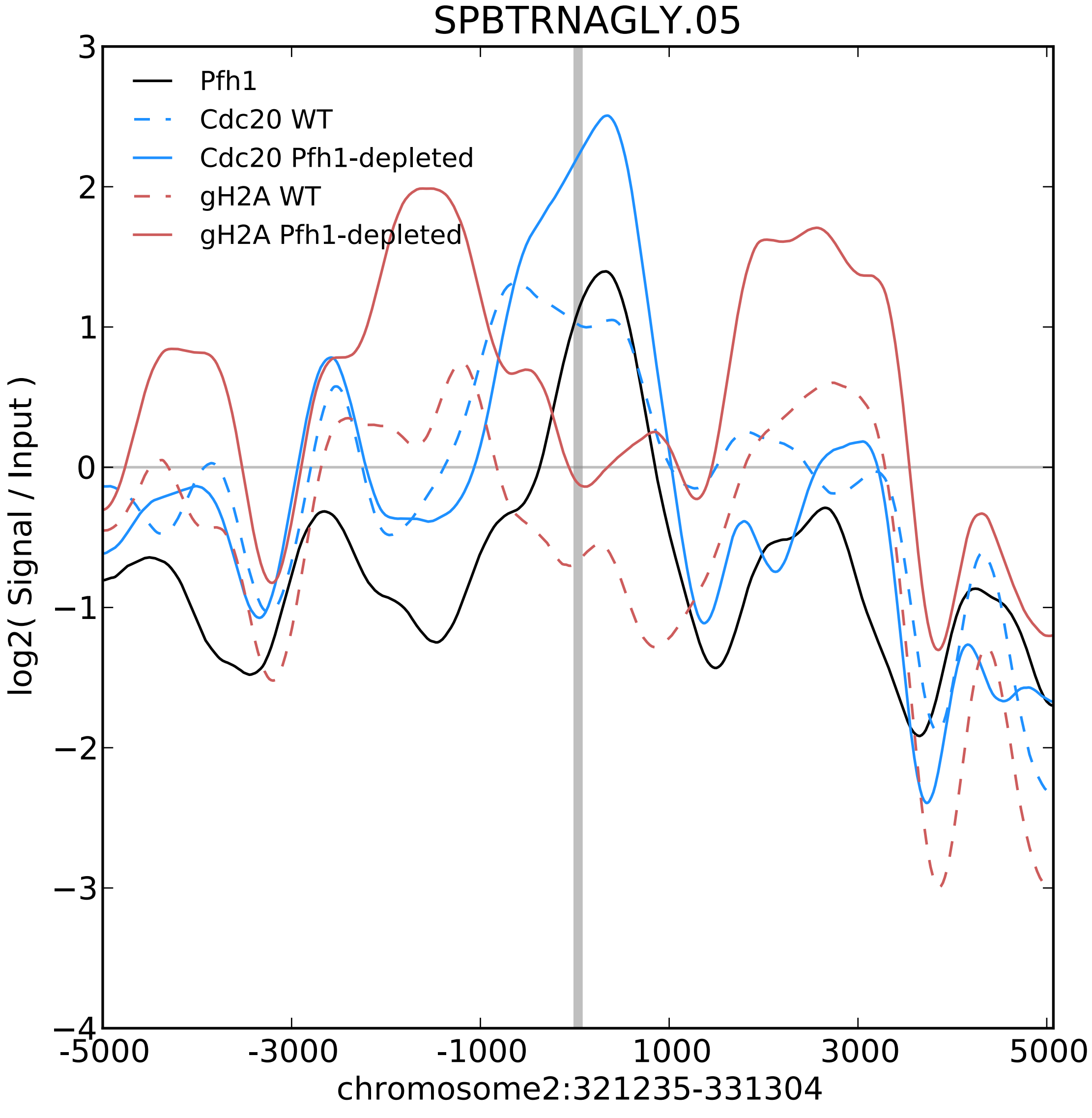

SPBTRNAGLY.05
Pfh1
Cdc20 WT
Cdc20 Pfh1-depleted
gH2A WT
gH2A Pfh1-depleted
3
2
1
0
−1
−2
−3
−4
log2(Signal / Input)
-5000
-3000
-1000
1000
3000
5000
chromosome2:321235-331304

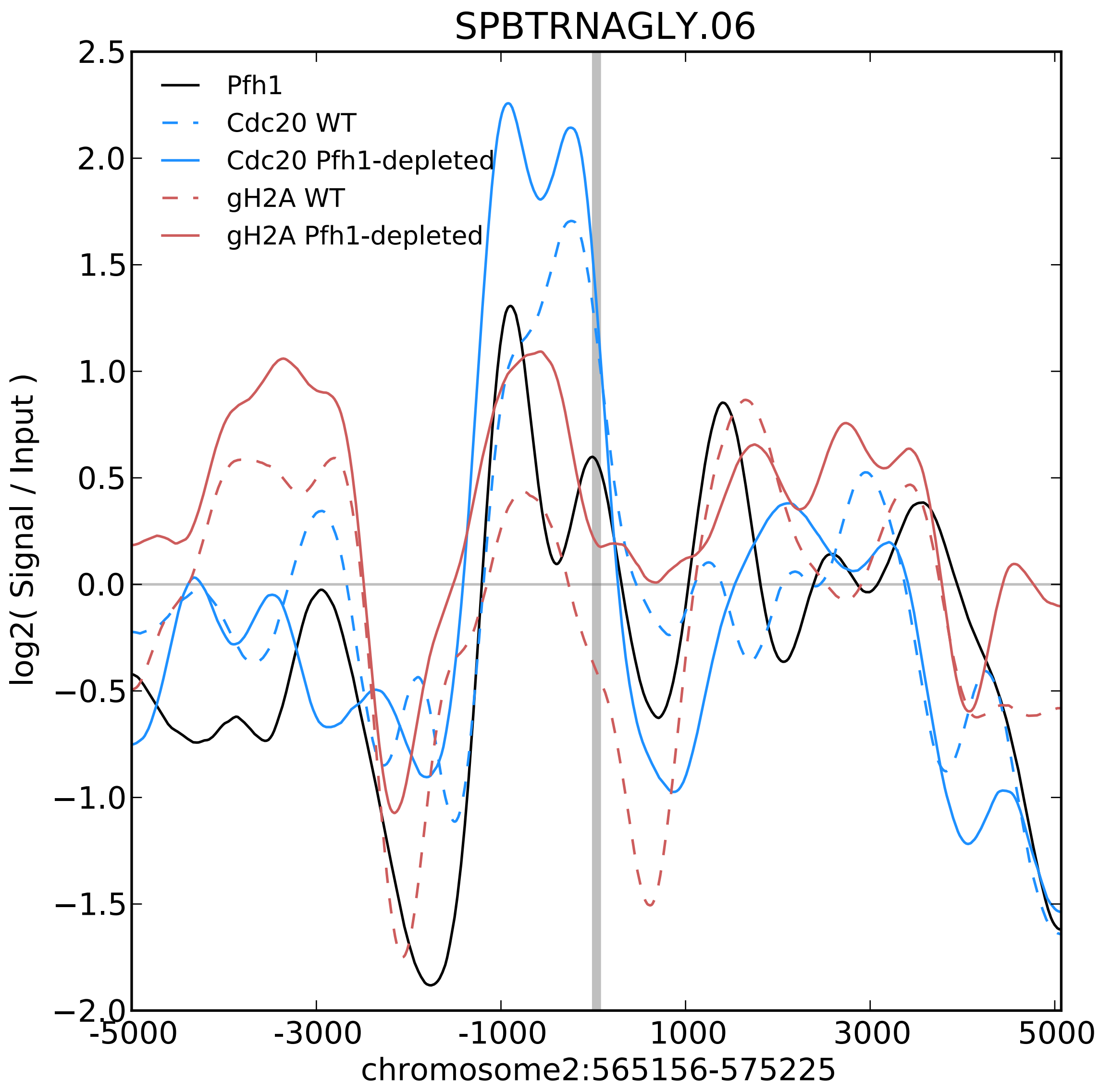
SPBTRNAGLY.06
Pfh1
Cdc20 WT
Cdc20 Pfh1-depleted
gH2A WT
gH2A Pfh1-depleted
log2(Signal / Input)
2.5
2.0
1.5
1.0
0.5
0.0
−0.5
−1.0
−1.5
−2.0
-5000
-3000
-1000
1000
3000
5000
chromosome2:565156-575225

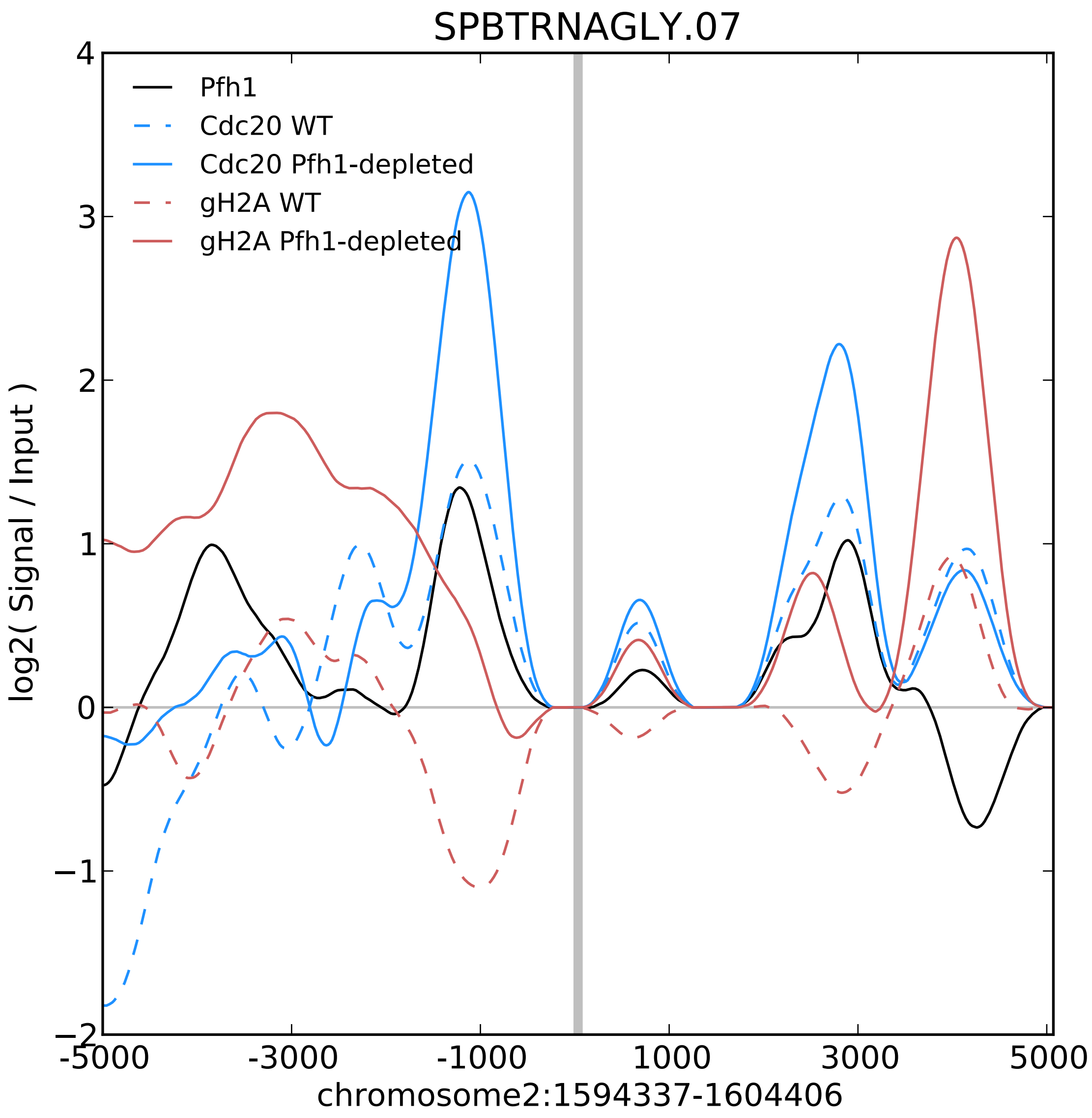

SPBTRNAGLY.07
Pfh1
Cdc20 WT
Cdc20 Pfh1-depleted
gH2A WT
gH2A Pfh1-depleted
4
3
2
1
0
−1
−2
log2(Signal / Input)
-5000
-3000
-1000
1000
3000
5000
chromosome2:1594337-1604406

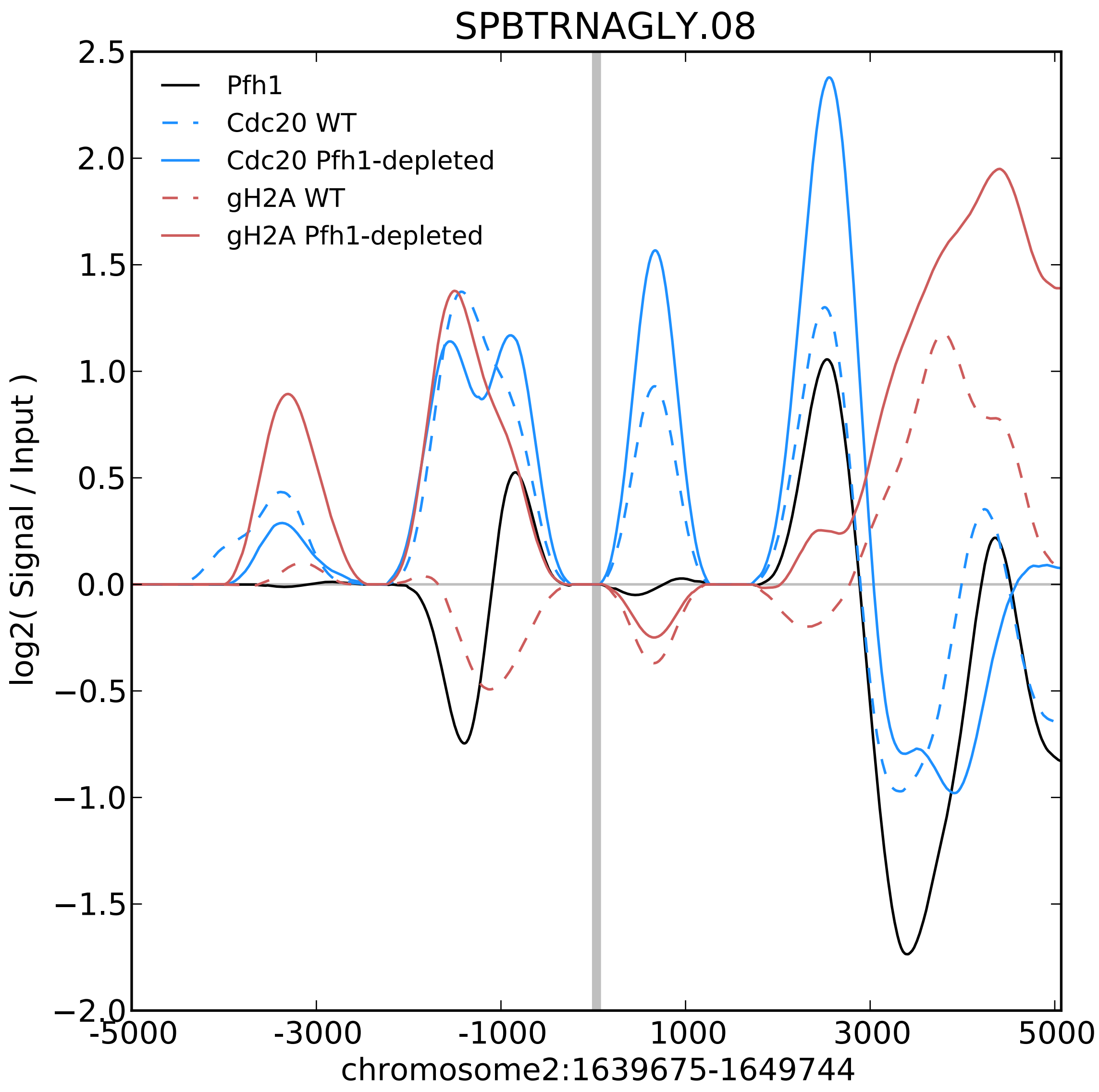

SPBTRNAGLY.08
Pfh1
Cdc20 WT
Cdc20 Pfh1-depleted
gH2A WT
gH2A Pfh1-depleted
log2(Signal / Input)
2.5
2.0
1.5
1.0
0.5
0.0
−0.5
−1.0
−1.5
−2.0
-5000
-3000
-1000
1000
3000
5000
chromosome2:1639675-1649744

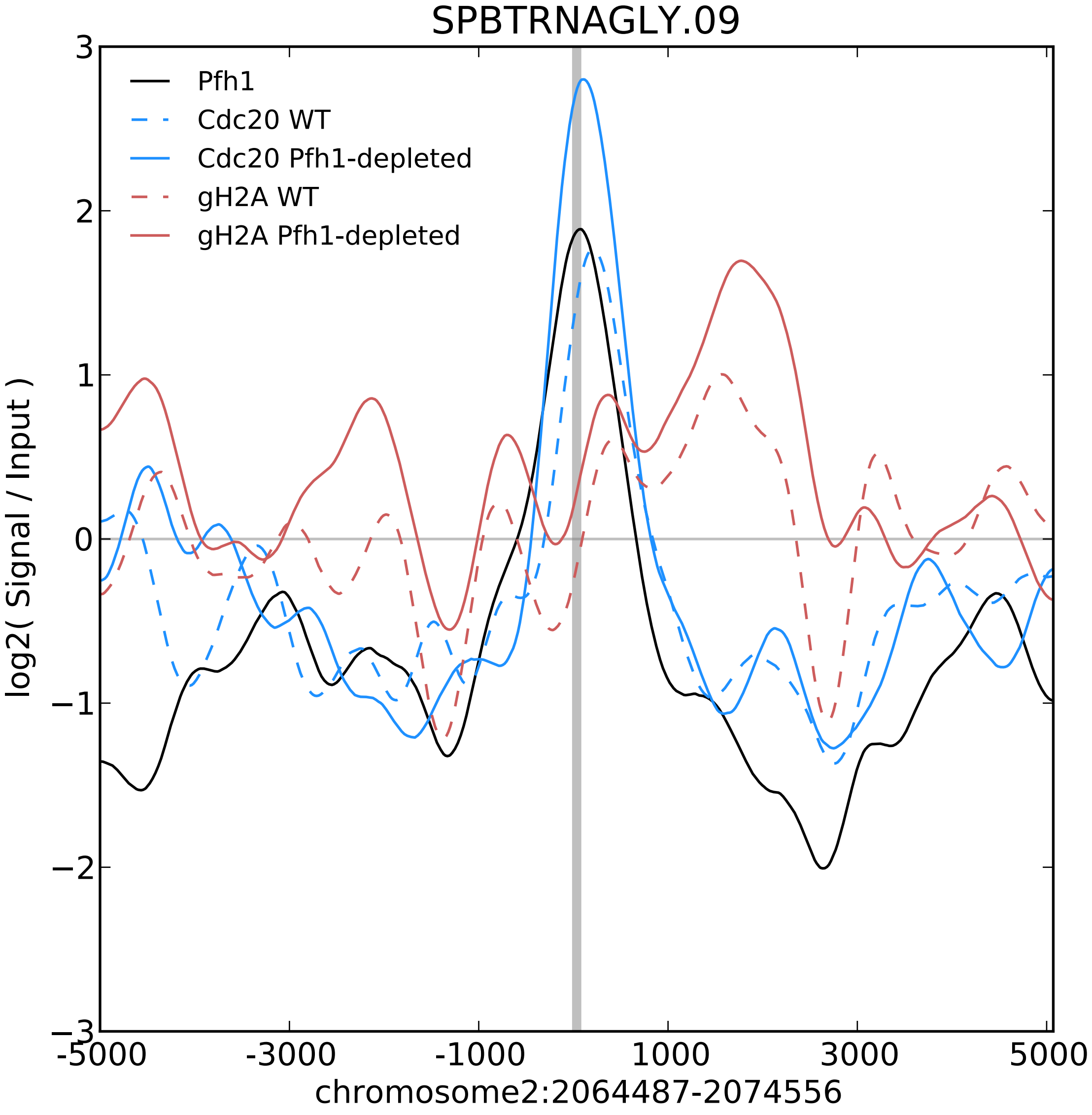
SPBTRNAGLY.09
Pfh1
Cdc20 WT
Cdc20 Pfh1-depleted
gH2A WT
gH2A Pfh1-depleted
log2(Signal / Input)
3
2
1
0
−1
−2
−3
-5000
-3000
-1000
1000
3000
5000
chromosome2:2064487-2074556

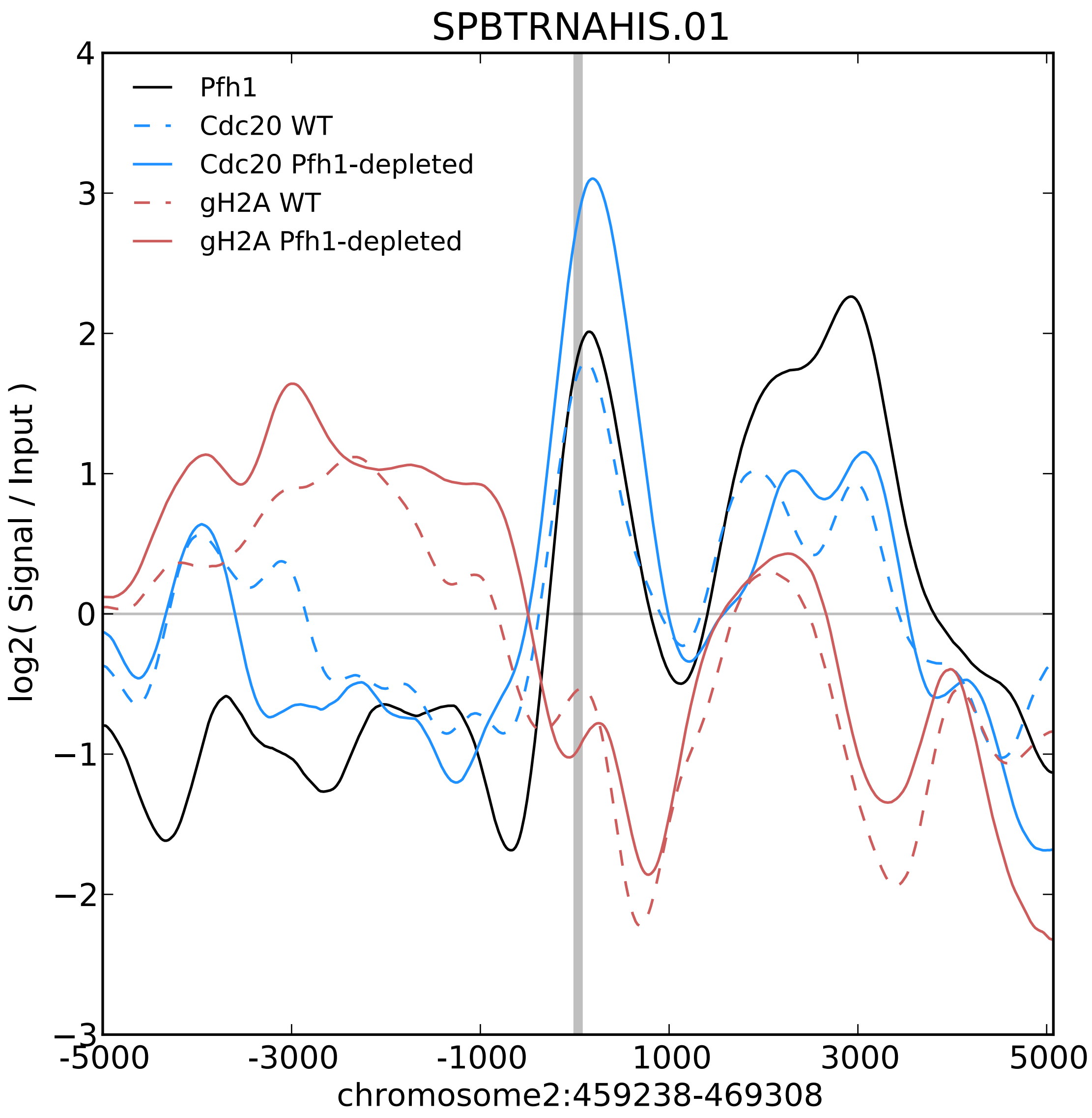
SPBTRNAHIS.01
Pfh1
Cdc20 WT
Cdc20 Pfh1-depleted
gH2A WT
gH2A Pfh1-depleted
log2(Signal / Input)
4
3
2
1
0
−1
−2
−3
-5000
-3000
-1000
1000
3000
5000
chromosome2:459238-469308

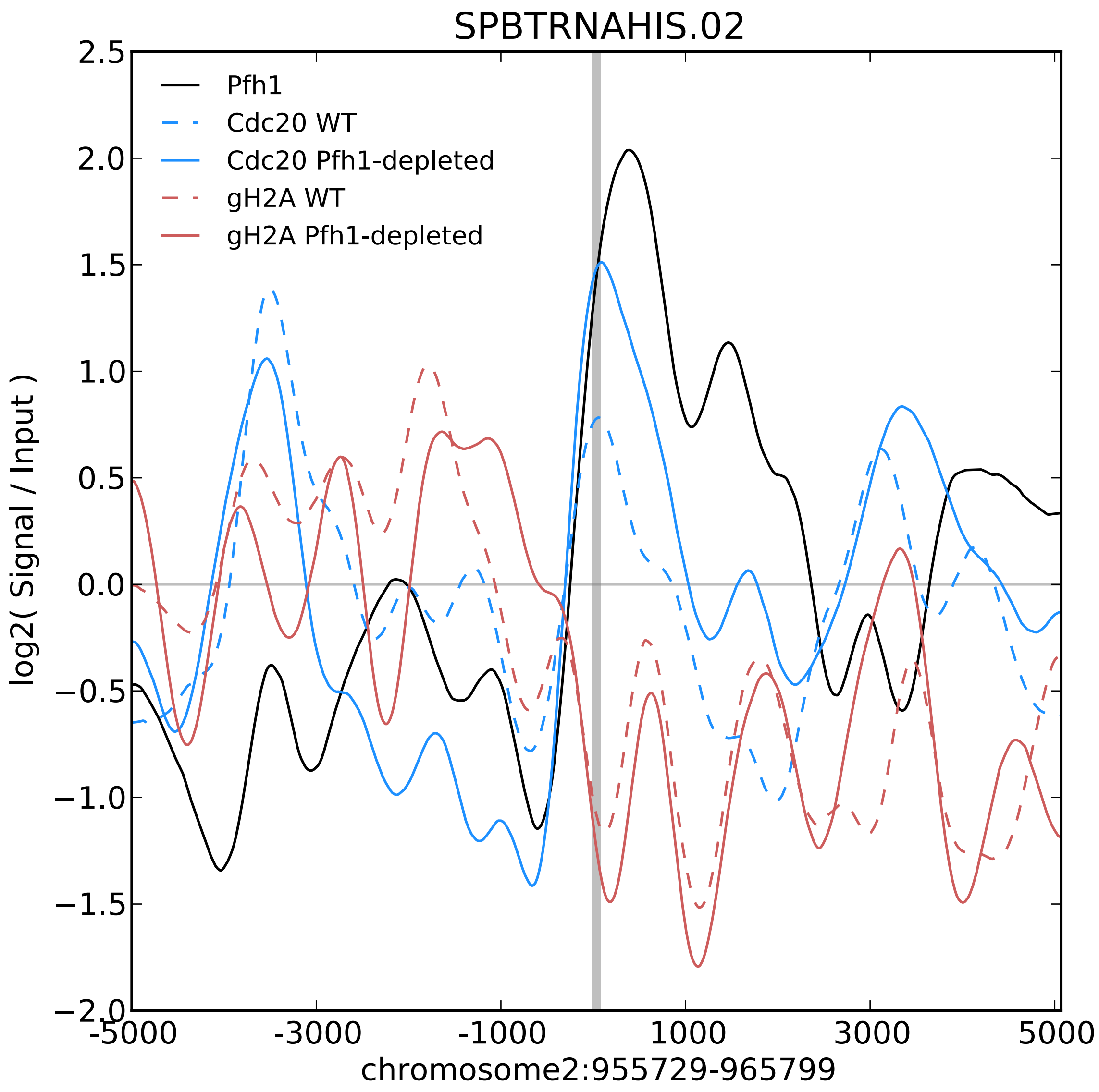

SPBTRNAHIS.02
Pfh1
Cdc20 WT
Cdc20 Pfh1-depleted
gH2A WT
gH2A Pfh1-depleted
log2(Signal / Input)
2.5
2.0
1.5
1.0
0.5
0.0
−0.5
−1.0
−1.5
−2.0
-5000
-3000
-1000
1000
3000
5000
chromosome2:955729-965799

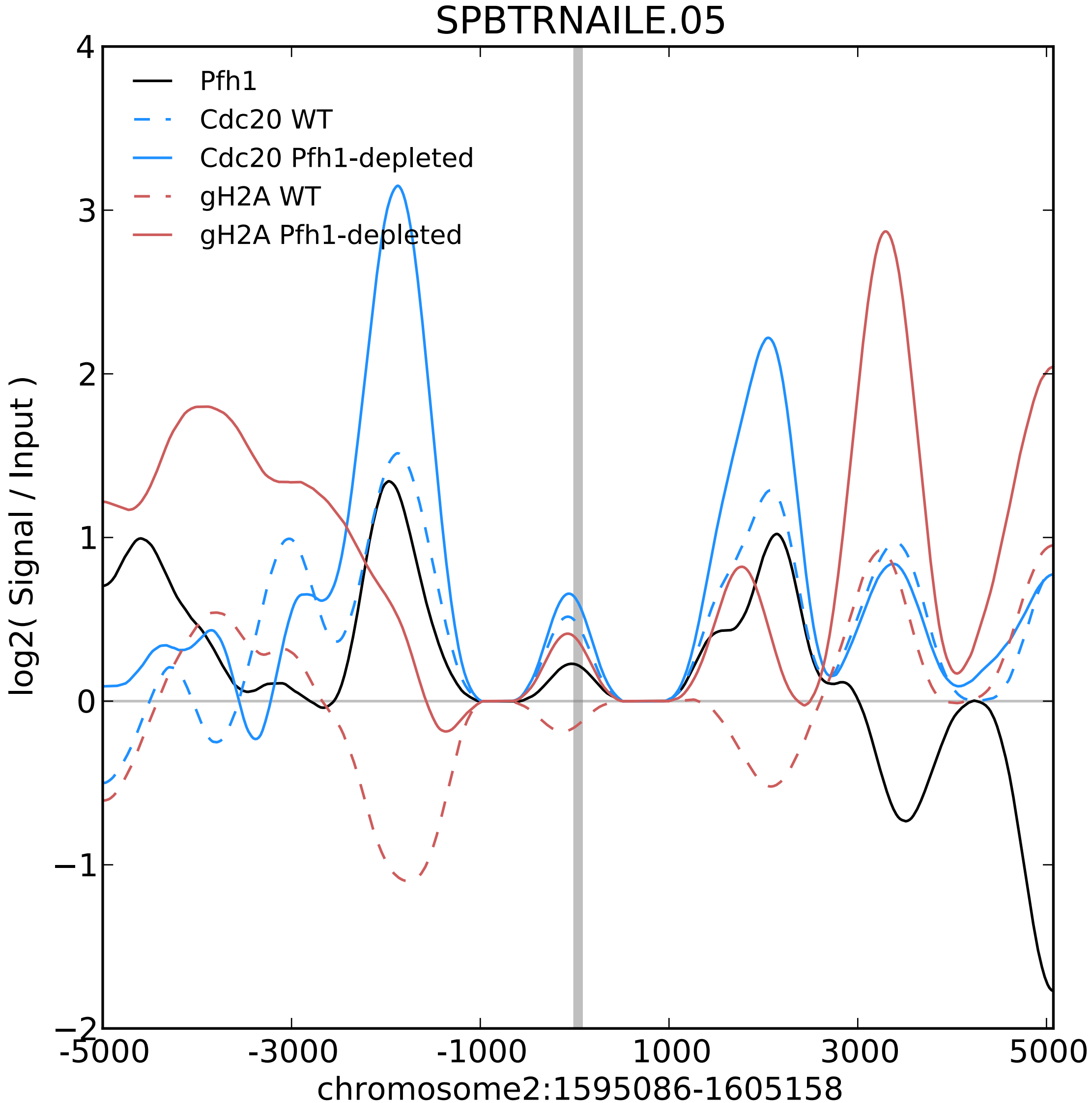
SPBTRNAILE.05
Pfh1
Cdc20 WT
Cdc20 Pfh1-depleted
gH2A WT
gH2A Pfh1-depleted
log2(Signal / Input)
4
3
2
1
0
−1
−2
-5000
-3000
-1000
1000
3000
5000
chromosome2:1595086-1605158

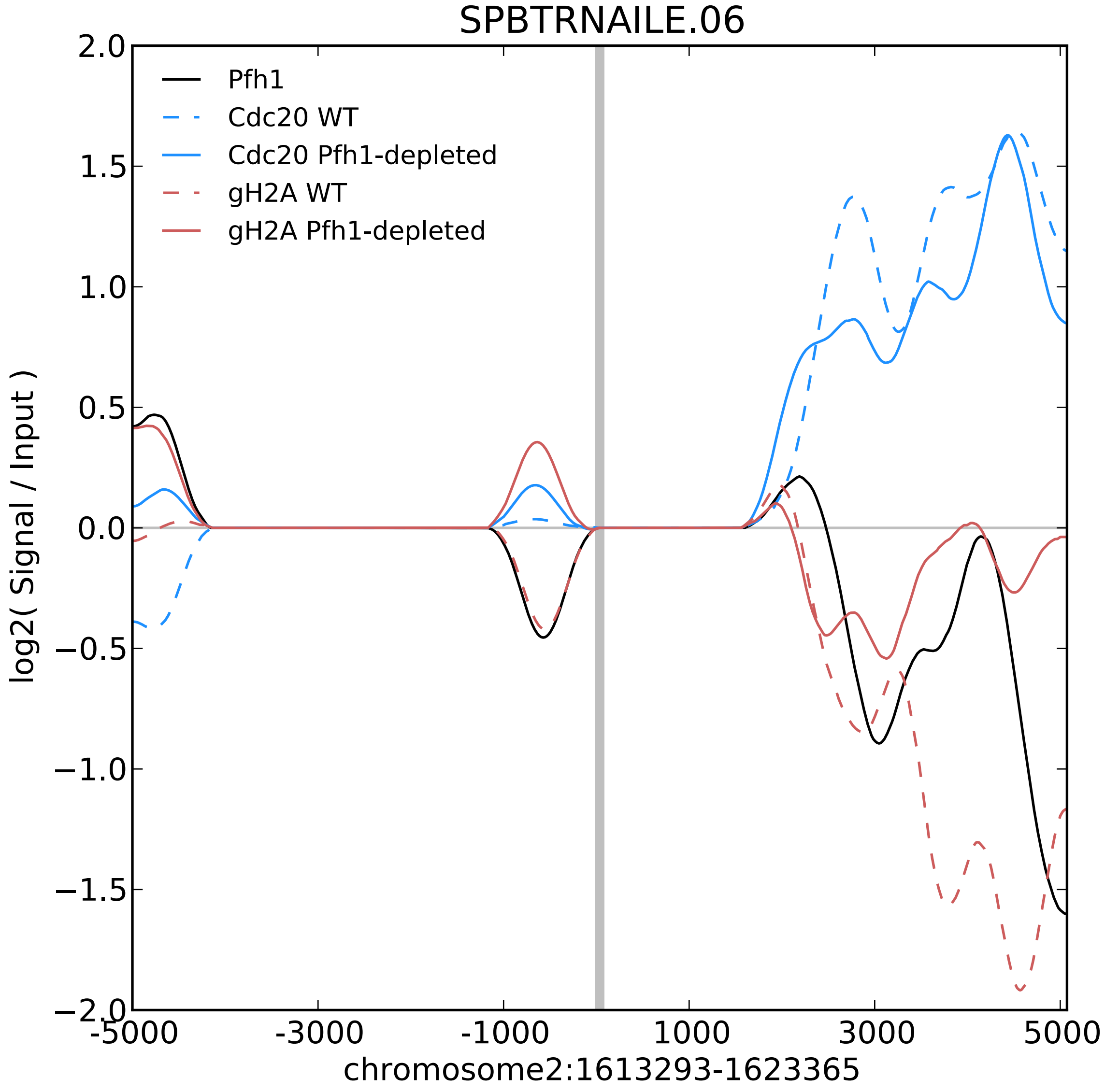
SPBTRNAILE.06
Pfh1
Cdc20 WT
Cdc20 Pfh1-depleted
gH2A WT
gH2A Pfh1-depleted
log2(Signal / Input)
2.0
1.5
1.0
0.5
0.0
−0.5
−1.0
−1.5
−2.0
-5000
-3000
-1000
1000
3000
5000
chromosome2:1613293-1623365

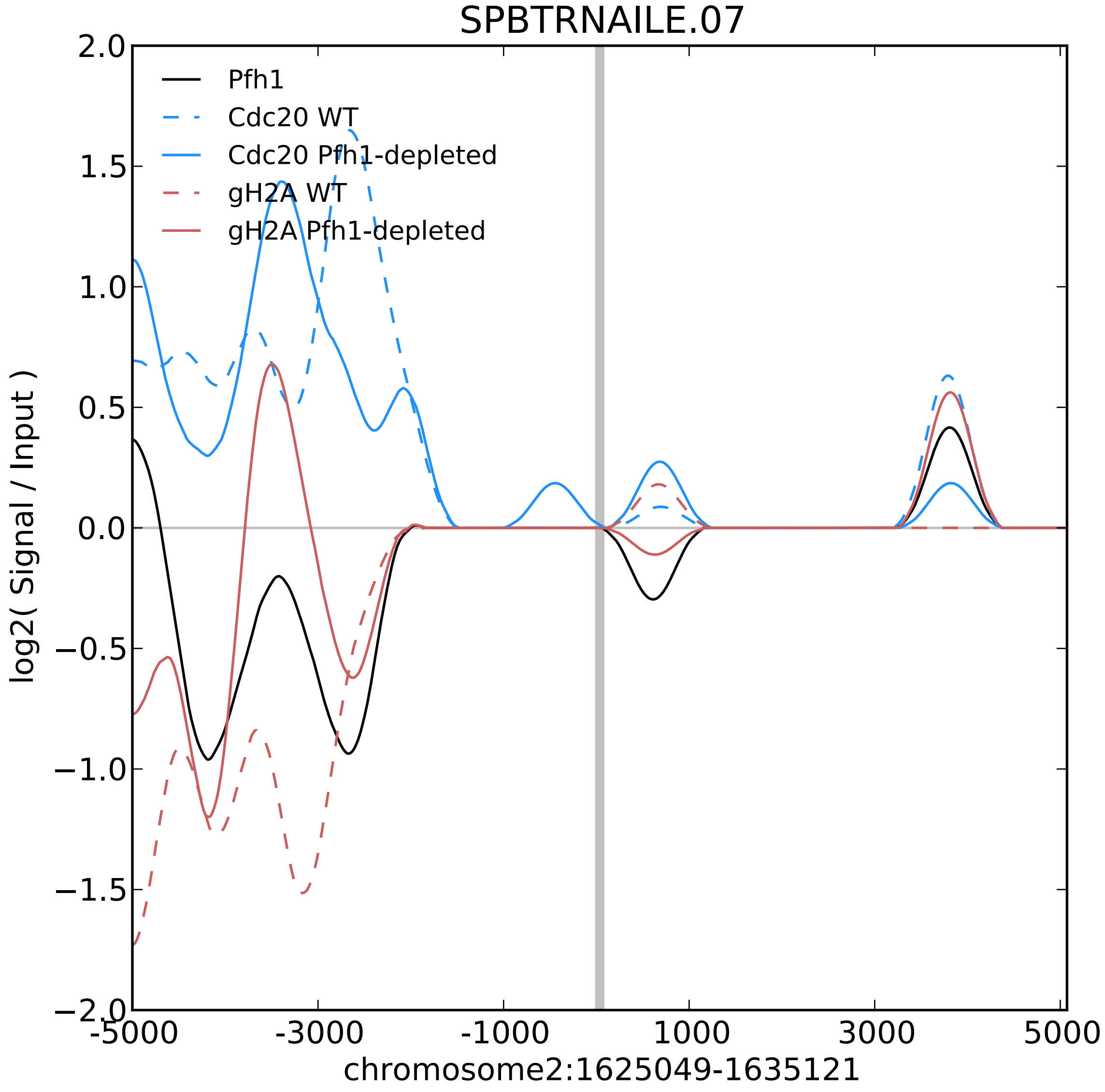
SPBTRNAILE.07
Pfh1
Cdc20 WT
Cdc20 Pfh1-depleted
gH2A WT
gH2A Pfh1-depleted
log2(Signal / Input)
2.0
1.5
1.0
0.5
0.0
−0.5
−1.0
−1.5
−2.0
-5000
-3000
-1000
1000
3000
5000
chromosome2:1625049-1635121

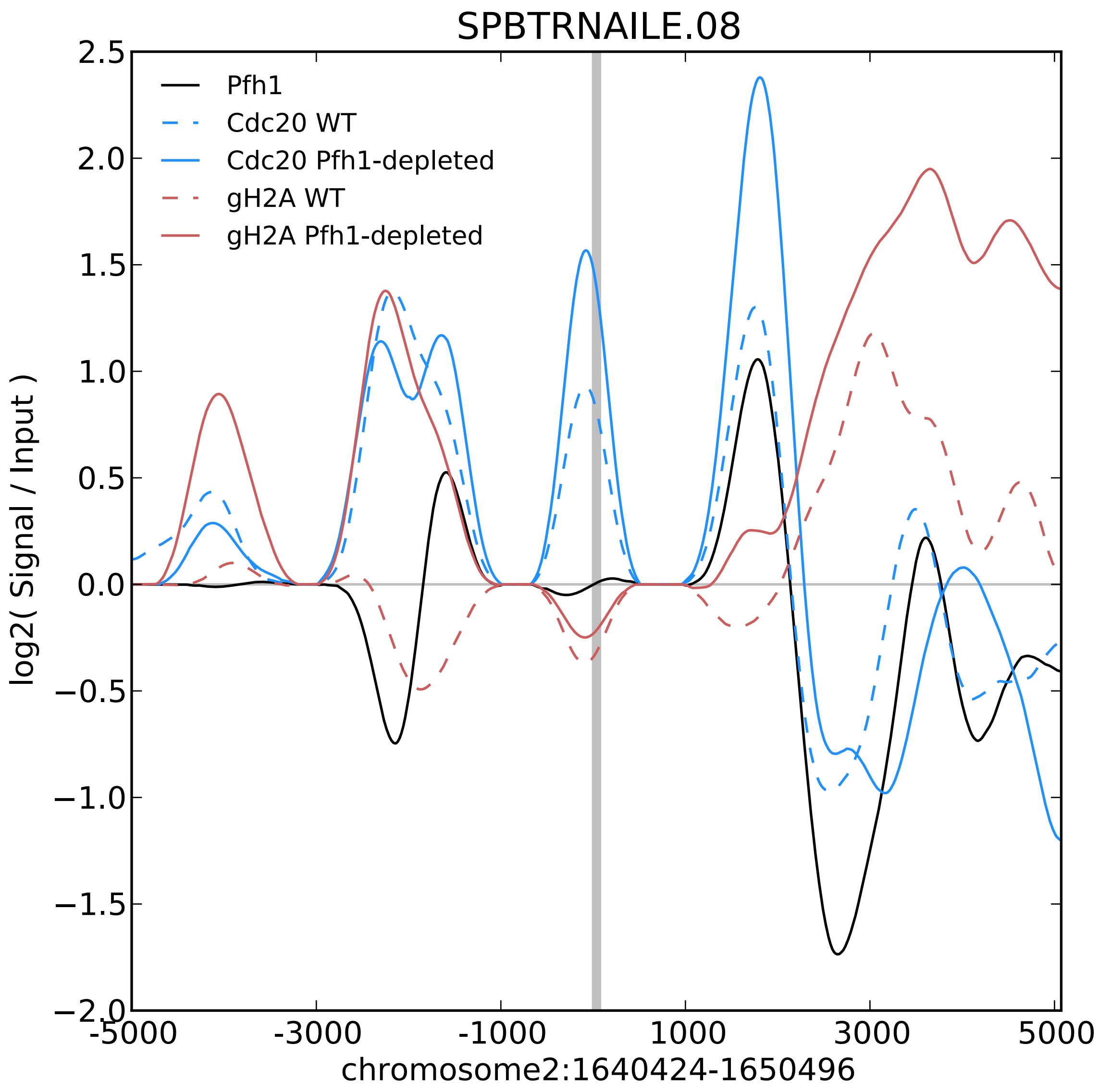
SPBTRNAILE.08
Pfh1
Cdc20 WT
Cdc20 Pfh1-depleted
gH2A WT
gH2A Pfh1-depleted
log2(Signal / Input)
2.5
2.0
1.5
1.0
0.5
0.0
−0.5
−1.0
−1.5
−2.0
-5000
-3000
-1000
1000
3000
5000
chromosome2:1640424-1650496

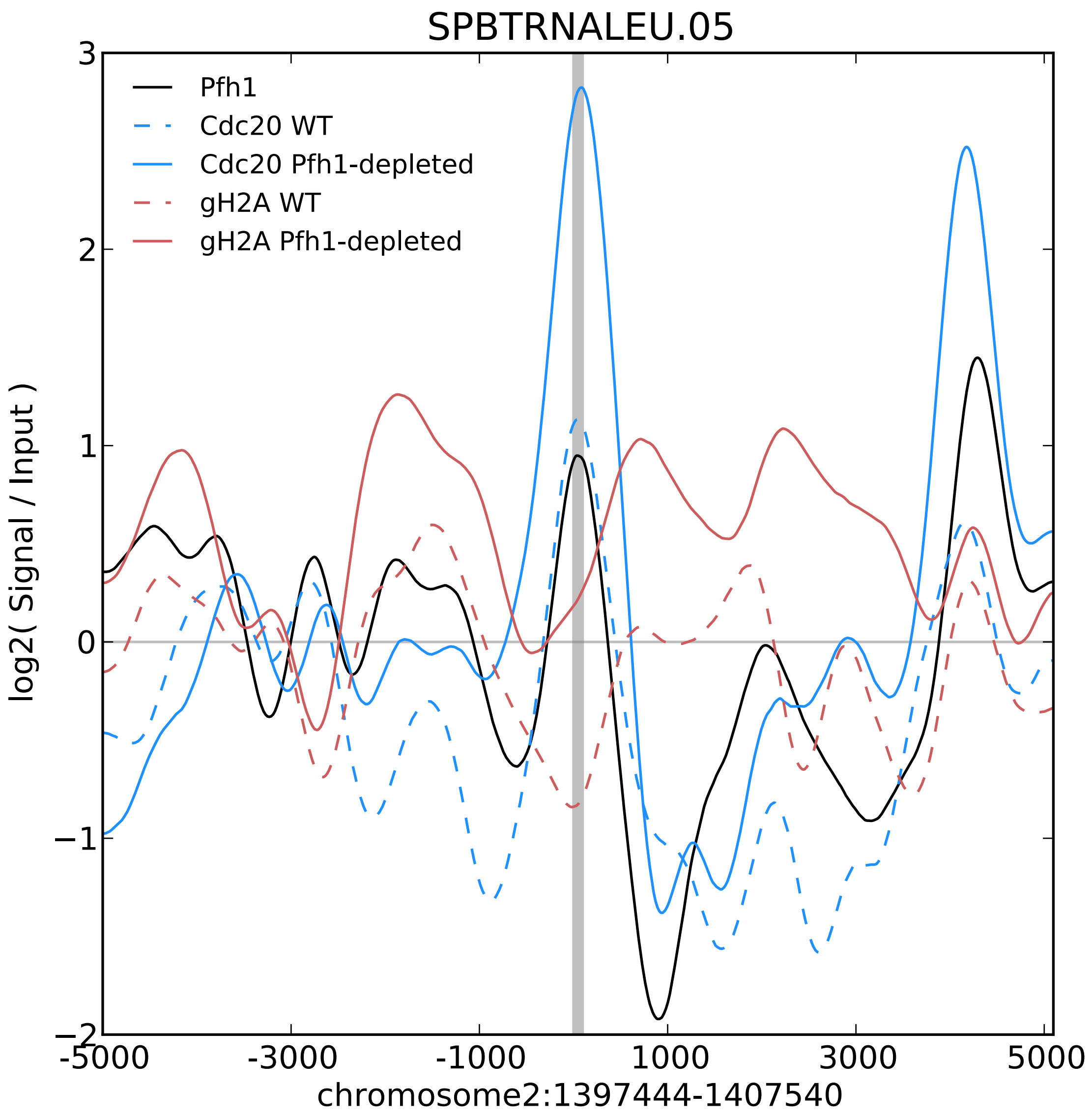

SPBTRNALEU.05
Pfh1
Cdc20 WT
Cdc20 Pfh1-depleted
gH2A WT
gH2A Pfh1-depleted
log2(Signal / Input)
3
2
1
0
−1
−2
-5000
-3000
-1000
1000
3000
5000
chromosome2:1397444-1407540

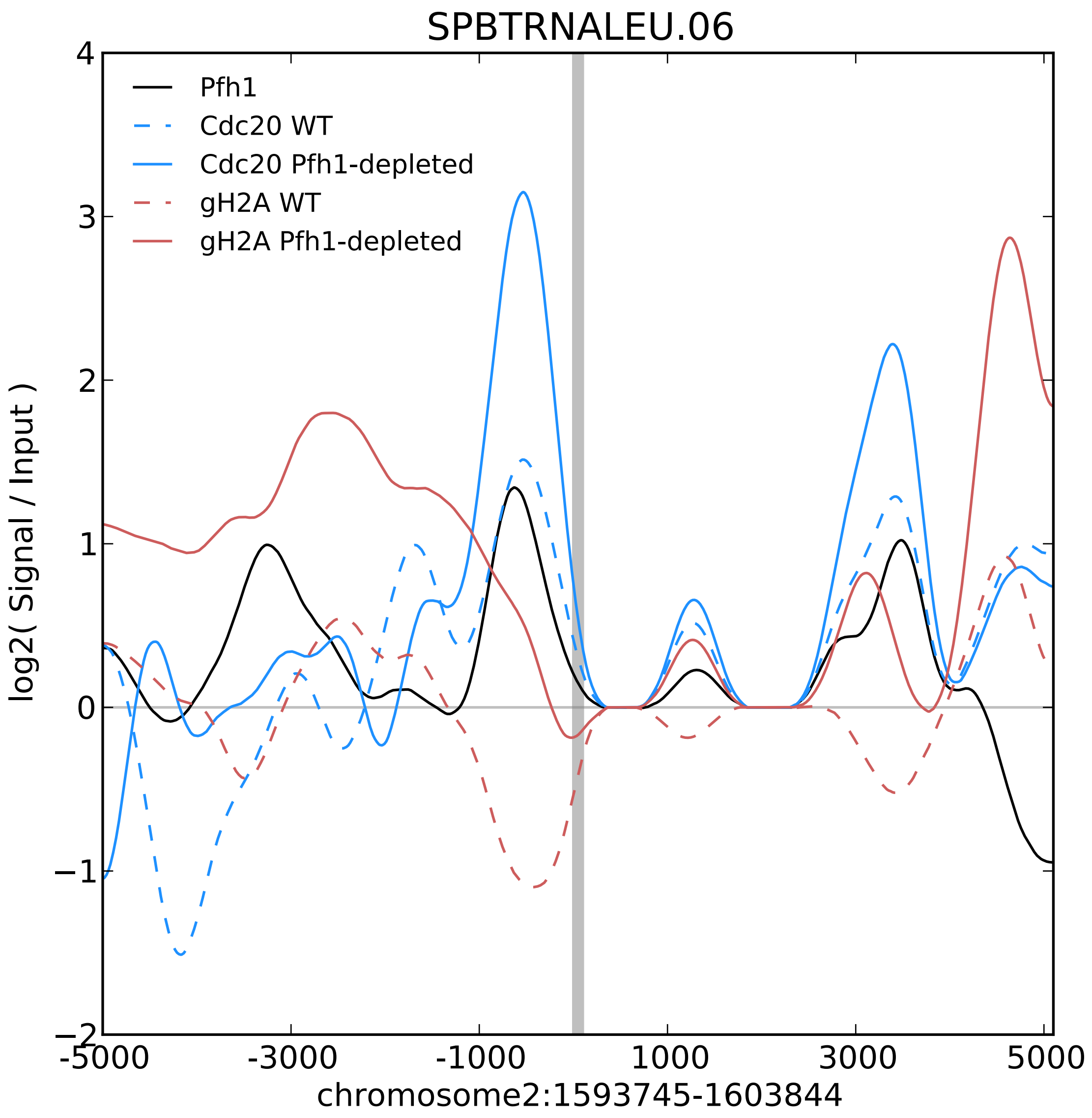
SPBTRNALEU.06
Pfh1
Cdc20 WT
Cdc20 Pfh1-depleted
gH2A WT
gH2A Pfh1-depleted
log2(Signal / Input)
4
3
2
1
0
−1
−2
-5000
-3000
-1000
1000
3000
5000
chromosome2:1593745-1603844

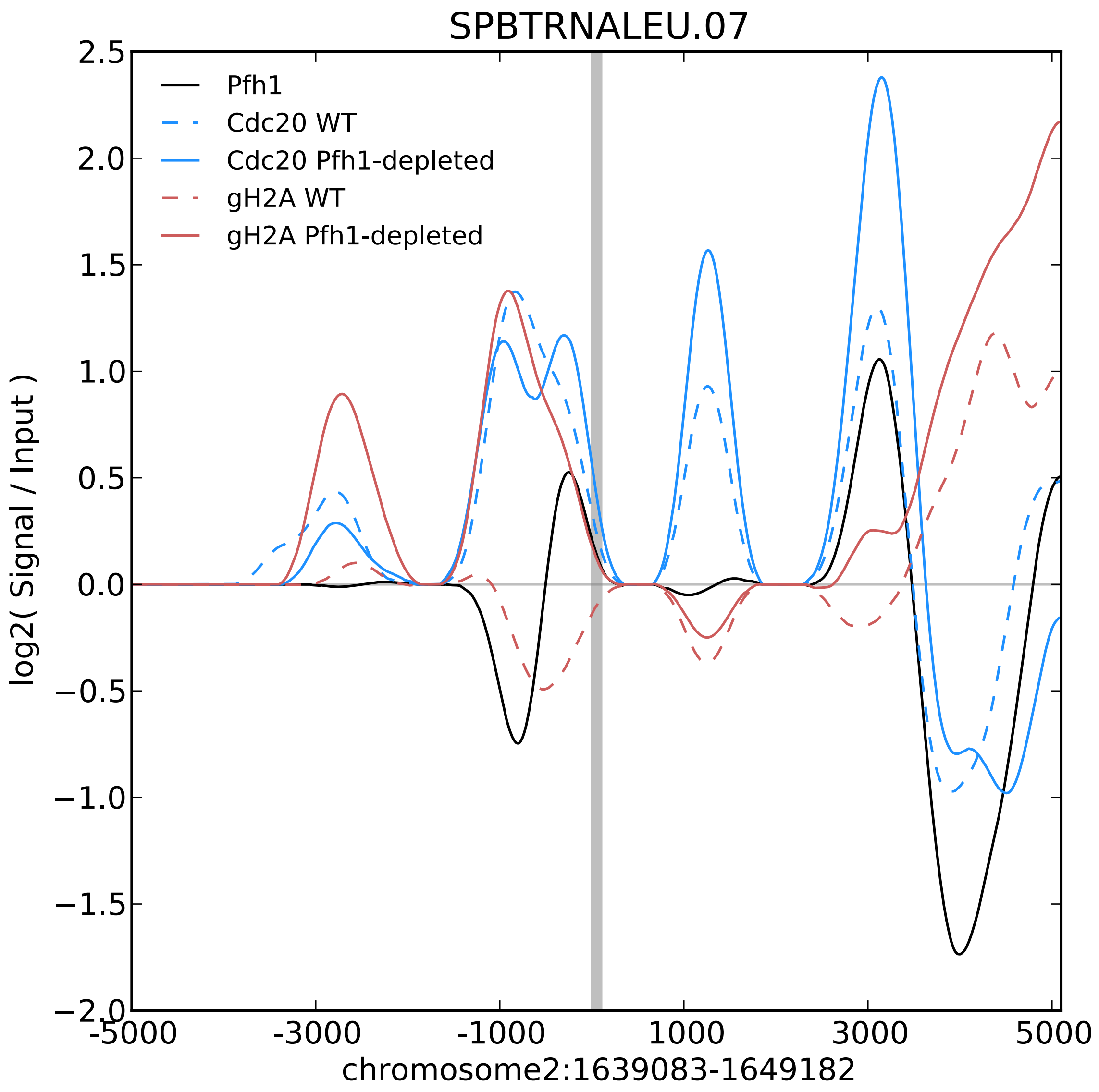

SPBTRNALEU.07
Pfh1
Cdc20 WT
Cdc20 Pfh1-depleted
gH2A WT
gH2A Pfh1-depleted
log2(Signal / Input)
2.5
2.0
1.5
1.0
0.5
0.0
−0.5
−1.0
−1.5
−2.0
-5000
-3000
-1000
1000
3000
5000
chromosome2:1639083-1649182

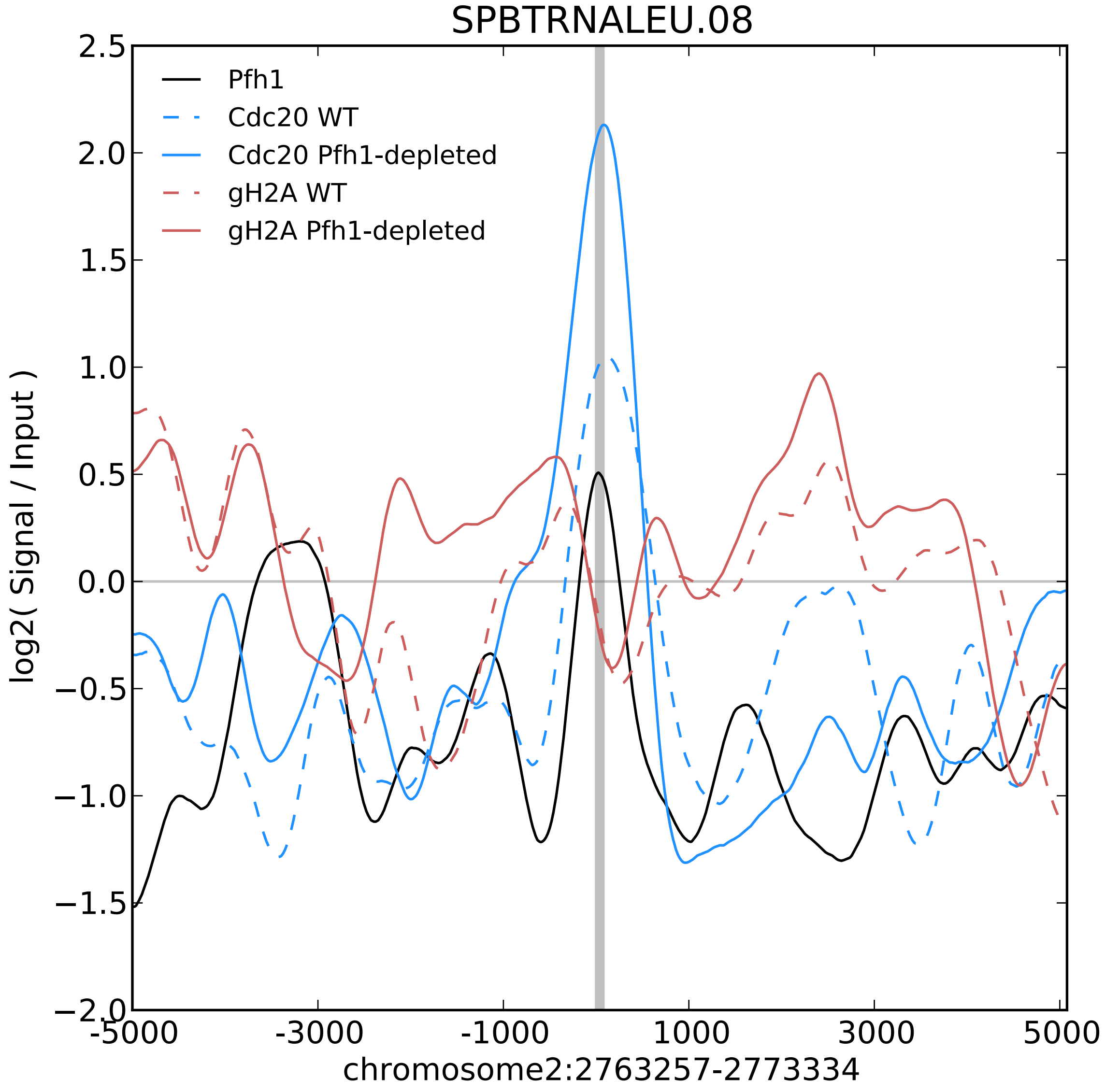
SPBTRNALEU.08
Pfh1
Cdc20 WT
Cdc20 Pfh1-depleted
gH2A WT
gH2A Pfh1-depleted
log2(Signal / Input)
2.5
2.0
1.5
1.0
0.5
0.0
−0.5
−1.0
−1.5
−2.0
-5000
-3000
-1000
1000
3000
5000
chromosome2:2763257-2773334

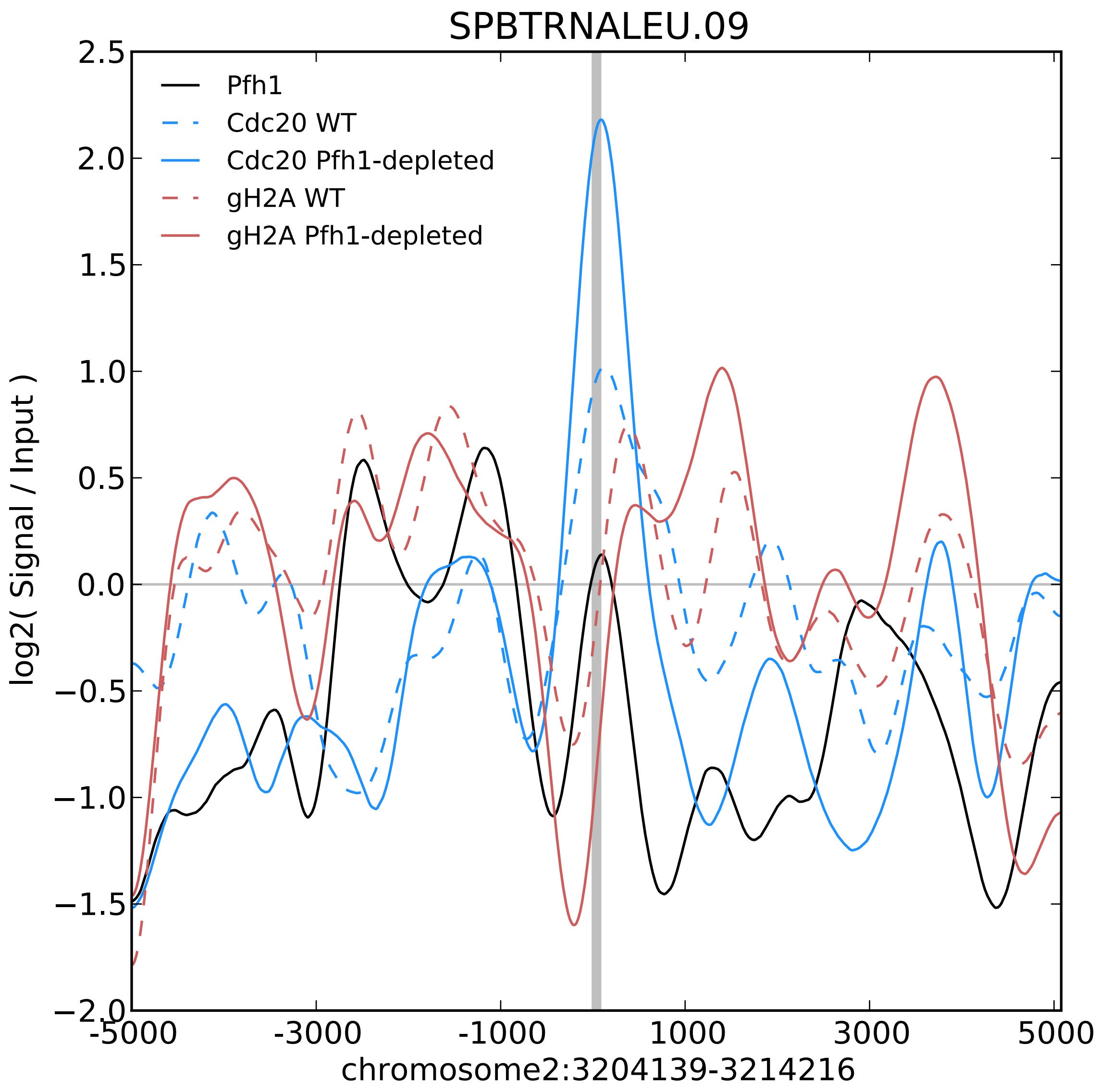
SPBTRNALEU.09
Pfh1
Cdc20 WT
Cdc20 Pfh1-depleted
gH2A WT
gH2A Pfh1-depleted
log2(Signal / Input)
2.5
2.0
1.5
1.0
0.5
0.0
−0.5
−1.0
−1.5
−2.0
-5000
-3000
-1000
1000
3000
5000
chromosome2:3204139-3214216

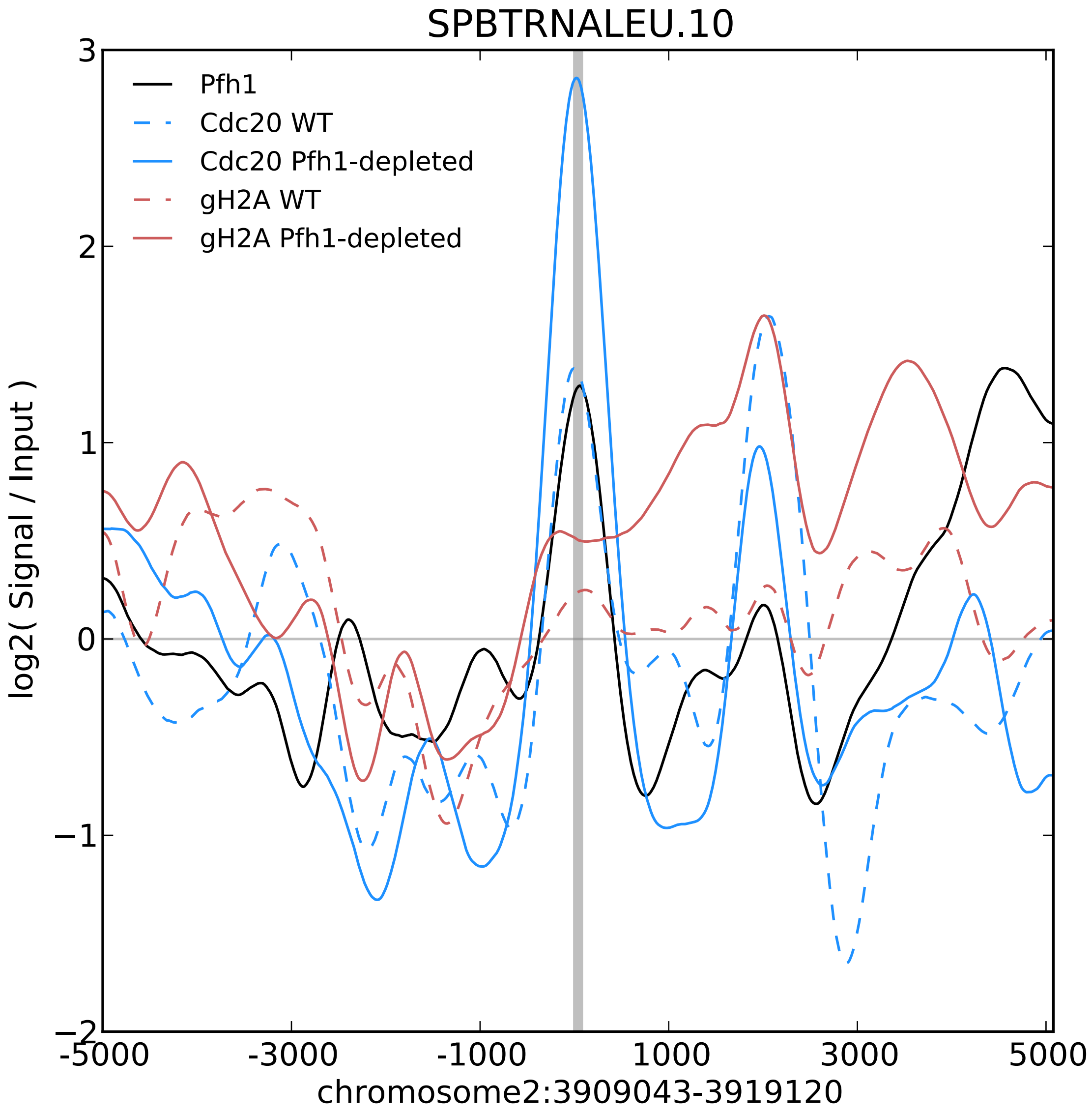
SPBTRNALEU.10
Pfh1
Cdc20 WT
Cdc20 Pfh1-depleted
gH2A WT
gH2A Pfh1-depleted
log2(Signal / Input)
3
2
1
0
−1
−2
-5000
-3000
-1000
1000
3000
5000
chromosome2:3909043-3919120

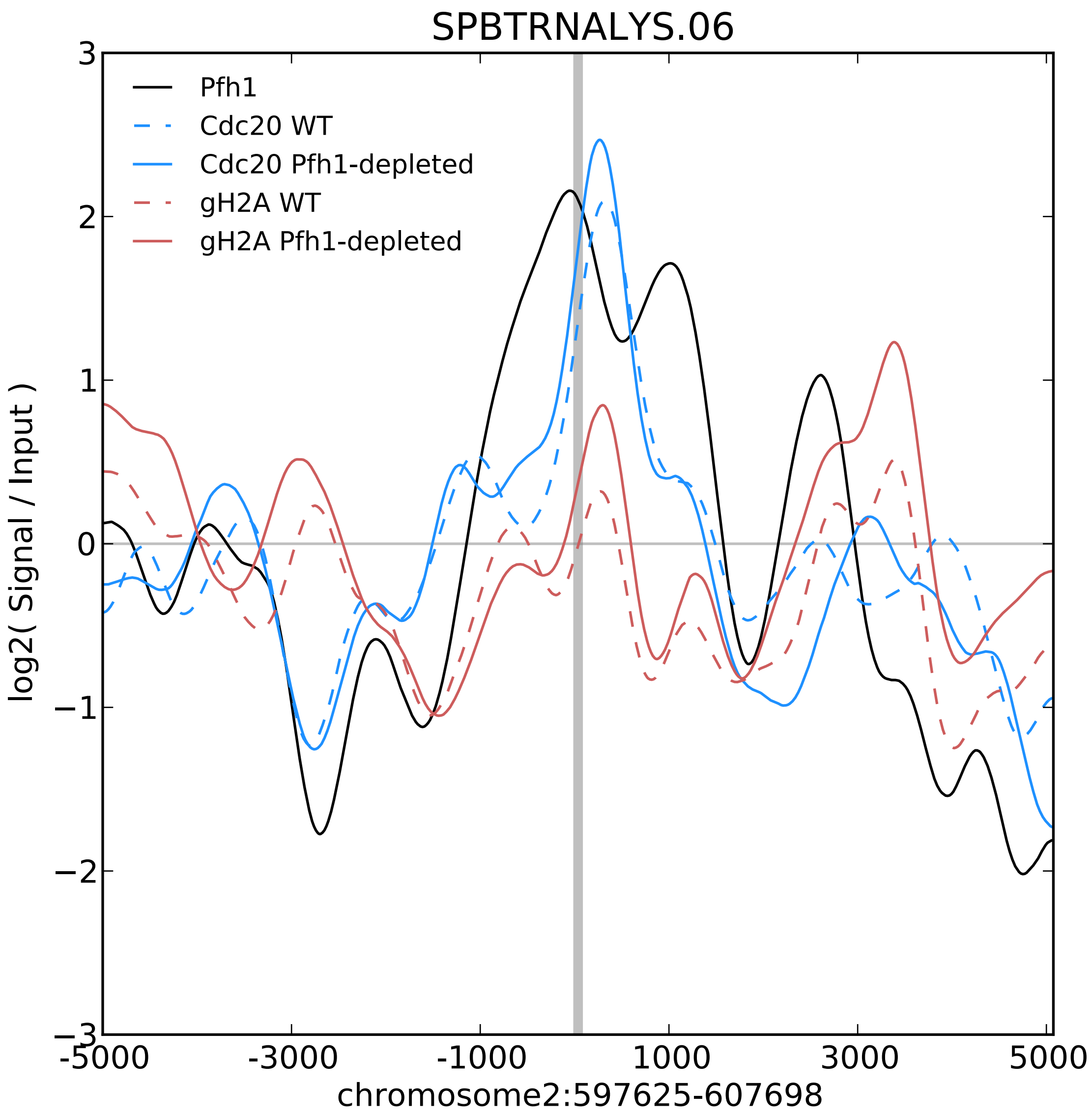
SPBTRNALYS.06
Pfh1
Cdc20 WT
Cdc20 Pfh1-depleted
gH2A WT
gH2A Pfh1-depleted
log2(Signal / Input)
3
2
1
0
−1
−2
−3
-5000
-3000
-1000
1000
3000
5000
chromosome2:597625-607698

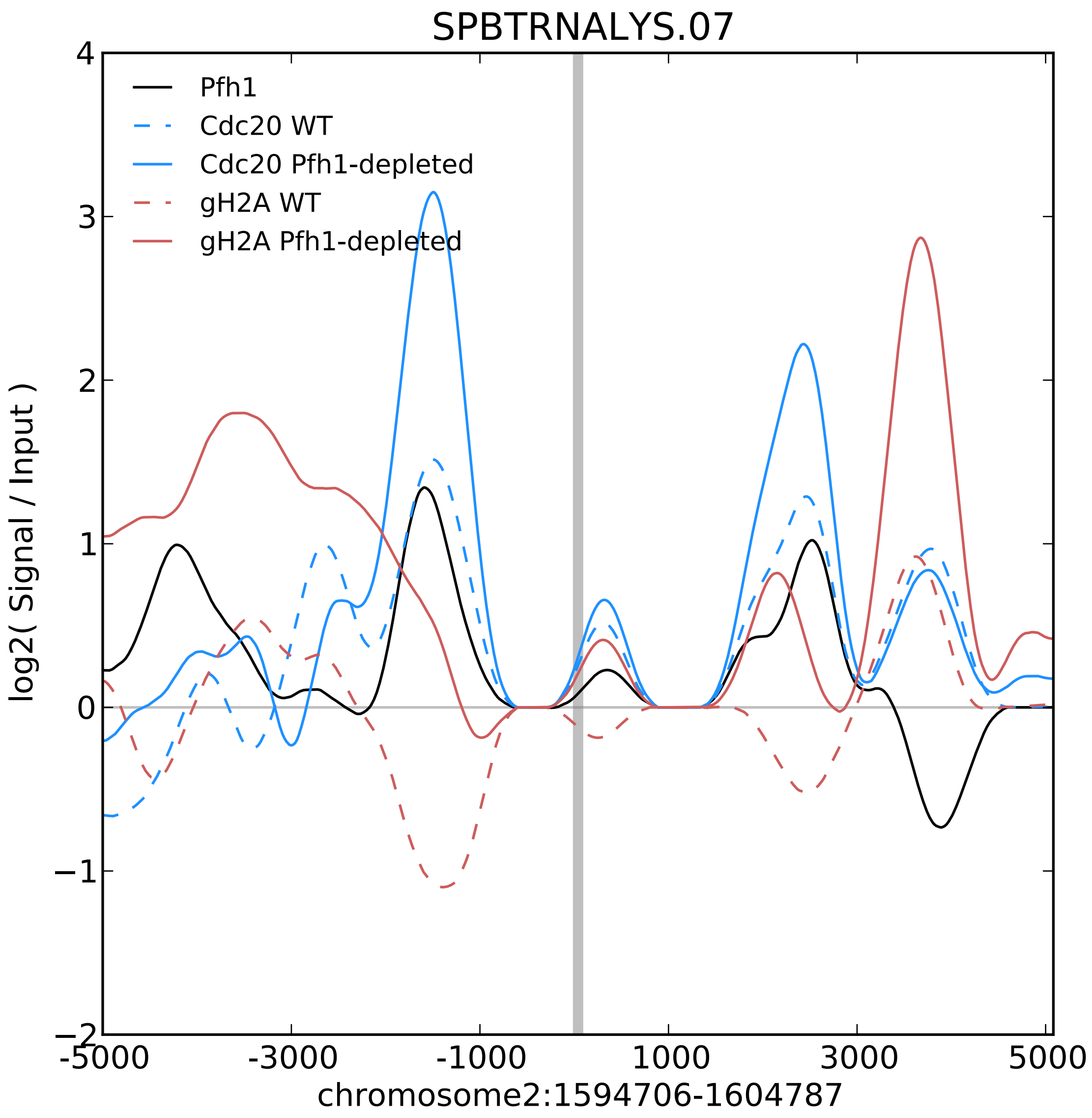
SPBTRNALYS.07
Pfh1
Cdc20 WT
Cdc20 Pfh1-depleted
gH2A WT
gH2A Pfh1-depleted
log2(Signal / Input)
4
3
2
1
0
−1
−2
-5000
-3000
-1000
1000
3000
5000
chromosome2:1594706-1604787

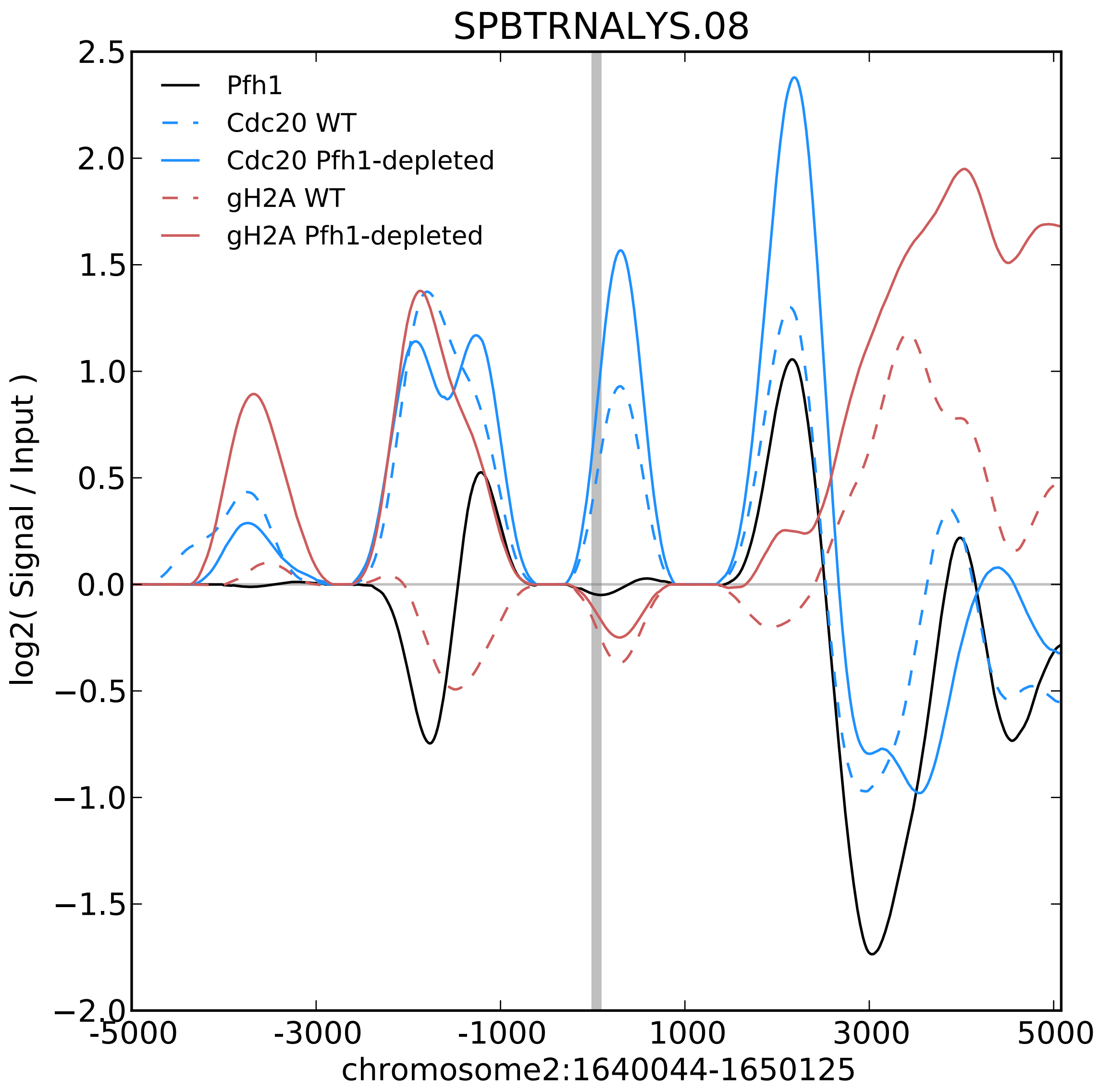

SPBTRNALYS.08
Pfh1
Cdc20 WT
Cdc20 Pfh1-depleted
gH2A WT
gH2A Pfh1-depleted
log2(Signal / Input)
2.5
2.0
1.5
1.0
0.5
0.0
−0.5
−1.0
−1.5
−2.0
-5000
-3000
-1000
1000
3000
5000
chromosome2:1640044-1650125

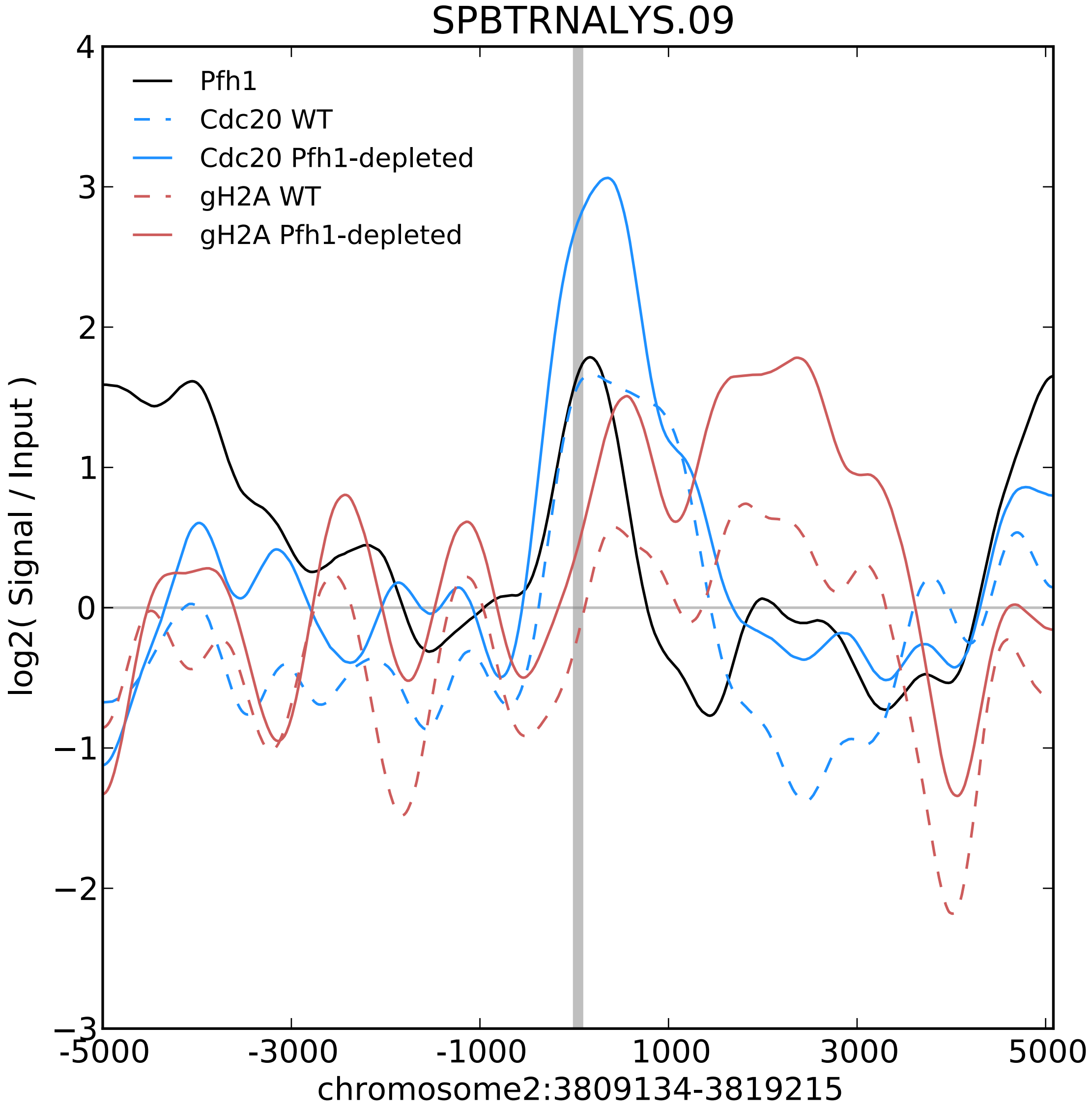

SPBTRNALYS.09
Pfh1
Cdc20 WT
Cdc20 Pfh1-depleted
gH2A WT
gH2A Pfh1-depleted
log2(Signal / Input)
4
3
2
1
0
−1
−2
−3
-5000
-3000
-1000
1000
3000
5000
chromosome2:3809134-3819215

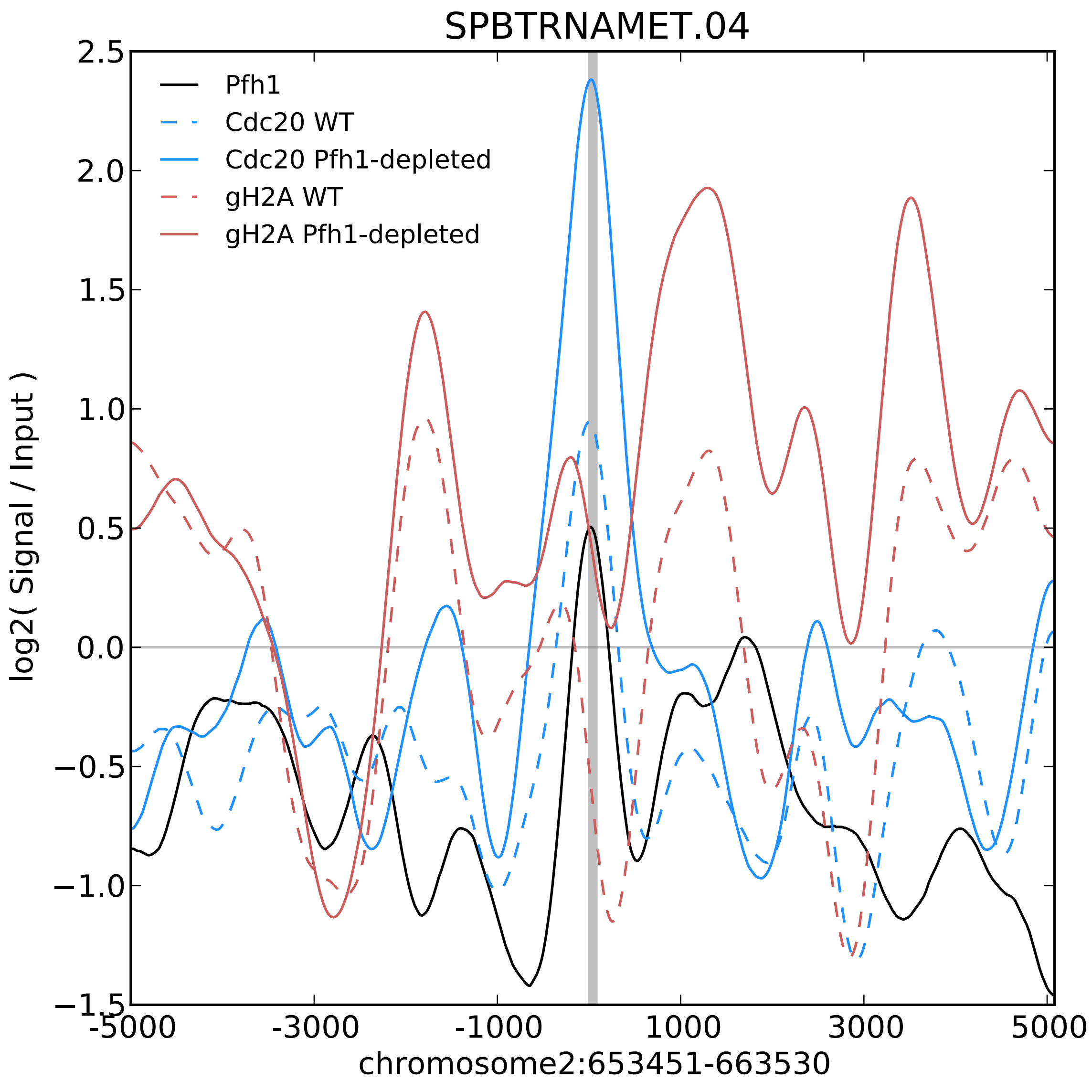
SPBTRNAMET.04
Pfh1
Cdc20 WT
Cdc20 Pfh1-depleted
gH2A WT
gH2A Pfh1-depleted
2.5
2.0
1.5
1.0
0.5
0.0
−0.5
−1.0
−1.5
log2(Signal / Input)
-5000
-3000
-1000
1000
3000
5000
chromosome2:653451-663530

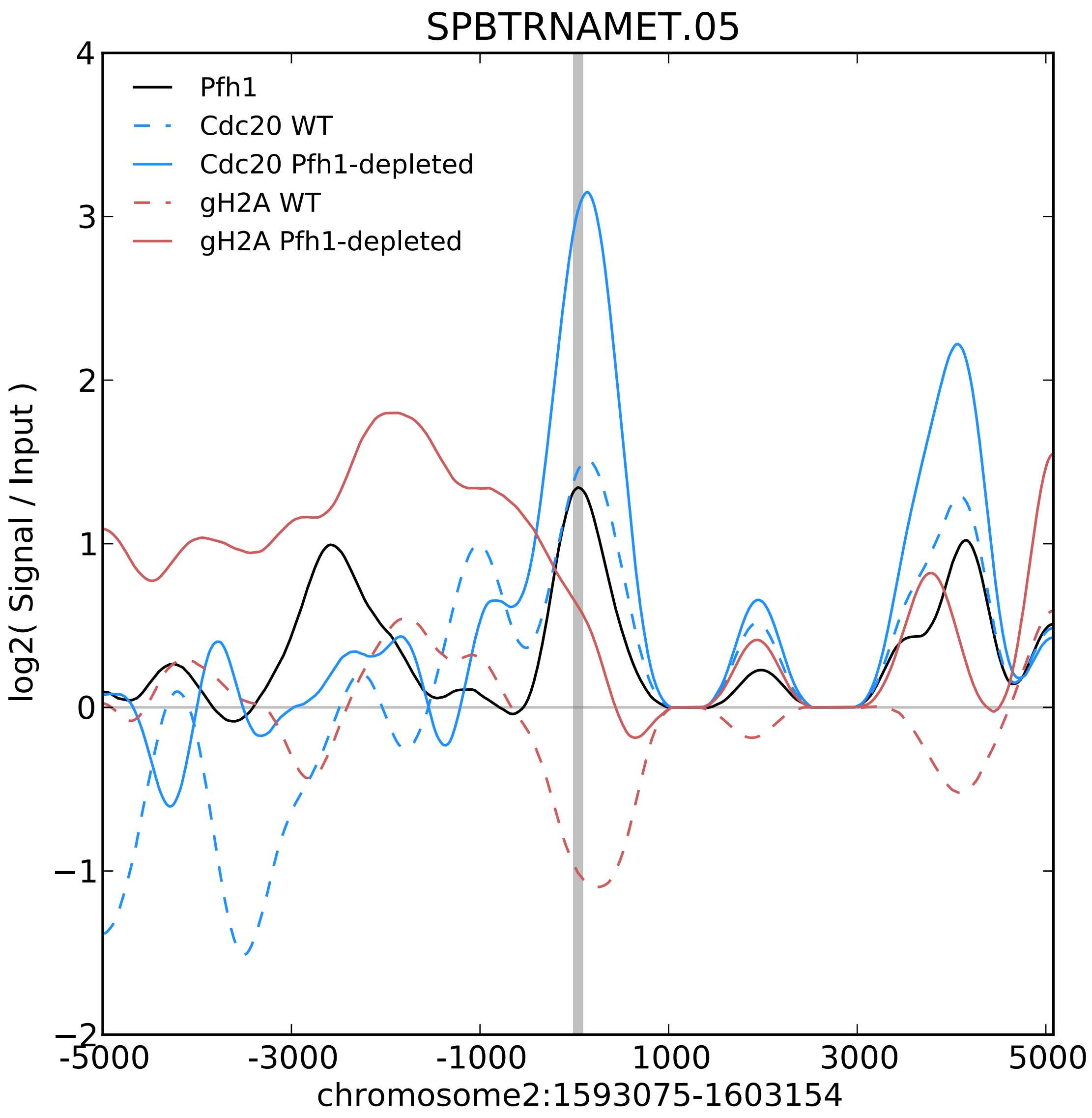

SPBTRNAMET.05
Pfh1
Cdc20 WT
Cdc20 Pfh1-depleted
gH2A WT
gH2A Pfh1-depleted
log2(Signal / Input)
4
3
2
1
0
−1
−2
-5000
-3000
-1000
1000
3000
5000
chromosome2:1593075-1603154

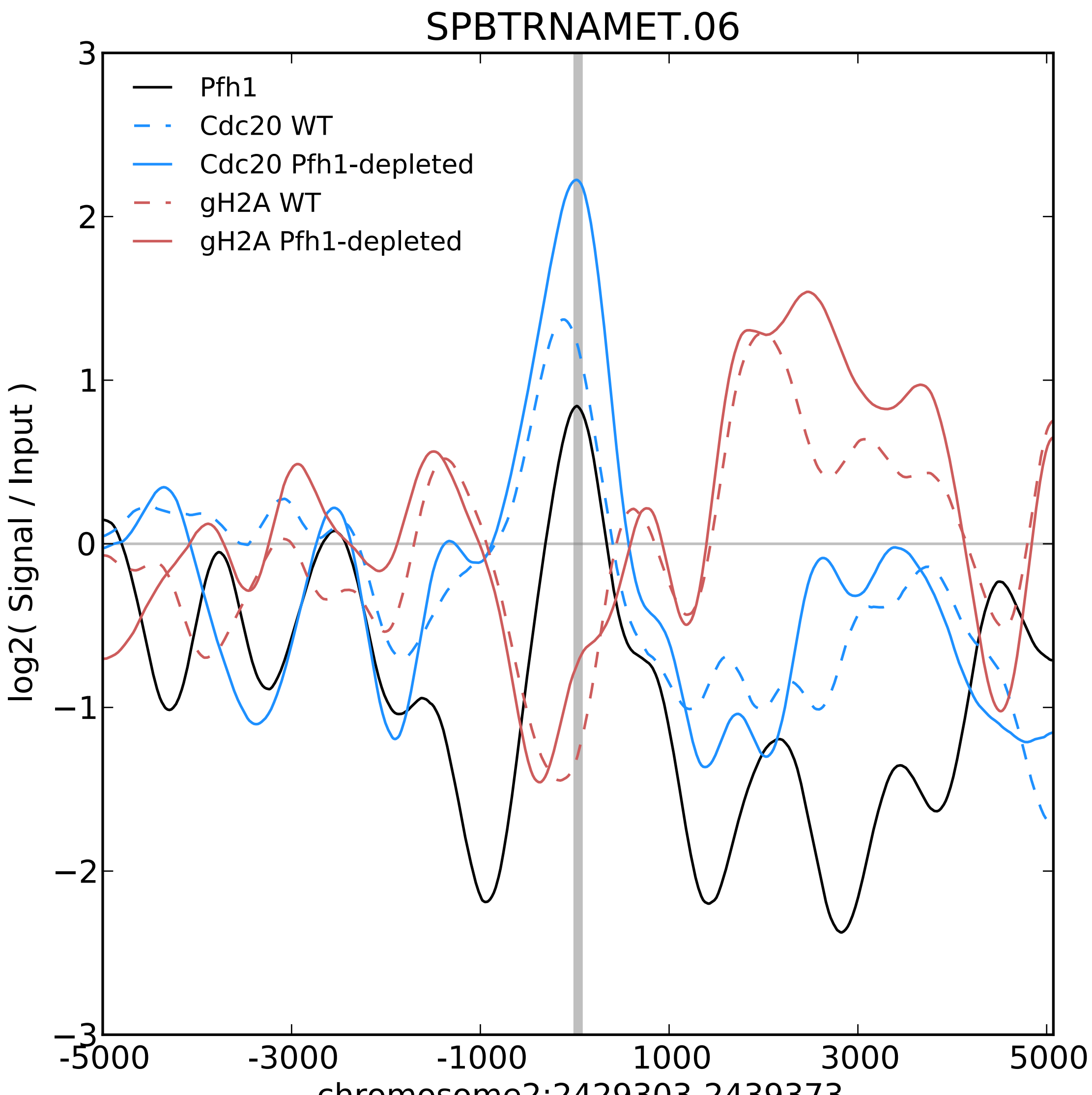
SPBTRNAMET.06
Pfh1
Cdc20 WT
Cdc20 Pfh1-depleted
gH2A WT
gH2A Pfh1-depleted
log2(Signal / Input)
3
2
1
0
−1
−2
−3
-5000
-3000
-1000
1000
3000
5000
chromosome2:2429303-2439373

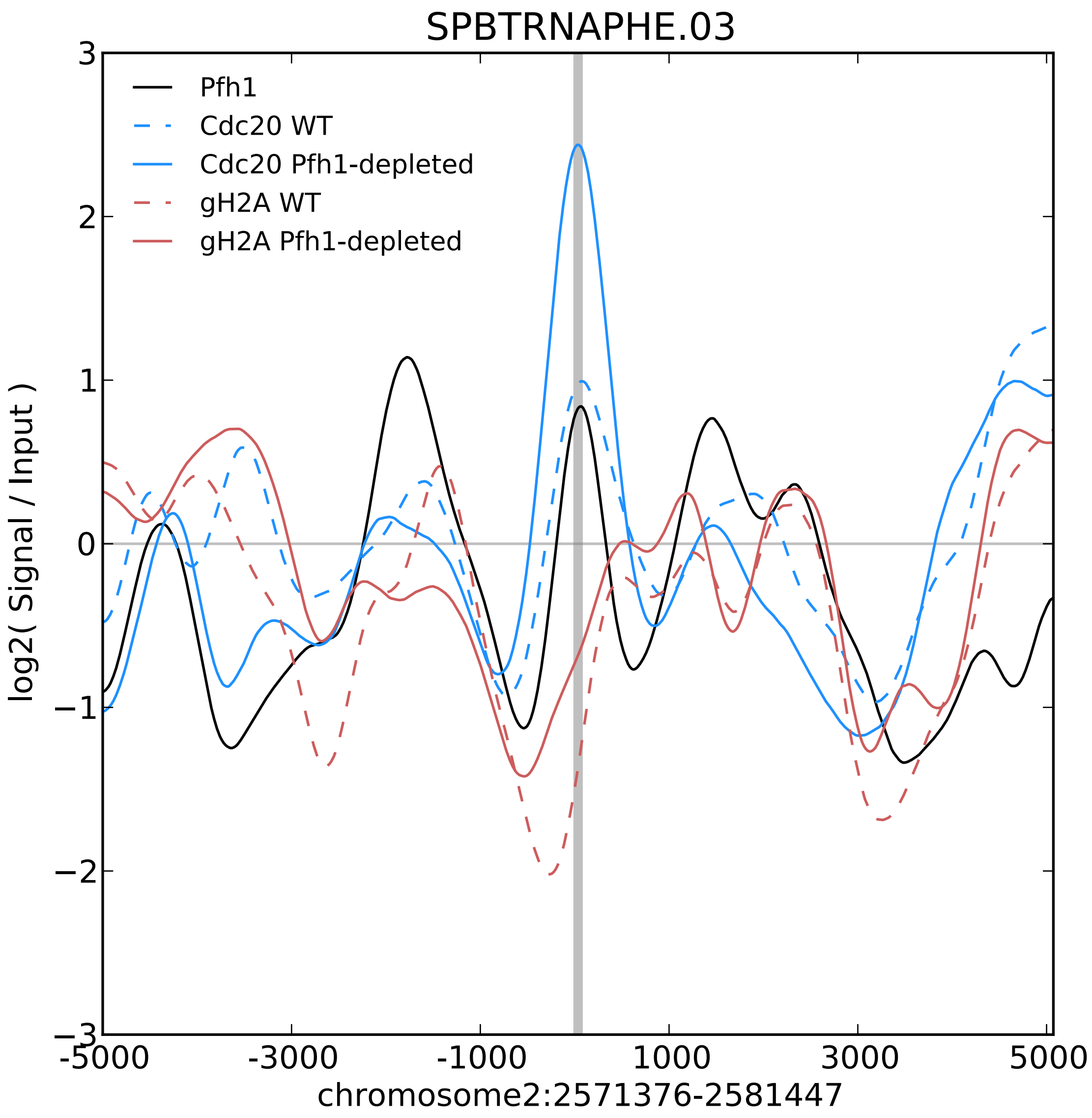
SPBTRNAPHE.03
Pfh1
Cdc20 WT
Cdc20 Pfh1-depleted
gH2A WT
gH2A Pfh1-depleted
log2(Signal / Input)
3
2
1
0
−1
−2
−3
-5000
-3000
-1000
1000
3000
5000
chromosome2:2571376-2581447

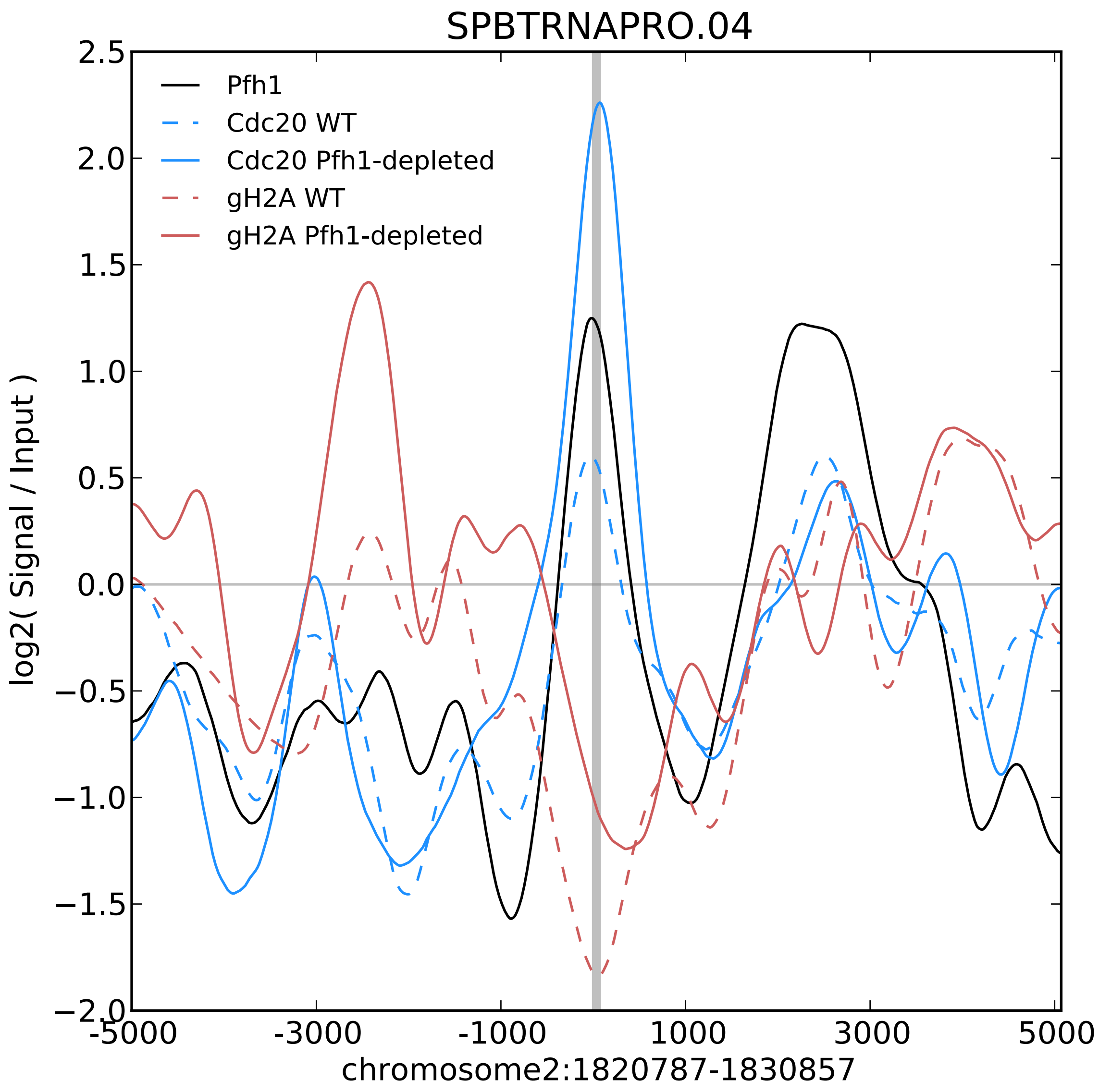
SPBTRNAPRO.04
Pfh1
Cdc20 WT
Cdc20 Pfh1-depleted
gH2A WT
gH2A Pfh1-depleted
log2(Signal / Input)
2.5
2.0
1.5
1.0
0.5
0.0
−0.5
−1.0
−1.5
−2.0
-5000
-3000
-1000
1000
3000
5000
chromosome2:1820787-1830857

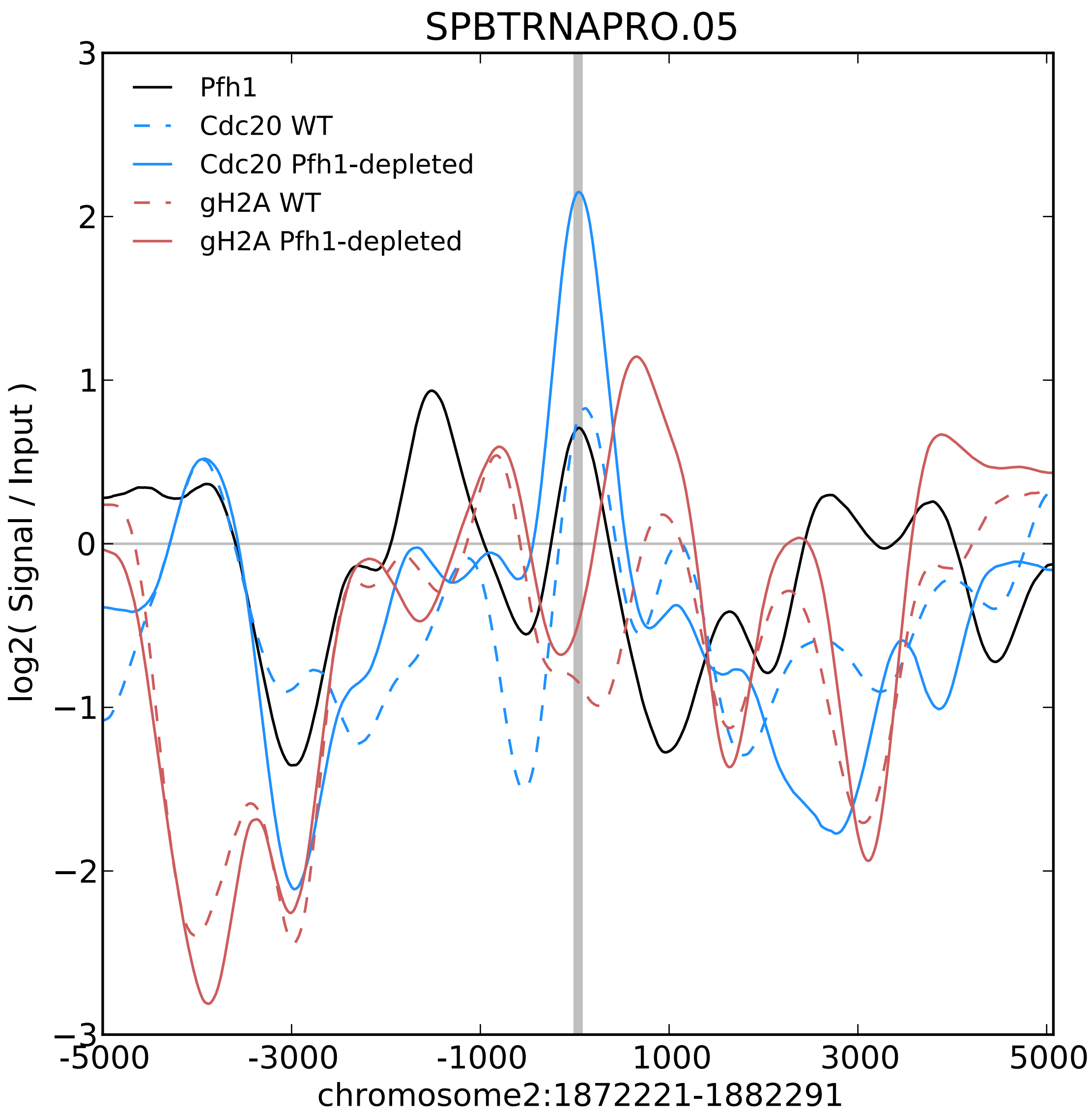

SPBTRNAPRO.05
Pfh1
Cdc20 WT
Cdc20 Pfh1-depleted
gH2A WT
gH2A Pfh1-depleted
log2(Signal / Input)
3
2
1
0
−1
−2
−3
-5000
-3000
-1000
1000
3000
5000
chromosome2:1872221-1882291

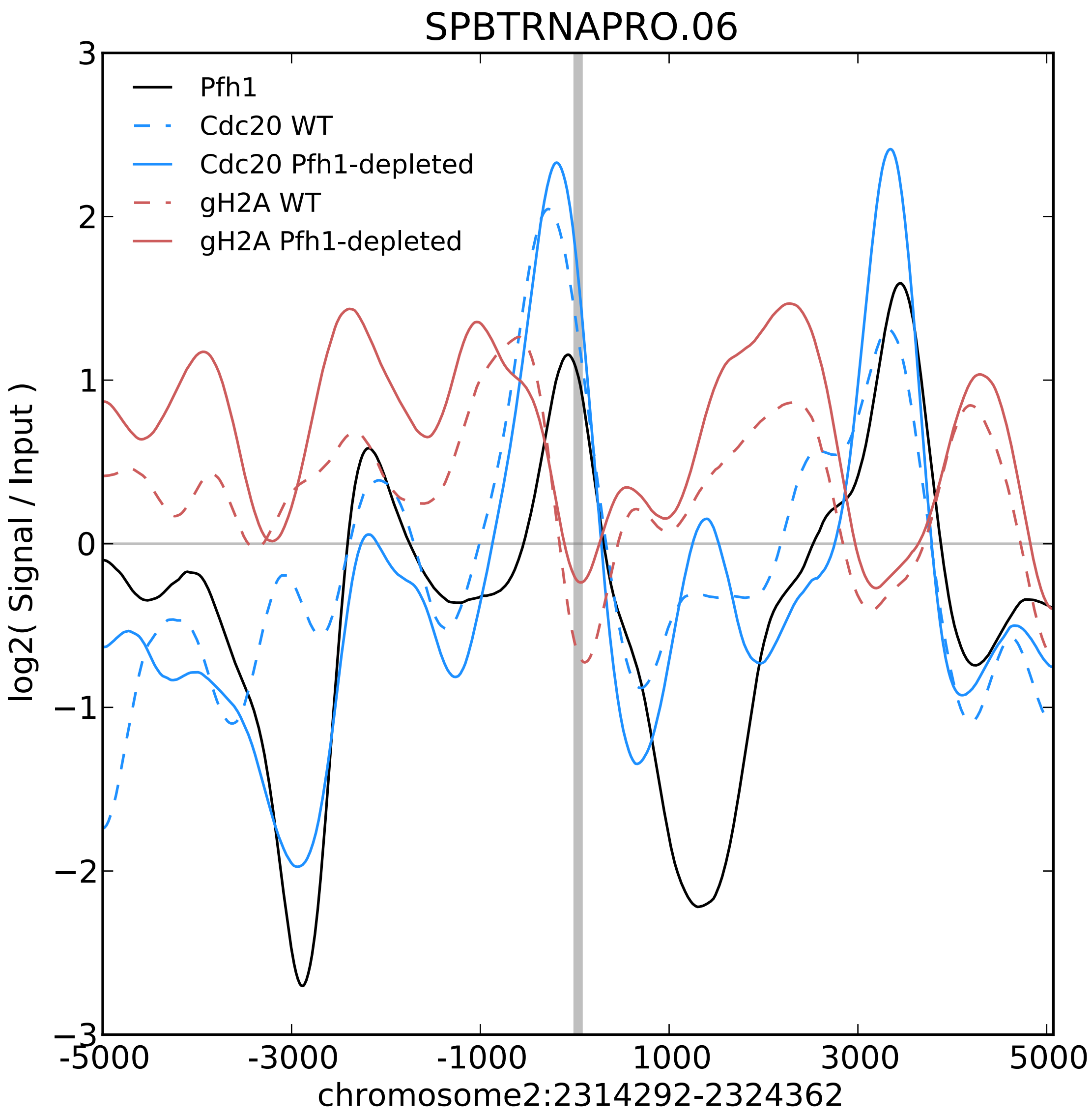
SPBTRNAPRO.06
Pfh1
Cdc20 WT
Cdc20 Pfh1-depleted
gH2A WT
gH2A Pfh1-depleted
log2(Signal / Input)
3
2
1
0
−1
−2
−3
-5000
-3000
-1000
1000
3000
5000
chromosome2:2314292-2324362

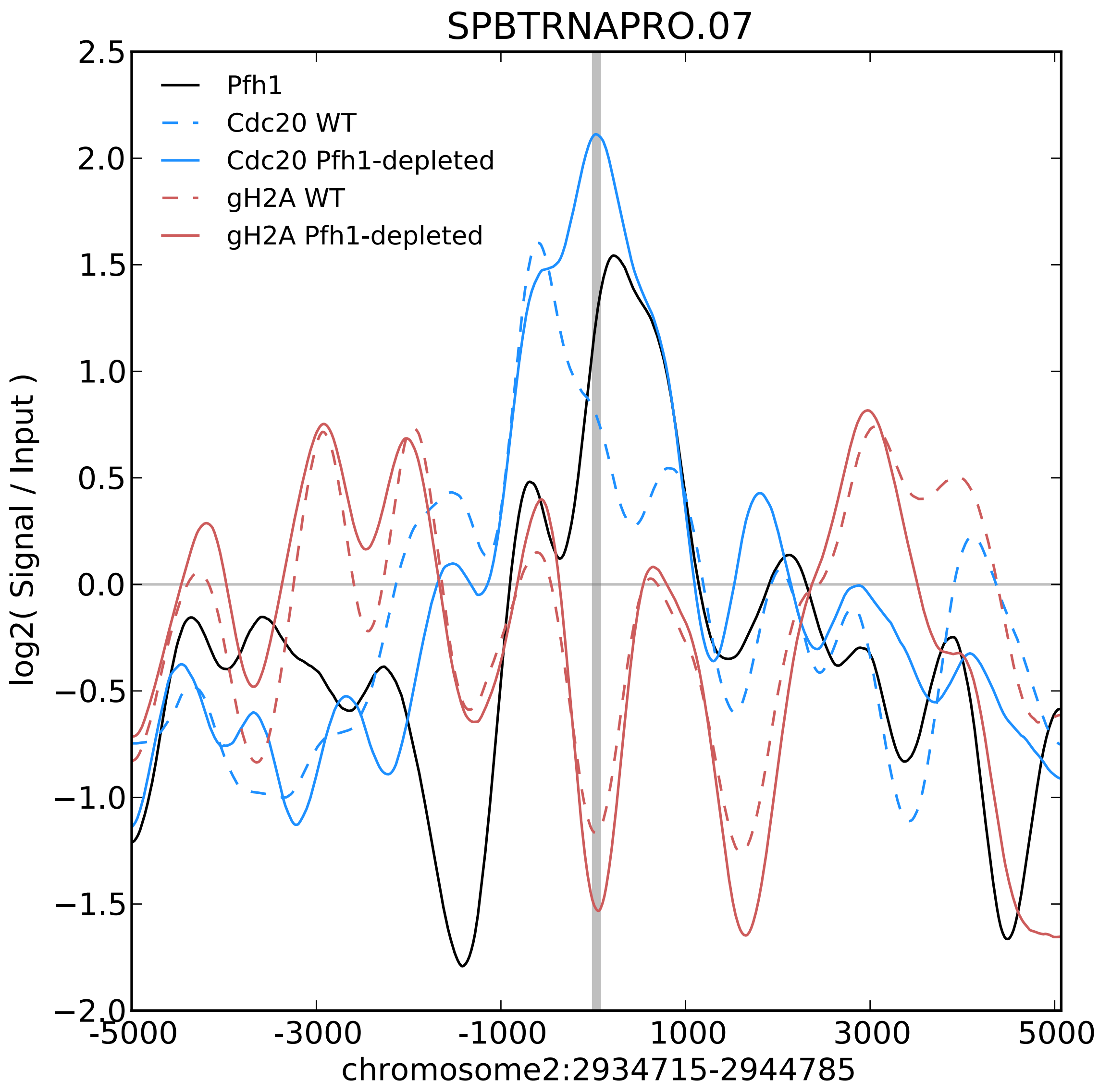

SPBTRNAPRO.07
Pfh1
Cdc20 WT
Cdc20 Pfh1-depleted
gH2A WT
gH2A Pfh1-depleted
log2(Signal / Input)
2.5
2.0
1.5
1.0
0.5
0.0
−0.5
−1.0
−1.5
−2.0
-5000
-3000
-1000
1000
3000
5000
chromosome2:2934715-2944785

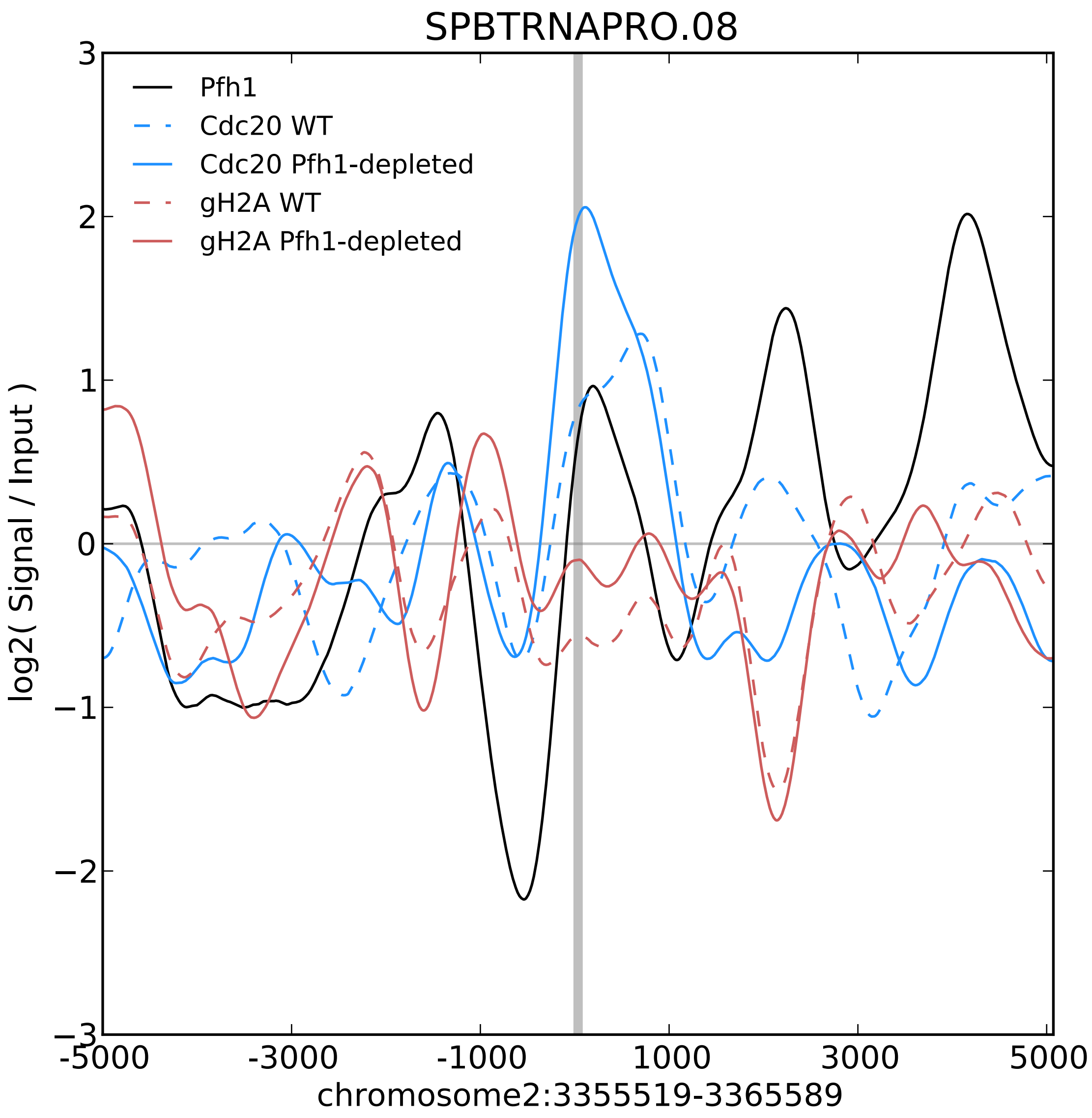

SPBTRNAPRO.08
Pfh1
Cdc20 WT
Cdc20 Pfh1-depleted
gH2A WT
gH2A Pfh1-depleted
log2(Signal / Input)
3
2
1
0
−1
−2
−3
-5000
-3000
-1000
1000
3000
5000
chromosome2:3355519-3365589

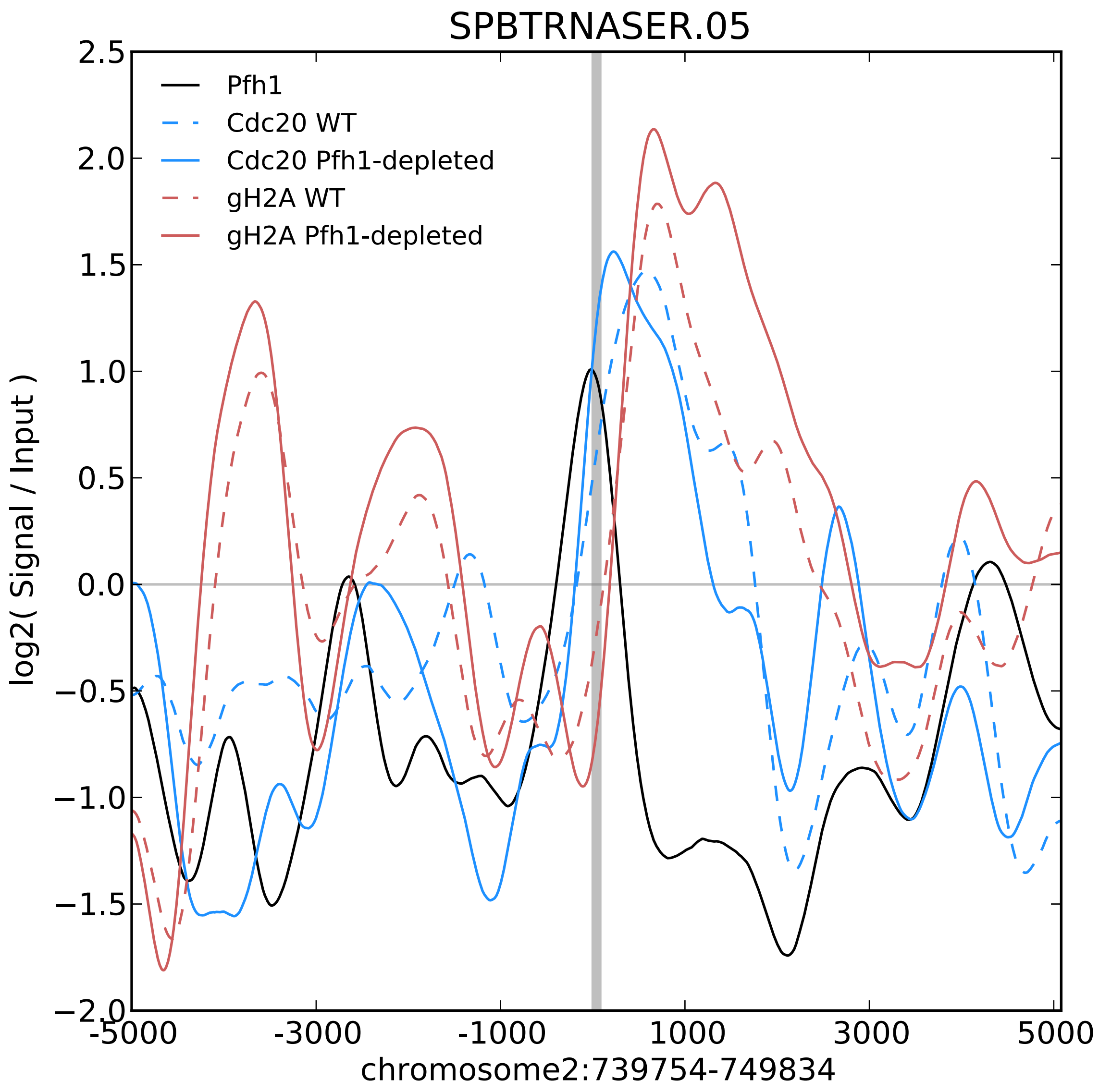
SPBTRNASER.05
Pfh1
Cdc20 WT
Cdc20 Pfh1-depleted
gH2A WT
gH2A Pfh1-depleted
log2(Signal / Input)
2.5
2.0
1.5
1.0
0.5
0.0
−0.5
−1.0
−1.5
−2.0
-5000
-3000
-1000
1000
3000
5000
chromosome2:739754-749834

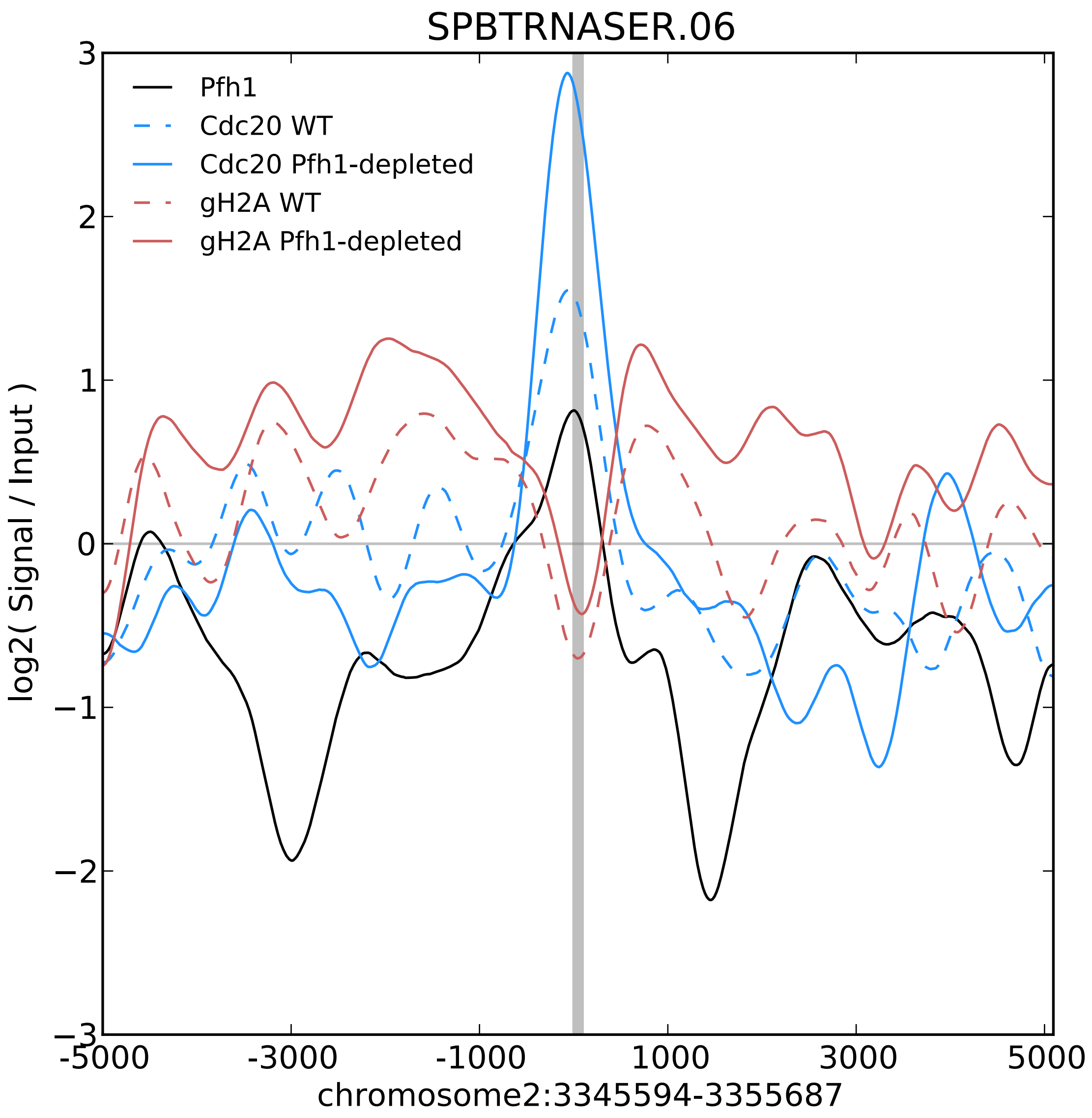

SPBTRNASER.06
Pfh1
Cdc20 WT
Cdc20 Pfh1-depleted
gH2A WT
gH2A Pfh1-depleted
log2(Signal / Input)
3
2
1
0
−1
−2
−3
-5000
-3000
-1000
1000
3000
5000
chromosome2:3345594-3355687

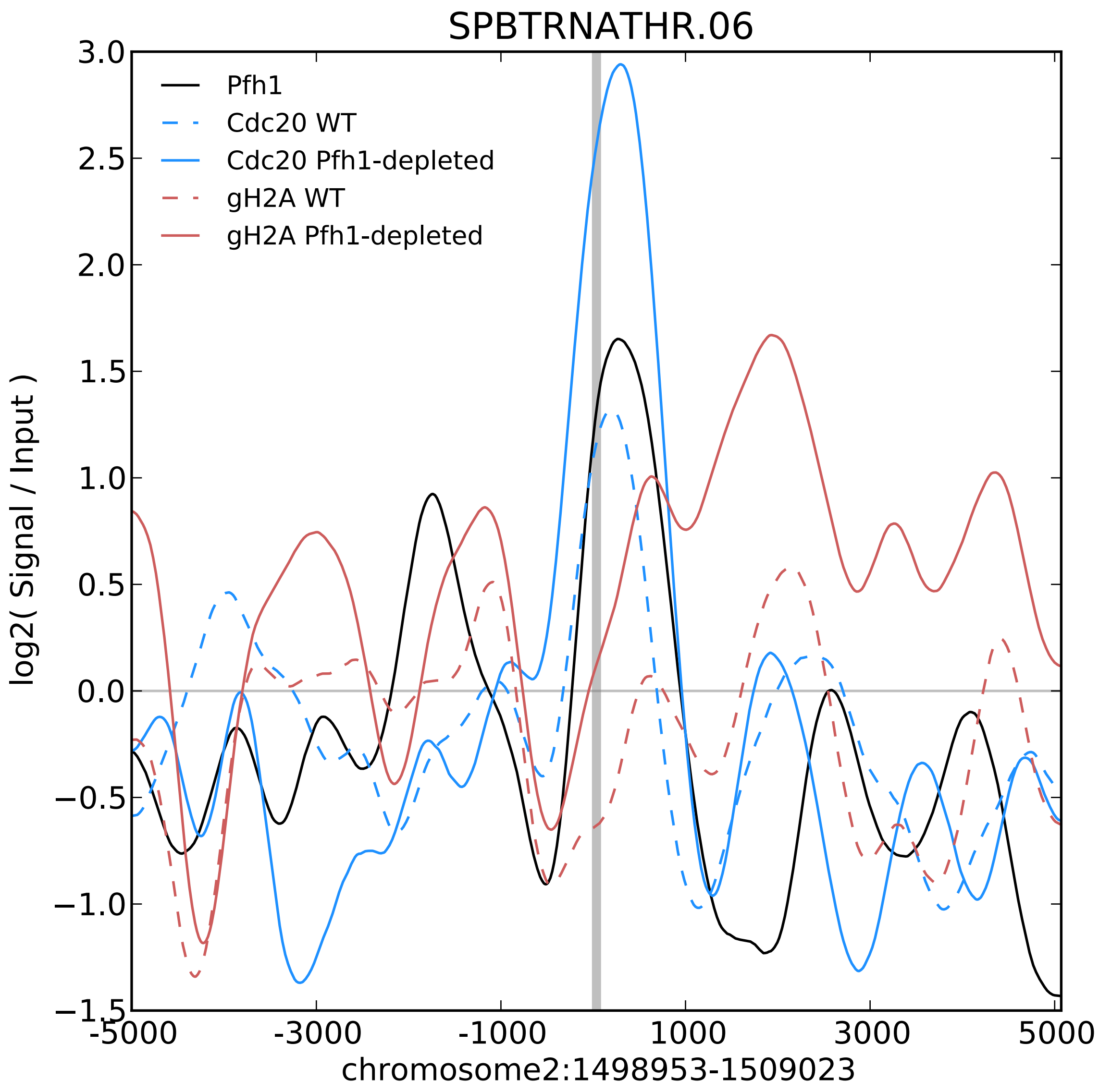

SPBTRNATHR.06
Pfh1
Cdc20 WT
Cdc20 Pfh1-depleted
gH2A WT
gH2A Pfh1-depleted
log2(Signal / Input)
3.0
2.5
2.0
1.5
1.0
0.5
0.0
−0.5
−1.0
−1.5
-5000
-3000
-1000
1000
3000
5000
chromosome2:1498953-1509023

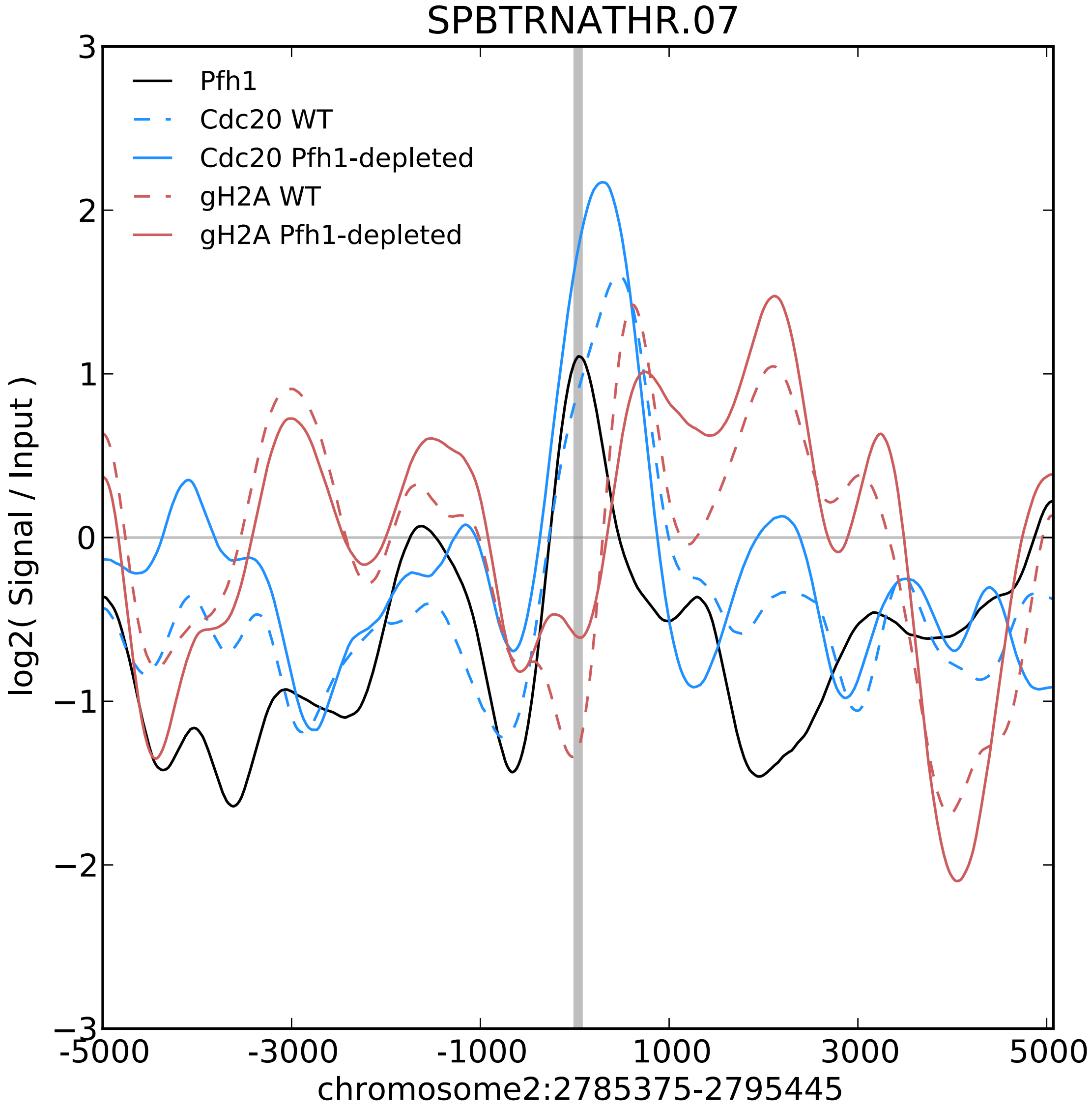

SPBTRNATHR.07
Pfh1
Cdc20 WT
Cdc20 Pfh1-depleted
gH2A WT
gH2A Pfh1-depleted
3
2
1
0
−1
−2
−3
log2(Signal / Input)
-5000
-3000
-1000
1000
3000
5000
chromosome2:2785375-2795445

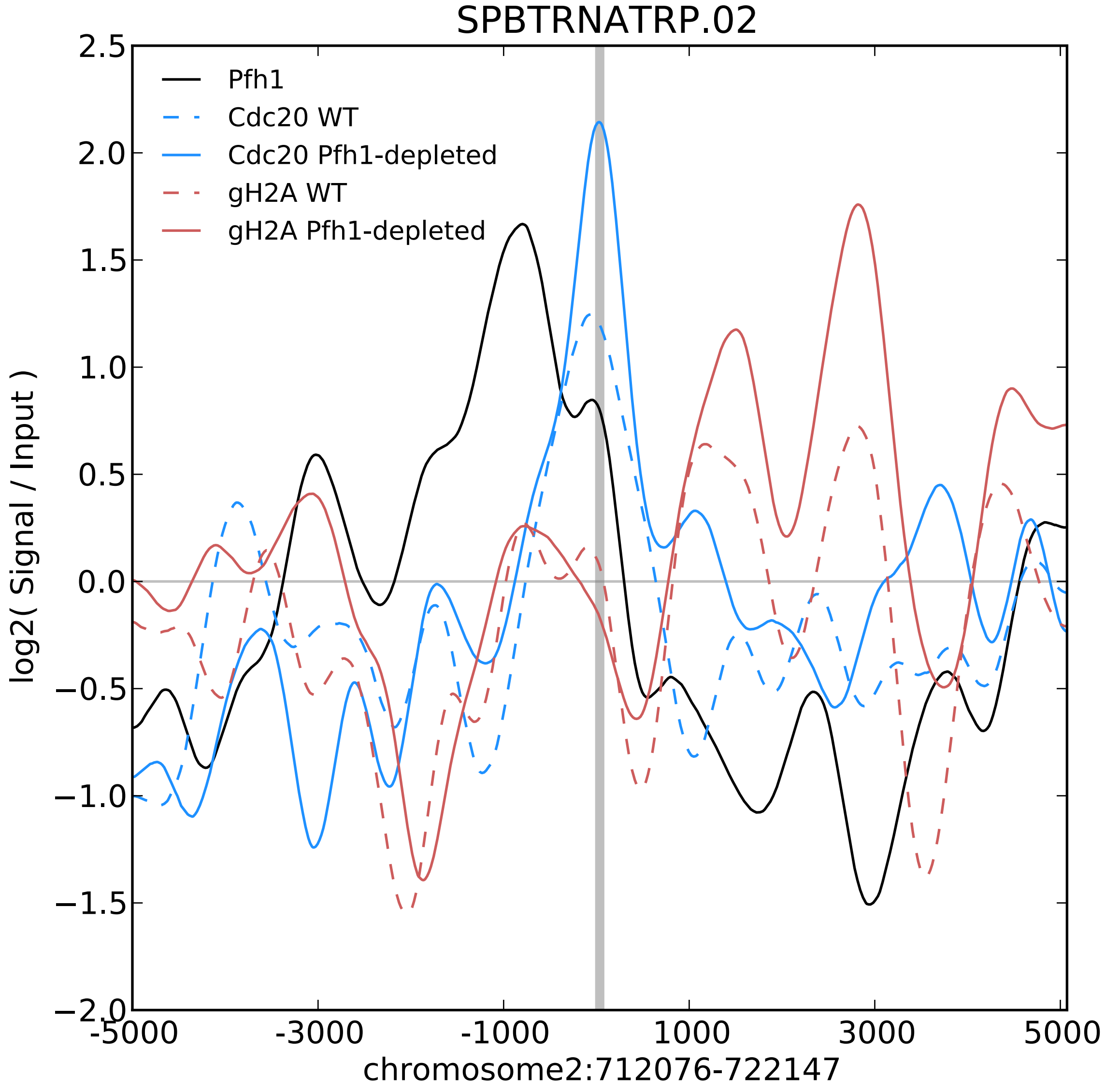
SPBTRNATRP.02
Pfh1
Cdc20 WT
Cdc20 Pfh1-depleted
gH2A WT
gH2A Pfh1-depleted
log2(Signal / Input)
2.5
2.0
1.5
1.0
0.5
0.0
−0.5
−1.0
−1.5
−2.0
-5000
-3000
-1000
1000
3000
5000
chromosome2:712076-722147

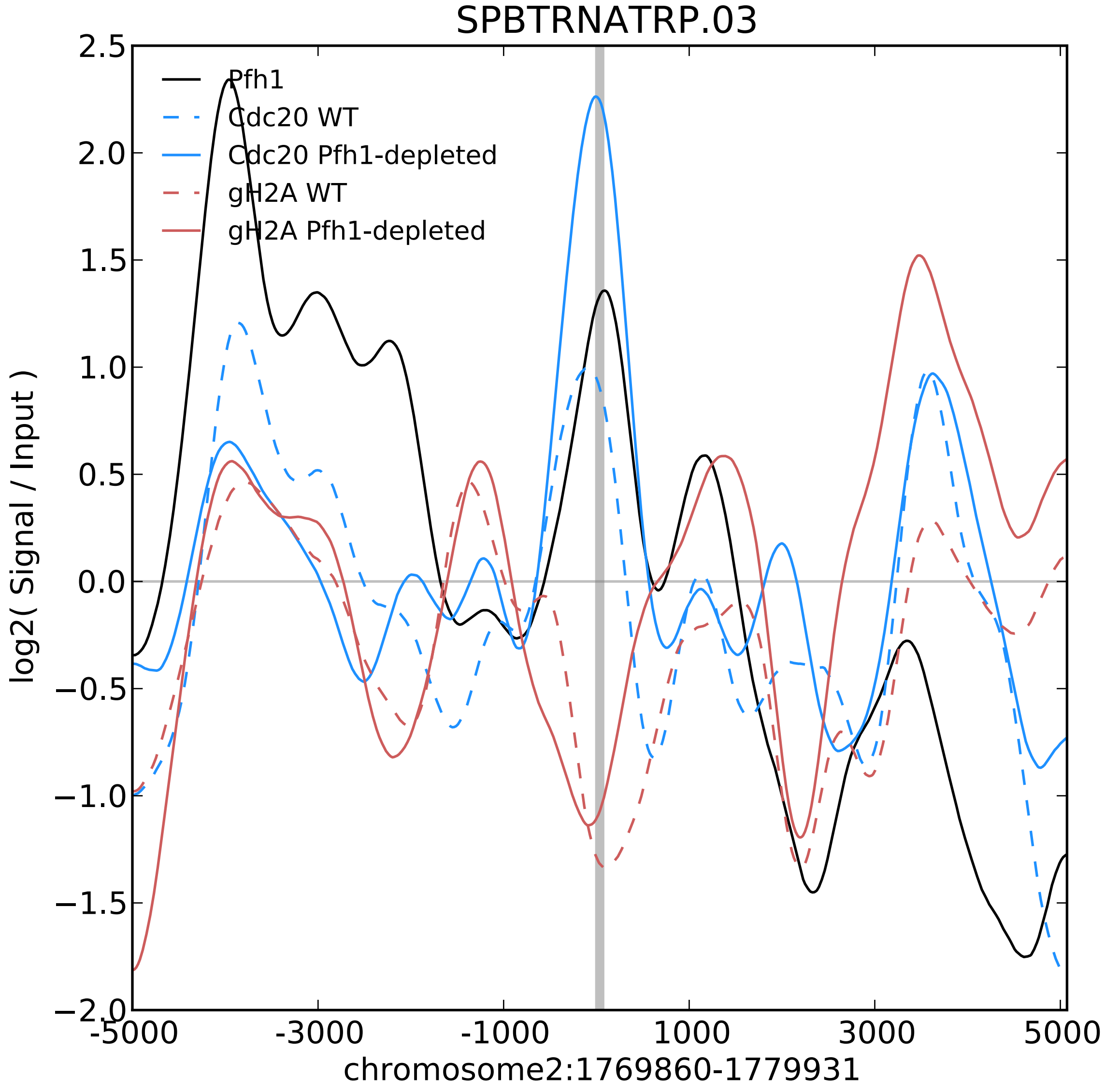

SPBTRNATRP.03
Pfh1
Cdc20 WT
Cdc20 Pfh1-depleted
gH2A WT
gH2A Pfh1-depleted
log2(Signal / Input)
chromosome2:1769860-1779931
2.5
2.0
1.5
1.0
0.5
0.0
−0.5
−1.0
−1.5
−2.0
-5000
-3000
-1000
1000
3000
5000

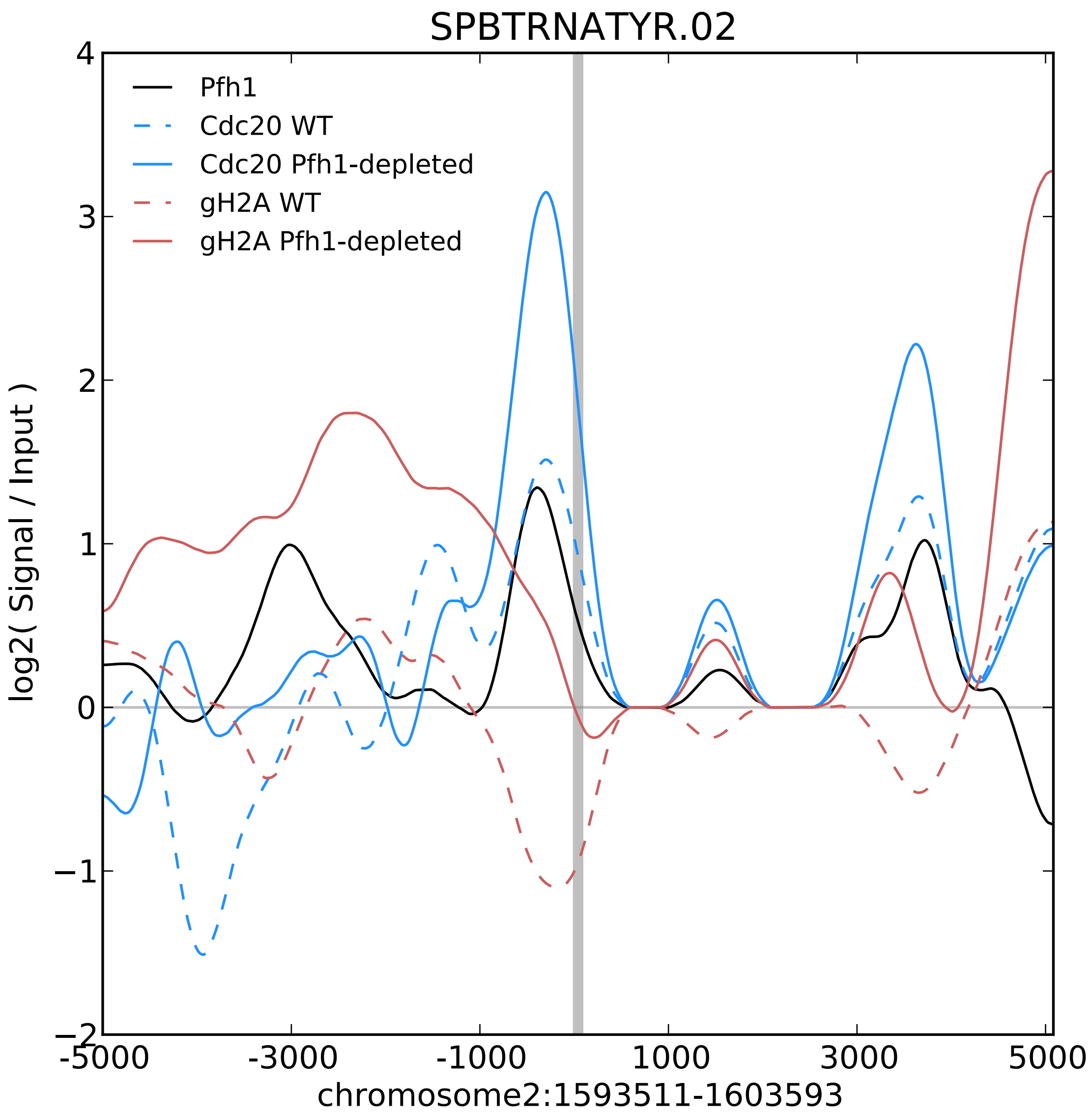
SPBTRNATYR.02
Pfh1
Cdc20 WT
Cdc20 Pfh1-depleted
gH2A WT
gH2A Pfh1-depleted
log2(Signal / Input)
4
3
2
1
0
−1
−2
-5000
-3000
-1000
1000
3000
5000
chromosome2:1593511-1603593

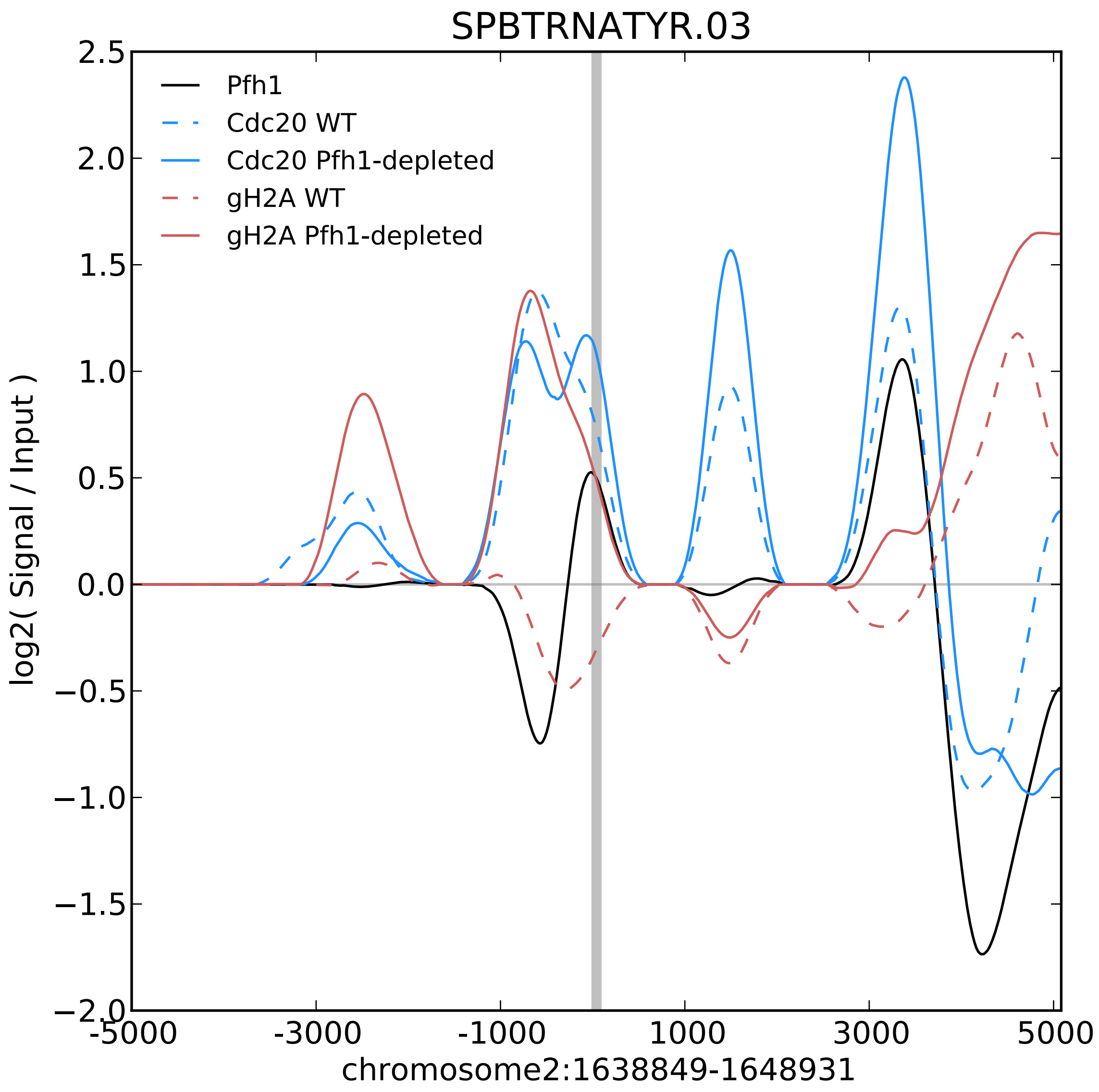
SPBTRNATYR.03
Pfh1
Cdc20 WT
Cdc20 Pfh1-depleted
gH2A WT
gH2A Pfh1-depleted
log2(Signal / Input)
2.5
2.0
1.5
1.0
0.5
0.0
−0.5
−1.0
−1.5
−2.0
-5000
-3000
-1000
1000
3000
5000
chromosome2:1638849-1648931

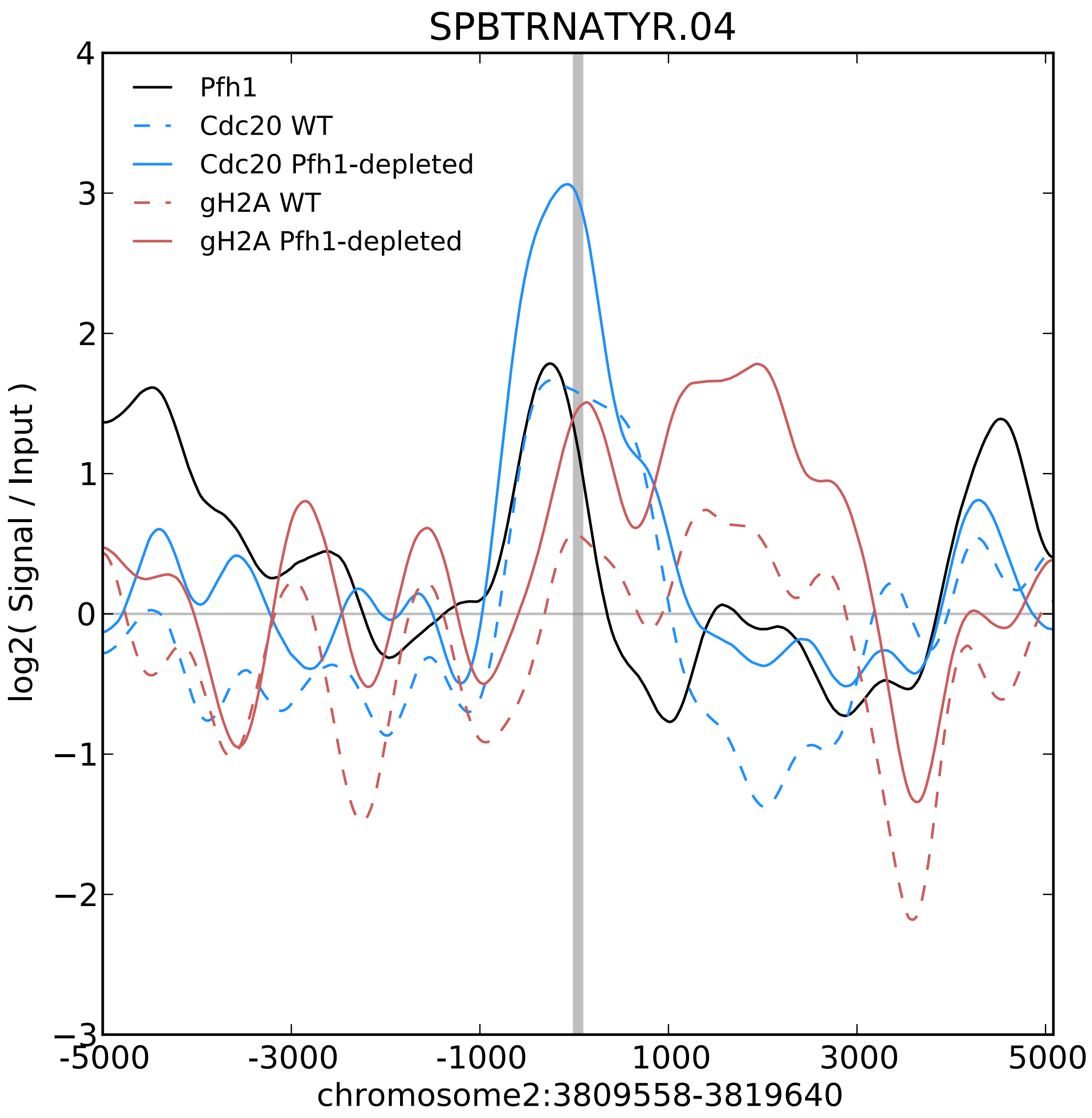

SPBTRNATYR.04
Pfh1
Cdc20 WT
Cdc20 Pfh1-depleted
gH2A WT
gH2A Pfh1-depleted
log2(Signal / Input)
4
3
2
1
0
−1
−2
−3
-5000
-3000
-1000
1000
3000
5000
chromosome2:3809558-3819640

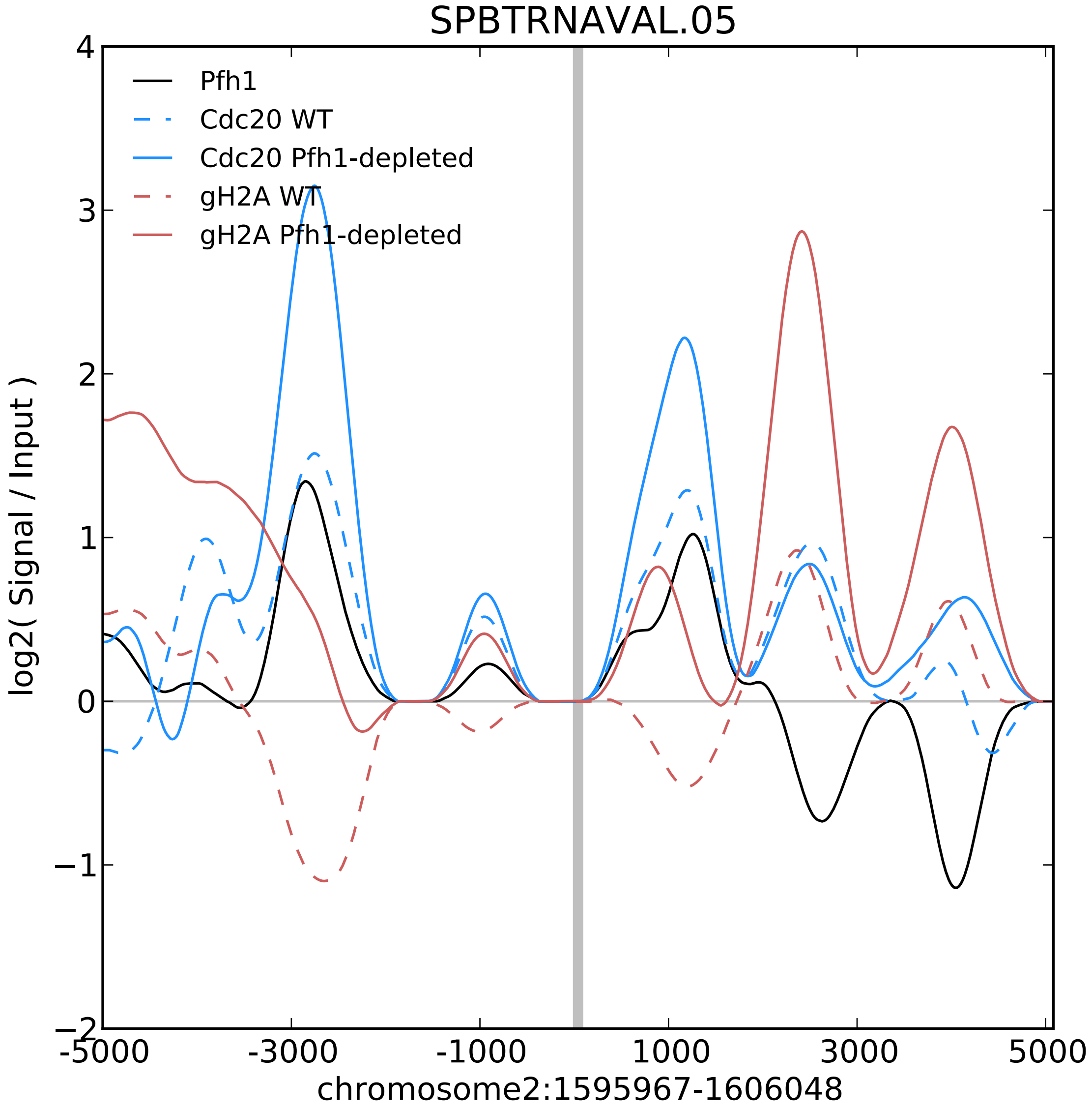
SPBTRNAVAL.05
Pfh1
Cdc20 WT
Cdc20 Pfh1-depleted
gH2A WT
gH2A Pfh1-depleted
log2(Signal / Input)
4
3
2
1
0
−1
−2
-5000
-3000
-1000
1000
3000
5000
chromosome2:1595967-1606048

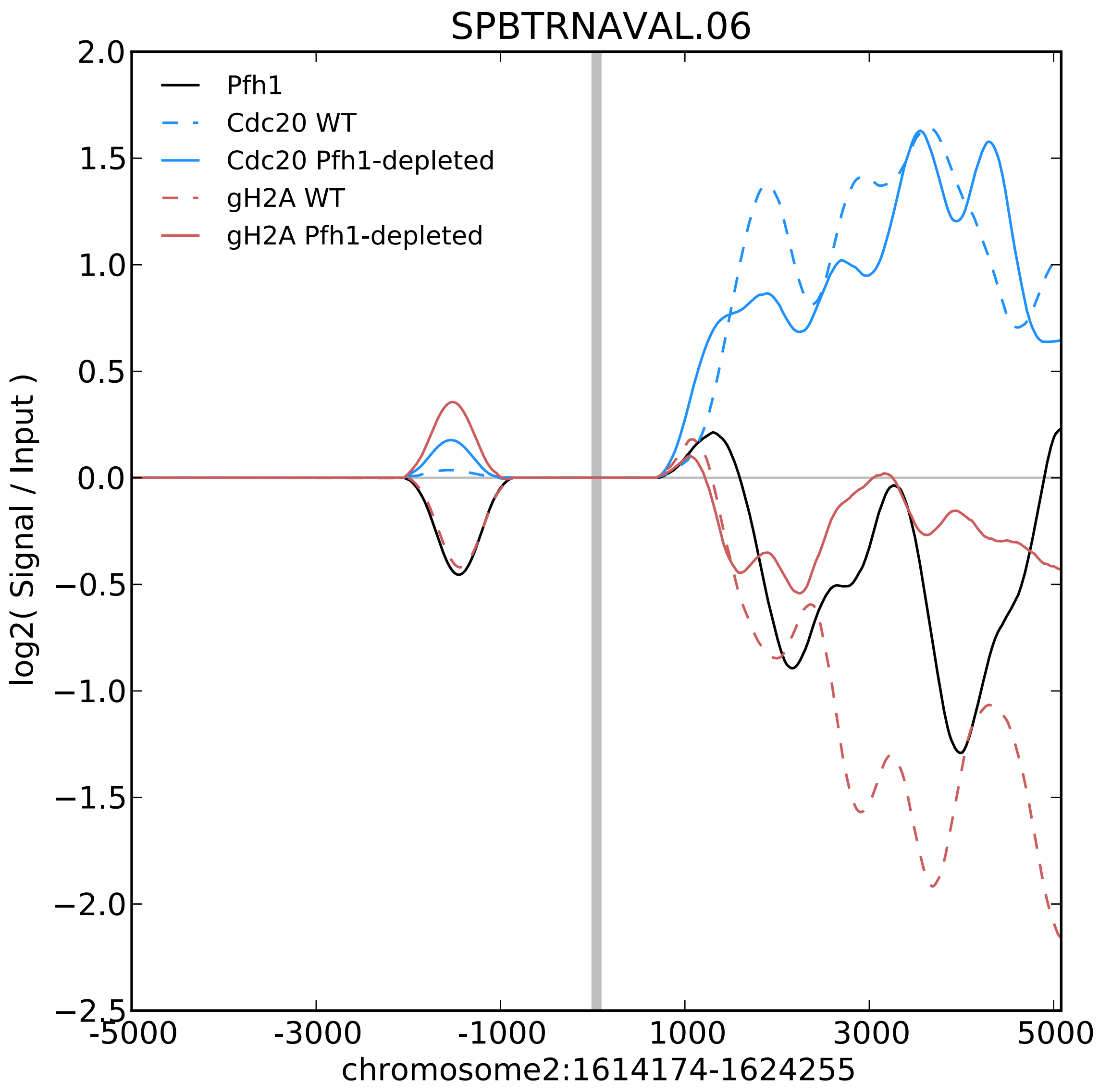

SPBTRNAVAL.06
Pfh1
Cdc20 WT
Cdc20 Pfh1-depleted
gH2A WT
gH2A Pfh1-depleted
log2(Signal / Input)
2.0
1.5
1.0
0.5
0.0
−0.5
−1.0
−1.5
−2.0
−2.5
-5000
-3000
-1000
1000
3000
5000
chromosome2:1614174-1624255

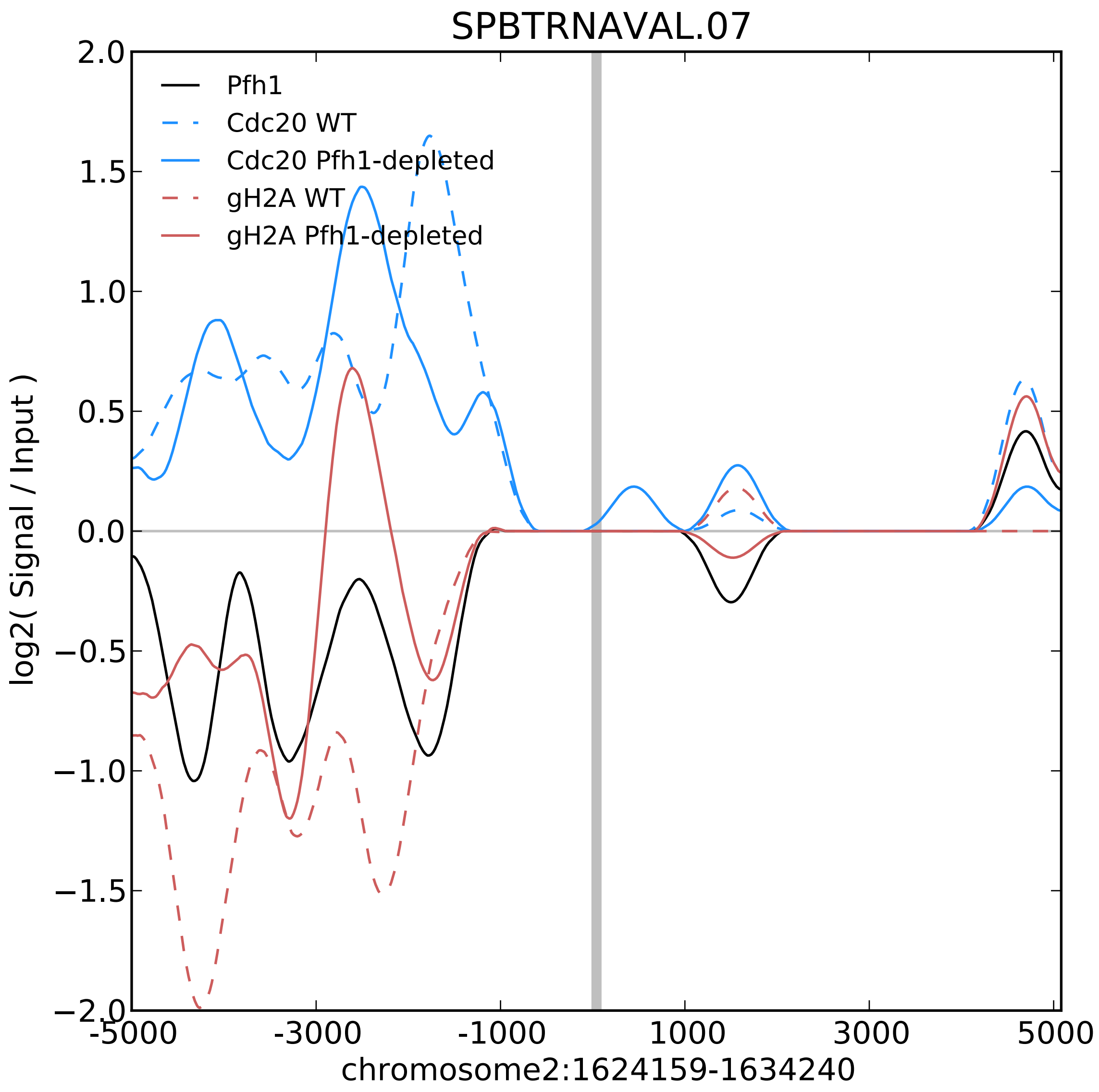
SPBTRNAVAL.07
Pfh1
Cdc20 WT
Cdc20 Pfh1-depleted
gH2A WT
gH2A Pfh1-depleted
2.0
1.5
1.0
0.5
0.0
−0.5
−1.0
−1.5
−2.0
log2(Signal / Input)
-5000
-3000
-1000
1000
3000
5000
chromosome2:1624159-1634240

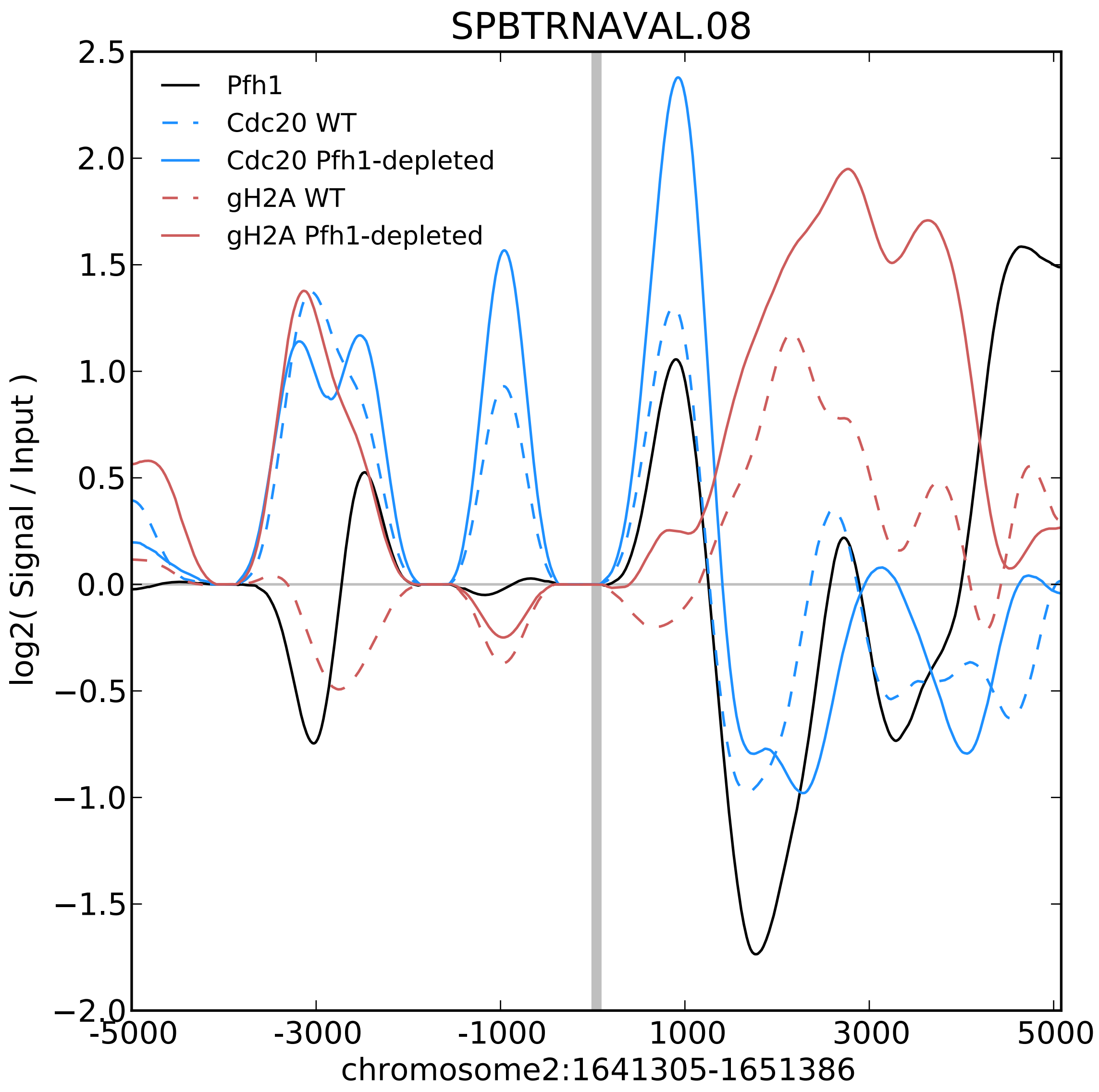

SPBTRNAVAL.08
Pfh1
Cdc20 WT
Cdc20 Pfh1-depleted
gH2A WT
gH2A Pfh1-depleted
2.5
2.0
1.5
1.0
0.5
0.0
−0.5
−1.0
−1.5
−2.0
log2(Signal / Input)
-5000
-3000
-1000
1000
3000
5000
chromosome2:1641305-1651386

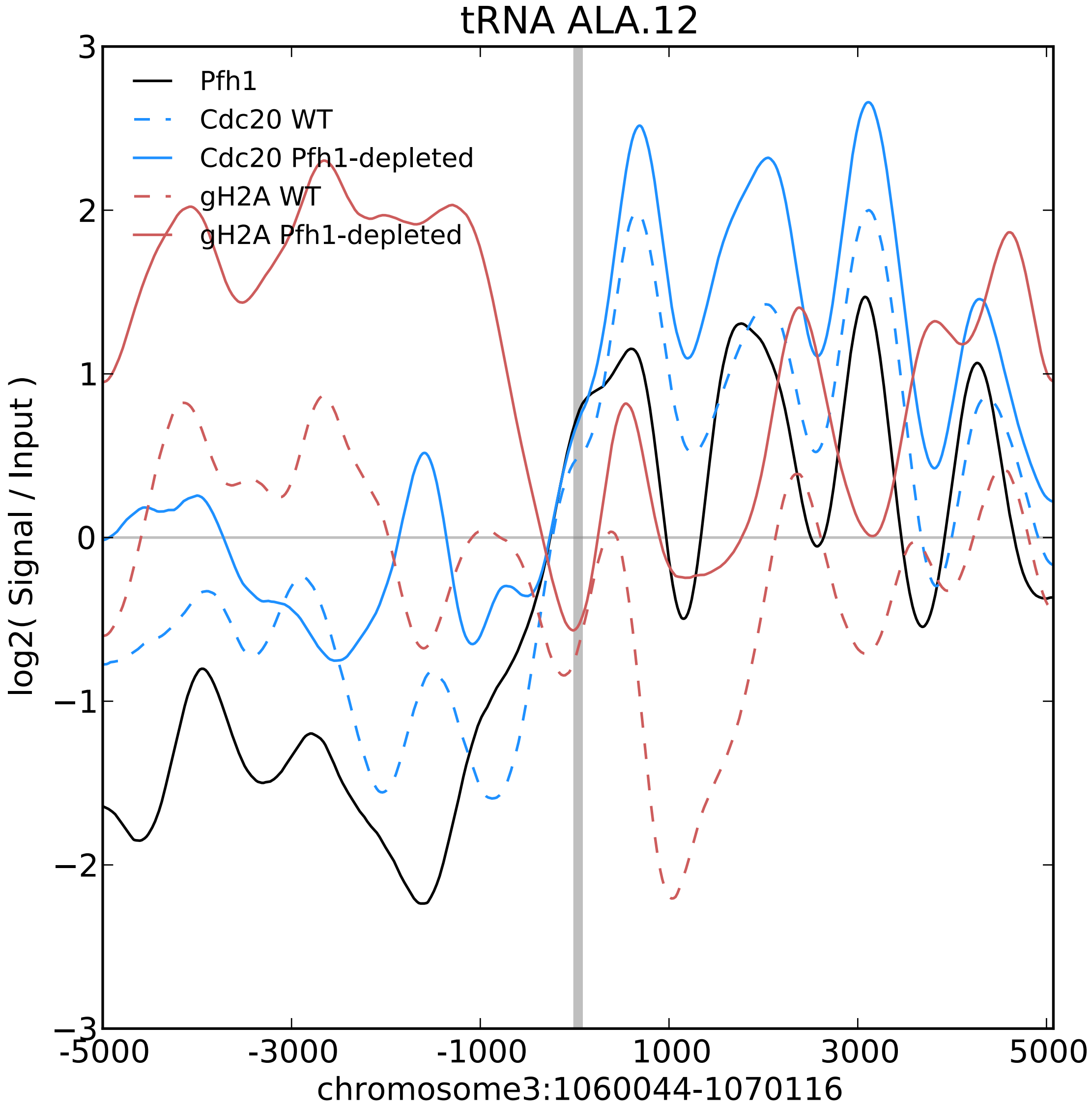

tRNA ALA.12
Pfh1
Cdc20 WT
Cdc20 Pfh1-depleted
gH2A WT
gH2A Pfh1-depleted
3
2
1
0
−1
−2
−3
log2(Signal / Input)
-5000
-3000
-1000
1000
3000
5000
chromosome3:1060044-1070116

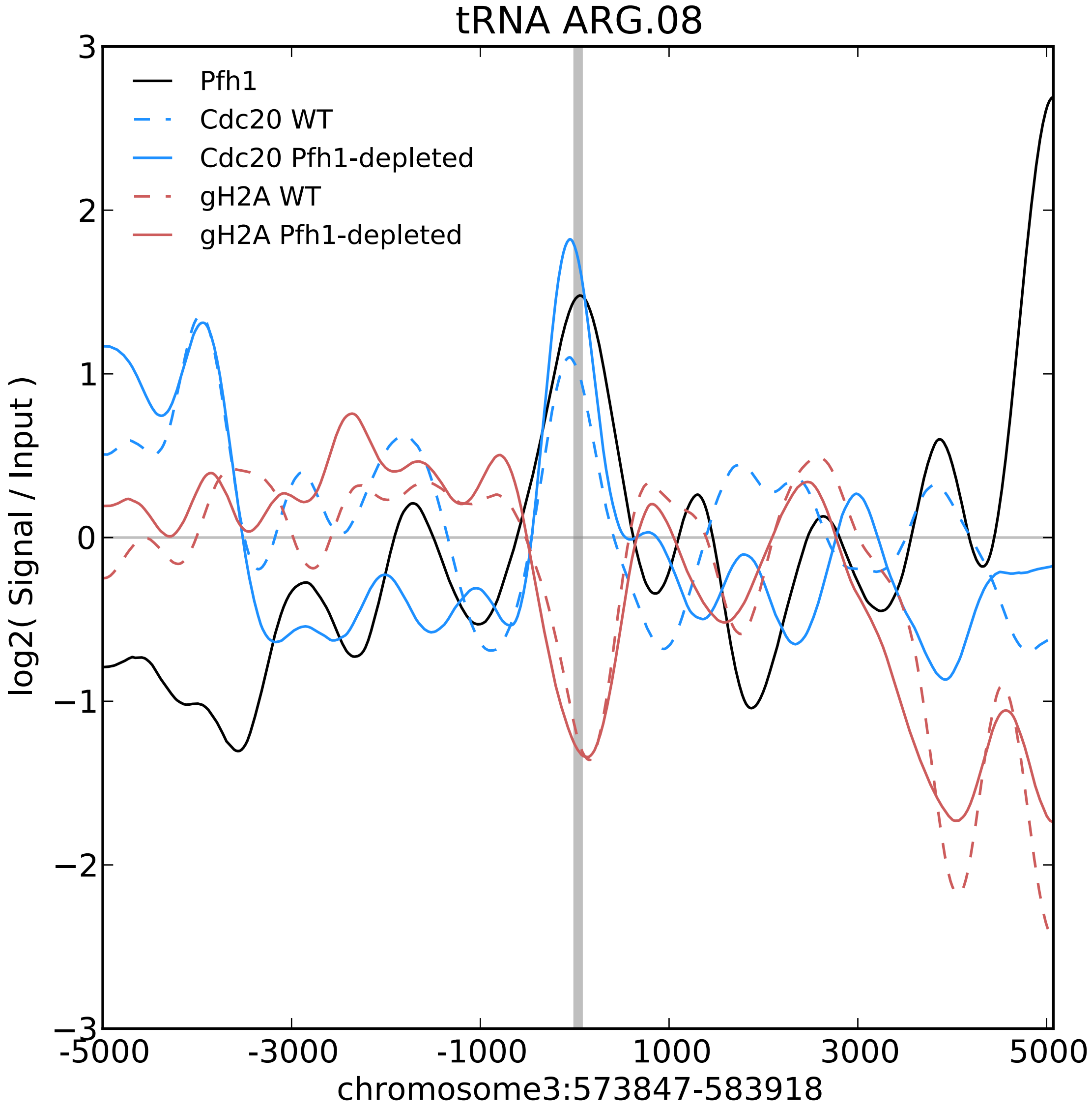

tRNA ARG.08
Pfh1
Cdc20 WT
Cdc20 Pfh1-depleted
gH2A WT
gH2A Pfh1-depleted
log2(Signal / Input)
3
2
1
0
−1
−2
−3
-5000
-3000
-1000
1000
3000
5000
chromosome3:573847-583918

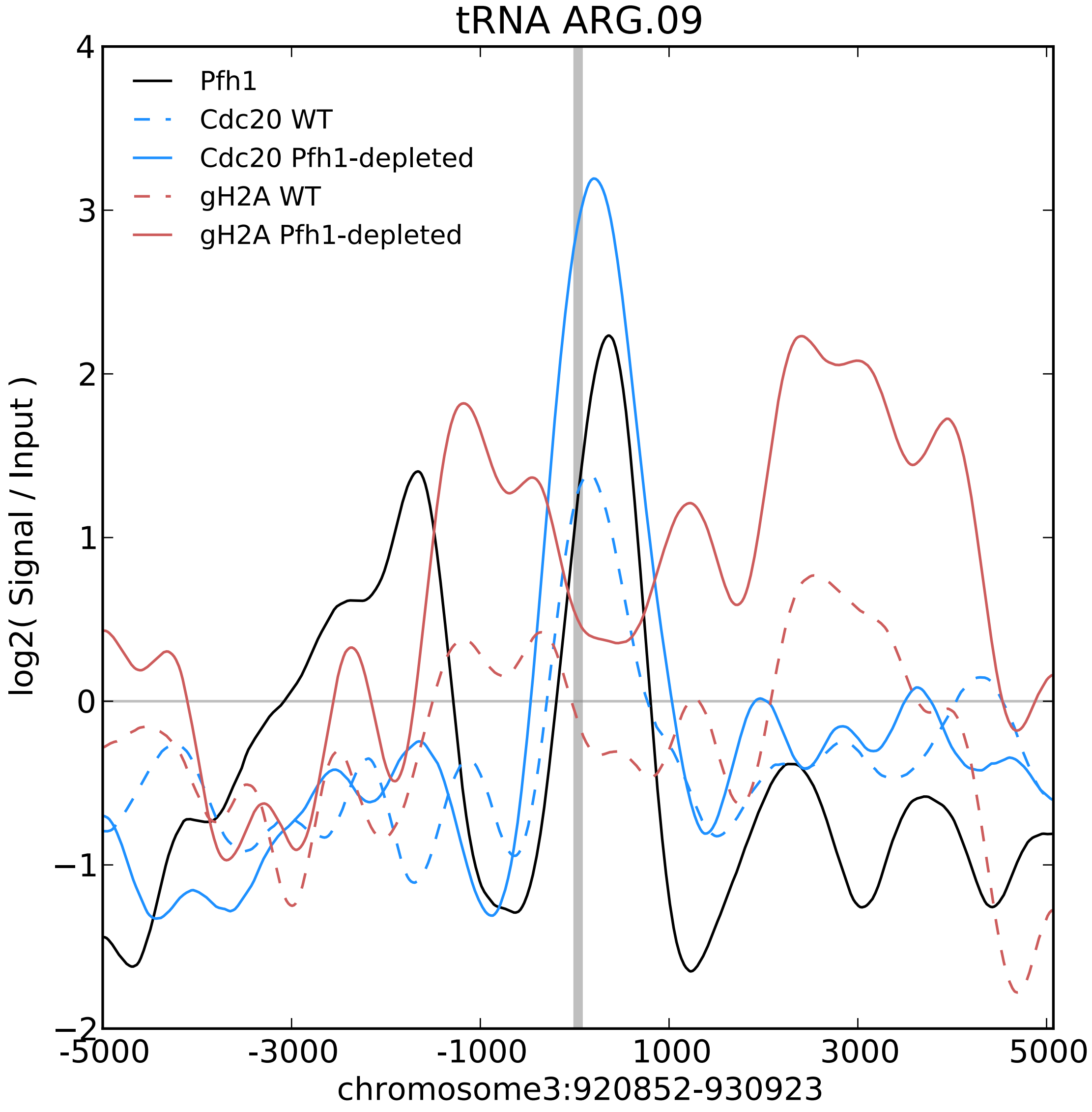

tRNA ARG.09
Pfh1
Cdc20 WT
Cdc20 Pfh1-depleted
gH2A WT
gH2A Pfh1-depleted
log2(Signal / Input)
4
3
2
1
0
−1
−2
-5000
-3000
-1000
1000
3000
5000
chromosome3:920852-930923

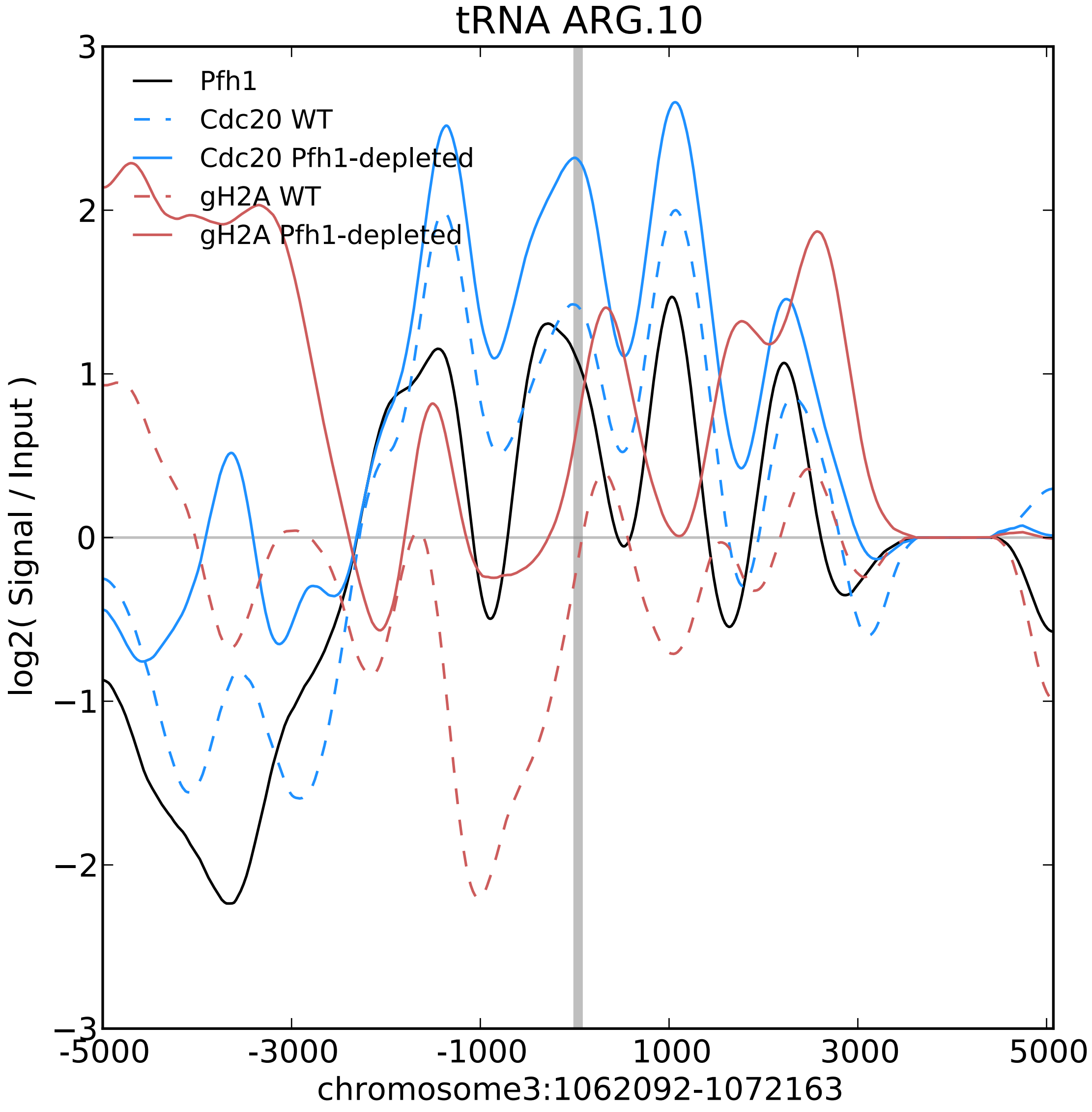

tRNA ARG.10
Pfh1
Cdc20 WT
Cdc20 Pfh1-depleted
gH2A WT
gH2A Pfh1-depleted
log2(Signal / Input)
3
2
1
0
−1
−2
−3
-5000
-3000
-1000
1000
3000
5000
chromosome3:1062092-1072163

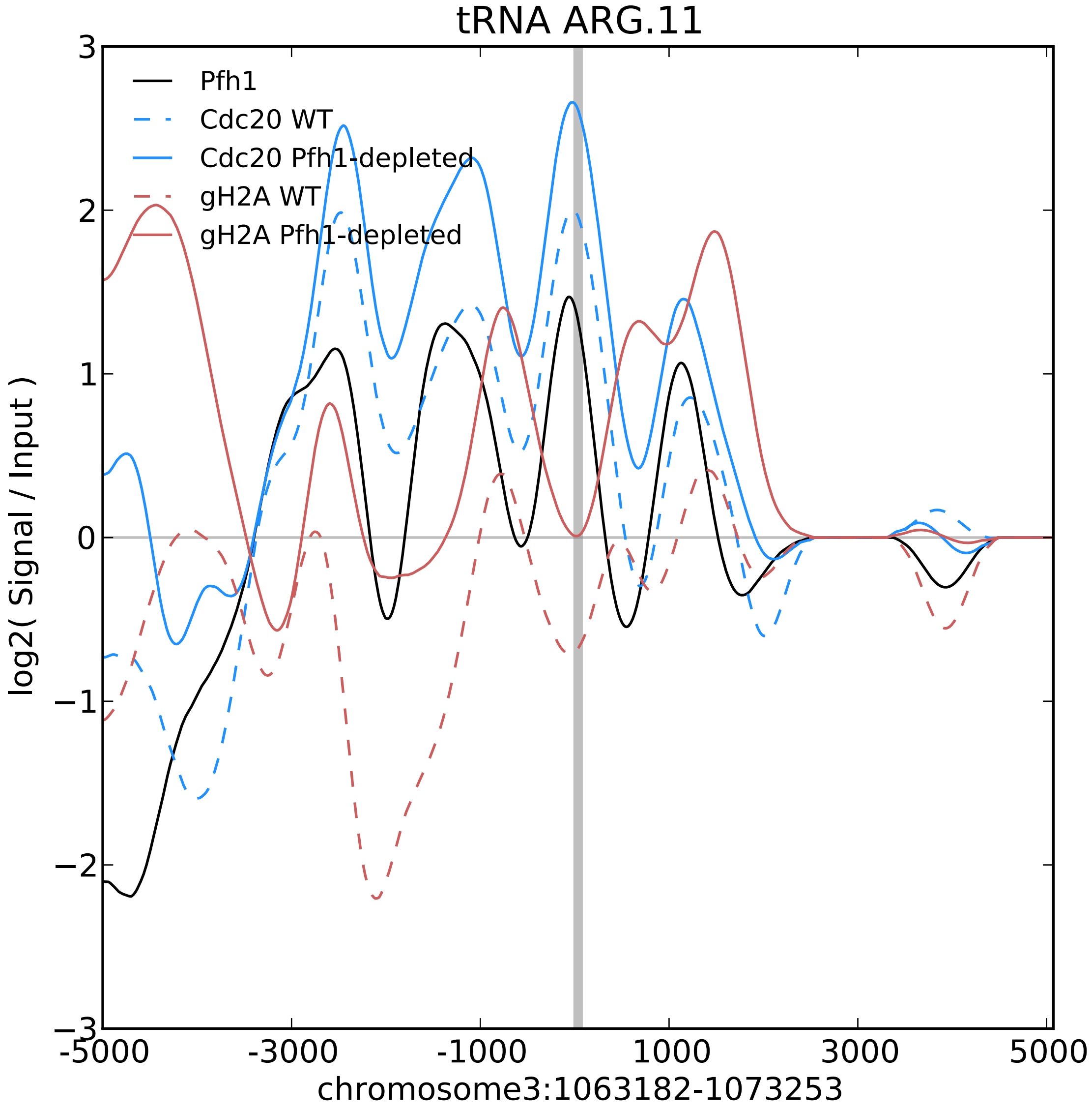
tRNA ARG.11
Pfh1
Cdc20 WT
Cdc20 Pfh1-depleted
gH2A WT
gH2A Pfh1-depleted
log2(Signal / Input)
3
2
1
0
−1
−2
−3
-5000
-3000
-1000
1000
3000
5000
chromosome3:1063182-1073253

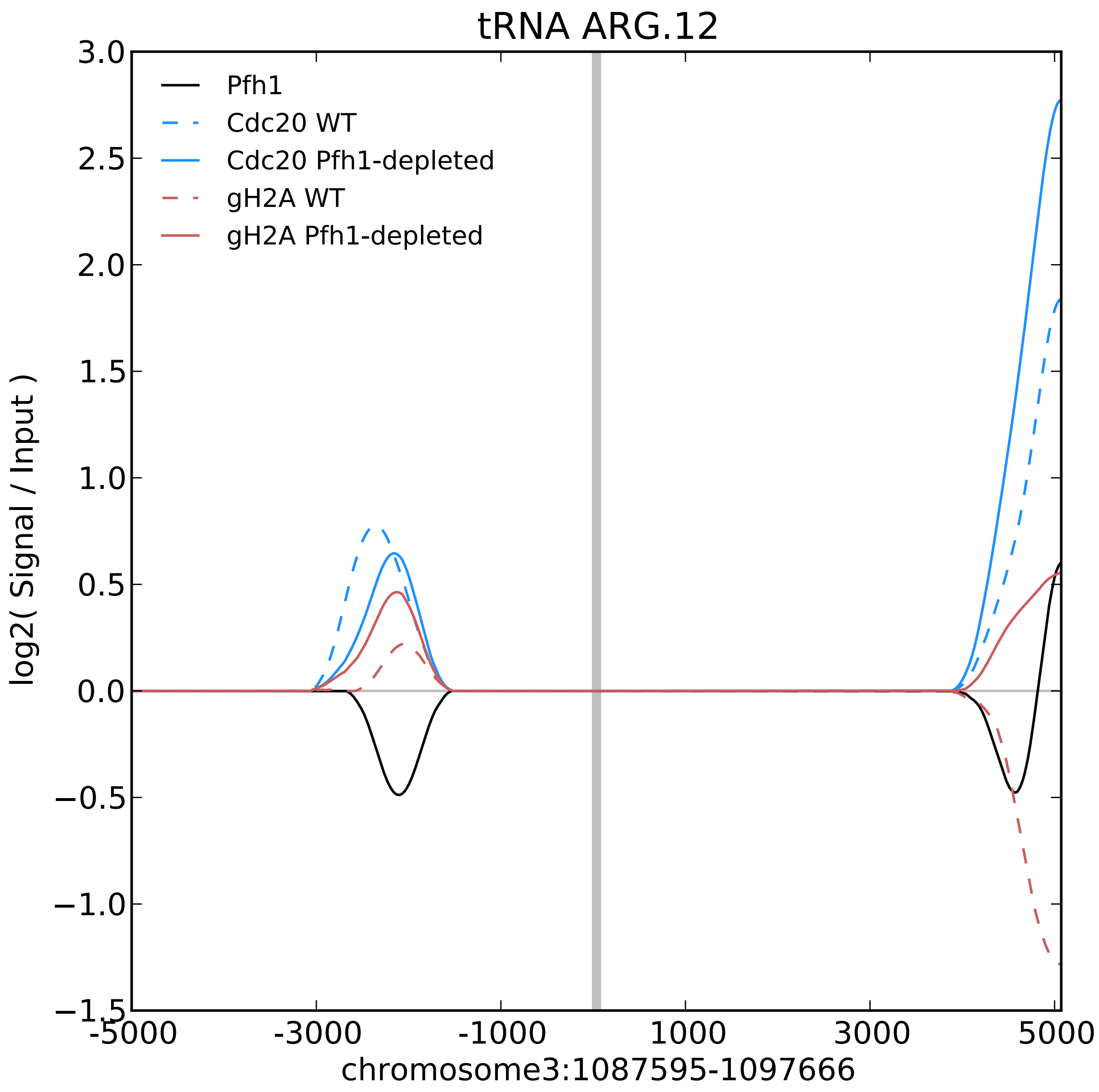
tRNA ARG.12
Pfh1
Cdc20 WT
Cdc20 Pfh1-depleted
gH2A WT
gH2A Pfh1-depleted
log2(Signal / Input)
3.0
2.5
2.0
1.5
1.0
0.5
0.0
−0.5
−1.0
−1.5
-5000
-3000
-1000
1000
3000
5000
chromosome3:1087595-1097666

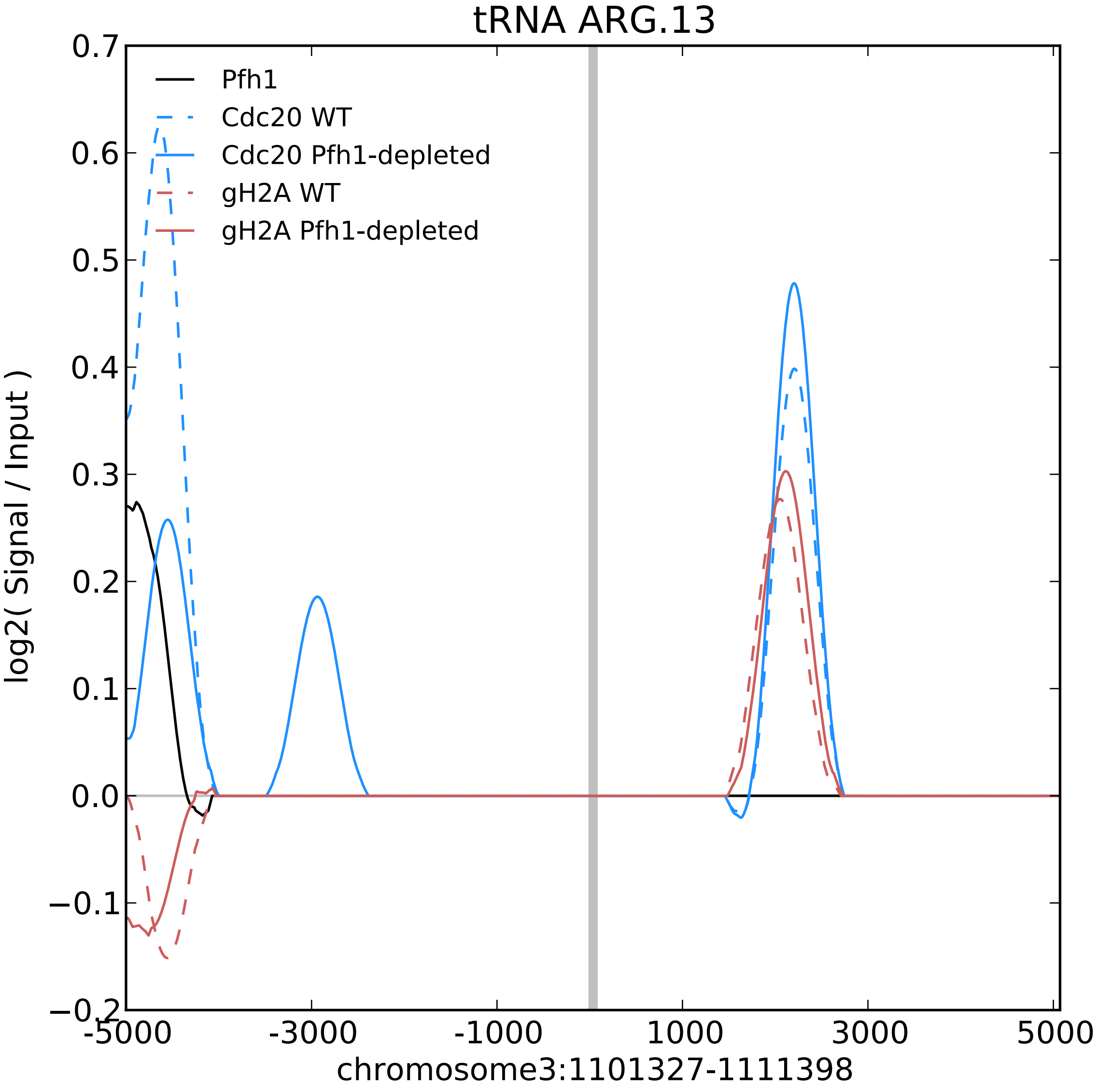
tRNA ARG.13
Pfh1
Cdc20 WT
Cdc20 Pfh1-depleted
gH2A WT
gH2A Pfh1-depleted
0.7
0.6
0.5
0.4
0.3
0.2
0.1
0.0
−0.1
−0.2
log2(Signal / Input)
-5000
-3000
-1000
1000
3000
5000
chromosome3:1101327-1111398

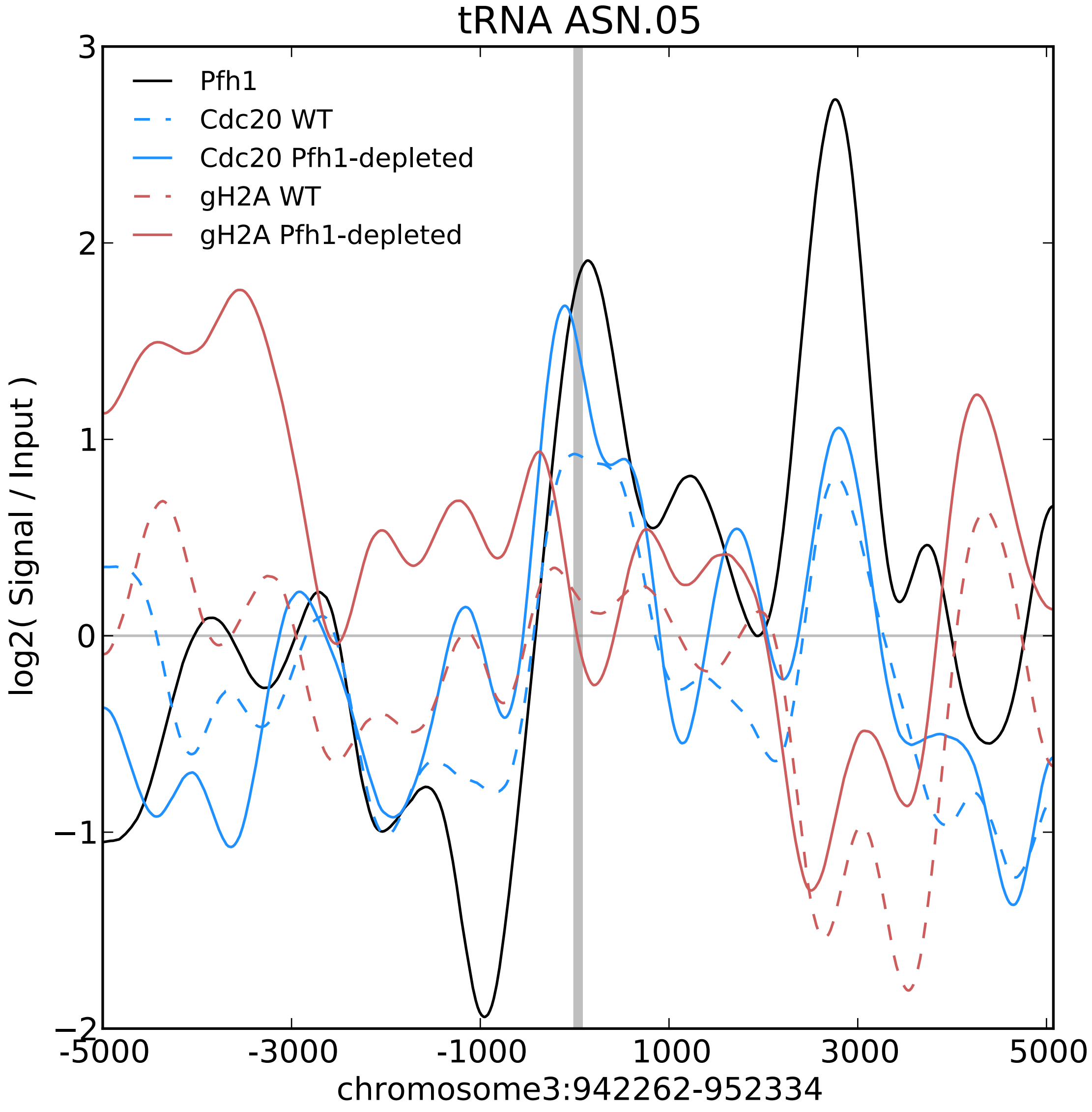

tRNA ASN.05
Pfh1
Cdc20 WT
Cdc20 Pfh1-depleted
gH2A WT
gH2A Pfh1-depleted
log2(Signal / Input)
3
2
1
0
−1
−2
-5000
-3000
-1000
1000
3000
5000
chromosome3:942262-952334

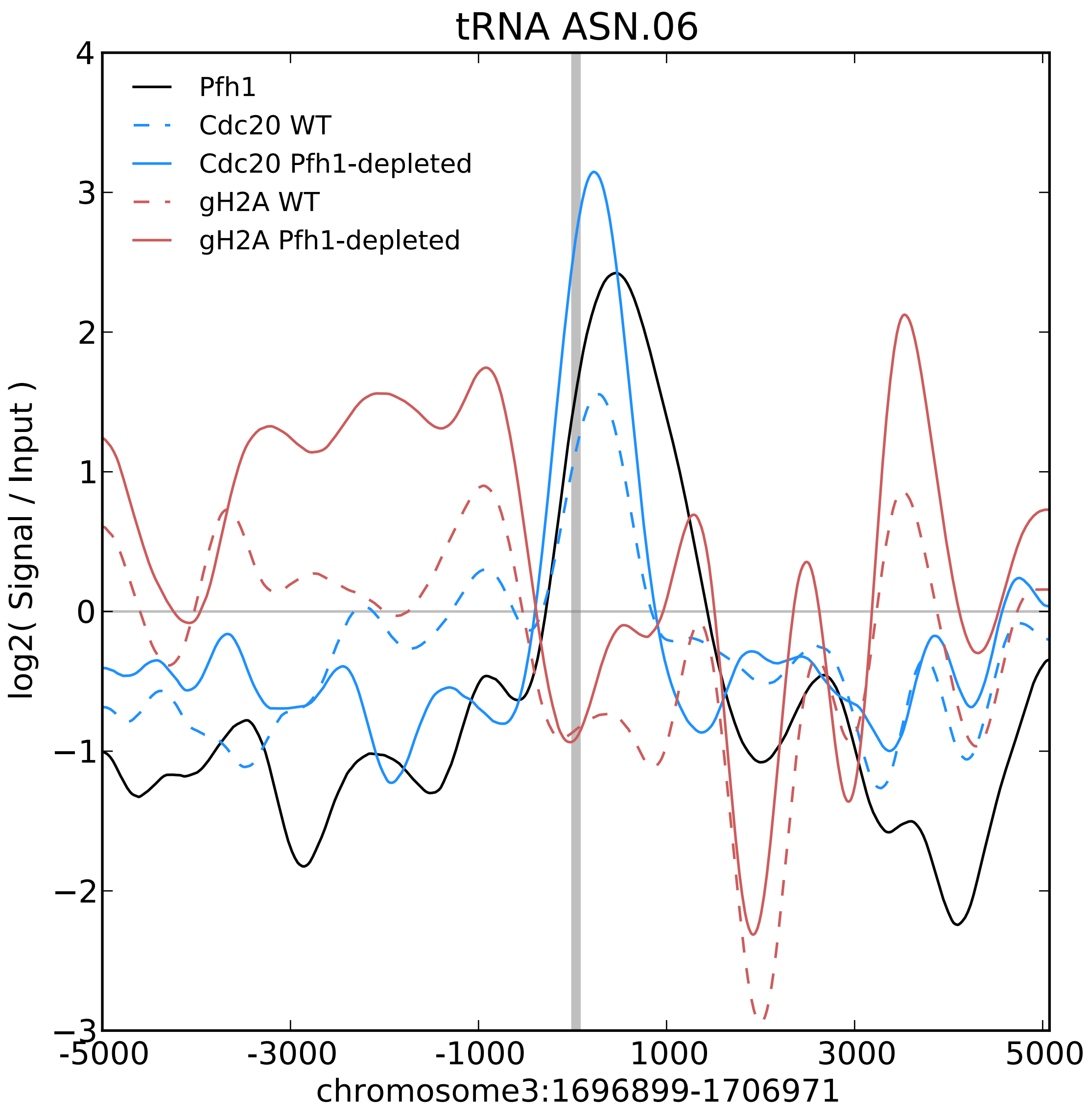

tRNA ASN.06
Pfh1
Cdc20 WT
Cdc20 Pfh1-depleted
gH2A WT
gH2A Pfh1-depleted
log2(Signal / Input)
4
3
2
1
0
−1
−2
−3
-5000
-3000
-1000
1000
3000
5000
chromosome3:1696899-1706971

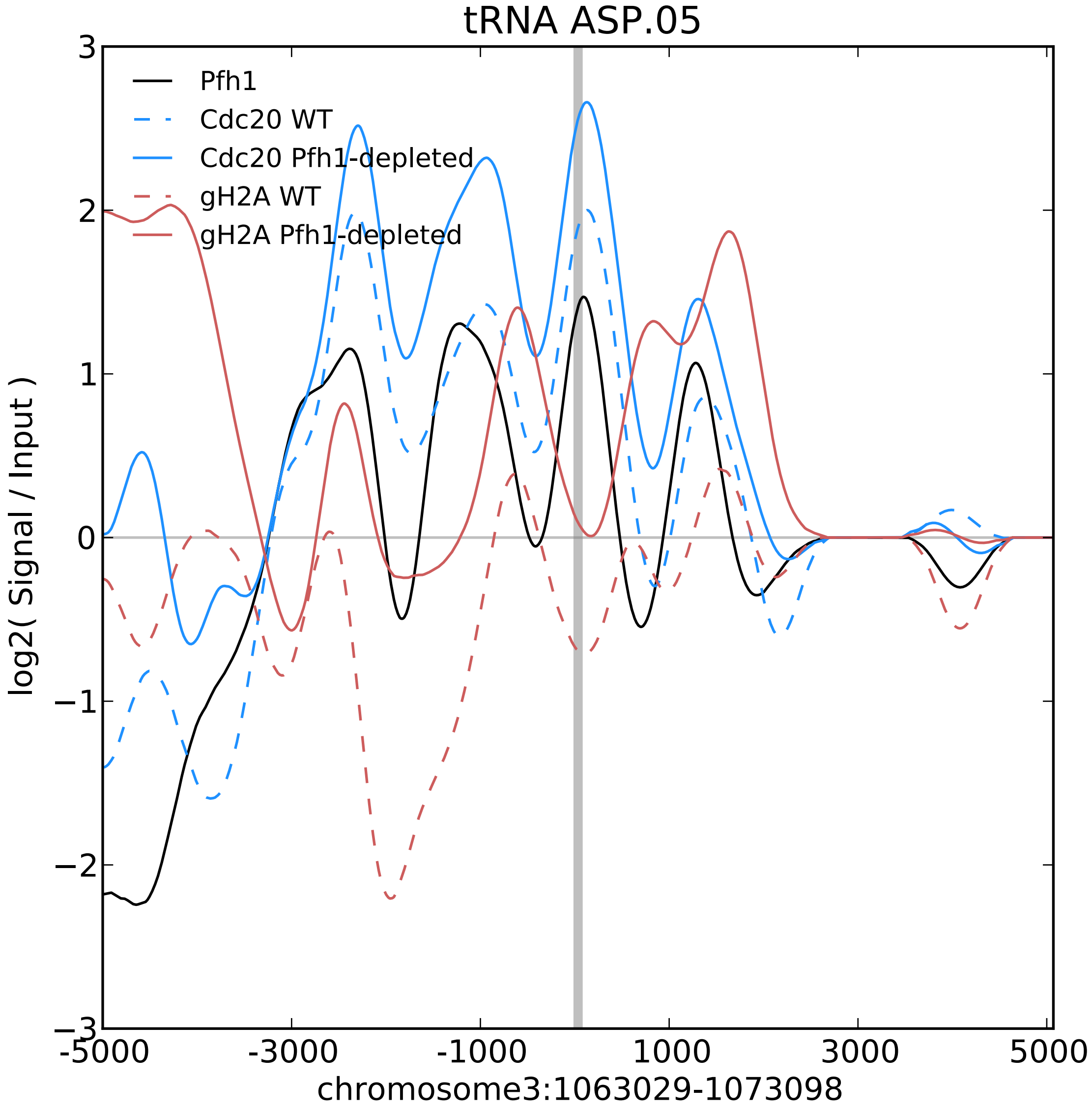

tRNA ASP.05
Pfh1
Cdc20 WT
Cdc20 Pfh1-depleted
gH2A WT
gH2A Pfh1-depleted
log2(Signal / Input)
3
2
1
0
−1
−2
−3
-5000
-3000
-1000
1000
3000
5000
chromosome3:1063029-1073098

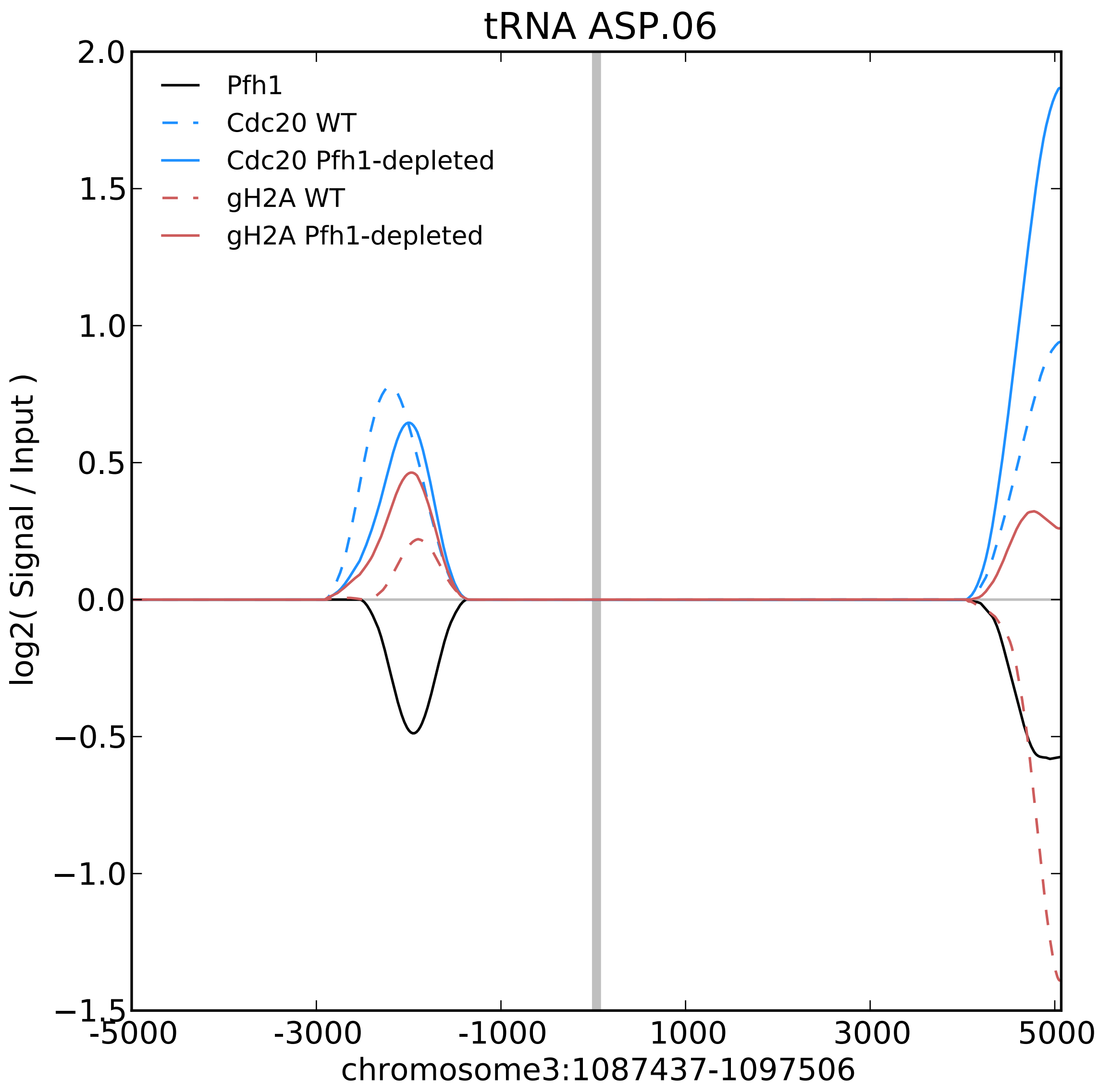

tRNA ASP.06
Pfh1
Cdc20 WT
Cdc20 Pfh1-depleted
gH2A WT
gH2A Pfh1-depleted
2.0
1.5
1.0
0.5
0.0
−0.5
−1.0
−1.5
log2(Signal / Input)
-5000
-3000
-1000
1000
3000
5000
chromosome3:1087437-1097506

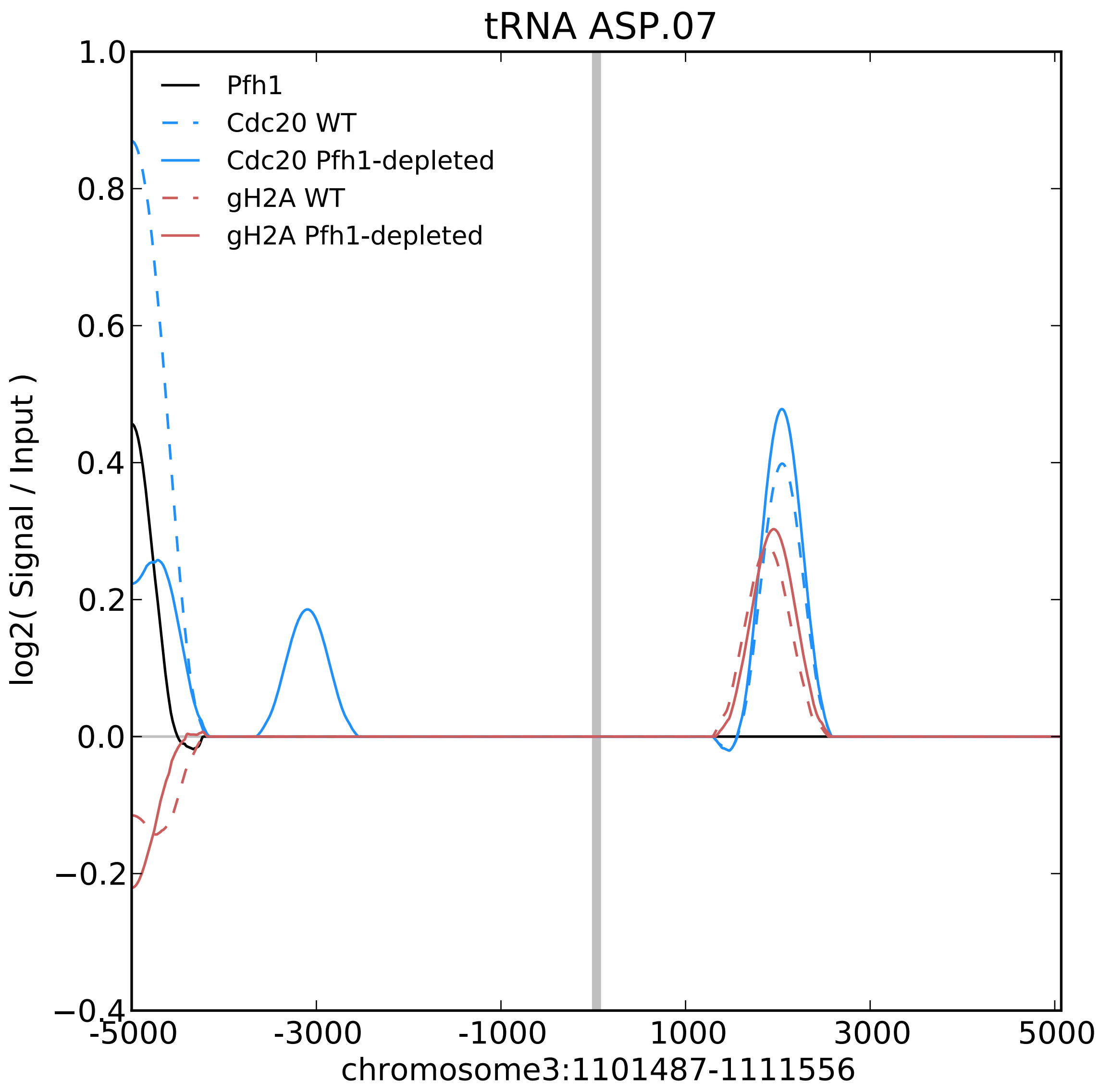
tRNA ASP.07
Pfh1
Cdc20 WT
Cdc20 Pfh1-depleted
gH2A WT
gH2A Pfh1-depleted
log2(Signal / Input)
1.0
0.8
0.6
0.4
0.2
0.0
−0.2
−0.4
-5000
-3000
-1000
1000
3000
5000
chromosome3:1101487-1111556

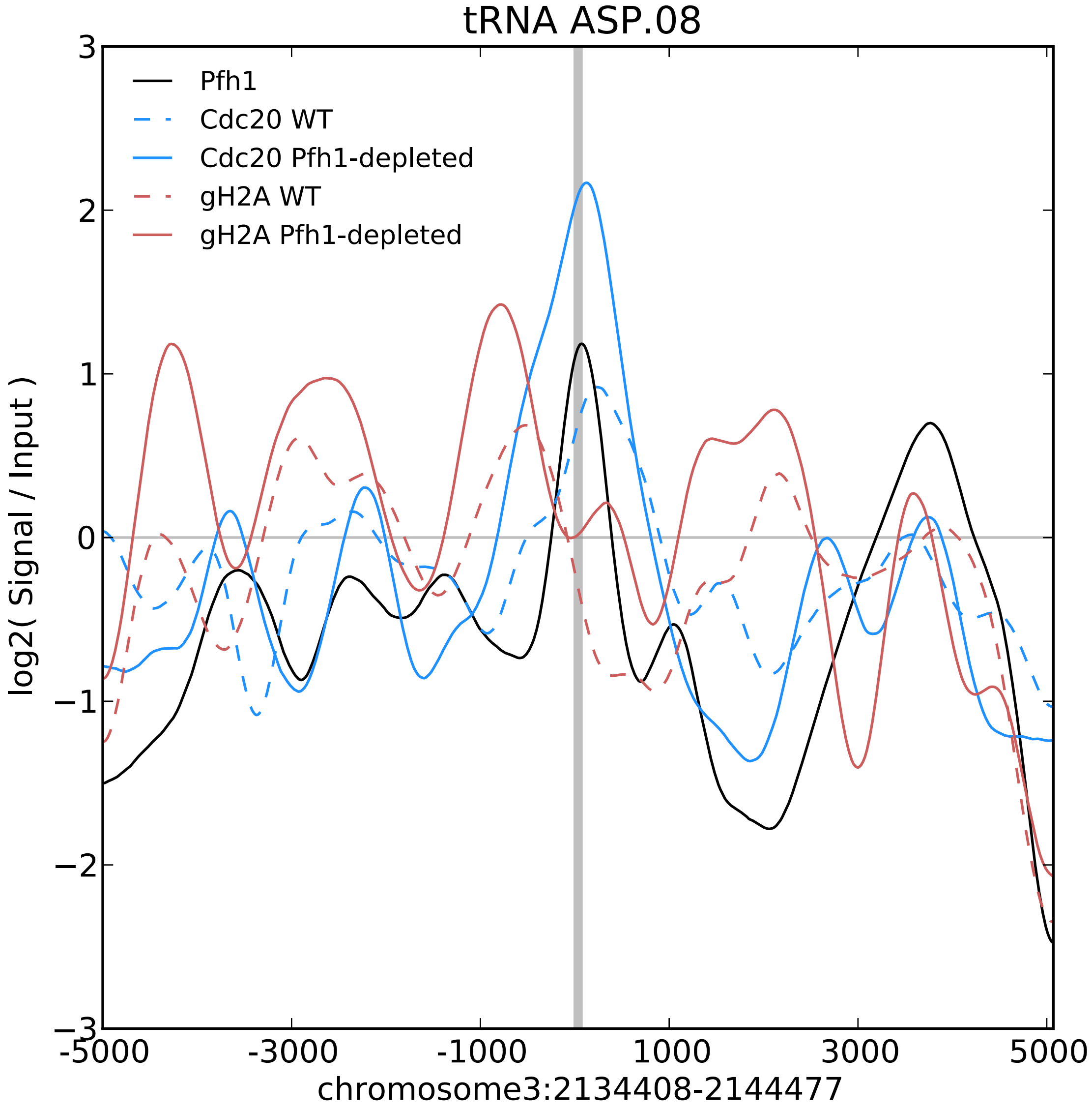

tRNA ASP.08
Pfh1
Cdc20 WT
Cdc20 Pfh1-depleted
gH2A WT
gH2A Pfh1-depleted
log2(Signal / Input)
3
2
1
0
−1
−2
−3
-5000
-3000
-1000
1000
3000
5000
chromosome3:2134408-2144477

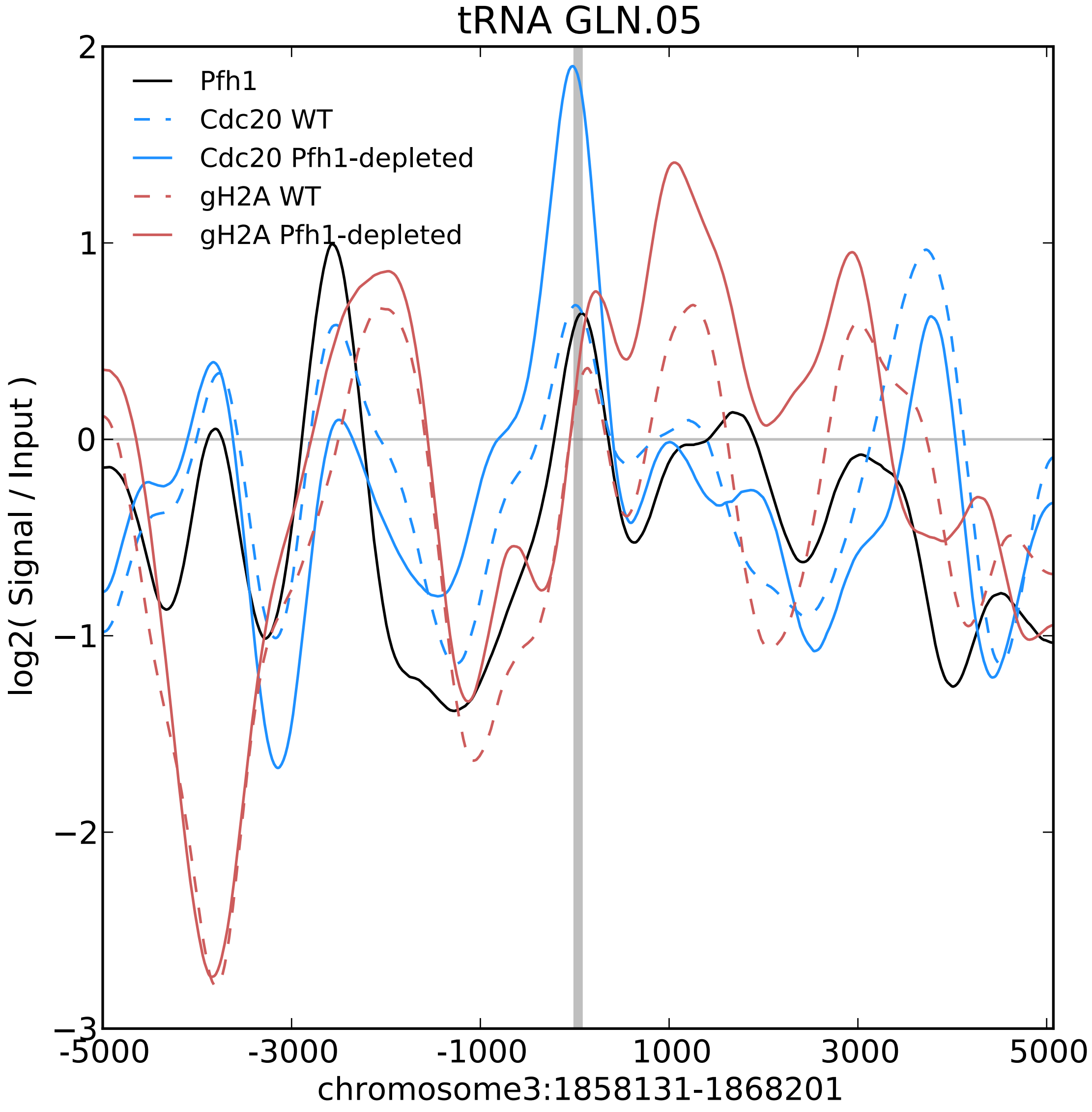

tRNA GLN.05
Pfh1
Cdc20 WT
Cdc20 Pfh1-depleted
gH2A WT
gH2A Pfh1-depleted
log2(Signal / Input)
2
1
0
−1
−2
−3
-5000
-3000
-1000
1000
3000
5000
chromosome3:1858131-1868201

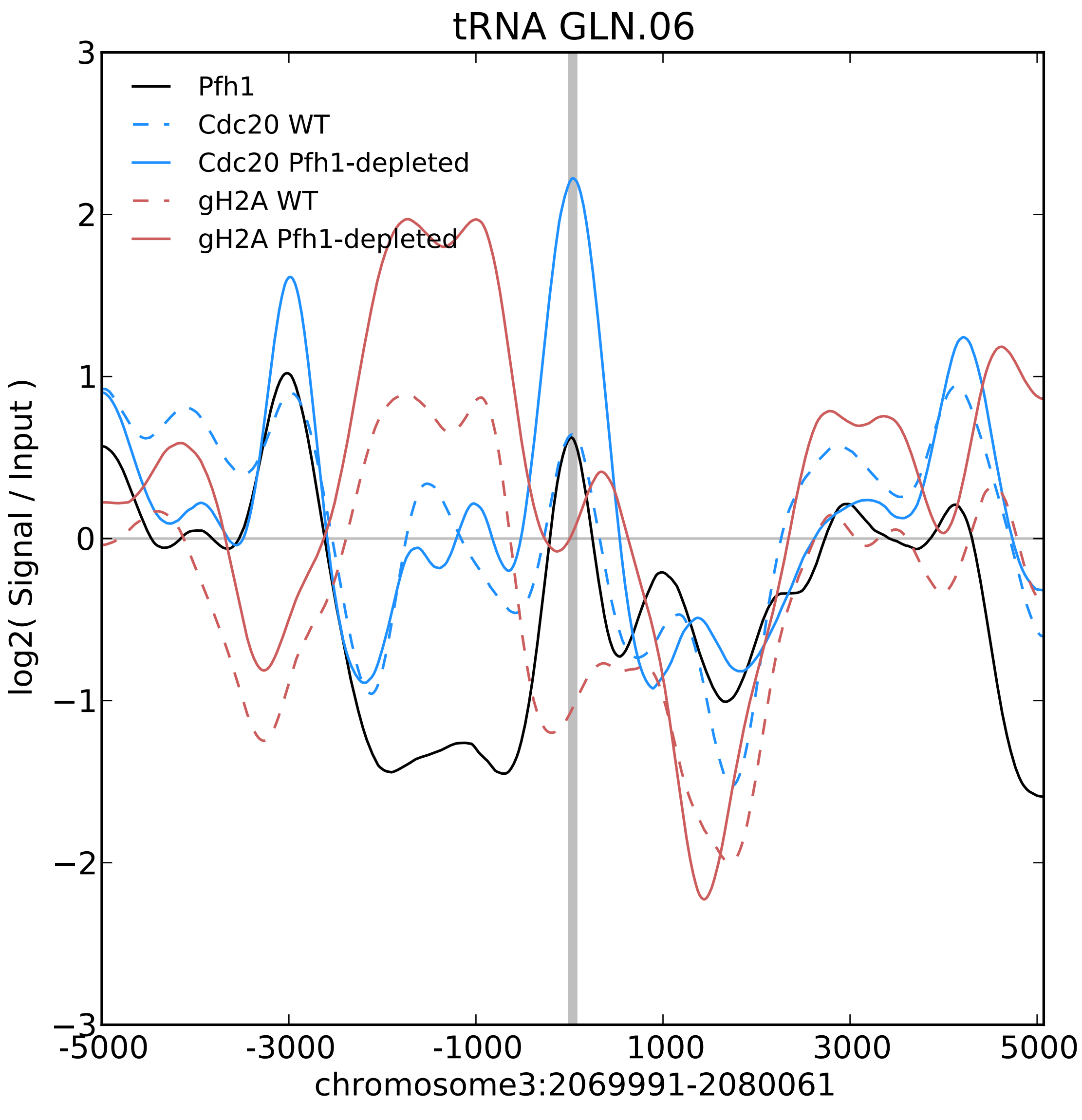
tRNA GLN.06
Pfh1
Cdc20 WT
Cdc20 Pfh1-depleted
gH2A WT
gH2A Pfh1-depleted
log2(Signal / Input)
chromosome3:2069991-2080061
3
2
1
0
−1
−2
−3
-5000
-3000
-1000
1000
3000
5000

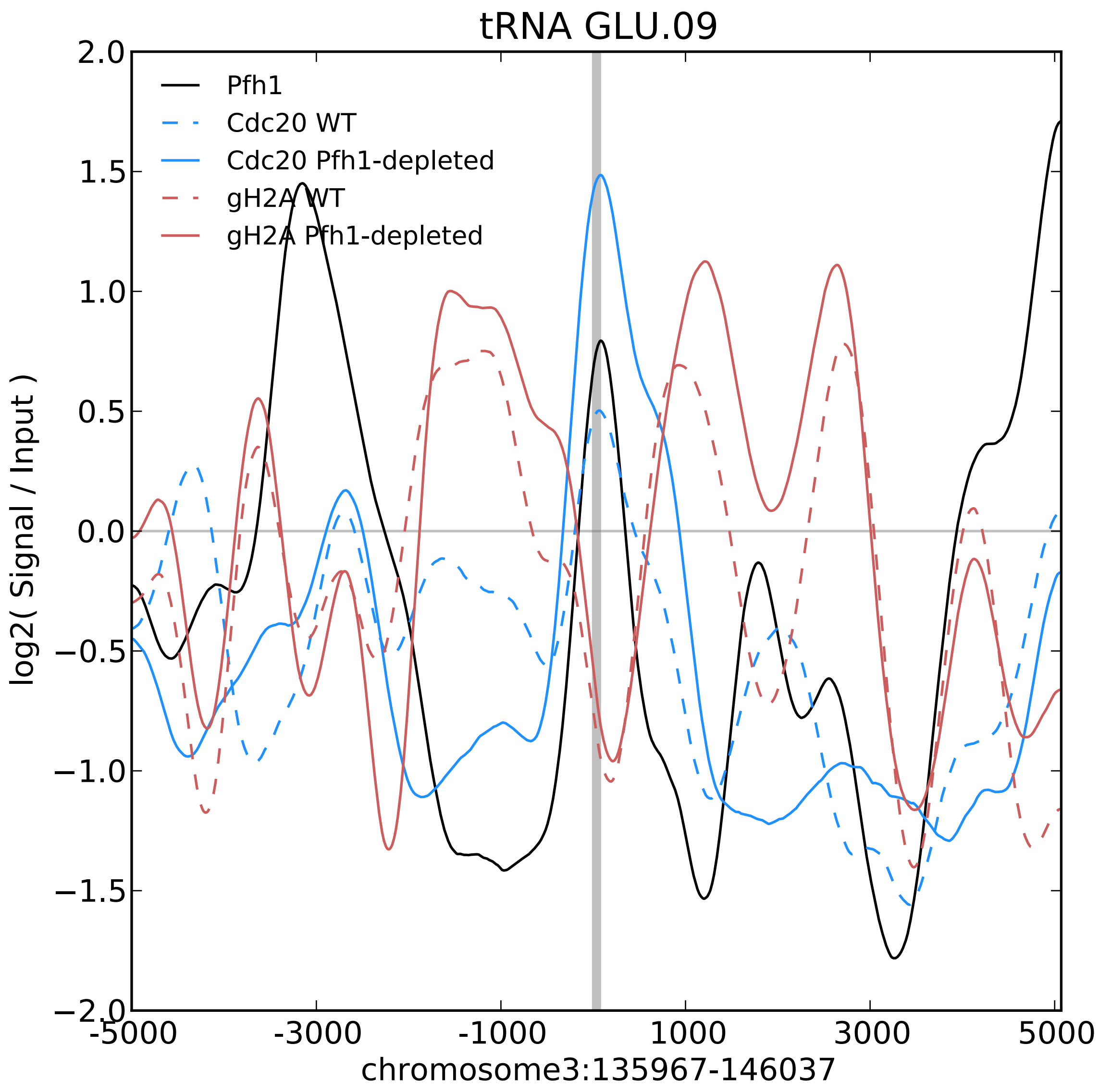
tRNA GLU.09
Pfh1
Cdc20 WT
Cdc20 Pfh1-depleted
gH2A WT
gH2A Pfh1-depleted
log2(Signal / Input)
2.0
1.5
1.0
0.5
0.0
−0.5
−1.0
−1.5
−2.0
-5000
-3000
-1000
1000
3000
5000
chromosome3:135967-146037

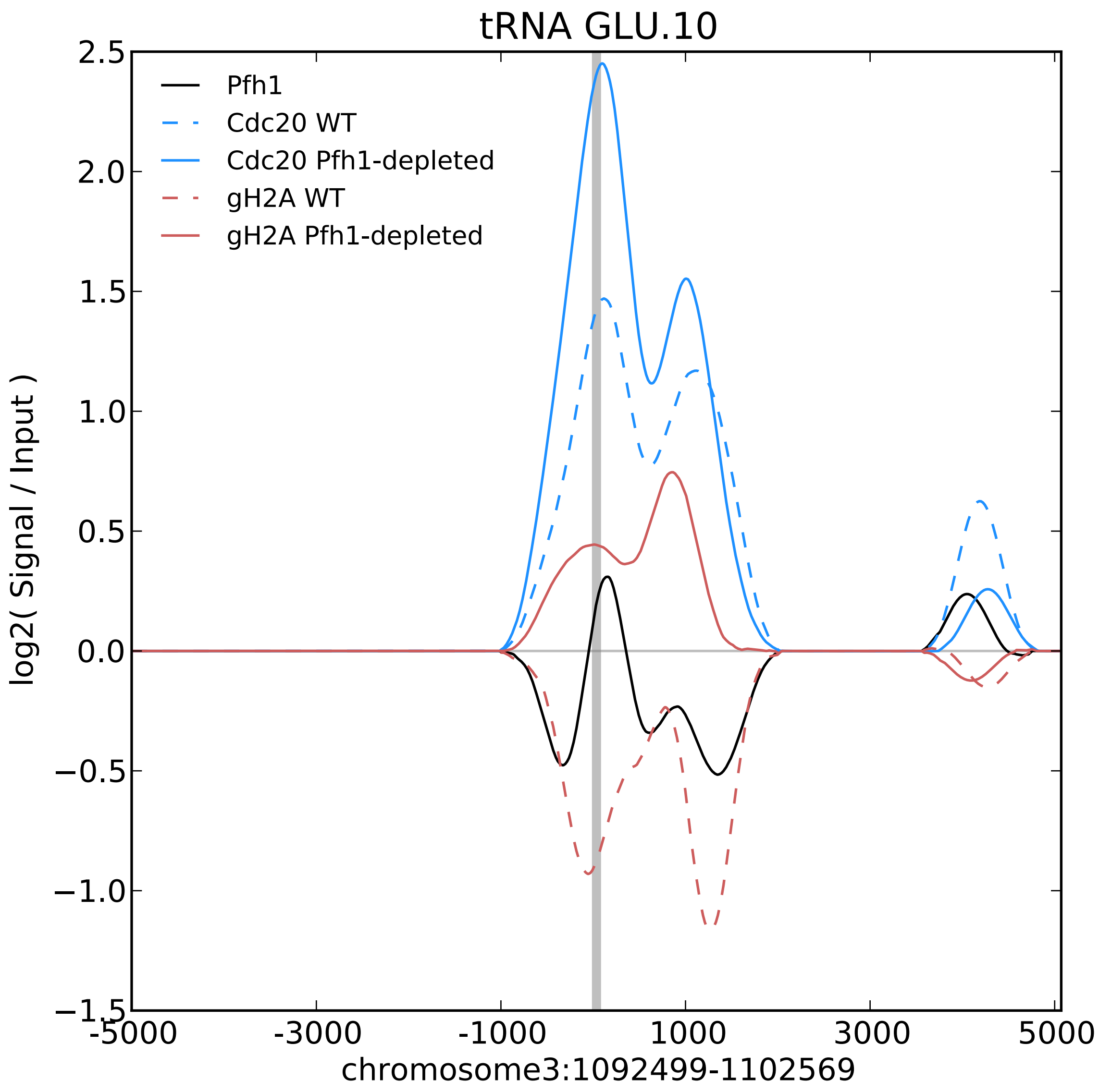

tRNA GLU.10
Pfh1
Cdc20 WT
Cdc20 Pfh1-depleted
gH2A WT
gH2A Pfh1-depleted
log2(Signal / Input)
2.5
2.0
1.5
1.0
0.5
0.0
−0.5
−1.0
−1.5
-5000
-3000
-1000
1000
3000
5000
chromosome3:1092499-1102569

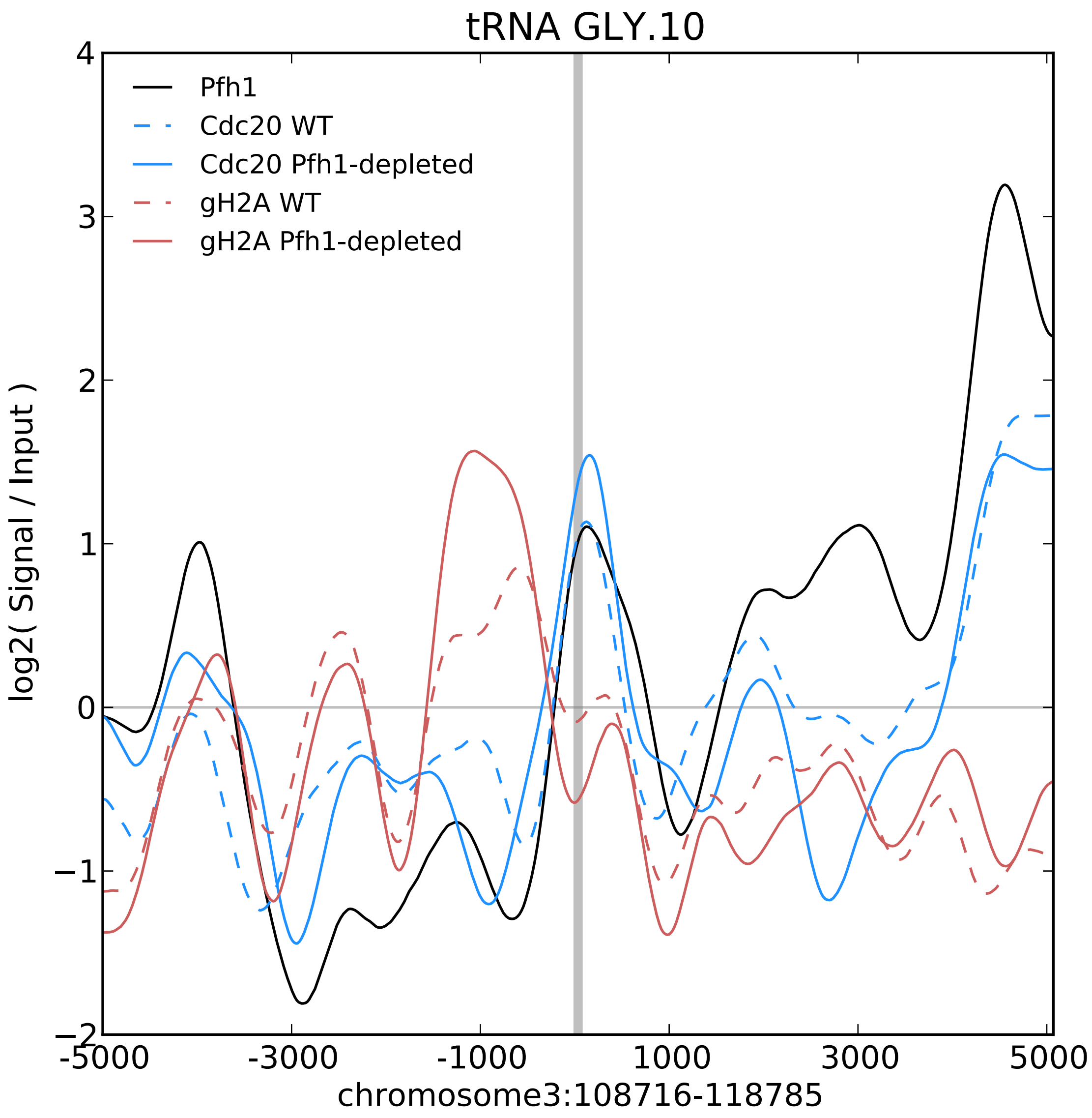
tRNA GLY.10
Pfh1
Cdc20 WT
Cdc20 Pfh1-depleted
gH2A WT
gH2A Pfh1-depleted
log2(Signal / Input)
4
3
2
1
0
−1
−2
-5000
-3000
-1000
1000
3000
5000
chromosome3:108716-118785

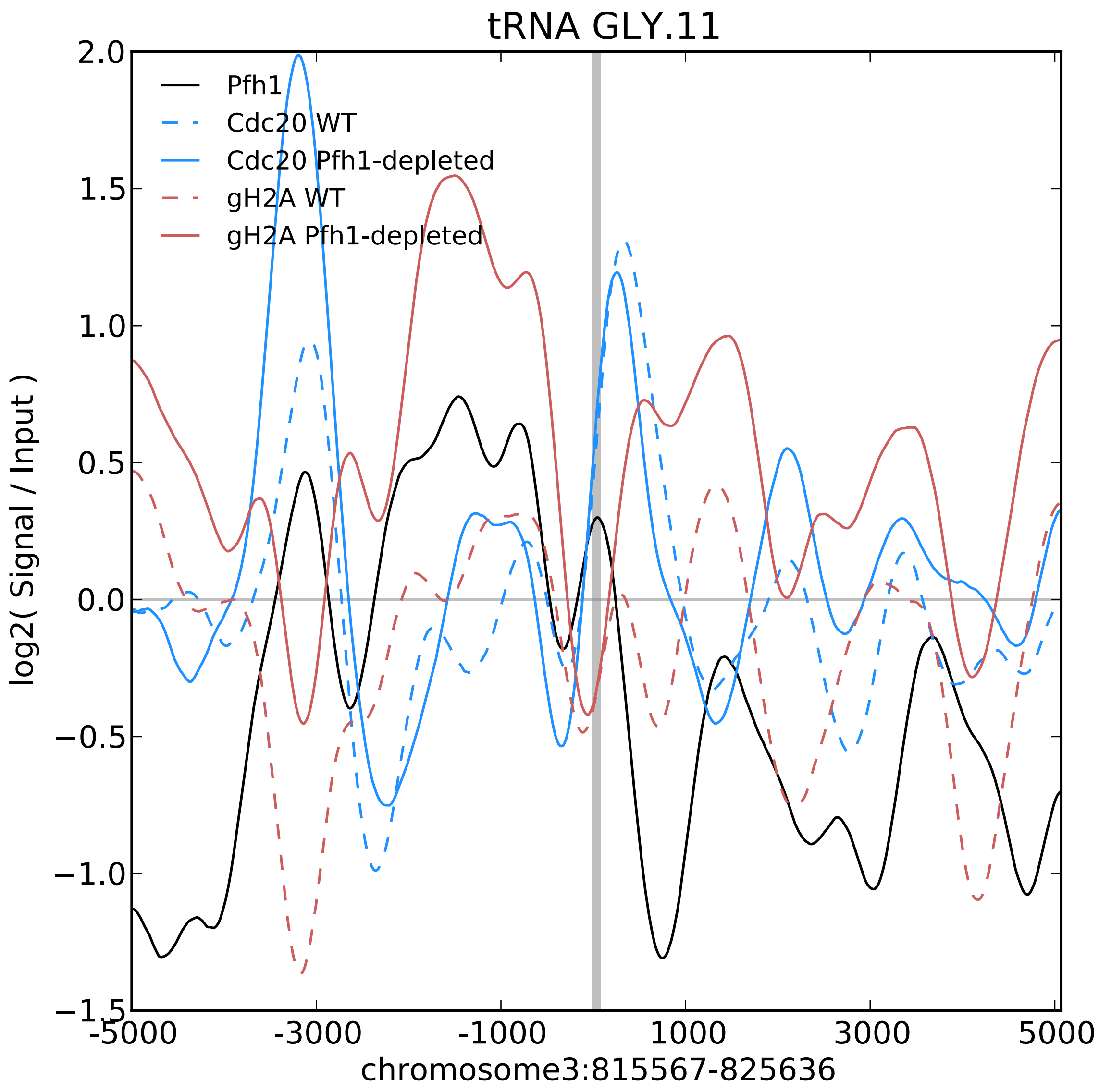

tRNA GLY.11
Pfh1
Cdc20 WT
Cdc20 Pfh1-depleted
gH2A WT
gH2A Pfh1-depleted
2.0
1.5
1.0
0.5
0.0
−0.5
−1.0
−1.5
log2(Signal / Input)
-5000
-3000
-1000
1000
3000
5000
chromosome3:815567-825636

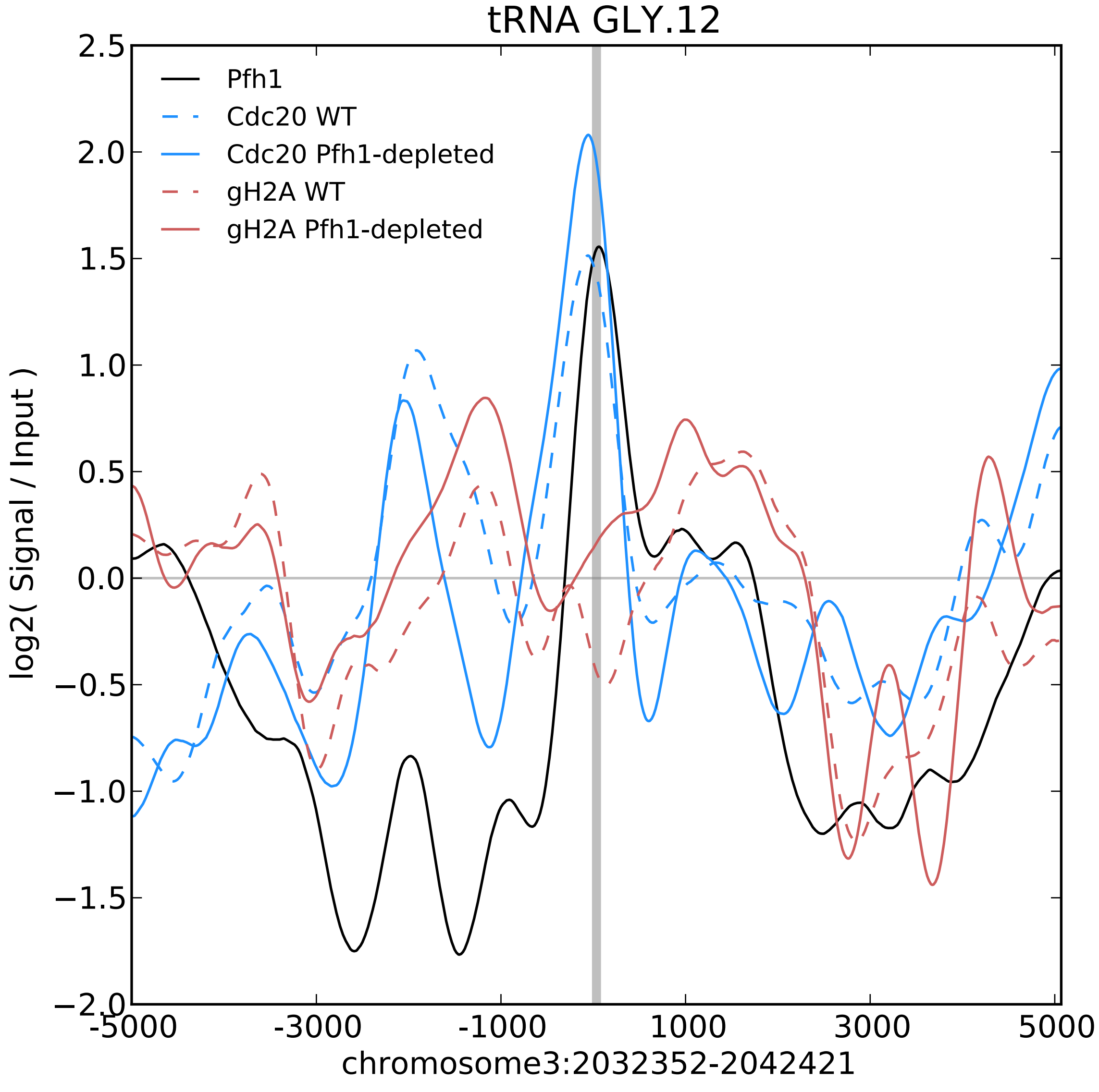

tRNA GLY.12
Pfh1
Cdc20 WT
Cdc20 Pfh1-depleted
gH2A WT
gH2A Pfh1-depleted
2.5
2.0
1.5
1.0
0.5
0.0
−0.5
−1.0
−1.5
−2.0
log2(Signal / Input)
-5000
-3000
-1000
1000
3000
5000
chromosome3:2032352-2042421

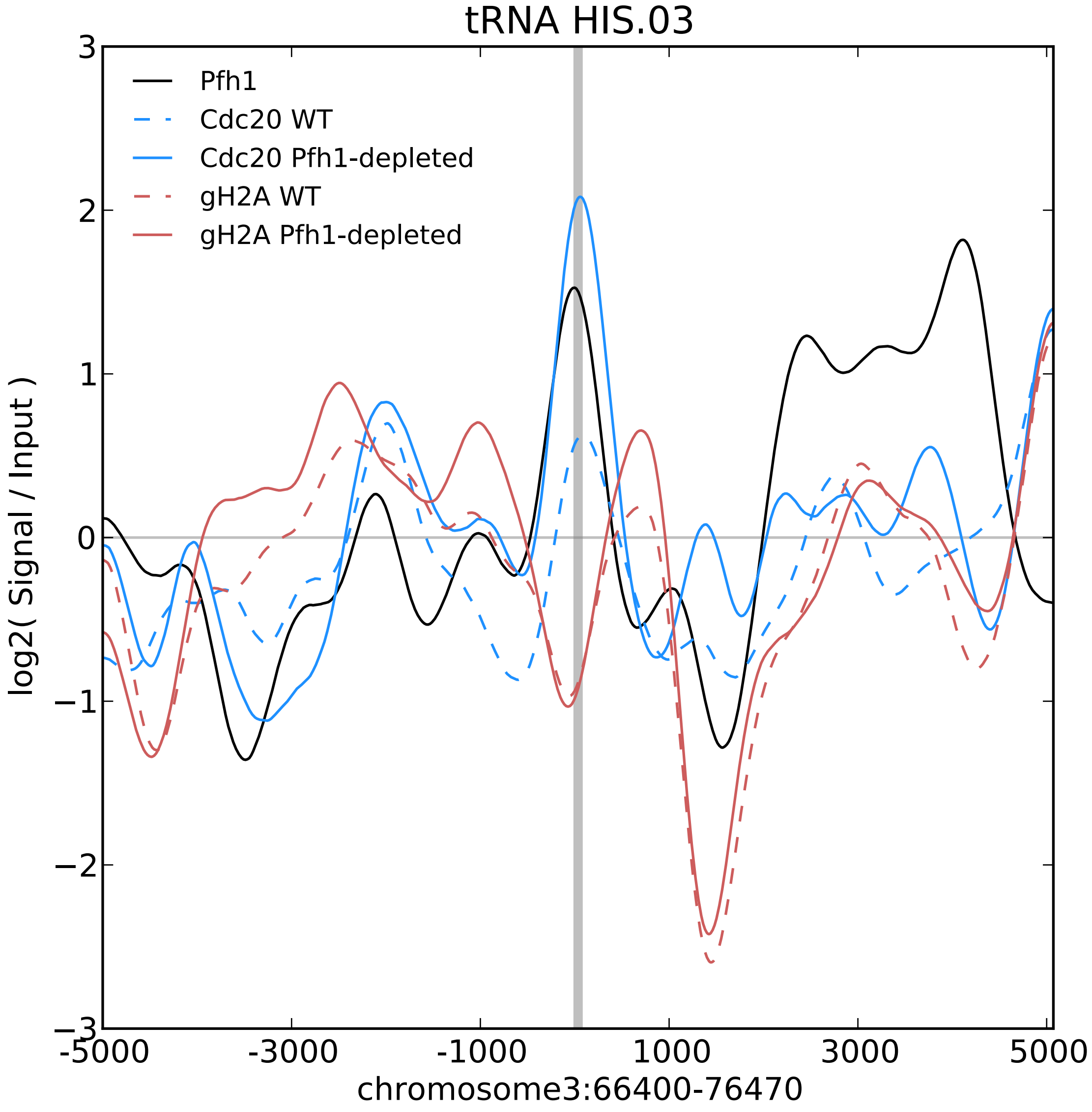

tRNA HIS.03
Pfh1
Cdc20 WT
Cdc20 Pfh1-depleted
gH2A WT
gH2A Pfh1-depleted
3
2
1
0
−1
−2
−3
log2(Signal / Input)
-5000
-3000
-1000
1000
3000
5000
chromosome3:66400-76470

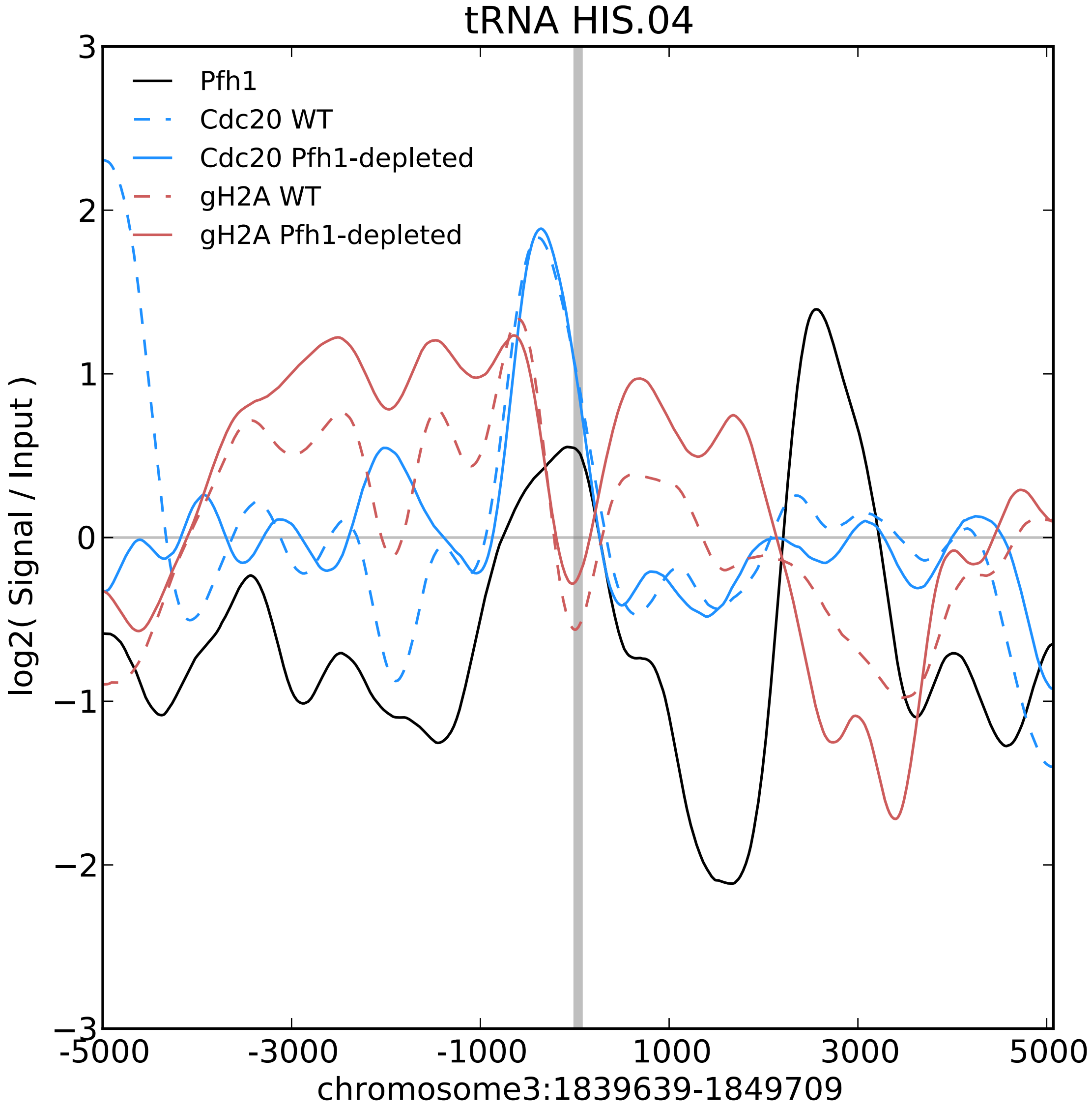

tRNA HIS.04
Pfh1
Cdc20 WT
Cdc20 Pfh1-depleted
gH2A WT
gH2A Pfh1-depleted
log2(Signal / Input)
3
2
1
0
−1
−2
−3
-5000
-3000
-1000
1000
3000
5000
chromosome3:1839639-1849709

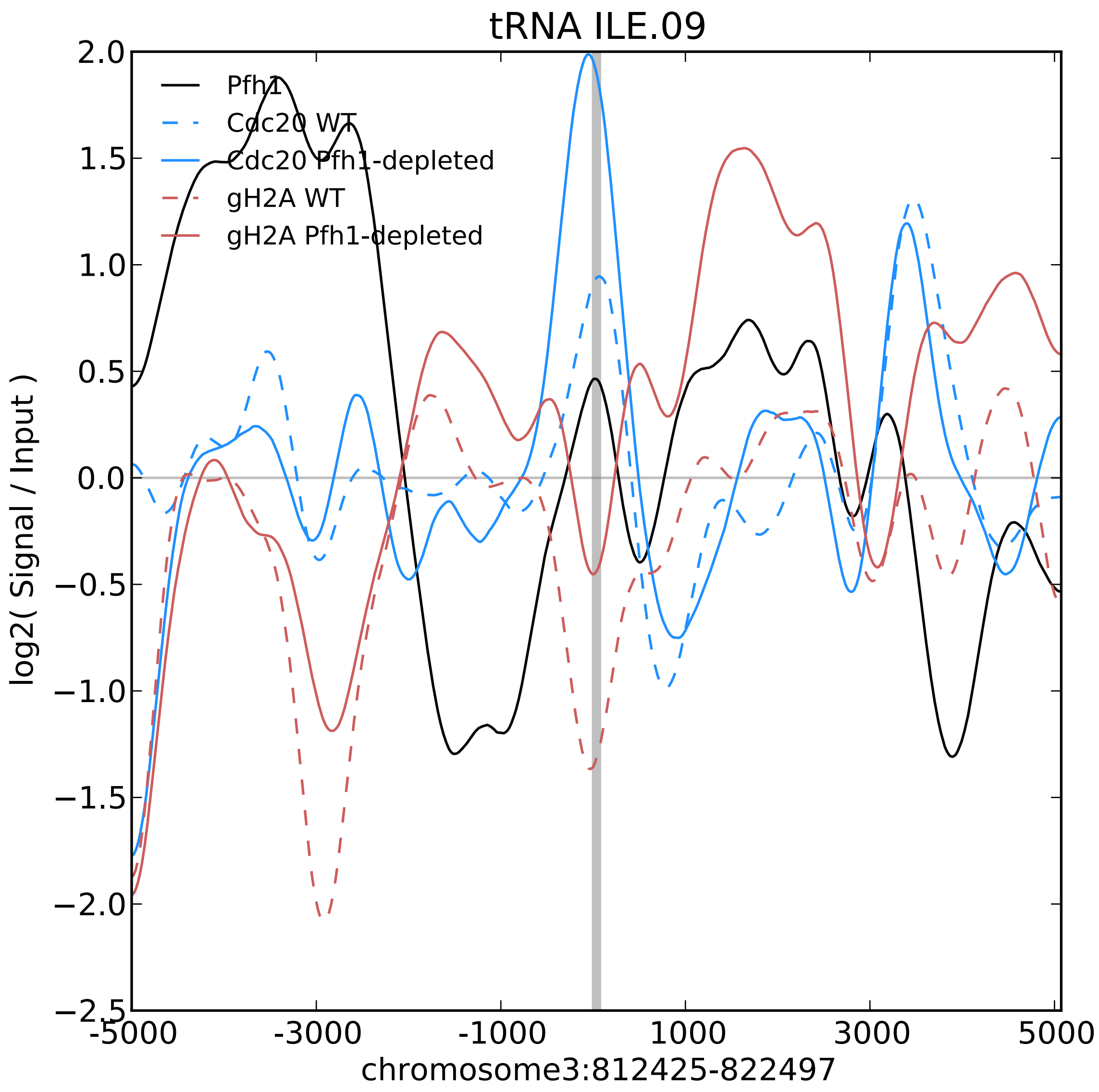

tRNA ILE.09
Pfh1
Cdc20 WT
Cdc20 Pfh1-depleted
gH2A WT
gH2A Pfh1-depleted
2.0
1.5
1.0
0.5
0.0
−0.5
−1.0
−1.5
−2.0
−2.5
log2(Signal / Input)
-5000
-3000
-1000
1000
3000
5000
chromosome3:812425-822497

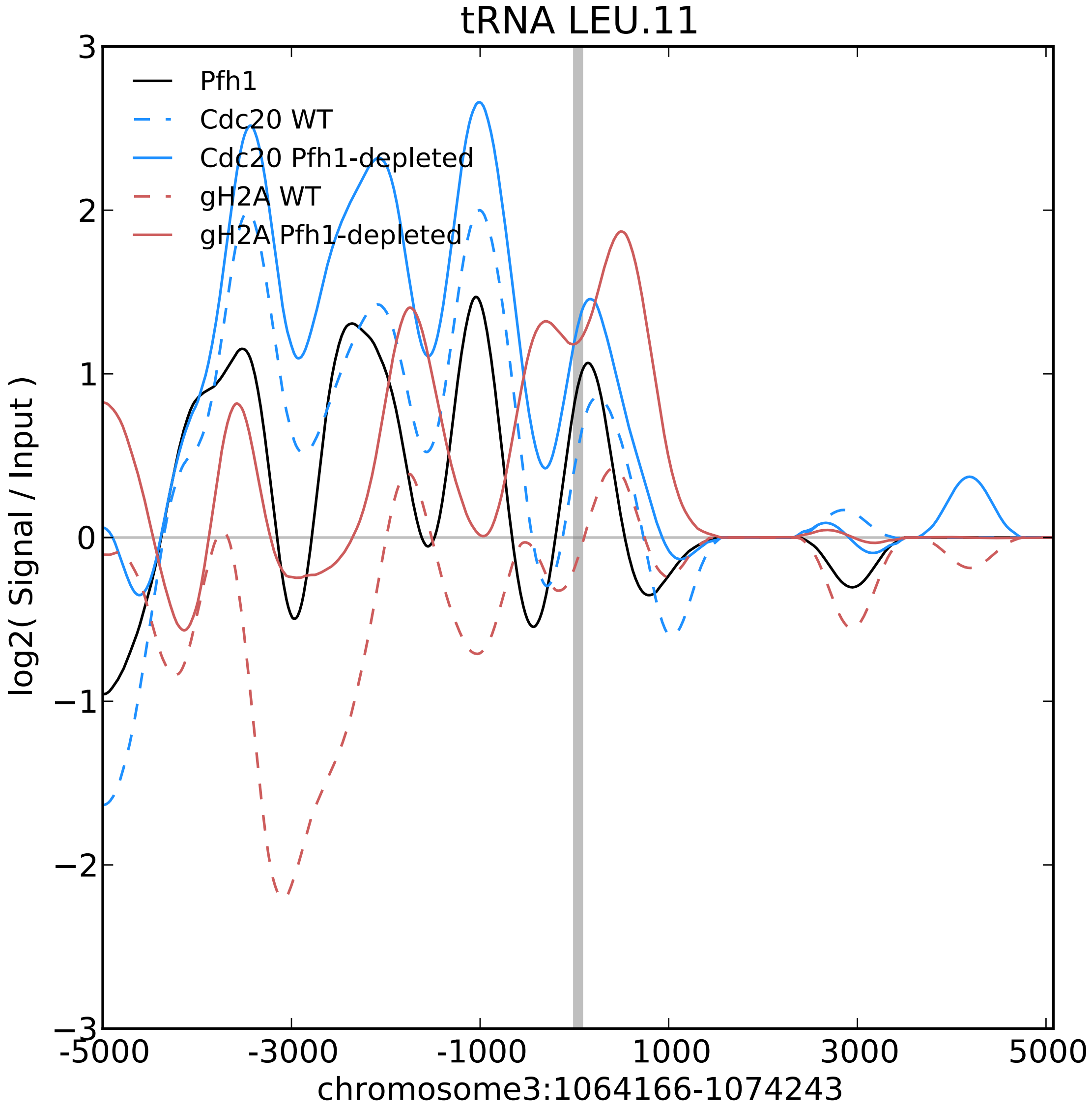

tRNA LEU.11
Pfh1
Cdc20 WT
Cdc20 Pfh1-depleted
gH2A WT
gH2A Pfh1-depleted
log2(Signal / Input)
3
2
1
0
−1
−2
−3
-5000
-3000
-1000
1000
3000
5000
chromosome3:1064166-1074243

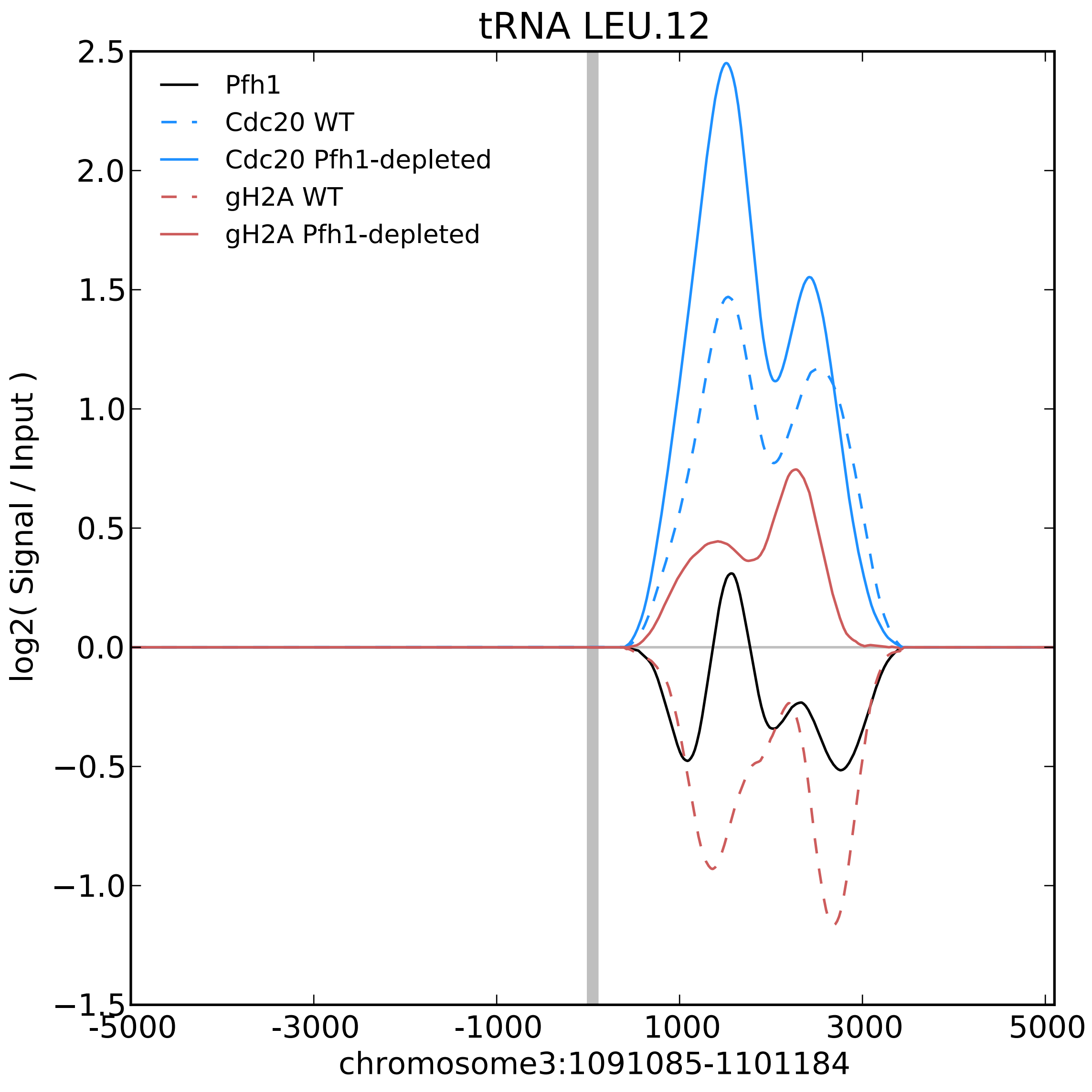
tRNA LEU.12
Pfh1
Cdc20 WT
Cdc20 Pfh1-depleted
gH2A WT
gH2A Pfh1-depleted
log2(Signal / Input)
2.5
2.0
1.5
1.0
0.5
0.0
−0.5
−1.0
−1.5
-5000
-3000
-1000
1000
3000
5000
chromosome3:1091085-1101184

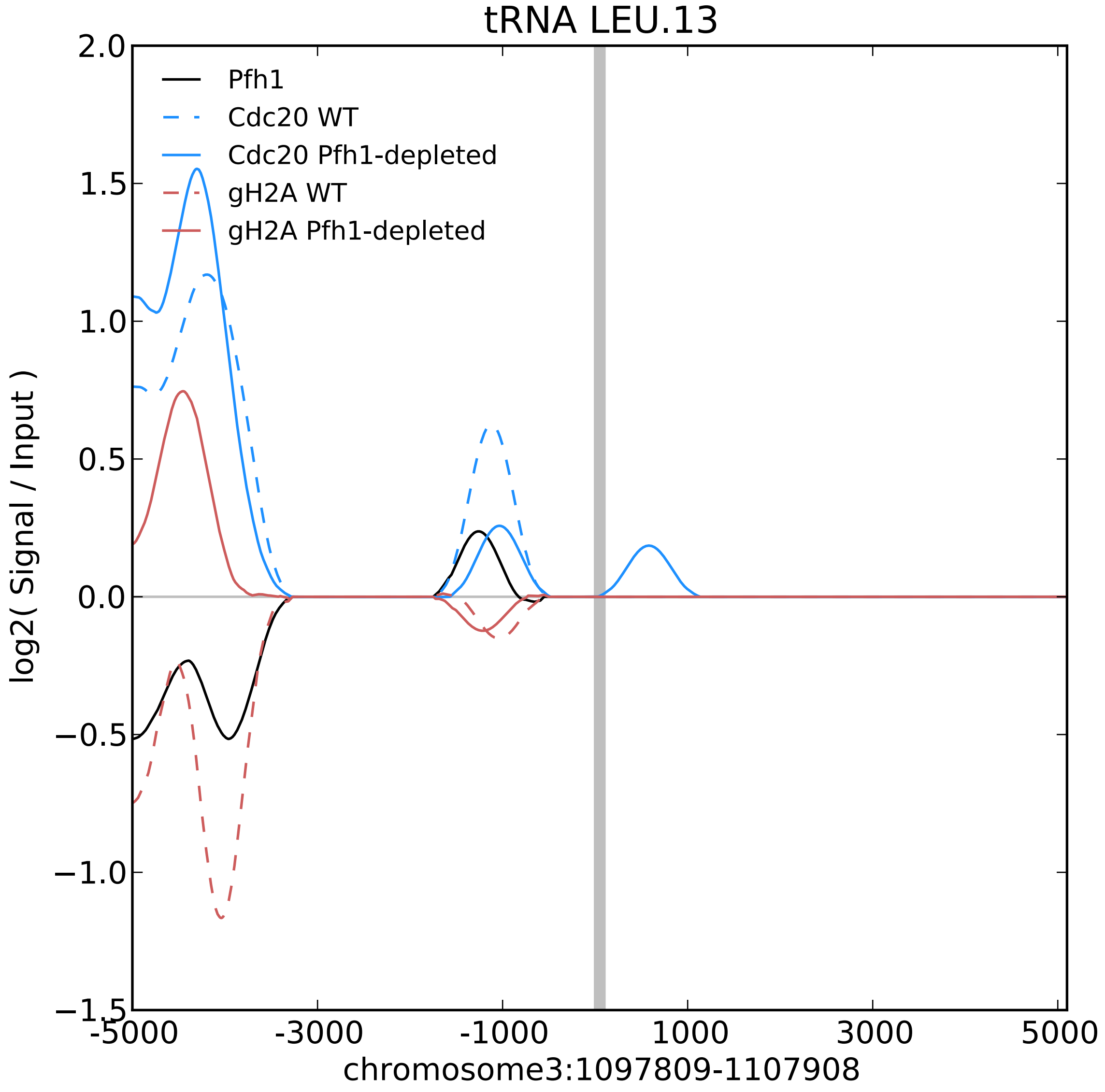
tRNA LEU.13
Pfh1
Cdc20 WT
Cdc20 Pfh1-depleted
gH2A WT
gH2A Pfh1-depleted
log2(Signal / Input)
2.0
1.5
1.0
0.5
0.0
−0.5
−1.0
−1.5
-5000
-3000
-1000
1000
3000
5000
chromosome3:1097809-1107908

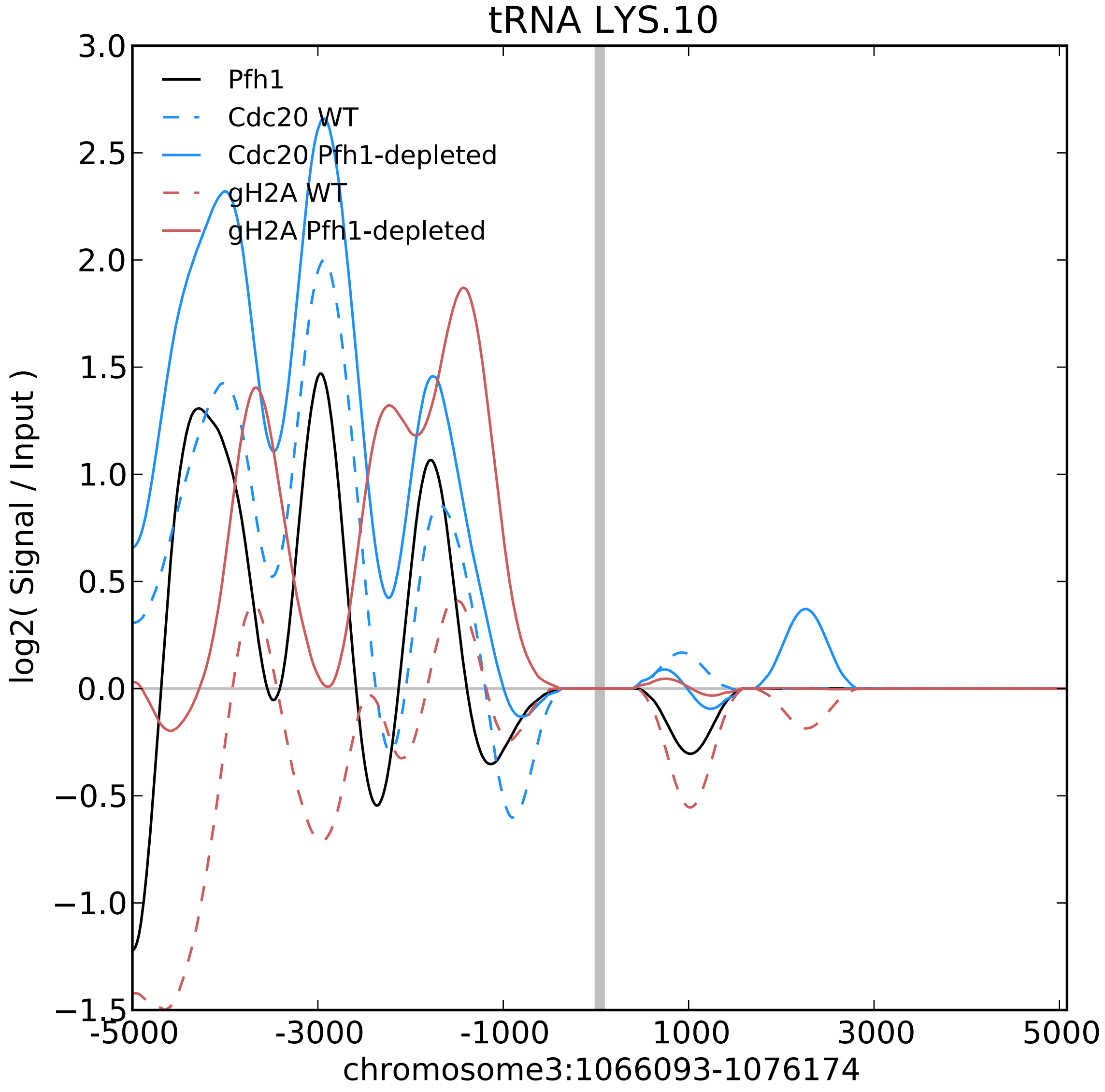

tRNA LYS.10
Pfh1
Cdc20 WT
Cdc20 Pfh1-depleted
gH2A WT
gH2A Pfh1-depleted
3.0
2.5
2.0
1.5
1.0
0.5
0.0
−0.5
−1.0
−1.5
log2(Signal / Input)
-5000
-3000
-1000
1000
3000
5000
chromosome3:1066093-1076174

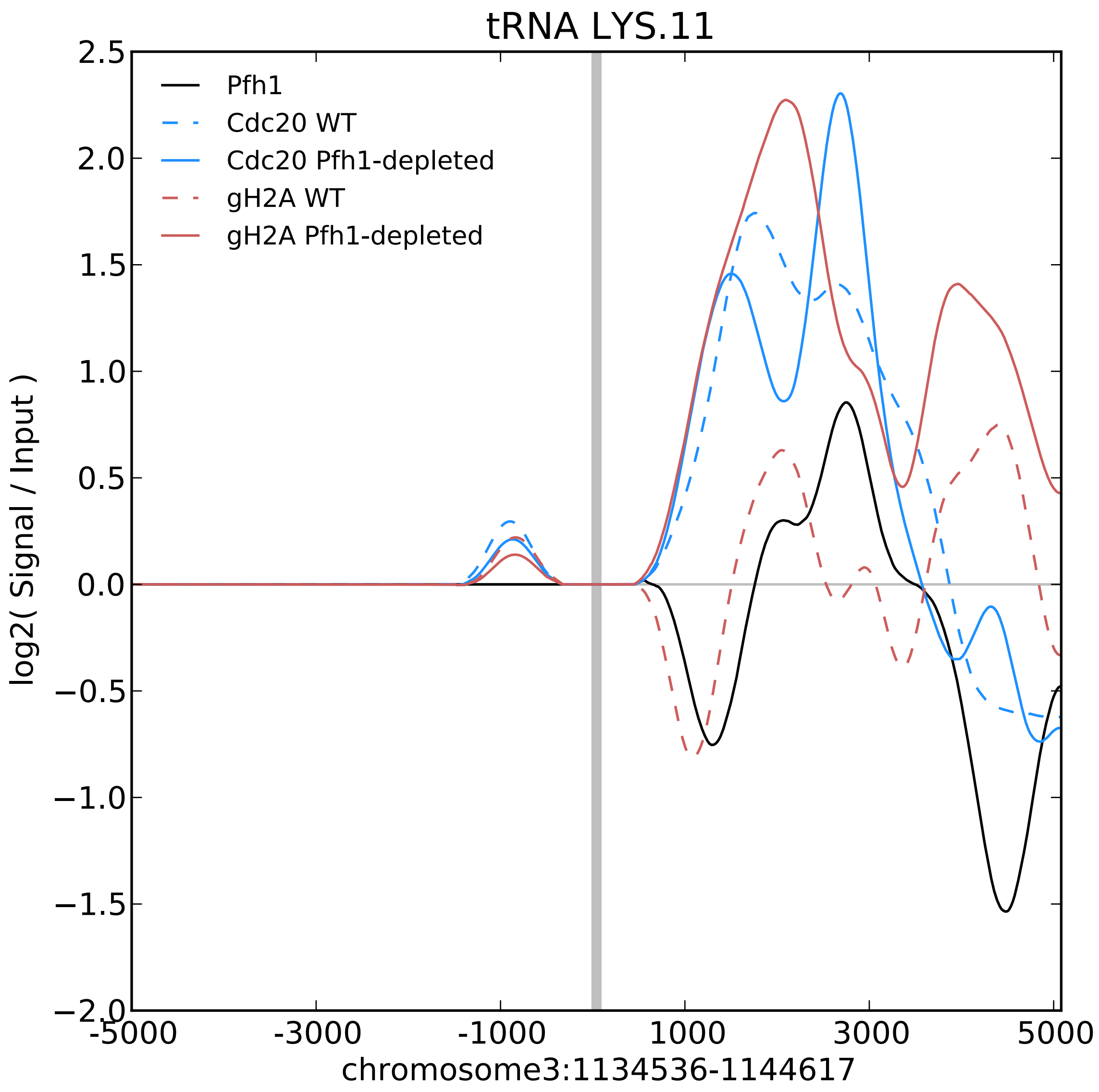

tRNA LYS.11
Pfh1
Cdc20 WT
Cdc20 Pfh1-depleted
gH2A WT
gH2A Pfh1-depleted
2.5
2.0
1.5
1.0
0.5
0.0
−0.5
−1.0
−1.5
−2.0
log2(Signal / Input)
-5000
-3000
-1000
1000
3000
5000
chromosome3:1134536-1144617

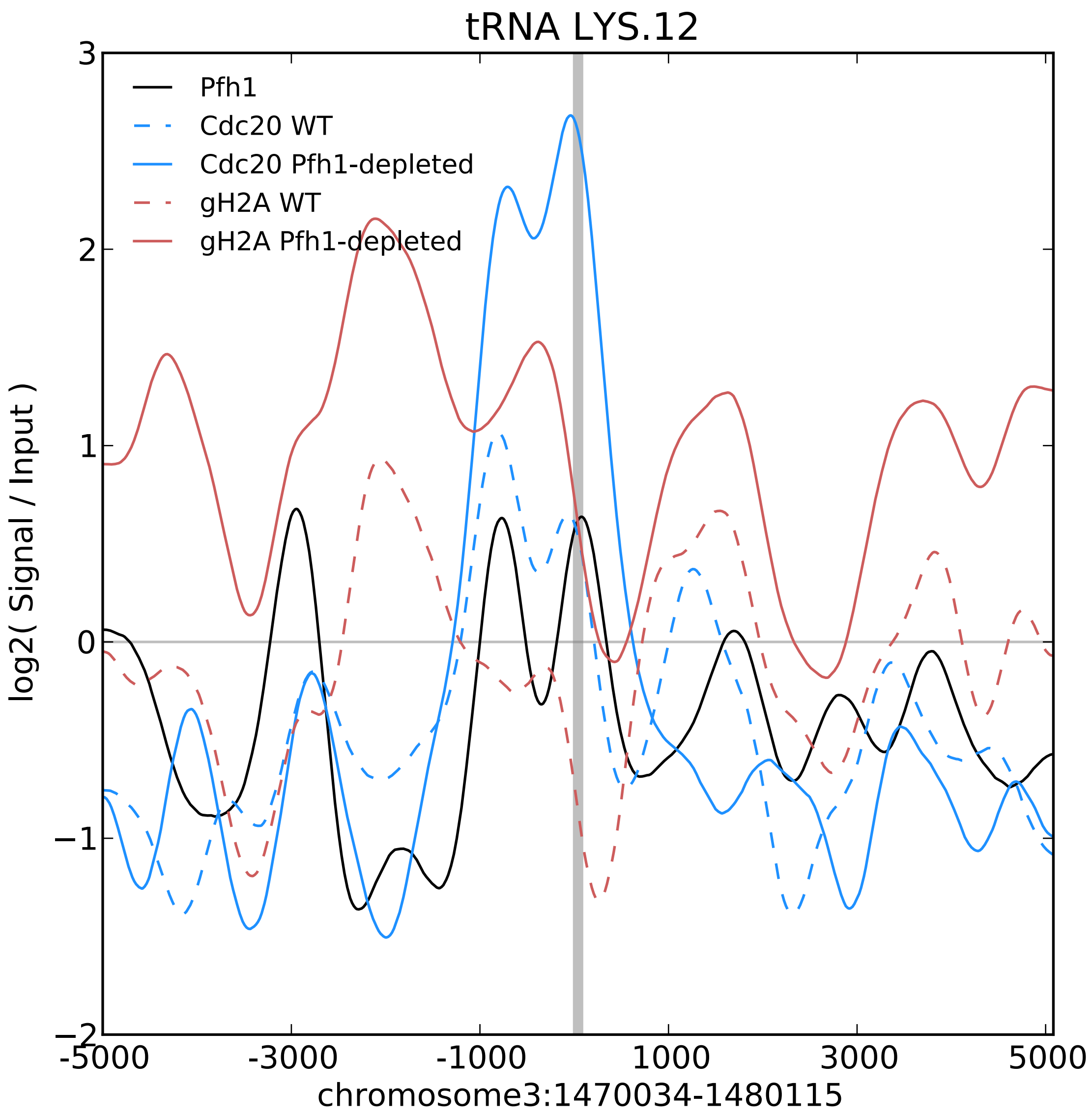

tRNA LYS.12
Pfh1
Cdc20 WT
Cdc20 Pfh1-depleted
gH2A WT
gH2A Pfh1-depleted
3
2
1
0
−1
−2
log2(Signal / Input)
-5000
-3000
-1000
1000
3000
5000
chromosome3:1470034-1480115

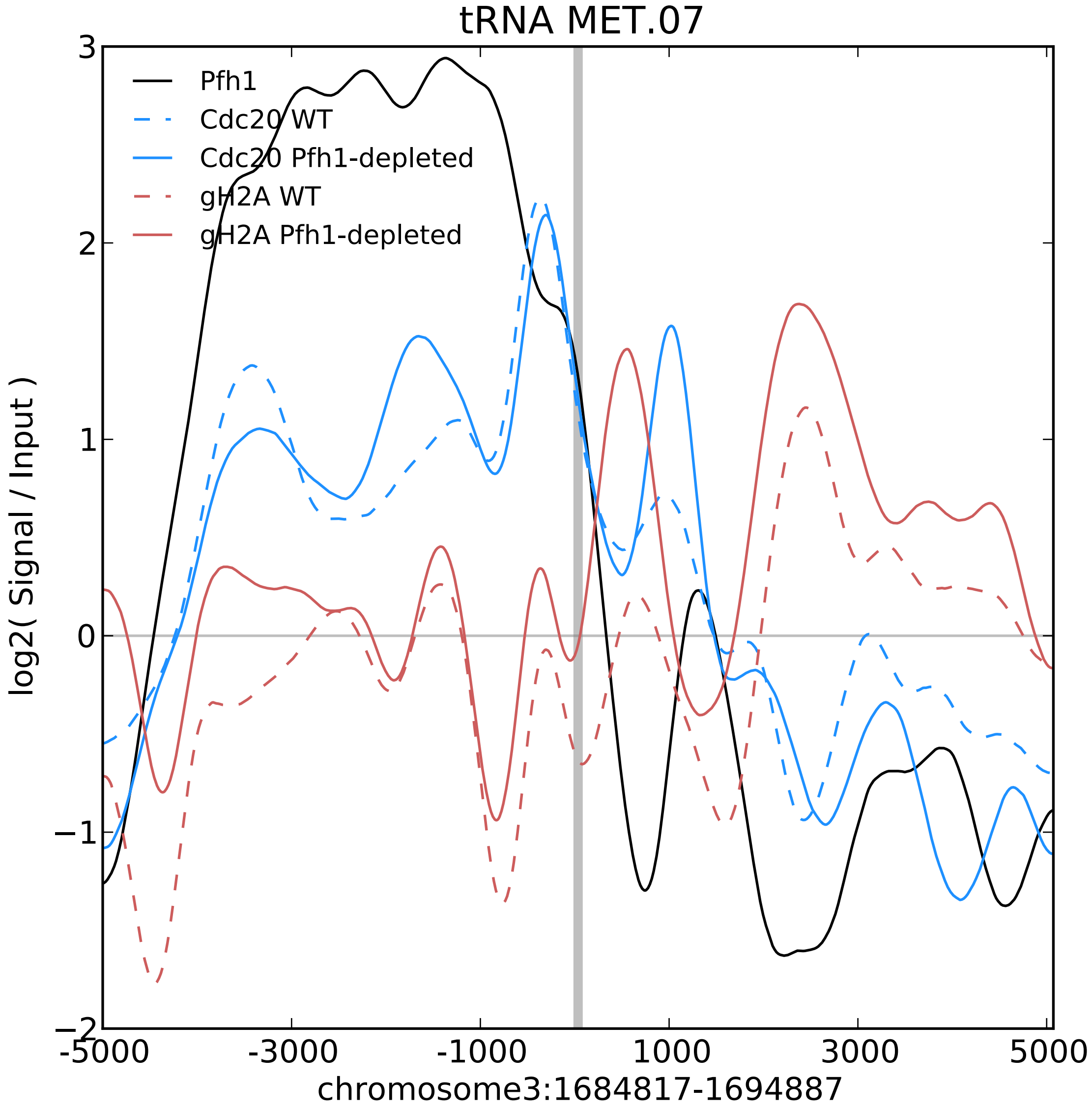

tRNA MET.07
Pfh1
Cdc20 WT
Cdc20 Pfh1-depleted
gH2A WT
gH2A Pfh1-depleted
log2(Signal / Input)
3
2
1
0
−1
−2
-5000
-3000
-1000
1000
3000
5000
chromosome3:1684817-1694887

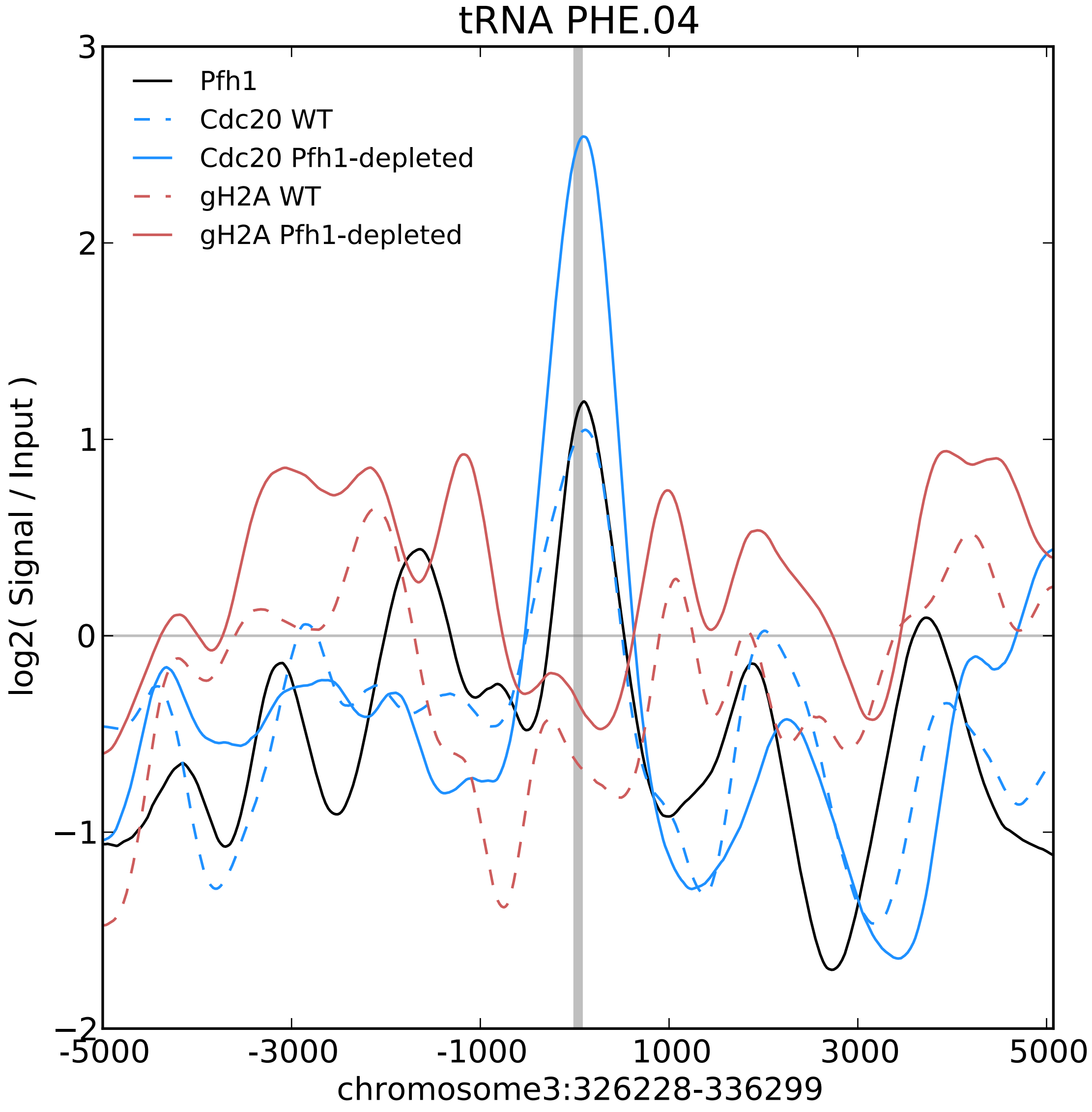

tRNA PHE.04
Pfh1
Cdc20 WT
Cdc20 Pfh1-depleted
gH2A WT
gH2A Pfh1-depleted
log2(Signal / Input)
3
2
1
0
−1
−2
-5000
-3000
-1000
1000
3000
5000
chromosome3:326228-336299

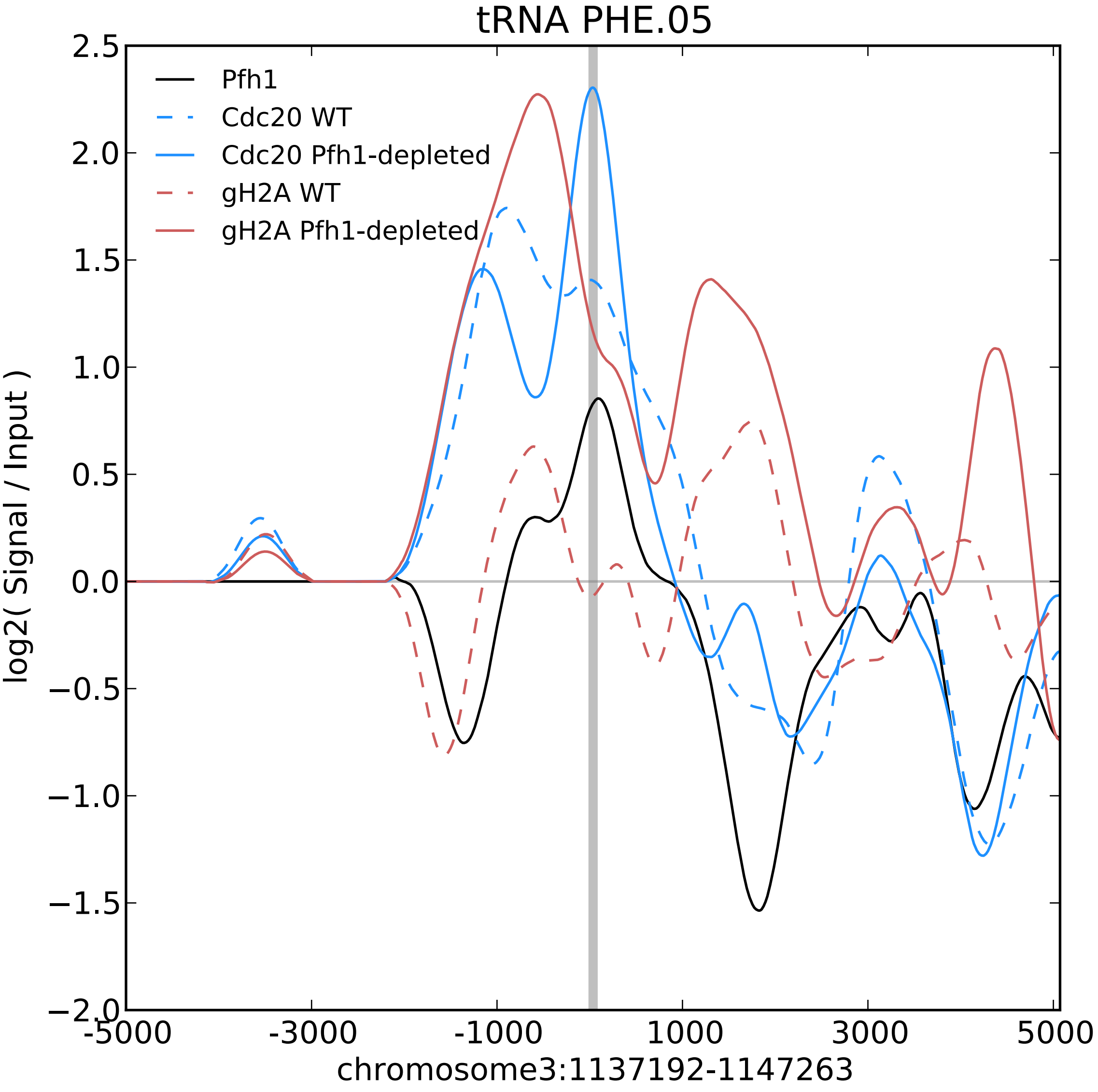

tRNA PHE.05
Pfh1
Cdc20 WT
Cdc20 Pfh1-depleted
gH2A WT
gH2A Pfh1-depleted
log2(Signal / Input)
chromosome3:1137192-1147263
2.5
2.0
1.5
1.0
0.5
0.0
−0.5
−1.0
−1.5
−2.0
-5000
-3000
-1000
1000
3000
5000

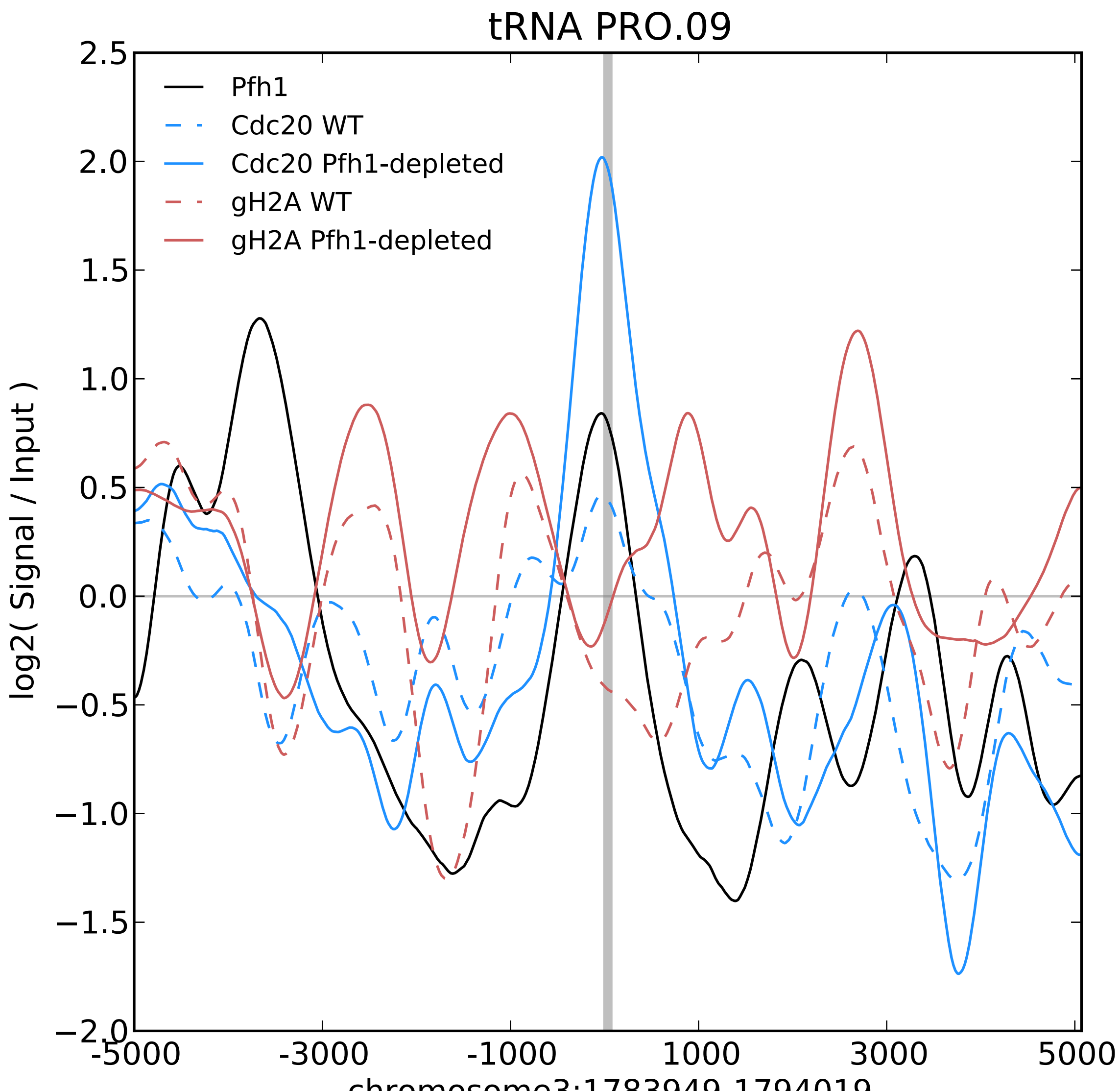

tRNA PRO.09
Pfh1
Cdc20 WT
Cdc20 Pfh1-depleted
gH2A WT
gH2A Pfh1-depleted
log2(Signal / Input)
2.5
2.0
1.5
1.0
0.5
0.0
−0.5
−1.0
−1.5
−2.0
-5000
-3000
-1000
1000
3000
5000
chromosome3:1783949-1794019

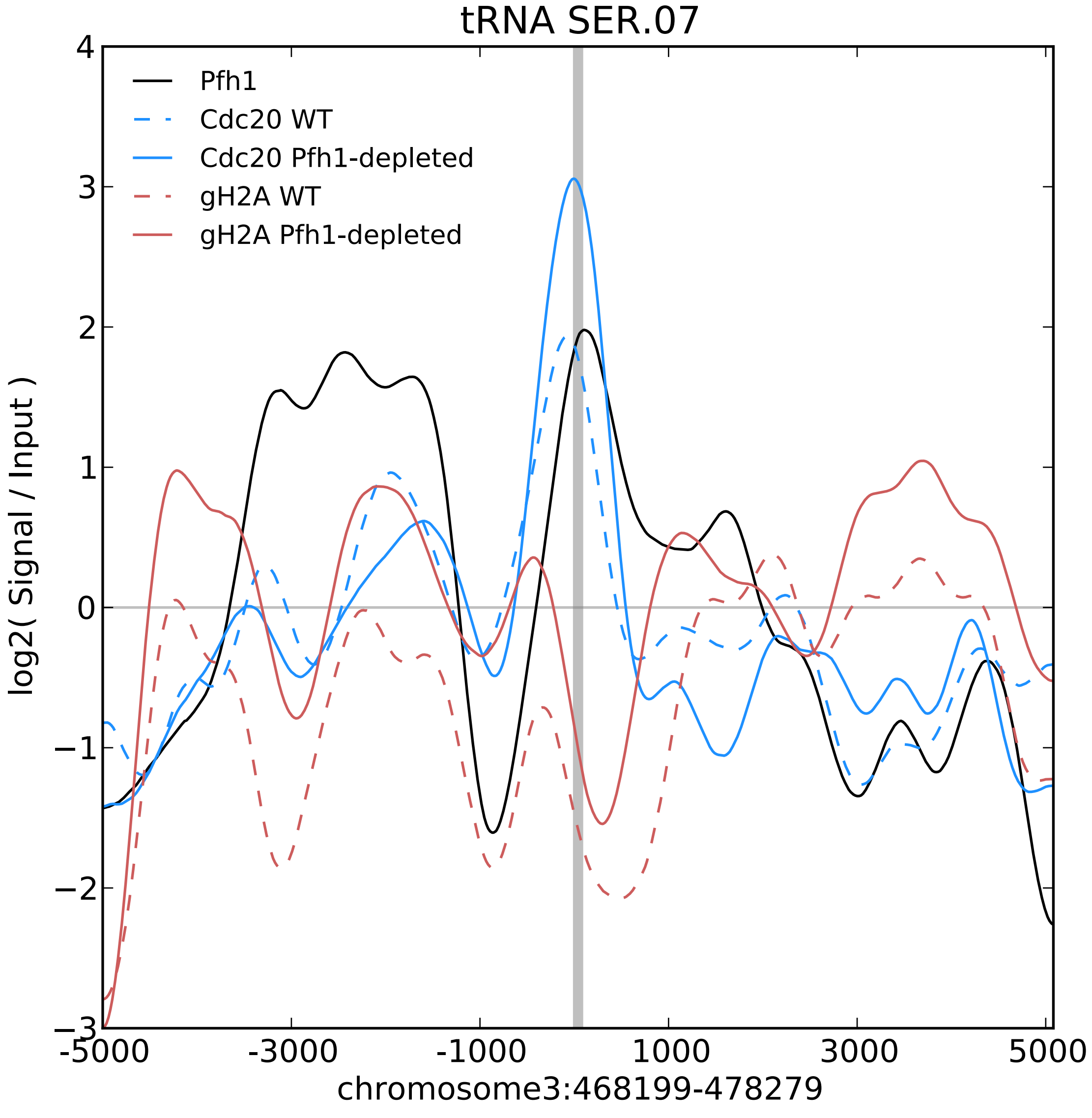

tRNA SER.07
Pfh1
Cdc20 WT
Cdc20 Pfh1-depleted
gH2A WT
gH2A Pfh1-depleted
log2(Signal / Input)
4
3
2
1
0
−1
−2
−3
-5000
-3000
-1000
1000
3000
5000
chromosome3:468199-478279

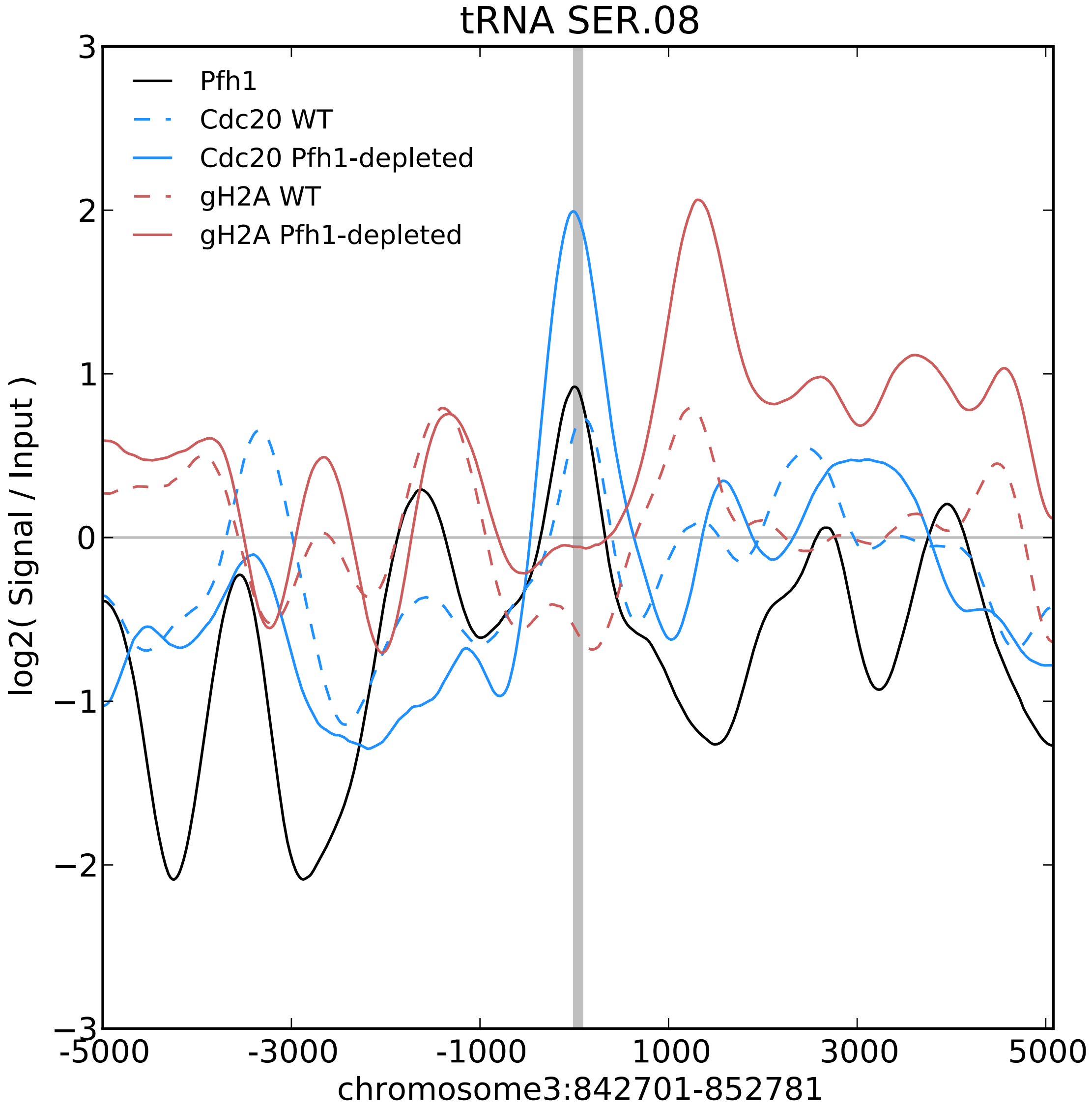

tRNA SER.08
Pfh1
Cdc20 WT
Cdc20 Pfh1-depleted
gH2A WT
gH2A Pfh1-depleted
log2(Signal / Input)
3
2
1
0
−1
−2
−3
-5000
-3000
-1000
1000
3000
5000
chromosome3:842701-852781

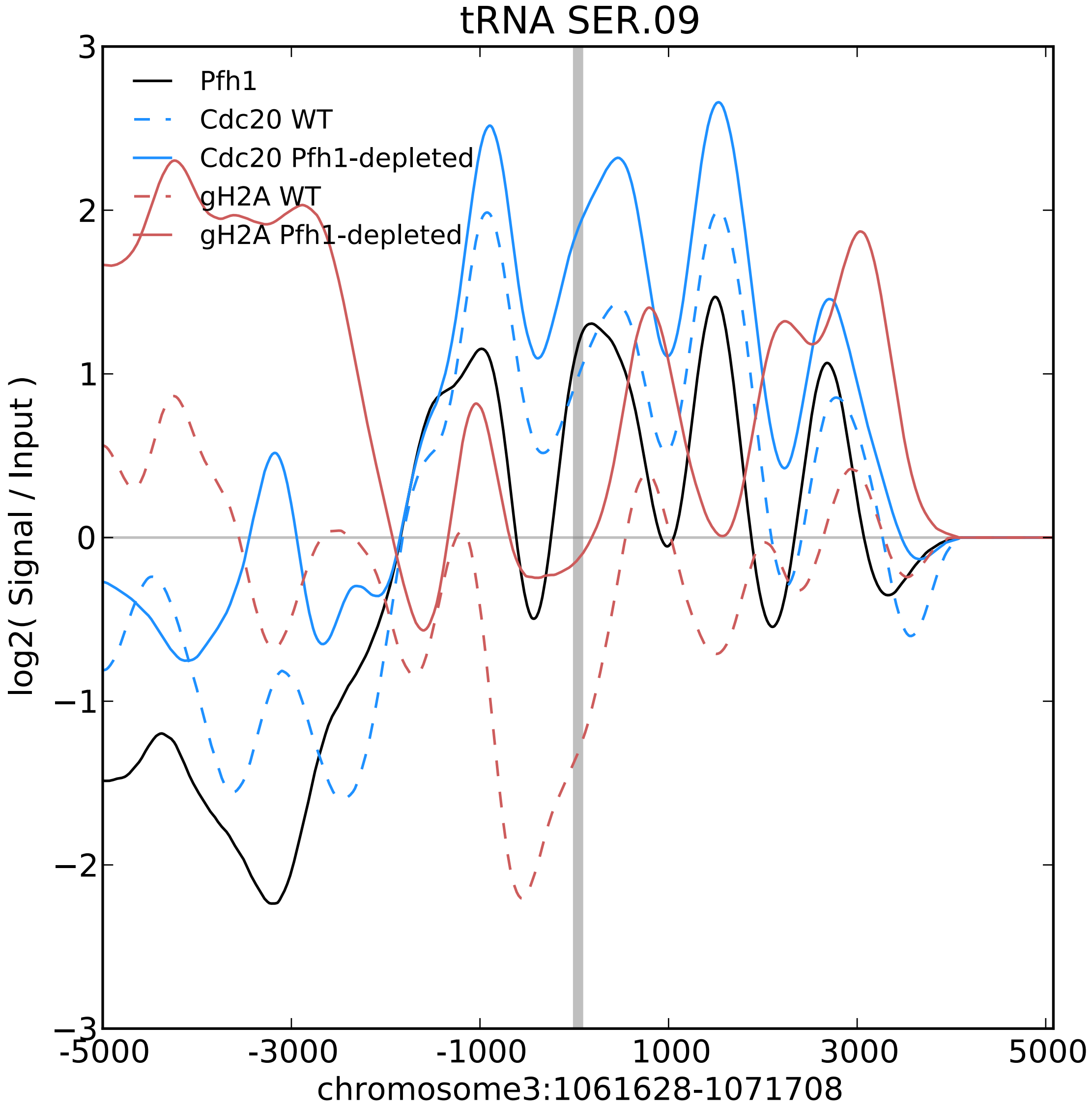

tRNA SER.09
Pfh1
Cdc20 WT
Cdc20 Pfh1-depleted
gH2A WT
gH2A Pfh1-depleted
log2(Signal / Input)
3
2
1
0
−1
−2
−3
-5000
-3000
-1000
1000
3000
5000
chromosome3:1061628-1071708

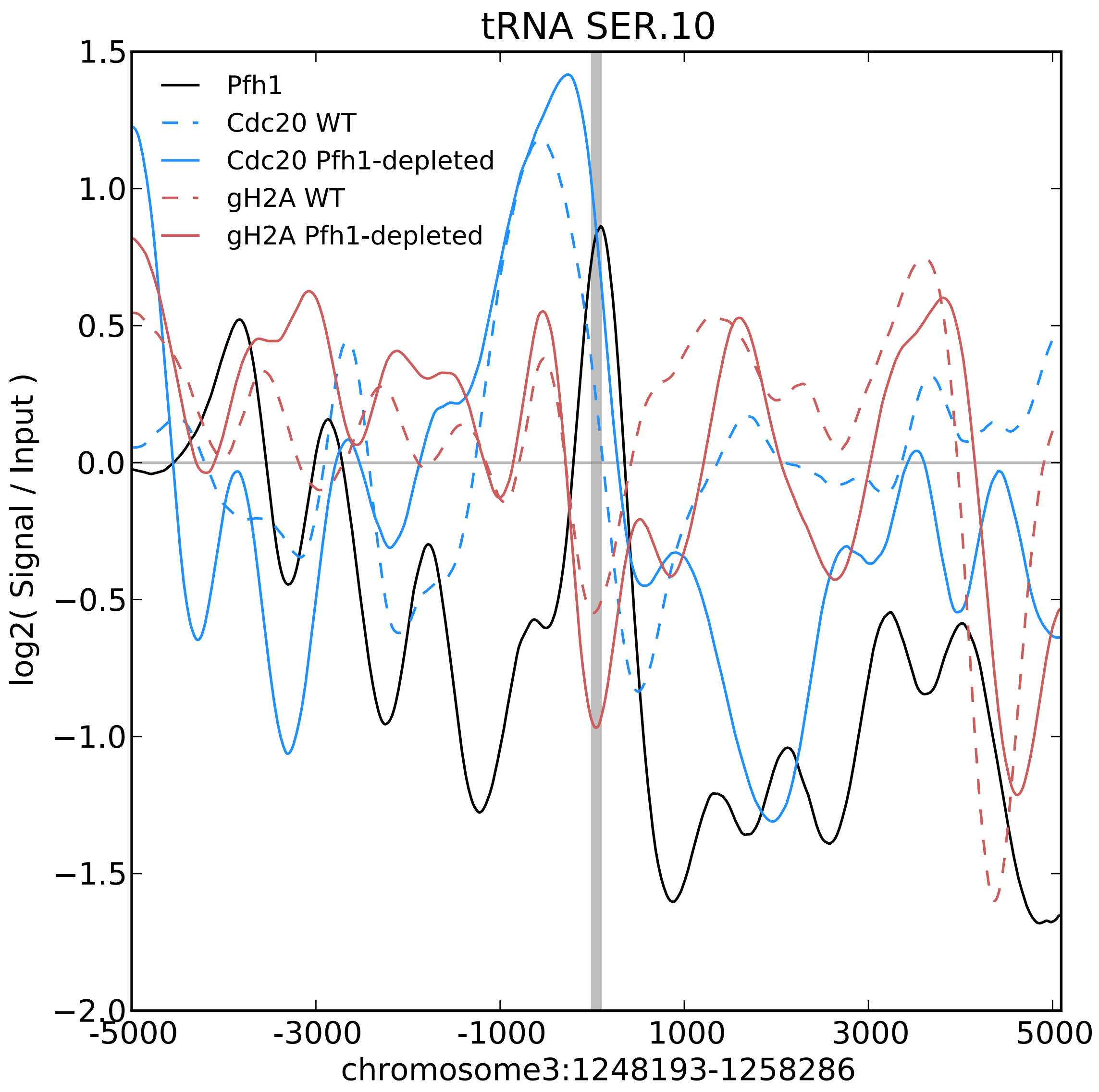

tRNA SER.10
Pfh1
Cdc20 WT
Cdc20 Pfh1-depleted
gH2A WT
gH2A Pfh1-depleted
log2(Signal / Input)
1.5
1.0
0.5
0.0
−0.5
−1.0
−1.5
−2.0
-5000
-3000
-1000
1000
3000
5000
chromosome3:1248193-1258286

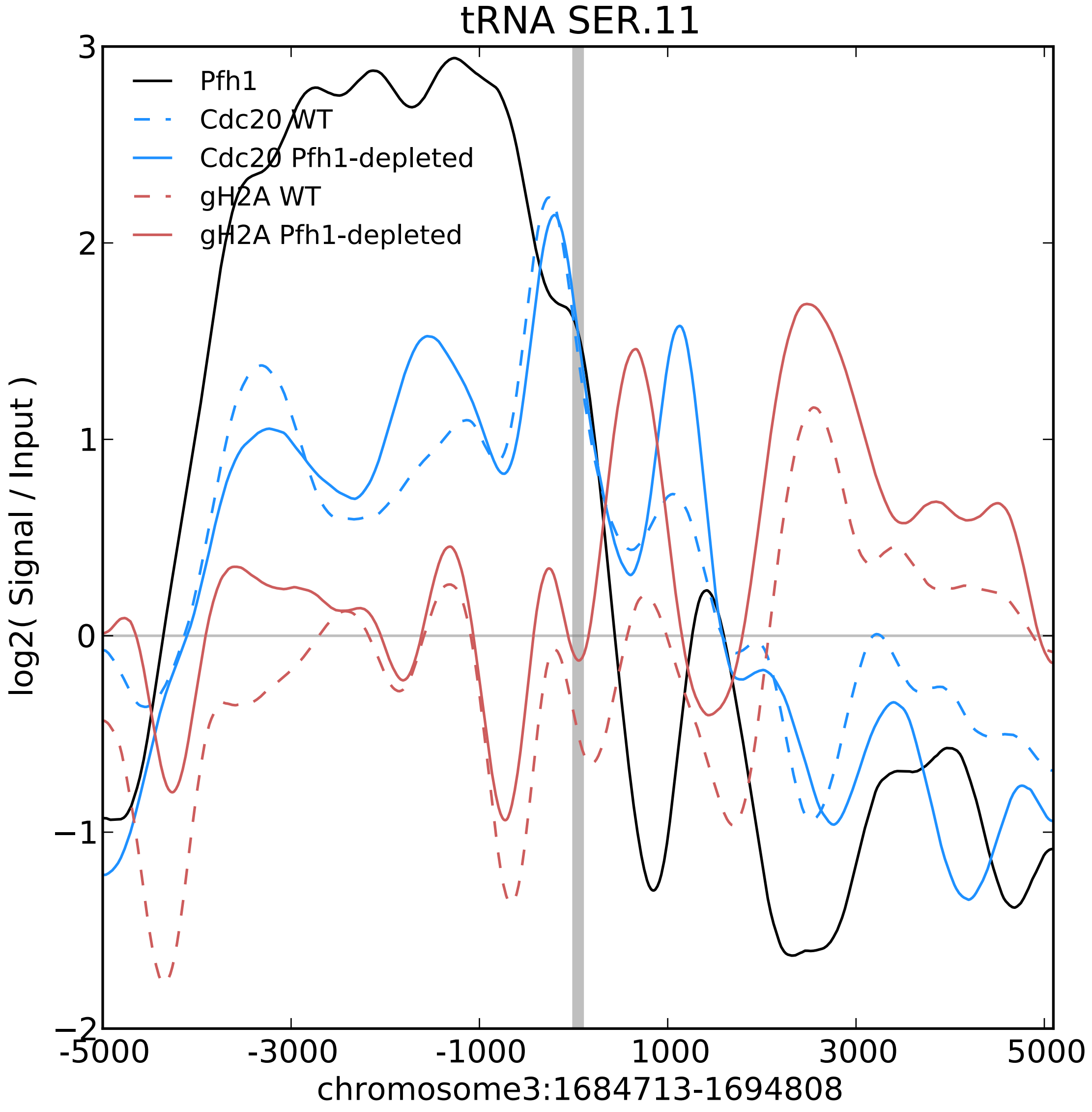

tRNA SER.11
Pfh1
Cdc20 WT
Cdc20 Pfh1-depleted
gH2A WT
gH2A Pfh1-depleted
log2(Signal / Input)
3
2
1
0
−1
−2
-5000
-3000
-1000
1000
3000
5000
chromosome3:1684713-1694808

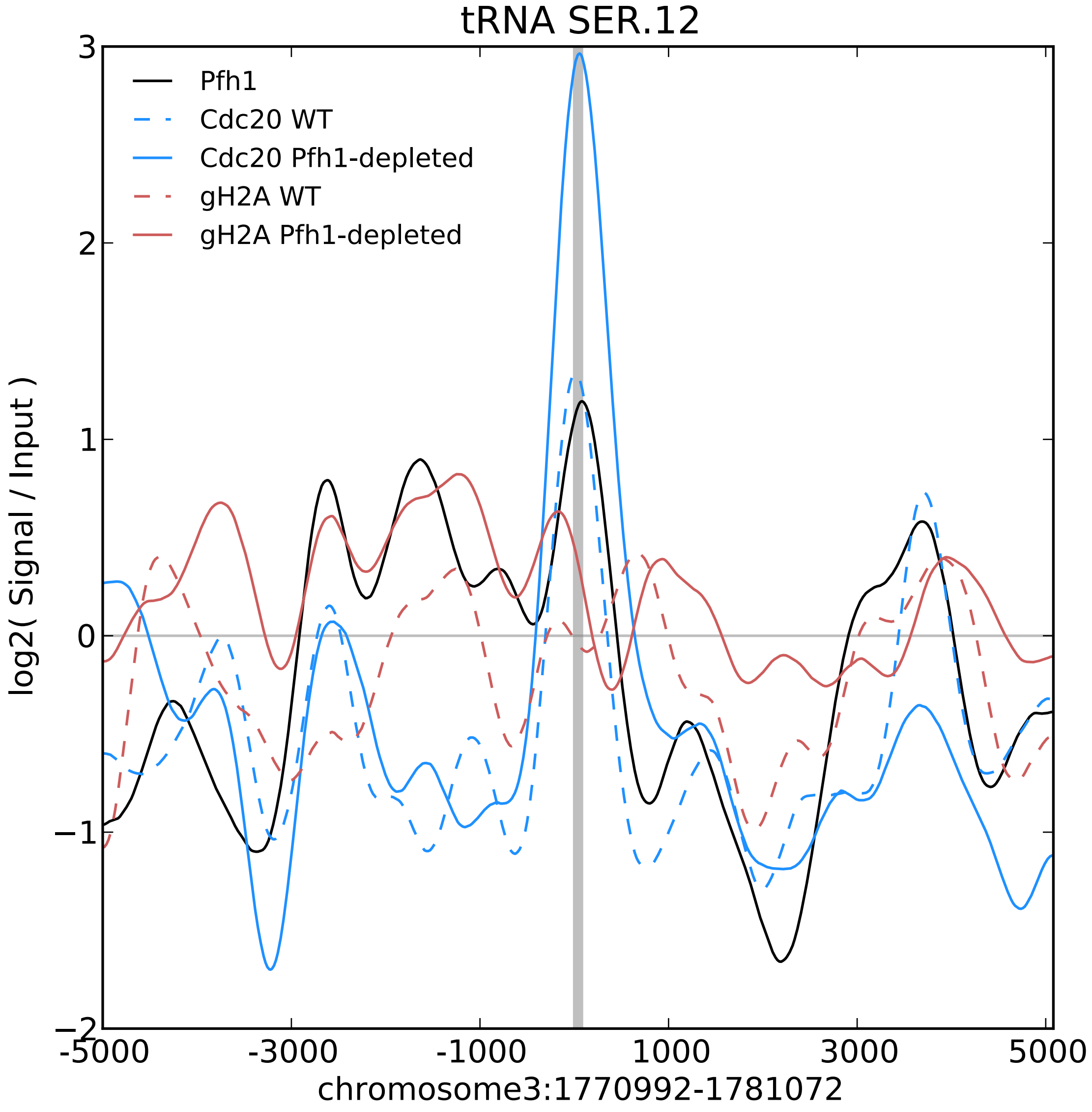
tRNA SER.12
Pfh1
Cdc20 WT
Cdc20 Pfh1-depleted
gH2A WT
gH2A Pfh1-depleted
log2(Signal / Input)
3
2
1
0
−1
−2
-5000
-3000
-1000
1000
3000
5000
chromosome3:1770992-1781072

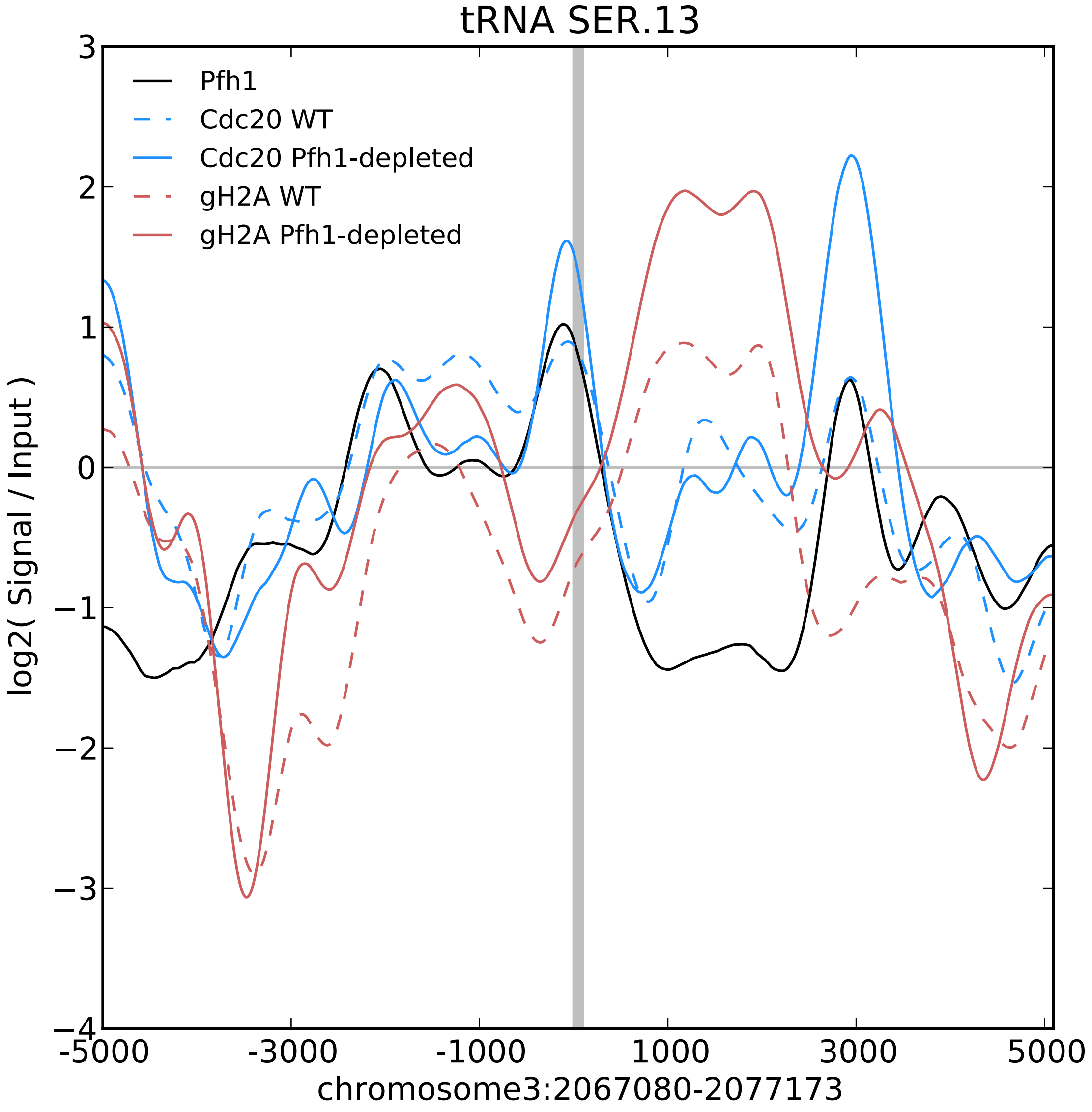
tRNA SER.13
Pfh1
Cdc20 WT
Cdc20 Pfh1-depleted
gH2A WT
gH2A Pfh1-depleted
log2(Signal / Input)
3
2
1
0
−1
−2
−3
−4
-5000
-3000
-1000
1000
3000
5000
chromosome3:2067080-2077173

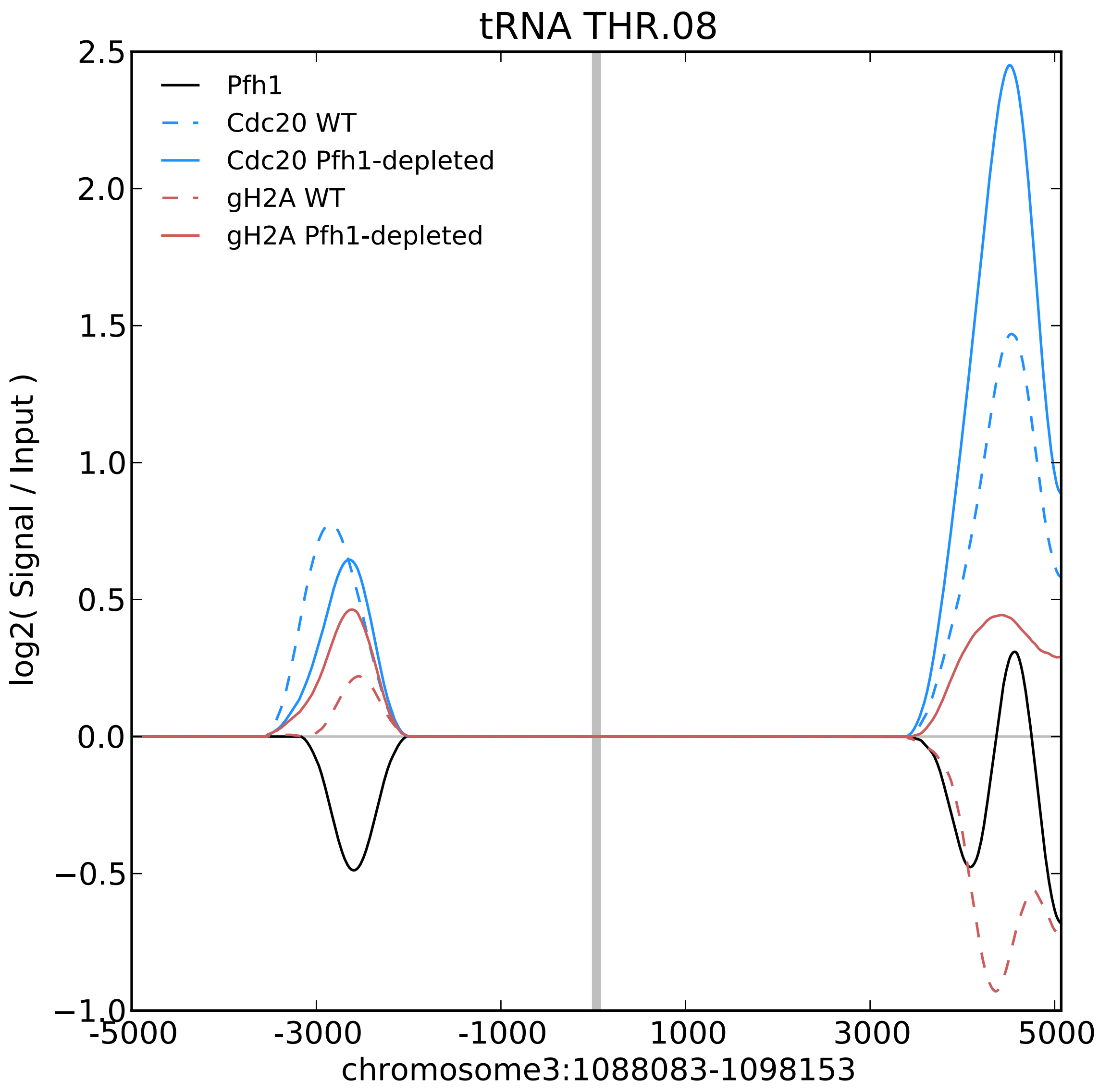

tRNA THR.08
Pfh1
Cdc20 WT
Cdc20 Pfh1-depleted
gH2A WT
gH2A Pfh1-depleted
2.5
2.0
1.5
1.0
0.5
0.0
−0.5
−1.0
log2(Signal / Input)
-5000
-3000
-1000
1000
3000
5000
chromosome3:1088083-1098153

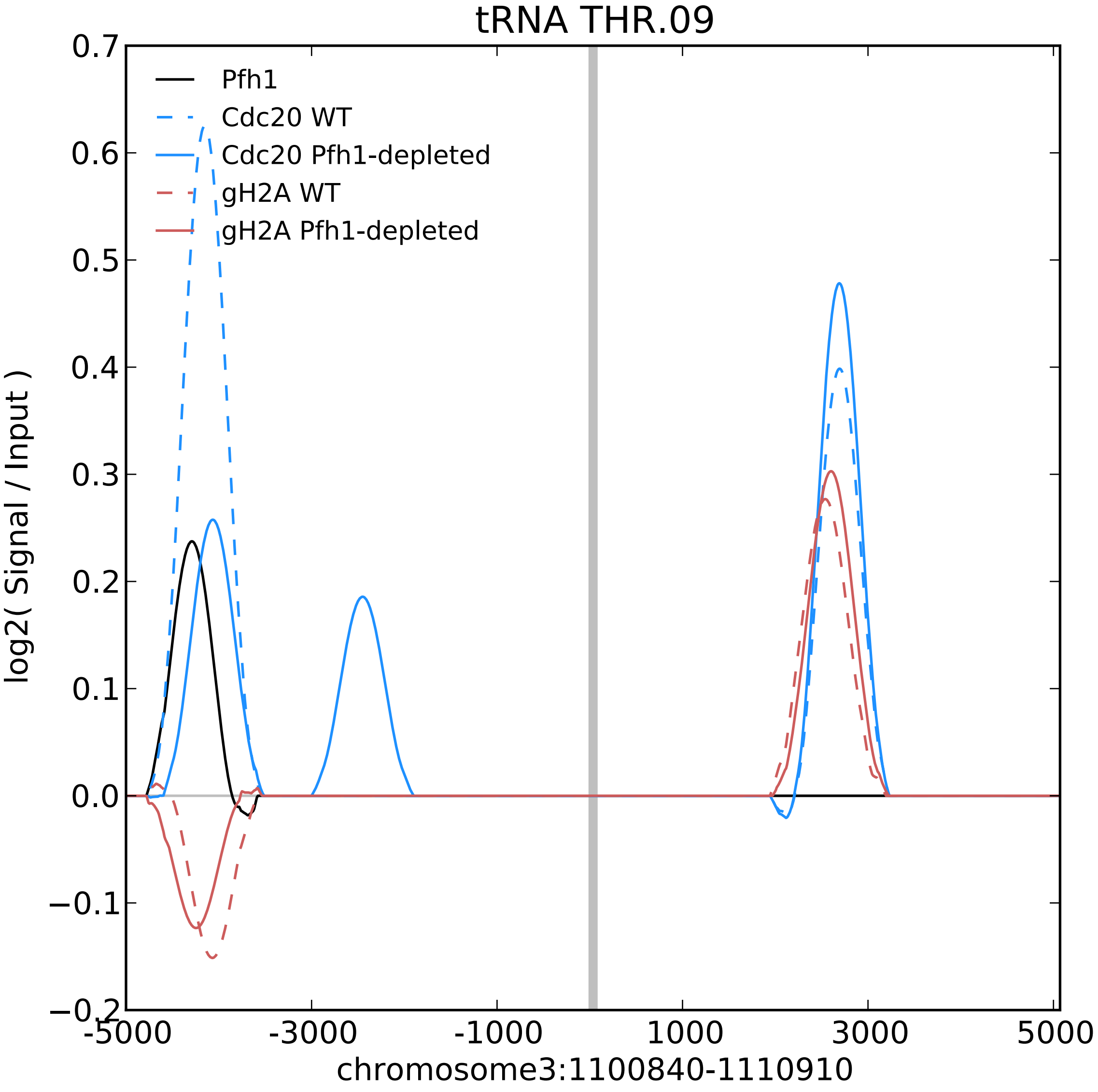
tRNA THR.09
Pfh1
Cdc20 WT
Cdc20 Pfh1-depleted
gH2A WT
gH2A Pfh1-depleted
log2(Signal / Input)
0.7
0.6
0.5
0.4
0.3
0.2
0.1
0.0
−0.1
−0.2
-5000
-3000
-1000
1000
3000
5000
chromosome3:1100840-1110910

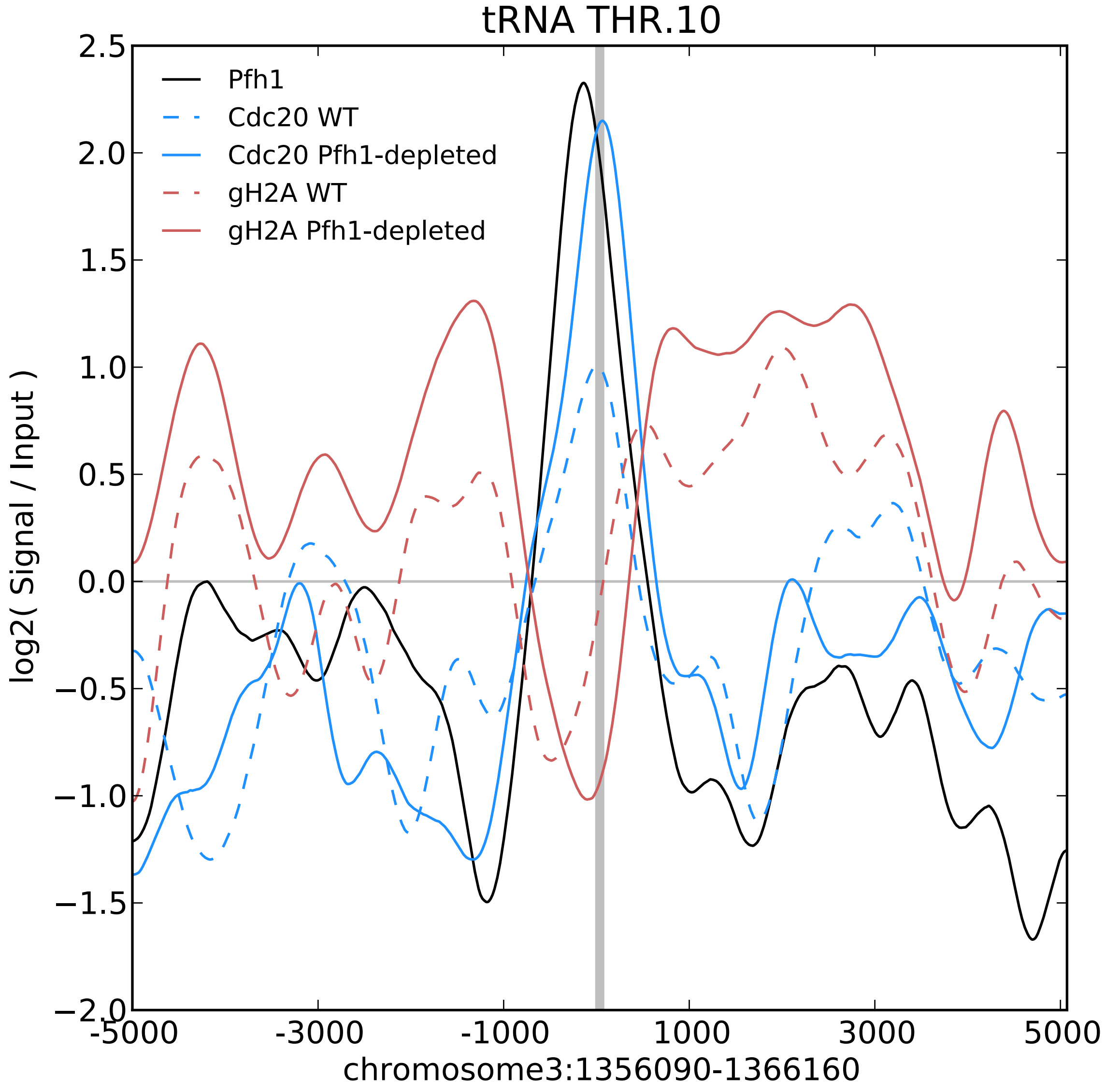

tRNA THR.10
Pfh1
Cdc20 WT
Cdc20 Pfh1-depleted
gH2A WT
gH2A Pfh1-depleted
2.5
2.0
1.5
1.0
0.5
0.0
−0.5
−1.0
−1.5
−2.0
log2(Signal / Input)
-5000
-3000
-1000
1000
3000
5000
chromosome3:1356090-1366160

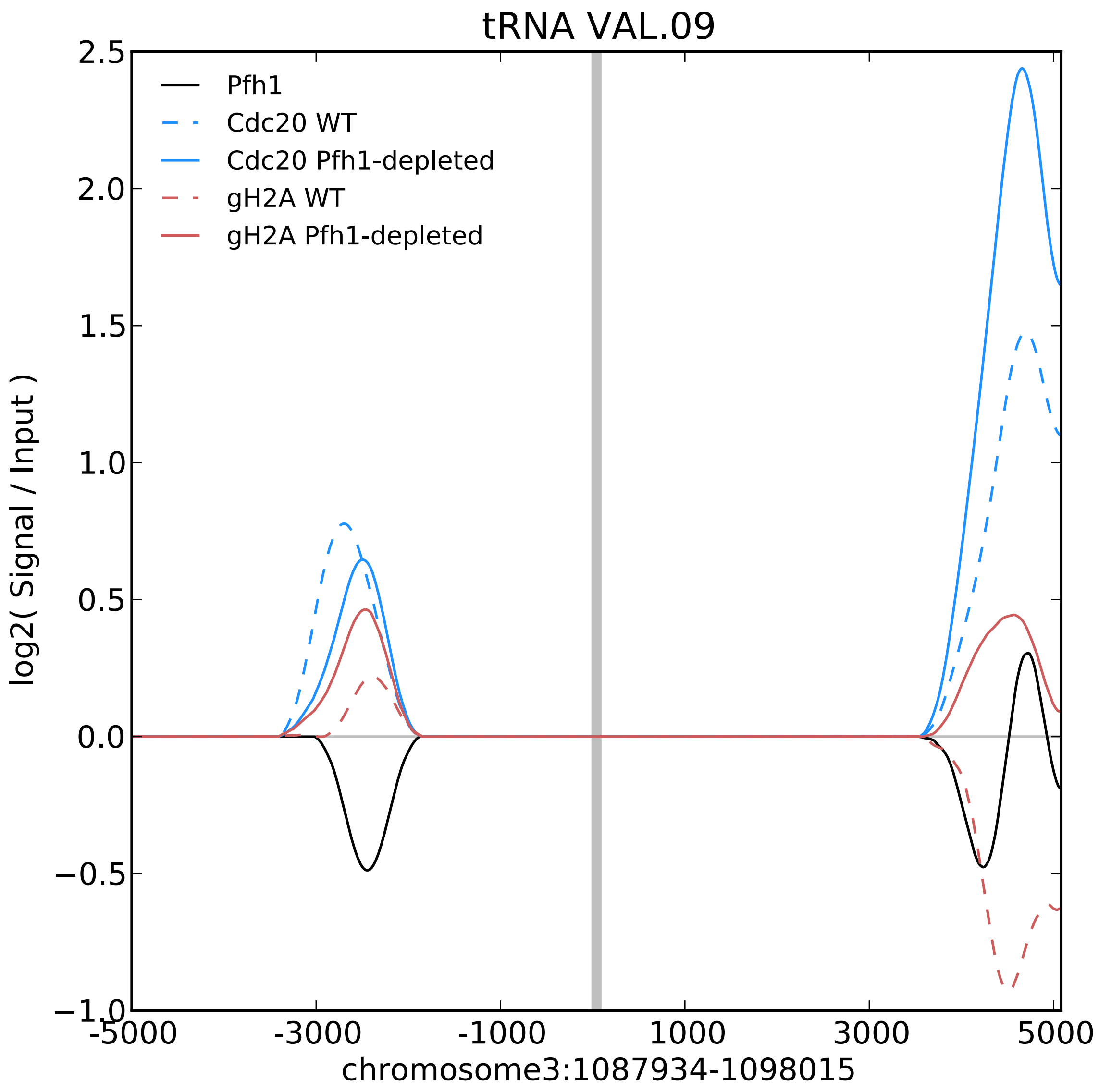
tRNA VAL.09
Pfh1
Cdc20 WT
Cdc20 Pfh1-depleted
gH2A WT
gH2A Pfh1-depleted
2.5
2.0
1.5
1.0
0.5
0.0
−0.5
−1.0
-5000
-3000
-1000
1000
3000
5000
log2(Signal / Input)
chromosome3:1087934-1098015

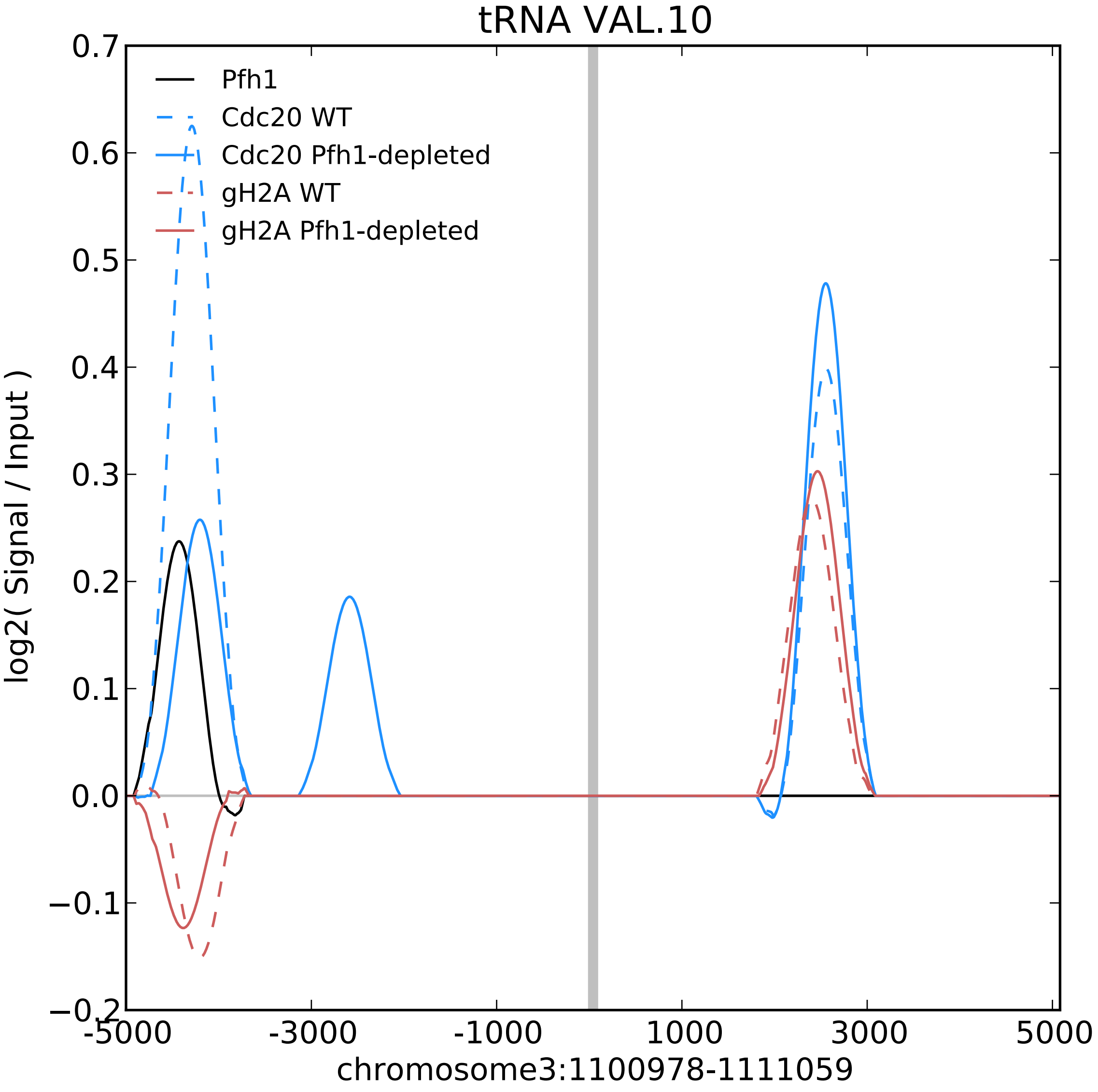

tRNA VAL.10
Pfh1
Cdc20 WT
Cdc20 Pfh1-depleted
gH2A WT
gH2A Pfh1-depleted
0.7
0.6
0.5
0.4
0.3
0.2
0.1
0.0
−0.1
−0.2
log2(Signal / Input)
-5000
-3000
-1000
1000
3000
5000
chromosome3:1100978-1111059

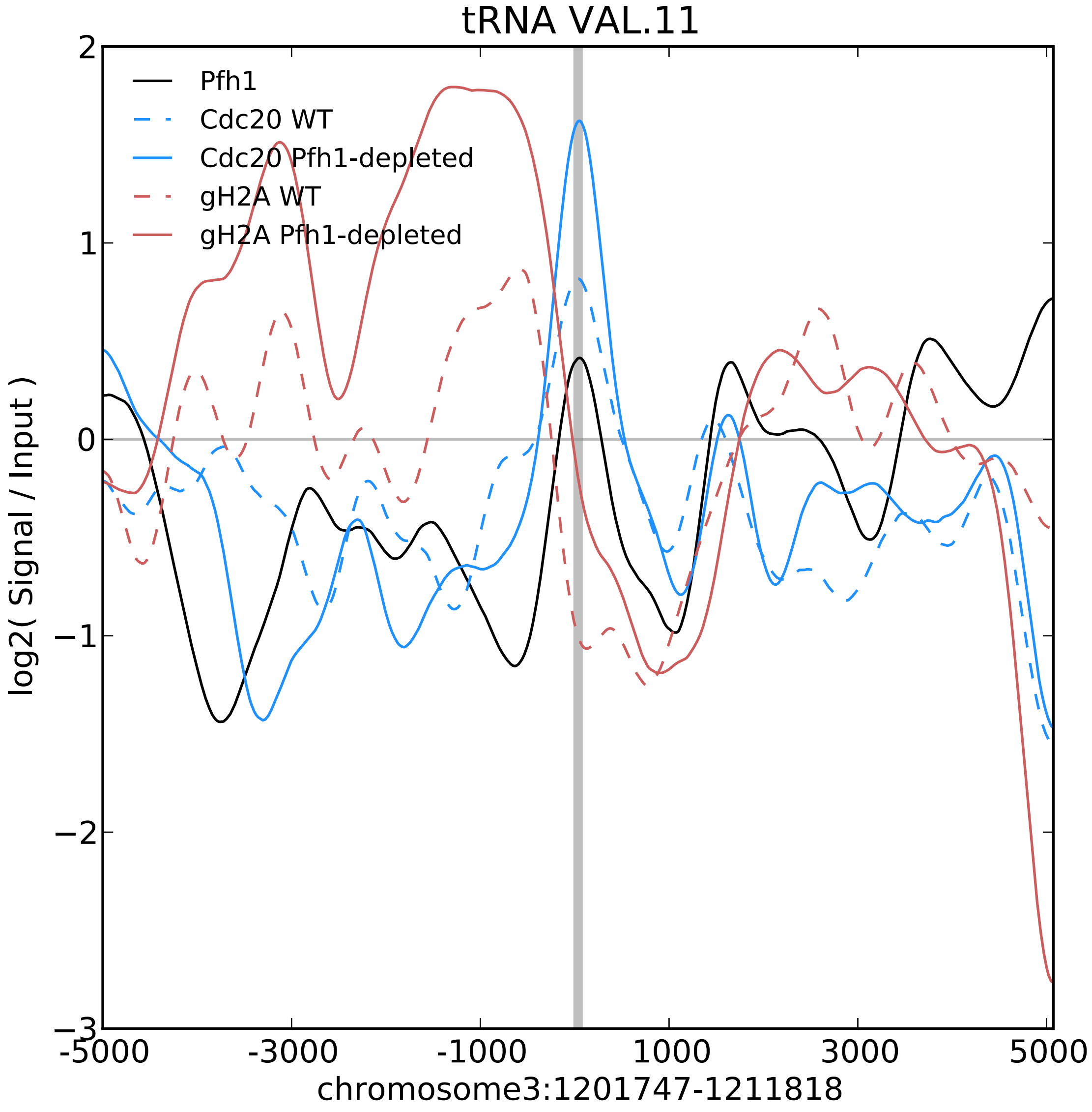

tRNA VAL.11
Pfh1
Cdc20 WT
Cdc20 Pfh1-depleted
gH2A WT
gH2A Pfh1-depleted
log2(Signal / Input)
2
1
0
−1
−2
−3
-5000
-3000
-1000
1000
3000
5000
chromosome3:1201747-1211818

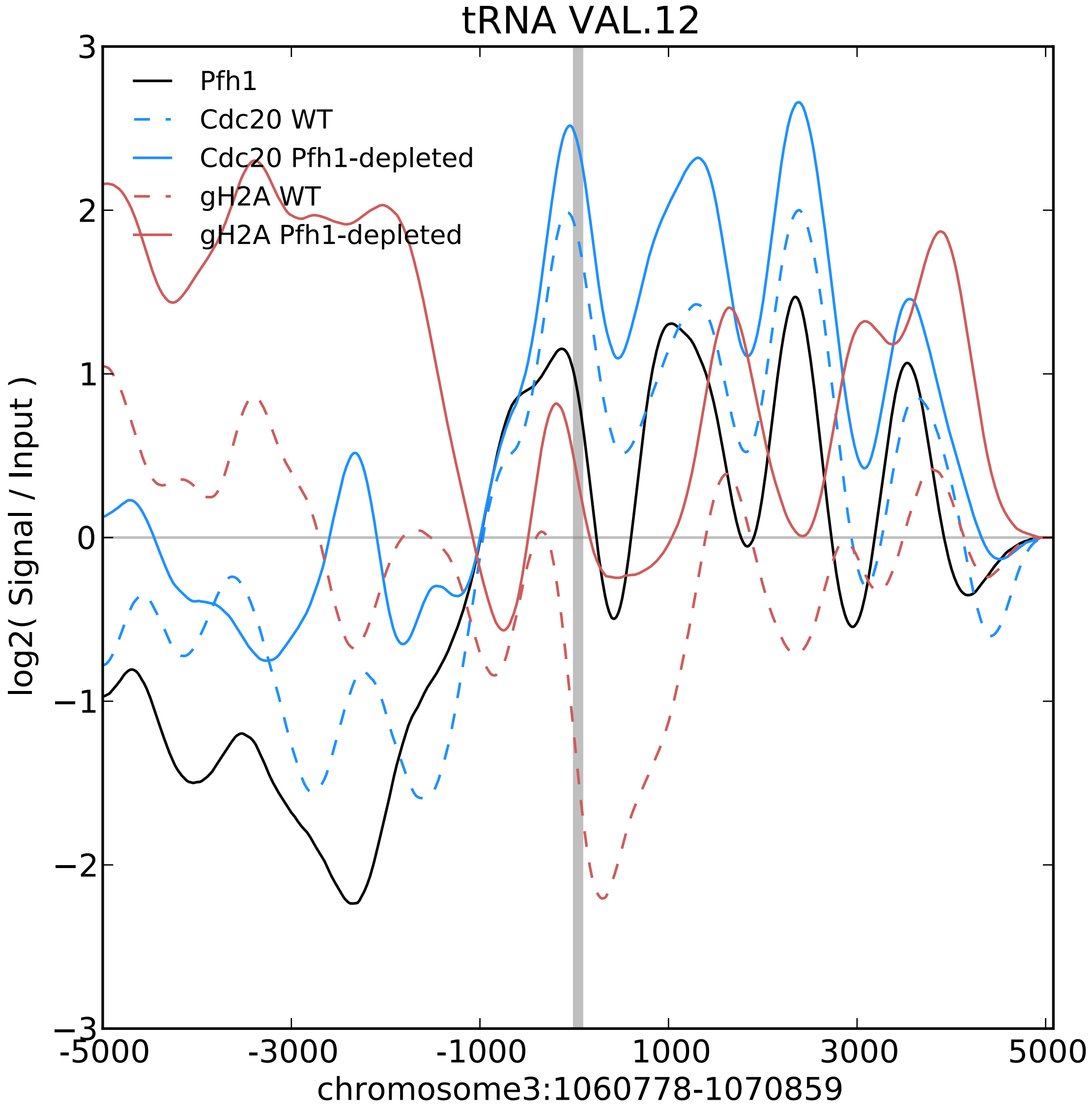

tRNA VAL.12
Pfh1
Cdc20 WT
Cdc20 Pfh1-depleted
gH2A WT
gH2A Pfh1-depleted
log2(Signal / Input)
3
2
1
0
−1
−2
−3
-5000
-3000
-1000
1000
3000
5000
chromosome3:1060778-1070859
