# Supplementary material for: Pfh1 Is an Accessory Replicative Helicase that Interacts with the Replisome to Facilitate Fork Progression and Preserve Genome Integrity
Source: PLoS Genet. 2016 Sep 9;12(9):e1006238. doi: 10.1371/journal.pgen.1006238 (PMC5017727; doi:10.1371/journal.pgen.1006238)
Supplement: S4 Fig — Details are as in Fig 3. (PDF) [file pgen.1006238.s004.pdf]

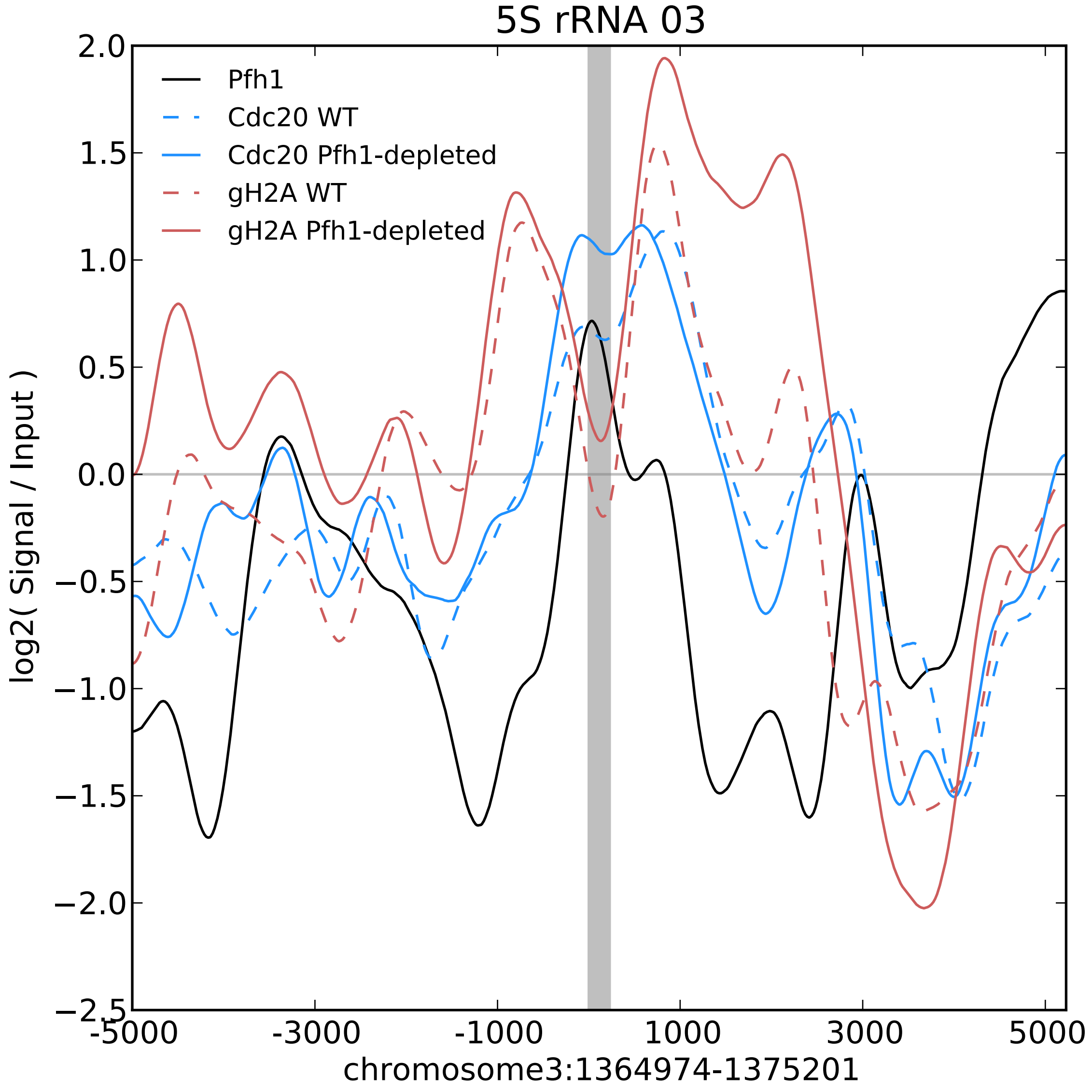

5S rRNA 03
Pfh1
Cdc20 WT
Cdc20 Pfh1-depleted
gH2A WT
gH2A Pfh1-depleted
log2(Signal / Input)
2.0
1.5
1.0
0.5
0.0
−0.5
−1.0
−1.5
−2.0
−2.5
-5000
-3000
-1000
1000
3000
5000
chromosome3:1364974-1375201

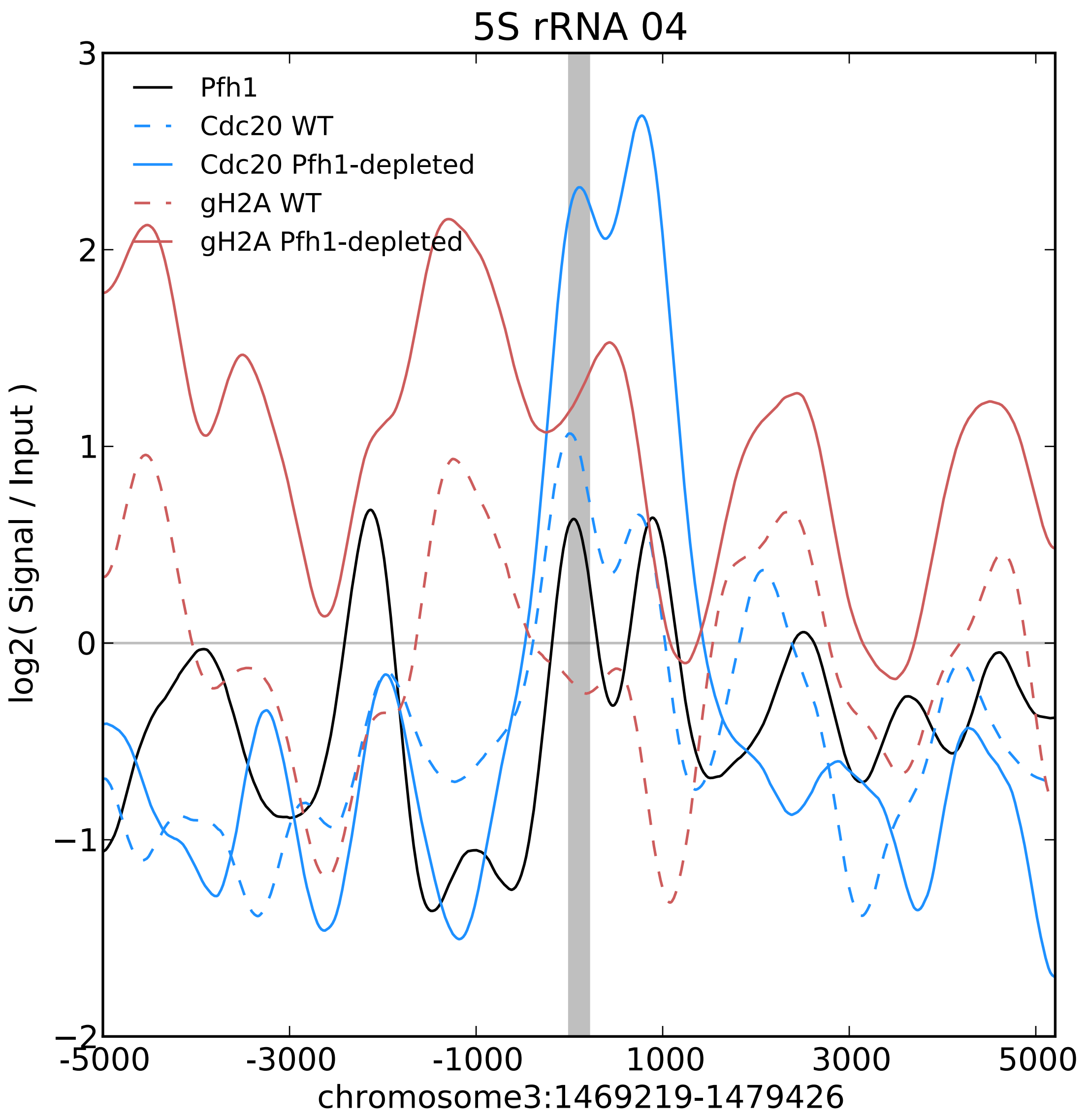
5S rRNA 04
Pfh1
Cdc20 WT
Cdc20 Pfh1-depleted
gH2A WT
gH2A Pfh1-depleted
log2(Signal / Input)
3
2
1
0
−1
−2
-5000
-3000
-1000
1000
3000
5000
chromosome3:1469219-1479426

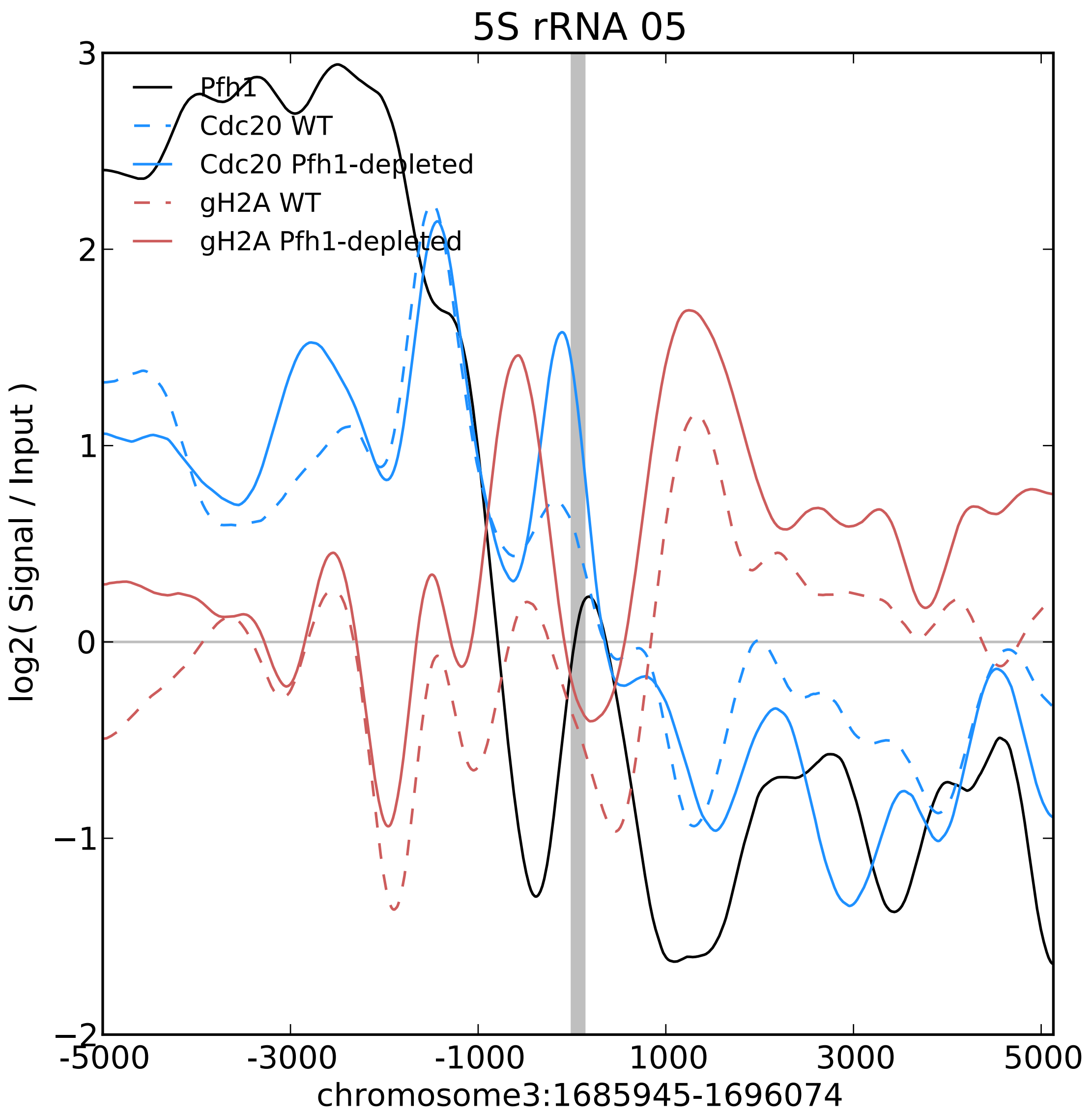

5S rRNA 05
Pfh1
Cdc20 WT
Cdc20 Pfh1-depleted
gH2A WT
gH2A Pfh1-depleted
log2(Signal / Input)
3
2
1
0
−1
−2
-5000
-3000
-1000
1000
3000
5000
chromosome3:1685945-1696074

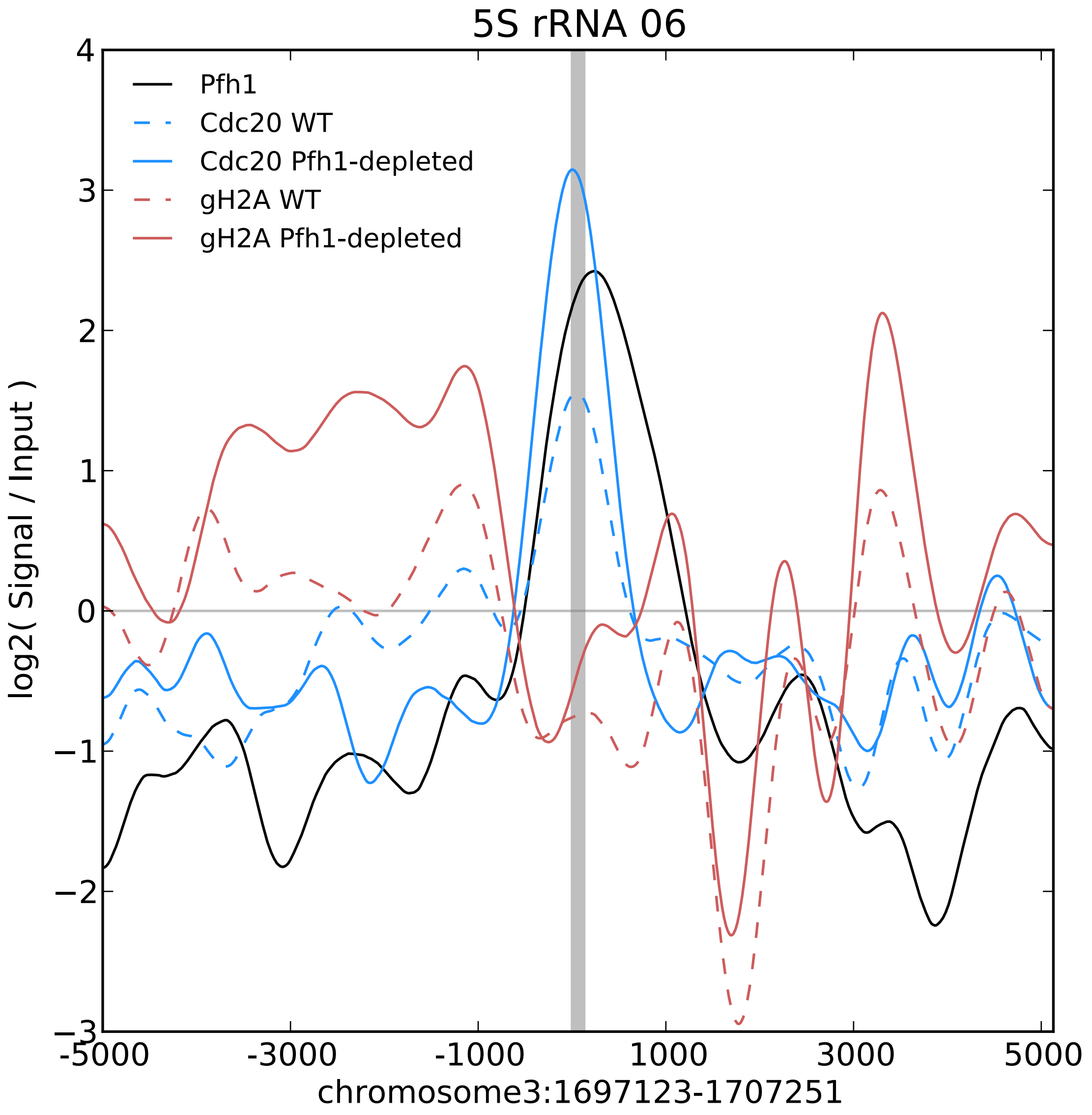
5S rRNA 06
Pfh1
Cdc20 WT
Cdc20 Pfh1-depleted
gH2A WT
gH2A Pfh1-depleted
log2(Signal / Input)
4
3
2
1
0
−1
−2
−3
-5000
-3000
-1000
1000
3000
5000
chromosome3:1697123-1707251

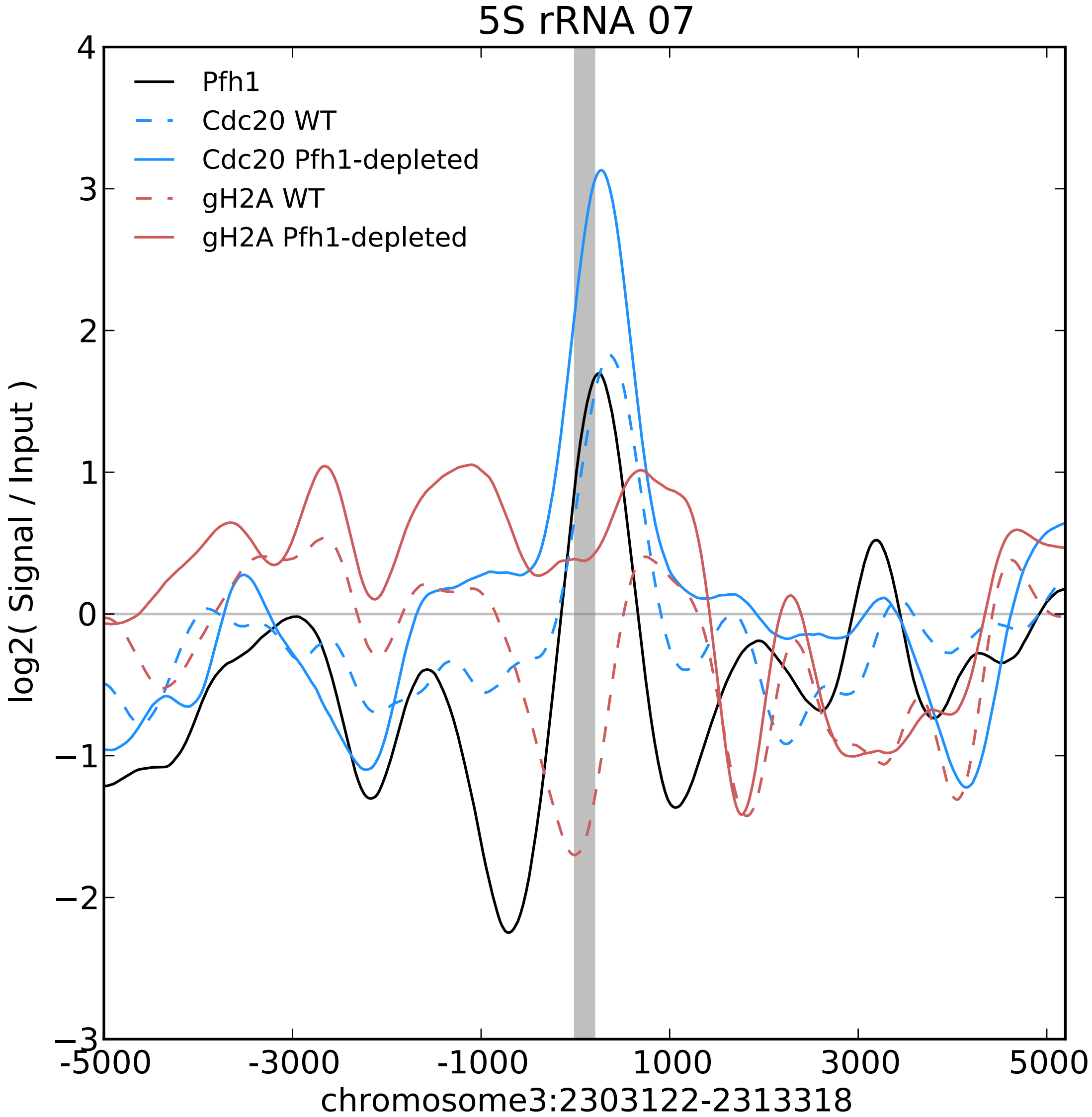

5S rRNA 07
Pfh1
Cdc20 WT
Cdc20 Pfh1-depleted
gH2A WT
gH2A Pfh1-depleted
log2(Signal / Input)
4
3
2
1
0
−1
−2
−3
-5000
-3000
-1000
1000
3000
5000
chromosome3:2303122-2313318

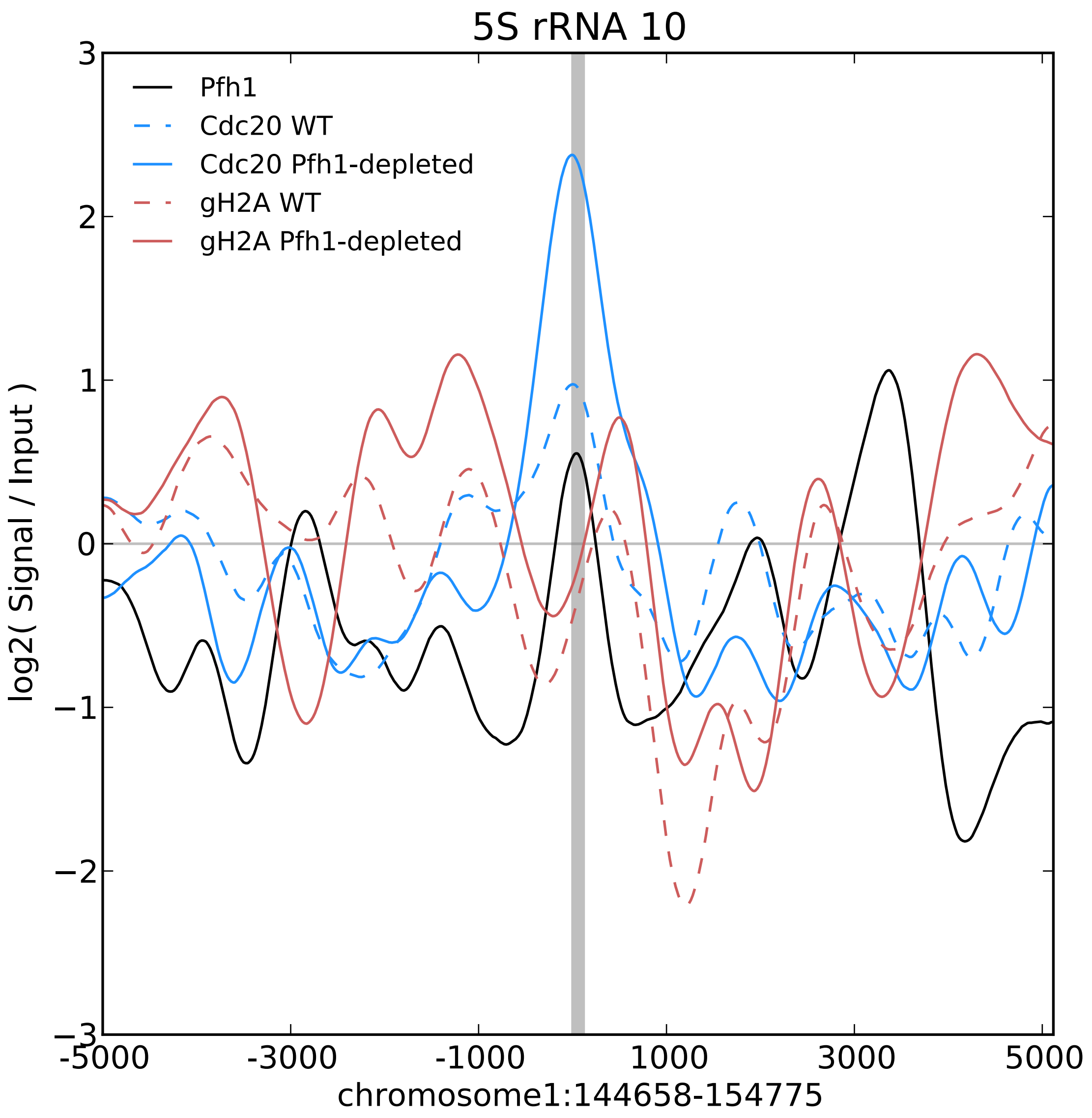

5S rRNA 10
Pfh1
Cdc20 WT
Cdc20 Pfh1-depleted
gH2A WT
gH2A Pfh1-depleted
log2(Signal / Input)
3
2
1
0
−1
−2
−3
-5000
-3000
-1000
1000
3000
5000
chromosome1:144658-154775

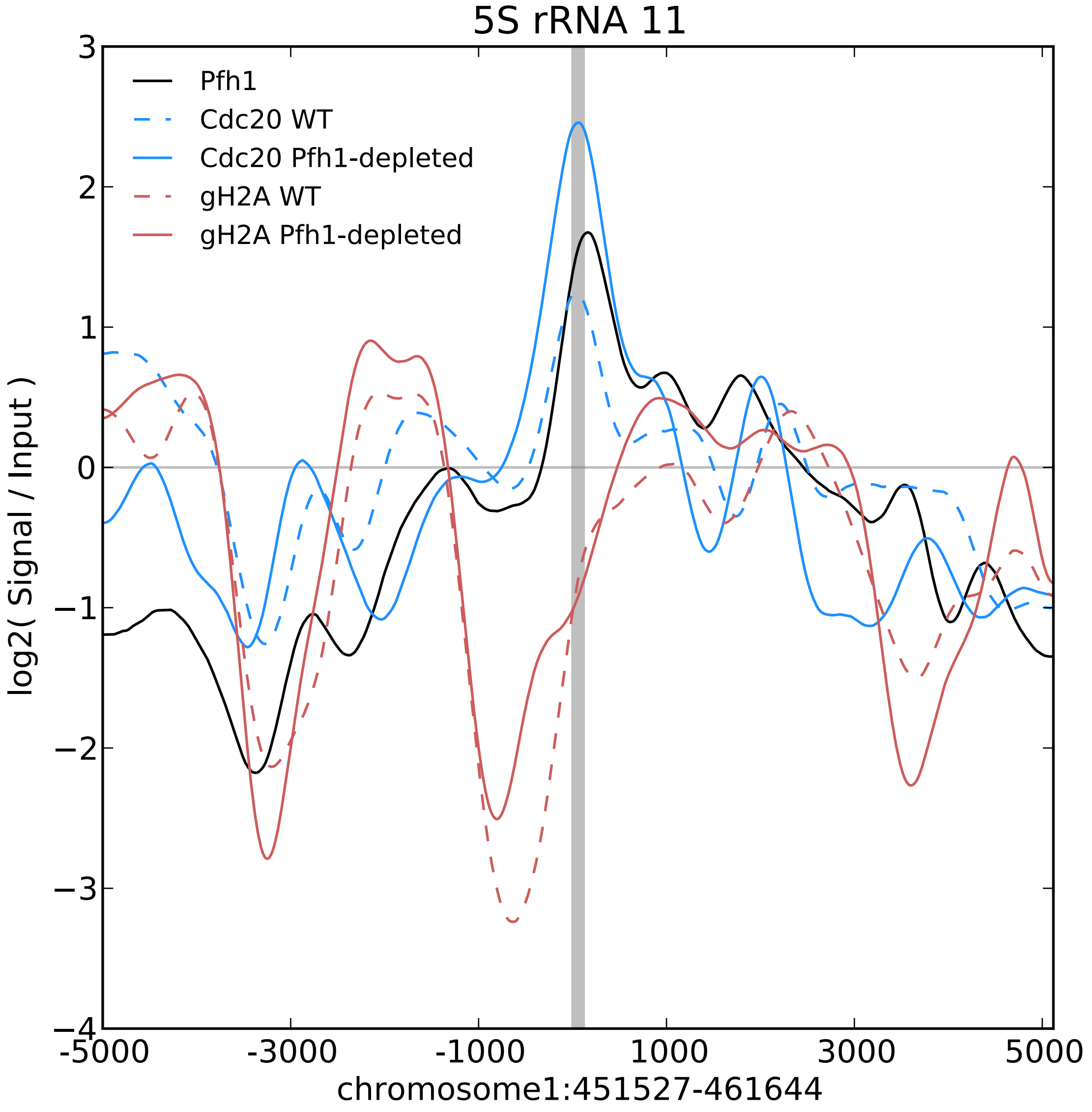
5S rRNA 11
Pfh1
Cdc20 WT
Cdc20 Pfh1-depleted
gH2A WT
gH2A Pfh1-depleted
log2(Signal / Input)
3
2
1
0
−1
−2
−3
−4
-5000
-3000
-1000
1000
3000
5000
chromosome1:451527-461644

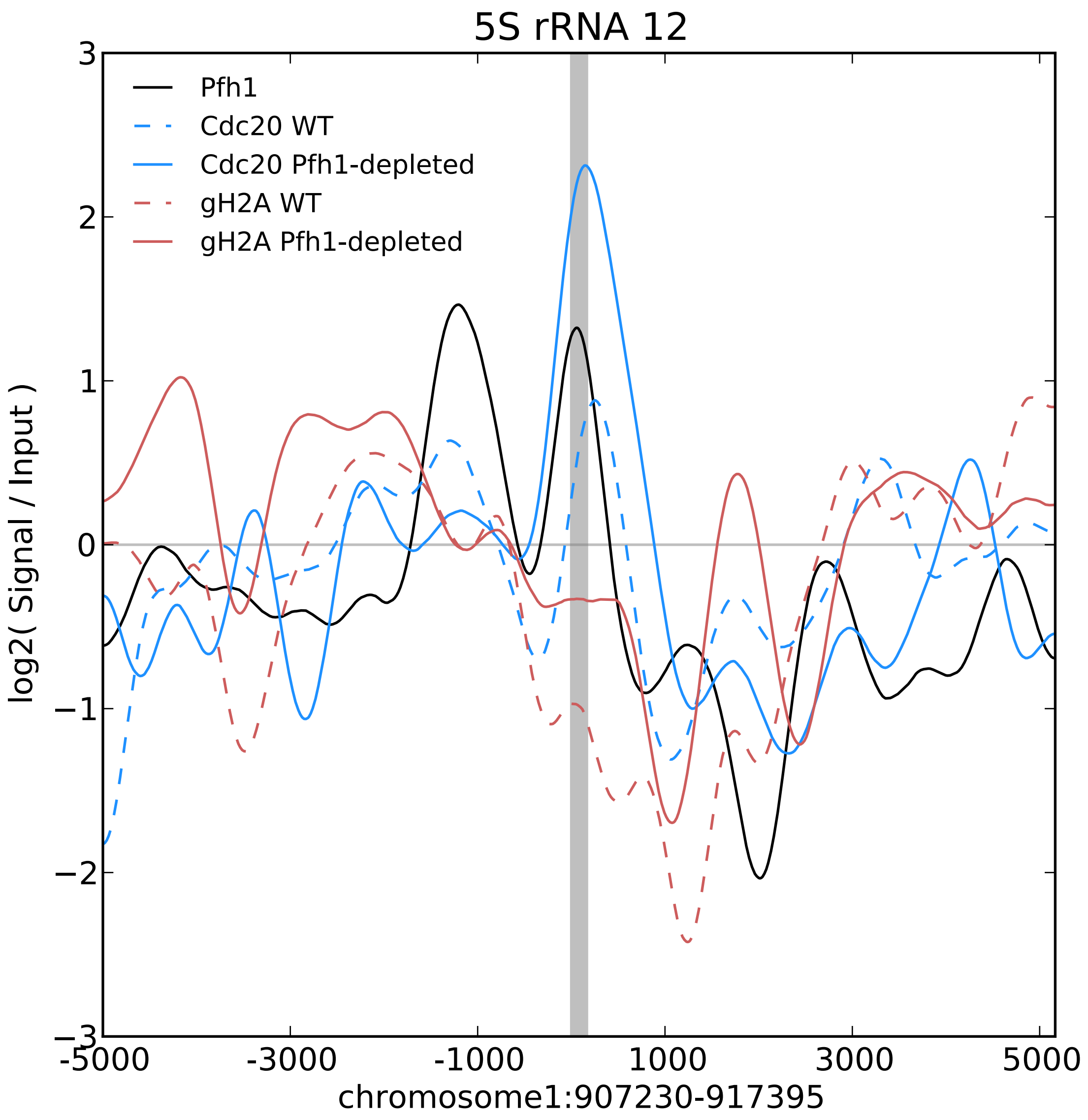

5S rRNA 12
Pfh1
Cdc20 WT
Cdc20 Pfh1-depleted
gH2A WT
gH2A Pfh1-depleted
log2(Signal / Input)
3
2
1
0
−1
−2
−3
-5000
-3000
-1000
1000
3000
5000
chromosome1:907230-917395

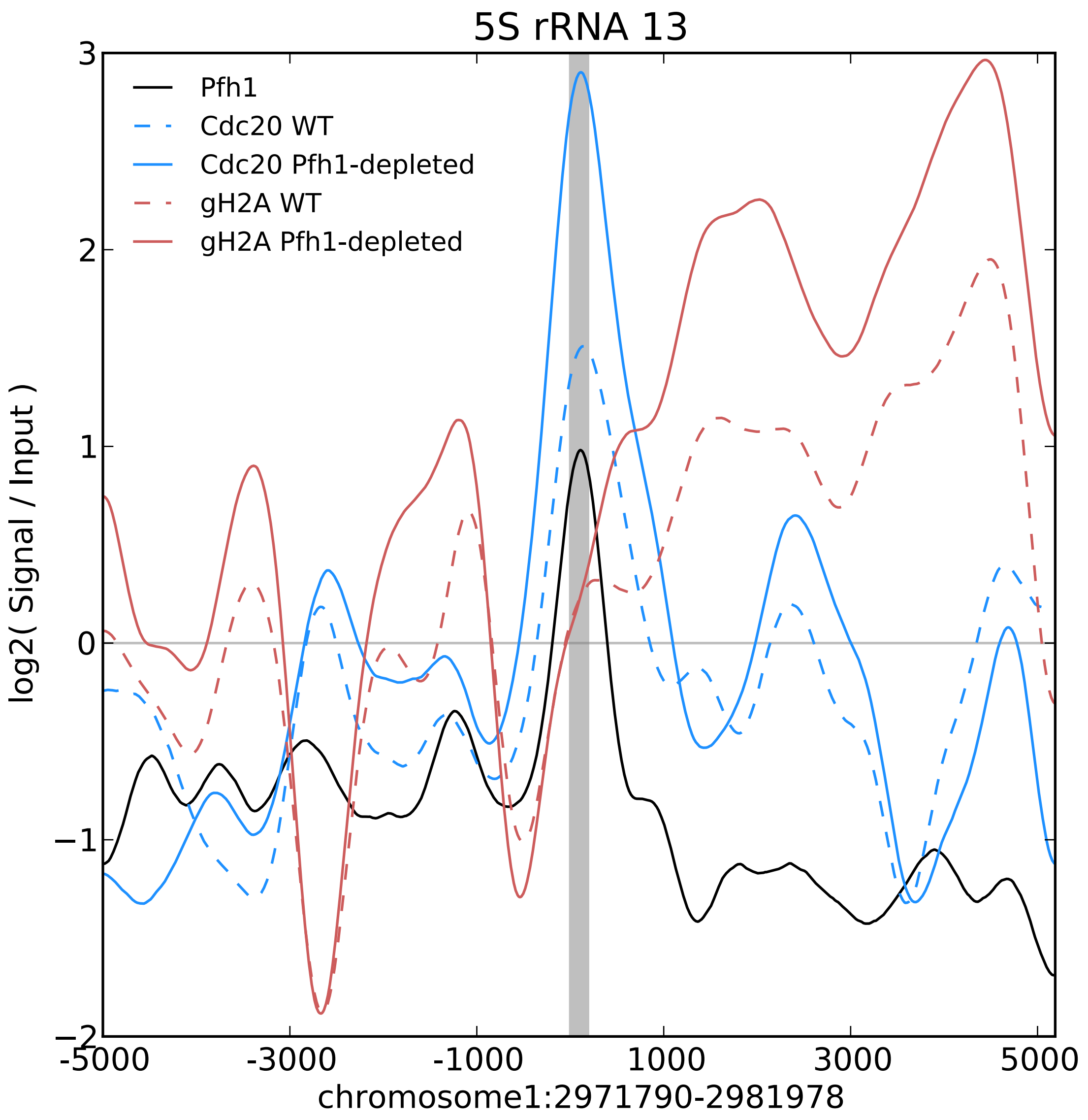

5S rRNA 13
Pfh1
Cdc20 WT
Cdc20 Pfh1-depleted
gH2A WT
gH2A Pfh1-depleted
log2(Signal / Input)
3
2
1
0
−1
−2
-5000
-3000
-1000
1000
3000
5000
chromosome1:2971790-2981978

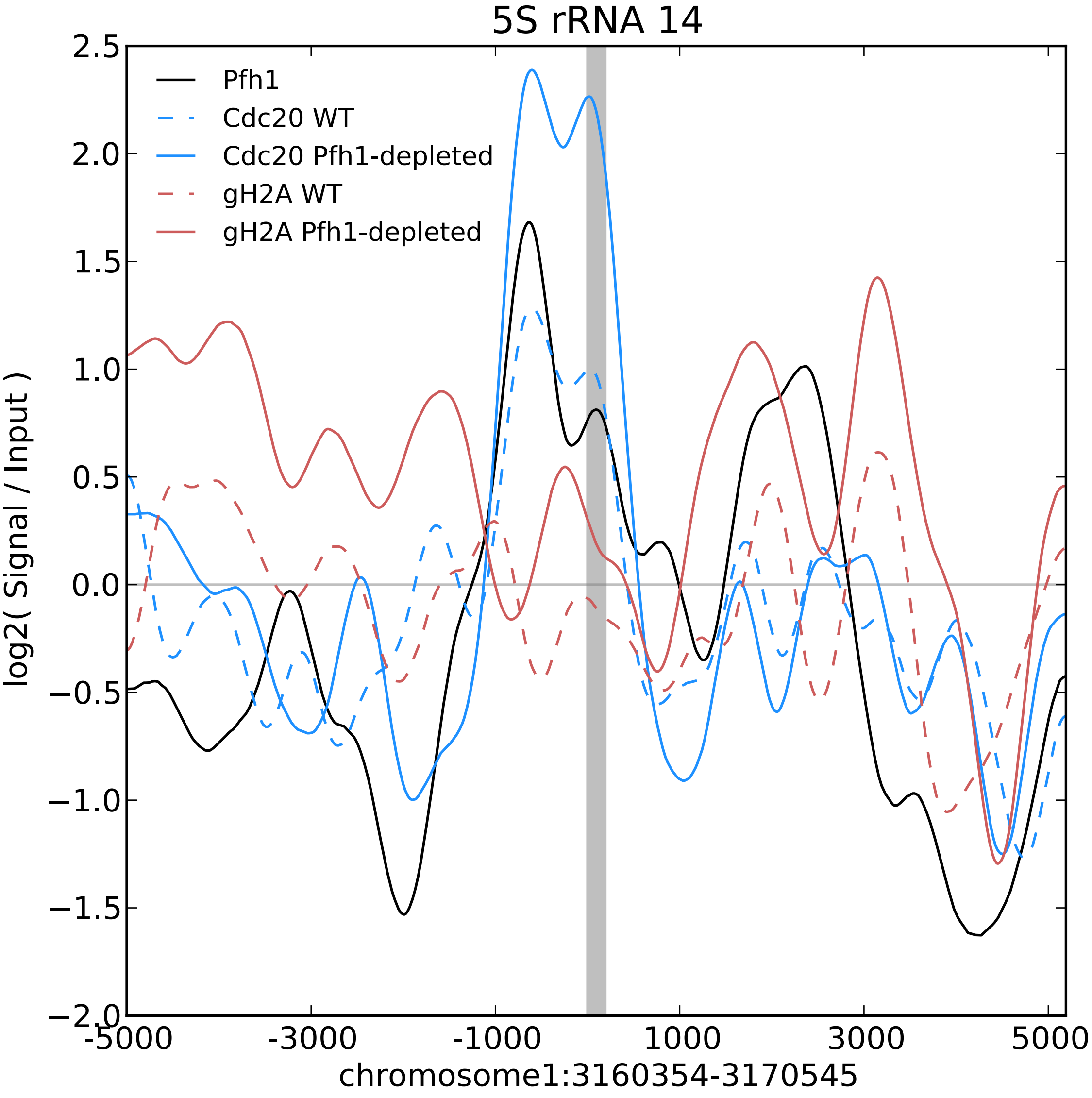

5S rRNA 14
Pfh1
Cdc20 WT
Cdc20 Pfh1-depleted
gH2A WT
gH2A Pfh1-depleted
log2(Signal / Input)
2.5
2.0
1.5
1.0
0.5
0.0
−0.5
−1.0
−1.5
−2.0
-5000
-3000
-1000
1000
3000
5000
chromosome1:3160354-3170545

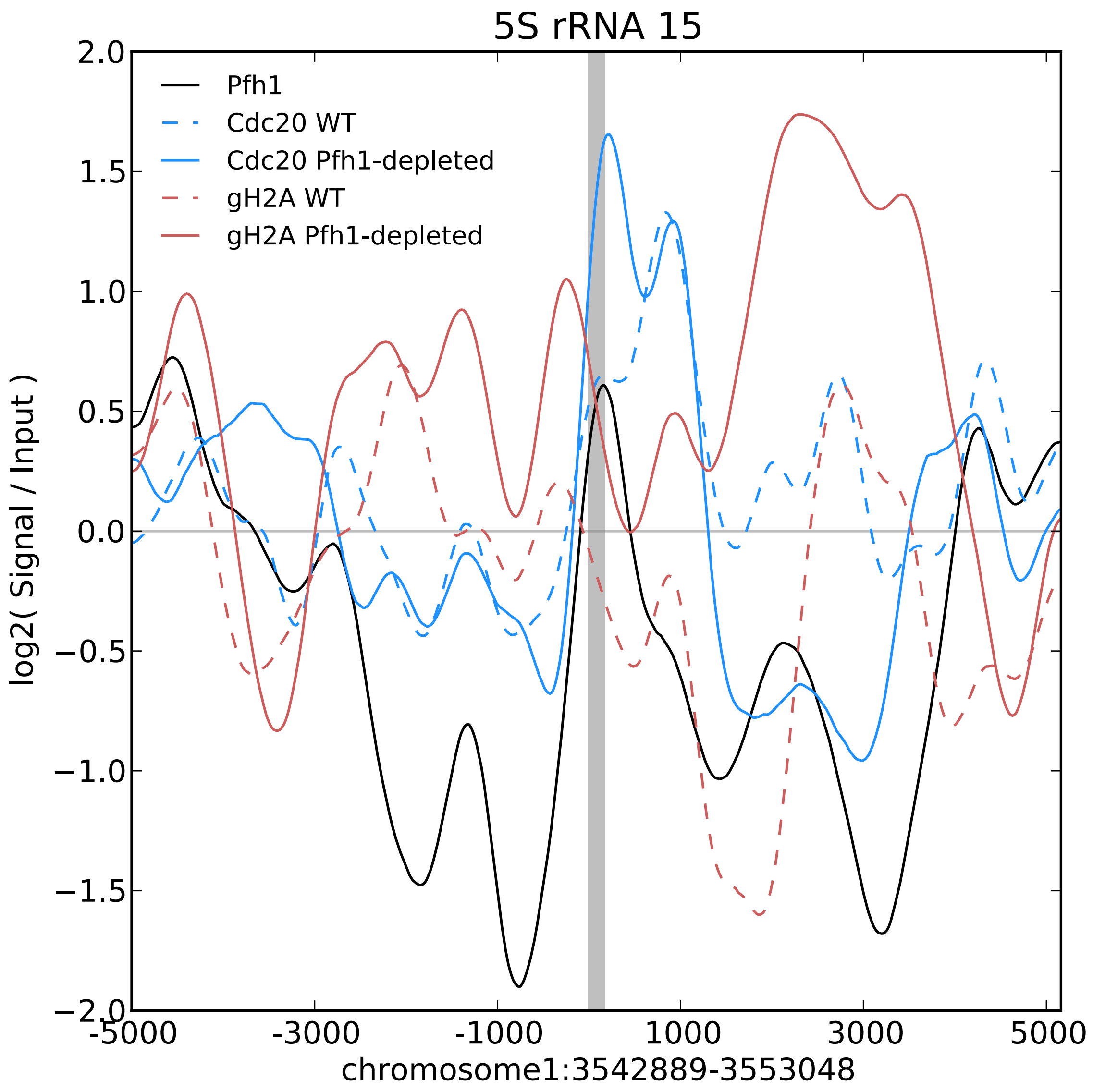
5S rRNA 15
Pfh1
Cdc20 WT
Cdc20 Pfh1-depleted
gH2A WT
gH2A Pfh1-depleted
log2(Signal / Input)
2.0
1.5
1.0
0.5
0.0
−0.5
−1.0
−1.5
−2.0
-5000
-3000
-1000
1000
3000
5000
chromosome1:3542889-3553048

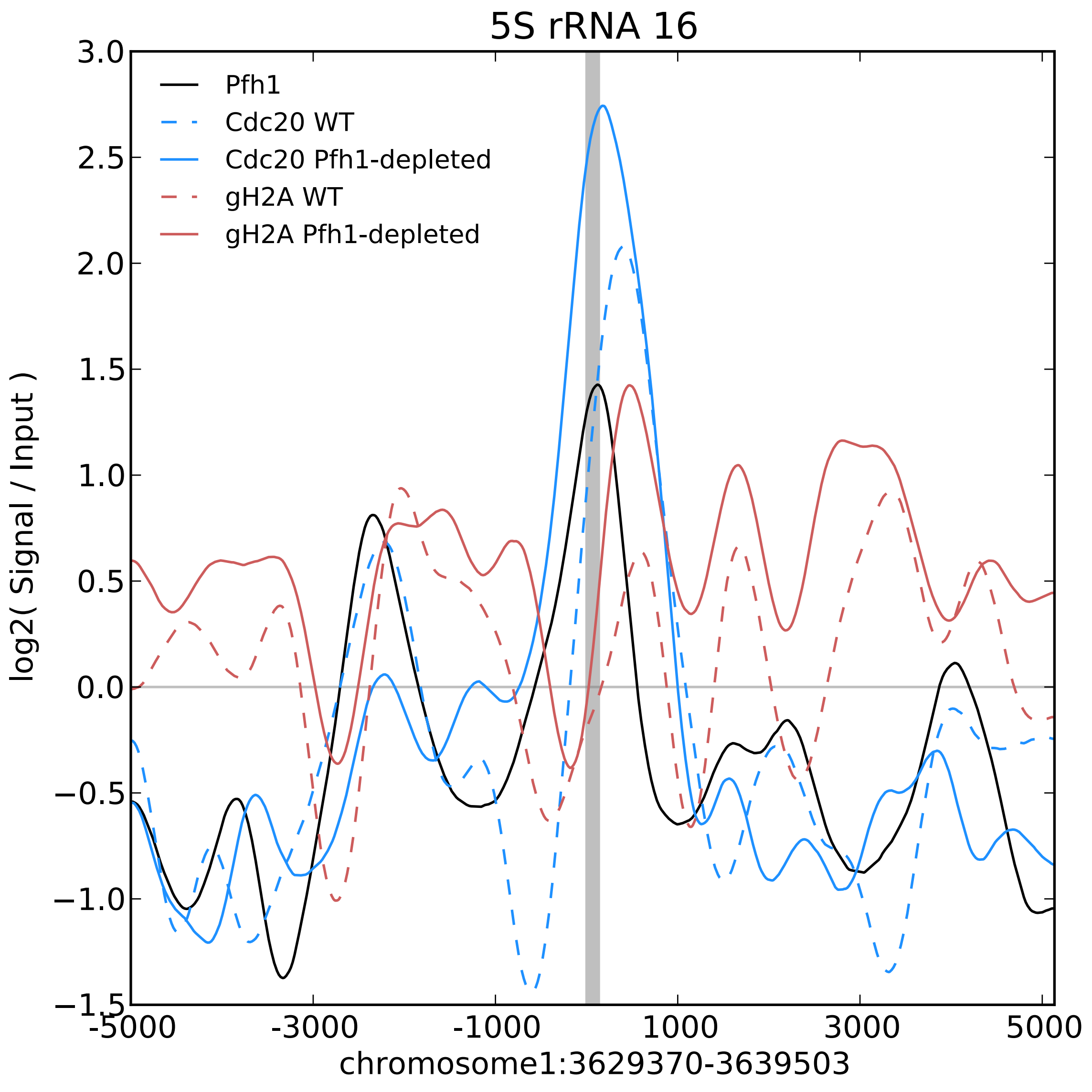
5S rRNA 16
Pfh1
Cdc20 WT
Cdc20 Pfh1-depleted
gH2A WT
gH2A Pfh1-depleted
log2(Signal / Input)
3.0
2.5
2.0
1.5
1.0
0.5
0.0
−0.5
−1.0
−1.5
-5000
-3000
-1000
1000
3000
5000
chromosome1:3629370-3639503

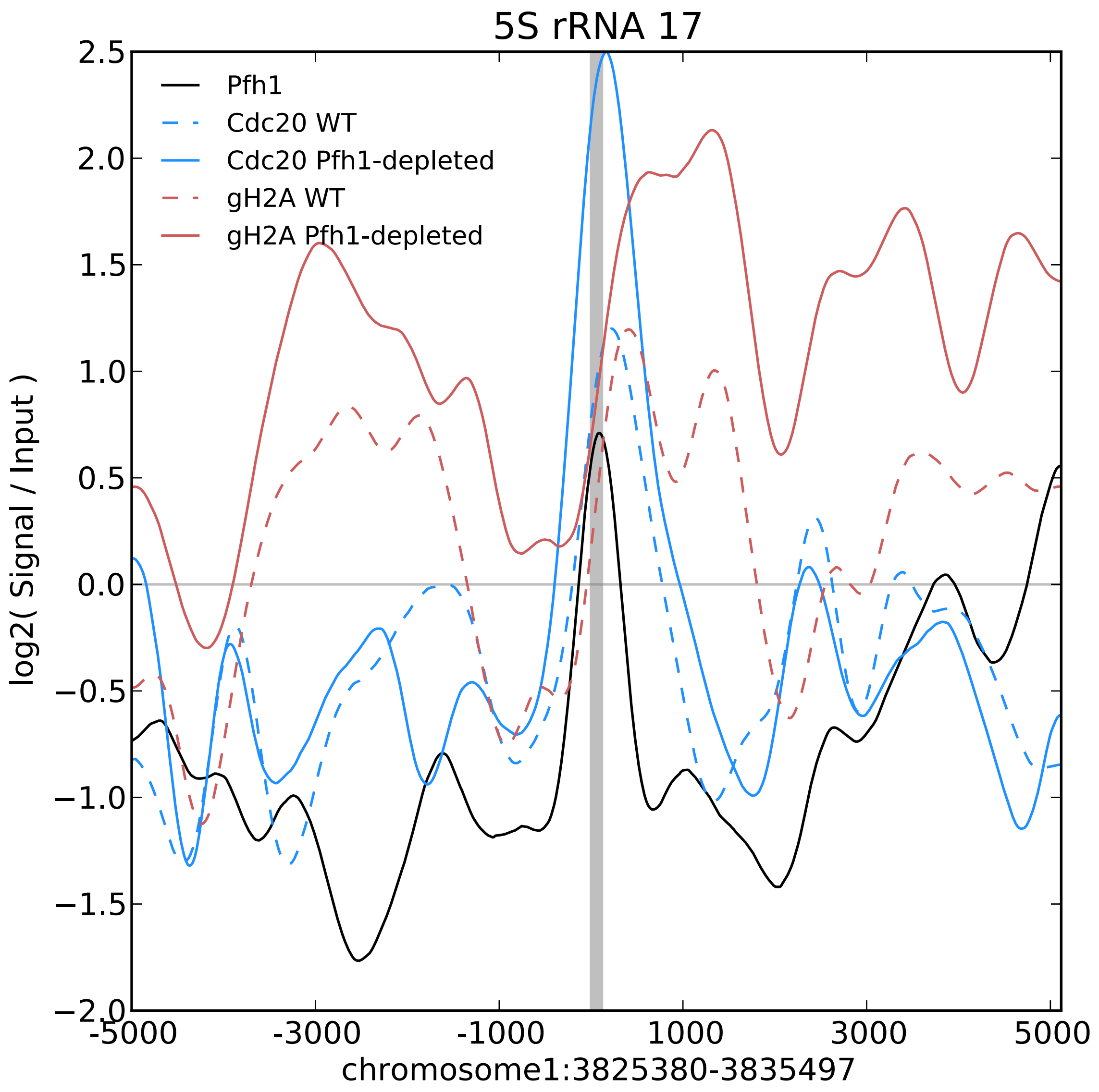

5S rRNA 17
Pfh1
Cdc20 WT
Cdc20 Pfh1-depleted
gH2A WT
gH2A Pfh1-depleted
2.5
2.0
1.5
1.0
0.5
0.0
−0.5
−1.0
−1.5
−2.0
log2(Signal / Input)
-5000
-3000
-1000
1000
3000
5000
chromosome1:3825380-3835497

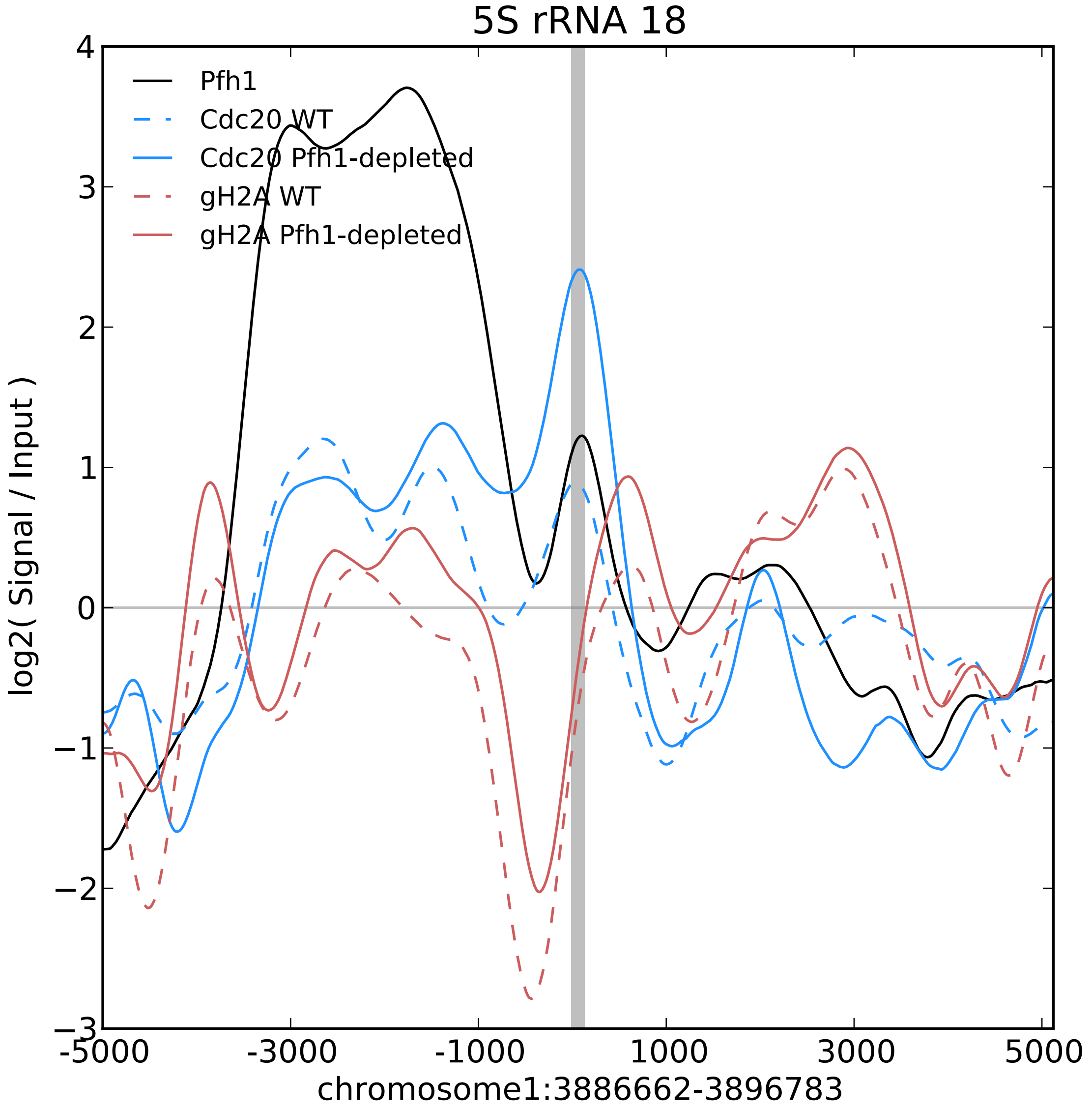
5S rRNA 18
Pfh1
Cdc20 WT
Cdc20 Pfh1-depleted
gH2A WT
gH2A Pfh1-depleted
log2(Signal / Input)
4
3
2
1
0
−1
−2
−3
-5000
-3000
-1000
1000
3000
5000
chromosome1:3886662-3896783

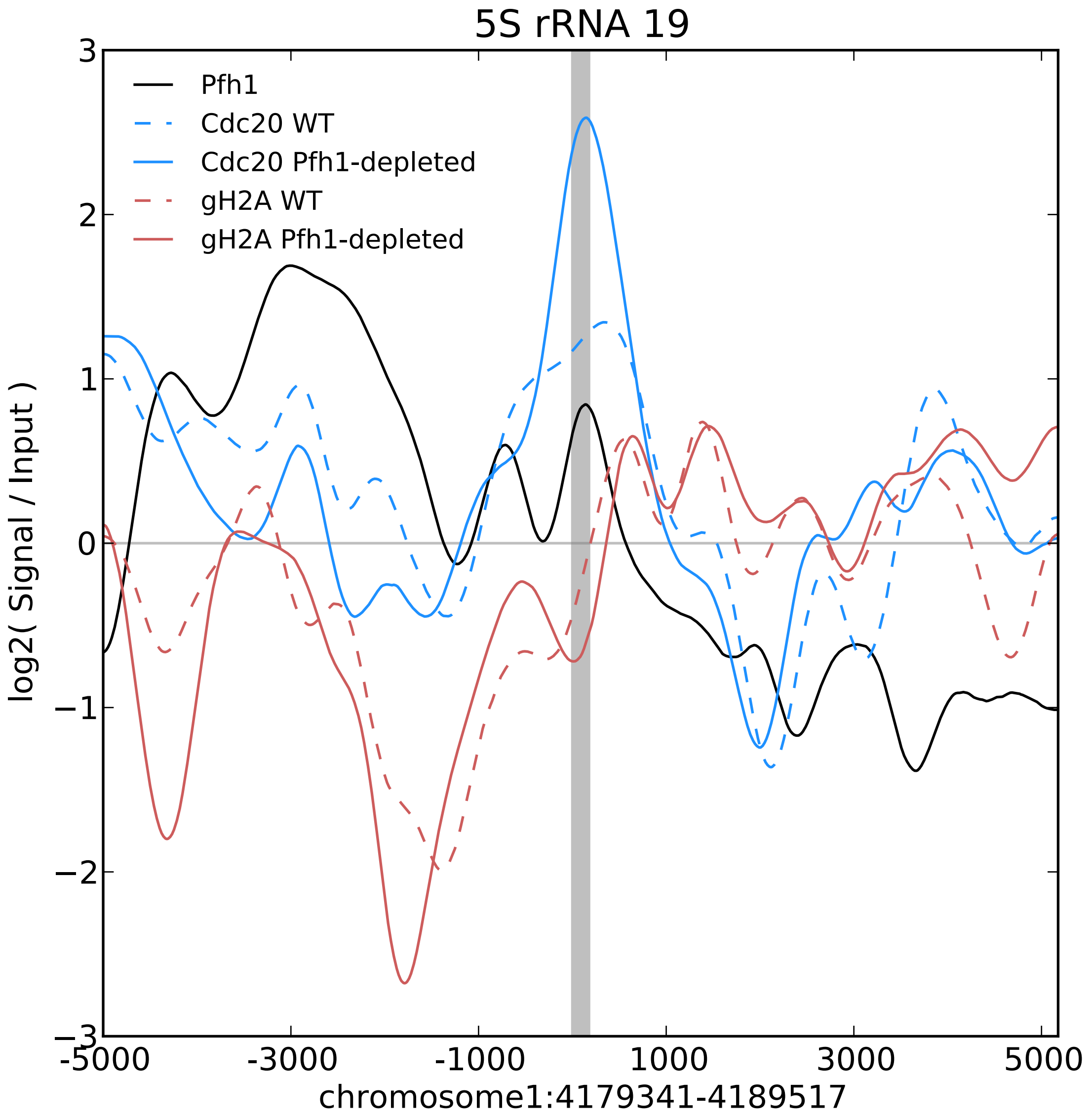

5S rRNA 19
Pfh1
Cdc20 WT
Cdc20 Pfh1-depleted
gH2A WT
gH2A Pfh1-depleted
log2(Signal / Input)
3
2
1
0
−1
−2
−3
-5000
-3000
-1000
1000
3000
5000
chromosome1:4179341-4189517

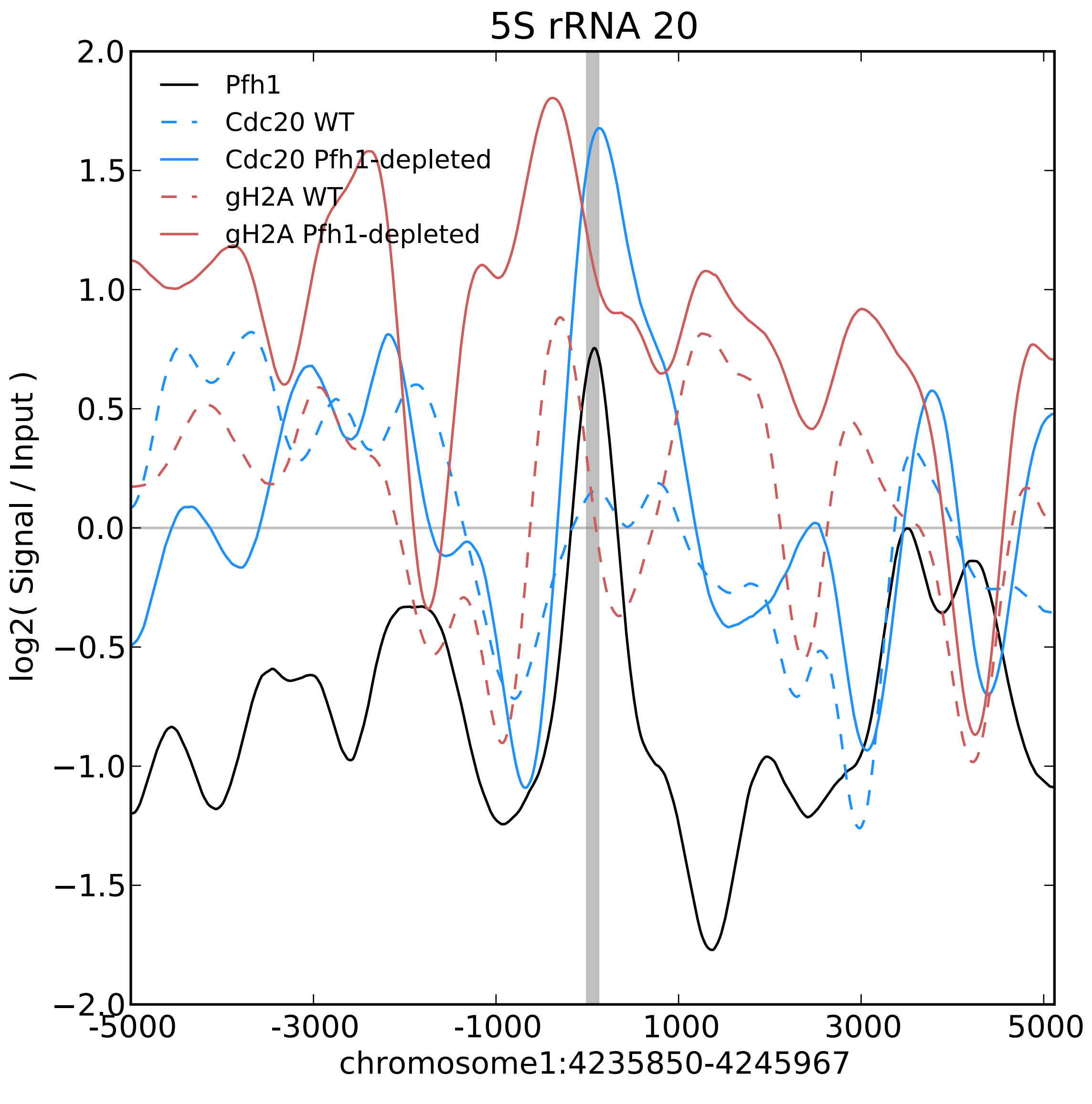
5S rRNA 20
Pfh1
Cdc20 WT
Cdc20 Pfh1-depleted
gH2A WT
gH2A Pfh1-depleted
2.0
1.5
1.0
0.5
0.0
−0.5
−1.0
−1.5
−2.0
log2(Signal / Input)
-5000
-3000
-1000
1000
3000
5000
chromosome1:4235850-4245967

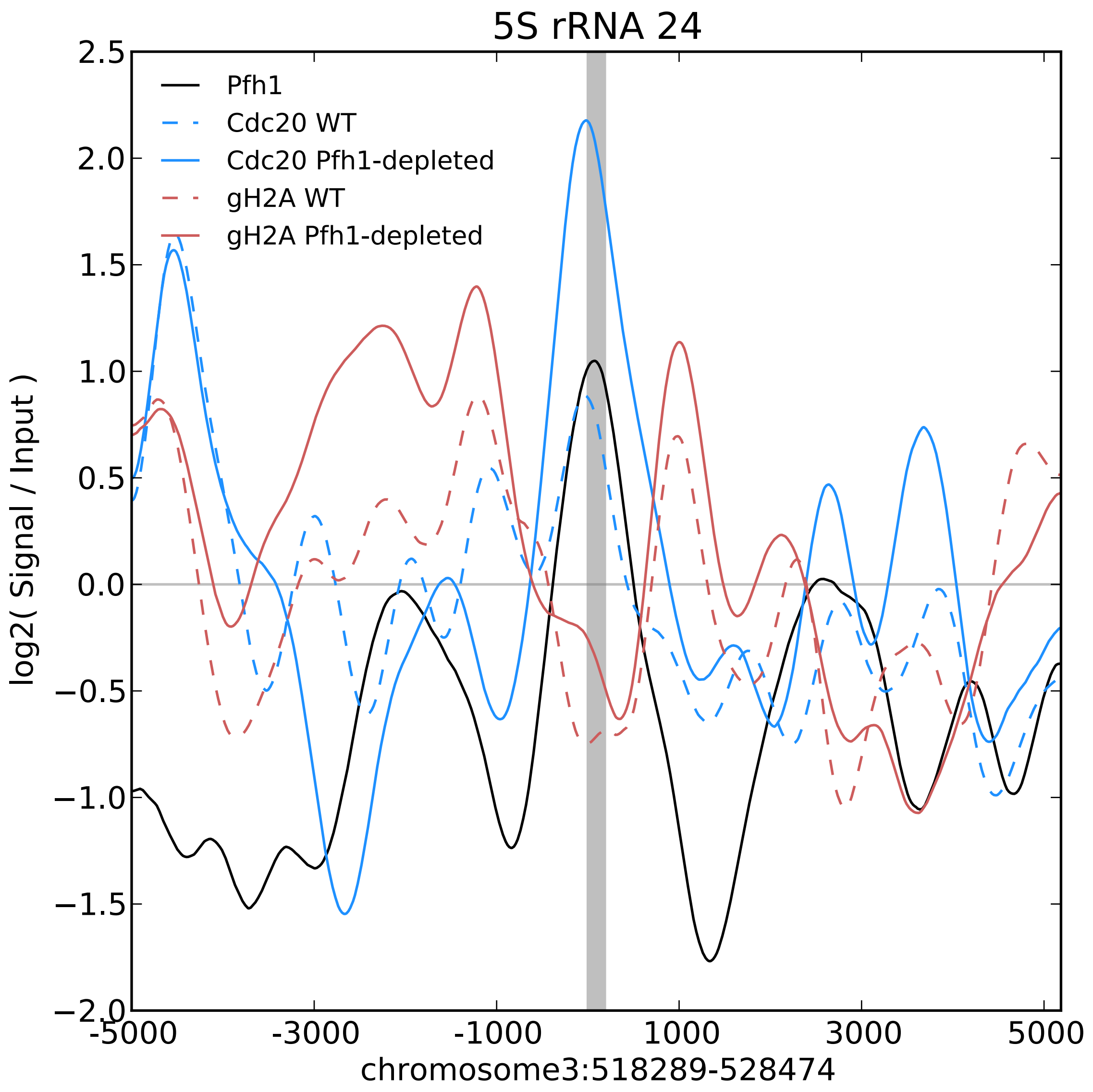

5S rRNA 24
Pfh1
Cdc20 WT
Cdc20 Pfh1-depleted
gH2A WT
gH2A Pfh1-depleted
2.5
2.0
1.5
1.0
0.5
0.0
−0.5
−1.0
−1.5
−2.0
log2(Signal / Input)
-5000
-3000
-1000
1000
3000
5000
chromosome3:518289-528474

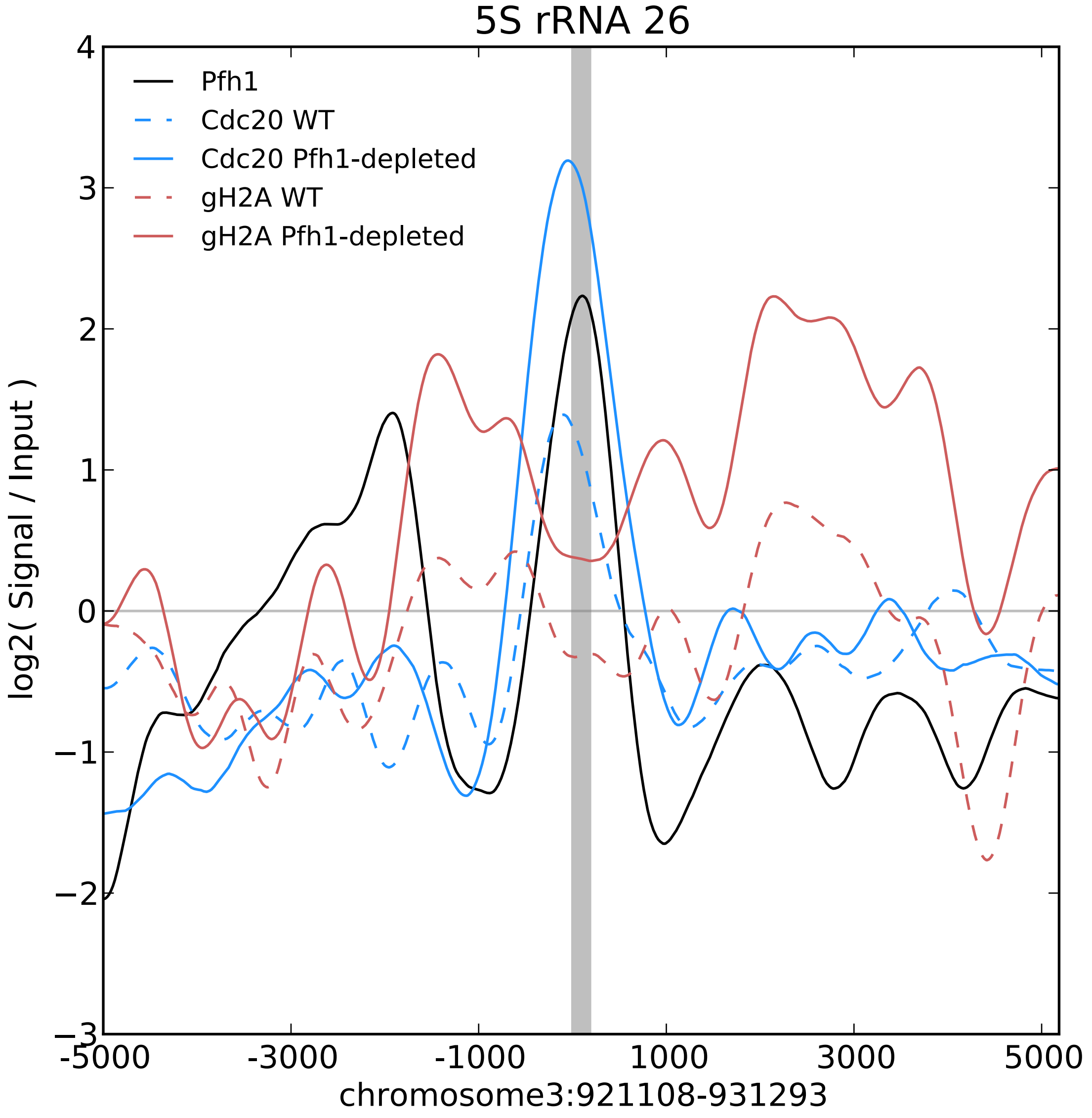

5S rRNA 26
Pfh1
Cdc20 WT
Cdc20 Pfh1-depleted
gH2A WT
gH2A Pfh1-depleted
log2(Signal / Input)
4
3
2
1
0
−1
−2
−3
-5000
-3000
-1000
1000
3000
5000
chromosome3:921108-931293

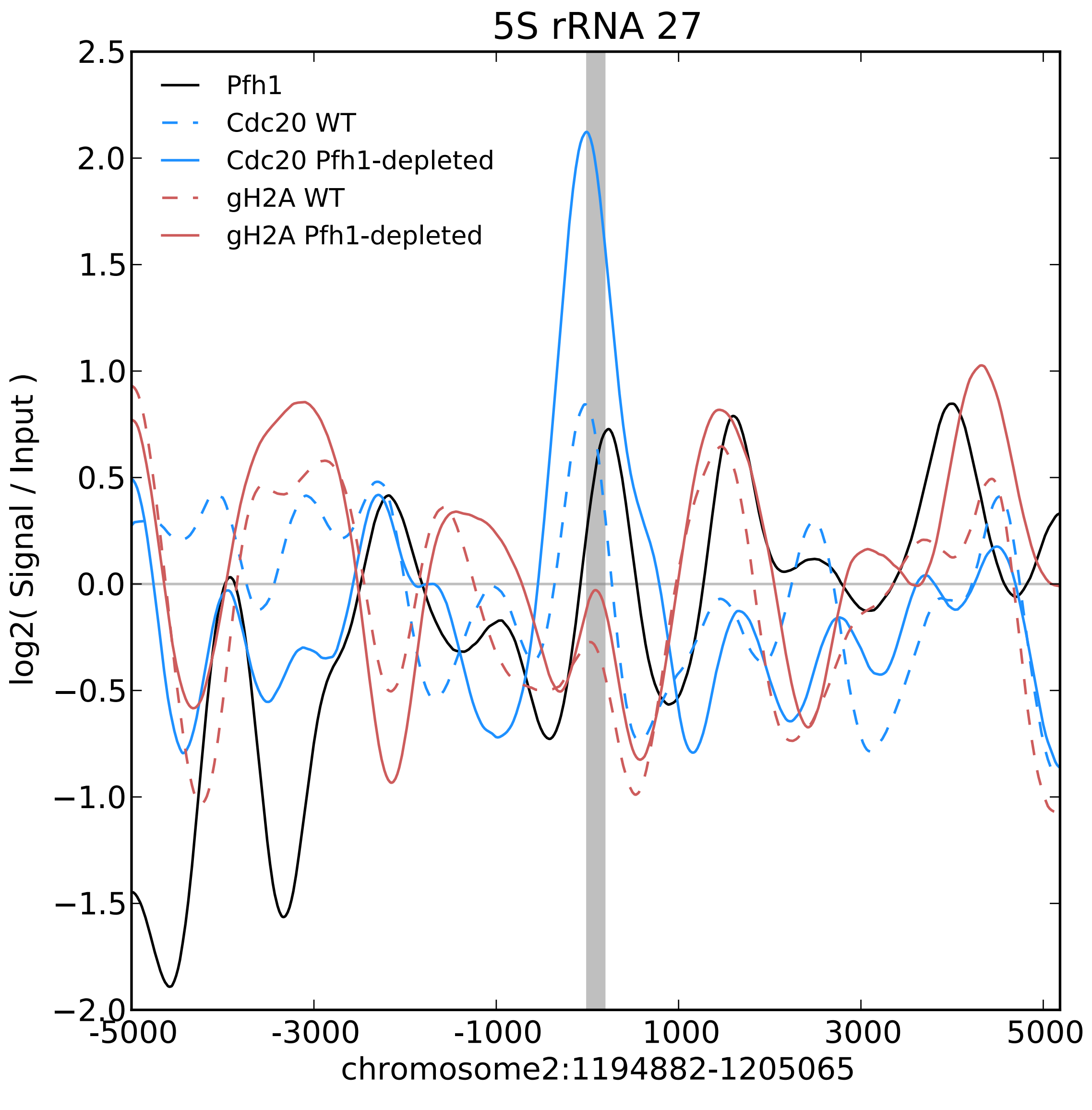

5S rRNA 27
Pfh1
Cdc20 WT
Cdc20 Pfh1-depleted
gH2A WT
gH2A Pfh1-depleted
log2(Signal / Input)
2.5
2.0
1.5
1.0
0.5
0.0
−0.5
−1.0
−1.5
−2.0
-5000
-3000
-1000
1000
3000
5000
chromosome2:1194882-1205065

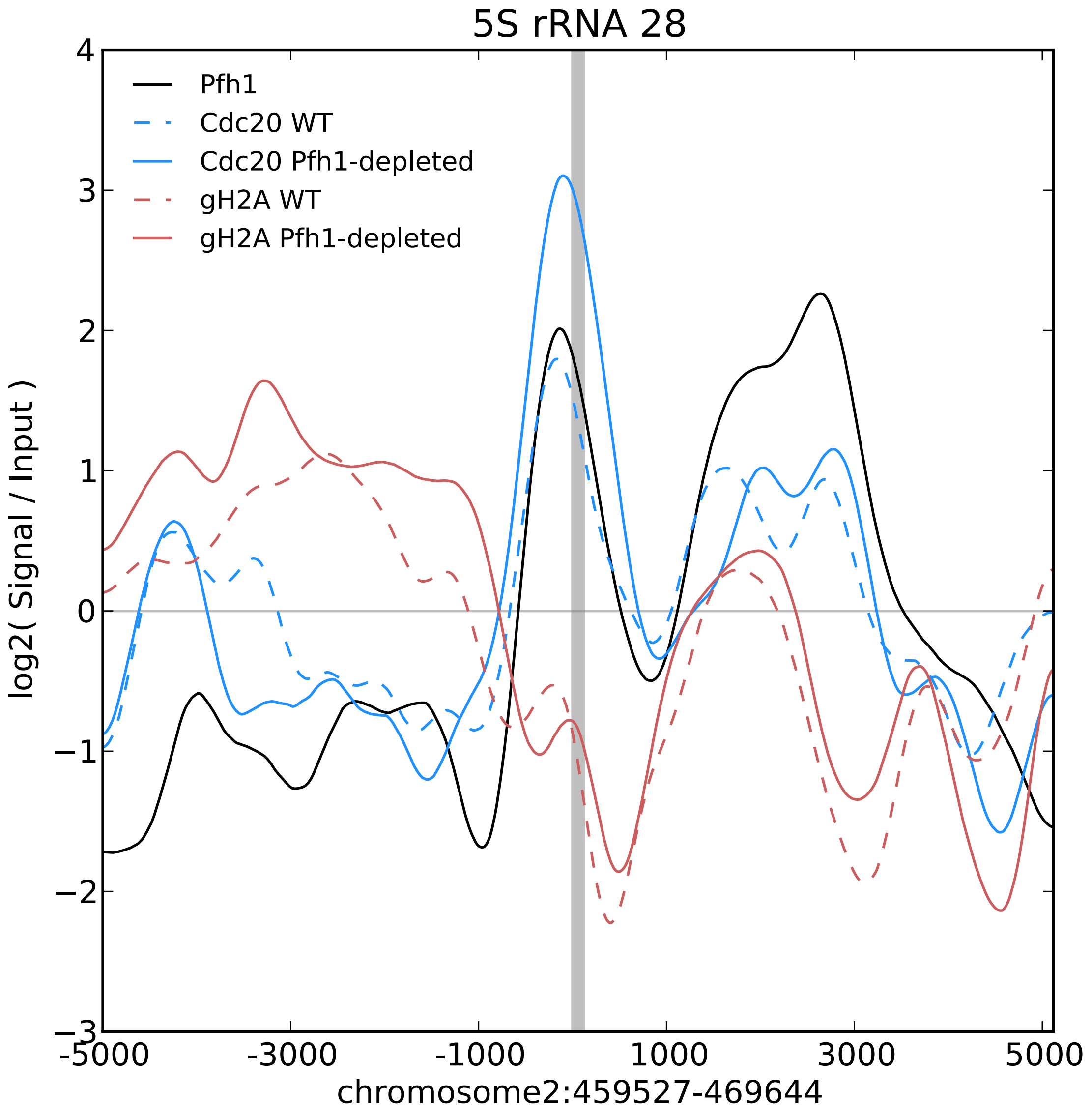
5S rRNA 28
Pfh1
Cdc20 WT
Cdc20 Pfh1-depleted
gH2A WT
gH2A Pfh1-depleted
log2(Signal / Input)
4
3
2
1
0
−1
−2
−3
-5000
-3000
-1000
1000
3000
5000
chromosome2:459527-469644

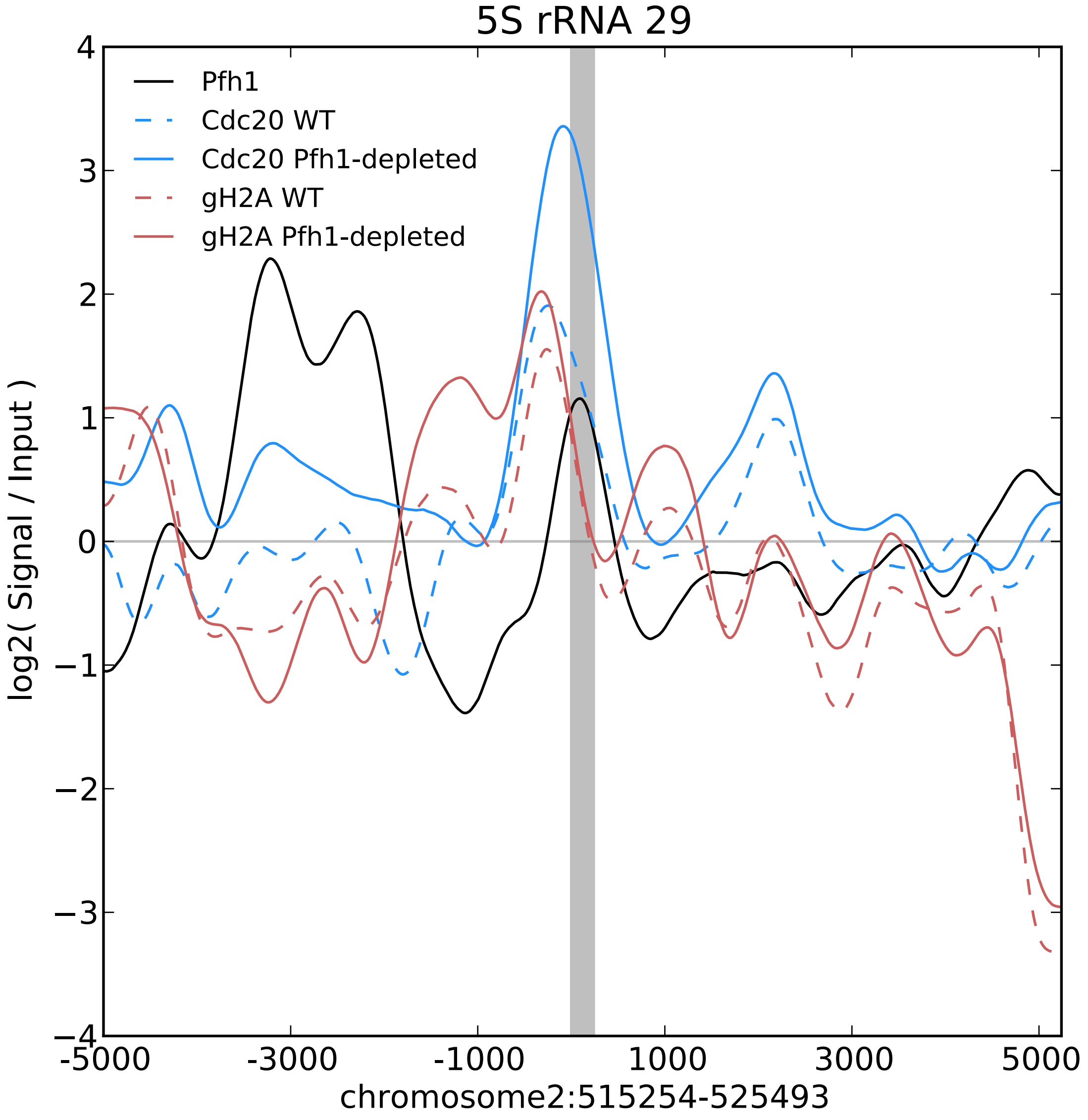

5S rRNA 29
Pfh1
Cdc20 WT
Cdc20 Pfh1-depleted
gH2A WT
gH2A Pfh1-depleted
log2(Signal / Input)
4
3
2
1
0
−1
−2
−3
−4
-5000
-3000
-1000
1000
3000
5000
chromosome2:515254-525493

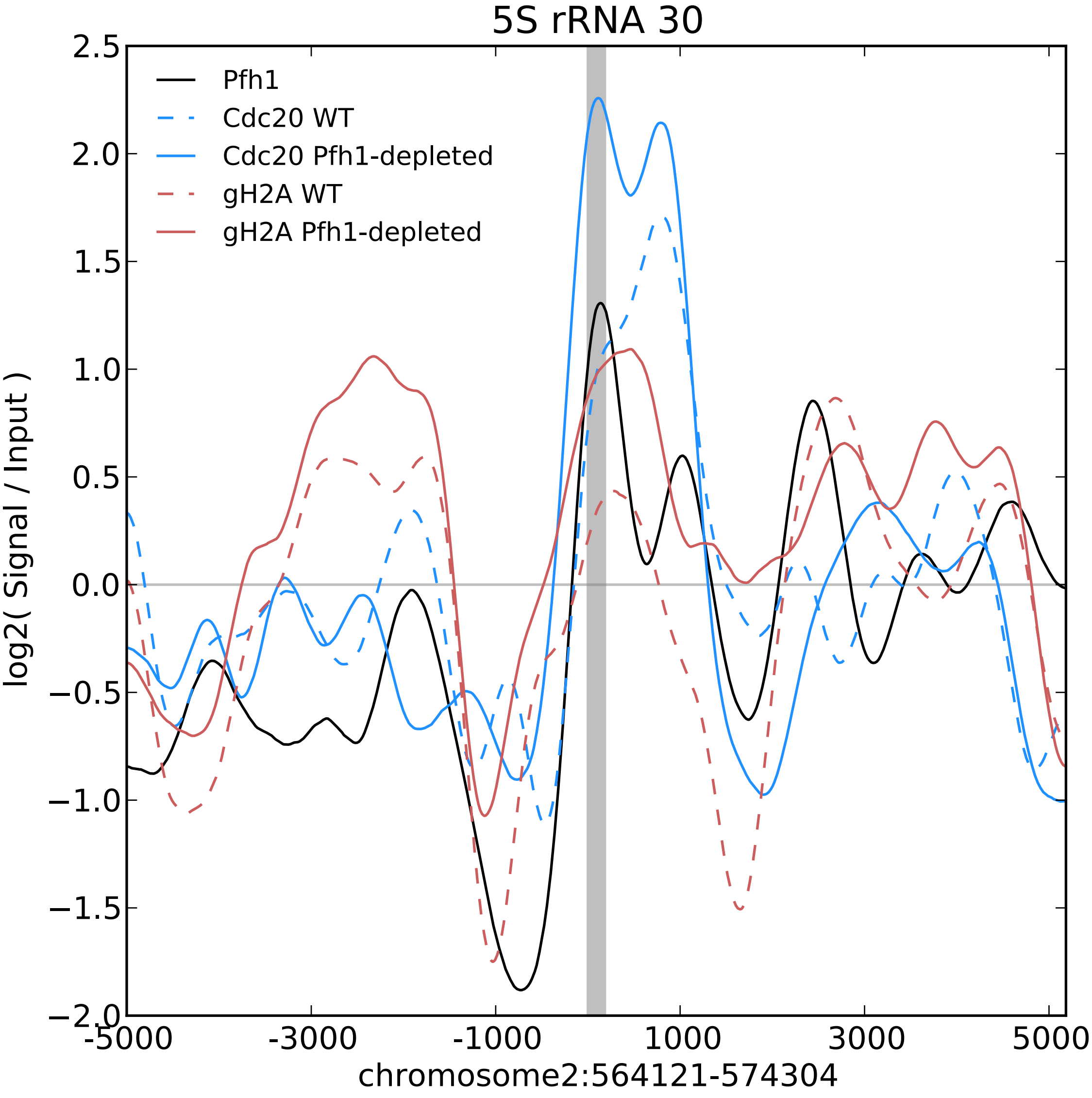

5S rRNA 30
Pfh1
Cdc20 WT
Cdc20 Pfh1-depleted
gH2A WT
gH2A Pfh1-depleted
log2(Signal / Input)
2.5
2.0
1.5
1.0
0.5
0.0
−0.5
−1.0
−1.5
−2.0
-5000
-3000
-1000
1000
3000
5000
chromosome2:564121-574304

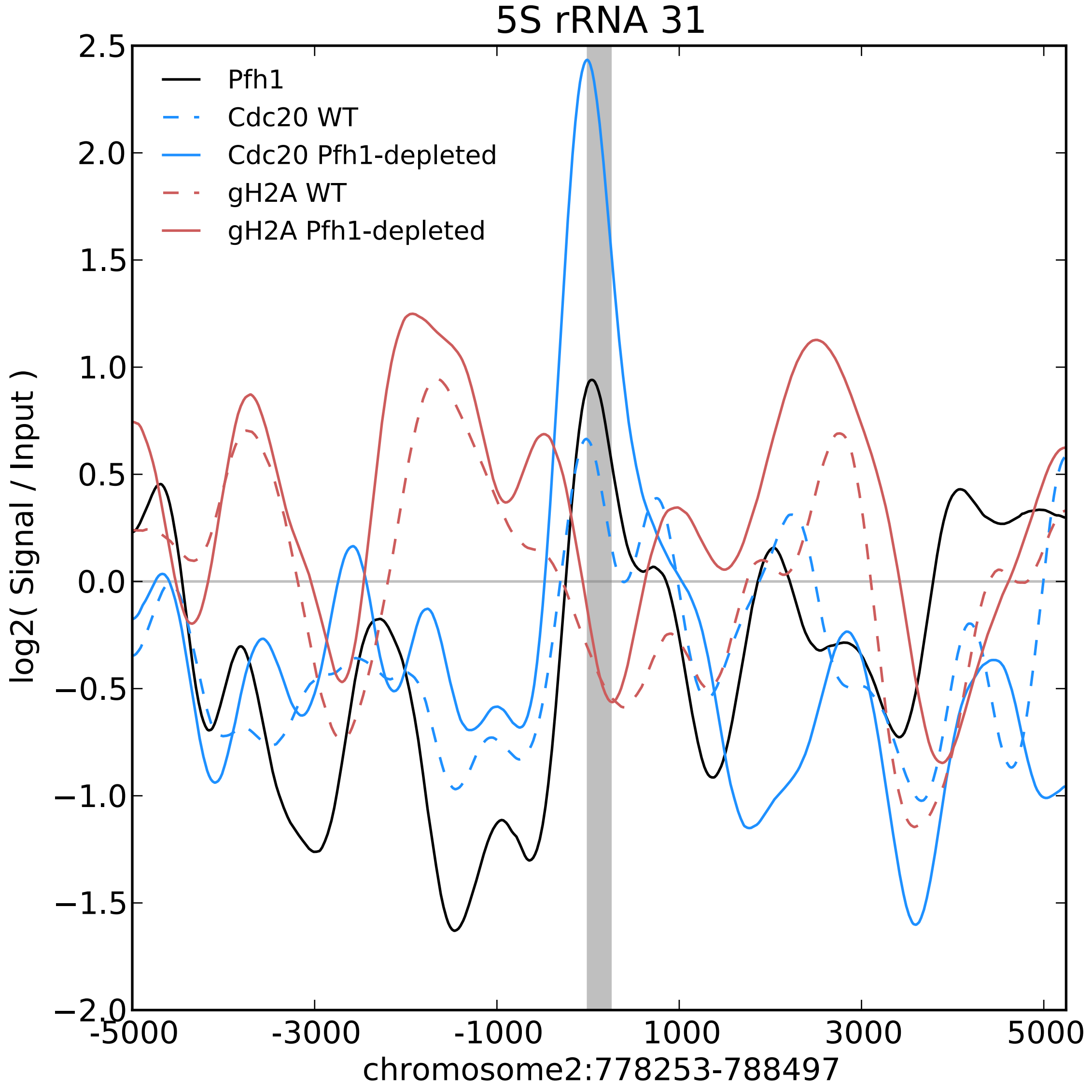
5S rRNA 31
Pfh1
Cdc20 WT
Cdc20 Pfh1-depleted
gH2A WT
gH2A Pfh1-depleted
log2(Signal / Input)
2.5
2.0
1.5
1.0
0.5
0.0
−0.5
−1.0
−1.5
−2.0
-5000
-3000
-1000
1000
3000
5000
chromosome2:778253-788497

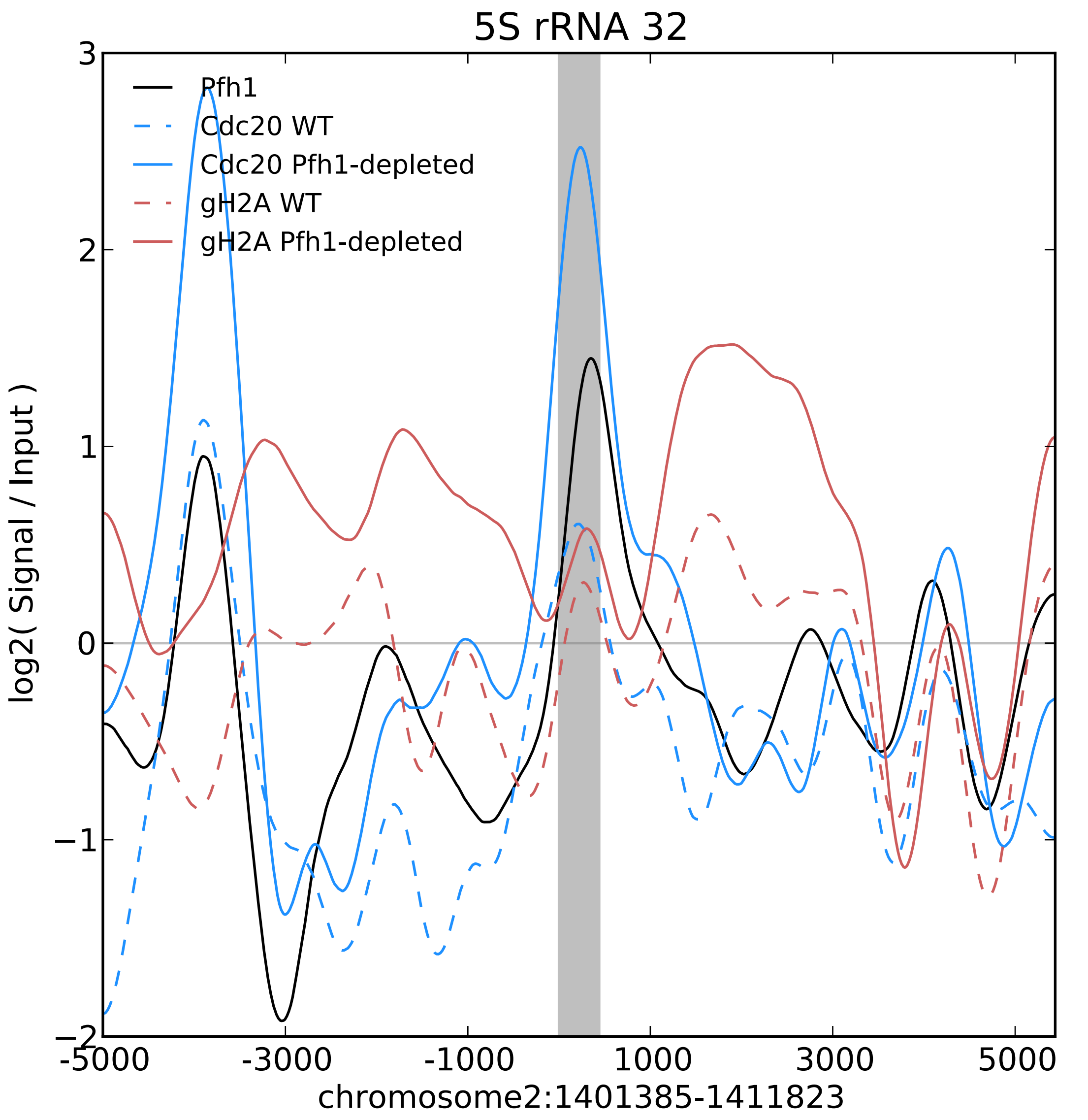
5S rRNA 32
Pfh1
Cdc20 WT
Cdc20 Pfh1-depleted
gH2A WT
gH2A Pfh1-depleted
log2(Signal / Input)
3
2
1
0
−1
−2
-5000
-3000
-1000
1000
3000
5000
chromosome2:1401385-1411823

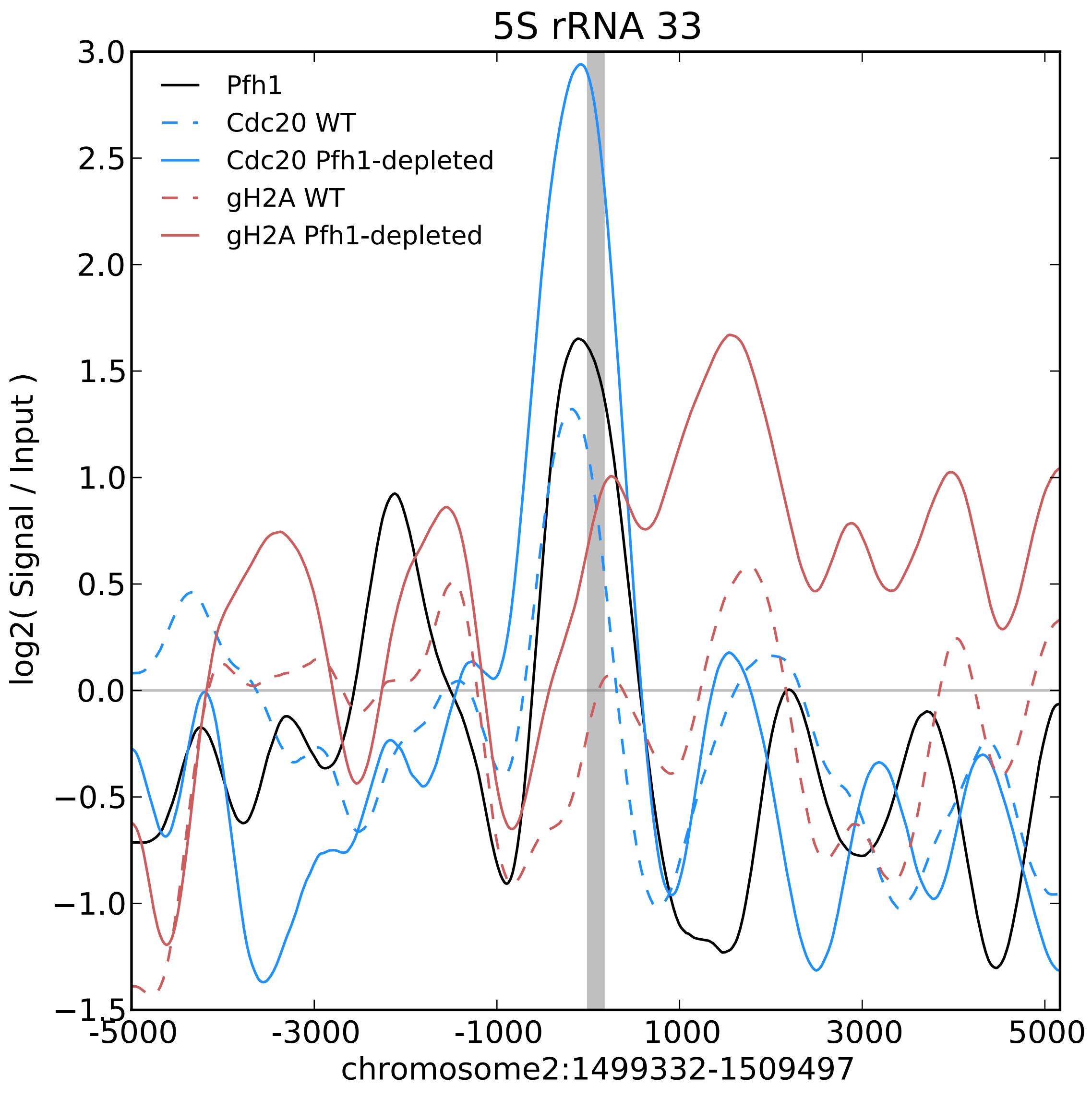
5S rRNA 33
Pfh1
Cdc20 WT
Cdc20 Pfh1-depleted
gH2A WT
gH2A Pfh1-depleted
log2(Signal / Input)
3.0
2.5
2.0
1.5
1.0
0.5
0.0
−0.5
−1.0
−1.5
-5000
-3000
-1000
1000
3000
5000
chromosome2:1499332-1509497

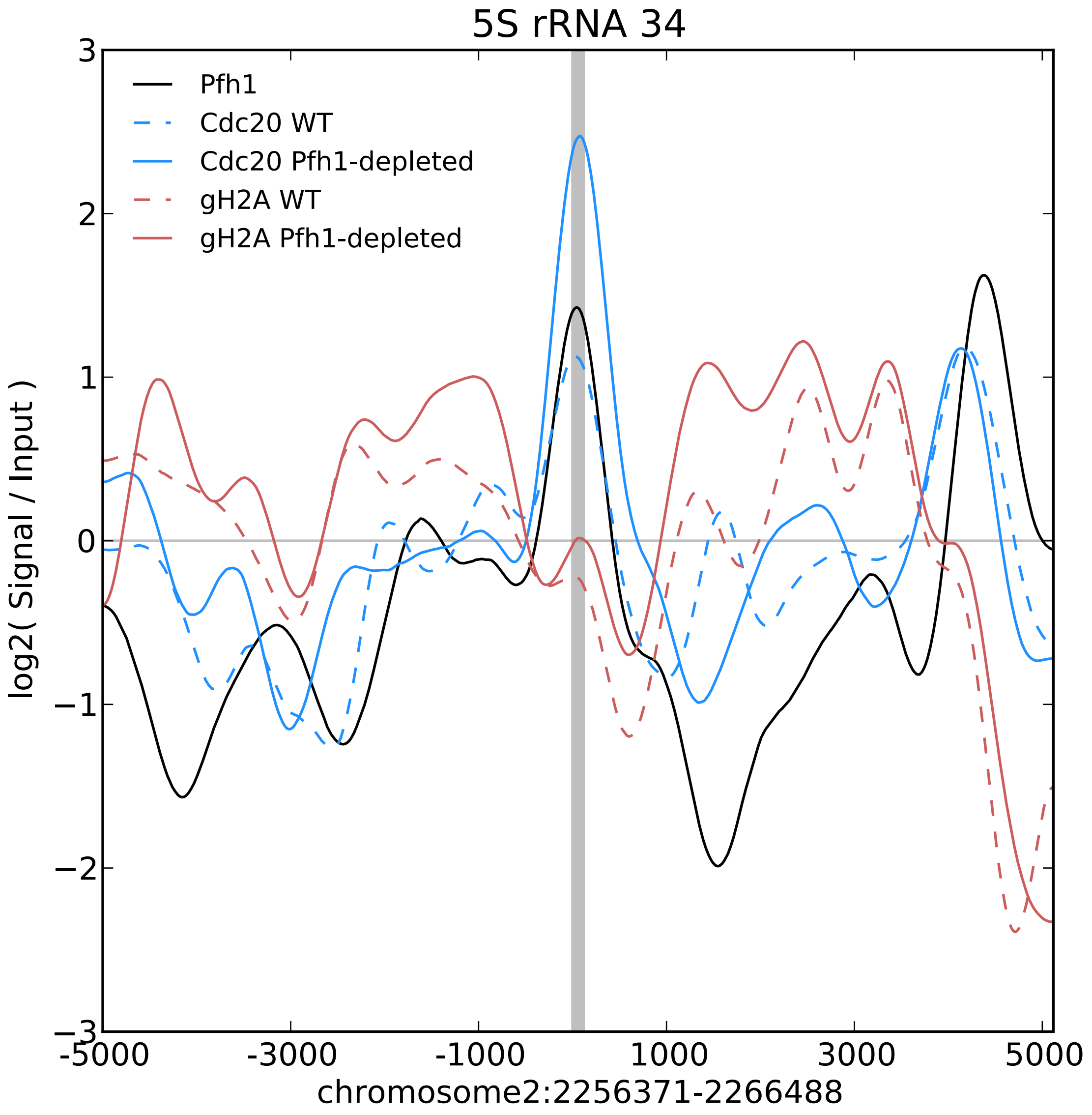
5S rRNA 34
Pfh1
Cdc20 WT
Cdc20 Pfh1-depleted
gH2A WT
gH2A Pfh1-depleted
log2(Signal / Input)
3
2
1
0
−1
−2
−3
-5000
-3000
-1000
1000
3000
5000
chromosome2:2256371-2266488

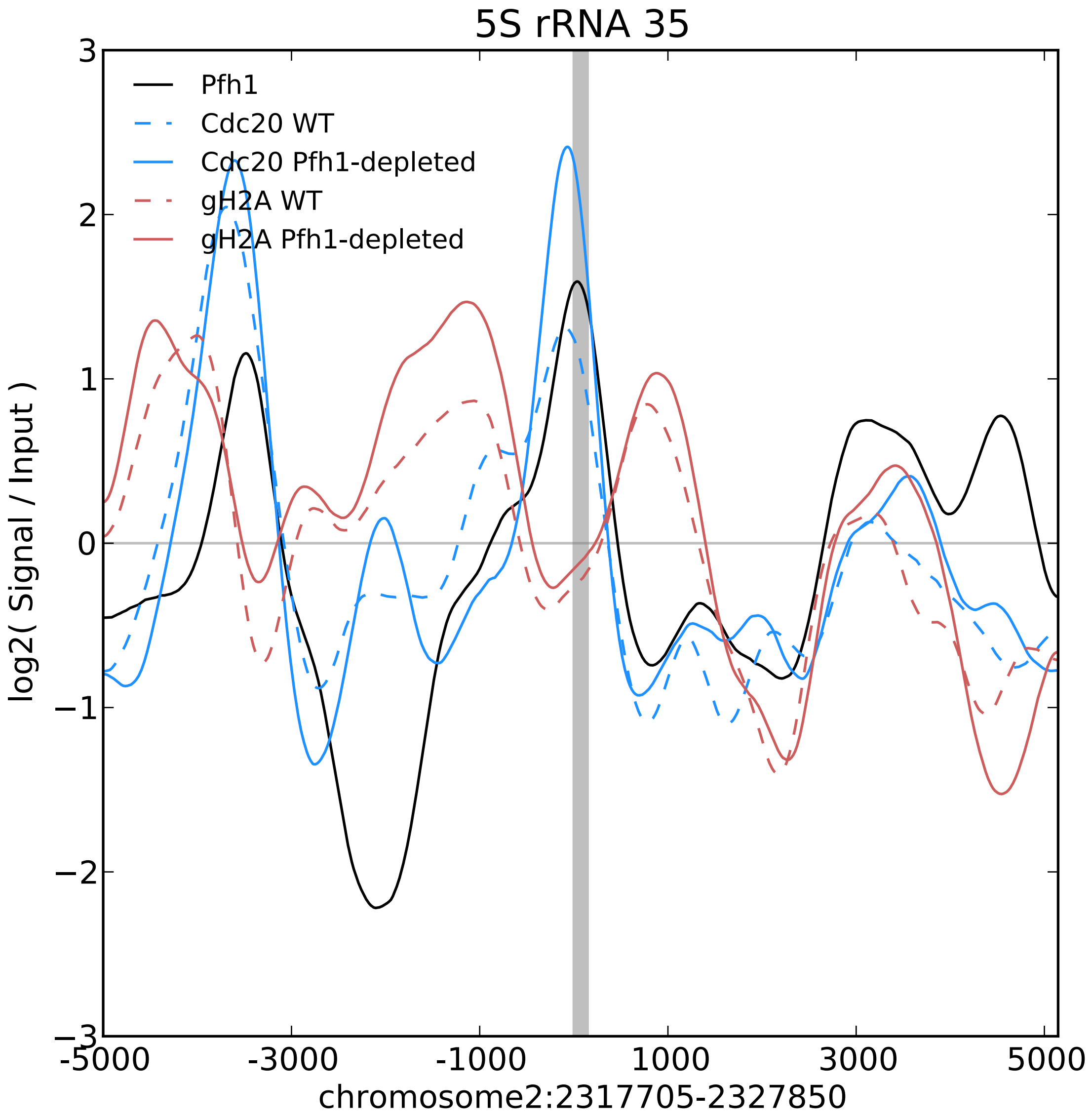

5S rRNA 35
Pfh1
Cdc20 WT
Cdc20 Pfh1-depleted
gH2A WT
gH2A Pfh1-depleted
log2(Signal / Input)
3
2
1
0
−1
−2
−3
-5000
-3000
-1000
1000
3000
5000
chromosome2:2317705-2327850

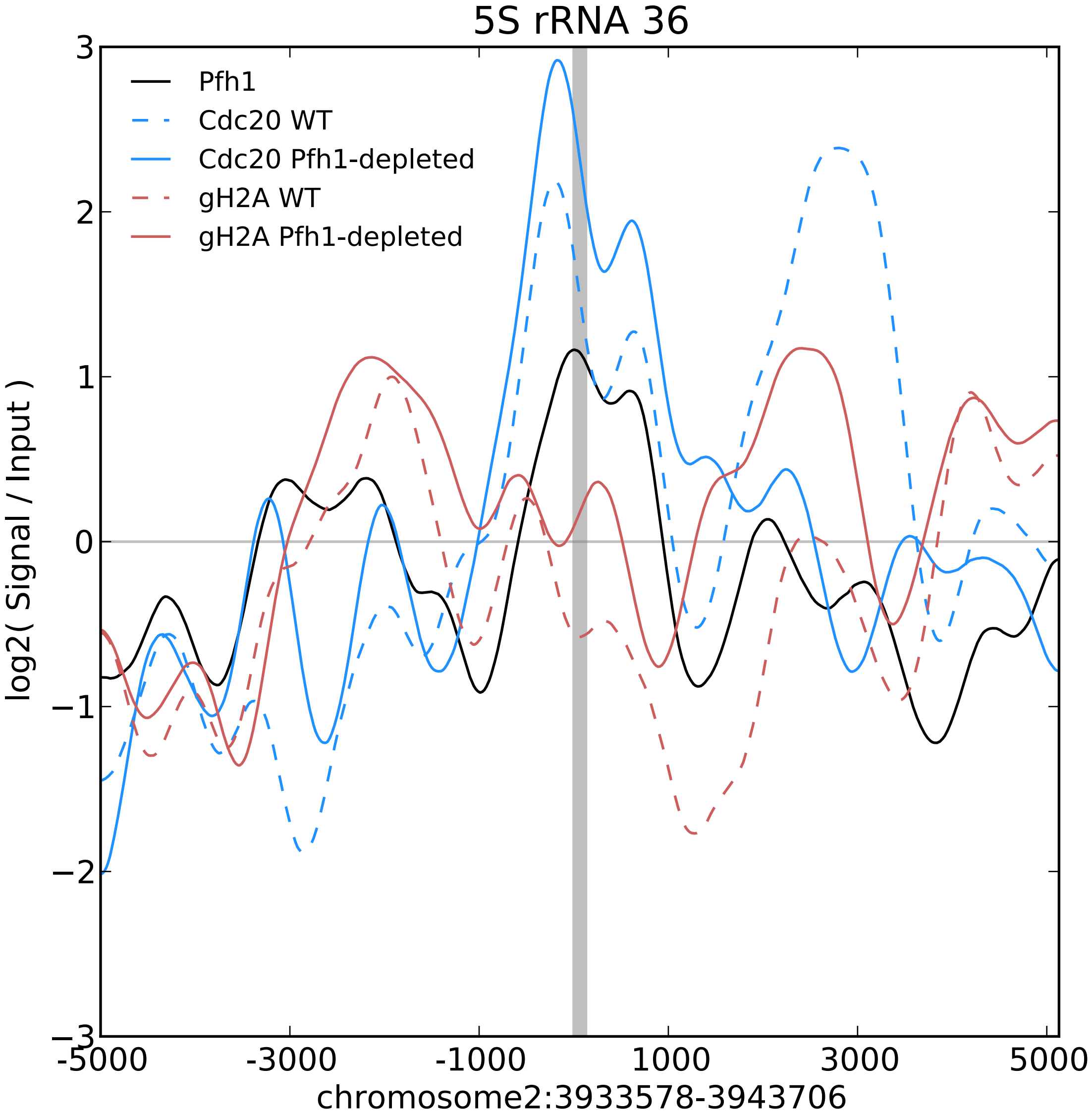
5S rRNA 36
Pfh1
Cdc20 WT
Cdc20 Pfh1-depleted
gH2A WT
gH2A Pfh1-depleted
log2(Signal / Input)
3
2
1
0
−1
−2
−3
-5000
-3000
-1000
1000
3000
5000
chromosome2:3933578-3943706

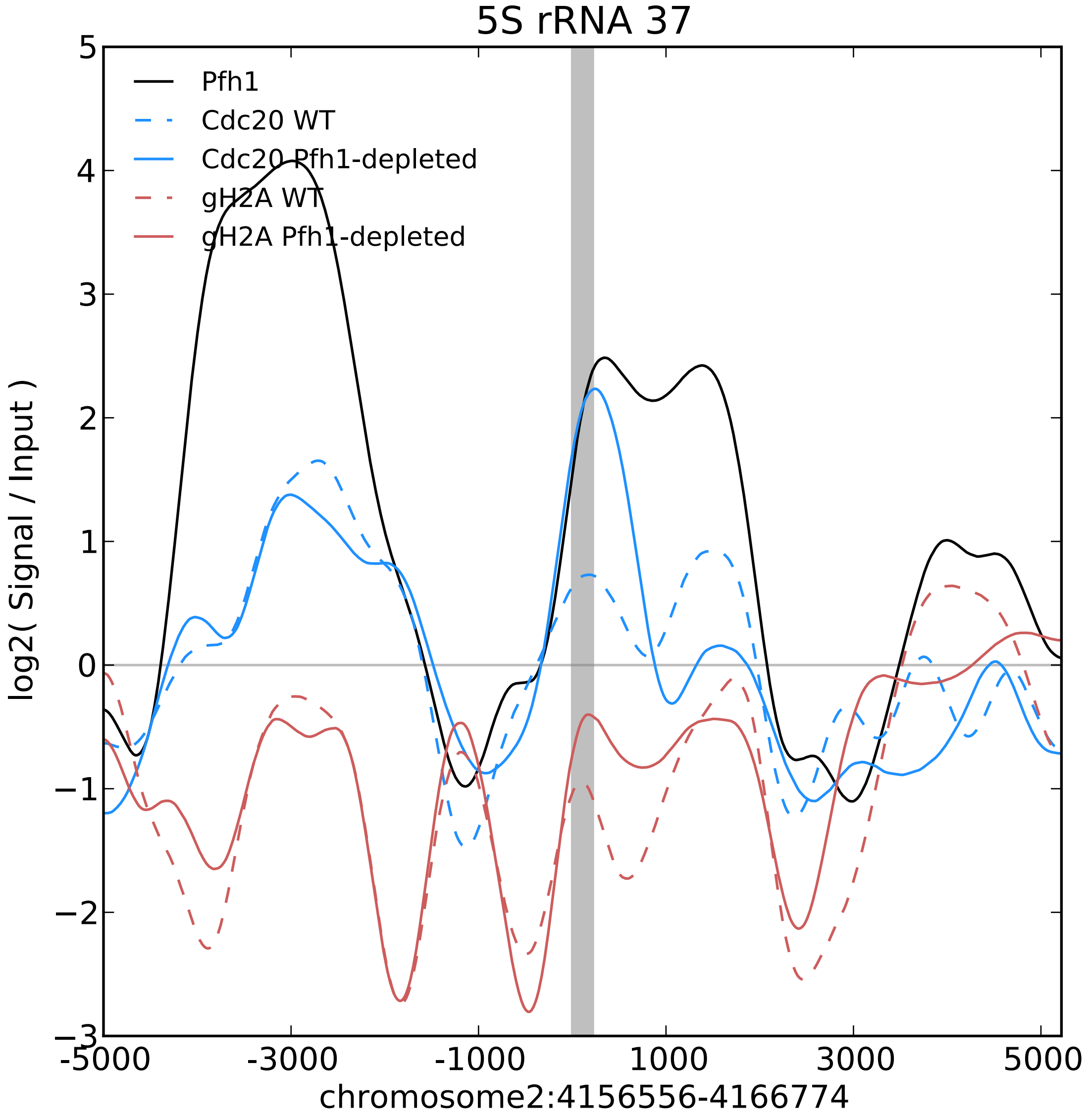

5S rRNA 37
Pfh1
Cdc20 WT
Cdc20 Pfh1-depleted
gH2A WT
gH2A Pfh1-depleted
log2(Signal / Input)
5
4
3
2
1
0
−1
−2
−3
-5000
-3000
-1000
1000
3000
5000
chromosome2:4156556-4166774

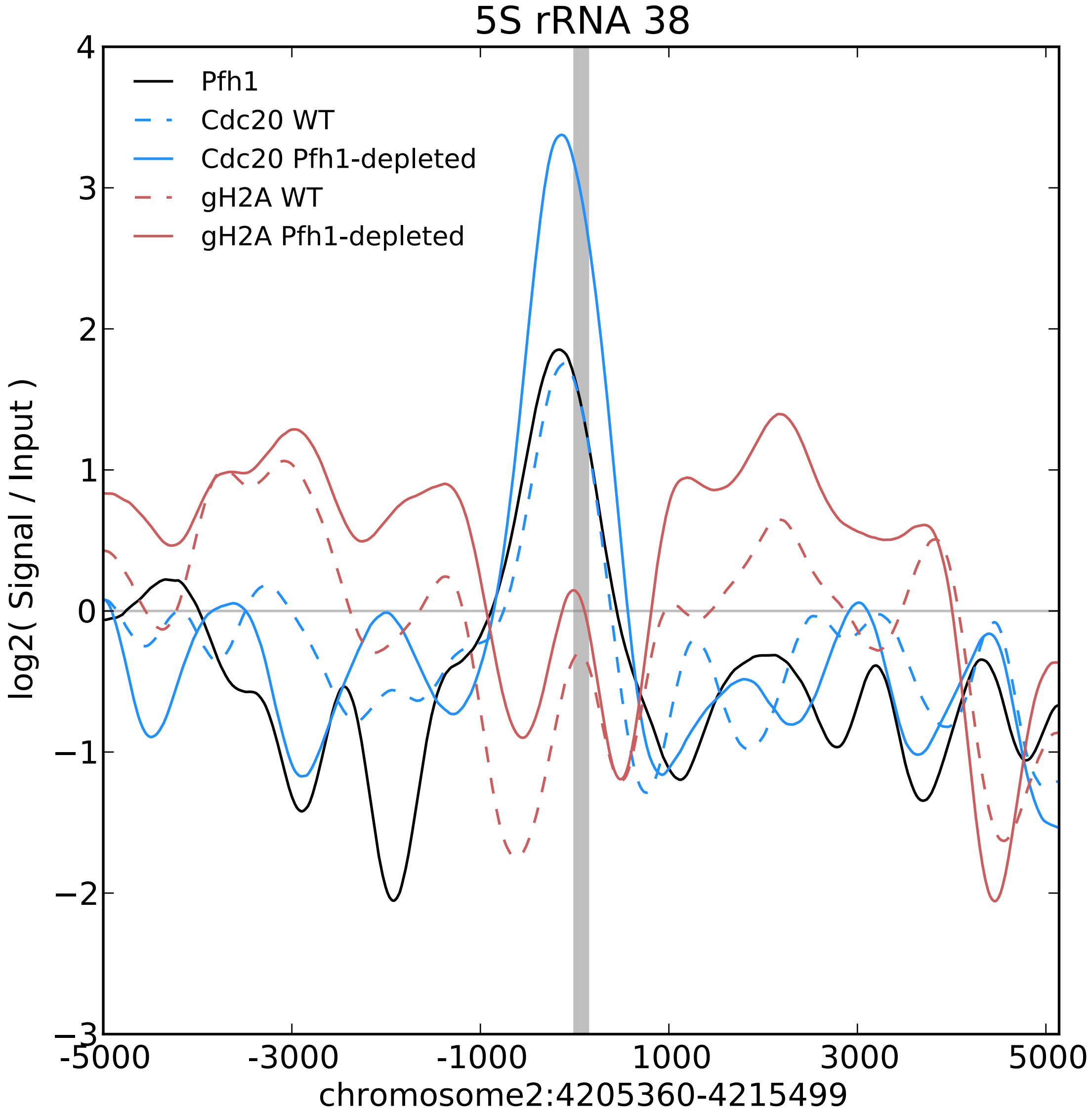
5S rRNA 38
Pfh1
Cdc20 WT
Cdc20 Pfh1-depleted
gH2A WT
gH2A Pfh1-depleted
log2(Signal / Input)
chromosome2:4205360-4215499
4
3
2
1
0
−1
−2
−3
-5000
-3000
-1000
1000
3000
5000

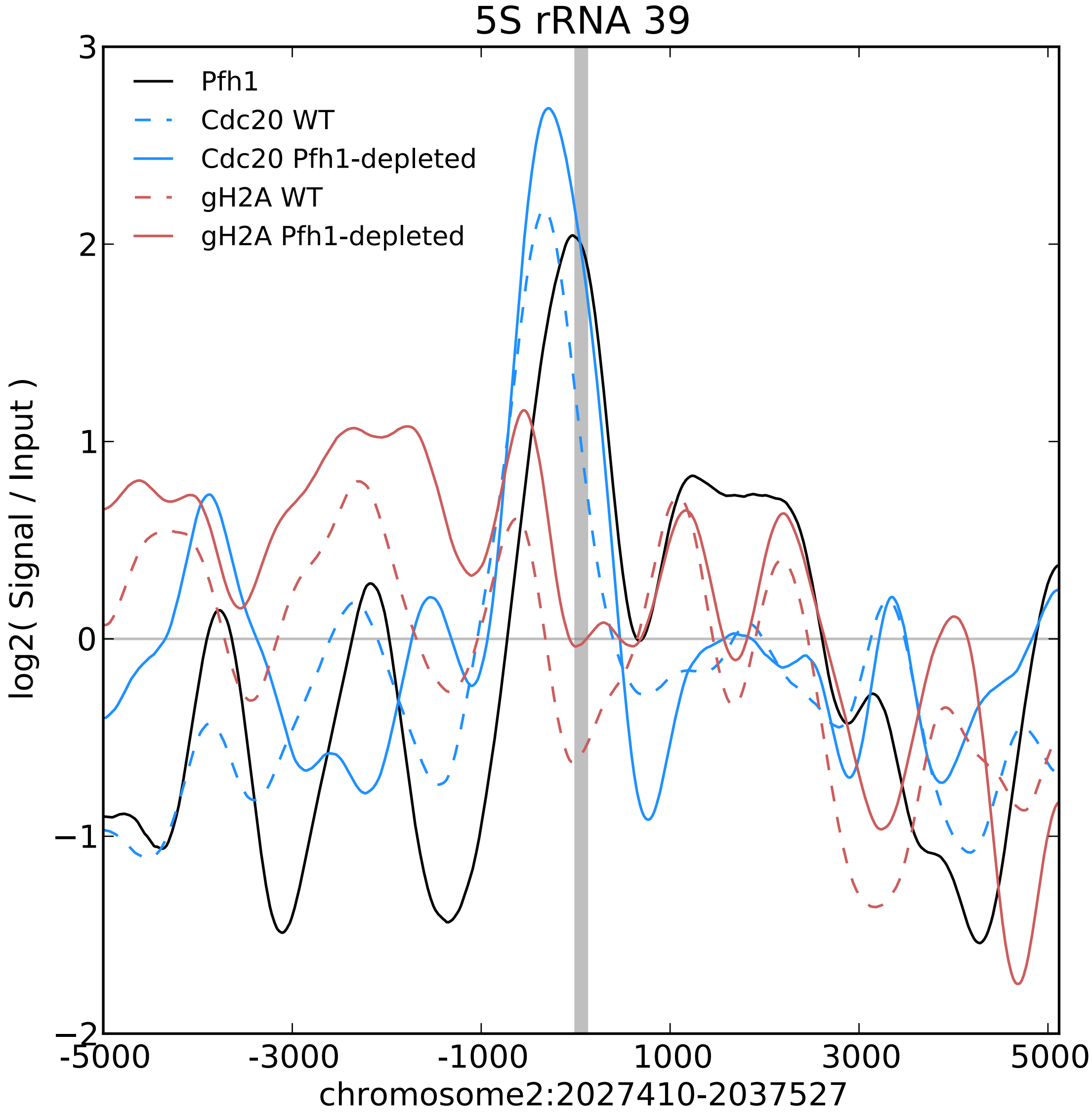

5S rRNA 39
Pfh1
Cdc20 WT
Cdc20 Pfh1-depleted
gH2A WT
gH2A Pfh1-depleted
log2(Signal / Input)
3
2
1
0
−1
−2
-5000
-3000
-1000
1000
3000
5000
chromosome2:2027410-2037527

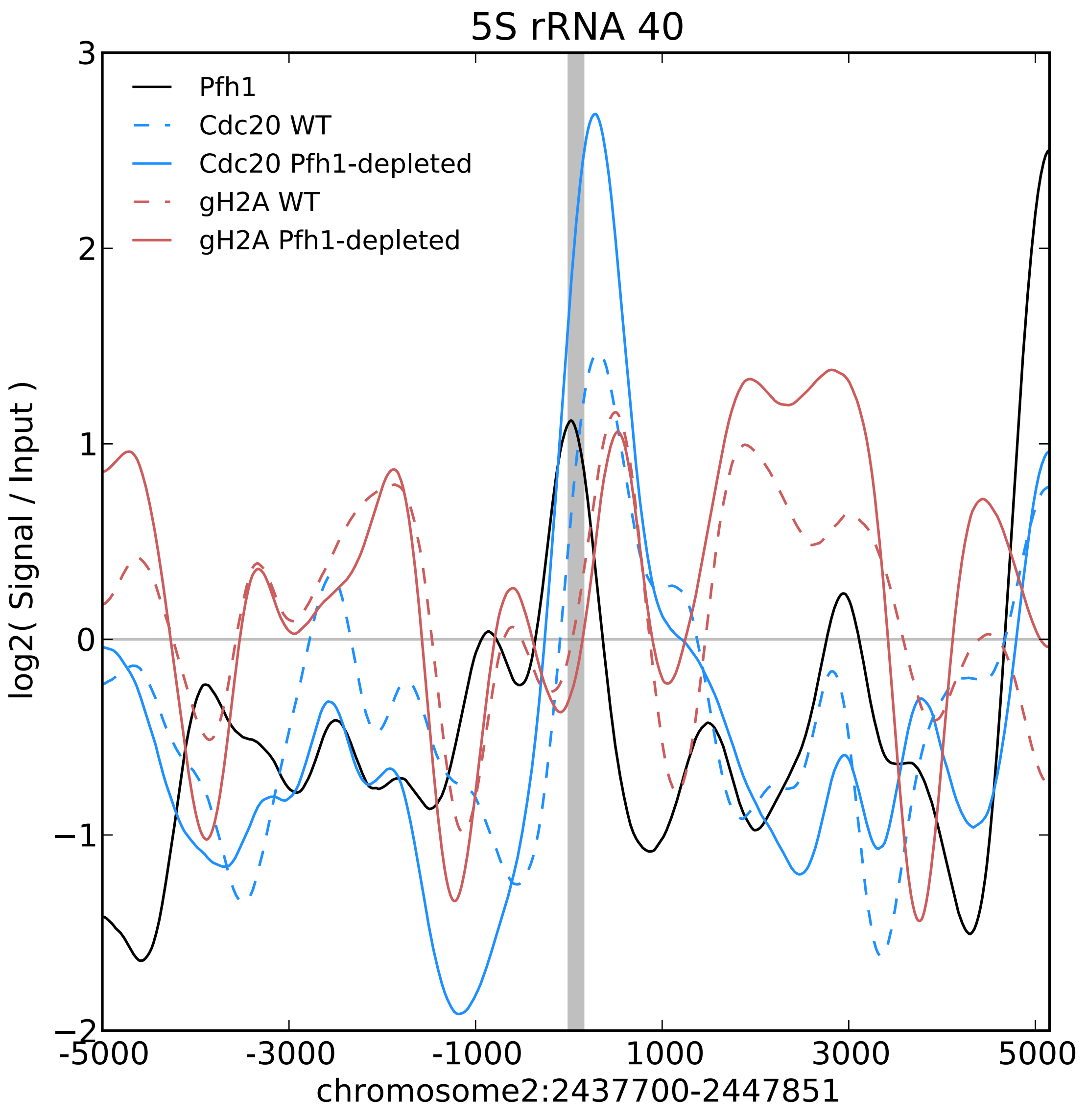

5S rRNA 40
Pfh1
Cdc20 WT
Cdc20 Pfh1-depleted
gH2A WT
gH2A Pfh1-depleted
log2(Signal / Input)
3
2
1
0
−1
−2
-5000
-3000
-1000
1000
3000
5000
chromosome2:2437700-2447851

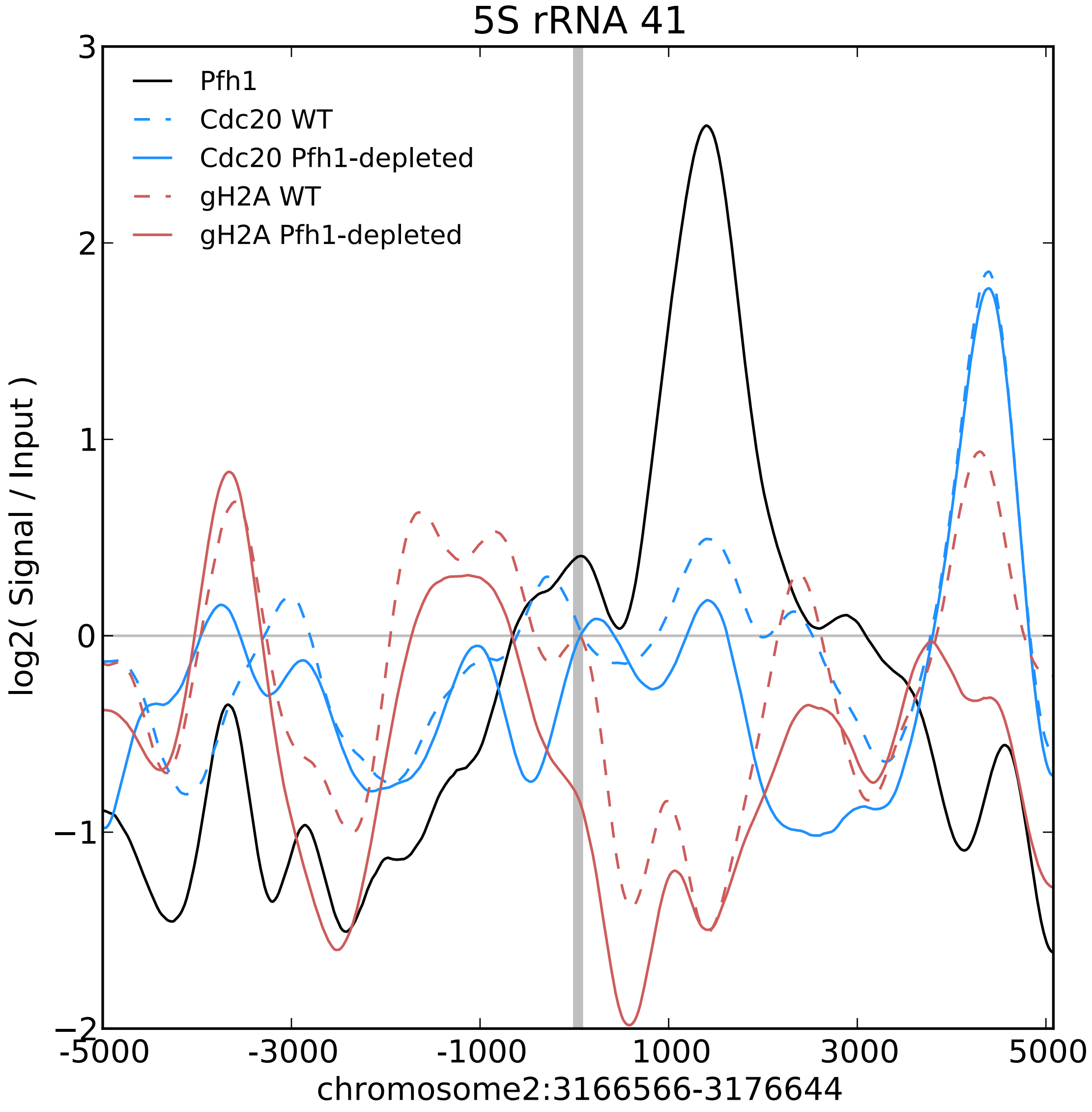
5S rRNA 41
Pfh1
Cdc20 WT
Cdc20 Pfh1-depleted
gH2A WT
gH2A Pfh1-depleted
log2(Signal / Input)
3
2
1
0
−1
−2
-5000
-3000
-1000
1000
3000
5000
chromosome2:3166566-3176644
