# Supplementary material for: Pfh1 Is an Accessory Replicative Helicase that Interacts with the Replisome to Facilitate Fork Progression and Preserve Genome Integrity
Source: PLoS Genet. 2016 Sep 9;12(9):e1006238. doi: 10.1371/journal.pgen.1006238 (PMC5017727; doi:10.1371/journal.pgen.1006238)
Supplement: S8 Table — (DOCX) [file pgen.1006238.s015.docx]

**Supplementary Table 8**. *S. pombe* strains used in this study

| Strain | Mating type | Genotype | Source |
| --- | --- | --- | --- |
| YNS103 | h- | *cdc20+::cdc20-3HA-kanmx6 leu1-32::PJK148-Pfh1-13MYC-kanmx6 cdc25-22* | This study |
| YNS76 | h+ | *ade6-M210 leu1-32 cdc25-22* | N.Walworth lab |
| YSP398 |  | *leu1-32::leu1-pfh1-GFP chk1-3HA-kanMX6 his3-D1 ura4-D18* | Pinter et al. 2008 |
| YKM333 | h+ | *ade6-M210 leu1-32::pJK148-pfh1-GFP cdc25-22 his3^+^ ura4^+^* | This study |
| YKM346 | h+ | *ade6-M210 leu1-32::pJK148-kanMX6-P3nmt-GFP-NLS cdc25-22 his3^+^ ura4^+^* | This study |
