# Supplementary material for: Pfh1 Is an Accessory Replicative Helicase that Interacts with the Replisome to Facilitate Fork Progression and Preserve Genome Integrity
Source: PLoS Genet. 2016 Sep 9;12(9):e1006238. doi: 10.1371/journal.pgen.1006238 (PMC5017727; doi:10.1371/journal.pgen.1006238)
Supplement: S9 Table — (DOCX) [file pgen.1006238.s016.docx]

| **Name of oligo** | **Sequence 5'-3'** | **Direction** |
| --- | --- | --- |
| ars3002_F | CGCTAAACAATCTCTGAATAC | Forward |
| ars3002_R | TTTACGATATATTTCCACAGC | Reverse |
| ars3002_18kb_F | GCTAACGCCTTATCCTACTTG | Forward |
| ars3002_18kb_R | TCCAACCAAAGTCAGAAAGC | Reverse |
| ars3002_30kbF | ATTGCCAAGCCTTCCAGTTC | Forward |
| ars3002_30kbR | CTTGTGACTAAATTGCTCTTGAATAG | Reverse |
| ars2004_F^1^ | TTGCTTATCTTTTGGGTAGTTTTCG | Forward |
| ars2004_R^1^ | CTTACATTTTCGGGAACTTATTAGTCAA | Reverse |
| ars2004_30kb_F^2^ | TACGCGACGAACCTTGCATAT | Forward |
| ars2004_30kb_R^2^ | TTATCAGACCATGGAGCCCATT | Reverse |
| ars3005_F^3^ | ATCCACGCCAGAGTGCTT | Forward |
| ars3005_R^3^ | TTGTTGGATGGCAATAAGAGG | Reverse |
| ars3005_26kb_F^3^ | ATAAGCACTTCGTGGGGCAA | Forward |
| ars3005_26kb_R^3^ | CCGGCCATGAAACCTACTGT | Reverse |
| Chr II_nonars_1236154_F | TACGTTCGGGCGAATTTCCA | Forward |
| Chr II_nonars_1236154_R | TAATGCTCAACTGCTGCCCA | Reverse |
| Chr II_nonars_1272741_F | GAATTGGCACCGCCAAAGTT | Forward |
| Chr II_nonars_1272741_R | GAAACCGGTCGACAAAACCG | Reverse |
| tRNAGLU.05_F^4^ | CAAAGATTGGGATGCTATGACAC | Forward |
| tRNAGLU.05_R^4^ | TGCGTACAAGACTTGGATGAG | Reverse |
| tRNAASN.05_F | GTAAGGGATTGTTGTAAGGACTG | Forward |
| tRNAASN.05R | CGAACCGAATGGGAAAGACC | Reverse |
| tRNAASN.06_6kb_F | ACTCCGTGATACCAGGTCCA | Forward |
| tRNAASN.06_6kb_R | TCGTGGAGTCAGGAGGTGAT | Reverse |
| Hsp90_F | CGTGCCATTCTTTTCGTCCC | Forward |
| Hsp90_R | ACACGGCGAACATAGAGCTT | Reverse |
| Tdh1_F | TAAGAAACGTCGGGTGGGTG | Forward |
| Tdh1_R | TGCAAACGACAACGACTCCT | Reverse |
| Adh1_F | CCCATGCTACCATCATCCCC | Forward |
| Adh1_R | GCCGACCTTGGATTCCTTCA | Reverse |
| Hta1_F^4^ | GGAACAACACCACCTTGAGCAATG | Forward |
| Hta1_R^4^ | CCGTCATCTTCAACTCGCCATCC | Reverse |
| Ade6_F^4^ | TTAAGCTGAGCTGCCAAGGT | Forward |
| Ade6_R^4^ | GGCTGCCTCTACCATCATTC | Reverse |

**Supplementary Table 9**. Oligonucleotides used for qPCR experiments.

^1^ oligonucleotide sequence from Wu and Nurse. Cell, 2009.

^2^ oligonucleotide sequence from Moser et al. EMBO J, 2009.

^3^ oligonucleotide sequence from Tapia-Alveal and O’Connell. MBoC, 2011.

^4^ oligonucleotide sequence from Sabouri et al. Genes & Dev., 2012.
